# TiCl_3_‑Mediated Reductive Aminohydroxylation of Alkenes to (Benzo)Furo[3,2‑*b*]Indolines

**DOI:** 10.1021/acs.orglett.6c01004

**Published:** 2026-04-02

**Authors:** Dina Boyarskaya, Paul Gri, Bastien Delayre, Qian Wang, Jieping Zhu

**Affiliations:** Laboratory of Synthesis and Natural Products (LSPN), Institute of Chemical Sciences and Engineering, Ecole Polytechnique Fédérale de Lausanne, EPFL-SB-ISIC-LSPN, BCH 5304, 1015 Lausanne, Switzerland

## Abstract

We report a TiCl_3_-mediated reductive double cyclization of *ortho*-nitroaryl-substituted styrene and stilbene derivatives that enables efficient access to dihydrofuro­[3,2-*b*]­indolines and benzofuro­[3,2-*b*]­indolines under mild conditions. The transformation proceeds through a single-electron transfer (SET) reduction of the nitro group to a nitroso intermediate, followed by a concerted 5-center, 6π-electrocyclization to generate a nitrone. Intramolecular trapping of this nitrone by a tethered hydroxy or phenol group forms a cyclic hydroxylamine, which undergoes further SET reduction to furnish the fused indoline products. This domino reductive cascade provides a mechanistically distinct and complementary alternative to established oxidative methods for constructing [3,2-*b*] fused indoline architectures.

## Graphical abstract

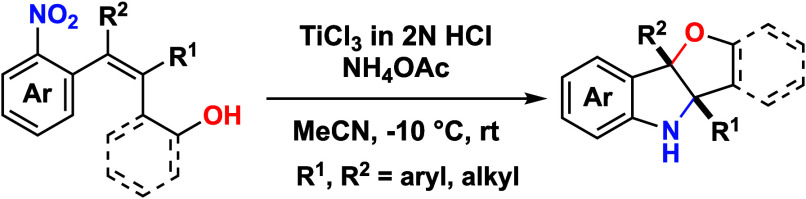

Furoindoline architectures constitute a prominent structural class in indole alkaloids, yet their two possible regioisomeric fusion patterns display a striking imbalance in both natural occurrence and synthetic accessibility. Dihydrofuro­[2,3-*b*]­indolines **1** and benzofuro­[2,3-*b*]­indolines **2** are structural motifs found in the akuammiline family of indole alkaloids, including aspidophylline A (**3**, Scheme 1a),^1,2^ as well as in cyclic peptides such as diazonamide and azonazine.^3^ By contrast, the regioisomeric dihydrofuro­[3,2-*b*]­indolines **4** and benzofuro­[3,2-*b*]­indolines **5** are rarely encountered in Nature and to date, phalarine (**6**)^4^ and cymoside^5^ remain, to the best of our knowledge, the only alkaloids featuring this structural motif (Scheme 1b). The distinctive propeller-like architecture of phalarine has stimulated sustained synthetic efforts, with particular emphasis on forging the benzofuro­[3,2-*b*]­indoline core.^6–8^

**Scheme 1:**
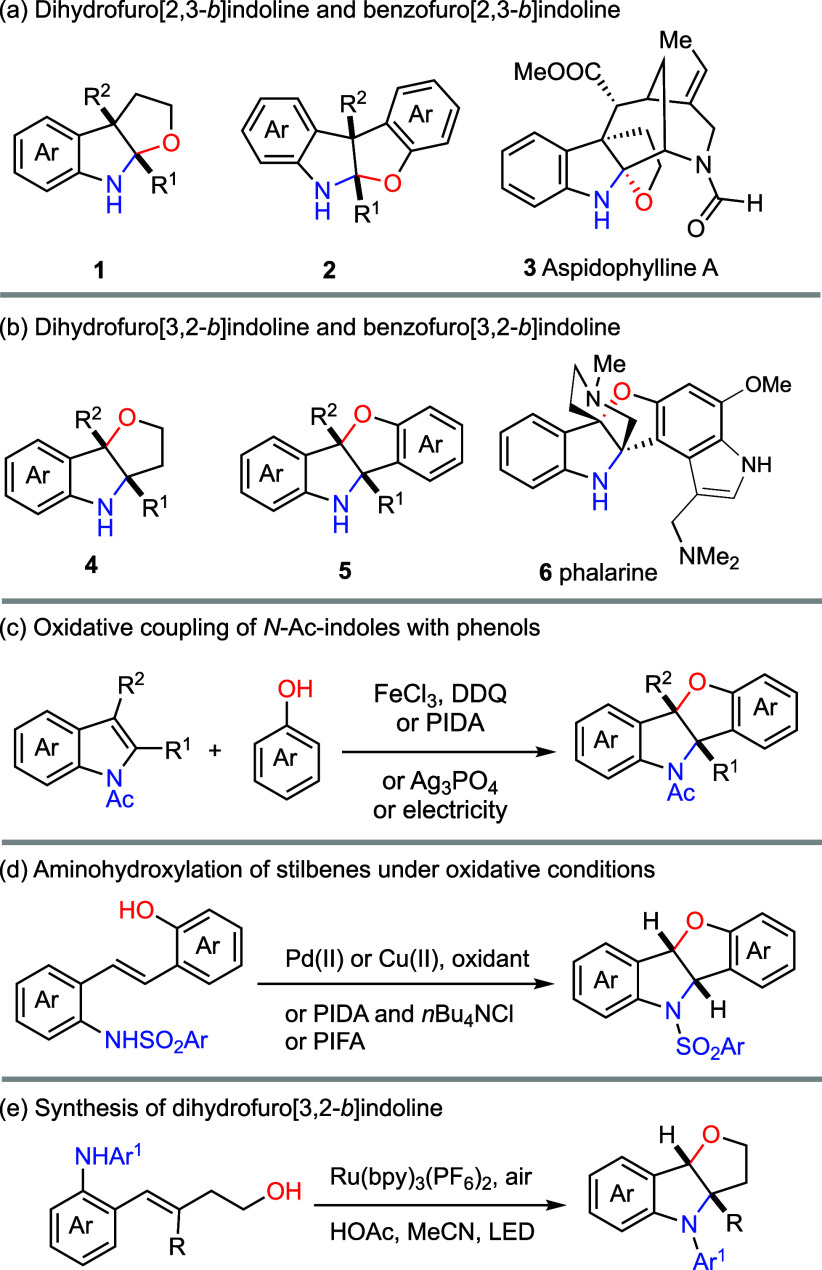
(Benzo)­Furoindolines: Structures and Syntheses^a^

a Abbreviations: PIDA = phenyliodine diacetate; PIFA = phenyliodine bis­(trifluoroacetate); DDQ = 2,3-dichloro-5,6-dicyano-1,4-benzoquinone; bpy = 2,2-bipyridine.

The pronounced disparity between the two isomeric furoindoline scaffolds highlights a fundamental challenge in controlling oxidative indole–phenol cross coupling to favor the less-accessible [3,2-*b*] fusion mode. Early attempts to mimic the proposed biosynthetic oxidative coupling of indoles with phenols predominantly delivered benzofuro­[2,3-*b*]­indolines **2** in accordance with the inherent reactivity of the two coupling partners.^9^ A significant advance was achieved by Vincent and co-workers, who demonstrated that *N*-acylation of indoles can override this innate reactivity preference, diverting the oxidative coupling toward the regioselective formation of benzofuro­[3,2-*b*]­indolines **5** (Scheme 1c).^10^ Subsequent developments established electrochemical oxidation,^11^ PIDA,^8^ and Ag_3_PO_4_ ^12^ as effective mediators of this transformation and this strategy was later implemented as a key step in Jia’s total synthesis of phalarine (**6**).^8^

Complementary approaches to benzofuro­[3,2-*b*]­indolines **5** have also been developed. Intramolecular aminohydroxylation of stilbenes provides direct access to this framework, and both transition-metal-catalyzed (Pd,^13^ Cu^14^) and hypervalent iodine-mediated variants^15^ have been reported (Scheme 1d). The latter strategy was notably featured in Chen’s synthesis of phalarine (**6**).^6,16^ In addition, Zheng disclosed a photoredox-catalyzed construction of dihydrofuro­[3,2-*b*]­indolines **4** ^17^ further expanding the methodological repertoire for assembling this synthetically demanding motif.^18^

A notable limitation common to these approaches is the requirement for nitrogen protection. In most cases, the requisite anilides or sulfonamides are prepared from the corresponding nitroarenes through a two-step sequence involving nitro reduction followed by *N*-protection of the resulting aniline. This detour not only reduces step economy but also obscures the possibility of exploiting the intrinsic reactivity of the nitro group. Moreover, no unified strategy has been established that enables access to both dihydrofuro­[3,2-*b*]­indolines **4** and benzofuro­[3,2-*b*]­indolines **5** from a common precursor class.

Building on these considerations, we questioned whether a reductive cyclization strategy could bypass preformed anilides or sulfonamides and enable a unified entry to both [3,2-*b*]-fused products. Our previous studies established that aqueous TiCl_3_ (in 2 N HCl) efficiently promotes the Cadogan–Sundberg reaction^19,20^ at room temperature (Scheme 2a),^21^ a protocol validated in the total synthesis of complex indole alkaloids, including (+)-1,2-dehydroaspidospermidine, (+)-condyfoline, and (−)-tubifoline.^21^ These precedents led us to hypothesize that a TiCl_3_-mediated reductive cyclization of *ortho*-nitro-substituted styrene or stilbene derivative (**7** and **8**) might provide a unified approach to dihydrofuro­[3,2-*b*]­indoline **4** and benzofuro­[3,2-*b*]­indoline **5**, respectively. The hypothetic reaction pathway is outlined in Scheme 2b. Partial reduction of nitro to nitroso group followed by a 5-center-6π-electrocyclization^22^ would generate nitrone intermediate **9** from precursor **7** or **8**. A subsequent cyclization would furnish **10**, which upon reduction of the hydroxylamine function would provide **4** or **5**, depending on the nature of the tether connecting the hydroxyl group and the Csp^2^ carbon. However, intermediate **9** could undergo 1,2-Wagner–Meerwein rearrangement to afford either **11** (pathway b) or **12** (pathway c).^21b,23^ Intermediate **11** could be further reduced to the 2,3,3-trisubstituted indolenine **13**, whereas **12** could evolve to **1** or **2** via a sequence of cyclization and reduction. Thus, precise control of the reaction conditions is critical to override rearrangement pathways and enforce the selective formation of the kinetically programmed [3,2-*b*] framework. Herein, we report that TiCl_3_-mediated reductive aminohydroxylation of nitroarene derivatives **7** or **8** proceeded smoothly to afford **4** or **5** in good to excellent yields.

**Scheme 2:**
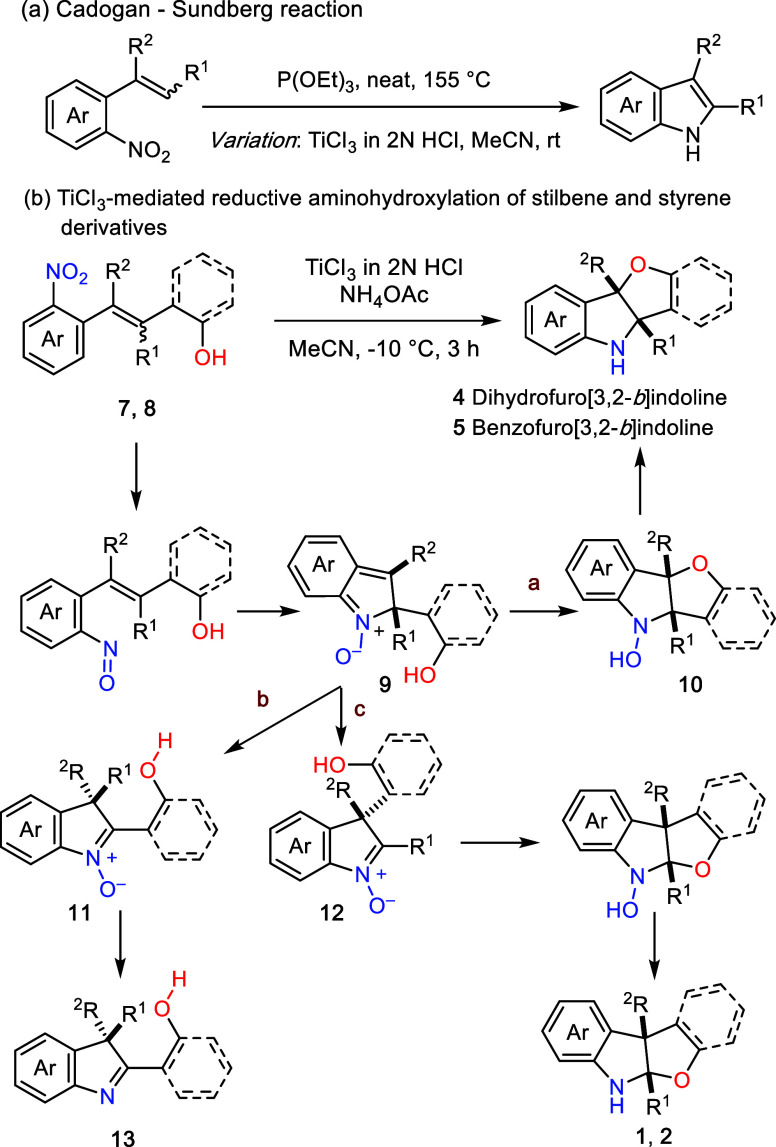
Reductive Aminohydroxylation of Stilbene and Styrene Derivatives

(*E*)-4-(2-nitrophenyl)-3,4-diphenylbut-3-en-1-ol (**7a**, R^1^ = R^2^ = Ph) was selected as the model substrate for the evaluation of reaction conditions. The following key experimental observations emerged (see the Supporting Information (SI) for details): (a) the solvent (MeCN) needed to be carefully degassed to prevent the cleavage of the double bond; (b) addition of ammonium salt to attenuate the acidity of the reaction mixture proved beneficial, with a 1:4 ratio of TiCl_3_ (2N in HCl)/NH_4_OAc identified as optimal; and (c) the reaction was best performed at −10 °C to suppress overreduction of nitroarene to the corresponding aniline.^24^ Overall, the optimal conditions involved slow addition of degassed aqueous TiCl_3_ (15.0 equiv) to a solution of **7a** and NH_4_OAc (60.0 equiv) in degassed MeCN (*c* 0.05 M) at −10 °C, followed by stirring at the same temperature for 3 h. Under these conditions, the desired dihydrofuro­[3,2-*b*]­indoline **4a** was isolated in 59% yield (Scheme 3).

**Scheme 3:**
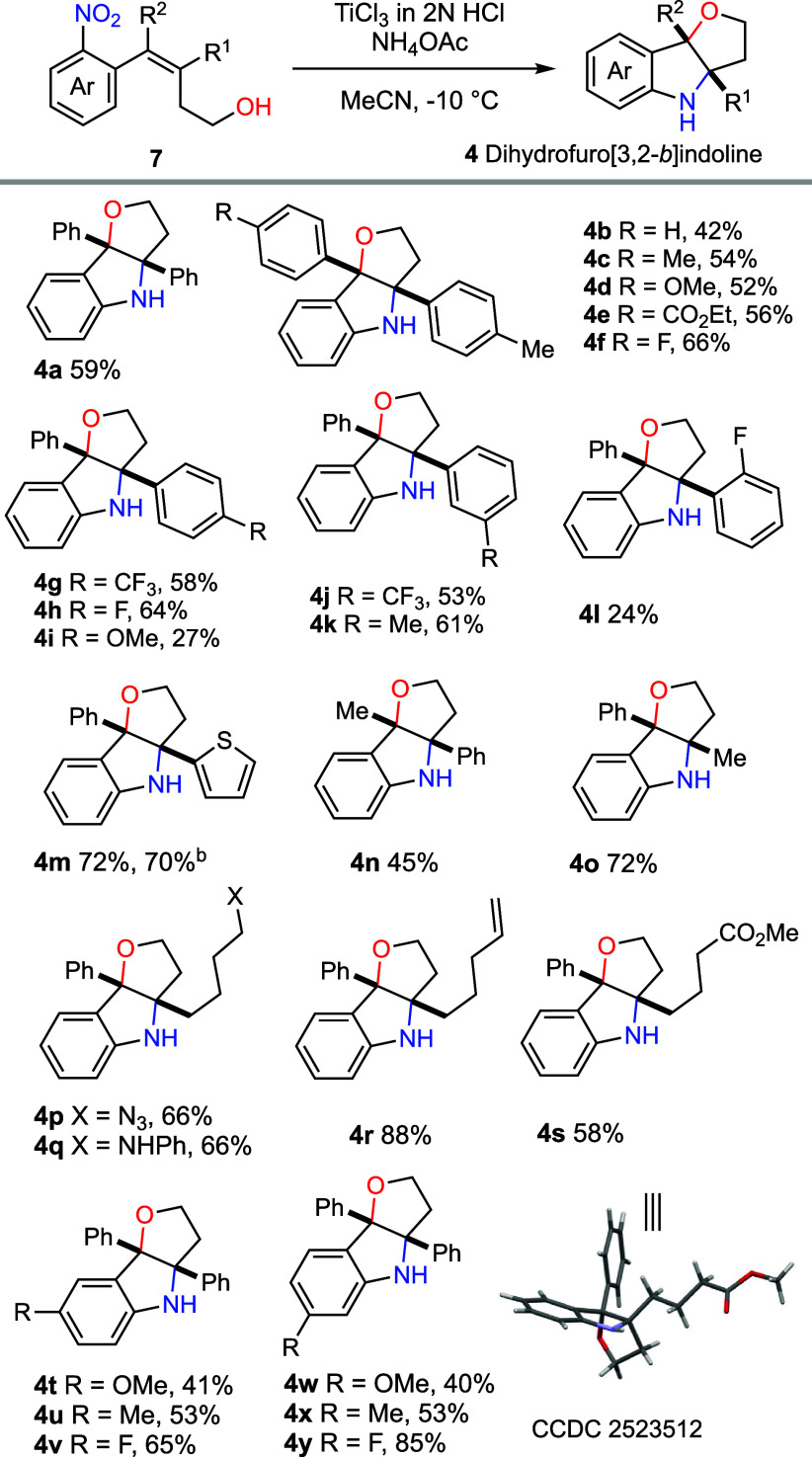
Reductive Aminohydroxylation of Styrene Derivatives^a^

a Standard conditions: **7** (0.1 mmol), TiCl_3_ (15.0 equiv), NH_4_OAc (60 equiv), degassed MeCN (1.0 mL, *c* 0.05), −10 °C. Yield refers to isolated pure product. ^*b*^Reaction performed at 2.4 mmol scale.

With the optimized conditions in hand, the scope of this reaction was examined. The starting materials were prepared by Suzuki–Miyaura cross-coupling between enol tosylates and arylboronates according to Gosselin (see the SI).^25^ As shown in Scheme 3, a wide range of aryl substituents at the C3 and C4 positions of 4-(2-nitrophenyl)­but-3-en-1-ol were well-tolerated. Substrates bearing either electron-donating (Me, OMe) or electron-withdrawing groups (CO_2_Et, F, CF_3_), at the *para*- or *meta*-position reacted smoothly to furnish the corresponding tricyclic products. In contrast, introduction of an *ortho* substituent on the aryl ring significantly diminished the yield of the desired product (**4l**). A similar decrease in efficiency was observed for substrate **4i**, bearing a 4-methoxyphenyl group at the C3 position, presumably due to competitive side reactions outlined in Scheme 2b. Notably, a thiophene substituent was compatible with the reaction conditions affording **4m** in 72% yield.

Alkyl substituents at the C3 and C4 positions bearing different functional groups (N_3_, aniline, alkene and CO_2_Me) underwent smooth double cyclization to deliver the desired products (**4n**, **4o**, **4p**–**4s**). Furthermore, both electron-donating and electron-withdrawing substituents on the nitroarene moiety were well-tolerated, providing the corresponding products (**4t**–**4y**). In all cases, anilines arising from complete reduction of the nitro group were isolated as minor byproducts, while the double bond geometry of **7** had no discernible effect on the reaction outcome.

Gratifyingly, performing the reductive aminohydroxylation of alkene **7m** on 2.4 mmol scale afforded **4m** in 70% yield, highlighting the practicality and scalability of this protocol.

We next turned our attention to the reductive aminohydroxylation of stilbene derivatives (Scheme 4). The reaction conditions were reoptimized and under optimized conditions [TiCl_3_ (20.0 equiv), Me_2_CO-H_2_O (v/v = 1:1), rt, 16 h], **8a** (R^1^ = *n*Bu, R^2^ = Ph) underwent smooth double cyclization to afford benzofuro­[3,2-*b*]­indolines **5a** in 69% yield. Tetracyclic compounds **5b** and **5c** were similarly prepared in yields of 89% and 73%, respectively. Interestingly, reaction of **8d** under the same conditions produced two separable compounds **5d** (42%) and **2d** (40%), in approximately 1:1 ratio. While compound **5d** was generated following the expected pathway involving the cyclization of the nitrone intermediate **9d**, the formation of **2d** can be rationalized by a Wagner–Meerwein rearrangement of **9d**, followed by cyclization of the resulting 3,3-disubstituted indolenine *N*-oxide **12d** and subsequent reduction (cf Scheme 2b, pathway c). The high stability of the nitrone intermediate, due to the 4-methoxyphenyl group at the C3 position of **9d**, together with the high migratory aptitude of *ortho*-hydroxyphenyl group, renders this competing pathway significant.

**Scheme 4:**
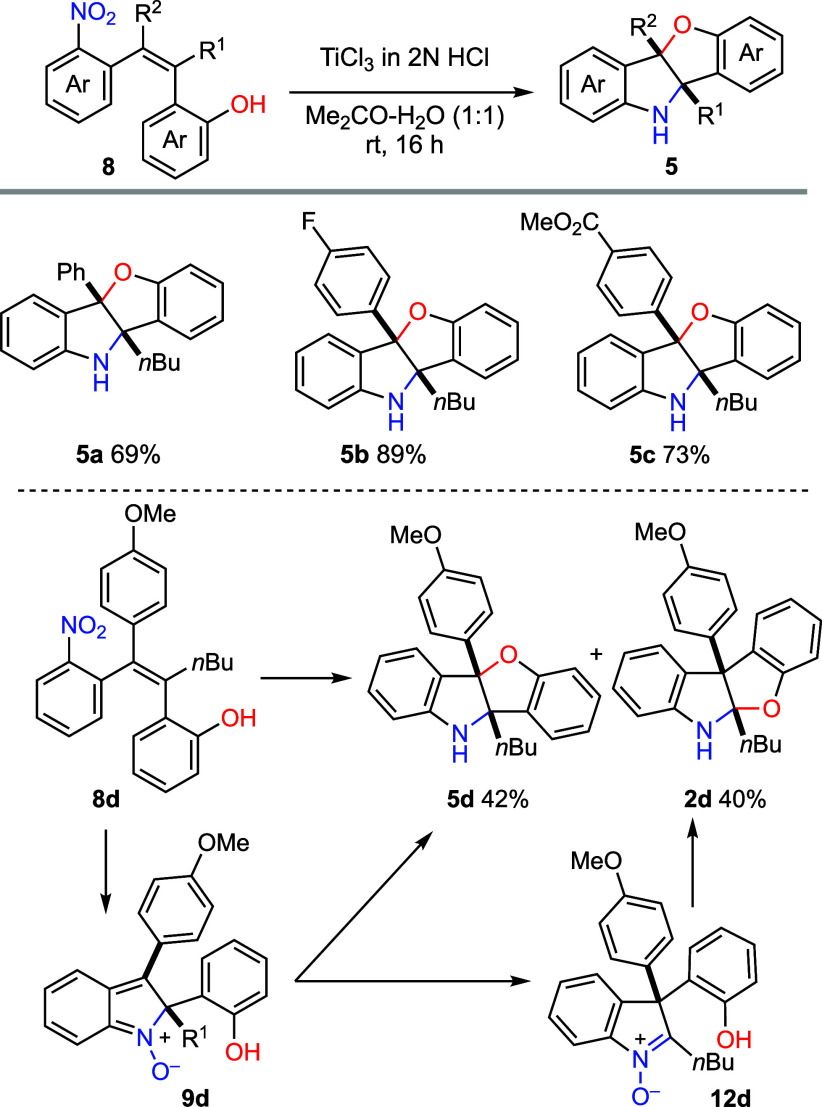
Reductive Aminohydroxylation of Stilbene Derivatives^a^

a Standard conditions: **8** (0.1 mmol), TiCl_3_ (20.0 equiv), Me_2_CO–H_2_O (1:1, *c* 0.05), room temperature (rt). Yield refers to isolated pure product.

A triple cyclization was subsequently developed (Scheme 5). Treatment of an acetonitrile solution of **7z** with TiCl_3_ and NH_4_OAc afforded tetracyclic compound **4z** in 51% yield. This domino transformation proceeds through the sequential formation of two C–N bonds and one C–O bond, illustrating the power of orchestrated multiple bond-forming processes. Notably, all three rings are constructed in a single operation, highlighting the synthetic advantage of the present reductive double-cyclization protocol. Such a transformation would be unattainable under previously reported oxidative conditions, which rely on protected aniline as one of the internal nucleophiles.

**Scheme 5:**
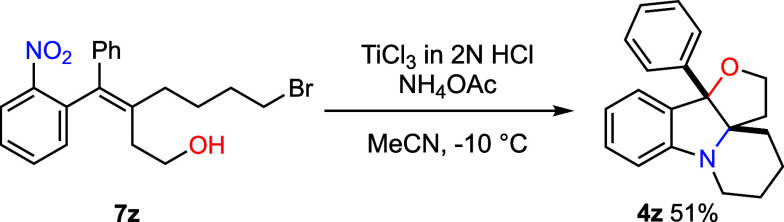
Triple Cyclization Leading to Hexahydro-Furo­[3,2-*b*]­pyrido­[1,2-*a*]­indole

By carefully monitoring the reaction progress, we were able to isolate hydroxylamine **10y**. Subsequent reduction of **10y** under standard conditions proceeded smoothly to furnish **4y**, supporting the notion that **10y** served as an intermediate in the reductive aminohydroxylation process (Scheme 6a). An additional control experiment indicated that dihydrofuro­[3,2-*b*]­indoline **4a** undergoes further reduction with aqueous TiCl_3_ at room temperature to afford the 2,2,3-trisubstituted indoline **14**, a structural motif that, although rare, is found in indole alkaloids (Scheme 6b).^26^ Consistent with this observation, compound **14** was also formed when the reductive cyclization of **7a** was conducted at room temperature with extended reaction time. Mechanistically, single electron transfer (SET) reduction of **4a**, followed by protonation of the alkoxide, would generate the stabilized radical **15**, which underwent a second SET to give intermediate **16**. Protonation of **16** would then afford the observed product **14**.

**Scheme 6:**
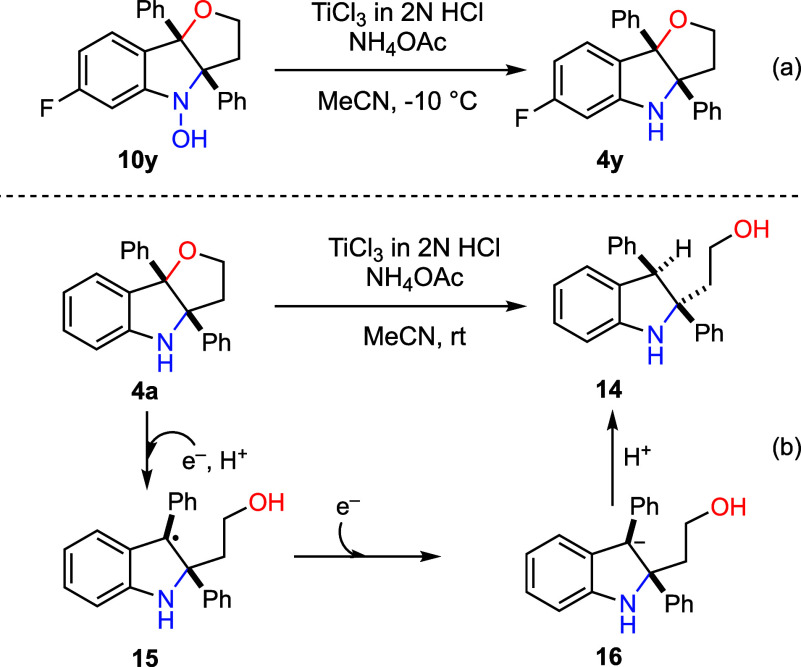
Isolation of Hydroxylamine and Its Further Reduction

In summary, we have developed a TiCl_3_-mediated reductive cyclization of *ortho*-nitroaryl substituted styrene and stilbene derivatives for the synthesis of dihydrofuro­[3,2-*b*]­indolines and benzofuro­[3,2-*b*]­indolines. The reductive strategy complements previously reported oxidative protocol, providing a practical and mechanistically distinct route to these tricyclic and tetracyclic frameworks.

## Supplementary Material



## Data Availability

The data underlying this study are available in the published article and its Supporting Information.

## Notes

Experimental procedures and characterization data, additional experimental details, crystallographic data of **4s**, copies of ^1^H and ^13^C NMR spectra (PDF)
