# Supplementary material for: TiCl3‑Mediated Reductive Aminohydroxylation of Alkenes to (Benzo)Furo[3,2‑b]Indolines
Source: Org Lett. 2026 Apr 2;28(15):4941–6. doi: 10.1021/acs.orglett.6c01004 (PMC13097258; doi:10.1021/acs.orglett.6c01004)

Supporting information

$TiCl_3$–Mediated Reductive Aminohydroxylation of Alkenes to (Benzo)Furo[3,2-*b*]Indolines

Dina Boyarskaya,† Paul Gri,†Bastien Delayre, Qian Wang, Jieping Zhu*

Laboratory of Synthesis and Natural Products (LSPN), Institute of Chemical Sciences and Engineering, Ecole Polytechnique Fedérale de Lausanne, EPFL-SB-ISIC-LSPN, BCH 5304, 1015 Lausanne, Switzerland

*Correspondence to: jieping.zhu@epfl.ch

1) General information 2

2) Screening of conditions for the reductive cyclization of *o*-nitrostyrene 7a 3

3) Synthesis of the starting materials – general procedure and characterization data 4

3.1) General procedure A for the alkylation of ketones 4

3.2) General procedure B for the synthesis of enol tosylates **22** 11

3.3) General procedure C for the synthesis of alcohols **7** 18

3.4) Procedure for the synthesis of substrates **7p,q,r,s,z** 28

3.6) General procedure E for the reductive cyclization of *o*-nitrostyrenes 40

4) References 56

5) X-Ray Crystallographic data 57

6) Copies of NMR spectra 78

1) General information

Reagents and solvents were purchased from commercial sources and preserved under argon. More sensitive compounds were stored in a desiccator or in a glove-box if required. Reagents were used without further purification unless otherwise noted. All reactions were performed under argon (or nitrogen) and with stirring unless otherwise noted. When needed, glassware was dried for at least overnight in an oven (170 °C) or under vacuum with a heat gun (650 °C).

Solvents indicated as dry were either purchased as such, distilled prior to use or dried by a passage through a column of anhydrous alumina or copper using a Puresolv MD 5 from Innovative Technology Inc., based on the Grubbs' design. Flash column chromatography was performed using Silicycle SiliaFlash® P60 230- 400 mesh. Reactions were monitored using Merck Kieselgel 60F254 aluminum or glass backed plates. TLC spots were visualized by UV fluorescence (254 nm) then one of the following stains: $KMnO_4$, phosphomolybdic acid, ninhydrin, pancaldi, *p*-anisaldehyde, vanillin.

NMR spectra were recorded on a Brüker AvanceIII-400, Brüker Avance-400 Brüker DPX-400 spectrometer at room temperature. The ^{1}H frequency is at 400.13 MHz and the ^{13}C frequency is at 100.62 MHz. Chemical shifts (δ) were reported in parts per million (ppm) relative to residual solvent peaks rounded to the nearest 0.01 for ^{1}H and 0.1 for ^{13}C (ref: $CHCl_3$ [^{1}H: 7.26, ^{13}C: 77.2] and CD_2HOD [^{1}H: 3.34, ^{13}C: 49.9]). Coupling constants (*J*) were reported in Hz to the nearest 0.1 Hz. Peak multiplicities were indicated as follows: s (singlet), d (doublet), t (triplet), q (quartet), p (pentet), sext (sextet), hept (heptet), m (multiplet) and br (broad). Assignments were based on peak multiplicities and integrals. COSY, HSQC, HMBC and NOESY experiments were performed when necessary to confirm the assignments.

IR spectra were recorded using a Jasco FT/IR-4100 spectrometer outfitted with a PIKE technology MIRacleTM ATR accessory as neat films compressed onto a Zinc Selenide window or using a Perkin Elmer Spectrum BX FTIR spectrometer. The spectra are reported in cm^{-1}. Abbreviations used are: w (weak), m (medium), s (strong) and br (broad).

Mass spectra were recorded on a Waters ACQUITY H-class UPLC/MS ACQ-SQD using electron ionization (EI positive and negative) or on a Finnigan TSQ7000 using electrospray ionization (ESI+). Accurate mass measurements were performed by the mass spectrometry service of the EPFL using ESI-TOF on a QTOF Ultima from Waters.

Melting points were determined using a Stuart SMP30 or Büchi B-540 melting point apparatus.

X-ray structures were determined with a SuperNova, Dual, Cu at home/near, Atlas diffractometer operating at T = 140.00(10) K or XtaLAB Synergy R, DW system, HyPix-Arc 150 diffractometer operating at T = 139.99(10) K. Data were measured using ω scans using Cu or Mo Kα radiation. The diffraction pattern was indexed and the total number of runs and images was based on the strategy calculation from the program CrysAlisPro. The unit cell was refined using CrysAlisPro. Data reduction, scaling and absorption corrections were performed using CrysAlisPro. A gaussian absorption correction was performed using CrysAlisPro. The numerical absorption correction was based on gaussian integration over a multifaceted crystal model. The empirical absorption correction was carried out using spherical harmonics, implemented 162 in SCALE3 ABSPACK scaling algorithm. The structure was solved and the space group determined by the ShelXT structure solution program using dual methods and refined by full matrix least squares minimisation on F 2 using ShelXL. All non-hydrogen atoms were refined anisotropically. Hydrogen atom positions were calculated geometrically and refined using the riding model.

2) Screening of conditions for the reductive cyclization of *o*-nitrostyrene **7a**

Table S1: Screening of conditions for the reductive cyclization of *o*-nitrostyrene **7a**

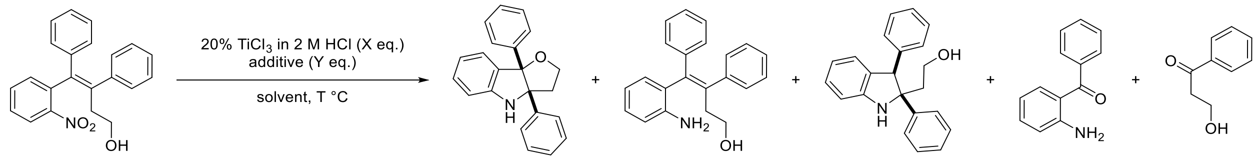

7a **4a** **32** **14** **A** **B**

Entry [a]	$TiCl_3$	Additive (eq.)	Solvent (M)	Temperature	**4a**	**32**	**14**	**A+B**
1	10 eq.	-	Acetone (0.05)[b]	rt	-	36%	-	38%
2	10 eq.	HCO_2Na (40)	Acetone (0.1)[b]	rt	-	32%	-	62%
3	10 eq.	HCO_2Na (40)	MeCN (0.1)[b]	rt	-	-	-	90%
4	10 eq.	NH_4OAc (40)	MeCN (0.1)[b]	rt	-	30%	-	60%
5	10 eq.	-	Acetone (0.1)	rt	-	17%	-	-
6	10 eq.	-	MeCN (0.1)	rt	-	29%	-	-
7	10 eq.	NH_4OAc (20)	MeCN (0.1)	rt	-	25%	-	-
8	10 eq.	NH_4OAc (60)	MeCN (0.1)	rt	10%	58%	30%	-
9	10 eq.	NH_4OAc (80)	MeCN (0.1)	rt	15%	57%	30%	-
10	10 eq.	NH_4OAc (40)	MeCN (0.05)	rt	15%	20%	10%	-
11	20 eq.	NH_4OAc (80)	MeCN (0.05)	rt	21%	30%	30%	-
12	50 eq.	NH_4OAc (200)	MeCN (0.01)	rt	16%	38%	42%	-
13	20 eq.	NH_4OAc (100)	MeCN (0.05)	rt	12%	45%	28%	-
14	20 eq.	NH_4OAc (80)	MeCN (0.01)	rt	-	14%	4%	-
15	20 eq.	NH_4OAc (160)	MeCN (0.01)	rt	24%	33%	35%	-
16	20 eq.	NH_4HCO_2 (80)	MeCN (0.01)	rt	-	24%	-	-
17	20 eq.	NH_4Cl (80)	MeCN (0.01)	rt	-	50%	-	-
18	20 eq.	NaI (80)	MeCN (0.01)	rt	-	98%	-	-
19	20 eq.	NaOAc (80)	MeCN (0.01)	rt	15%	65%	13%	-
20	20 eq.	NH_4OAc (80) + SDS (2)	MeCN (0.01)	rt	22%	30%	25%	-
21	20 eq.	NH_4OAc (80)	MeCN (0.05)	0 °C	43%	20%	5%	-
22	20 eq.	NH_4OAc (80)	MeCN (0.05)	-10 °C	52%	40%	-	-
23	20 eq.	NH_4OAc (80)	MeCN (0.05)	-20 °C	36%	31%	-	-
**24**	**15 eq.**	**NH_4OAc (60)**	**MeCN (0.05)**	**-10 °C**	**63%**	**25%**	**-**	**-**
25	15 eq.	NH_4OAc (60)	MeCN (0.05)	-20 °C	50%	39%	-	-
26	15 eq.	NBu_4OAc (60)	MeCN (0.05)	-10 °C	-	98%	-	-
27	15 eq.	Et_3N (60)	MeCN (0.05)	-10 °C	-	91%	-	-

[a] All the reactions were performed on a 0.1 mmol scale according to the following procedure: In a 10 mL pressure resistant round bottom flask was charged the *o*-nitrostyrene **A** (1 equiv), the additive (unless noted otherwise) and MeCN (unless other solvents were tested, entries 1, 2 and 5). Then $TiCl_3$ (1.3 M solution in HCl, X equiv) was added dropwise at the corresponding temperature over 5 min. The reaction mixture was warmed to rt (unless entries 21-27) and stirred for 3 h. After completion of the reaction, an aqueous $NaHCO_3$ solution was added slowly at 0 °C to quench the reaction. The mixture was extracted with EtOAc. The combined organic layers were washed with brine, dried, filtered and concentrated under reduced pressure. Yields were determined by ^1H NMR with an internal standard (1,3,5-trimethoxybenzene – 1 eq.). [b] Solvents were not degassed by freeze-pump-thaw cycles.

3) Synthesis of the starting materials – general procedure and characterization data

3.1) General procedure A for the alkylation of ketones

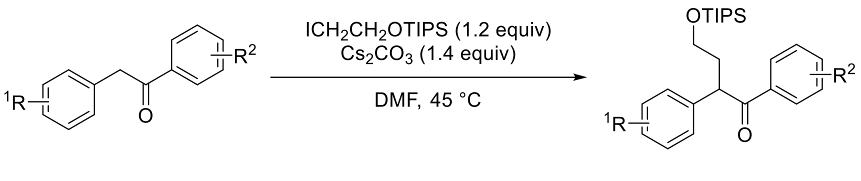

18

Glassware and stirring bar were stored in the oven and the reaction was carried out under inert atmosphere and dry conditions. To a solution of 1,2-diphenylethan-1-one (4.0 g, 20.0 mmol, 1.0 equiv) in DMF (40 mL, 0.5 M) were added Cs_2CO_3 (9.3 g, 28.6 mmol, 1.4 equiv) and (2-iodoethoxy)triisopropylsilane (8.0 g, 24.5 mmol, 1.2 equiv) sequentially. The reaction mixture was heated at 45 °C in an oil bath for 3 h. After completion of the reaction, a saturated aqueous NH_4Cl solution was added slowly at 0 °C to quench the reaction. The reaction mixture was extracted with EtOAc. The combined organic layers were washed with brine, dried, filtered and concentrated under reduced pressure. The residue was subjected to flash column chromatography on silica gel to afford the desired ketone **18a**.

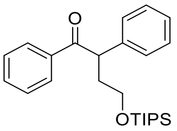

1,2-diphenyl-4-((triisopropylsilyl)oxy)butan-1-one (**18a**)

This compound was prepared following general procedure **A** from 1,2-diphenylethan-1-one (4.0 g, 20.0 mmol). Yield: 58% (3.5 g), isolated as colorless oil. Purification: Flash chromatography (PE/EtOAc, 99:1), $R_f = 0.57$ (PE/EtOAc 95:5).

^{1}H NMR (400 MHz, $CDCl_3$) δ 8.01 – 7.97 (m, 2H), 7.50 – 7.45 (m, 1H), 7.40 – 7.35 (m, 2H), 7.34 – 7.26 (m, 4H), 7.22 – 7.16 (m, 1H), 4.98 (t, J = 7.2 Hz, 1H), 3.70 (ddd, J = 10.1, 6.3, 4.9 Hz, 1H), 3.61 (ddd, J = 10.1, 7.3, 4.7 Hz, 1H), 2.48 – 2.40 (m, 1H), 2.05 – 1.96 (m, 1H), 1.09 – 1.00 (m, 21H).

^{13}C NMR (101 MHz, $CDCl_3$) δ 200.3, 139.6, 137.1, 132.9, 129.0, 128.9, 128.7, 128.6, 127.1, 60.7, 49.4, 37.1, 18.2, 12.1.

HRMS (ESI/QTOF) m/z: $[M + Na]^+$ Calcd for $C_{25}H_{36}NaO_2Si^+$ 419.2377; Found 419.2379.

IR (ν_{max}, cm^{-1}) 2946 (m), 2866 (m), 1720 (m), 1684 (m), 1460 (m), 1272 (m), 1103 (m), 883 (m), 754 (m).

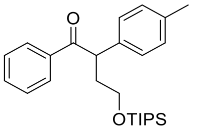

1-phenyl-2-(p-tolyl)-4-((triisopropylsilyl)oxy)butan-1-one (**18b**)

This compound was prepared following general procedure **A** from 1-phenyl-2-(p-tolyl)ethan-1-one (1.35 g, 6.4 mmol). Yield: 76% (2.0 g), isolated as colorless oil. Purification: Flash chromatography (PE/EtOAc, 98:2), $R_f = 0.91$ (PE/EtOAc 90:10).

^{1}H NMR (400 MHz, $CDCl_3$) δ 8.00 – 7.96 (m, 2H), 7.50 – 7.44 (m, 1H), 7.39 – 7.35 (m, 2H), 7.22 – 7.18 (m, 2H), 7.09 (d, J = 7.9 Hz, 2H), 4.93 (t, J = 7.1 Hz, 1H), 3.69 (ddd, J = 10.1, 6.3, 4.9 Hz, 1H), 3.62 (ddd, J = 10.1, 7.3, 4.8 Hz, 1H), 2.42 (dtd, J = 14.1, 7.2, 4.9 Hz, 1H), 2.28 (s, 3H), 1.97 (dtd, J = 13.6, 6.6, 4.7 Hz, 1H), 1.05 – 1.00 (m, 21H).

^{13}C NMR (101 MHz, $CDCl_3$): δ 200.4, 137.1, 136.7, 136.5, 132.8, 129.7, 128.9, 128.6, 128.5, 60.8, 49.0, 37.1, 21.2, 18.2, 12.1.

HRMS (ESI/QTOF) m/z: $[M + Na]^+$ Calcd for $C_{26}H_{38}NaO_2Si^+$ 433.2533; Found 433.2527.

IR (ν_{max}, cm^{-1}) 2932 (m), 2863 (m), 1676 (m), 1460 (w), 1266 (w), 1100 (m), 1064 (m), 992 (m), 800 (m).

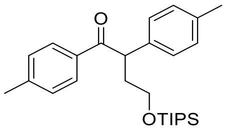

1,2-di-p-tolyl-4-((triisopropylsilyl)oxy)butan-1-one (**18c**)

This compound was prepared following general procedure **A** from 1,2-di-*p*-tolylethan-1-one (3.0 g, 13.4 mmol). Yield: 61% (3.5 g), isolated as colorless oil. Purification: Flash chromatography (PE/EtOAc, 99:1), $R_f = 0.58$ (PE/EtOAc 95:5).

^{1}H NMR (400 MHz, $CDCl_3$) δ 7.90 – 7.87 (m, 2H), 7.20 – 7.16 (m, 4H), 7.08 (d, J = 7.8 Hz, 2H), 4.90 (t, J = 6.9 Hz, 1H), 3.70 – 3.58 (m, 2H), 2.45 – 2.31 (m, 1H), 2.34 (s, 3H), 2.27 (s, 3H), 2.00 – 1.94 (m, 1H), 1.12 – 0.98 (m, 21H).

^{13}C NMR (101 MHz, $CDCl_3$): δ 200.0, 143.6, 136.8, 136.6, 134.6, 129.6, 129.2, 129.0, 128.5, 60.8, 48.8, 37.1, 21.7, 21.2, 18.2, 12.1.

HRMS (ESI/QTOF) m/z: $[M + Na]^+$ Calcd for $C_{27}H_{40}NaO_2Si^+$ 447.2690; Found 447.2695.

IR (ν_{max}, cm^{-1}) 2946 (s), 2863 (s), 1676 (s), 1608 (m), 1460 (m), 1266 (m), 1100 (s), 904 (m), 883 (m), 736 (s).

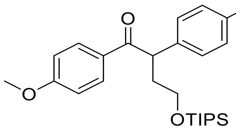

1-(4-methoxyphenyl)-2-(p-tolyl)-4-((triisopropylsilyl)oxy)butan-1-one (**18d**)

This compound was prepared following general procedure **A** from 1-(4-methoxyphenyl)-2-(*p*-tolyl)ethan-1-one (3.0 g, 12.5 mmol). Yield: 69% (3.8 g), isolated as colorless oil. Purification: Flash chromatography (PE/EtOAc, 99:1), $R_f = 0.43$ (PE/EtOAc 95:5).

^{1}H NMR (400 MHz, $CDCl_3$) δ 7.99 – 7.96 (m, 2H), 7.21 – 7.18 (m, 2H), 7.08 (d, J = 7.9 Hz, 2H), 6.87 – 6.83 (m, 2H), 4.88 (t, J = 7.1 Hz, 1H), 3.82 (s, 3H), 3.71 – 3.57 (m, 2H), 2.45 – 2.36 (m, 1H), 2.28 (s, 3H), 2.00 – 1.92 (m, 1H), 1.08 – 1.00 (m, 21H).

^{13}C NMR (101 MHz, $CDCl_3$): δ 198.9, 163.3, 137.0, 136.6, 131.2, 130.1, 129.6, 128.4, 113.7, 60.8, 55.5, 48.6, 37.2, 21.2, 18.2, 12.1.

HRMS (ESI/QTOF) m/z: $[M + Na]^+$ Calcd for $C_{27}H_{40}NaO_3Si^+$ 463.2639; Found 463.2656.

IR (ν_{max}, cm^{-1}) 2942 (m), 2866 (m), 1672 (s), 1600 (s), 1511 (s), 1258 (s), 1168 (s), 1106 (s), 883 (s), 808 (s).

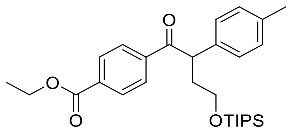

ethyl 4-(2-(p-tolyl)-4-((triisopropylsilyl)oxy)butanoyl)benzoate (**18e**)

This compound was prepared following general procedure **A** from ethyl 4-(2-(*p*-tolyl)acetyl)benzoate (3.2 g, 11.3 mmol). Yield: 42% (2.3 g), isolated as colorless oil. Purification: Flash chromatography (PE/EtOAc, 99.5:0.5), $R_f = 0.53$ (PE/EtOAc 95:5).

^{1}H NMR (400 MHz, $CDCl_3$) δ 8.05 – 7.98 (m, 4H), 7.18 – 7.15 (m, 2H), 7.10 – 7.07 (m, 2H), 4.92 (t, J = 7.1 Hz, 1H), 4.37 (q, J = 7.1 Hz, 2H), 3.70 (ddd, J = 10.1, 6.4, 4.8 Hz, 1H), 3.62 (ddd, J = 10.1, 7.3, 4.6 Hz, 1H), 2.42 (dtd, J = 14.2, 7.2, 4.8 Hz, 1H), 2.27 (s, 3H), 1.97 (dtd, J = 13.6, 6.6, 4.6 Hz, 1H), 1.38 (t, J = 7.1 Hz, 3H), 1.08 – 1.00 (m, 21H).

^{13}C NMR (101 MHz, $CDCl_3$): δ 200.0, 166.0, 140.4, 137.0, 136.0, 133.9, 129.8, 129.7, 128.7, 128.5, 61.5, 60.6, 49.5, 36.9, 21.2, 18.2, 14.4, 12.1.

HRMS (ESI/QTOF) m/z: $[M + Na]^+$ Calcd for $C_{29}H_{42}NaO_4Si^+$ 505.2750; Found 505.2745.

IR (ν_{max}, cm^{-1}) 2918 (s), 2864 (s), 1724 (s), 1273 (s), 1106 (s), 914 (m), 732 (s).

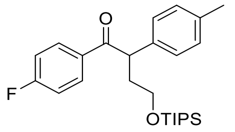

1-(4-fluorophenyl)-2-(p-tolyl)-4-((triisopropylsilyl)oxy)butan-1-one (**18f**)

This compound was prepared following general procedure **A** from 1-(4-fluorophenyl)-2-(*p*-tolyl)ethan-1-one (2.2 g, 10.3 mmol). Yield: 77% (3.4 g), isolated as yellow oil. Purification: Flash chromatography (PE/EtOAc, 99:1), $R_f = 0.41$ (PE/EtOAc 90:10).

^{1}H NMR (400 MHz, $CDCl_3$) δ 8.03 – 7.97 (m, 2H), 7.20 – 7.15 (m, 2H), 7.11-7.08 (m, 2H), 7.06-7.01 (m, 2H), 4.88 (t, J = 7.1 Hz, 1H), 3.68 (ddd, J = 10.1, 6.4, 4.8 Hz, 1H), 3.61 (ddd, J = 10.2, 7.2, 4.7 Hz, 1H), 2.40 (dtd, J = 14.2, 7.2, 4.8 Hz, 1H), 2.28 (s, 3H), 1.96 (dtd, J = 13.6, 6.7, 4.6 Hz, 1H), 1.06 – 1.00 (m, 21H).

^{13}C NMR (101 MHz, $CDCl_3$): δ 198.8, 165.6 (d, J = 254.3 Hz), 136.9, 136.4, 133.5 (br), 131.5 (d, J = 9.2 Hz), 129.8, 128.4, 115.6 (d, J = 21.7 Hz), 60.7, 49.0, 37.1, 21.2, 18.2, 12.1.

^{19}F NMR (377 MHz, $CDCl_3$) δ -104.4.

HRMS (ESI/QTOF) m/z: $[M + Na]^+$ Calcd for $C_{26}H_{37}FNaO_2Si^+$ 451.2439; Found 451.2447.

IR (ν_{max}, cm^{-1}) 2943 (w), 2866 (w), 1685 (m), 1438 (m), 1277 (m), 1130 (m), 1021 (m), 770 (m).

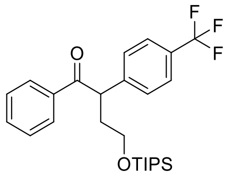

1-phenyl-2-(4-(trifluoromethyl)phenyl)-4-((triisopropylsilyl)oxy)butan-1-one (**18g**)

This compound was prepared following general procedure **A** from 1-phenyl-2-(4-(trifluoromethyl)phenyl)ethan-1-one (1.6 g, 6.0 mmol). Yield: 79% (2.2 g), isolated as colorless oil. Purification: Flash chromatography (PE/EtOAc, 99:1), $R_f = 0.88$ (PE/EtOAc 90:10).

^{1}H NMR (400 MHz, $CDCl_3$) δ 8.00 – 7.97 (m, 2H), 7.57 – 7.38 (m, 7H), 5.08 (t, J = 7.2 Hz, 1H), 3.71 (ddd, J = 10.7, 6.2, 4.7 Hz, 1H), 3.59 (ddd, J = 10.2, 7.5, 4.4 Hz, 1H), 2.50 – 2.42 (m, 1H), 2.04 – 1.96 (m, 1H), 1.12 – 1.00 (m, 21H).

^{13}C NMR (101 MHz, $CDCl_3$): δ 199.7, 143.6, 136.7, 133.3, 129.4 (q, J = 32.6 Hz), 129.1, 128.9, 128.8, 125.9 (q, J = 3.7 Hz), 124.2 (q, J = 272.0 Hz), 60.5, 49.0, 37.2, 18.1, 12.1.

^{19}F NMR (377 MHz, $CDCl_3$) δ -62.5.

HRMS (ESI/QTOF) m/z: $[M + Na]^+$ Calcd for $C_{26}H_{35}F_3NaO_2Si^+$ 487.2251; Found 487.2253.

IR (ν_{max}, cm^{-1}) 2943 (m), 2864 (m), 2344 (w), 1683 (m), 1464 (m), 1324 (s), 1166 (s), 1126 (s), 1069 (s).

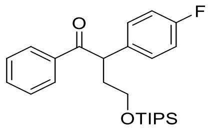

2-(4-fluorophenyl)-1-phenyl-4-((triisopropylsilyl)oxy)butan-1-one (**18h**)

This compound was prepared following general procedure **A** from 2-(4-fluorophenyl)-1-phenylethan-1-one (2.2 g, 10.3 mmol). Yield: 70% (3.0 g), isolated as colorless oil. Purification: Flash chromatography (PE/EtOAc, 99:1), R_f = 0.88 (PE/EtOAc 90:10).

^{1}H NMR (400 MHz, $CDCl_3$) δ 7.99 – 7.95 (m, 2H), 7.52 – 7.47 (m, 1H), 7.42 – 7.36 (m, 2H), 7.33 – 7.26 (m, 2H), 7.00 – 6.93 (m, 2H), 4.98 (t, J = 7.2 Hz, 1H), 3.69 (ddd, J = 10.7, 6.1, 4.8 Hz, 1H), 3.58 (ddd, J = 10.1, 7.6, 4.5 Hz, 1H), 2.40 (dtd, J = 14.2, 7.3, 4.8 Hz, 1H), 1.97 (dddd, J = 13.7, 7.5, 6.1, 4.5 Hz, 1H), 1.09 – 1.00 (m, 21H).

^{13}C NMR (101 MHz, $CDCl_3$): δ 200.3, 162.0 (d, J = 245.5 Hz), 136.9, 135.2 (d, J = 3.3 Hz), 133.1, 130.20 (d, J = 8.0 Hz), 128.9, 128.7, 115.81 (d, J = 21.3 Hz), 60.6, 48.4, 37.2, 18.2, 12.1.

^{19}F NMR (377 MHz, $CDCl_3$) δ -115.8.

HRMS (ESI/QTOF) m/z: $[M + Na]^+$ Calcd for $C_{25}H_{35}FNaO_2Si^+$ 437.2283; Found 437.2283.

IR (ν_{max}, cm^{-1}) 2943 (m), 2864 (m), 1683 (s), 1507 (s), 1227 (m), 1105 (s), 998 (m), 882 (s), 814 (m), 728 (s).

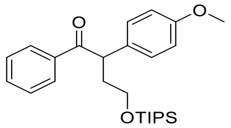

2-(4-methoxyphenyl)-1-phenyl-4-((triisopropylsilyl)oxy)butan-1-one (**18i**)

This compound was prepared following general procedure **A** from 2-(4-methoxyphenyl)-1-phenylethan-1-one (1.4 g, 6.1 mmol). Yield: 76% (2.0 g), isolated as yellow oil. Purification: Flash chromatography (PE/EtOAc, 98:2), R_f = 0.68 (PE/EtOAc 90:10).

^{1}H NMR (400 MHz, $CDCl_3$) δ 8.00 – 7.95 (m, 2H), 7.49 – 7.45 (m, 1H), 7.42 – 7.34 (m, 2H), 7.25 – 7.21 (m, 2H), 6.82 (d, J = 8.7 Hz, 2H), 4.92 (t, J = 7.2 Hz, 1H), 3.75 (s, 3H), 3.69 (ddd, J = 10.1, 6.1, 4.9 Hz, 1H), 3.61 (ddd, J = 10.1, 7.4, 4.7 Hz, 1H), 2.40 (dtd, J = 14.2, 7.2, 5.0 Hz, 1H), 1.97 (dddd, J = 13.6, 7.4, 6.1, 4.6 Hz, 1H), 1.10 – 0.99 (m, 21H).

^{13}C NMR (101 MHz, $CDCl_3$): δ 200.5, 158.7, 137.1, 132.8, 131.5, 129.7, 128.9, 128.6, 114.4, 60.7, 55.4, 48.5, 37.1, 18.2, 12.1.

HRMS (ESI/QTOF) m/z: $[M + Na]^+$ Calcd for $C_{26}H_{38}NaO_3Si^+$ 449.2482; Found 449.2486.

IR (ν_{max}, cm^{-1}) 2963 (m), 2850 (m), 1677 (m), 1491 (m), 1236 (m), 1108 (m), 865 (m), 760 (s).

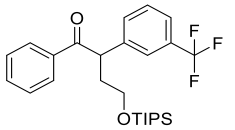

1-phenyl-2-(3-(trifluoromethyl)phenyl)-4-((triisopropylsilyl)oxy)butan-1-one (**18j**)

This compound was prepared following general procedure **A** from 1-phenyl-2-(3-(trifluoromethyl)phenyl)ethan-1-one (2.4 g, 9.1 mmol). Yield: 61% (2.6 g), isolated as colorless oil. Purification: Flash chromatography (PE/EtOAc, 99.4:0.6), R_f = 0.84 (PE/EtOAc 90:10).

^{1}H NMR (400 MHz, $CDCl_3$) δ 8.01 – 7.97 (m, 2H), 7.61 (brs, 1H), 7.56 – 7.46 (m, 3H), 7.44 – 7.39 (m, 3H), 5.09 (t, J = 7.2 Hz, 1H), 3.72 (ddd, J = 10.4, 5.9, 4.7 Hz, 1H), 3.56 (ddd, J = 10.2, 7.9, 4.2 Hz, 1H), 2.45 (dddd, J = 14.4, 8.0, 6.7, 4.7 Hz, 1H), 2.05 – 1.97 (m, 1H), 1.11 – 0.98 (m, 21H).

^{13}C NMR (101 MHz, $CDCl_3$): δ 199.8, 140.4, 136.7, 133.3, 132.1, 131.2 (q, J = 32.3 Hz), 129.4, 128.9, 128.8, 125.6 (q, J = 4.0 Hz), 124.2 (q, J = 272.0 Hz), 124.1 (q, J = 3.9 Hz), 60.4, 48.9, 37.2, 18.1, 12.0.

^{19}F NMR (377 MHz, $CDCl_3$) δ -62.6.

HRMS (ESI/QTOF) m/z: $[M + H]^+$ Calcd for $C_{26}H_{36}F_3O_2Si^+$ 465.2431; Found 465.2425.

IR (ν_{max}, cm^{-1}) 2943 (m), 2864 (m), 1686 (m), 1449 (m), 1327 (s), 1166 (s), 1126 (s), 1076 (s), 882 (m).

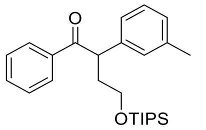

1-phenyl-2-(m-tolyl)-4-((triisopropylsilyl)oxy)butan-1-one (**18k**)

This compound was prepared following general procedure **A** from 1-phenyl-2-(m-tolyl)ethan-1-one (2.6 g, 12.3 mmol). Yield: 79% (4.0 g), isolated as yellow oil. Purification: Flash chromatography (PE/EtOAc, 99:1), R_f = 0.4 (PE/EtOAc 95:5).

^{1}H NMR (400 MHz, $CDCl_3$) δ 8.01 – 7.95 (m, 2H), 7.49 – 7.45 (m, 1H), 7.40 – 7.36 (m, 2H), 7.20 – 7.10 (m, 3H), 7.02 – 6.98 (m, 1H), 4.93 (t, J = 7.2 Hz, 1H), 3.69 (ddd, J = 10.1, 6.2, 4.9 Hz, 1H), 3.60 (ddd, J = 10.1, 7.4, 4.7 Hz, 1H), 2.41 (dtd, J = 13.7, 7.2, 4.9 Hz, 1H), 2.30 (s, 3H), 1.99 (dddd, J = 13.6, 7.3, 6.2, 4.7 Hz, 1H), 1.07 – 1.00 (m, 21H).

^{13}C NMR (101 MHz, $CDCl_3$): δ 200.3, 139.5, 138.6, 137.1, 132.9, 129.3, 128.9, 128.8, 128.6, 127.9, 125.8, 60.7, 49.3, 37.1, 21.6, 18.2, 12.1.

HRMS (ESI/QTOF) m/z: $[M + Na]^+$ Calcd for $C_{26}H_{38}NaO_2Si^+$ 433.2533; Found 433.2526.

IR (ν_{max}, cm^{-1}) 2947 (m), 2868 (m), 1679 (m), 1460 (m), 1273 (m), 1101 (s), 1065 (s), 882 (s), 778 (s).

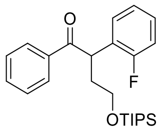

2-(2-fluorophenyl)-1-phenyl-4-((triisopropylsilyl)oxy)butan-1-one (**18l**)

This compound was prepared following general procedure **A** from 2-(2-fluorophenyl)-1-phenylethan-1-one (2.0 g, 9.3 mmol). Yield: 65% (2.5 g), isolated as colorless oil. Purification: Flash chromatography (PE/EtOAc, 99.4:0.6), R_f = 0.34 (PE/EtOAc 90:10).

^{1}H NMR (400 MHz, $CDCl_3$) δ 8.02 – 8.00 (m, 2H), 7.51 – 7.46 (m, 1H), 7.44 – 7.36 (m, 2H), 7.30 – 7.25 (m, 1H), 7.23 – 7.14 (m, 1H), 7.09 – 7.00 (m, 2H), 5.34 (t, J = 7.0 Hz, 1H), 3.73 – 3.64 (m, 2H), 2.53 – 2.42 (m, 1H), 2.05 – 1.97 (m, 1H), 1.10 – 0.98 (m, 21H)

^{13}**C NMR** (101 MHz, $CDCl_3$): δ 199.7, 160.3 (d, J = 245.6 Hz), 136.6, 133.1, 129.5 (d, J = 3.7 Hz), 128.8, 128.7, 128.66, 126.69 (d, J = 15.2 Hz), 124.6 (d, J = 3.5 Hz), 115.8 (d, J = 22.9 Hz), 60.9, 41.0, 36.2, 18.1, 12.1.

^{19}**F NMR** (377 MHz, $CDCl_3$) δ -116.0.

HRMS (ESI/QTOF) m/z: $[M + Na]^+$ Calcd for $C_{25}H_{35}FNaO_2Si^+$ 437.2283; Found 437.2283.

IR (ν_{max}, cm^{-1}) 2947 (m), 2864 (m), 1686 (m), 1489 (m), 1449 (m), 1230 (m), 1108 (m), 882 (m), 756 (s).

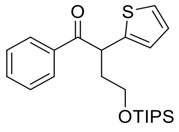

1-phenyl-2-(thiophen-2-yl)-4-((triisopropylsilyl)oxy)butan-1-one (**18m**)

This compound was prepared following general procedure **A** from 1-phenyl-2-(thiophen-2-yl)ethan-1-one (2.3 g, 11.3 mmol). Yield: 55% (2.5 g), isolated as colorless oil. Purification: Flash chromatography (PE/EtOAc, 99:1), R_f = 0.69 (PE/EtOAc 90:10).

^{1}**H NMR** (400 MHz, $CDCl_3$) δ 8.07 – 8.03 (m, 2H), 7.55 – 7.51 (m, 1H), 7.47 – 7.40 (m, 2H), 7.19 – 7.18 (m, 1H), 6.94 – 6.90 (m, 2H), 5.32 (t, J = 7.1 Hz, 1H), 3.76 – 3.66 (m, 2H), 2.43 (dddd, J = 14.0, 7.6, 6.6, 4.9 Hz, 1H), 2.07 (dddd, J = 13.6, 7.6, 5.9, 4.4 Hz, 1H), 1.09 – 1.00 (m, 21H).

^{13}**C NMR** (101 MHz, $CDCl_3$): δ 199.2, 141.8, 136.5, 133.2, 129.0, 128.7, 126.9, 126.0, 125.0, 60.5, 43.9, 38.1, 18.1, 12.1.

HRMS (ESI/QTOF) m/z: $[M + Na]^+$ Calculated for $C_{23}H_{34}NaO_2SSi^+$ 425.1941; Found 425.1951.

IR (ν_{max}, cm^{-1}) 2962 (w), 2941 (w), 2922 (w), 2893 (w), 2864 (m), 2841 (w), 1684 (m), 1597 (w), 1464 (w), 1446 (w), 1427 (w), 1385 (w), 1346 (w), 1327 (w), 1288 (w), 1255 (w), 1236 (m), 1205 (w), 1178 (w), 1157 (w), 1101 (m), 1065 (m), 1032 (w), 1012 (w), 982 (w), 943 (w), 920 (w), 881 (m), 852 (w), 812 (w).

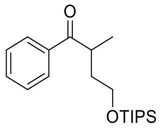

2-methyl-1-phenyl-4-((triisopropylsilyl)oxy)butan-1-one (**18o**)

This compound was prepared following general procedure **A** from propiophenone (1.5 g, 11.0 mmol). Yield: 52% (3.6 g), isolated as colorless oil. Purification: Flash chromatography (PE), R_f = 0.73 (PE/EtOAc 95:5).

^{1}**H NMR** (400 MHz, $CDCl_3$) δ 8.03 – 8.01 (m, 2H), 7.56 – 7.51 (m, 1H), 7.46 – 7.42 (m, 2H), 3.85 – 3.70 (m, 3H), 2.18 – 2.05 (m, 1H), 1.63 – 1.53 (m, 1H), 1.21 (d, J = 6.7 Hz, 3H), 1.14-1.00 (m, 21H).

^{13}**C NMR** (101 MHz, $CDCl_3$): δ 204.6, 136.7, 132.9, 128.64, 128.62, 61.0, 37.0, 36.9, 18.1, 16.9, 12.1.

HRMS (ESI/QTOF) m/z: $[M + Na]^+$ Calcd for $C_{20}H_{34}NaO_2Si^+$ 357.2220; Found 357.2223.

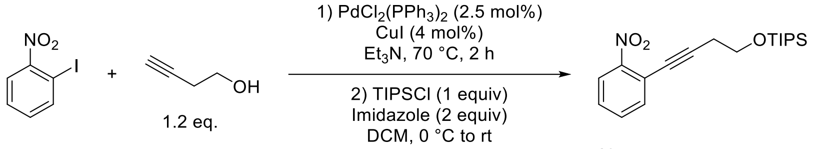

19, 74% yield over 2 steps

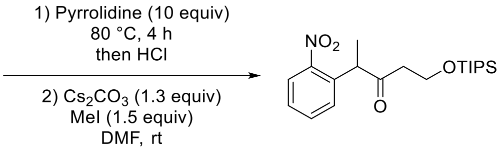

21, 64% yield over 2 steps

To a solution of 1-iodo-2-nitrobenzene (5.0 g, 20.0 mmol, 1.0 equiv) in Et_3N (40 mL, 0.5 M) were added but-3-yn-1-ol (1.83 mL, 24 mmol, 1.2 equiv), $PdCl_2(PPh_3)_2$ (350 mg, 0.5 mmol, 2.5 mol%) and CuI (150 mg, 0.8 mmol, 4 mol%). The reaction mixture was heated at 70 °C for 3 h. After cooling to room temperature, water was added, and the mixture was filtered through Celite. The filtrate was extracted with EtOAc (3 times). The organic layers were combined, washed with brine and dried over sodium sulfate. The residue was subjected to the next step without purification.

To a solution of 4-(2-nitrophenyl)but-3-yn-1-ol (3.0 g, 15.7 mmol, 1.0 equiv) in DCM (35 mL, 0.5 M) was added imidazole (2.14 g, 31.4 mmol, 2.0 equiv), followed by dropwise addition of TIPSCl (3.26 mL, 15.7 mmol, 1.0 equiv) at 0 °C. The reaction mixture was stirred at rt for 3 h, then the reaction was quenched with $NaHCO_3$. The reaction mixture was extracted with DCM (3 times). The organic layers were washed with brine and dried over sodium sulfate. The residue was subjected to silica gel column chromatography to afford the desired protected alcohol **19.**

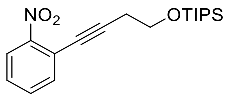

triisopropyl((4-(2-nitrophenyl)but-3-yn-1-yl)oxy)silane (**19**)

This compound was prepared from 4-(2-nitrophenyl)but-3-yn-1-ol (3.0 g, 15.7 mmol). Yield: 95% (5.2 g), isolated as colorless oil. Purification: Flash chromatography (PE/EtOAc, 90:10).

^{1}H NMR (400 MHz, $CDCl_3$) δ 7.97 (dd, J = 8.3, 1.3 Hz, 1H), 7.58 (dd, J = 7.8, 1.6 Hz, 1H), 7.52 (td, J = 7.5, 1.3 Hz, 1H), 7.42-7.38 (m, 1H), 3.93 (t, J = 7.2 Hz, 2H), 2.73 (t, J = 7.1 Hz, 2H), 1.11 – 1.05 (m, 21H).

^{13}C NMR (101 MHz, $CDCl_3$): δ 150.2, 135.0, 132.7, 128.2, 124.6, 119.2, 96.3, 77.0, 61.9, 24.5, 18.1, 12.1.

HRMS (ESI/QTOF) m/z: $[M + Na]^+$ Calcd for $C_{19}H_{29}NNaO_3Si^+$ 370.1809; Found 370.1806.

IR (ν_{max}, cm^{-1}) 2940 (m), 2865 (m), 1527 (s), 1463 (m), 1343 (m), 1107 (s), 881 (s), 744 (s).

A solution of triisopropyl((4-(2-nitrophenyl)but-3-yn-1-yl)oxy)silane **19** (5.2 g, 15 mmol, 1.0 equiv) in pyrrolidine (25 mL, 0.6 M) was heated at 80 °C for 3 h. Upon completion of the reaction, excess pyrrolidine was removed under reduced pressure. The residue was acidified with 1N HCl and stirred for 30 min, then extracted with EtOAc. The combined organic layers were washed with brine, dried, filtered and concentrated under reduced pressure. The residue was purified by silica gel column chromatography to afford the desired ketone **20**.

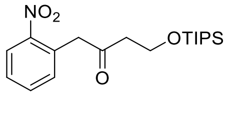

1-(2-nitrophenyl)-4-((triisopropylsilyl)oxy)butan-2-one (**20**)

This compound was prepared from triisopropyl((4-(2-nitrophenyl)but-3-yn-1-yl)oxy)silane (5.2 g, 15 mmol). Yield: 75% (4.2 g), isolated as colorless crystals. Purification: Flash chromatography (PE/EtOAc, 95:5), R_f = 0.32 (PE/EtOAc 90:10).

^{1}H NMR (400 MHz, $CDCl_3$) δ 8.11 (dd, J = 8.2, 1.4 Hz, 1H), 7.58 (td, J = 7.5, 1.4 Hz, 1H), 7.45 (ddd, J = 8.8, 7.5, 1.5 Hz, 1H), 7.27 – 7.24 (m, 1H), 4.19 (s, 2H), 4.04 (t, J = 6.3 Hz, 2H), 2.82 (t, J = 6.3 Hz, 2H), 1.16 – 1.03 (m, 21H).

^{13}C NMR (101 MHz, $CDCl_3$): δ 205.1, 148.9, 133.7, 133.67, 130.5, 128.5, 125.4, 59.3, 49.0, 46.2, 18.1, 12.0.

HRMS (ESI/QTOF) m/z: $[M + Na]^+$ Calcd for $C_{19}H_{31}NNaO_4Si^+$ 388.1915; Found 388.1919.

Glassware and stirring bar were stored in the oven and the reaction was carried out under inert atmosphere and dry conditions. To a solution of ketone **20** (0.75 g, 2.0 mmol, 1.0 equiv) in DMF (4.0 mL, 0.5 M) in a 50 mL pressure resistant round bottom flask were added Cs_2CO_3 (1.0 g, 3.0 mmol, 1.5 equiv) and MeI (8.0 g, 24.5 mmol, 1.2 equiv) sequentially. The reaction mixture was heated at 45 °C in an oil bath for 1 h. After completion of the reaction, a saturated aqueous NH_4Cl solution was added slowly at 0 °C to quench the reaction. The reaction mixture was extracted with EtOAc. The combined organic layers were washed with brine, dried, filtered and concentrated under reduced pressure. The residue was directly subjected to silica gel column chromatography to afford the desired ketone **21**.

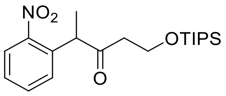

4-(2-nitrophenyl)-1-((triisopropylsilyl)oxy)pentan-3-one (**21**)

This compound was prepared from 1-(2-nitrophenyl)-4-((triisopropylsilyl)oxy)butan-2-one **20** (2.2 g, 10.3 mmol). Yield: 83% (650 mg), isolated as orange oil. Purification: Flash chromatography (PE/EtOAc, 97:3), R_f = 0.47 (PE/EtOAc 90:10).

^{1}H NMR (400 MHz, $CDCl_3$) δ 7.91 (dd, J = 8.1, 1.4 Hz, 1H), 7.58 (td, J = 7.6, 1.4 Hz, 1H), 7.42 (ddd, J = 8.6, 7.4, 1.4 Hz, 1H), 7.36 (dd, J = 7.8, 1.4 Hz, 1H), 4.38 (q, J = 7.0 Hz, 1H), 4.00 – 3.89 (m, 2H), 2.74 (ddd, J = 16.2, 6.9, 6.1 Hz, 1H), 2.62 (dt, J = 16.3, 5.9 Hz, 1H), 1.48 (d, J = 7.0 Hz, 3H), 1.09 – 0.98 (m, 21H).

^{13}C NMR (101 MHz, $CDCl_3$): δ 208.1, 149.5, 135.1, 133.4, 130.0, 128.1, 124.9, 59.1, 48.3, 44.7, 18.1, 17.1, 12.0.

HRMS (ESI/QTOF) m/z: $[M + Na]^+$ Calcd for $C_{20}H_{33}NNaO_4Si^+$ 402.2071; Found 402.2076.

IR (ν_{max}, cm^{-1}) 2946 (m), 2866 (m), 1720 (m), 1525 (s), 1460 (m), 1352 (m), 1099 (s), 883 (s), 746 (s).

3.2) General procedure B for the synthesis of enol tosylates **22**

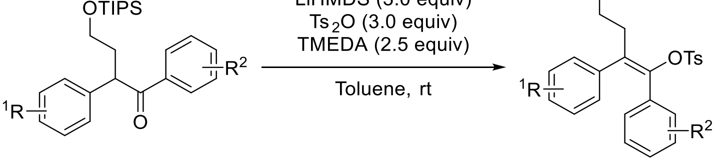

18, 21 **22**

Stereoisomerically pure enol tosylates were synthesized according to a reported procedure:[1] Glassware and stirring bar were stored in the oven and the reaction was carried out under inert atmosphere and dry conditions. To a stirred solution of LiHMDS (1.0 M in toluene, 7.6 mL, 7.6 mmol, 3.0 equiv) was added TMEDA (0.8 mL, 7.6 mmol, 3.0 equiv). After stirring for 15 min., a solution of ketone **18a** (1.0 g, 2.5 mmol, 1.0 equiv) in toluene (2.5 mL, 1.0 M) was added dropwise. After stirring for 1 h, the reaction mixture was placed in a 23 °C water bath and recrystallized Ts_2O (2.06 g, 6.0 mmol, 2.5 equiv) was added portion-wise. The reaction mixture was stirred vigorously for 3 h. After completion of the reaction, water was added slowly. The reaction mixture was extracted with DCM. The combined organic layers were dried, filtered and concentrated under reduced pressure. The residue was subjected to silica gel column chromatography to afford the desired enol tosylate **22a**.

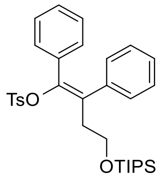

(*E*)-1,2-diphenyl-4-((triisopropylsilyl)oxy)but-1-en-1-yl 4-methylbenzenesulfonate (**22a**)

This compound was prepared following general procedure **B** from **18a** (1.0 g, 2.5 mmol). Yield: 64% (800 mg), isolated as white solid. Purification: Flash chromatography (PE/EtOAc, 98:2), R_f = 0.48 (PE/EtOAc 90:10).

^{1}H NMR (400 MHz, $CDCl_3$) δ 7.50 (d, J = 8.4 Hz, 2H), 7.15 – 7.09 (m, 5H), 7.04 – 6.98 (m, 3H), 6.93 – 6.88 (m, 4H), 3.61 (t, J = 6.9 Hz, 2H), 2.88 (t, J = 6.9 Hz, 2H), 2.36 (s, 3H), 1.01 – 0.97 (m, 21H).

^{13}C NMR (101 MHz, $CDCl_3$): δ 144.48, 144.45, 138.4, 134.5, 134.2, 132.5, 130.0, 129.6, 129.4, 128.20, 128.15, 127.8, 127.5, 127.2, 60.8, 36.6, 21.7, 18.1, 12.1.

HRMS (ESI/QTOF) m/z: $[M + Na]^+$ Calcd for $C_{32}H_{42}NaO_4SSi^+$ 573.2465; Found 573.2477.

IR (ν_{max}, cm^{-1}) 3356 (w), 2954 (m), 2868 (m), 1367 (m), 1187 (m), 1176 (s), 1044 (s), 878 (m), 782 (s).

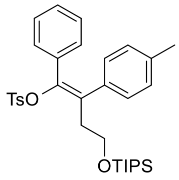

(*E*)-1-phenyl-2-(p-tolyl)-4-((triisopropylsilyl)oxy)but-1-en-1-yl 4-methylbenzenesulfonate (**22b**)

This compound was prepared following general procedure **B** from **18b** (2.0 g, 4.9 mmol). Yield: 22% (600 mg), isolated as brown solid. Purification: Flash chromatography (PE/EtOAc, 98:2), R_f = 0.55 (PE/EtOAc 90:10).

^{1}H NMR (400 MHz, $CDCl_3$) δ 7.50 (d, J = 8.4 Hz, 2H), 7.10 (d, J = 8.2 Hz, 2H), 7.03 – 6.97 (m, 1H), 6.97 – 6.88 (m, 8H), 3.60 (t, J = 6.9 Hz, 2H), 2.86 (t, J = 6.9 Hz, 2H), 2.36 (s, 3H), 2.25 (s, 3H), 1.02 – 0.95 (m, 21H).

^{13}C NMR (101 MHz, $CDCl_3$): δ 144.4, 144.2, 136.8, 135.3, 134.6, 134.4, 132.3, 130.0, 129.4, 129.36, 128.9, 128.1, 127.7, 127.5, 60.8, 36.7, 21.7, 21.3, 18.1, 12.1.

HRMS (ESI/QTOF) m/z: $[M + Na]^+$ Calcd for $C_{33}H_{44}NaO_4SSi^+$ 587.2622; Found 587.2631.

IR (ν_{max}, cm^{-1}) 2942 (m), 2866 (m), 1464 (m), 1374 (m), 1189 (s), 1179 (s), 1106 (s), 1049 (m), 883 (s), 818 (s), 760 (s).

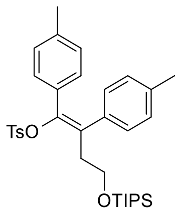

(E)-1,2-di-p-tolyl-4-((triisopropylsilyl)oxy)but-1-en-1-yl 4-methylbenzenesulfonate (**22c**)

This compound was prepared following general procedure **B** from **18c** (2.5 g, 5.9 mmol). Yield: 37% (1.3 g), isolated as colorless oil. Purification: Flash chromatography (PE/EtOAc, 99:1), R_f = 0.32 (PE/EtOAc 95:5).

^{1}H NMR (400 MHz, $CDCl_3$) δ 7.51 (d, J = 8.4 Hz, 2H), 7.12 – 7.10 (m, 2H), 6.97 – 6.90 (m, 4H), 6.80 (d, J = 8.2 Hz, 2H), 6.73 (d, J = 7.9 Hz, 2H), 3.57 (t, J = 7.0 Hz, 2H), 2.81 (t, J = 7.0 Hz, 2H), 2.37 (s, 3H), 2.25 (s, 3H), 2.18 (s, 3H), 1.00 – 0.96 (m, 21H).

^{13}C NMR (101 MHz, $CDCl_3$): δ 144.4, 144.3, 137.6, 136.7, 135.5, 134.7, 131.4, 129.9, 129.4, 129.3, 128.9, 128.22, 128.17, 60.9, 36.7, 21.7, 21.4, 21.3, 18.1, 12.1.

HRMS (ESI/QTOF) m/z: $[M + Na]^+$ Calcd for $C_{34}H_{46}NaO_4SSi^+$ 601.2778; Found 601.2783.

IR (ν_{max}, cm^{-1}) 3381 (w), 2936 (w), 2864 (m), 1507 (m), 1367 (m), 1176 (s), 1044 (m), 969 (m), 825 (s).

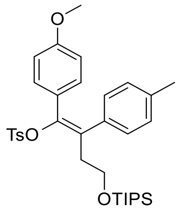

(E)-1-(4-methoxyphenyl)-2-(p-tolyl)-4-((triisopropylsilyl)oxy)but-1-en-1-yl 4-methylbenzenesulfonate (**22d**)

This compound was prepared following general procedure **B** from **18d** (2.5 g, 5.7 mmol). Yield: 47% (1.6 g), isolated as yellow solid. Purification: Flash chromatography (PE/EtOAc, 99:1), R_f = 0.3 (PE/EtOAc 90:10).

^{1}H NMR (400 MHz, $CDCl_3$) δ 7.52 (d, J = 8.3 Hz, 2H), 7.13 – 7.11 (m, 2H), 6.96 – 6.89 (m, 4H), 6.83 (d, J = 8.8 Hz, 2H), 6.45 (d, J = 8.9 Hz, 2H), 3.68 (s, 3H), 3.58 (t, J = 7.0 Hz, 2H), 2.83 (t, J = 7.0 Hz, 2H), 2.37 (s, 3H), 2.25 (s, 3H), 1.00 – 0.96 (m, 21H).

^{13}C NMR (101 MHz, $CDCl_3$): δ 159.0, 144.3, 144.2, 136.7, 135.6, 134.8, 131.3, 130.9, 129.4, 129.3, 128.9, 128.2, 126.8, 113.0, 60.9, 55.2, 36.6, 21.7, 21.3, 18.1, 12.1.

HRMS (ESI/QTOF) m/z: $[M + Na]^+$ Calcd for $C_{34}H_{46}NaO_5SSi^+$ 617.2727; Found 617.2726.

IR (ν_{max}, cm^{-1}) 3385 (w), 2938 (w), 2863 (w), 1669 (w), 1601 (m), 1510 (w), 1258 (s), 1172 (s), 1125 (s), 1035 (s), 1009 (s), 814 (s).

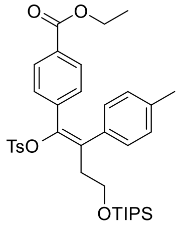

ethyl (E)-4-(2-(p-tolyl)-1-(tosyloxy)-4-((triisopropylsilyl)oxy)but-1-en-1-yl)benzoate (**22e**)

This compound was prepared following general procedure **B** from **18e** (2.0 g, 4.1 mmol). Yield: 43% (1.1 g), isolated as orange solid. Purification: Flash chromatography (PE/EtOAc, 99:1), R_f = 0.24 (PE/EtOAc 95:5).

^{1}H NMR (400 MHz, $CDCl_3$) δ 7.59 (d, J = 8.4 Hz, 2H), 7.52 (d, J = 8.4 Hz, 2H), 7.11 (d, J = 8.2 Hz, 2H), 6.97 – 6.88 (m, 6H), 4.31 (q, J = 7.1 Hz, 2H), 3.58 (t, J = 6.8 Hz, 2H), 2.85 (t, J = 6.8 Hz, 2H), 2.36 (s, 3H), 2.26 (s, 3H), 1.35 (t, J = 7.1 Hz, 3H), 1.01 – 0.97 (m, 21H).

^{13}C NMR (101 MHz, $CDCl_3$): δ 166.3, 144.9, 143.0, 139.0, 137.4, 134.8, 134.4, 134.3, 129.8, 129.5, 129.3, 129.3, 129.1, 128.7, 128.2, 61.1, 60.7, 36.8, 21.7, 21.3, 18.1, 14.5, 12.1.

HRMS (ESI/QTOF) m/z: $[M + Na]^+$ Calcd for $C_{36}H_{48}NaO_6SSi^+$ 659.2833; Found 659.2847.

IR (ν_{max}, cm^{-1}) 2968 (m), 2868 (m), 1722 (m), 1683 (m), 1468 (m), 1406 (m), 1273 (s), 1101 (s), 1019 (s), 878 (m), 731 (s).

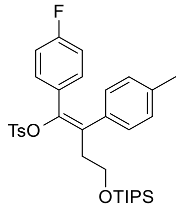

(*E*)-1-(4-fluorophenyl)-2-(p-tolyl)-4-((triisopropylsilyl)oxy)but-1-en-1-yl 4-methylbenzenesulfonate (**22f**)

This compound was prepared following general procedure **B** from **18f** (3.0 g, 7.0 mmol). Yield: 49% (2 g), isolated as colorless oil. Purification: Flash chromatography (PE/EtOAc, 98:2), R_f = 0.4 (PE/EtOAc 95:5).

^{1}H NMR (400 MHz, $CDCl_3$) δ 7.52 (d, J = 8.4 Hz, 2H), 7.15 – 7.13 (m, 2H), 6.97 – 6.87 (m, 6H), 6.61 (t, J = 8.8 Hz, 2H), 3.59 (t, J = 6.8 Hz, 2H), 2.84 (t, J = 6.8 Hz, 2H), 2.38 (s, 3H), 2.26 (s, 3H), 1.05 – 0.96 (m, 21H).

^{13}C NMR (101 MHz, $CDCl_3$): δ 162.0 (d, J = 248.3 Hz), 144.7, 143.2, 137.0, 135.1, 134.6, 132.6, 131.8 (d, J = 8.2 Hz), 130.5 (d, J = 3.4 Hz), 129.4, 129.3, 129.0, 128.1, 114.6 (d, J = 21.7 Hz), 60.7, 36.6, 21.7, 21.3, 18.1, 12.1.

^{19}F NMR (377 MHz, $CDCl_3$) δ -113.2.

HRMS (ESI/QTOF) m/z: $[M + Na]^+$ Calcd for $C_{33}H_{43}FNaO_4SSi^+$ 605.2528; Found 605.2537.

IR (ν_{max}, cm^{-1}) 2942 (m), 2866 (m), 2358 (m), 1507 (m), 1370 (m), 1229 (m), 1189 (s), 1179 (s), 1106 (s), 840 (s), 814 (s), 757 (s).

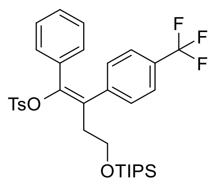

(*E*)-1-phenyl-2-(4-(trifluoromethyl)phenyl)-4-((triisopropylsilyl)oxy)but-1-en-1-yl 4-methylbenzenesulfonate (**22g**)

This compound was prepared following general procedure **B** from **18g** (2.0 g, 4.3 mmol). Yield: 31% (830 mg), isolated as yellow oil. Purification: Flash chromatography (PE/EtOAc, 98:2).

^{1}H NMR (400 MHz, $CDCl_3$) δ 7.47 (d, J = 8.2 Hz, 2H), 7.40 (d, J = 8.1 Hz, 2H), 7.17 (d, J = 8.0 Hz, 2H), 7.09 (d, J = 8.1 Hz, 2H), 7.07 – 7.00 (m, 1H), 6.93 (t, J = 7.6 Hz, 2H), 6.88 (d, J = 7.3 Hz, 2H), 3.65 (t, J = 6.5 Hz, 2H), 2.94 (t, J = 6.5 Hz, 2H), 2.36 (s, 3H), 1.03 – 0.89 (m, 21H).

^{13}C NMR (101 MHz, $CDCl_3$): δ 145.4, 144.6, 142.6, 134.4, 133.5, 131.6, 130.0, 129.4, 129.2 (q, J = 32.4 Hz), 128.2, 128.1, 127.7, 125.1 (q, J = 3.7 Hz), 124.1 (q, J = 272.0 Hz), 60.8, 36.4, 21.7, 18.1, 12.0.

^{19}F NMR (377 MHz, $CDCl_3$) δ -62.5.

HRMS (ESI/QTOF) m/z: $[M + Na]^+$ Calcd for $C_{33}H_{41}F_3NaO_4SSi^+$ 641.2340; Found 641.2357.

IR (ν_{max}, cm^{-1}) 2945 (m), 2830 (m), 1383 (m), 1167 (s), 1103 (m), 883 (m), 754 (s).

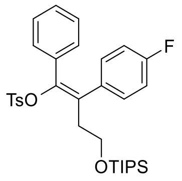

(E)-2-(4-fluorophenyl)-1-phenyl-4-((triisopropylsilyl)oxy)but-1-en-1-yl 4-methylbenzenesulfonate (**22h**)

This compound was prepared following general procedure **B** from **18h** (2.8 g, 6.8 mmol). Yield: 55% (2.1 g), isolated as white solid. Purification: Flash chromatography (PE/EtOAc, 98:2), R_f = 0.29 (PE/EtOAc 95:5).

^{1}H NMR (400 MHz, $CDCl_3$) δ 7.48 (d, J = 8.4 Hz, 2H), 7.09 (d, J = 8.1 Hz, 2H), 7.05 – 6.97 (m, 3H), 6.96 – 6.80 (m, 6H), 3.62 (t, J = 6.7 Hz, 2H), 2.88 (t, J = 6.7 Hz, 2H), 2.36 (s, 3H), 1.04 – 0.94 (m, 21H).

^{13}C NMR (101 MHz, $CDCl_3$): δ 144.8, 144.5, 134.5, 134.0, 131.6, 131.3 (d, J = 8.2 Hz), 130.0, 129.4, 128.1, 127.9, 127.6, 115.2 (d, J = 21.4 Hz), 60.7, 36.6, 21.7, 18.1, 12.1. *Two aromatic quaternary carbons were not observed due to weak signal intensities.*

^{19}F NMR (377 MHz, $CDCl_3$) δ -114.3.

HRMS (ESI/QTOF) m/z: $[M + Na]^+$ Calcd for $C_{32}H_{41}FNaO_4SSi^+$ 591.2371; Found 591.2387.

IR (ν_{max}, cm^{-1}) 3553 (w), 3374 (w), 2950 (m), 2864 (m), 1507 (m), 1367 (m), 1227 (m), 1191 (s), 1176 (s), 1051 (m), 836 (m), 754 (s).

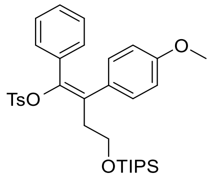

(E)-2-(4-methoxyphenyl)-1-phenyl-4-((triisopropylsilyl)oxy)but-1-en-1-yl 4-methylbenzenesulfonate (**22i**)

This compound was prepared following general procedure **B** from **18i** (1.9 g, 4.5 mmol). Yield: 48% (1.25 g), isolated as colorless oil. Purification: Flash chromatography (PE/EtOAc, 97:3), R_f = 0.39 (PE/EtOAc 90:10).

^{1}H NMR (400 MHz, $CDCl_3$) δ 7.50 (d, J = 8.4 Hz, 2H), 7.09 (d, J = 8.1 Hz, 2H), 7.03 – 6.97 (m, 1H), 6.96 – 6.88 (m, 6H), 6.68 (d, J = 8.7 Hz, 2H), 3.73 (s, 3H), 3.60 (t, J = 6.9 Hz, 2H), 2.86 (t, J = 6.9 Hz, 2H), 2.36 (s, 3H), 1.01 – 0.96 (m, 21H).

^{13}C NMR (101 MHz, $CDCl_3$): δ 158.7, 144.4, 144.1, 134.6, 134.4, 131.9, 130.7, 130.5, 130.0, 129.4, 128.1, 127.6, 127.5, 113.7, 60.9, 55.3, 36.6, 21.7, 18.1, 12.1.

HRMS (ESI/QTOF) m/z: $[M + Na]^+$ Calcd for $C_{33}H_{44}NaO_5SSi^+$ 603.2571; Found 603.2580.

IR (ν_{max}, cm^{-1}) 2954 (m), 2864 (m), 1510 (m), 1374 (m), 1248 (m), 1176 (s), 1094 (m), 1044 (s), 753 (m).

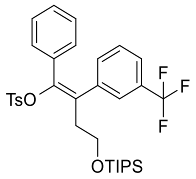

(E)-1-phenyl-2-(3-(trifluoromethyl)phenyl)-4-((triisopropylsilyl)oxy)but-1-en-1-yl 4-methylbenzenesulfonate (**22j**)

This compound was prepared following general procedure **B** from **18j** (2.6 g, 5.6 mmol). Yield: 46% (1.6 g), isolated as white solid. Purification: Flash chromatography (PE/EtOAc, 98:2), R_f = 0.45 (PE/EtOAc 90:10).

^{1}H NMR (400 MHz, $CDCl_3$) δ 7.47 (d, J = 8.4 Hz, 2H), 7.40 – 7.34 (m, 1H), 7.32 – 7.19 (m, 3H), 7.12 – 7.07 (m, 2H), 7.06 – 7.00 (m, 1H), 6.95 – 6.88 (m, 2H), 6.88 – 6.84 (m, 2H), 3.65 (t, J = 6.5 Hz, 2H), 2.94 (t, J = 6.5 Hz, 2H), 2.36 (s, 3H), 1.01 – 0.92 (m, 21H).

^{13}C NMR (101 MHz, $CDCl_3$): δ 145.6, 144.6, 139.5, 134.4, 133.5, 133.1, 131.5, 130.6 (q, J = 32.3 Hz), 130.0, 129.4, 128.6, 128.2, 128.1, 127.7, 126.5 (q, J = 3.8 Hz), 124.0 (q, J = 272.1 Hz), 123.9 (q, J = 3.8 Hz), 60.8, 36.2, 21.7, 18.0, 12.0.

^{19}F NMR (377 MHz, $CDCl_3$) δ -62.9.

HRMS (ESI/QTOF) m/z: $[M + Na]^+$ Calcd for $C_{33}H_{41}F_3NaO_4SSi^+$ 641.2339; Found 641.2351.

IR (ν_{max}, cm^{-1}) 2939 (w), 2864 (w), 1460 (m), 1374 (m), 1338 (s), 1176 (s), 1126 (s), 997 (m), 807 (s), 756 (m).

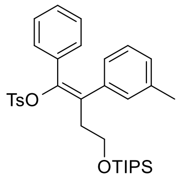

(E)-1-phenyl-2-(m-tolyl)-4-((triisopropylsilyl)oxy)but-1-en-1-yl 4-methylbenzenesulfonate (**22k**)

This compound was prepared following general procedure **B** from **18k** (3.5 g, 8.5 mmol). Yield: 35% (1.7 g), isolated as colorless oil. Purification: Flash chromatography (PE/EtOAc, 98:2), R_f = 0.25 (PE/EtOAc 95:5).

^{1}H NMR (400 MHz, $CDCl_3$) δ 7.50 (d, J = 8.4 Hz, 2H), 7.12 – 7.07 (m, 2H), 7.03 – 6.96 (m, 2H), 6.96 – 6.86 (m, 6H), 6.77 (dt, J = 7.7, 1.8 Hz, 1H), 3.60 (t, J = 6.9 Hz, 2H), 2.87 (t, J = 6.9 Hz, 2H), 2.36 (s, 3H), 2.19 (s, 3H), 1.01 – 0.97 (m, 21H).

^{13}C NMR (101 MHz, $CDCl_3$): δ 144.4, 144.3, 138.3, 137.7, 134.6, 134.2, 132.5, 130.1, 129.9, 129.4, 128.1, 128.0, 127.9, 127.7, 127.4, 126.7, 60.8, 36.6, 21.7, 21.4, 18.1, 12.1.

HRMS (ESI/QTOF) m/z: $[M + Na]^+$ Calcd for $C_{33}H_{44}NaO_4SSi^+$ 587.2622; Found 587.2640.

IR (ν_{max}, cm^{-1}) 2961 (m), 2864 (m), 1468 (m), 1367 (m), 1176 (s), 1047 (s), 828 (s), 753 (s).

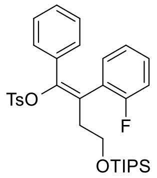

(E)-2-(2-fluorophenyl)-1-phenyl-4-((triisopropylsilyl)oxy)but-1-en-1-yl 4-methylbenzenesulfonate (**22l**)

This compound was prepared following general procedure **B** from **18l** (1.8 g, 4.4 mmol). Yield: 48% (1.2 g), isolated as colorless oil. Purification: Flash chromatography (PE/EtOAc, 98:2), $R_f = 0.25$ (PE/EtOAc 95:5).

^{1}H NMR (400 MHz, $CDCl_3$) δ 7.56 (d, $J = 8.4$ Hz, 2H), 7.14 – 7.12 (m, 3H), 7.06 – 7.01 (m, 1H), 7.00 – 6.86 (m, 7H), 3.63 (t, $J = 6.8$ Hz, 2H), 2.80 (t, $J = 6.9$ Hz, 2H), 2.37 (s, 3H), 1.04 – 0.98 (m, 21H).

^{13}C NMR (101 MHz, $CDCl_3$): δ 160.1 (d, $J = 246.7$ Hz), 145.8, 144.6, 134.0 (d, $J = 2.5$ Hz), 131.7 (d, $J = 3.6$ Hz), 129.4, 129.3, 129.28, 129.26, 128.2, 128.1, 127.5, 127.0, 126.0 (d, $J = 15.7$ Hz), 123.9 (d, $J = 3.5$ Hz), 115.5 (d, $J = 21.7$ Hz), 60.7, 35.6, 21.7, 18.0, 12.0.

^{19}F NMR (377 MHz, $CDCl_3$) δ -117.2.

HRMS (ESI/QTOF) m/z: $[M + Na]^+$ Calcd for $C_{32}H_{41}FNaO_4SSi^+$ 591.2371; Found 591.2390.

IR (ν_{max}, cm^{-1}) 3384 (w), 2939 (m), 2864 (m), 1446 (m), 1371 (m), 1176 (s), 1054 (s), 818 (s), 757 (s).

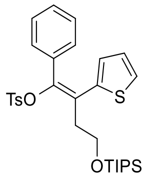

(*E*)-1-phenyl-2-(thiophen-2-yl)-4-((triisopropylsilyl)oxy)but-1-en-1-yl 4-methylbenzenesulfonate (**22m**)

This compound was prepared following general procedure **B** from **18m** (2.3 g, 5.8 mmol). Yield: 65% (2.1 g), isolated as green oil, mixture of *E*- and *Z*-isomers (7 to 1 ratio). Purification: Flash chromatography (PE/EtOAc, 98:2), $R_f = 0.57$ (PE/EtOAc 90:10).

^{1}H NMR (400 MHz, $CDCl_3$) 2 isomers in a ratio around 7:1: δ 7.47-7.45 (m, 2/7H), 7.35-6.99 (m, 12H + 8/7H), 6.79 (dd, $J = 5.1$, 3.6 Hz, 1/7H), 6.71 (dd, $J = 3.6$, 1.2 Hz, 1/7H), 3.77 (t, $J = 7.2$ Hz, 2/7H), 3.66 (t, $J = 7.2$ Hz, 2H), 2.95 (t, $J = 7.2$ Hz, 2/7H), 2.70 (t, $J = 7.2$ Hz, 2H), 2.36 (s, 3/7H), 2.33 (s, 3H), 1.06-0.93 (m, 21H + 21/7H).

^{13}C NMR (101 MHz, $CDCl_3$) (for only major isomer): δ 144.2, 143.4, 138.3, 134.4, 133.3, 130.4, 129.1, 128.8, 128.0, 128.0, 126.7, 126.4, 122.8, 62.1, 36.4, 21.7, 18.0, 12.0.

HRMS (ESI/QTOF) m/z: $[M + Na]^+$ Calculated for $C_{30}H_{40}NaO_4S_2Si^+$ 579.2029; Found 579.2037.

IR (ν_{max}, cm^{-1}) 2962 (w), 2925 (w), 2891 (w), 2864 (w), 1464 (w), 1444 (w), 1373 (m), 1257 (w), 1190 (m), 1174 (s), 1097 (m), 1065 (m), 1036 (m), 1018 (m), 989 (m), 943 (w), 918 (w), 881 (m), 856 (w), 839 (m), 808 (s).

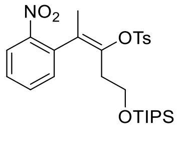

(*E*)-2-(2-nitrophenyl)-5-((triisopropylsilyl)oxy)pent-2-en-3-yl 4-methylbenzenesulfonate (**22n**)

This compound was prepared following general procedure **B** from **21** (0.67 g, 1.8 mmol) as starting material. Yield: 43% (440 mg), isolated as yellow oil. Purification: Flash chromatography (PE/EtOAc, 97:3), $R_f = 0.34$ (PE/EtOAc 90:10).

^{1}H NMR (400 MHz, $CDCl_3$) δ 7.99 (dd, $J = 8.2$, 1.3 Hz, 1H), 7.92 (d, $J = 8.3$ Hz, 2H), 7.57 (td, $J = 7.5$, 1.3 Hz, 1H), 7.45 (ddd, $J = 8.2$, 7.4, 1.5 Hz, 1H), 7.40 – 7.38 (m, 2H), 7.33 (dd, $J = 7.7$, 1.5 Hz, 1H), 3.75 (dt, $J = 9.7$, 7.5 Hz, 1H), 3.57 (ddd, $J = 9.7$, 7.3, 5.3 Hz, 1H), 2.46 (s, 3H), 2.32 – 2.26 (m, 2H), 1.73 (s, 3H), 1.00 – 0.93 (m, 21H).

^{13}C NMR (101 MHz, $CDCl_3$): δ 148.0, 145.3, 142.7, 135.3, 134.3, 133.5, 131.8, 130.1, 128.8, 128.3, 127.8, 124.7, 60.0, 35.3, 21.9, 18.7, 18.0, 12.0.

HRMS (ESI/QTOF) m/z: [M + Na]$^+$ Calcd for $C_{27}H_{39}NNaO_6SSi^+$ 556.2160; Found 556.2155.

IR (ν_{max}, cm^{-1}) 2965 (s), 2889 (s), 1528 (s), 1367 (s), 1176 (s), 1058 (s), 878 (s), 754 (s).

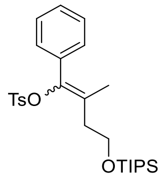

2-methyl-1-phenyl-4-((triisopropylsilyl)oxy)but-1-en-1-yl 4-methylbenzenesulfonate (**22o**)

This compound was prepared following general procedure **B** from **18o** (2.0 g, 6.0 mmol). Yield: 30% (850 mg), isolated as colorless oil. Purification: Flash chromatography (PE/EtOAc, 99.5:0.5), R_f = 0.55 (PE/EtOAc 90:10).

^{1}H NMR (400 MHz, $CDCl_3$) δ (mixture of 2 isomers: 2:1) 7.44 (d, J = 8.3 Hz, 1H), 7.41 (d, J = 8.3 Hz, 2H), 7.21– 7.11 (m, 5 + 2.5 H), 7.06 – 7.02 (m, 2 + 1H), 3.88 (t, J = 6.7 Hz, 2H), 3.75 (t, J = 6.7 Hz, 1H), 2.57 (t, J = 6.7 Hz, 2H), 2.33 (s, 3 + 1.5H), 2.32 (t, J = 6.7 Hz, 1H), 1.90 (s, 1.5H), 1.80 (s, 3H), 1.10 – 0.99 (m, 21 + 10.5H).

^{13}C NMR (101 MHz, $CDCl_3$): δ (major isomer) 144.2, 142.2, 134.7, 133.9, 129.8, 129.3, 128.1, 128.04, 128.0, 127.8, 62.0, 36.0, 21.7, 18.9, 18.2, 12.1.

HRMS (ESI/QTOF) m/z: [M + Na]$^+$ Calcd for $C_{27}H_{40}NaO_4SSi^+$ 511.2309; Found 511.2310.

IR (ν_{max}, cm^{-1}) 2942 (m), 2866 (m), 1464 (w), 1374 (m), 1176 (s), 1089 (s), 984 (m), 811 (m).

3.3) General procedure C for the synthesis of alcohols **7**

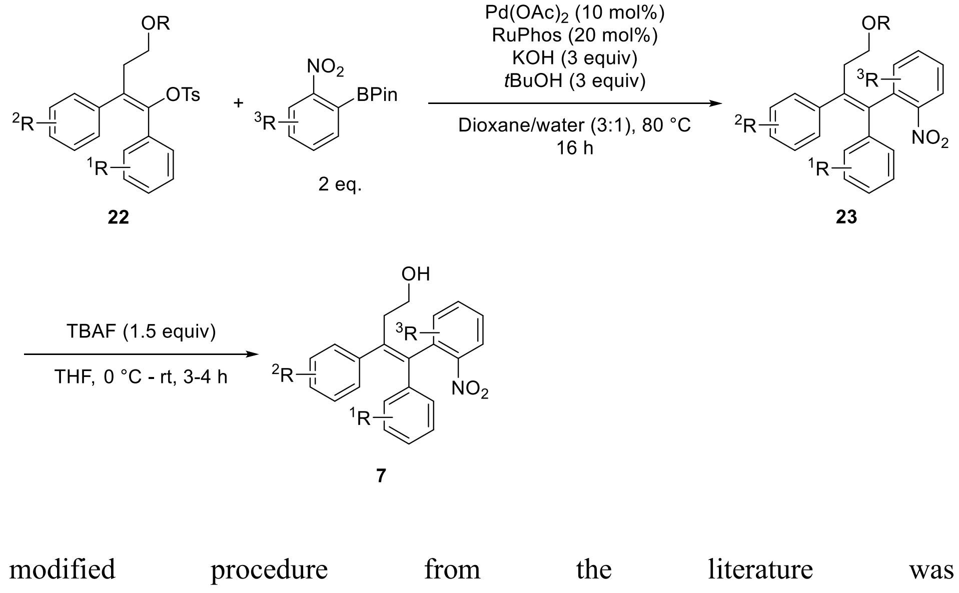

A modified procedure from the literature was used:[1] Glassware and stirring bar were stored in an oven and the reaction was carried out under an inert atmosphere and anhydrous conditions. Dioxane and water were degassed using Freeze-pump-thaw technique. To a solution of enol tosylate **22a** (1.6 g, 3.0 mmol, 1.0 equiv) in dioxane/water (3:1, 0.06 M) were added 2-nitrophenylboronic acid pinacol ester (1.5 g, 6.0 mmol, 2.0 equiv), Pd(OAc)$_2$ (67 mg, 0.3 mmol, 10 mol%), RuPhos (280 mg, 0.6 mmol, 20 mol%), KOH (510 mg, 9.0 mmol, 3.0 equiv) and *t*-BuOH (0.86 mL, 9.0 mmol, 3.0 equiv). The reaction mixture was heated at 80 °C overnight. After cooling to room temperature, water was added, and the mixture was extracted with EtOAc (3 times). The organic layers were combined, washed with brine and dried over sodium sulfate. The residue was subjected to

silica gel column chromatography to remove Pd-nanoparticles. The resulting material was used directly in the next step. To a solution of alkene **23a** (270 mg, 0.5 mmol, 1.0 equiv) in THF (5 mL, 0.1 M) at 0 °C was added dropwise TBAF (1 M in THF, 0.8 mL, 0.8 mmol, 1.5 equiv). After being stirred for 3 hours at room temperature, the reaction mixture was quenched with saturated aqueous $NaHCO_3$ and extracted with EtOAc. The organic layers were combined, washed with brine and dried over sodium sulfate, filtered and evaporated *in vacuo*. The residue was subjected to silica gel column chromatography (PE:EtOAc) to give the alcohol **7a**.

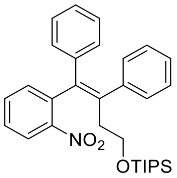

(*E*)-triisopropyl((4-(2-nitrophenyl)-3,4-diphenylbut-3-en-1-yl)oxy)silane (**23a**)

This compound was prepared following general procedure **C** using substrate **22a** (1.7 g, 3.0 mmol) as starting material. Yield: 60% (910 mg), isolated as orange solid. Purification: Flash chromatography (PE/EtOAc, 98:2), Rf = 0.69 (PE/EtOAc 90:10).

^{1}H NMR (400 MHz, $CDCl_3$) δ 7.98 (dd, J = 8.2, 1.3 Hz, 1H), 7.70 (dd, J = 7.6, 1.6 Hz, 1H), 7.64 (td, J = 7.5, 1.3 Hz, 1H), 7.48 – 7.44 (m, 1H), 7.20 – 7.10 (m, 5H), 7.03 – 6.95 (m, 3H), 6.94 – 6.85 (m, 2H), 3.60 – 3.50 (m, 2H), 2.72 – 2.56 (m, 2H), 1.03 – 0.91 (m, 21H).

^{13}C NMR (101 MHz, $CDCl_3$): δ 148.9, 140.9, 140.1, 138.6, 138.0, 136.3, 133.3, 132.9, 130.7, 129.7, 128.2, 128.0, 127.5, 126.8, 126.5, 124.9, 61.2, 40.0, 18.12, 18.10, 12.0.

HRMS (ESI/QTOF) m/z: $[M + Na]^+$ Calcd for $C_{31}H_{39}NNaO_3Si^+$ 524.2591; Found 524.2598.

IR (ν_{max}, cm^{-1}) 3415 (w), 2900 (m), 1625 (m), 1530 (s), 1506 (s), 1250 (m), 1180 (m), 1037 (s), 835 (m), 754 (s).

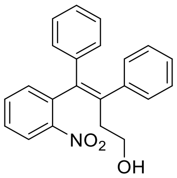

(*E*)-4-(2-nitrophenyl)-3,4-diphenylbut-3-en-1-ol (**7a**)

This compound was prepared following general procedure **C** using substrate **23a** (270 mg, 0.5 mmol) as starting material. Yield: 93% (174 mg), isolated as yellow crystals. Purification: Flash chromatography (PE/EtOAc, 82:18), R_f = 0.13 (PE/EtOAc 80:20).

^{1}H NMR (400 MHz, $CDCl_3$) δ 8.02 (dd, J = 8.2, 1.3 Hz, 1H), 7.66 (td, J = 7.5, 1.3 Hz, 1H), 7.56 (dd, J = 7.7, 1.4 Hz, 1H), 7.48 (td, J = 7.8, 1.4 Hz, 1H), 7.23 – 7.14 (m, 5H), 7.02 – 6.99 (m, 3H), 6.96 – 6.92 (m, 2H), 3.54 – 3.47 (m, 2H), 2.67 – 2.60 (m, 2H).

^{13}C NMR (101 MHz, $CDCl_3$): δ 148.8, 140.7, 139.9, 138.0, 137.8, 137.0, 133.3, 132.8, 130.7, 129.5, 128.4, 128.3, 127.6, 127.1, 126.7, 125.0, 60.6, 39.3.

HRMS (ESI/QTOF) m/z: $[M + Na]^+$ Calcd for $C_{22}H_{19}NNaO_3^+$ 368.1257; Found 368.1258.

IR (ν_{max}, cm^{-1}) 3360 (w), 3015 (w), 2955 (w), 2334 (w), 1615 (w), 1540 (s), 1348 (s), 1027 (m), 756 (s).

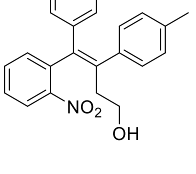

(*E*)-4-(2-nitrophenyl)-4-phenyl-3-(p-tolyl)but-3-en-1-ol (**7b**)

This compound was prepared following general procedure **C** using substrate **22b** (570 mg, 1.0 mmol) as starting material. Yield: 33% over 2 steps (120 mg), isolated as yellow oil. Purification: Flash chromatography (PE/EtOAc, 75:25), $R_f = 0.25$ (PE/EtOAc 70:30).

^{1}H NMR (400 MHz, $CDCl_3$) δ 8.00 (dd, $J = 8.1, 1.3$ Hz, 1H), 7.65 (td, $J = 7.5, 1.3$ Hz, 1H), 7.54 (dd, $J = 7.7, 1.5$ Hz, 1H), 7.49 – 7.45 (m, 1H), 7.07 (d, $J = 8.1$ Hz, 2H), 7.04 – 6.97 (m, 5H), 6.97 – 6.92 (m, 2H), 3.55 – 3.44 (m, 2H), 2.68 – 2.55 (m, 2H), 2.28 (s, 3H).

^{13}C NMR (101 MHz, $CDCl_3$): δ 148.8, 140.1, 138.0, 137.9, 137.6, 136.7, 136.6, 133.3, 132.9, 130.7, 129.3, 129.1, 128.3, 127.6, 126.6, 125.0, 60.8, 39.3, 21.3.

HRMS (ESI/QTOF) m/z: $[M + Na]^+$ Calcd for $C_{23}H_{21}NNaO_3^+$ 382.1414; Found 382.1414.

IR (ν_{max}, cm^{-1}) 3393 (w), 3061 (w), 2920 (w), 2866 (w), 2358 (w), 2332 (w), 1608 (w), 1521 (s), 1442 (m), 1352 (s), 1031 (m), 757 (s).

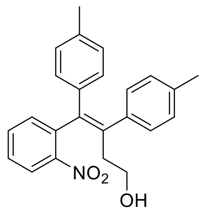

(*E*)-4-(2-nitrophenyl)-3,4-di-p-tolylbut-3-en-1-ol (**7c**)

This compound was prepared following general procedure **C** using substrate **22c** (1.1 g, 1.9 mmol) as starting material. Yield: 48% over 2 steps (340 mg), isolated as yellow solid. Purification: Flash chromatography (PE/EtOAc, 80:20), $R_f = 0.26$ (PE/EtOAc 70:30).

^{1}H NMR (400 MHz, $CDCl_3$) δ 7.99 (dd, $J = 8.2, 1.3$ Hz, 1H), 7.64 (td, $J = 7.5, 1.3$ Hz, 1H), 7.52 (dd, $J = 7.7, 1.5$ Hz, 1H), 7.49 – 7.43 (m, 1H), 7.08 (d, $J = 8.2$ Hz, 2H), 7.02 (d, $J = 7.8$ Hz, 2H), 6.84 – 6.78 (m, 4H), 3.53 – 3.43 (m, 2H), 2.65 – 2.54 (m, 2H), 2.29 (s, 3H), 2.18 (s, 3H).

^{13}C NMR (101 MHz, $CDCl_3$): δ 148.8, 138.3, 137.9, 137.24, 137.17, 136.6, 136.5, 136.3, 133.2, 132.8, 130.5, 129.3, 129.1, 128.4, 128.2, 124.9, 60.8, 39.4, 21.3, 21.2.

HRMS (ESI/QTOF) m/z: $[M + Na]^+$ Calcd for $C_{24}H_{23}NNaO_3^+$ 396.1570; Found 396.1578.

IR (ν_{max}, cm^{-1}) 3381 (w), 2947 (m), 2365 (w), 1525 (s), 1345 (m), 1047 (m), 814 (m), 757 (s).

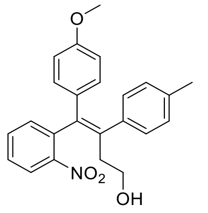

(*E*)-4-(4-methoxyphenyl)-4-(2-nitrophenyl)-3-(p-tolyl)but-3-en-1-ol (**7d**)

This compound was prepared following general procedure **C** using substrate **22d** (1.4 g, 2.4 mmol) as starting material. Yield: 34% over 2 steps (312 mg), isolated as yellow oil. Purification: Flash chromatography (PE/EtOAc, 78:22), $R_f = 0.27$ (PE/EtOAc 70:30).

^{1}H NMR (400 MHz, $CDCl_3$) δ 7.99 (dd, $J = 8.1, 1.3$ Hz, 1H), 7.65 (td, $J = 7.5, 1.3$ Hz, 1H), 7.52 (dd, $J = 7.7, 1.5$ Hz, 1H), 7.49 – 7.44 (m, 1H), 7.08 (d, $J = 8.2$ Hz, 2H), 7.02 (d, $J = 8.0$ Hz, 2H), 6.83 (d, $J = 8.8$ Hz, 1H), 6.54 (d, $J = 8.8$ Hz, 2H), 3.67 (s, 3H), 3.54 – 3.43 (m, 2H), 2.59 (t, $J = 6.7$ Hz, 2H), 2.29 (s, 3H).

^{13}C NMR (101 MHz, $CDCl_3$): δ 158.1, 148.8, 138.3, 137.9, 136.8, 136.6, 136.2, 133.2, 132.8, 132.6, 131.9, 129.4, 129.1, 128.2, 125.0, 113.0, 60.8, 55.2, 39.4, 21.3.

HRMS (ESI/QTOF) m/z: $[M + Na]^+$ Calcd for $C_{24}H_{23}NNaO_4^+$ 412.1519; Found 412.1518.

IR (ν_{max}, cm^{-1}) 3402 (w), 2922 (m), 1604 8m), 1525 (s), 1507 (s), 1349 (m), 1288 (m), 1245 (s), 1173 (m), 1037 (s), 828 (m), 754 (s).

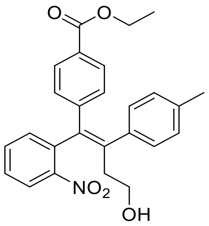

ethyl (*E*)-4-(4-hydroxy-1-(2-nitrophenyl)-2-(p-tolyl)but-1-en-1-yl)benzoate (**7e**)

This compound was prepared following general procedure **C** using substrate **22e** (1.0 g, 1.6 mmol) as starting material. Yield: 22% over 2 steps (145 mg), isolated as yellow solid. Purification: Flash chromatography (PE/EtOAc, 78:22), R_f = 0.20 (PE/EtOAc 70:30).

^{1}H NMR (400 MHz, $CDCl_3$) δ 8.03 (dd, J = 8.2, 1.3 Hz, 1H), 7.70 – 7.66 (m, 3H), 7.55 (dd, J = 7.7, 1.5 Hz, 1H), 7.53 – 7.47 (m, 1H), 7.06 (d, J = 8.3 Hz, 2H), 7.03 – 6.98 (m, 4H), 4.28 (q, J = 7.1 Hz, 2H), 3.55 – 3.43 (m, 2H), 2.68 – 2.55 (m, 2H), 2.29 (s, 3H), 1.32 (t, J = 7.1 Hz, 3H).

^{13}C NMR (101 MHz, $CDCl_3$): δ 166.5, 148.8, 144.9, 139.6, 137.3, 137.23, 137.16, 135.8, 133.4, 133.0, 130.7, 129.3, 128.9, 128.6, 128.4, 125.1, 61.0, 60.6, 39.4, 21.3, 14.4.

HRMS (ESI/QTOF) m/z: $[M + Na]^+$ Calcd for $C_{26}H_{25}NNaO_5^+$ 454.1625; Found 454.1625.

IR (ν_{max}, cm^{-1}) 3454 (w), 2964 (m), 2870 (m), 2365 (w), 1716 (s), 1604 (m), 1528 (s), 1348 (m), 1276 (s), 1107 (m), 1020 (m), 854 (m), 743 (m).

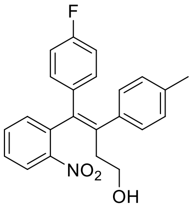

(*E*)-4-(4-fluorophenyl)-4-(2-nitrophenyl)-3-(p-tolyl)but-3-en-1-ol (**7f**)

This compound was prepared following general procedure **C** using substrate **22f** (1.4 g, 2.4 mmol) as starting material. Yield: 45% 2 steps (400 mg), isolated as yellow solid. Purification: Flash chromatography (PE/EtOAc, 80:20), R_f = 0.25 (PE/EtOAc 70:30).

^{1}H NMR (400 MHz, $CDCl_3$) δ 8.01 (dd, J = 8.1, 1.3 Hz, 1H), 7.66 (td, J = 7.5, 1.3 Hz, 1H), 7.54 (dd, J = 7.7, 1.5 Hz, 1H), 7.51 – 7.46 (m, 1H), 7.05 (d, J = 8.5 Hz, 2H), 7.02 (d, J = 8.5 Hz, 2H), 6.93 – 6.87 (m, 2H), 6.73 – 6.68 (m, 2H), 3.54 – 3.41 (m, 2H), 2.65 – 2.54 (m, 2H), 2.29 (s, 3H).

^{13}C NMR (101 MHz, $CDCl_3$): δ 161.36 (d, J = 246.7 Hz), 148.8, 138.2, 137.7, 137.4, 136.9, 136.1 (d, J = 3.6 Hz), 135.5, 133.4, 132.8, 132.31 (d, J = 8.0 Hz), 129.3, 129.2, 128.4, 125.1, 114.62 (d, J = 21.3 Hz), 60.7, 39.3, 21.3.

^{19}F NMR (377 MHz, $CDCl_3$) δ -115.3.

HRMS (ESI/QTOF) m/z: $[M + H]^+$ Calcd for $C_{23}H_{21}NFO_3^+$ 378.1500; Found 378.1499.

IR (ν_{max}, cm^{-1}) 3404 8w), 2928 (m), 2354 (w), 2257 (w), 1604 (m), 1525 (m), 1507 (m), 1352 (m), 1233 (m), 1160 (m), 1046 (m), 904 (s), 732 (s).

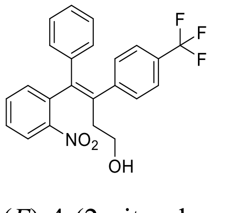

(E)-4-(2-nitrophenyl)-4-phenyl-3-(4-(trifluoromethyl)phenyl)but-3-en-1-ol (**7g**)

This compound was prepared following general procedure **C** using substrate **22g** (830 g, 1.3 mmol) as starting material. Yield: 63% (200 mg), isolated as orange oil. Purification: Flash chromatography (PE/EtOAc, 80:20), R_f = 0.25 (PE/EtOAc 70:30).

^{1}H NMR (400 MHz, $CDCl_3$) δ 8.04 (dd, J = 8.2, 1.3 Hz, 1H), 7.68 (td, J = 7.5, 1.3 Hz, 1H), 7.57 (dd, J = 7.7, 1.5 Hz, 1H), 7.53 – 7.47 (m, 1H), 7.46 (d, J = 8.1 Hz, 2H), 7.31 (d, J = 8.0 Hz, 2H), 7.06 – 6.98 (m, 3H), 6.95 – 6.88 (m, 2H), 3.54 – 3.42 (m, 2H), 2.70 – 2.59 (m, 2H), 1.28 – 1.23 (m, OH).

^{13}C NMR (101 MHz, $CDCl_3$): δ 148.6, 144.8, 139.3, 138.5, 137.3, 136.7, 133.4, 132.6, 130.6, 129.9, 129.1 (q, J = 32.3 Hz), 128.7, 127.9, 127.2, 125.27 (q, J = 4.0 Hz), 125.1, 124.2 (q, J = 271.8 Hz), 60.3, 39.1.

^{19}F NMR (377 MHz, $CDCl_3$) δ -62.5.

HRMS $[M + Na]^+$ Calcd for $C_{23}H_{18}NF_3NaO_3^+$ 436.1131; Found 436.1139.

IR (ν_{max}, cm^{-1}) 3428 (w), 3058 (w), 2929 (w), 2358 (w), 1611 (w), 1521 (s), 1352 (m), 1324 (s), 1166 (m), 1123 (s), 1062 (m), 1019 (m), 846 (m), 753 (m), 706 (m).

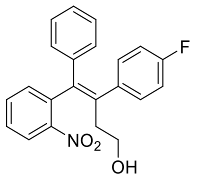

(E)-3-(4-fluorophenyl)-4-(2-nitrophenyl)-4-phenylbut-3-en-1-ol (**7h**)

This compound was prepared following general procedure **C** using substrate **22h** (1.4 g, 2.4 mmol) as starting material. Yield: 44% over 2 steps (380 mg), isolated as yellow solid. Purification: Flash chromatography (PE/EtOAc, 80:20), R_f = 0.10 (PE/EtOAc 80:20). For X-Ray submission, the compound was recrystallized in Acetone/Hexane mixture with slow evaporation at room temperature.

^{1}H NMR (400 MHz, $CDCl_3$) δ 8.02 (dd, J = 8.2, 1.3 Hz, 1H), 7.66 (td, J = 7.5, 1.3 Hz, 1H), 7.54 (dd, J = 7.7, 1.5 Hz, 1H), 7.52 – 7.46 (m, 1H), 7.18 – 7.13 (m, 2H), 7.05 – 7.00 (m, 3H), 6.94 – 6.87 (m, 4H), 3.56 – 3.42 (m, 2H), 2.67 – 2.55 (m, 2H).

^{13}C NMR (101 MHz, $CDCl_3$): δ 161.77 (d, J = 246.3 Hz), 148.6, 139.8, 137.6, 137.3, 136.9, 136.59 (d, J = 3.4 Hz), 133.3, 132.7, 131.10 (d, J = 7.9 Hz), 130.6, 128.4, 127.7, 126.8, 125.0, 115.26 (d, J = 21.3 Hz), 60.4, 39.2.

^{19}F NMR (377 MHz, $CDCl_3$) δ -115.0.

HRMS (ESI/QTOF) m/z: $[M + Na]^+$ Calcd for $C_{22}H_{18}FNNaO_3^+$ 386.1163; Found 386.1165.

IR (ν_{max}, cm^{-1}) 3407 (w), 2927 (w), 2848 (w), 2351 (w), 2250 (w), 1604 (w), 1525 (s), 1507 (s), 1352 (m), 1222 (m), 1160 (m), 1045 (m), 908 (s), 843 (m), 735 (s).

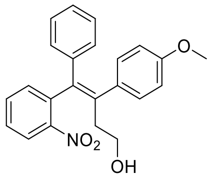

(*E*)-3-(4-methoxyphenyl)-4-(2-nitrophenyl)-4-phenylbut-3-en-1-ol (**7i**)

This compound was prepared following general procedure **C** using substrate **22i** (1.2 g, 2.1 mmol) as starting material. Yield: 24% over 2 steps (150 mg), isolated as yellow oil. Purification: Flash chromatography (PE/EtOAc, 80:20), R_f = 0.18 (PE/EtOAc 70:30).

^{1}H NMR (400 MHz, $CDCl_3$) δ 8.00 (dd, J = 8.1, 1.3 Hz, 1H), 7.65 (td, J = 7.5, 1.3 Hz, 1H), 7.53 (dd, J = 7.7, 1.5 Hz, 1H), 7.50 – 7.44 (m, 1H), 7.10 (d, J = 8.7 Hz, 2H), 7.04 – 7.00 (m, 3H), 6.95 – 6.92 (m, 2H), 6.74 (d, J = 8.7 Hz, 2H), 3.76 (s, 3H), 3.55 – 3.45 (m, 2H), 2.67 – 2.56 (m, 2H).

^{13}C NMR (101 MHz, $CDCl_3$): δ 158.6, 148.8, 140.2, 138.1, 137.5, 136.4, 133.3, 132.9, 130.74, 130.66, 128.3, 127.7, 126.6, 125.0, 113.8, 60.8, 55.3, 39.3.

HRMS (ESI/QTOF) m/z: $[M + Na]^+$ Calcd for $C_{23}H_{21}NNaO_4^+$ 398.1363; Found 398.1377.

IR (ν_{max}, cm^{-1}) 3402 (w), 3030 (w), 2934 (w), 2846 (w), 2358 (w), 2322 (w), 1604 (m), 1525 (s), 1507 (s), 1349 (m), 1284 (m), 1248 (s), 1176 (m), 1033 (m), 832 (m), 756 (s).

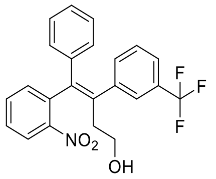

(*E*)-4-(2-nitrophenyl)-4-phenyl-3-(3-(trifluoromethyl)phenyl)but-3-en-1-ol (**7j**)

This compound was prepared following general procedure **C** using substrate **22j** (1.4 g, 2.3 mmol) as starting material. Yield: 55% (500 mg), isolated as white solid. Purification: Flash chromatography (PE/EtOAc, 80:20), R_f = 0.13 (PE/EtOAc 80:20).

^{1}H NMR (400 MHz, $CDCl_3$) δ 8.04 (d, J = 8.2 Hz, 1H), 7.69 (t, J = 7.5 Hz, 1H), 7.58 (d, J = 7.7 Hz, 1H), 7.50 (t, J = 7.8 Hz, 1H), 7.45 – 7.27 (m, 4H), 7.06 – 6.98 (m, 3H), 6.96 – 6.86 (m, 2H), 3.56 – 3.45 (m, 2H), 2.72 – 2.61 (m, 2H).

^{13}C NMR (101 MHz, $CDCl_3$): δ 148.6, 141.7, 139.3, 138.6, 137.3, 136.5, 133.5, 133.1, 132.6, 130.7 (q, J = 32.5 Hz), 130.6, 128.7, 128.6, 127.9, 127.1, 126.3 (q, J = 4.1 Hz), 125.1, 124.0 (q, J = 272.4 Hz), 123.8 (q, J = 4.0 Hz), 60.4, 38.9.

^{19}F NMR (377 MHz, $CDCl_3$) δ -62.7.

HRMS (ESI/QTOF) m/z: $[M + H]^+$ Calcd for $C_{23}H_{19}NF_3O_3^+$ 414.1312; Found 414.1316.

IR (ν_{max}, cm^{-1}) 3394 (w), 3038 (w), 2955 (w), 2886 (w), 2359 (m), 1605 (w), 1524 (s), 1338 (s), 1168 (s), 1124 (s), 1073 (m), 1030 (m), 765 (m).

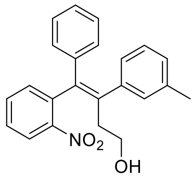

(*E*)-4-(2-nitrophenyl)-4-phenyl-3-(m-tolyl)but-3-en-1-ol (**7k**)

This compound was prepared following general procedure **C** using substrate **22k** (1.4 g, 2.5 mmol) as starting material. Yield: 35% (310 mg), isolated as orange solid. Purification: Flash chromatography (PE/EtOAc, 80:20), R_f = 0.27 (PE/EtOAc 70:30).

^{1}H NMR (400 MHz, $CDCl_3$) δ 8.01 (dd, J = 8.2, 1.3 Hz, 1H), 7.66 (td, J = 7.5, 1.4 Hz, 1H), 7.55 (dd, J = 7.7, 1.5 Hz, 1H), 7.51 – 7.44 (m, 1H), 7.07 (t, J = 7.5 Hz, 1H), 7.04 – 6.81 (m, 8H), 3.55 – 3.45 (m, 2H), 2.68 – 2.57 (m, 2H), 2.24 (s, 3H).

^{13}C NMR (101 MHz, $CDCl_3$): δ 148.8, 140.6, 140.0, 138.1, 137.90, 137.87, 136.8, 133.3, 132.8, 130.6, 130.0, 128.3, 128.2, 127.9, 127.6, 126.7, 125.0, 60.8, 39.4, 21.5.

HRMS (ESI/QTOF) m/z: $[M + Na]^+$ Calcd for $C_{23}H_{21}NNaO_3^+$ 382.1414; Found 382.1413.

IR (ν_{max}, cm^{-1}) 3382 (w), 3021 (w), 2920 (w), 2344 (w), 1604 (w), 1525 (s), 1442 (w), 1348 (s), 1031 (m), 793 (m), 753 (s).

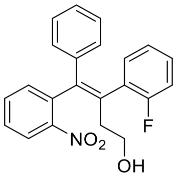

(*E*)-3-(2-fluorophenyl)-4-(2-nitrophenyl)-4-phenylbut-3-en-1-ol (**7l**)

This compound was prepared following general procedure **C** using substrate **22l** (1.2 g, 2.1 mmol) as starting material. Yield: 21% over 2 steps (150 mg), isolated as white solid. Purification: Flash chromatography (PE/EtOAc, 80:20), $R_f = 0.33$ (PE/EtOAc 70:30).

^{1}H NMR (400 MHz, $CDCl_3$) δ 8.03 (dd, J = 8.2, 1.3 Hz, 1H), 7.67 (td, J = 7.4, 1.3 Hz, 1H), 7.62 (dd, J = 7.6, 1.7 Hz, 1H), 7.50 – 7.46 (m, 1H), 7.21 – 7.13 (m, 2H), 7.05 – 6.90 (m, 7H), 3.57 – 3.46 (m, 2H), 2.67 – 2.55 (m, 2H).

^{13}C NMR (101 MHz, $CDCl_3$): δ 160.14 (d, J = 244.5 Hz), 148.5, 139.8, 139.6, 137.0, 133.4, 132.8, 132.5, 131.7 (d, J = 4.0 Hz), 129.7, 129.1 (d, J = 8.1 Hz), 128.5, 128.1 (d, J = 16.3 Hz), 127.7, 127.0, 125.0, 124.1 (d, J = 3.4 Hz), 115.5 (d, J = 22.3 Hz), 60.3, 38.7 (d, J = 1.6 Hz).

^{19}F NMR (377 MHz, $CDCl_3$) δ -114.5.

HRMS HRMS (ESI/QTOF) m/z: $[M + Na]^+$ Calcd for $C_{22}H_{18}FNNaO_3^+$ 386.1163; Found 386.1172.

IR (ν_{max}, cm^{-1}) 3362 (w), 2958 (w), 2893 (w), 2363 (m), 1527 (s), 1487 (m), 1444 (m), 1349 (m), 1215 (m), 1048 (m), 758 (s).

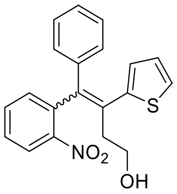

4-(2-nitrophenyl)-4-phenyl-3-(thiophen-2-yl)but-3-en-1-ol (**7m**)

This compound was prepared following the general procedure **C** using substrate **22m** (1.0 g, 1.8 mmol) as starting material. Yield: 82% (514 mg), isolated as yellow oil, mixture of *E* and *Z* isomers. Purification: Flash chromatography (PE/EtOAc, 85:15), $R_f = 0.1$ and 0.16 (PE/EtOAc 80:20).

^{1}H NMR (400 MHz, $CDCl_3$) isomer 1: δ 8.00 (dd, J = 8.2, 1.3 Hz, 1H), 7.66 (td, J = 7.5, 1.3 Hz, 1H), 7.57 (dd, J = 7.7, 1.5 Hz, 1H), 7.48 (ddd, J = 8.6, 7.4, 1.5 Hz, 1H), 7.17 – 7.07 (m, 6H), 6.88 – 6.80 (m, 2H), 3.72 – 3.61 (m, 2H), 2.74-2.61 (m, 2H).

^{1}H NMR (400 MHz, $CDCl_3$) isomer 2: δ 7.82 (dd, J = 8.1, 1.4 Hz, 1H), 7.45 (td, J = 7.6, 1.3 Hz, 1H), 7.40 – 7.28 (m, 7H), 7.14 (dd, J = 5.0, 1.2 Hz, 1H), 6.83 – 6.74 (m, 2H), 3.75 – 3.57 (q, J = 7.1 Hz, 2H), 3.65 (br s, OH), 3.00 – 2.86 (m, 1H), 2.83 – 2.72 (m, 1H).

^{13}C NMR (101 MHz, $CDCl_3$) isomer 1: δ 148.5, 142.71, 140.1, 138.4, 137.6, 133.4, 132.6, 130.7, 130.4, 128.5, 128.1, 127.9, 127.4, 126.8, 125.9, 125.1, 61.1, 40.0.

^{13}C NMR (126 MHz, $CDCl_3$) isomer 2: δ 149.2, 142.9, 139.8, 138.63, 138.62, 133.1, 133.0, 131.0, 129.8, 128.5, 128.1, 127.8, 127.6, 126.9, 126.0, 124.7, 61.7, 38.7.

HRMS (ESI/QTOF) m/z: $[M + Na]^+$ Calcd for $C_{20}H_{17}NNaO_3S^+$ 374.0821; Found 374.0814.

IR (ν_{max}, cm^{-1}) 1604 (w), 1523 (s), 1491 (w), 1442 (w), 1346 (m), 1311 (w), 1294 (w), 1275 (w), 1240 (w), 1080 (w), 1032 (m), 1012 (w), 1001 (w), 908 (w), 852 (m), 833 (w).

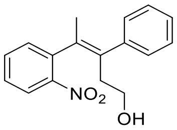

(*E*)-4-(2-nitrophenyl)-3-phenylpent-3-en-1-ol (**7n**)

This compound was prepared following general procedure **C** using substrate **22n** (400 mg, 0.75 mmol) as starting material. Yield: 49% over 2 steps (100 mg), isolated as yellow solid. Purification: Flash chromatography (PE/EtOAc, 80:20), $R_f = 0.4$ (PE/EtOAc 70:30).

^{1}H NMR (400 MHz, $CDCl_3$) δ 8.05 (dd, J = 8.3, 1.3 Hz, 1H), 7.65 (td, J = 7.5, 1.3 Hz, 1H), 7.50 – 7.44 (m, 1H), 7.44 – 7.37 (m, 3H), 7.34 – 7.26 (m, 3H), 3.34 (t, J = 6.5 Hz, 2H), 2.42 – 2.29 (m, 2H), 1.86 (s, 3H).

^{13}C NMR (101 MHz, $CDCl_3$): δ 148.3, 140.8, 139.2, 135.0, 133.6, 133.0, 131.4, 128.7, 128.6, 128.0, 127.1, 124.9, 60.4, 38.7, 22.1.

HRMS (ESI/QTOF) m/z: $[M + Na]^+$ Calcd for $C_{17}H_{17}NNaO_3^+$ 306.1101; Found 306.1108.

IR (ν_{max}, cm^{-1}) 3374 (w), 2954 (w), 2857 (w), 1521 (s), 1439 (m), 1349 (s), 1037 (s), 858 (m), 754 (s), 703 (s).

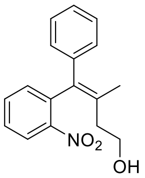

(*Z*)-3-methyl-4-(2-nitrophenyl)-4-phenylbut-3-en-1-ol (**7o**)

This compound was prepared following the general procedure **C** using substrate **22o** (850 mg, 1.7 mmol) as starting material. Yield: 31% over 2 steps (150 mg), isolated as yellow oil. Purification: Flash chromatography (PE/EtOAc, 85:15), $R_f = 0.2$ (PE/EtOAc 75:25).

^{1}H NMR (400 MHz, $CDCl_3$) δ 7.87 (dd, J = 8.2, 1.3 Hz, 1H), 7.60 (td, J = 7.5, 1.3 Hz, 1H), 7.45 – 7.39 (m, 2H), 7.28 – 7.22 (m, 2H), 7.21 – 7.13 (m, 3H), 3.80 – 3.74 (m, 1H), 3.74 – 3.66 (m, 1H), 2.50 (ddd, J = 13.8, 7.7, 6.3 Hz, 1H), 2.26 (dt, J = 13.5, 5.8 Hz, 1H), 1.90 (s, 3H).

^{13}C NMR (101 MHz, $CDCl_3$): δ 149.0, 139.9, 137.6, 135.5, 133.3, 133.0, 132.8, 129.9, 128.1, 128.0, 127.1, 124.8, 60.6, 39.3, 19.4.

HRMS (APCI/QTOF) m/z: $[M + Na]^+$ Calcd for $C_{17}H_{17}NNaO_3^+$ 306.1101; Found 306.1105.

IR (ν_{max}, cm^{-1}) 3374 (w), 2914 (w), 1521 (s), 1345 (m), 1047 (m), 850 (m), 764 (m), 749 (m).

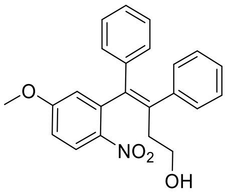

(*E*)-4-(5-methoxy-2-nitrophenyl)-3,4-diphenylbut-3-en-1-ol (**7t**)

This compound was prepared following the general procedure **C** using substrate **22a** (790 mg, 1.4 mmol) as starting material. Yield: 50% over 2 steps (274 mg, 0.73 mmol), isolated as yellow solid. Purification: Flash chromatography (PE/EtOAc, 9:1), R_f = 0.21 (PE/EtOAc 8:2).

^{1}H NMR (400 MHz, $CDCl_3$): δ 8.13 (d, J = 9.1 Hz, 1H), 7.23 – 7.14 (m, 5H), 7.05 – 6.89 (m, 7H), 3.93 (s, 3H), 3.55 – 3.45 (m, 2H), 2.70-2.59 (m, 2H).

^{13}C NMR (101 MHz, $CDCl_3$): δ 163.3, 141.6, 140.7, 140.6, 139.65, 137.6, 137.1, 130.6, 129.5, 128.3, 127.8, 127.6, 127.0, 126.7, 117.7, 113.17, 60.6, 56.1, 39.4.

HRMS (ESI/QTOF) m/z: $[M + Na]^+$ Calculated for $C_{23}H_{21}NNaO_4^+$ 398.1363; Found 398.1355.

IR (ν_{max}, cm^{-1}) 3342 (w), 2925 (w), 2873 (w), 1493 (s), 1446 (m), 1263 (m), 1215 (m), 1144 (m), 1032 (m), 933 (w), 812 (w), 746 (m), 698 (s).

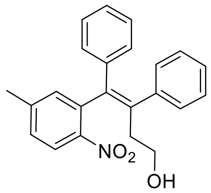

(E)-4-(5-methyl-2-nitrophenyl)-3,4-diphenylbut-3-en-1-ol (**7u**)

This compound was prepared following the general procedure **C** using substrate **22a** (790 mg, 1.4 mmol) as starting material. Yield: 50% over 2 steps (258 mg, 0.72 mmol), isolated as yellow solid. Purification: Flash chromatography (PE/EtOAc, 9:1), R_f = 0.12 (PE/EtOAc 85:15).

^{1}H NMR (400 MHz, $CDCl_3$): δ 7.97 (d, J = 8.4 Hz, 1H), 7.30 (brs, 1H), 7.27 – 7.12 (m, 6H), 7.03 – 6.92 (m, 5H), 3.53 – 3.43 (m, 2H), 2.67-2.56 (m, 2H), 2.47 (s, 3H).

^{13}C NMR (101 MHz, $CDCl_3$): δ 146.4, 144.6, 140.9, 140.0, 137.9, 137.4, 137.3, 133.1, 130.6, 129.5, 129.0, 128.3, 127.6, 127.0, 126.6, 125.2, 60.7, 39.4, 21.7.

HRMS (ESI/QTOF) m/z: $[M + Na]^+$ Calculated for $C_{23}H_{21}NNaO_3^+$ 382.1414; Found 382.1417.

IR (ν_{max}, cm^{-1}) 3235 (w), 2959 (w), 2922 (w), 2156 (w), 1599 (w), 1582 (m), 1518 (s), 1510 (s), 1491 (m), 1442 (m), 1340 (s), 1055 (m), 1043 (m), 1029 (m), 1017 (m), 827 (m).

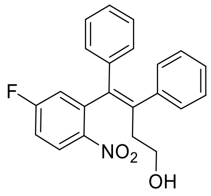

(E)-4-(5-fluoro-2-nitrophenyl)-3,4-diphenylbut-3-en-1-ol (**7v**)

This compound was prepared following the general procedure **C** using substrate **22a** (790 mg, 1.4 mmol) as starting material. Yield: 52% over 2 steps (275 mg, 0.75 mmol), isolated as yellow solid. Purification: Flash chromatography (PE/EtOAc, 9:1), R_f = 0.27 (PE/EtOAc 8:2).

^{1}H NMR (400 MHz, $CDCl_3$): δ 8.10 (dd, J = 9.0, 5.1 Hz, 1H), 7.29 (dd, J = 8.6, 2.8 Hz, 1H), 7.24 – 7.12 (m, 6H), 7.04 – 7.00 (m, 3H), 6.96 – 6.91 (m, 2H), 3.56 – 3.44 (m, 2H), 2.70 – 2.57 (m, 2H).

^{13}C NMR (101 MHz, $CDCl_3$): δ 164.7 (d, J = 257.9 Hz), 144.9 (d, J = 3.0 Hz), 141.1 (d, J = 9.1 Hz), 140.3, 139.4, 138.4, 136.2, 130.6, 129.4, 128.4, 127.8 (d, J = 10.0 Hz), 127.8, 127.2, 127.0, 119.8 (d, J = 23.0 Hz), 115.4 (d, J = 23.2 Hz), 60.4, 39.2.

^{19}F NMR (377 MHz, $CDCl_3$) δ -103.4.

HRMS (ESI/QTOF) m/z: [M + Na]+ Calcd for $C_{22}H_{18}FNNaO_3^+$ 386.1163; Found 386.1168.

IR (ν_{max}, cm^{-1}) 1616 (w), 1579 (m), 1523 (s), 1493 (w), 1473 (w), 1442 (w), 1344 (m), 1304 (w), 1271 (m), 1209 (w), 1072 (w), 1045 (m), 1030 (m), 1014 (w), 908 (m), 868 (w), 835 (w).

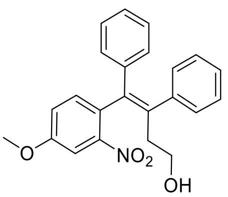

(*E*)-4-(4-methoxy-2-nitrophenyl)-3,4-diphenylbut-3-en-1-ol (**7w**)

This compound was prepared following the general procedure **C** using substrate **22a** (790 mg, 1.4 mmol) as starting material. Yield: 48% over 2 steps (260 mg, 0.69 mmol), isolated as yellow solid. Purification: Flash chromatography (PE/EtOAc, 98:2), R_f = 0.12 (PE/EtOAc 8:2).

^{1}H NMR (400 MHz, $CDCl_3$): δ 8.13 (d, J = 9.1 Hz, 1H), 7.23 – 7.12 (m, 5H), 7.05 – 6.89 (m, 7H), 3.93 (s, 3H), 3.55– 3.44 (m, 2H), 2.70-2.59 (m, 2H).

^{13}C NMR (101 MHz, $CDCl_3$): δ 163.3, 141.6, 140.8, 140.6, 139.6, 137.6, 137.1, 130.6, 129.54, 128.31, 127.82, 127.56, 127.03, 126.7, 117.70, 113.2, 60.6, 56.1, 39.3.

HRMS (ESI/QTOF) m/z: $[M + Na]^+$ Calculated for $C_{23}H_{21}NNaO_4^+$ 398.1363; Found 398.1349.

IR (ν_{max}, cm^{-1}) 2960 (w), 2362 (m), 2339 (w), 1603 (m), 1442 (m), 1334 (s), 1288 (m), 1275 (m), 1248 (m), 1225 (m), 1097 (m), 1070 (m), 1047 (m), 1028 (s), 833 (m).

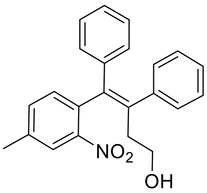

(*E*)-4-(4-methyl-2-nitrophenyl)-3,4-diphenylbut-3-en-1-ol (**7x**)

This compound was prepared following the general procedure **C** using substrate **22a** (790 mg, 1.4 mmol) as starting material. Yield: 68% over 2 steps (356 mg, 0.99 mmol), isolated as yellow oil. Purification: Flash chromatography (PE/EtOAc, 98:2), R_f = 0.19 (PE/EtOAc 8:2).

^{1}H NMR (400 MHz, $CDCl_3$): δ 7.83 (brs, 1H), 7.48 – 7.41 (m, 2H), 7.22 – 7.13 (m, 5H), 7.02 – 6.91 (m, 5H), 3.55 – 3.44 (m, 2H), 2.69 – 2.58 (m, 2H), 2.46 (s, 3H).

^{13}C NMR (101 MHz, $CDCl_3$): δ 148.6, 140.9, 140.1, 138.9, 137.1, 137.0, 134.9, 134.1, 132.5, 130.6, 129.5, 128.3, 127.6, 127.0, 126.6, 125.3, 60.7, 39.4, 21.1.

HRMS (ESI/QTOF) m/z: [M + Na]+ Calculated for $C_{23}H_{21}NNaO_3^+$ 382.1414; Found 382.1410.

IR (ν_{max}, cm^{-1}) 2922 (w), 1525 (s), 1489 (w), 1442 (w), 1350 (m), 1032 (m), 908 (m), 837 (w), 800 (w), 731 (s), 698 (s).

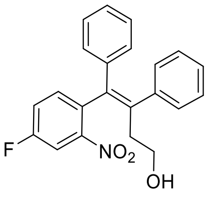

(*E*)-4-(4-fluoro-2-nitrophenyl)-3,4-diphenylbut-3-en-1-ol (**7y**)

This compound was prepared following the general procedure **C** using substrate **22a** (790 mg, 1.4 mmol) as starting material. Yield: 36% over 2 steps (192 mg, 0.53 mmol), isolated as yellow oil. Purification: Flash chromatography (PE/EtOAc, 98:2), R_f = 0.27 (PE/EtOAc 8:2).

^{1}H NMR (400 MHz, $CDCl_3$): δ 7.75 (dd, J = 8.3, 2.6 Hz, 1H), 7.59 (dd, J = 8.5, 5.6 Hz, 1H), 7.40 (td, J = 8.1, 2.7 Hz, 1H), 7.23-7.13 (m, 5H), 7.03-6.97 (m, 3H), 6.93-6.88 (m, 2H), 3.55 – 3.44 (m, 2H), 2.68-2.56 (m, 2H), 2.68-2.56 (m, 2H).

^{13}C NMR (101 MHz, $CDCl_3$): δ 161.0 (d, *J* = 251.7 Hz), 149.0 (d, *J* = 9.0 Hz) 140.4, 139.6, 138.5, 135.9, 134.4 (d, *J* = 7.5 Hz), 133.8 (d, *J* = 4.0 Hz) 130.5, 129.3, 128.2, 127.6, 127.1, 126.7, 120.5 (d, *J* = 20.8 Hz), 112.4 (d, *J* = 26.4 Hz), 60.3, 39.1.

^{19}F NMR (377 MHz, $CDCl_3$) δ -110.9

HRMS (ESI/QTOF) m/z: [M + Na]+ Calcd for $C_{22}H_{18}FNNaO_3^+$ 386.1163; Found 386.1161

IR (ν_{max}, cm^{-1}) 2962 (w), 1533 (s), 1493 (m), 1442 (w), 1348 (m), 1263 (m), 1211 (m), 1045 (m), 1030 (m), 1012 (w), 908 (m), 876 (w), 841 (w), 806 (m).

3.4) Procedure for the synthesis of substrates **7p,q,r,s,z**

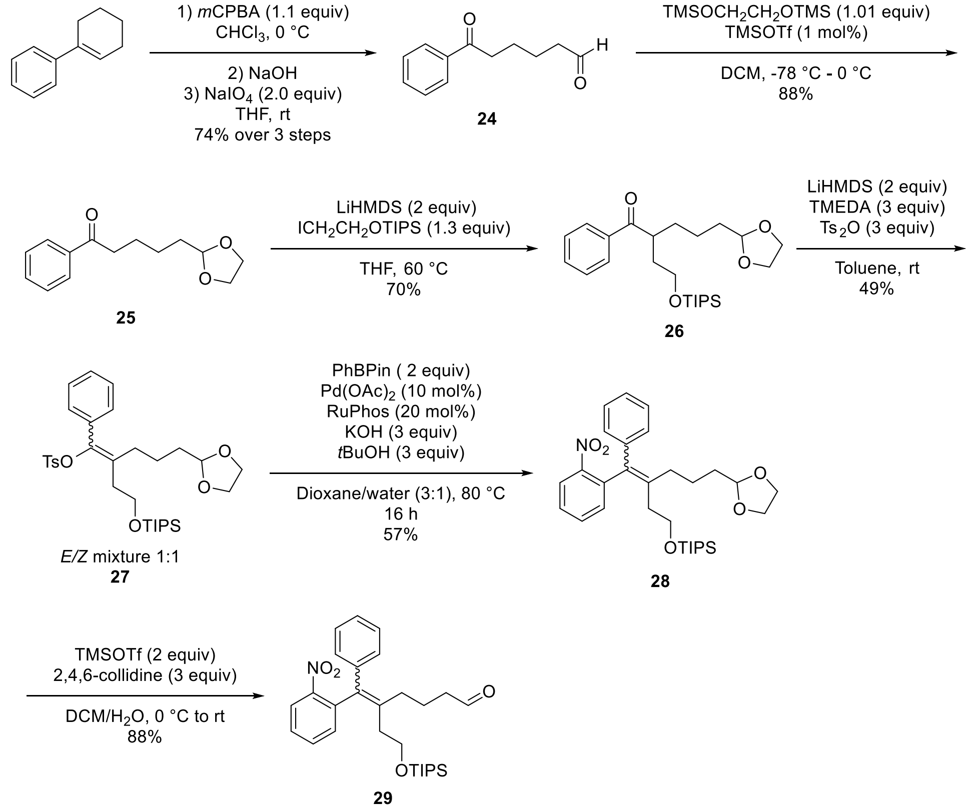

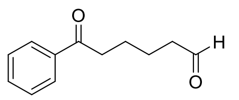

6-oxo-6-phenylhexanal (**24**)

6-Oxo-6-phenylhexanal (**24**) was prepared according to the literature method:[2] To a stirred solution of *m*-CPBA (8.5 g, 34.8 mmol, 1.1 equiv) in $CHCl_3$ (150 mL) was added dropwise 1-phenyl-cyclohexene (5.0 g, 31.6 mmol, 1.0 equiv) at 0 ºC. The mixture was stirred at room temperature for 4 h before 10% NaOH aq. (85 mL) was added at 0 ºC. The organic layer was separated and washed sequentially with saturated

$NaHSO_3$ and brine. Then the solvent was removed, and the residue was dissolved in THF (5.6 mL). The resulting mixture was added dropwise to a solution of sodium periodate (13.5 g, 63 mmol, 2.0 equiv) in THF/H_2O (v/v 2:1, 84 mL). Upon reaction completion after 3 h, the white precipitate was filtered off. The aqueous layer was extracted with Et_2O. The combined organic layers were washed with brine and dried over anhydrous Na_2SO_4. After removal of solvents, the residue was purified by flash chromatography (PE/EtOAc, 9:1, $R_f = 0.32$ (PE/EtOAc, 95:5)), to yield 6-oxo-6-phenylhexanal (**24**) as a white solid (4.4 g, 74 %).

The spectroscopic data are consistent with those reported in the literature.[2]

^{1}H NMR (400 MHz, $CDCl_3$) δ 9.78 (t, J = 1.6 Hz, 1H), 7.96 – 7.92 (m, 2H), 7.59 – 7.52 (m, 1H), 7.49-7.43 (m, 2H), 3.00 (t, J = 6.9 Hz, 2H), 2.50 (td, J = 7.1, 1.6 Hz, 2H), 1.83 – 1.68 (m, 4H).

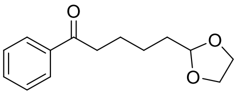

5-(1,3-dioxolan-2-yl)-1-phenylpentan-1-one (**25**)

To a stirred solution of **24** (10 g, 53 mmol, 1.0 equiv) in DCM (100 mL) was added 1,2-bis(trimethylsiloxy)ethane (13 mL, 53 mmol, 1.01 equiv) in dry DCM (250 mL) at room temperature. The solution was cooled to -78 ℃ and TMSOTf (0.14 mL, 5 mmol, 0.01 equiv) was added. The resulting mixture was stirred for 15 minutes at -78 ℃ and then slowly warmed to 0 °C. Pyridine (50 mL) was then added to the reaction mixture followed by addition of DCM. The mixture was washed with water and brine and the organic layer was dried and concentrated under reduced pressure. The residue was subjected to silica gel column chromatography to afford the desired ketone **25**.

Purification: Flash chromatography (PE/EtOAc, 85:15), $R_f = 0.25$ (PE/EtOAc 80:20). Yield: 88% (13 g), isolated as white solid.

^{1}H NMR (400 MHz, $CDCl_3$) δ 7.98 – 7.93 (m, 2H), 7.58 – 7.53 (m, 1H), 7.49 – 7.43 (m, 2H), 4.87 (t, J = 4.7 Hz, 1H), 4.00 – 3.91 (m, 2H), 3.89 – 3.83 (m, 2H), 2.99 (t, J = 7.4 Hz, 2H), 1.84 – 1.70 (m, 4H), 1.56 – 1.48 (m, 2H).

^{13}C NMR (101 MHz, $CDCl_3$): δ 200.4, 137.2, 133.1, 128.7, 128.2, 104.5, 65.0, 38.6, 33.8, 24.3, 23.9.

HRMS (ESI/QTOF) m/z: $[M + Na]^+$ Calcd for $C_{14}H_{18}NaO_3^+$ 257.1148; Found 257.1141.

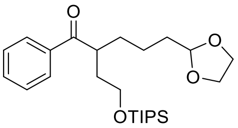

5-(1,3-dioxolan-2-yl)-1-phenyl-2-(2-((triisopropylsilyl)oxy)ethyl)pentan-1-one (**26**)

Glassware and stirring bar were stored in the oven and the reaction was carried out under inert atmosphere and dry conditions. To a solution of ketone **25** (13 g, 68 mmol, 1.0 equiv) in THF (360 mL, 0.2 M) was added LiHMDS (1.0 M in THF, 111 mL, 136 mmol, 2.0 equiv), followed by addition of alkyl iodide (31 g, 96 mmol, 1.4 equiv). The reaction mixture was heated at 65 °C in an oil bath for 2-3 h. After completion of the reaction, a saturated aqueous NH_4Cl solution was added slowly at 0 °C to quench the reaction. The reaction mixture was extracted with EtOAc. The combined organic layers were washed with brine, dried over Na_2SO_4, filtered and concentrated under reduced pressure. The residue was subjected to silica gel column chromatography to afford the desired ketone **26**.

Yield: 41% (12 g), isolated as amorphous solid. Purification: Flash chromatography (PE/EtOAc, 92:8), $R_f = 0.37$ (PE/EtOAc 85:15).

^{1}H NMR (400 MHz, $CDCl_3$) δ 8.03 – 7.99 (m, 2H), 7.57 – 7.51 (m, 1H), 7.47 – 7.40 (m, 2H), 4.79 (t, J = 4.8 Hz, 1H), 3.94-3.87 (m, 2H), 3.84-3.70 (m, 4H), 3.66-3.60 (m, 1H), 2.07 – 1.98 (m, 1H), 1.88 – 1.79 (m, 1H), 1.73-1.36 (m, 6H), 1.08 – 0.98 (m, 21H).

^{13}C NMR (101 MHz, $CDCl_3$): δ 204.5, 137.6, 133.0, 128.7, 128.6, 104.5, 65.0, 61.1, 42.4, 35.7, 34.1, 32.2, 22.3, 18.1, 12.1.

HRMS (ESI/QTOF) m/z: $[M + Na]^+$ Calcd for $C_{25}H_{42}NaO_4Si^+$ 457.2750; Found 457.2755.

IR (ν_{max}, cm^{-1}) 2944 (m), 2868 (m), 1676 (m), 1466 (m), 1364 (m), 1240 (m), 1102 (s), 1000 (s), 884 (s).

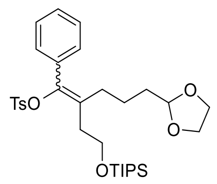

5-(1,3-dioxolan-2-yl)-1-phenyl-2-(2-((triisopropylsilyl)oxy)ethyl)pent-1-en-1-yl 4-methylbenzenesulfonate (**27**), mixture of *Z* and *E* isomers

Glassware and stirring bar were stored in the oven and the reaction was carried out under inert atmosphere and dry conditions. To a solution of LiHMDS (1.0 M in toluene, 12.6 mL, 12.6 mmol, 2.0 equiv) was added dropwise TMEDA (2.0 mL, 18.9 mmol, 3.0 equiv). The reaction mixture was placed in a water bath at 10 °C and a solution of ketone **26** (2.7 g, 6.3 mmol, 1.0 equiv) in toluene (0.3 M) was added via syringe. After stirring for 20 min, Ts_2O (6.2 g, 19 mmol, 3.0 equiv) was added over 5 min. The reaction mixture was stirred for 2-3 h. After completion of the reaction, water was added to quench the reaction. The reaction mixture was extracted with EtOAc. The combined organic layers were dried over Na_2SO_4, filtered and concentrated under reduced pressure. The residue was subjected to silica gel column chromatography to afford the desired enol tosylate **27** as a mixture of *Z* and *E* isomers.

Yield: 59% (2.2 g), isolated as colorless oil. Purification: Flash chromatography (PE/EtOAc, 95:5), R_f = 0.39 (PE/EtOAc 80:20).

^{1}H NMR (400 MHz, $CDCl_3$) 2 isomers in a ratio of around 1:1: δ 7.42 – 7.37 (m, 4H), 7.20 – 7.12 (m, 10H), 7.06 – 7.04 (m, 4H), 4.88 (t, J = 4.6 Hz, 1H), 4.75 (t, J = 4.3 Hz, 1H), 4.00-3.96 (m, 2H), 3.93-3.84 (m, 6H), 3.82-3.78 (m, 2H), 3.70 (t, J = 7.0 Hz, 2H), 2.55 (t, J = 6.7 Hz, 2H), 2.39-2.28 (m, 4H), 2.33 (s, 6H), 2.16 – 2.12 (m, 2H), 1.74 – 1.67 (m, 2H), 1.66-1.51 (m, 6H), 1.13 - 0.97 (m, 42H).

^{13}C NMR (101 MHz, $CDCl_3$): δ 144.1, 143.7, 143.2, 134.7, 134.66, 133.9, 133.8, 131.6, 130.7, 130.0, 129.8, 129.3, 128.28, 128.25, 127.94, 127.93, 127.89, 127.87, 104.6, 104.3, 64.99, 64.96, 62.2, 61.9, 33.8, 33.7, 33.5, 32.6, 31.2, 29.5, 22.8, 22.2, 21.7, 18.2, 18.1, 12.1, 12.0.

HRMS (ESI/QTOF) m/z: $[M + Na]^+$ Calcd for $C_{32}H_{48}NaO_6SSi^+$ 611.2833; Found 611.2850.

IR (ν_{max}, cm^{-1}) 2940 (m), 2865 (m), 1463 (s), 1343 (s), 992 (m), 850 (m).

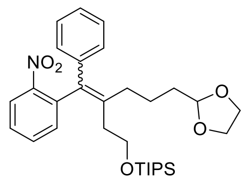

((6-(1,3-dioxolan-2-yl)-3-((2-nitrophenyl)(phenyl)methylene)hexyl)oxy)triisopropylsilane (**28**)

Glassware and stirring bar were stored in the oven and the reaction was carried out under inert atmosphere and dry conditions. Dioxane and water were degassed with Freeze-Pump-Thaw technique. To a solution of enol tosylate **27** (790 mg, 1.3 mmol, 1.0 equiv) in dioxane/water (22 mL, 3:1, 0.06 M) were added 2-nitrophenylboronic acid pinacol ester (670 mg, 2.6 mmol, 2.0 equiv), $Pd(OAc)_2$ (30 mg, 0.13 mmol, 10 mol%), RuPhos (125 mg, 0.27 mmol, 20 mol%), KOH (230 mg, 4.0 mmol, 3.0 equiv) and *t*-BuOH (0.38 mL, 4.0 mmol, 3.0 equiv). The reaction mixture was heated at 80 °C overnight, then water was added, followed by extraction with EtOAc (3 times). The organic layers were combined, washed with brine and dried over sodium sulfate. The residue was subjected to silica gel column chromatography to afford the desired alkene **28** as a mixture of *Z* and *E* isomers.

Yield: 59% (430 mg), isolated as yellow oil. Purification: Flash chromatography (PE/EtOAc, 95:5), R_f = 0.19 (PE/EtOAc 90:10).

Isomer 1:

^{1}H NMR (400 MHz, $CDCl_3$) δ 7.86 (dd, J = 8.2, 1.3 Hz, 1H), 7.54 (td, J = 7.5, 1.3 Hz, 1H), 7.42 (dd, J = 7.7, 1.5 Hz, 1H), 7.36 (ddd, J = 8.6, 7.4, 1.5 Hz, 1H), 7.25 – 7.15 (m, 5H), 4.76 – 4.74 (m, 1H), 3.93 – 3.88 (m, 2H), 3.85 – 3.77 (m, 2H), 3.76 – 3.62 (m, 2H), 2.38 – 2.23 (m, 4H), 1.62-1.57 (m, 4H), 1.04-0.93 (m, 21H).

^{13}C NMR (101 MHz, $CDCl_3$): δ 155.4, 148.9, 140.5, 137.9, 135.2, 132.9, 132.8, 129.8, 128.2, 127.8, 127.0, 124.6, 104.5, 64.9, 61.7, 35.9, 33.5, 31.7, 22.7, 18.1, 12.1.

IR (ν_{max}, cm^{-1}) 2923 (m), 2870 (m), 2359 (s), 1526 (m), 1097 (m), 668 (m).

Isomer 2:

^{1}H NMR (400 MHz, $CDCl_3$) δ 7.87 (dd, J = 8.1, 1.3 Hz, 1H), 7.53 (td, J = 7.5, 1.3 Hz, 1H), 7.38-7.32 (m, 2H), 7.27 – 7.16 (m, 5H), 4.73-4.71 (m, 1H), 3.93 – 3.77 (m, 4H), 3.72-3.67 (m, 2H), 2.51 – 2.46 (m, 2H), 2.12-1.97 (m, 2H), 1.63-1.48 (m, 4H), 1.01-0.95 (m, 21H).

^{13}C NMR (101 MHz, $CDCl_3$): δ 148.9, 140.6, 137.9, 135.0, 132.8, 132.5, 129.8, 128.3, 127.8, 127.0, 124.5, 104.4, 64.9, 61.9, 34.8, 33.7, 33.2, 22.5, 18.2, 12.1.

HRMS (ESI/QTOF) m/z: $[M + Na]^+$ Calcd for $C_{31}H_{45}NNaO_5Si^+$ 562.2959; Found 562.2965.

IR (ν_{max}, cm^{-1}) 2924 (s), 2865 (s), 1526 (s), 1457 (m), 1352 (m), 1097 (s), 765 (s).

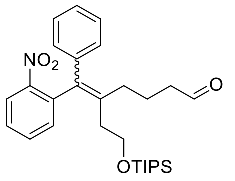

5-((2-nitrophenyl)(phenyl)methylene)-7-((triisopropylsilyl)oxy)heptanal (**29**)

To a solution of dioxolane **28** (2.21 g, 2.77 mmol) in CH_2Cl_2 (50 mL) were added 2,4,6-trimethylpyridine (1.10 mL, 8.31 mmol) and TMSOTf (1.00 mL, 5.54 mmol) at 0 °C. After stirring for 1 h at this temperature, water (50 mL) was added and stirring was continued at ambient temperature for 2 h. The layers were separated and the aqueous phase was extracted with EtOAc. The combined extracts were dried over Na_2SO_4, filtered and concentrated. The residue was subjected to silica gel column chromatography to afford the desired aldehyde **29**.

Yield: 88% (1.5 g), isolated as colorless oil. Purification: Flash chromatography (PE/EtOAc, 95:5), R_f = 0.19 (PE/EtOAc 90:10).

Isomer 1:

^{1}H NMR (400 MHz, $CDCl_3$) δ 9.64 (t, J = 1.6 Hz, 1H), 7.88-7.85 (m, 1H), 7.56-7.51 (m, 1H), 7.40 – 7.35 (m, 2H), 7.29-7.18 (m, 5H), 3.75 – 3.64 (m, 2H), 2.42 – 2.25 (m, 6H), 1.82 – 1.73 (m, 2H), 1.03 – 0.93 (m, 21H).

^{13}C NMR (101 MHz, $CDCl_3$): δ 202.5, 148.8, 140.5, 137.6, 137.3, 135.7, 132.9, 132.7, 129.6, 128.4, 127.9, 127.2, 124.6, 61.7, 43.3, 35.8, 31.0, 20.6, 18.1, 12.0.

IR (ν_{max}, cm^{-1}) 2924 (s), 2864 (s),2359 (m), 1724 (m), 1526 (s), 1457 (m), 1351 (m), 1097 (m), 882 (m).

Isomer 2:

^{1}H NMR (400 MHz, $CDCl_3$) δ 9.65 (t, J = 1.7 Hz, 1H), 7.88 (dd, J = 8.2, 1.2 Hz, 1H), 7.56 (td, J = 7.6, 1.3 Hz, 1H), 7.39 (td, J = 7.8, 1.4 Hz, 1H), 7.34 (dd, J = 7.7, 1.4 Hz, 1H), 7.27 – 7.18 (m, 5H), 3.73 –

3.69 (m, 2H), 2.57-2.41 (m, 2H), 2.36-2.30 (m, 2H), 2.13 – 2.01 (m, 2H), 1.76 (p, J = 7.5 Hz, 2H), 1.05-0.91 (m, 21H).

^{13}C NMR (101 MHz, $CDCl_3$): δ 202.3, 148.8, 140.3, 137.6, 137.2, 135.7, 132.9, 132.4, 129.8, 128.3, 128.0, 127.1, 124.7, 61.9, 43.5, 34.7, 32.7, 20.5, 18.1, 12.0.

IR (ν_{max}, cm^{-1}) 2924 (s), 2864 (s), 1725 (m), 1526 (s), 1462 (m), 1352 (m), 1097 (s), 882 (m).

HRMS (ESI/QTOF) m/z: $[M + Na]^+$ Calcd for $C_{29}H_{41}NNaO_4Si^+$ 518.2698; Found 518.2693.

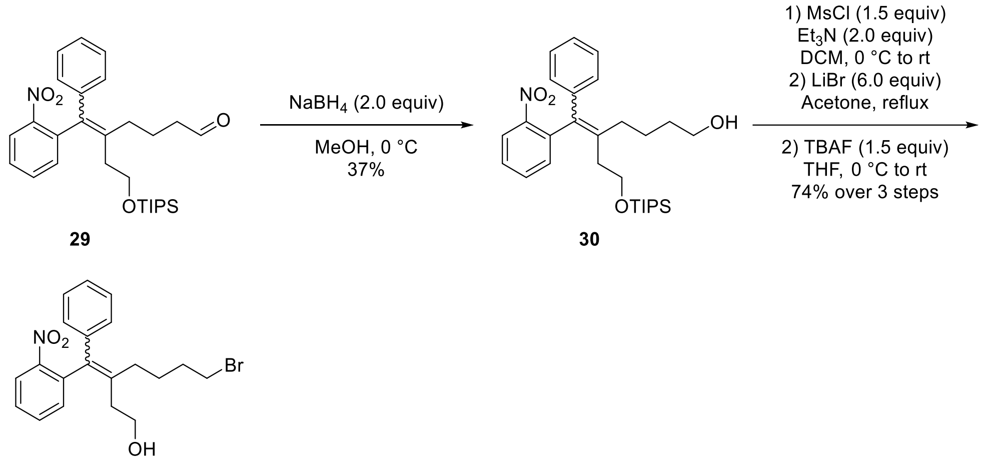

7z

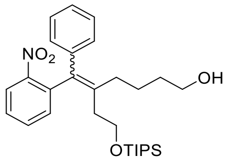

5-((2-nitrophenyl)(phenyl)methylene)-7-((triisopropylsilyl)oxy)heptan-1-ol (**30**)

To a solution of aldehyde **29** (1.2 g, 2.4 mmol, 1.0 equiv) in MeOH (24 mL, 0.1 M) was added sodium borohydride (180 mg, 4.8 mmol, 2 equiv) at 0 °C, and the resulting mixture was stirred for 2 hours. The solvent was removed under reduced pressure, and a saturated aqueous NH_4Cl solution was added. The reaction mixture was extracted with ethyl acetate, and the combined organic layers were washed with water, and brine. The organic layer was then dried over sodium sulfate, and concentrated under reduced pressure. The residue was subjected to silica gel column chromatography to afford the desired alcohol **30**.

Yield: 37% (450 mg), isolated as yellow oil. Purification: Flash chromatography (PE/EtOAc, 85:15), R_f = 0.32 (DCM).

Isomer 1:

^{1}H NMR (400 MHz, $CDCl_3$) δ 7.86 (dd, J = 8.2, 1.3 Hz, 1H), 7.54 (td, J = 7.5, 1.3 Hz, 1H), 7.42 – 7.34 (m, 2H), 7.29 – 7.18 (m, 5H), 3.75 – 3.64 (m, 2H), 3.52 (t, J = 6.1 Hz, 2H), 2.38 – 2.19 (m, 4H), 1.57-1.43 (m, 4H), 1.05-0.92 (m, 21H).

^{13}C NMR (101 MHz, $CDCl_3$): δ 148.9, 140.7, 138.1, 137.8, 135.0, 132.8, 129.7, 128.2, 127.8, 127.1, 124.5, 62.8, 61.8, 36.0, 32.4, 31.5, 24.4, 18.1, 12.1.

IR (ν_{max}, cm^{-1}) 2940 (m), 2864 (m), 1605 (s), 1570 (m), 1491 (m); 1442 (m), 1381 (m), 1093 (s), 1067 (s), 920 (m), 785 (m).

Isomer 2:

^{1}H NMR (400 MHz, $CDCl_3$) δ 7.86 (dd, J = 8.1, 1.3 Hz, 1H), 7.53 (td, J = 7.5, 1.4 Hz, 1H), 7.38-7.32 (m, 2H), 7.30 – 7.16 (m, 5H), 3.75 – 3.62 (m, 2H), 3.56 – 3.46 (m, 2H), 2.54-2.40 (m, 2H), 2.12 – 1.89 (m, 2H), 1.53 – 1.35 (m, 4H), 1.04-0.91 (m, 21H).

^{13}C NMR (101 MHz, $CDCl_3$): δ 148.9, 140.6, 138.0, 137.9, 134.9, 132.8, 132.5, 129.8, 128.3, 127.8, 127.0, 124.5, 62.8, 62.0, 34.9, 33.1, 32.7, 24.3, 18.1, 12.1.

IR (ν_{max}, cm^{-1}) 2941 (m), 2864 (m), 2359 (w), 1491 (s), 1461 (m), 1096 (s), 1013 (s), 919 (m), 850 (m), 764 (m).

HRMS (ESI/QTOF) m/z: $[M + Na]^+$ Calcd for $C_{29}H_{43}NNaO_4Si^+$ 520.2854; Found 520.2856.

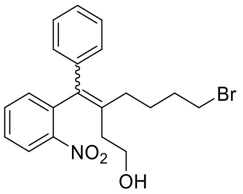

7-bromo-3-((2-nitrophenyl)(phenyl)methylene)heptan-1-ol (**7z**)

To a solution of alcohol **30** (400 mg, 0.8 mmol, 1.0 equiv) in DCM (8 mL, 0.1 M) were added MsCl (0.1 mL, 1.2 mmol, 1.5 equiv) and triethylamine (0.22 mL, 1.6 mmol, 2.0 equiv) at 0 °C. After being stirred for 3 h at room temperature, the reaction mixture was quenched with 2 M HCl and extracted with ethyl acetate. The combined organic extracts were washed with brine, dried over Na_2SO_4, filtered and concentrated under reduced pressure. The residue was subjected to the next step without purification.

To a solution of crude mesylate (200 mg, 0.35 mmol, 1.0 equiv) in acetone (1.1 mL, 0.3 M) was added LiBr (180 mg, 2.1 mmol, 6.0 equiv) at room temperature. The reaction mixture was heated at 50 °C for 3 h, then water was added. The mixture was extracted with EtOAc (3 times). The combined organic extracts were washed with brine, dried over Na_2SO_4, filtered and concentrated under reduced pressure. The residue was subjected to the next step without purification.

To a solution of crude bromide (270 mg, 0.5 mmol, 1.0 equiv) in THF (5 mL, 0.1 M) was added dropwise TBAF (1 M in THF, 0.8 mL, 0.8 mmol, 1.5 equiv) at 0 °C. After being stirred for 3 h at room temperature, the reaction mixture was quenched with saturated aqueous $NaHCO_3$ and extracted with EtOAc. The organic layers were combined, washed with brine and dried over sodium sulfate, filtered and evaporated *in vacuo*. The residue was subjected to silica gel column chromatography (PE:EtOAc) to give alcohol **7z**.

Yield: 74% (93 mg), isolated as yellow oil, mixture of *E* and *Z* isomers. Purification: Flash chromatography (PE/EtOAc, 70:30), R_f = 0.36 and 0.24 (PE/EtOAc 70:30).

Isomer 1:

^{1}H NMR (400 MHz, $CDCl_3$) δ 7.90 (dd, J = 8.2, 1.3 Hz, 1H), 7.56 (td, J = 7.5, 1.3 Hz, 1H), 7.40 (ddd, J = 8.2, 7.4, 1.5 Hz, 1H), 7.33 (dd, J = 7.7, 1.5 Hz, 1H), 7.31 – 7.20 (m, 5H), 3.74 – 3.61 (m, 2H), 3.29-3.23 (m, 2H), 2.61 (dt, J = 13.9, 7.0 Hz, 1H), 2.42 (dt, J = 13.6, 6.3 Hz, 1H), 2.12 – 1.95 (m, 2H), 1.78 – 1.69 (m, 2H), 1.61-1.50 (m, 2H).

^{13}C NMR (101 MHz, $CDCl_3$): δ 148.7, 140.4, 137.5, 136.9, 136.2, 133.1, 132.3, 129.7, 128.5, 128.1, 127.4, 124.7, 61.2, 34.5, 33.5, 32.4, 31.8, 26.4.

Isomer 2:

^{1}H NMR (400 MHz, $CDCl_3$) δ 7.87 (dd, J = 8.5, 1.3 Hz, 1H), 7.60 (td, J = 7.5, 1.4 Hz, 1H), 7.42 – 7.38 (m, 2H), 7.29 – 7.16 (m, 5H), 3.77-3.62 (m, 2H), 3.27 (t, J = 6.6 Hz, 2H), 2.47 (dt, J = 13.7, 6.8 Hz, 1H), 2.37 – 2.20 (m, 3H), 1.77 – 1.72 (m, 2H), 1.62 – 1.56 (m, 2H).

^{13}C NMR (101 MHz, $CDCl_3$): δ 148.9, 139.9, 137.7, 136.9, 136.8, 133.2, 132.6, 129.7, 128.3, 128.2, 127.3, 124.9, 60.8, 35.7, 33.6, 32.3, 30.3, 26.6.

HRMS (Sicrit plasma/LTQ-Orbitrap) m/z: $[M + H]^+$ Calcd for $C_{20}H_{23}BrNO_3^+$ 404.0856; Found 404.0858.

IR (ν_{max}, cm^{-1}) 2922 (w), 2849 (w), 2363 (w), 1716 (w), 1357 (w), 1062 (w), 910 (s), 728 (s).

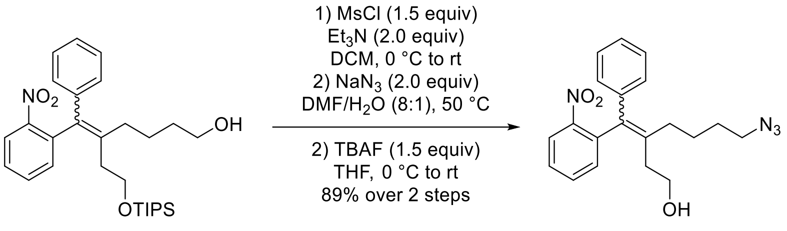

30 **7p**

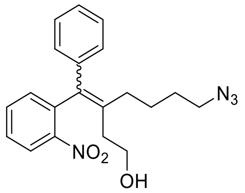

7-azido-3-((2-nitrophenyl)(phenyl)methylene)heptan-1-ol (**7p**)

To a solution of alcohol **30** (400 mg, 0.8 mmol, 1.0 equiv) in DCM (8 mL, 0.1 M) were added MsCl (0.1 mL, 1.2 mmol, 1.5 equiv) and triethylamine (0.22 mL, 1.6 mmol, 2.0 equiv) at 0 °C. After being stirred for 3 h at room temperature, the reaction mixture was quenched with 2 M HCl and extracted with ethyl acetate. The combined organic extracts were washed with brine, dried over Na_2SO_4, filtered and concentrated under reduced pressure. The residue was subjected to the next step without purification.

To a solution of crude mesylate (200 mg, 0.35 mmol, 1.0 equiv) in a mixture of DMF/H_2O (8:1, 1.1 mL, 0.3 M) was added NaN_3 (45 mg, 0.7 mmol, 2.0 equiv) at room temperature. The reaction mixture was heated at 50 °C for 3 h, then water was added. The reaction mixture was extracted with EtOAc (3 times). The combined organic extracts were washed with brine, dried over Na_2SO_4, filtered and concentrated under reduced pressure. The residue was subjected to the next step without purification.

To a solution of crude azide (160 mg, 0.3 mmol, 1.0 equiv) in THF (3 mL, 0.1 M) was added dropwise TBAF (1 M in THF, 0.46 mL, 0.46 mmol, 1.5 equiv) at 0 °C. After being stirred at room temperature for 3 h, the reaction mixture was quenched with saturated aqueous $NaHCO_3$ and extracted with EtOAc. The organic layers were combined, washed with brine and dried over sodium sulfate, filtered and evaporated *in vacuo*. The residue was subjected to silica gel column chromatography (PE:EtOAc) to give alcohol **7p**.

Yield: 89% (100 mg), isolated as yellow oil, mixture of *E* and *Z* isomers. Purification: Flash chromatography (PE/EtOAc, 70:30), R_f = 0.31 and 0.21 (PE/EtOAc 70:30).

Isomer 1:

^{1}H NMR (400 MHz, $CDCl_3$) δ 7.88 (dd, *J* = 8.2, 1.3 Hz, 1H), 7.54 (td, *J* = 7.5, 1.3 Hz, 1H), 7.38 (ddd, *J* = 8.2, 7.4, 1.5 Hz, 1H), 7.30 (dd, *J* = 7.6, 1.4 Hz, 1H), 7.28 – 7.16 (m, 5H), 3.72 – 3.60 (m, 2H), 3.15-3.11 (m, 2H), 2.59 (dt, *J* = 13.9, 7.0 Hz, 1H), 2.40 (dt, *J* = 13.6, 6.3 Hz, 1H), 2.10 – 1.94 (m, 2H), 1.50 – 1.39 (m, 4H).

^{13}C NMR (101 MHz, $CDCl_3$): δ 148.8, 140.4, 137.5, 136.9, 136.2, 133.1, 132.3, 129.7, 128.5, 128.1, 127.4, 124.6, 61.2, 51.2, 34.4, 32.2, 28.7, 25.1.

Isomer 2:

^{1}H NMR (400 MHz, $CDCl_3$) δ 7.87 (dd, *J* = 8.5, 1.3 Hz, 1H), 7.59 (td, *J* = 7.5, 1.4 Hz, 1H), 7.43 – 7.38 (m, 2H), 7.30 – 7.16 (m, 5H), 3.76-3.61 (m, 2H), 3.14 – 3.11 (m, 2H), 2.46 (dt, *J* = 13.7, 6.9 Hz, 1H), 2.37 – 2.20 (m, 3H), 1.52 – 1.46 (m, 4H).

^{13}C NMR (101 MHz, $CDCl_3$): δ 148.9, 140.1, 137.6, 136.8, 136.8, 133.2, 132.6, 129.7, 128.3, 128.2, 127.3, 124.9, 60.8, 51.1, 35.7, 30.7, 28.4, 25.2.

Mixture of 2 isomers (1:0.6): spectra 130,131

^{1}H NMR (400 MHz, $CDCl_3$) δ 7.90 – 7.85 (m, 1 + 0.6H), 7.60 – 7.53 (m, 1 + 0.6H), 7.41 – 7.37 (m, 1 + 1.2H), 7.32 (dd, J = 7.7, 1.5 Hz, 1H), 7.29 – 7.13 (m, 5 + 3H), 3.74 – 3.61 (m, 2 + 1.2H), 3.17 – 3.10 (m, 2 + 1.2H), 2.59 (dt, J = 13.9, 7.0 Hz, 1H), 2.48 - 2.38 (m, 1.6H), 2.36 – 2.20 (m, 1.8H), 2.15 – 1.94 (m, 2H), 1.67-1.38 (m, 4H + 2.4H).

^{13}C NMR (101 MHz, $CDCl_3$): δ 148.9, 148.8, 140.4, 140.0, 137.6, 137.5, 137.0, 136.83, 136.81, 136.2, 133.14, 133.06, 132.6, 132.3, 129.74, 129.68, 128.5, 128.3, 128.2, 128.1, 127.3, 124.9, 124.6, 61.2, 60.8, 51.2, 51.1, 35.7, 34.5, 32.2, 30.7, 28.7, 28.4, 25.2, 25.1.

HRMS (Sicrit plasma/LTQ-Orbitrap) m/z: $[M + H]^+$ Calcd for $C_{20}H_{23}N_4O_3^+$ 367.1765; Found 367.1764.

IR (ν_{max}, cm^{-1}) 2922 (m), 2849 (w), 2101 (m), 1734 (w), 1523 (w), 1353 (w), 914 (s), 739 (s).

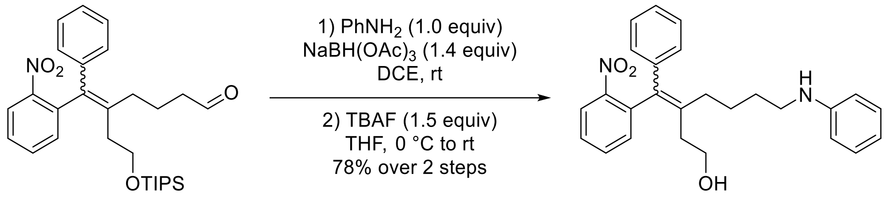

29 **7q**

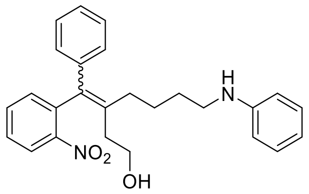

3-((2-nitrophenyl)(phenyl)methylene)-7-(phenylamino)heptan-1-ol (**7q**)

To a solution of aldehyde **29** (200 mg, 0.4 mmol, 1.0 equiv) and amine (0.04 mL, 0.4 mmol, 1.0 equiv) in DCE (2.0 mL, 0.2 M) was added portionwise $NaBH(OAc)_3$ (120 mg, 0.56 mmol, 1.4 equiv) at 0 °C under inert atmosphere of Ar. After being stirred for 2 h at room temperature, the reaction mixture was quenched with 1.0 M solution of NaOH and extracted with EtOAc. The organic layers were combined, washed with brine and dried over sodium sulfate, filtered and evaporated *in vacuo*. The residue was subjected to the next step without further purification.

To a solution of crude amine (200 mg, 0.35 mmol, 1.0 equiv) in THF (3.5 mL, 0.1 M) was added dropwise TBAF (1 M in THF, 0.52 mL, 0.52 mmol, 1.5 equiv) at 0 °C. After being stirred for 3 h at room temperature, the reaction mixture was quenched with saturated aqueous $NaHCO_3$ and extracted with EtOAc. The organic layers were combined, washed with brine and dried over sodium sulfate, filtered and evaporated *in vacuo*. The residue was subjected to silica gel column chromatography (PE:EtOAc) to give alcohol **7q**.

Yield: 78% over 2 steps (131 mg), isolated as orange oil, mixture of *E* and *Z* isomers. Purification: Flash chromatography (PE/EtOAc, 70:30), R_f = 0.21 and 0.32 (PE/EtOAc 70:30).

^{1}H NMR (400 MHz, $CDCl_3$) (ratio of the 2 isomers 1 : 0.3) δ 7.91 – 7.84 (m, 1 + 0.3H), 7.58 (td, J = 7.5, 1.4 Hz, 0.3H), 7.50 (td, J = 7.6, 1.3 Hz, 1H), 7.42 – 7.13 (m, 9 + 2.7H), 6.71 – 6.66 (m, 1 + 0.3H), 6.56 – 6.51 (m, 2 + 0.6H), 3.75 – 3.62 (m, 2 + 0.6H), 2.98 – 2.95 (m, 2 + 0.6H), 2.63 – 2.21 (m, 2 + 1.2H), 2.15 – 1.93 (m, 2H), 1.60 – 1.42 (m, 4.0 + 1.2H).

^{13}C NMR (101 MHz, $CDCl_3$) 2 isomers δ 148.9, 148.8, 148.44, 148.4, 140.5, 140.1, 137.7, 137.6, 137.2, 137.19, 136.6, 136.0, 133.1, 133.0, 132.7, 132.3, 129.8, 129.7, 129.4, 128.5, 128.3, 128.1, 128.0, 127.3,

124.9, 124.6, 117.4, 117.3, 112.92, 112.89, 61.2, 60.9, 43.62, 43.60, 35.8, 34.6, 32.4, 30.9, 29.3, 29.0, 25.6, 25.5.

HRMS (ESI/QTOF) m/z: $[M + H]^+$ Calcd for $C_{26}H_{29}N_2O_3^+$ 417.2173; Found 417.2176.

IR (ν_{max}, cm^{-1}) 3417 (w), 2943 (w), 2853 (w), 2347 (w), 2258 (w), 1600 (m), 1521 (m), 1352 (m), 1033 (m), 907 (s), 739 (s).

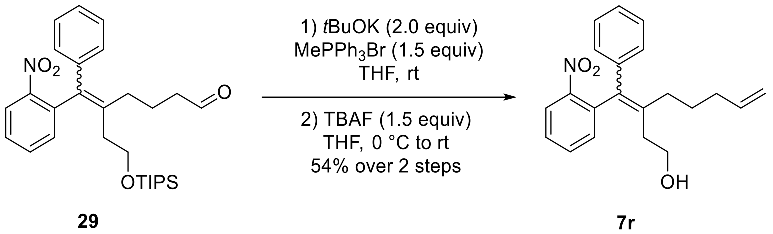

29 **7r**

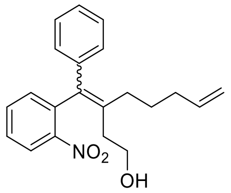

3-((2-nitrophenyl)(phenyl)methylene)oct-7-en-1-ol (**7r**)

To a solution of *t*-BuOK (86 mg, 0.8 mmol, 2.0 equiv) in THF (4 mL, 0.1 M) was added methyltriphenylphosphonium bromide (210 mg, 0.6 mmol, 1.5 equiv) at 0 °C. After 20 min, the aldehyde **29** (190 mg, 0.4 mmol, 1.0 equiv) was added and the mixture was stirred at room temperature for 2 h. After completion of the reaction, a saturated aqueous NH_4Cl solution was added slowly at 0 °C to quench the reaction. The reaction mixture was extracted with EtOAc. The combined organic layers were washed with brine, dried over Na_2SO_4, filtered and concentrated under reduced pressure.

To a solution of crude alkene (110 mg, 0.22 mmol, 1.0 equiv) in THF (2.2 mL, 0.1 M) was added dropwise TBAF (1 M in THF, 0.33 mL, 0.33 mmol, 1.5 equiv) at 0 °C. After being stirred for 3 h at room temperature, the reaction mixture was quenched with saturated aqueous $NaHCO_3$ and extracted with EtOAc. The organic layers were combined, washed with brine and dried over sodium sulfate, filtered and evaporated *in vacuo*. The residue was subjected to silica gel column chromatography (PE:EtOAc) to give alcohol **7r**.

Yield: 54% over 2 steps (73 mg), isolated as orange oil, mixture of *E* and *Z* isomers. Purification: Flash chromatography (PE/EtOAc, 70:30), R_f = 0.44 and 0.62 (PE/EtOAc 70:30).

^{1}H NMR (400 MHz, $CDCl_3$) (ratio of the 2 isomers 1 : 0.4) δ 7.91 – 7.85 (m, 1 + 0.4H), 7.61 – 7.53 (m, 1 + 0.4H), 7.42-7.16 (m, 7 + 2.8H), 5.72 – 5.58 (m, 1 + 0.4H), 4.96 – 4.84 (m, 2 + 0.8H), 3.78 – 3.60 (m, 2 + 0.8H), 2.65 – 2.58 (m, 1H), 2.50 – 1.88 (m, 5 + 2.4H), 1.57 – 1.39 (m, 2 + 0.8H).

^{13}C NMR (101 MHz, $CDCl_3$) 2 isomers δ 148.9, 148.8, 140.6, 140.0, 138.4, 138.3, 137.8, 137.7, 137.4, 136.4, 135.9, 133.1, 133.0, 132.7, 132.4, 129.8, 129.7, 128.5, 128.2, 128.1, 127.9, 127.3, 127.2, 124.9, 124.6, 115.0, 114.9, 61.2, 60.8, 35.9, 34.6, 33.6, 33.56, 32.2, 31.0, 27.6, 27.3.

HRMS (ESI/QTOF) m/z: $[M + Na]^+$ Calcd for $C_{21}H_{23}NNaO_3^+$ 360.1571; Found 360.1584.

IR (ν_{max}, cm^{-1}) 3396 (w), 3076 (w), 2924 (w), 2866 (w), 2361 (w), 1524 (s), 1348 (m), 1038 (m), 916 (m), 750 (m), 702 (s).

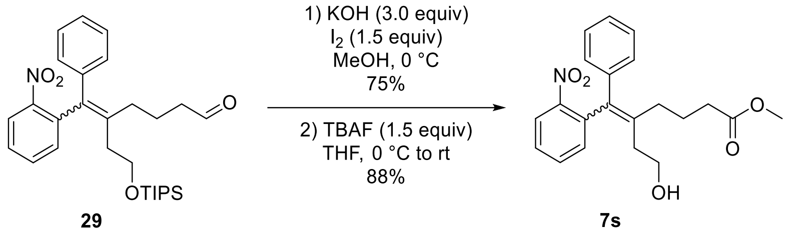

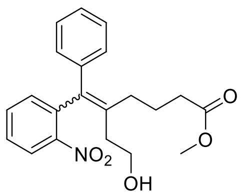

methyl 7-hydroxy-5-((2-nitrophenyl)(phenyl)methylene)heptanoate (**7s**)

To a solution of aldehyde **29** (180 mg, 0.36 mmol, 1.0 equiv) in MeOH (5.4 mL, 0.07 M) was added dropwise a solution of KOH in MeOH (0.2 M, 61 mg, 0.9 mmol, 2.6 equiv) followed by addition of a solution of I_2 in MeOH (0.2 M, 69 mg, 1.3 equiv) at 0 °C. After being stirred for 2 h at room temperature, the reaction mixture was quenched with saturated solution of NH_4Cl and extracted with EtOAc. The organic layers were combined, washed with brine and dried over sodium sulfate, filtered and evaporated *in vacuo*. The residue was subjected to the next step without further purification.

To a solution of crude ester (145 mg, 0.27 mmol, 1.0 equiv) in THF (2.7 mL, 0.1 M) was added dropwise TBAF (1 M in THF, 0.4 mL, 0.4 mmol, 1.5 equiv) at 0 °C. After being stirred for 3 h at room temperature, the reaction mixture was quenched with saturated aqueous $NaHCO_3$ and extracted with EtOAc. The organic layers were combined, washed with brine and dried over sodium sulfate, filtered and evaporated *in vacuo*. The residue was subjected to silica gel column chromatography (PE:EtOAc) to give alcohol **7s**.

Yield: 66% over 2 steps (110 mg), isolated as brown oil, mixture of *E* and *Z* isomers. Purification: Flash chromatography (PE/EtOAc, 65:35), $R_f = 0.15$ (PE/EtOAc 70:30).

^{1}H NMR (400 MHz, $CDCl_3$) (ratio of the 2 isomers 1 : 0.6) δ 7.91 – 7.86 (m, 1 + 0.6H), 7.61 – 7.52 (m, 1 + 0.6H), 7.44 – 7.37 (m, 1 + 1.2H), 7.35 – 7.13 (m, 6 + 3H), 3.78 – 3.64 (m, 3.2H), 3.593, 3.589 (two s, 3 + 1.8H), 2.65 – 2.58 (m, 1H), 2.52 – 1.95 (m, 5 + 3.6H), 1.84 – 1.66 (m, 2 + 1.2H).

^{13}C NMR (101 MHz, $CDCl_3$): δ 173.9, 173.8, 148.8, 148.7, 140.4, 139.9, 137.6, 137.5, 137.1, 136.7, 136.6, 136.5, 133.2, 133.1, 132.6, 132.3, 129.8, 129.7, 128.5, 128.3, 128.2, 128.0, 127.4, 127.3, 124.9, 124.6, 61.1, 60.8, 51.7, 35.6, 34.4, 33.7, 33.6, 32.1, 30.7, 23.4, 23.2.

HRMS (ESI/QTOF) m/z: $[M + Na]^+$ Calcd for $C_{21}H_{23}NNaO_5^+$ 392.1468; Found 392.1477.

IR (ν_{max}, cm^{-1}) 3458 (w), 2960 (w), 2870 (w), 1738 (s), 1525 (s), 1352 (s), 1200 (m), 1045 (m), 908 (m), 743 (s).

3.5) General procedure D for the synthesis of *o*-nitrostylbenes

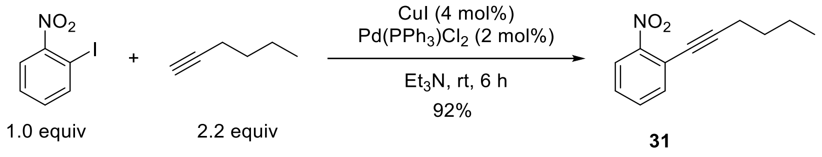

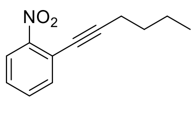

1-(hex-1-yn-1-yl)-2-nitrobenzene (**31**)

To a solution of 1-iodo-2-nitrobenzene (4.25 g, 17.07 mmol, 1.00 equiv) in NEt_3 (85 mL) were added 1-hexyne (3.08 g, 37.5 mmol, 2.20 equiv), CuI (130 mg, 682 µmol, 0.04 equiv) and $Pd(PPh_3)Cl_2$ (240 mg, 341 µmol, 0.02 equiv). The mixture was stirred at 23 °C for 6 h, then concentrated under reduced pressure. The residue was purified by FCC (30:1 → 20:1 PE:EtOAc) to afford 1-(hex-1-yn-1-yl)-2-nitrobenzene **31** as a yellow oil that solidified in the freezer (3.21 g 15.8 mmol, 92%).

^{1}H NMR (600 MHz, $CDCl_3$) δ 7.96 (dd, *J* = 8.3, 1.3 Hz, 1H), 7.57 (dd, *J* = 7.8, 1.6 Hz, 1H), 7.51 (td, *J* = 7.5, 1.3 Hz, 1H), 7.38 (ddd, *J* = 8.2, 7.3, 1.6 Hz, 1H), 2.48 (t, *J* = 7.0 Hz, 2H), 1.67 – 1.57 (m, 2H), 1.56 – 1.44 (m, 2H), 0.95 (t, *J* = 7.3 Hz, 3H).

^{13}C NMR (151 MHz, $CDCl_3$) δ 150.2, 134.9, 132.6, 127.9, 124.5, 119.5, 99.5, 76.0, 30.5, 22.1, 19.6, 13.7.

HRMS (nanochip-ESI/LTQ-Orbitrap) m/z: $[M + Na]^+$ Calcd for $C_{12}H_{13}NNaO_2^+$ 226.0838; Found 226.0832.

IR (vmax, cm-1) 2156 (s), 1487 (s), 1454 (s), 1348 (s), 1522 (s), 2956 (s), 3512 (s), 750 (s), 700 (s), 1038 (s), 1178 (s).

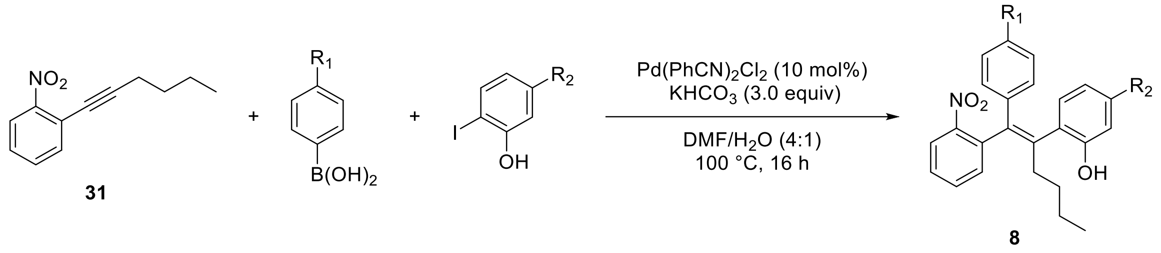

To a 100 mL flask were added 1-(hex-1-yn-1-yl)-2-nitrobenzene **31** (500 mg, 2.46 mmol, 1.0 equiv), arylboronic acid (7.38 mmol, 3.0 equiv), 2-iodophenol (4.92 mmol, 2.0 equiv), $KHCO_3$ (739 mg, 7.38 mmol, 3.0 equiv), DMF (40 mL) and water (10 mL), and the reaction mixture was degassed and placed under an argon atmosphere. A solution of $Pd(PhCN)_2Cl_2$ (95 mg, 246 µmol, 0.1 equiv) in DMF (1 mL) was added in one portion. The mixture was heated at 100 °C for 16 h. After cooling to room temperature, the mixture was poured into cold water (300 mL) and extracted with Et_2O (3 x 100 mL). The combined organic layers were washed with brine, dried over sodium sulfate and concentrated under reduced pressure. Purification by FCC (PE:EtOAc) afforded the desired products **8** in 8-35% yields.

*Note: due to steric hindrance from the ortho substituents, rotamers are observed in the ^{1}H and ^{13}C NMR spectra of all o-*nitrostilbenes.

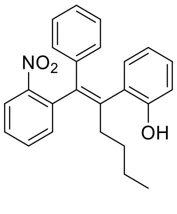

(*E*)-2-(1-(2-nitrophenyl)-1-phenylhex-1-en-2-yl)phenol (**8a**)

This compound was prepared following the general procedure **D**. Yield: 35% (321 mg, 0.86 mmol), isolated as yellow solid. Purification: Flash chromatography (PE/EtOAc, 10:1).

^{1}H NMR (600 MHz, $CDCl_3$) δ 8.10 (d, *J* = 8.3 Hz, 1H), 7.60 (t, *J* = 7.6 Hz, 1H), 7.49 (t, *J* = 7.9 Hz, 1H), 7.27 (d, *J* = 7.5 Hz, 1H), 7.19 (d, *J* = 7.5 Hz, 1H), 7.15 – 7.02 (m, 6H), 6.90 (t, *J* = 7.4 Hz, 1H), 6.73 (d, *J* = 8.2 Hz, 1H), 6.06 (s, 1H), 2.31 – 2.20 (m, 1H), 2.12 – 2.01 (m, 1H), 1.25 – 1.04 (m, 4H), 0.71 (t, *J* = 7.0 Hz, 3H).

^{13}C NMR (151 MHz, $CDCl_3$) δ 153.0, 148.5, 139.5, 137.8, 137.0, 136.6, 133.9, 132.5, 129.8, 129.0, 128.9, 128.4, 128.0, 127.6, 126.5, 124.9, 120.0, 116.0, 36.7, 29.5, 23.0, 13.8.

HRMS (ESI/QTOF) m/z: [M + H]$^+$ Calcd for $C_{24}H_{24}NO_3^+$ 374.1751; Found 374.1743.

IR (vmax, cm-1) 2156 (s), 1487 (s), 1454 (s), 1348 (s), 1522 (s), 2956 (s), 3512 (s), 750 (s), 700 (s), 1038 (s), 1178 (s)

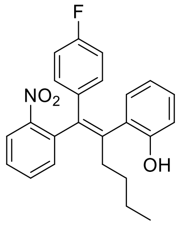

(E)-2-(1-(4-fluorophenyl)-1-(2-nitrophenyl)hex-1-en-2-yl)phenol (**8b**)

This compound was prepared following the general procedure **D**. Yield: 17% (233 mg, 0.54 mmol), isolated as yellow solid. Purification: Flash chromatography (PE/EtOAc, 10:1).

^{1}H NMR (400 MHz, $CDCl_3$) δ 8.12 – 8.04 (m, 1H), 7.65 – 7.58 (m, 1H), 7.55 – 7.47 (m, 1H), 7.25 – 7.21 (m, 1H), 7.18 – 7.05 (m, 4H), 6.89 (t, *J* = 7.4 Hz, 1H), 6.80 – 6.71 (m, 3H), 5.98 (s, 1H), 2.31-2.17 (m, 1H), 2.09-1.98 (m, 1H), 1.24 – 1.00 (m, 4H), 0.71 (t, *J* = 6.9 Hz, 3H).

^{13}C NMR (101 MHz, $CDCl_3$) δ 161.9 (d, *J* = 248.1 Hz), 152.9, 148.6, 137.5, 137.3, 135.6, 135.5 (d, *J* = 3.4 Hz), 134.0, 132.4, 131.6 (d, *J* = 8.1 Hz), 129.2, 128.8, 128.6, 126.3, 124.9, 120.1, 116.1, 115.1 (d, *J* = 21.5 Hz), 36.6, 29.6, 23.0, 13.8.

HRMS (Sicrit plasma/LTQ-Orbitrap) m/z: [M + H]$^+$ Calcd for $C_{24}H_{23}FNO_3^+$ 392.1656; Found 392.1654.

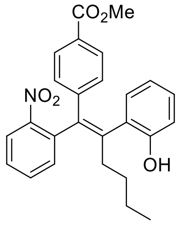

methyl (*E*)-4-(2-(2-hydroxyphenyl)-1-(2-nitrophenyl)hex-1-en-1-yl)benzoate (**8c**)

This compound was prepared following the general procedure **D**. Yield: 22% (233 mg, 0.54 mmol), isolated as yellow solid. Purification: Flash chromatography (PE/EtOAc, 10:1).

^{1}H NMR (400 MHz, $CDCl_3$) δ 8.12 (d, *J* = 8.3 Hz, 1H), 7.73 (d, *J* = 8.1 Hz, 2H), 7.62 (t, *J* = 7.3 Hz, 1H), 7.54 – 7.49 (m, 1H), 7.25 – 7.20 (m, 1H), 7.19 – 7.05 (m, 4H), 6.89 (td, *J* = 7.4, 1.2 Hz, 1H), 6.71 (d, *J* = 8.1 Hz, 1H), 5.96 (s, 1H), 3.82 (s, 3H), 2.32-2.20 (m, 1H), 2.12-2.02 (m, 1H), 1.22-1.03 (m, 4H), 0.71 (t, *J* = 6.9 Hz, 3H).

^{13}C NMR (101 MHz, $CDCl_3$) δ 166.8, 152.9, 148.6, 144.2, 138.8, 137.0, 135.8, 134.0, 132.4, 129.8, 129.4, 129.3, 128.9, 128.8, 126.0, 125.1, 120.2, 116.2, 52.1, 36.6, 29.5, 22.9, 13.8.

HRMS (ESI/QTOF) m/z: [M + Na]$^+$ Calcd for $C_{26}H_{25}NNaO_5^+$ 454.1625; Found 454.1614.

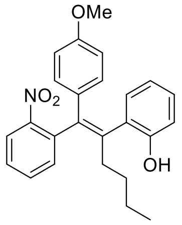

(*E*)-2-(1-(4-methoxyphenyl)-1-(2-nitrophenyl)hex-1-en-2-yl)phenol (**8d**)

This compound was prepared following the general procedure **3.5**. Yield: 10% (99 mg, 0.245 mmol), isolated as yellow solid. Purification: Flash chromatography (PE/EtOAc, 10:1).

^{1}H NMR (600 MHz, $CDCl_3$) δ 8.09 (d, J = 8.2 Hz, 1H), 7.60 (t, J = 7.5 Hz, 1H), 7.48 (t, J = 7.8 Hz, 1H), 7.39 (d, J = 8.6 Hz, 1H), 7.18 (dd, J = 7.6, 1.7 Hz, 1H), 7.14 – 7.11 (m, 1H), 7.01 (d, J = 8.8 Hz, 2H), 6.90 (t, J = 7.4 Hz, 1H), 6.75 (d, J = 8.1 Hz, 1H), 6.60 (d, J = 8.6 Hz, 2H), 6.07 (s, 1H), 3.69 (s, 3H), 2.27-2.18 (m, 1H), 2.04-1.97 (m, 1H), 1.19 – 1.00 (m, 4H), 0.70 (t, J = 7.1 Hz, 3H).

^{13}C NMR (151 MHz, $CDCl_3$) δ 158.8, 153.0, 148.6, 138.2, 136.0, 135.7, 133.9, 132.6, 131.1, 130.4, 128.9, 128.8, 128.3, 126.7, 124.8, 120.1, 114.9, 113.4, 55.2, 36.8, 29.6, 23.0, 13.8.

HRMS (ESI/QTOF) m/z: $[M + Na]^+$ Calcd for $C_{25}H_{25}NNaO_4^+$ 426.1676; Found 426.1661.

3.6) General procedure E for the reductive cyclization of *o*-nitrostyrenes

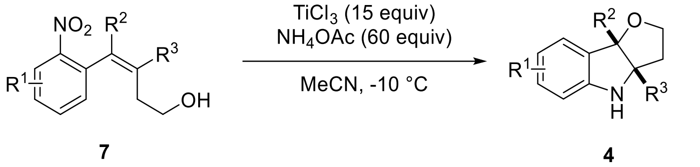

Glassware and stirring bar were stored in the oven and the reaction was carried out under inert atmosphere and dry conditions. MeCN and the solution of $TiCl_3$ were degassed with Freeze-pump-thaw technique. In a 10 mL pressure resistant round bottomed flask was charged the nitrostyrene **7** (1.0 equiv) and NH_4OAc (60 equiv) in MeCN (0.05 M). Then $TiCl_3$ (1.3 M solution in HCl, 15 equiv) was added dropwise at -10 °C upon 10 min. The reaction mixture was stirred at -10 °C for 3 h. After completion of the reaction, $NaHCO_3$ was added slowly at -10 °C to quench the reaction. The mixture was extracted with EtOAc. The combined organic layers were washed with brine, dried, filtered and concentrated under reduced pressure. The residue was subjected to silica gel column chromatography (PE:EtOAc) to give the desired furo[3,2-*b*]indoline **4**.

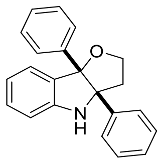

3a,8b-diphenyl-3,3a,4,8b-tetrahydro-2*H*-furo[3,2-b]indole (**4a**)

This compound was prepared following the general procedure **E** using substrate **7a** (34.5 mg, 0.1 mmol) as starting material. Yield: 59% (18.5 mg), isolated as yellow solid. Purification: Flash chromatography (PE/EtOAc, 98:2), R_f = 0.38 (PE/EtOAc 90:10).

^{1}H NMR (400 MHz, $CDCl_3$) δ 7.24 (t, J = 7.6 Hz, 1H), 7.10 (d, J = 7.3 Hz, 2H), 7.04 – 6.97 (m, 8H), 6.96 (d, J = 7.4 Hz, 1H), 6.82 (d, J = 8.0 Hz, 1H), 6.77 (t, J = 7.4 Hz, 1H), 4.45 (t, J = 8.1 Hz, 1H), 4.28 (br, 1H), 4.07 (ddd, J = 11.1, 8.5, 5.2 Hz, 1H), 2.92 (td, J = 11.9, 7.6 Hz, 1H), 2.28 (dd, J = 12.8, 5.1 Hz, 1H).

^{13}C NMR (101 MHz, $CDCl_3$): δ 151.6, 142.1, 140.9, 130.5, 130.0, 127.8, 127.6, 127.1, 126.83, 126.80, 126.76, 126.5, 119.4, 108.6, 98.5, 80.4, 67.3, 41.5.

HRMS (nanochip-ESI/LTQ-Orbitrap) m/z: $[M + H]^+$ Calcd for $C_{22}H_{20}NO^+$ 314.1539; Found 314.1530.

IR (ν_{max}, cm^{-1}) 3352 (w), 3016 (w), 2830 (w), 1614 (m), 1507 (m), 1382 (m), 1259 (m), 1031 (m), 753 (s).

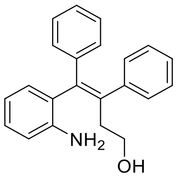

(E)-4-(2-aminophenyl)-3,4-diphenylbut-3-en-1-ol (**32**)

Glassware and stirring bar were stored in the oven and the reaction was carried out under inert atmosphere and dry conditions. MeCN and the solution of $TiCl_3$ were degassed with Freeze-pump-thaw technique. In a 10 mL pressure resistant round bottomed flask was charged the nitrostyrene **7a** (34.5 mg, 0.1 mmol, 1.0 equiv) and NBu_4OAc (60 equiv) in MeCN (0.05 M). Then $TiCl_3$ (1.3 M solution in HCl, 15 equiv) was added dropwise at -10 °C upon 10 min. The reaction mixture was stirred at -10 °C for 3 h. After completion of the reaction, $NaHCO_3$ was added slowly at -10 °C to quench the reaction. The mixture was extracted with EtOAc. The combined organic layers were washed with brine, dried, filtered and concentrated under reduced pressure. Yield: 98% (NMR yield), isolated as yellow oil. Purification: Flash chromatography (PE/EtOAc, 80:20), R_f = 0.22 (PE/EtOAc 80:20).

^{1}H NMR (400 MHz, $CDCl_3$) δ 7.24 – 7.11 (m, 7H), 7.06 – 6.98 (m, 3H), 6.98 – 6.92 (m, 2H), 6.84 (td, J = 7.4, 1.2 Hz, 1H), 6.78 (dd, J = 8.0, 1.2 Hz, 1H), 3.60 – 3.48 (m, 2H), 3.38 (br, 2H), 2.77 – 2.62 (m, 2H).

^{13}C NMR (101 MHz, $CDCl_3$): δ 143.2, 141.2, 141.1, 140.1, 137.6, 130.7, 130.1, 129.7, 129.5, 128.4, 128.3, 127.8, 126.9, 126.4, 119.5, 116.5, 60.4, 39.3.

HRMS (ESI/QTOF) m/z: $[M + H]^+$ Calcd for $C_{22}H_{22}NO^+$ 316.1696; Found 316.1691.

IR (ν_{max}, cm^{-1}) 3345 (w), 2959 (m), 2832 (m), 1604 (s), 1512 (s), 1475 (s), 1216 (m), 1054 (m), 810 (m), 746 (s).

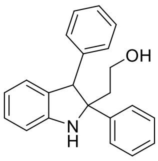

2-(2,3-diphenylindolin-2-yl)ethan-1-ol (**14**)

Glassware and stirring bar were stored in the oven and the reaction was carried out under inert atmosphere and dry conditions. MeCN and the solution of $TiCl_3$ were degassed with Freeze-pump-thaw technique. In a 10 mL pressure resistant round bottomed flask was charged the nitrostyrene **7a** (34.5 mg, 0.1 mmol, 1.0 equiv) and NH_4OAc (60 equiv) in MeCN (0.1 M). Then $TiCl_3$ (1.3 M solution in HCl, 10 equiv) was added dropwise at rt upon 10 min. The reaction mixture was stirred at rt for 3 h. After completion of the reaction, $NaHCO_3$ was added slowly at rt to quench the reaction. The mixture was extracted with EtOAc. The combined organic layers were washed with brine, dried, filtered and concentrated under reduced pressure. Yield: 30% (NMR yield), isolated as yellow oil. Purification: Flash chromatography (PE/EtOAc, 80:20), R_f = 0.27 (PE/EtOAc 70:30).

^{1}H NMR (400 MHz, $CDCl_3$) δ 7.15 (t, J = 7.6 Hz, 1H), 7.03 – 6.95 (m, 8H), 6.87 (d, J = 7.5 Hz, 1H), 6.84 (d, J = 7.5 Hz, 1H), 6.75 – 6.70 (m, 3H), 4.57 (s, 1H), 3.91 (ddd, J = 11.1, 5.7, 4.8 Hz, 1H), 3.67 (ddd, J = 11.1, 8.3, 4.4 Hz, 1H), 2.54 (ddd, J = 14.4, 8.3, 4.8 Hz, 1H), 2.37 (ddd, J = 14.5, 5.7, 4.5 Hz, 1H).

^{13}C NMR (101 MHz, $CDCl_3$): δ 150.4, 142.2, 139.8, 131.6, 129.7, 128.3, 127.73, 127.70, 127.1, 126.6, 126.4, 125.9, 119.4, 109.5, 74.7, 60.8, 60.4, 42.2.

HRMS (ESI/QTOF) m/z: $[M + H]^+$ Calcd for $C_{22}H_{22}NO^+$ 316.1696; Found 316.1699.

IR (ν_{max}, cm^{-1}) 3354 (w), 2999 (m), 2916 (m), 1617 (m), 1460 (m), 1410 (m), 1278 (s), 1057 (s), 754 (s).

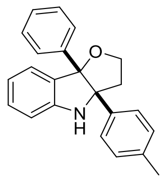

8b-phenyl-3a-(p-tolyl)-3,3a,4,8b-tetrahydro-2*H*-furo[3,2-b]indole (**4b**)

This compound was prepared following the general procedure **E** using substrate **7b** (36.0 mg, 0.1 mmol) as starting material. Yield: 42% (14 mg), isolated as orange oil. Purification: Flash chromatography (PE/EtOAc, 98:2), R_f = 0.41 (PE/EtOAc 90:10).

^{1}H NMR (400 MHz, $CDCl_3$) δ 7.23 (td, J = 7.6, 1.4 Hz, 1H), 7.00 – 6.94 (m, 8H), 6.83 – 6.79 (m, 3H), 6.76 (td, J = 7.4, 1.0 Hz, 1H), 4.43 (ddd, J = 8.7, 7.7, 1.2 Hz, 1H), 4.27 (br, 1H), 4.06 (ddd, J = 11.1, 8.3, 5.1 Hz, 1H), 2.89 (ddd, J = 12.5, 11.1, 7.6 Hz, 1H), 2.25 (ddd, J = 12.6, 5.1, 1.3 Hz, 1H), 2.16 (s, 3H).

^{13}C NMR (101 MHz, $CDCl_3$): δ 151.6, 140.9, 138.9, 136.4, 130.6, 130.0, 128.5, 127.6, 127.0, 126.8, 126.7, 126.4, 119.2, 108.6, 98.2, 80.4, 67.3, 41.3, 20.9.

HRMS (ESI/QTOF) m/z: $[M + H]^+$ Calcd for $C_{23}H_{22}NO^+$ 328.1696; Found 328.1696.

IR (ν_{max}, cm^{-1}) 3377 (w), 3033 (w), 2922 (w), 2864 (w), 1607 (m), 1482 (m), 1468 (m), 1152 (m), 1047 (m), 742 (s).

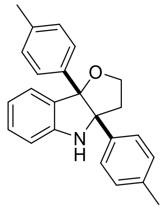

3a,8b-di-p-tolyl-3,3a,4,8b-tetrahydro-2*H*-furo[3,2-b]indole (**4c**)

This compound was prepared following the general procedure **E** using substrate **7c** (43.8 mg, 0.1 mmol) as starting material. Yield: 54% (18.4 mg), isolated as yellow oil. Purification: Flash chromatography (PE/EtOAc, 98:2), R_f = 0.34 (PE/EtOAc 90:10).

^{1}H NMR (400 MHz, $CDCl_3$) δ 7.22 (td, J = 7.6, 1.3 Hz, 1H), 6.97 (d, J = 8.3 Hz, 2H), 6.96 – 6.93 (m, 1H), 6.88 (d, J = 8.3 Hz, 2H), 6.85 – 6.78 (m, 5H), 6.75 (td, J = 7.4, 1.0 Hz, 1H), 4.41 (ddd, J = 8.6, 7.7, 1.2 Hz, 1H), 4.26 (br, 1H), 4.05 (ddd, J = 11.2, 8.3, 5.1 Hz, 1H), 2.87 (ddd, J = 12.5, 11.2, 7.6 Hz, 1H), 2.24 (ddd, J = 12.6, 5.1, 1.2 Hz, 1H), 2.18 (s, 6H).

^{13}C NMR (101 MHz, $CDCl_3$): δ 151.6, 139.1, 137.9, 136.4, 136.2, 130.9, 129.9, 128.5, 127.8, 127.5, 126.8, 126.4, 119.2, 108.5, 98.1, 80.3, 67.2, 41.3, 21.1, 21.0.

HRMS HRMS (ESI/QTOF) m/z: $[M + H]^+$ Calcd for $C_{24}H_{24}NO^+$ 342.1852; Found 342.1856.

IR (ν_{max}, cm^{-1}) 3368 (w), 3032 (w), 2863 (w), 1604 (m), 1511 (m), 1482 (m), 1467 (m), 1323 (w), 1215 (w), 1046 (m), 804 (m), 746 (s).

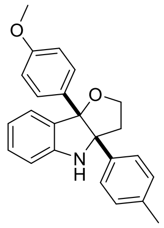

8b-(4-methoxyphenyl)-3a-(p-tolyl)-3,3a,4,8b-tetrahydro-2*H*-furo[3,2-b]indole (**4d**)

This compound was prepared following the general procedure **E** using substrate **7d** (46.4 mg, 0.1 mmol) as starting material. Yield: 52% (18.6 mg), isolated as yellow oil. Purification: Flash chromatography (PE/EtOAc, 95:5), R_f = 0.23 (PE/EtOAc 90:10).

^{1}H NMR (400 MHz, $CDCl_3$) δ 7.22 (td, J = 7.7, 1.3 Hz, 1H), 6.99 – 6.93 (m, 3H), 6.91 (d, J = 8.7 Hz, 2H), 6.84 (d, J = 8.1 Hz, 2H), 6.79 (d, J = 7.9 Hz, 1H), 6.75 (td, J = 7.4, 1.0 Hz, 1H), 6.54 (d, J = 9.0 Hz, 2H), 4.43 – 4.37 (m, 1H), 4.25 (brs, 1H), 4.04 (ddd, J = 11.2, 8.3, 5.1 Hz, 1H), 3.69 (s, 3H), 2.87 (ddd, J = 12.5, 11.2, 7.6 Hz, 1H), 2.24 (ddd, J = 12.6, 5.1, 1.2 Hz, 1H), 2.18 (s, 3H).

^{13}C NMR (101 MHz, $CDCl_3$): δ 158.4, 151.7, 139.0, 136.4, 133.2, 130.8, 130.0, 128.8, 128.6, 126.8, 126.4, 119.2, 112.5, 108.5, 98.0, 80.2, 67.2, 55.3, 41.1, 21.0.

HRMS (ESI/QTOF) m/z: $[M + H]^+$ Calcd for $C_{24}H_{24}NO_2^+$ 358.1802; Found 358.1803.

IR (ν_{max}, cm^{-1}) 3411 (w), 3025 (w), 2841 (w), 1604 (m), 1521 (m), 1507 (m), 1352 (m), 1251 (m), 1179 (m), 1031 (m), 829 (m), 753 (s).

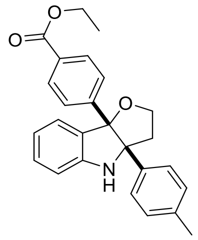

ethyl 4-(3a-(p-tolyl)-2,3,3a,4-tetrahydro-8b*H*-furo[3,2-b]indol-8b-yl)benzoate (**4e**)

This compound was prepared following the general procedure **E** using substrate **7e** (43.2 mg, 0.1 mmol) as starting material. Yield: 56% (22.3 mg), isolated as orange oil. Purification: Flash chromatography (PE/EtOAc, 95:5), R_f = 0.25 (PE/EtOAc 90:10).

^{1}H NMR (400 MHz, $CDCl_3$) δ 7.67 (d, J = 8.8 Hz, 2H), 7.23 (td, J = 7.6, 1.4 Hz, 1H), 7.08 (d, J = 8.1 Hz, 2H), 6.98 (d, J = 8.3 Hz, 2H), 6.88 (dd, J = 7.6, 1.3 Hz, 1H), 6.84 – 6.81 (m, 3H), 6.75 (td, J = 7.4, 1.0 Hz, 1H), 4.44 (td, J = 8.1, 1.3 Hz, 1H), 4.35 – 4.22 (m, 3H), 4.04 (ddd, J = 11.1, 8.4, 5.2 Hz, 1H), 2.88 (ddd, J = 12.7, 11.1, 7.7 Hz, 1H), 2.28 (ddd, J = 12.7, 5.3, 1.3 Hz, 1H), 2.16 (s, 3H), 1.34 (t, J = 7.1 Hz, 3H).

^{13}C NMR (101 MHz, $CDCl_3$): δ 166.8, 151.5, 146.3, 138.6, 136.7, 130.2, 130.2, 128.8, 128.7, 128.4, 127.6, 126.6, 126.3, 119.4, 108.8, 98.0, 80.5, 67.3, 60.9, 42.0, 21.0, 14.4.

HRMS (ESI/QTOF) m/z: $[M + H]^+$ Calcd for $C_{26}H_{26}NO_3^+$ 400.1907; Found 400.1908.

IR (ν_{max}, cm^{-1}) 3370 (w), 2990 (m), 2904 (m), 1712 (m), 1607 (m), 1485 (m), 1468 (m), 1407 (m), 1278 (s), 1105 (s), 1047 (s), 1022 (s), 754 (s).

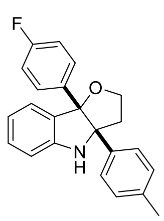

8b-(4-fluorophenyl)-3a-(p-tolyl)-3,3a,4,8b-tetrahydro-2*H*-furo[3,2-b]indole (**4f**)

This compound was prepared following the general procedure **E** using substrate **7f** (40 mg, 0.106 mmol) as starting material. Yield: 66% (24.3 mg), isolated as brown solid. Purification: Flash chromatography (PE/EtOAc, 95:5), R_f = 0.4 (PE/EtOAc 90:10).

^{1}H NMR (400 MHz, $CDCl_3$) δ 7.23 (td, J = 7.7, 1.4 Hz, 1H), 6.99 – 6.94 (m, 4H), 6.93 (dd, J = 7.8, 1.2 Hz, 1H), 6.85 (d, J = 8.1 Hz, 2H), 6.80 (d, J = 7.9 Hz, 1H), 6.79 – 6.74 (m, 1H), 6.68 (t, J = 8.9 Hz, 2H),

4.41 (ddd, J = 8.7, 7.6, 1.3 Hz, 1H), 4.26 (br, 1H), 4.03 (ddd, J = 11.2, 8.4, 5.1 Hz, 1H), 2.87 (ddd, J = 12.7, 11.2, 7.7 Hz, 1H), 2.25 (ddd, J = 12.6, 5.1, 1.3 Hz, 1H), 2.18 (s, 3H).

^{13}C NMR (101 MHz, $CDCl_3$): δ 161.8 (d, J = 244.9 Hz), 151.6, 138.8, 136.9 (d, J = 3.0 Hz), 136.6, 130.3, 130.2, 129.3 (d, J = 8.1 Hz), 128.7, 126.7, 126.3, 119.3, 113.9 (d, J = 21.3 Hz), 108.7, 97.9, 80.2, 67.3, 41.3, 21.0.

^{19}F NMR (377 MHz, $CDCl_3$) δ -116.5.

HRMS (ESI/QTOF) m/z: [M + H]$^+$ Calcd for $C_{23}H_{21}FNO^+$ 346.1602; Found 346.1604.

IR (ν_{max}, cm^{-1}) 3370 (w), 2975 (m), 2882 (m), 1604 (s), 1507 (s), 1482 (s), 1468 (s), 1220 (m), 1044 (m), 807 (m), 746 (s).

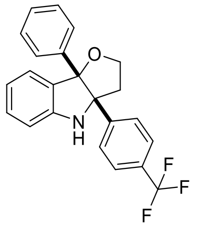

8b-phenyl-3a-(4-(trifluoromethyl)phenyl)-3,3a,4,8b-tetrahydro-2*H*-furo[3,2-b]indole (**4g**)

This compound was prepared following the general procedure **E** using substrate **7g** (41 mg, 0.1 mmol) as starting material. Yield: 58% (22 mg), isolated as brown solid. Purification: Flash chromatography (PE/EtOAc, 95:5), R_f = 0.38 (PE/EtOAc 90:10).

^{1}H NMR (400 MHz, $CDCl_3$) δ 7.27 – 7.23 (m, 5H), 7.01 – 6.95 (m, 6H), 6.84 (dt, J = 7.9, 0.8 Hz, 1H), 6.80 (td, J = 7.4, 0.9 Hz, 1H), 4.46 (ddd, J = 9.1, 7.8, 1.4 Hz, 1H), 4.03 (ddd, J = 11.0, 8.6, 5.4 Hz, 1H), 2.89 (ddd, J = 12.7, 11.0, 7.7 Hz, 1H), 2.32 (ddd, J = 12.8, 5.4, 1.4 Hz, 1H).

^{13}C NMR (101 MHz, $CDCl_3$): δ 151.0, 146.7, 140.5, 130.2, 130.1, 128.9 (q, J = 32.4 Hz), 127.3, 127.1, 127.0, 126.7, 124.5 (q, J = 3.9 Hz), 124.2 (q, J = 271.9 Hz), 119.8, 108.9, 98.9, 79.9, 67.1, 43.0.

^{19}F NMR (377 MHz, $CDCl_3$) δ -62.6.

HRMS (ESI/QTOF) m/z: [M + H]$^+$ Calcd for $C_{23}H_{19}F_3NO^+$ 382.1413; Found 382.1415.

IR (ν_{max}, cm^{-1}) 3359 (w), 3054 (w), 2954 (w), 2875 (w), 2358 (w), 1607 (m), 1485 (m), 1471 (m), 1327 (s), 1162 (s), 1116 (s), 1069 (s), 1015 (s), 746 (s).

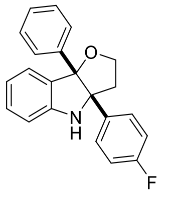

3a-(4-fluorophenyl)-8b-phenyl-3,3a,4,8b-tetrahydro-2*H*-furo[3,2-b]indole (**4h**)

This compound was prepared following the general procedure **E** using substrate **7h** (40 mg, 0.11 mmol) as starting material. Yield: 64% (23.3 mg), isolated as brown solid. Purification: Flash chromatography (PE/EtOAc, 95:5), R_f = 0.38 (PE/EtOAc 90:10).

^{1}H NMR (400 MHz, $CDCl_3$) δ 7.24 (td, J = 7.4, 1.3 Hz, 1H), 7.10 – 7.05 (m, 2H), 7.02 – 6.96 (m, 6H), 6.82 – 6.76 (m, 2H), 6.70 (t, J = 8.7 Hz, 2H), 4.43 (td, J = 8.1, 1.3 Hz, 1H), 4.23 (br, 1H), 4.03 (ddd, J = 11.1, 8.5, 5.2 Hz, 1H), 2.86 (ddd, J = 12.7, 11.1, 7.7 Hz, 1H), 2.28 (ddd, J = 12.7, 5.2, 1.3 Hz, 1H).

^{13}C NMR (101 MHz, $CDCl_3$): δ 161.64 (d, J = 245.8 Hz), 151.3, 140.8, 137.98 (d, J = 3.3 Hz), 130.2, 130.1, 128.16 (d, J = 8.0 Hz), 127.5, 127.2, 126.9, 126.8, 119.5, 114.44 (d, J = 21.3 Hz), 108.7, 98.4, 79.8, 67.1, 42.1.

^{19}F NMR (377 MHz, $CDCl_3$) δ -116.3.

HRMS (ESI/QTOF) m/z: $[M + H]^+$ Calcd for $C_{22}H_{19}FNO^+$ 332.1445; Found 332.1453

IR (ν_{max}, cm^{-1}) 3359 (w), 3061 (w), 2943 (w), 2864 (w), 1607 (m), 1507 (s), 1482 (m), 1468 (m), 1227 (m), 1044 (m), 746 (s).

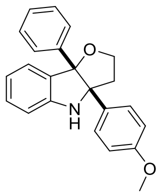

3a-(4-methoxyphenyl)-8b-phenyl-3,3a,4,8b-tetrahydro-2*H*-furo[3,2-b]indole (**4i**)

This compound was prepared following the general procedure **E** using substrate **7i** (37.5 mg, 0.1 mmol) as starting material. Yield: 27% (9.2 mg), isolated as yellow oil. Purification: Flash chromatography (PE/EtOAc, 98:2), R_f = 0.31 (PE/EtOAc 90:10).

^{1}H NMR (400 MHz, $CDCl_3$) δ 7.23 (td, *J* = 7.6, 1.4 Hz, 1H), 7.03 – 6.97 (m, 7H), 6.96 (dd, *J* = 7.5, 1.3 Hz, 1H), 6.80 (d, *J* = 7.9 Hz, 1H), 6.76 (td, *J* = 7.4, 1.0 Hz, 1H), 6.55 (d, *J* = 8.9 Hz, 2H), 4.42 (ddd, *J* = 8.6, 7.7, 1.2 Hz, 1H), 4.28 (s, 1H), 4.06 (ddd, *J* = 11.2, 8.4, 5.1 Hz, 1H), 3.67 (s, 3H), 2.86 (ddd, *J* = 12.6, 11.2, 7.6 Hz, 1H), 2.25 (ddd, *J* = 12.6, 5.1, 1.3 Hz, 1H).

^{13}C NMR (101 MHz, $CDCl_3$): δ 158.4, 151.7, 140.9, 134.0, 130.6, 130.0, 127.64, 127.60, 127.1, 126.84, 126.80, 119.3, 113.1, 108.6, 98.2, 80.2, 67.3, 55.3, 41.1.

HRMS (ESI/QTOF) m/z: $[M + H]^+$ Calcd for $C_{23}H_{22}NO_2^+$ 344.1645; Found 344.1646.

IR (ν_{max}, cm^{-1}) 3374 (w), 3044 (w), 2932 (w), 2868 (w), 2831 (w), 2358 (w), 1607 (s), 1511 (s), 1482 (m), 1468 (m), 1320 (m), 1299 (m), 1252 (s), 1180 (s), 1033 (s), 746 (s).

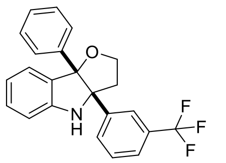

8b-phenyl-3a-(3-(trifluoromethyl)phenyl)-3,3a,4,8b-tetrahydro-2*H*-furo[3,2-b]indole (**4j**)

This compound was prepared following the general procedure **E** using substrate **7j** (42 mg, 0.1 mmol) as starting material. Yield: 53% (20.6 mg), isolated as yellow oil. Purification: Flash chromatography (PE/EtOAc, 95:5), R_f = 0.37 (PE/EtOAc 90:10).

^{1}H NMR (400 MHz, $CDCl_3$) δ 7.38 (s, 1H), 7.35 (d, *J* = 8.0 Hz, 1H), 7.28 – 7.23 (m, 2H), 7.13 (t, *J* = 7.8 Hz, 1H), 7.00 – 6.89 (m, 6H), 6.84 (d, *J* = 7.9 Hz, 1H), 6.80 (td, *J* = 7.4, 0.9 Hz, 1H), 4.46 (ddd, *J* = 8.8, 7.7, 1.3 Hz, 1H), 4.22 (br, 1H), 4.00 (ddd, *J* = 11.0, 8.7, 5.4 Hz, 1H), 2.88 (ddd, *J* = 12.9, 11.0, 7.7 Hz, 1H), 2.34 (ddd, *J* = 12.9, 5.4, 1.4 Hz, 1H).

^{13}C NMR (101 MHz, $CDCl_3$): δ 150.9, 143.8, 140.6, 130.2, 129.9 (q, *J* = 32.0 Hz), 129.9, 129.88 (br), 128.0, 127.3, 127.2, 127.0, 126.7, 124.1 (q, *J* = 270.7 Hz), 123.6 (q, *J* = 3.9 Hz), 123.4 (q, *J* = 3.8 Hz), 119.8, 108.9, 99.0, 79.6, 66.8, 43.5.

^{19}F NMR (377 MHz, $CDCl_3$) δ -62.8.

HRMS (ESI/QTOF) m/z: $[M + H]^+$ Calcd for $C_{23}H_{19}F_3NO^+$ 382.1413; Found 382.1413.

IR (ν_{max}, cm^{-1}) 3375 (w), 3039 (w), 2960 (w), 2874 (w), 1720 (w), 1604 (m), 1482 (m), 1334 (m), 1164 (m), 1121 (m), 1078 (m), 746 (s).

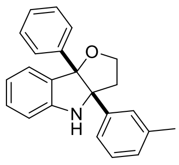

8b-phenyl-3a-(m-tolyl)-3,3a,4,8b-tetrahydro-2*H*-furo[3,2-b]indole (**4k**)

This compound was prepared following the general procedure **E** using substrate **7k** (40 mg, 0.11 mmol) as starting material. Yield: 61% (22.0 mg), isolated as orange oil. Purification: Flash chromatography (PE/EtOAc, 95:5), R_f = 0.5 (PE/EtOAc 90:10).

^{1}H NMR (400 MHz, $CDCl_3$) δ 7.24 (td, J = 7.7, 1.3 Hz, 1H), 7.00 (apparent s, 5H), 6.97 (dd, J = 7.5, 1.3 Hz, 1H), 6.94 – 6.90 (m, 2H), 6.86 (brs, 1H), 6.83 – 6.79 (m, 2H), 6.77 (td, J = 7.4, 1.0 Hz, 1H), 4.44 (ddd, J = 8.6, 7.6, 1.2 Hz, 1H), 4.27 (br, 1H), 4.05 (ddd, J = 11.1, 8.4, 5.1 Hz, 1H), 2.90 (ddd, J = 12.6, 11.2, 7.6 Hz, 1H), 2.27 (ddd, J = 12.6, 5.1, 1.3 Hz, 1H), 2.12 (s, 3H).

^{13}C NMR (101 MHz, $CDCl_3$): δ 151.6, 142.0, 140.9, 137.2, 130.4, 130.0, 127.62, 127.57, 127.42, 127.37, 126.9, 126.8, 126.7, 123.5, 119.2, 108.5, 98.5, 80.3, 67.2, 41.7, 21.5.

HRMS (ESI/QTOF) m/z: $[M + H]^+$ Calcd for $C_{23}H_{22}NO^+$ 328.1696; Found 328.1699.

IR (**ν_{max}**, cm^{-1}) 3367 (w), 3029 (w), 2950 (w), 2878 (w), 1607 (m), 1482 (m), 1468 (m), 1317 (w), 1267 (w), 1217 (w), 1044 (m), 742 (s).

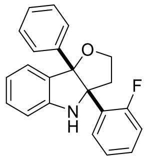

3a-(2-fluorophenyl)-8b-phenyl-3,3a,4,8b-tetrahydro-2*H*-furo[3,2-b]indole (**4l**)

This compound was prepared following the general procedure **E** using substrate **7l** (36 mg, 0.1 mmol) as starting material. Yield: 24% (8.0 mg), isolated as yellow oil. Purification: Flash chromatography (PE/EtOAc, 98:2), R_f = 0.33 (PE/EtOAc 90:10).

^{1}H NMR (400 MHz, $CDCl_3$) δ 7.57 (td, J = 8.0, 1.9 Hz, 1H), 7.21 (td, J = 7.6, 1.3 Hz, 1H), 7.13-7.06 (m, 2H), 7.06-6.96 (m, 4H), 6.96-6.90 (m, 2H), 6.79 – 6.74 (m, 2H), 6.61 (dd, J = 12.3, 8.0 Hz, 1H), 4.43 (t, J = 8.2 Hz, 1H), 4.12 (br, 1H), 3.94 – 3.86 (m, 1H), 3.17 (ddd, J = 12.6, 10.9, 7.9 Hz, 1H), 2.26 – 2.18 (m, 1H).

^{13}C NMR (101 MHz, $CDCl_3$): δ 160.1 (d, J = 248.3 Hz), 150.7, 140.8, 130.9, 130.0 (d, J = 11.0 Hz), 129.9, 129.1 (d, J = 8.8 Hz), 128.8 (d, J = 4.3 Hz), 127.3, 126.9, 126.8, 126.5, 123.4 (d, J = 3.4 Hz), 119.6, 116.1 (d, J = 23.8 Hz), 108.7, 98.6, 78.7 (d, J = 4.3 Hz), 66.5, 42.8 (d, J = 5.8 Hz).

^{19}F NMR (377 MHz, $CDCl_3$) δ -105.5.

HRMS (ESI/QTOF) m/z: $[M + H]^+$ Calcd for $C_{22}H_{19}FNO^+$ 332.1445; Found 332.1444.

IR (**ν_{max}**, cm^{-1}) 3364 (w), 3051 (w), 2922 (w), 2864 (w), 1609 (m), 1486 (m), 1446 (m), 1320 (m), 1270 (w), 1216 (m), 1044 (m), 749 (s).

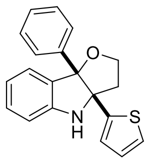

8b-phenyl-3a-(thiophen-2-yl)-3,3a,4,8b-tetrahydro-2*H*-furo[3,2-b]indole (**4m**)

This compound was prepared following the general procedure **E** using substrate **7m** (840 mg, 2.4 mmol) as starting material. Yield: 70% (531 mg), isolated as yellow oil. Purification: Flash chromatography (PE/EtOAc, 95:5), R_f = 0.43 (PE/EtOAc 90:10).

^{1}H NMR (400 MHz, $CDCl_3$) δ 7.23 (dd, J = 7.5, 1.3 Hz, 1H), 7.07 (br s, 5H), 6.99 – 6.96 (m, 1H), 6.93 (dd, J = 5.1, 1.2 Hz, 1H), 6.80 (d, J = 7.6 Hz, 2H), 6.68 (dd, J = 5.1, 3.6 Hz, 1H), 6.57 (dd, J = 3.6, 1.2 Hz, 1H), 4.40 (ddd, J = 8.7, 7.5, 1.2 Hz, 2H), 4.01 (ddd, J = 11.4, 8.5, 4.9 Hz, 1H), 2.77 (ddd, J = 12.8, 11.3, 7.6 Hz, 1H), 2.42 (ddd, J = 12.8, 5.0, 1.2 Hz, 1H).

^{13}C NMR (101 MHz, $CDCl_3$): δ 151.0, 148.1, 140.8, 130.1, 129.9, 127.5, 127.2, 127.1, 127.0, 126.9, 124.7, 124.3, 119.7, 108.8, 98.5, 79.0, 67.0, 42.8.

HRMS (ESI/QTOF) m/z: $[M + H]^+$ Calcd for $C_{20}H_{18}NOS^+$ 320.1104; Found 320.1112.

IR (ν_{max}, cm^{-1}) 3363 (w), 3053 (w), 2972 (w), 2877 (w), 1608 (m), 1483 (m), 1468 (m), 1446 (w), 1390 (w), 1313 (w), 1265 (w), 1236 (m), 1207 (w), 1174 (w), 1082 (w), 1047 (m), 1020 (w), 978 (w), 947 (w), 908 (m), 885 (w), 849 (w), 831 (w).

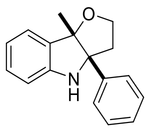

8b-methyl-3a-phenyl-3,3a,4,8b-tetrahydro-2*H*-furo[3,2-b]indole (**4n**)

This compound was prepared following the general procedure **E** using substrate **7n** (30 mg, 0.11 mmol) as starting material. Yield: 45% (12 mg), isolated as colorless oil. Purification: Flash chromatography (PE/EtOAc, 96.4:3.6), R_f = 0.39 (PE/EtOAc 90:10).

^{1}H NMR (400 MHz, $CDCl_3$) δ 7.48 – 7.42 (m, 2H), 7.37 – 7.31 (m, 2H), 7.31 – 7.25 (m, 1H), 7.24 – 7.15 (m, 2H), 6.79 (t, J = 7.4 Hz, 1H), 6.70 (d, J = 8.2 Hz, 1H), 4.25 – 4.11 (m, 2H), 3.81 – 3.73 (m, 1H), 2.93 – 2.81 (m, 1H), 2.26 – 2.16 (m, 1H), 1.12 (s, 3H).

^{13}C NMR (101 MHz, $CDCl_3$): δ 150.6, 142.8, 130.5, 129.9, 128.5, 127.5, 126.4, 124.6, 119.1, 108.6, 93.1, 77.6, 66.1, 41.1, 23.2.

HRMS (ESI/QTOF) m/z: $[M + H]^+$ Calcd for $C_{17}H_{18}NO^+$ 252.1383; Found 252.1380.

IR (ν_{max}, cm^{-1}) 3345 (w), 2968 (m), 2925 (m), 2878 (m), 1607 (m), 1484 (m), 1468 (m), 1446 (m), 1267 (m), 1105 (m), 1040 (m), 746 (s).

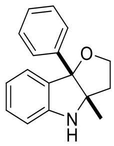

3a-methyl-8b-phenyl-3,3a,4,8b-tetrahydro-2\-furo[3,2-b]indole (**4o**)

This compound was prepared following the general procedure **E** using substrate **7o** (28 mg, 0.1 mmol) as starting material. Yield: 72% (18 mg), isolated as white solid. Purification: Flash chromatography (PE/EtOAc, 95:5), R_f = 0.24 (PE/EtOAc 90:10).

^{1}H NMR (400 MHz, $CDCl_3$) δ 7.38 – 7.24 (m, 5H), 7.19 (td, J = 7.6, 1.4 Hz, 1H), 7.03 (dd, J = 7.4, 1.3 Hz, 1H), 6.78 (td, J = 7.4, 1.0 Hz, 1H), 6.68 (d, J = 7.9 Hz, 1H), 4.21 (ddd, J = 8.8, 5.8, 3.2 Hz, 1H), 3.87 – 3.71 (m, 2H), 2.15 – 2.05 (m, 2H), 0.92 (s, 3H).

^{13}C NMR (101 MHz, $CDCl_3$): δ 151.1, 141.7, 130.9, 129.9, 127.8, 127.6, 127.3, 126.7, 119.3, 109.6, 96.4, 74.1, 66.6, 42.1, 25.2.

HRMS (ESI/QTOF) m/z: $[M + H]^+$ Calcd for $C_{17}H_{18}NO^+$ 252.1383; Found 252.1380.

IR (ν_{max}, cm^{-1}) 3352 (w), 3058 (w), 2975 (w), 2925 (w), 2871 (w), 2358 (w), 1609 (m), 1482 (m), 1468 (m), 1446 (m), 1309 (s), 1270 (m), 1213 (w), 1151 (w), 1044 (m), 986 (m), 943 (m), 746 (s).

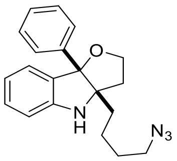

3a-(4-azidobutyl)-8b-phenyl-3,3a,4,8b-tetrahydro-2*H*-furo[3,2-b]indole (**4p**)

This compound was prepared following the general procedure **E** using substrate **7p** (30 mg, 0.08 mmol) as starting material. Yield: 66% (18 mg), isolated as colorless oil. Purification: Flash chromatography (PE/EtOAc, 96:4), R_f = 0.2 (PE/EtOAc 90:10).

^{1}H NMR (400 MHz, $CDCl_3$) δ 7.35 – 7.27 (m, 5H), 7.18 (td, J = 7.7, 1.3 Hz, 1H), 7.02 (dd, J = 7.5, 1.3 Hz, 1H), 6.77 (td, J = 7.4, 1.0 Hz, 1H), 6.68 (d, J = 7.8 Hz, 1H), 4.25 – 4.20 (m, 1H), 4.00 (brs, 1H), 3.79 – 3.73 (m, 1H), 3.14 (t, J = 6.3 Hz, 2H), 2.08 – 2.05 (m, 2H), 1.45 – 1.23 (m, 4H), 1.19 – 1.10 (m, 1H), 0.90 – 0.79 (m, 1H).

^{13}C NMR (101 MHz, $CDCl_3$): δ 151.1, 141.2, 130.7, 130.0, 127.8, 127.5, 126.6, 119.3, 109.4, 96.9, 76.3, 66.5, 51.3, 40.2, 38.3, 29.4, 22.9.

HRMS (ESI/QTOF) m/z: $[M + H]^+$ Calcd for $C_{20}H_{23}N_4O^+$ 335.1866; Found 335.1871.

IR (ν_{max}, cm^{-1}) 3365 (w), 2922 (m), 2843 (m), 2363 (w), 2094 (s), 1611 (s), 1483 (s), 1466 (s), 1269 (m), 1052 (m), 906 (m), 750 (s).

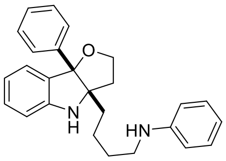

N-(4-(8b-phenyl-2,3,4,8b-tetrahydro-3a*H*-furo[3,2-b]indol-3a-yl)butyl)aniline (**4q**)

This compound was prepared following the general procedure **E** using substrate **7q** (35 mg, 0.09 mmol) as starting material. Yield: 66% (21.3 mg), isolated as brown oil. Purification: Flash chromatography (PE/EtOAc, 90:10), R_f = 0.58 (PE/EtOAc 65:35).

^{1}H NMR (400 MHz, $CDCl_3$) δ 7.38 – 7.27 (m, 5H), 7.22 – 7.12 (m, 3H), 7.03 (dd, J = 7.6, 1.3 Hz, 1H), 6.77 (td, J = 7.4, 1.0 Hz, 1H), 6.69 (t, J = 7.3 Hz, 1H), 6.67 (d, J =7.9 Hz, 1H), 6.53 (d, J =8.6 Hz, 2H), 4.23 (ddd, J = 8.7, 5.2, 3.6 Hz, 1H), 3.77 (td, J = 8.8, 7.4 Hz, 1H), 3.71 (brs, 1H), 2.98 (t, J = 6.5 Hz, 2H), 2.14 – 1.99 (m, 2H), 1.50 – 1.28 (m, 4H), 1.23 – 1.14 (m, 1H), 0.90 – 0.83 (m, 1H).

^{13}C NMR (101 MHz, $CDCl_3$): δ 151.1, 148.4, 141.3, 130.7, 129.9, 129.4, 127.84, 127.77, 127.5, 126.6, 119.2, 117.4, 112.8, 109.4, 96.9, 76.4, 66.6, 43.8, 40.2, 38.6, 30.1, 23.2.

HRMS (ESI/QTOF) m/z: $[M + H]^+$ Calcd for $C_{26}H_{29}N_2O^+$ 385.2274; Found 385.2271.

IR (ν_{max}, cm^{-1}) 3392 (w), 3029 (w), 2936 (m), 2864 (m), 1600 (m), 1508 (m), 1478 (m), 1468 (m), 1317 (m), 1255 (m), 1173 (m), 1054 (m), 746 (s).

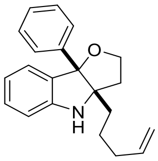

3a-(pent-4-en-1-yl)-8b-phenyl-3,3a,4,8b-tetrahydro-2*H*-furo[3,2-b]indole (**4r**)

This compound was prepared following the general procedure **E** using substrate **7r** (25 mg, 0.08 mmol) as starting material. Yield: 88% (20 mg), isolated as brown solid. Purification: Flash chromatography (PE/EtOAc, 95:5), R_f = 0.77 (PE/EtOAc 65:35).

^{1}H NMR (400 MHz, $CDCl_3$) δ 7.35 – 7.26 (m, 5H), 7.18 (td, J = 7.6, 1.3 Hz, 1H), 7.02 (dd, J = 7.5, 1.3 Hz, 1H), 6.76 (td, J = 7.4, 1.0 Hz, 1H), 6.67 (dt, J = 8.0, 0.8 Hz, 1H), 5.66 (ddt, J = 16.0, 10.8, 6.7 Hz, 1H), 4.91-4.85 (m, 2H), 4.23 (ddd, J = 8.7, 6.3, 2.4 Hz, 1H), 3.98 (br s, 1H), 3.78 (ddd, J = 9.9, 8.5, 6.1 Hz, 1H), 2.11 – 2.00 (m, 2H), 1.89 – 1.83 (m, 2H), 1.48 – 1.28 (m, 2H), 1.15 (ddd, J = 13.5, 11.8, 4.6 Hz, 1H), 0.81 (ddd, J = 13.5, 12.2, 4.6 Hz, 1H).

^{13}C NMR (101 MHz, $CDCl_3$): δ 151.2, 141.3, 138.3, 130.8, 129.9, 127.8, 127.7, 127.4, 126.6, 119.2, 114.8, 109.3, 96.9, 76.6, 66.7, 40.1, 38.2, 34.2, 25.1.

HRMS (ESI/QTOF) m/z: $[M + H]^+$ Calcd for $C_{21}H_{24}NO^+$ 306.1852; Found 306.1852.

IR (ν_{max}, cm^{-1}) 3374 (w), 3051 (w), 2939 (m), 2871 (m), 1607 (m), 1485 (m), 1468 (m), 1317 (w), 1270 (w), 1223 (w), 1048 (m), 907 (m), 746 (s), 702 (s).

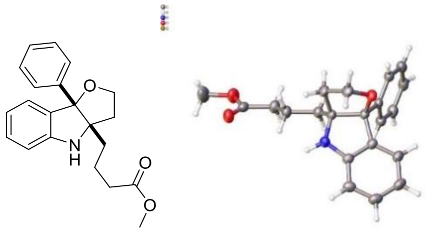

methyl 4-(8b-phenyl-2,3,4,8b-tetrahydro-3a*H*-furo[3,2-b]indol-3a-yl)butanoate (**4s**)

This compound was prepared following the general procedure **E** using substrate **7s** (30 mg, 0.08 mmol) as starting material. Yield: 58% (15.7 mg), isolated as purple crystals. Purification: Flash chromatography (PE/EtOAc, 90:10), R_f = 0.6 (PE/EtOAc 65:35). For X-Ray submission, the compound was recrystallized in Acetone/Hexane mixture with slow evaporation at room temperature.

^{1}H NMR (400 MHz, $CDCl_3$) δ 7.37 – 7.24 (m, 5H), 7.18 (ddd, J = 8.0, 7.4, 1.3 Hz, 1H), 7.01 (ddd, J = 7.5, 1.4, 0.6 Hz, 1H), 6.76 (td, J = 7.4, 1.0 Hz, 1H), 6.68 (dt, J = 7.9, 0.8 Hz, 1H), 4.23 (ddd, J = 8.7, 6.3, 2.4 Hz, 1H), 4.13 (brs, 1H), 3.77 (ddd, J = 9.9, 8.6, 6.2 Hz, 1H), 3.60 (s, 3H), 2.15 – 2.05 (m, 4H), 1.73 – 1.53 (m, 2H), 1.12 (ddd, J = 13.5, 11.8, 4.6 Hz, 1H), 0.82 (ddd, J = 13.5, 12.2, 4.9 Hz, 1H).

^{13}C NMR (101 MHz, $CDCl_3$): δ 173.9, 151.2, 141.2, 130.6, 129.9, 127.79, 127.76, 127.4, 126.6, 119.2, 109.4, 96.9, 76.3, 66.6, 51.6, 40.1, 38.1, 34.1, 21.0.

HRMS HRMS (ESI/QTOF) m/z: $[M + H]^+$ Calcd for $C_{21}H_{24}NO_3^+$ 338.1751; Found 338.1756.

IR (ν_{max}, cm^{-1}) 3381 (w), 3033 (w), 2943 (m), 2853 (w), 1737 (m), 1607 (m), 1482 (m), 1263 (m), 1170 (m), 1054 (m), 746 (s).

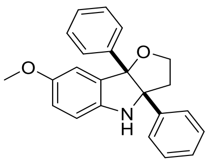

7-methoxy-3a,8b-diphenyl-3,3a,4,8b-tetrahydro-2*H*-furo[3,2-b]indole (**4t**)

This compound was prepared following the general procedure **E** using substrate **7t** (36.5 mg, 0.1 mmol) as starting material. Yield: 41% (14 mg), isolated as colorless oil. Purification: Flash chromatography (PE/EtOAc, 99:1), R_f = 0.31 (PE/EtOAc 90:10).

^{1}H NMR (400 MHz, $CDCl_3$) δ 7.13 – 7.09 (m, 2H), 7.06 – 6.96 (m, 8H), 6.85 (dd, J = 8.4, 2.6 Hz, 1H), 6.77 (m, 1H), 6.57 (brs, 1H), 4.45 (td, J = 8.0, 1.4 Hz, 1H), 4.15 (brs, 1H), 4.09 (ddd, J = 11.0, 8.4, 5.4 Hz, 1H), 3.67 (s, 3H), 2.91 (m, 1H), 2.28 (dd, J = 12.7, 5.1 Hz, 1H).

^{13}**C NMR** (151 MHz, $CDCl_3$): δ 153.9, 145.5, 142.3, 140.7, 131.6, 127.7, 127.5, 127.1, 126.8, 126.5, 116.9, 111.4, 109.9, 98.7, 81.0, 67.4, 56.1, 41.6.

HRMS (ESI/QTOF) m/z: $[M + H]^+$ Calculated for $C_{23}H_{22}NO_2^+$ 344.1645; Found 344.1637.

IR (ν_{max}, cm^{-1}) 3348 (w), 2927 (w), 2868 (w), 1493 (s), 1466 (m), 1448 (m), 1433 (m), 1263 (m), 1234 (m), 1213 (m), 1147 (m), 1047 (m), 1032 (m).

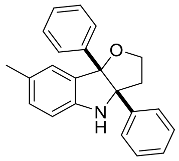

7-methyl-3a,8b-diphenyl-3,3a,4,8b-tetrahydro-2*H*-furo[3,2-b]indole (**4u**)

This compound was prepared following the general procedure **E** using substrate **7u** (35 mg, 0.1 mmol) as starting material. Yield: 53% (17 mg), isolated as colorless oil. Purification: Flash chromatography (PE/EtOAc, 90:10), R_f = 0.51 (PE/EtOAc 90:10).

^{1}**H NMR** (400 MHz, $CDCl_3$) δ 7.13-7.11 (m, 2H), 7.08-6.99 (m, 9H), 6.80 (brs, 1H), 6.75 (d, J = 8.0 Hz, 1H), 4.46 (t, J = 8.0 Hz, 1H), 4.19 (brs, 1H), 4.09 (ddd, J = 11.0, 8.3, 5.1 Hz, 1H), 2.92 (ddd, J = 12.5, 11.1, 7.6 Hz, 1H), 2.28 (ddd, J = 12.6, 5.3, 1.3 Hz, 1H), 2.24 (s, 3H).

^{13}**C NMR** (101 MHz, $CDCl_3$): δ 149.3, 142.2, 140.8, 130.6, 130.5, 128.7, 127.7, 127.5, 127.0, 126.7, 126.6, 126.4, 108.6, 98.4, 80.5, 67.2, 41.4, 20.8.

HRMS (ESI/QTOF) m/z: $[M + H]^+$ Calculated for $C_{23}H_{22}NO^+$ 328.1696; Found 328.1694.

IR (ν_{max}, cm^{-1}) 3367 (w), 3032 (w), 2927 (w), 2862 (w), 2158 (w), 1711 (w), 1614 (m), 1495 (s), 1446 (m), 1045 (m), 1034 (m).

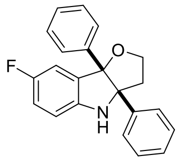

7-fluoro-3a,8b-diphenyl-3,3a,4,8b-tetrahydro-2*H*-furo[3,2-b]indole (**4v**)

This compound was prepared following the general procedure **E** using substrate **7v** (36 mg, 0.1 mmol) as starting material. Yield: 65% (21.5 mg), isolated as colorless oil. Purification: Flash chromatography (PE/EtOAc, 95:5), R_f = 0.43 (PE/EtOAc 90:10).

^{1}**H NMR** (400 MHz, $CDCl_3$) δ 7.11-7.08 (m, 2H), 7.05-6.92 (m, 9H), 6.74 (dd, J = 8.6, 4.1 Hz, 1H), 6.68 (dd, J = 8.1, 2.7 Hz, 1H), 4.46 (ddd, J = 8.7, 7.7, 1.4 Hz, 1H), 4.21 (br s, 1H), 4.07 (ddd, J = 11.1, 8.4, 5.3 Hz, 1H), 2.91 (ddd, J = 12.7, 11.0, 7.7 Hz, 1H), 2.29 (ddd, J = 12.7, 5.3, 1.4 Hz, 1H).

^{13}**C NMR** (101 MHz, $CDCl_3$): δ 157.4 (d, J = 235.9 Hz), 147.6, 142.0, 140.4, 132.1 (d, J = 7.0 Hz), 127.9, 127.5, 127.3, 127.1, 127.0, 126.5, 116.7 (d, J = 23.8 Hz), 113.6 (d, J = 23.8 Hz), 109.3 (d, J = 7.8 Hz), 98.3, 81.2, 67.5, 41.8.

^{19}**F NMR** (376 MHz, $CDCl_3$) δ -125.5

HRMS HRMS (ESI/QTOF) m/z: $[M + H]^+$ Calculated for $C_{22}H_{19}FNO^+$ 332.1445; Found 332.1441.

IR (ν_{max}, cm^{-1}) 127.8, 127.4, 127.2, 127.0, 126.9, 126.4, 116.7, 116.5, 113.6, 113.4, 109.2, 109.1, 98.3, 81.2, 67.5, 41.8. IR: ʋ (cm-1) 2920 (w), 1489 (s), 1446 (m), 1254 (m), 1203 (m), 1186 (m), 1138 (m), 1078 (m), 1045 (m), 1030 (m), 933 (m), 868 (m).

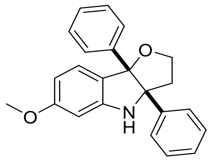

6-methoxy-3a,8b-diphenyl-3,3a,4,8b-tetrahydro-2*H*-furo[3,2-*b*]indole (**4w**)

This compound was prepared following the general procedure **E** using substrate **7w** (37 mg, 0.1 mmol) as starting material. Yield: 40% (13.7 mg), isolated as colorless oil. Purification: Flash chromatography (PE/EtOAc, 99:1), R_f = 0.34 (PE/EtOAc 90:10).

^{1}H NMR (400 MHz, $CDCl_3$) δ 7.13 – 7.09 (m, 2H), 7.06 – 6.95 (m, 8H), 6.85 (dd, J = 8.5, 2.6 Hz, 1H), 6.81-6.73 (m, 1H), 6.57 (d, J = 2.6 Hz, 1H), 4.45 (t, J = 8.0 Hz, 1H), 4.16 (brs, 1H), 4.09 (ddd, J = 11.0, 8.4, 5.4 Hz, 1H), 3.68 (s, 3H), 2.96-2.85 (m, 1H), 2.28 (dd, J = 12.6, 5.4 Hz, 1H).

^{13}C NMR (101 MHz, $CDCl_3$): δ 154.0, 145.6, 142.3, 140.7, 131.6, 127.8, 127.5, 127.1, 126.8, 126.5, 116.9, 111.4, 109.9, 98.8, 81.0, 67.4, 56.1, 41.7.

HRMS (ESI/QTOF) m/z: $[M + H]^+$ Calculated for $C_{23}H_{22}NO_2^+$ 344.1645; Found 344.1653.

IR (ν_{max}, cm^{-1}) 2922 (s), 2852 (m), 2158 (m), 2048 (m), 1493 (s), 1444 (m), 1263 (m), 1215 (m), 1034 (m).

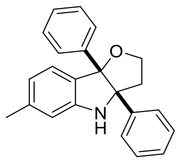

6-methyl-3a,8b-diphenyl-3,3a,4,8b-tetrahydro-2*H*-furo[3,2-*b*]indole (**4x**)

This compound was prepared following the general procedure **E** using substrate **7x** (36 mg, 0.1 mmol) as starting material. Yield: 53% (17.3 mg), isolated as colorless oil. Purification: Flash chromatography (PE/EtOAc, 90:10), R_f = 0.48 (PE/EtOAc 90:10).

^{1}H NMR (400 MHz, $CDCl_3$) δ 7.12 – 7.08 (m, 2H), 7.05 – 6.95 (m, 8H), 6.85 (d, J = 7.5 Hz, 1H), 6.65 (s, 1H), 6.61 (dt, J = 7.6, 1.1 Hz, 1H), 4.44 (ddd, J = 8.7, 7.6, 1.2 Hz, 1H), 4.26 (br s, 1H), 4.07 (ddd, J = 11.2, 8.4, 5.1 Hz, 1H), 2.91 (ddd, J = 12.7, 11.2, 7.6 Hz, 1H), 2.37 (s, 3H), 2.26 (ddd, J = 12.6, 5.1, 1.2 Hz, 1H).

^{13}C NMR (101 MHz, $CDCl_3$): δ 151.9, 142.2, 141.0, 140.2, 127.7, 127.7, 127.6, 127.0, 126.8, 126.7, 126.5, 120.3, 109.3, 98.3, 80.6, 67.2, 41.4, 21.9.

HRMS (ESI/QTOF) m/z: $[M + H]^+$ Calculated for $C_{23}H_{22}NO^+$ 328.1696; Found 328.1706.

IR (ν_{max}, cm^{-1}) 2920 (w), 1616 (w), 1593 (w), 1496 (w), 1466 (w), 1444 (w), 1317 (w), 1304 (w), 1281 (w), 1259 (w), 1228 (w), 1182 (w), 1153 (w), 1117 (w), 1080 (w), 1030 (w), 997 (w), 976 (w), 957 (w), 935 (w), 906 (s), 881 (w), 849 (w), 802 (w).

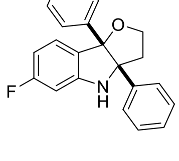

6-fluoro-3a,8b-diphenyl-3,3a,4,8b-tetrahydro-2*H*-furo[3,2-b]indole (**4y**)

This compound was prepared following the general procedure **E** using substrate **7y** (36 mg, 0.1 mmol) as starting material. Yield: 85% (28.2 mg), isolated as colorless oil. Purification: Flash chromatography (PE/EtOAc, 95:5), R_f = 0.46 (PE/EtOAc 90:10).

^{1}H NMR (400 MHz, $CDCl_3$) δ 7.10 – 6.96 (m, 10H), 6.87 (dd, J = 8.2, 5.7 Hz, 1H), 6.49 (dd, J = 9.8, 2.3 Hz, 1H), 6.44 (td, J = 8.8, 2.3 Hz, 1H), 4.45 (t, J = 8.0 Hz, 1H), 4.37 (brs, 1H), 4.06 (ddd, J = 11.2, 8.4, 5.0 Hz, 1H), 2.91 (td, J = 12.0, 7.6 Hz, 1H), 2.27 (dd, J = 12.7, 5.0 Hz, 1H).

^{13}C NMR (101 MHz, $CDCl_3$): δ 164.9 (d, J = 243.9 Hz), 152.9 (d, J = 2.2 Hz), 141.6, 140.6, 127.9, 127.8 (d, J = 11.0 Hz), 127.6, 127.1, 127.0, 126.9, 126.4, 126.0 (d, J = 2.2 Hz), 105.8 (d, J = 23.1 Hz), 97.8, 95.9 (d, J = 26.3 Hz), 81.3, 67.2, 41.3.

^{19}F NMR (376 MHz, $CDCl_3$) δ -112.6

HRMS (ESI/QTOF) m/z: $[M + H]^+$ Calculated for $C_{22}H_{19}FNO^+$ 332.1445; Found 332.1443.

IR (ν_{max}, cm^{-1}) 1618 (m), 1601 (m), 1495 (m), 1464 (w), 1444 (m), 1325 (w), 1288 (w), 1248 (w), 1227 (w), 1215 (w), 1173 (w), 1144 (m), 1099 (w), 1084 (w), 1043 (m), 999 (w), 980 (w), 962 (m), 941 (w), 906 (s), 881 (w), 829 (m).

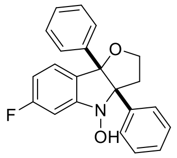

6-fluoro-3a,8b-diphenyl-2,3,3a,8b-tetrahydro-4H-furo[3,2-b]indol-4-ol (**10y**)

This compound was prepared following the general procedure **E** using substrate **7y** (36 mg, 0.1 mmol) as starting material. Reaction was stopped after 5h. Yield: 60% (22.6 mg), isolated as colorless oil. Purification: Flash chromatography (PE/EtOAc, 95:5), R_f = 0.46 (PE/EtOAc 90:10).

^{1}H NMR (600 MHz, $CDCl_3$) δ 7.24 (d, J = 7.8 Hz, 2H), 7.10 (t, J = 7.4 Hz, 2H), 7.05 (dd, J = 8.1, 5.8 Hz, 2H), 6.95 – 6.93 (m, 3H), 6.90 – 6.85 (m, 3H), 6.73 – 6.675 (m, 1H), 5.61 (s, 1H), 4.47 (td, J = 9.0, 4.4 Hz, 1H), 3.78 (q, J = 8.7 Hz, 1H), 2.93 (ddd, J = 13.9, 9.1, 4.4 Hz, 1H), 2.63 (dt, J = 14.3, 8.6 Hz, 1H).

^{13}C NMR (151 MHz, $CDCl_3$) δ 165.4, 163.8, 153.7 (d, J = 11.5 Hz), 140.4 (d, J = 133.5 Hz), 127.9, 127.2, 126.9 (d, J = 7.3 Hz), 126.7, 126.6, 126.4, 125.6, 110.1 (d, J = 22.9 Hz), 100.8 (d, J = 26.8 Hz), 95.4, 88.4, 67.047, 37.0.

^{19}F NMR (376 MHz, $CDCl_3$) δ -111.8.

IR (ν_{max}, cm^{-1}) 3358 (w), 3027 (w), 2884 (w), 1611 (m), 1486 (m), 1446 (m), 1327 (w), 1180 (w), 1158 (m), 1104 (w), 1065 (w), 1049 (m), 1034 (m), 987 (w), 946 (w), 909 (m), 852 (w), 808 (w), 751 (m), 735 (s), 698 (s)

HRMS (Sicrit plasma/LTQ-Orbitrap) m/z: $[M + H]^+$ Calcd for $C_{22}H_{19}FNO_2^+$ 348.1394; Found 348.1393.

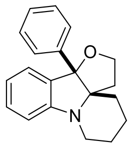

12b-phenyl-2,3,4,5,6,7-hexahydro-12b*H*-furo[3,2-b]pyrido[1,2-a]indole (**4z**)

This compound was prepared following the general procedure **E** using substrate **7z** (31.5 mg, 0.08 mmol) as starting material. Yield: 51% (14.8 mg), isolated as colorless oil. Purification: Flash chromatography (PE/EtOAc, 93:7), R_f = 0.57 (PE/EtOAc 80:20).

^{1}H NMR (600 MHz, $CDCl_3$) δ 7.34 – 7.26 (m, 5H), 7.19 (td, J = 7.7, 1.3 Hz, 1H), 7.04 (dd, J = 7.4, 1.4 Hz, 1H), 6.62 (td, J = 7.3, 1.0 Hz, 1H), 6.45 (d, J = 8.0 Hz, 1H), 4.26 (td, J = 8.2, 2.0 Hz, 1H), 3.76 (ddd, J = 10.7, 8.5, 5.9 Hz, 1H), 3.70 – 3.64 (m, 2H), 3.00 (td, J = 13.0, 3.2 Hz, 1H), 2.39 (ddd, J = 12.6, 5.9,

2.0 Hz, 1H), 1.91 (ddd, J = 12.6, 10.7, 7.9 Hz, 1H), 1.72-1.67 (m, 1H), 1.52 – 1.35 (m, 3H),1.03 (td, J = 13.1, 3.8 Hz, 1H).

^{13}C NMR (151 MHz, $CDCl_3$): δ 150.8, 141.0, 130.3, 129.9, 127.9, 127.7, 127.3, 126.4, 116.6, 105.0, 95.6, 67.5, 40.7, 34.3, 32.7, 29.9, 24.8, 22.5.

HRMS (ESI/QTOF) m/z: $[M + H]^+$ Calcd for $C_{20}H_{22}NO^+$ 292.1696; Found 292.1719.

IR (ν_{max}, cm^{-1}) 3378 (w), 2964 (m), 2881 (m), 2336 (w), 1524 (s), 1356 (m), 1262 (m), 1179 (m), 1042 (m), 739 (s), 706 (s).

3.7) General procedure F for the reductive cyclization of *o*-nitrostylbenes

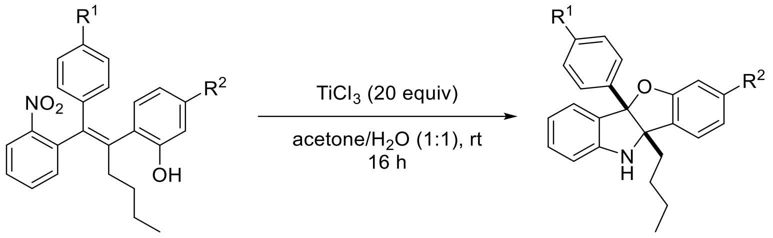

To a solution of *o*-nitrostilbene **8** (0.1 mmol, 1.0 equiv) in acetone (1.7 mL) at 23 °C was added a commercial solution of aqueous $TiCl_3$ (1.3 M solution in HCl, 1.7 mL, 20 equiv) in one portion. The solution was stirred at room temperature for 18 hours. The solution was poured into sat. aq. $NaHCO_3$ (20 mL) and extracted with DCM (3 x 10 mL). The combined organic layers are washed with brine, dried over sodium sulfate and concentrated. FCC (PE:EtOAc) afforded the corresponding benzofuroindoline.

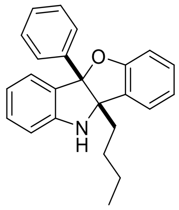

9b-butyl-4b-phenyl-4b,9b-dihydro-10*H*-benzofuro[3,2-b]indole (**5a**)

This compound was prepared following the general procedure **F** using substrate **8a** as starting material. Yield: 69% (23.6 mg, 0.069 mmol), isolated as yellow oil. Purification: Flash chromatography (PE/EtOAc, 4:1).

^{1}H NMR (600 MHz, $CDCl_3$) δ 7.37 – 7.23 (m, 6H), 7.23 – 7.12 (m, 3H), 6.97 – 6.89 (m, 2H), 6.79 (td, J = 7.6, 0.9 Hz, 1H), 6.71 (d, J = 7.8 Hz, 1H), 4.35 (s, 1H), 1.53-1.42 (m, 2H), 1.18 – 1.06 (m, 1H), 1.04 – 0.87 (m, 3H), 0.62 (t, J = 7.1 Hz, 3H).

^{13}C NMR (151 MHz, $CDCl_3$) δ 158.8, 150.2, 139.6, 132.4, 132.2, 130.2, 129.5, 127.9, 127.8, 127.3, 126.3, 123.9, 121.1, 120.3, 110.8, 110.8, 101.2, 77.4, 37.4, 25.9, 23.1, 13.8.

HRMS (ESI/QTOF) m/z: $[M + H]^+$ Calcd for $C_{24}H_{24}NO^+$ 342.1852; Found 342.1851.

IR (vmax, cm-1) 2933 (w), 1608 (m), 1466 (m), 1313 (w), 1240 (m), 1097 (w), 982 (m), 906 (m), 744 (s), 700 (s)

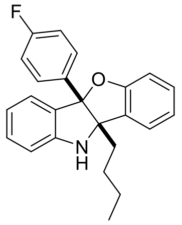

9b-butyl-4b-(4-fluorophenyl)-4b,9b-dihydro-10*H*-benzofuro[3,2-b]indole (**5b**)

This compound was prepared following the general procedure **F** using substrate **8b** as starting material. Yield: 89% (23.6 mg, 0.089 mmol), isolated as yellow oil. Purification: Flash chromatography (PE/EtOAc, 20:1).

^{1}H NMR (400 MHz, $CDCl_3$) δ 7.33 – 7.09 (m, 6H), 7.02 (tt, *J* = 8.7, 1.2 Hz, 2H), 6.96 – 6.90 (m, 2H), 6.78 (td, *J* = 7.5, 1.0 Hz, 1H), 6.70 (dt, *J* = 7.9, 0.8 Hz, 1H), 4.35 (s, 1H), 1.49 – 1.39 (m, 2H), 1.20 – 1.07 (m, 1H), 1.04-0.90 (m, 3H), 0.65 (t, *J* = 7.0 Hz, 3H).

^{13}C NMR (101 MHz, $CDCl_3$) δ 162.4 (d, *J* = 246.4 Hz), 158.6, 150.1, 135.5 (d, *J* = 3.1 Hz), 132.2, 131.9, 130.3, 129.5, 129.1 (d, *J* = 8.1 Hz), 126.2, 124.0, 121.3, 120.3, 114.8 (d, *J* = 21.5 Hz), 110.9, 110.8, 100.8, 77.3, 37.5, 25.9, 23.1, 13.8.

HRMS (ESI/QTOF) m/z: $[M + H]^+$ Calcd for $C_{24}H_{23}FNO^+$ 360.1758; Found 360.1762.

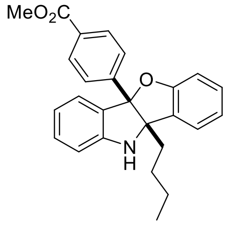

methyl 4-(9b-butyl-9b,10-dihydro-4b*H*-benzofuro[3,2-b]indol-4b-yl)benzoate (**5c**)

This compound was prepared following the general procedure **F** using substrate **8c** as starting material. Yield: 73% (23.6 mg, 0.073 mmol), isolated as yellow oil. Purification: Flash chromatography (PE/EtOAc, 3:1).

^{1}H NMR (400 MHz, $CDCl_3$) δ 8.01 – 7.99 (m, 2H), 7.39 (brs, 2H), 7.25 – 7.13 (m, 3H), 7.10 – 7.05 (m, 1H), 6.96-6.91 (m, 2H), 6.78 (td, *J* = 7.5, 1.0 Hz, 1H), 6.72 (dt, *J* = 7.9, 0.8 Hz, 1H), 4.39 (s, 1H), 3.92 (s, 3H), 1.50 – 1.44 (m, 2H), 1.17 – 1.03 (m, 1H), 1.02 – 0.85 (m, 3H), 0.61 (t, *J* = 7.0 Hz, 3H).

^{13}C NMR (101 MHz, $CDCl_3$) δ 167.1, 158.5, 150.1, 144.8, 132.1, 131.9, 130.4, 129.6, 129.6, 129.2, 127.3, 126.1, 123.9, 121.4, 120.4, 111.0, 110.9, 100.8, 77.8, 52.3, 37.4, 25.9, 23.0, 13.8.

HRMS (ESI/QTOF) m/z: $[M + H]^+$ Calcd for $C_{26}H_{26}NO_3^+$ 400.1907; Found 400.1902.

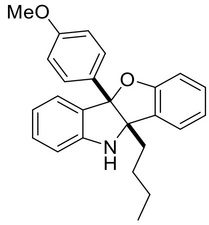

9b-butyl-4b-(4-methoxyphenyl)-4b,9b-dihydro-10*H*-benzofuro[3,2-b]indole (**5d**)

This compound was prepared following the general procedure **F** using substrate **8d** as starting material. Yield: 42% (23.6 mg, 0.042 mmol), isolated as yellow oil. Purification: Flash chromatography (PE/EtOAc, 5:1).

^{1}H NMR (800 MHz, $CDCl_3$) δ 7.24 (d, *J* = 7.4 Hz, 1H), 7.20 (brs, 2H), 7.18 (t, *J* = 7.7 Hz, 1H), 7.15 – 7.13 (m, 2H), 6.93-6.90 (m, 2H), 6.85 (d, *J* = 8.4 Hz, 2H), 6.78 (t, *J* = 7.4 Hz, 1H), 6.69 (d, *J* = 7.9 Hz, 1H), 4.35 (brs, 1H), 3.81 (s, 3H), 1.50 – 1.47 (m, 1H), 1.42 (td, *J* = 13.4, 4.1 Hz, 1H), 1.20 – 1.14 (m, 1H), 1.03 – 0.93 (m, 3H), 0.65 (t, *J* = 7.0 Hz, 3H).

^{13}C NMR (201 MHz, $CDCl_3$) δ 159.2, 158.8, 150.2, 132.4, 132.1, 131.7, 130.2, 129.4, 128.6, 126.4, 124.0, 121.0, 120.2, 113.3, 110.8, 110.7, 101.3, 77.4, 55.4, 37.5, 26.0, 23.2, 13.9.

HRMS (ESI/QTOF) m/z: $[M + H]^+$ Calcd for $C_{25}H_{26}NO_2^+$ 372.1958; Found 372.1955.

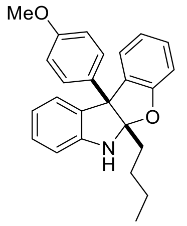

5a-butyl-10b-(4-methoxyphenyl)-5a,10b-dihydro-6*H*-benzofuro[2,3-b]indole (**2d**)

This compound was prepared following the general procedure **F** using substrate **8d** as starting material. Yield: 40% (23.6 mg, 0.040 mmol), isolated as yellow oil. Purification: Flash chromatography (PE/EtOAc, 5:1).

^{1}H NMR (400 MHz, $CDCl_3$) δ 7.26 – 7.23 (m, 1H), 7.14-7.04 (m, 3H), 7.00 – 6.96 (m, 2H), 6.90 (td, *J* = 7.5, 1.0 Hz, 1H), 6.84 – 6.75 (m, 4H), 6.71 (dt, *J* = 7.8, 0.8 Hz, 1H), 4.87 (brs, 1H), 3.80 (s, 3H), 1.47 (ddd, *J* = 13.6, 11.2, 4.1 Hz, 1H), 1.34 – 1.10 (m, 5H), 0.78 (t, *J* = 7.3 Hz, 3H).

^{13}C NMR (101 MHz, $CDCl_3$) δ 159.5, 158.9, 147.8, 133.7, 132.6, 132.3, 130.6, 128.4, 128.3, 125.2, 125.0, 121.0, 120.2, 113.5, 112.8, 109.8, 109.4, 66.9, 55.4, 36.4, 25.5, 23.1, 14.1.

HRMS (ESI/QTOF) m/z: $[M + H]^+$ Calcd for $C_{25}H_{26}NO_2^+$ 372.1958; Found 372.1970.

4) References

1. Li, B. X.; Le, D. N.; Mack, K. A.; McClory, A.; Lim, N. K.; Cravillion, T.; Savage, S.; Han, C.; Collum, D. B.; Zhang, H.; Gosselin, F. *J. Am. Chem. Soc.* **2017**, *139*, 10777−10783.
2. Lator, A.; Gaillard, Q. G.; Mérel, D. S.; Lohier, J.-F.; Gaillard, S.; Poater, A.; Renaud, J.-L. *J. Org. Chem.* **2019**, *84*, 6813–6829.

5) X-Ray Crystallographic data

X-Ray crystallographic data for 7h

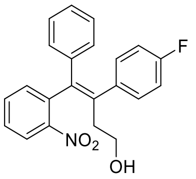

R_1=4.53%

Solved by: **Rosario Scopelliti**

Crystal Data and Experimental

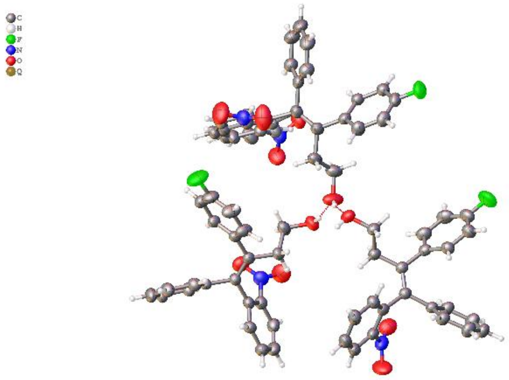

CCDC: 2523511
The ellipsoids are displayed at 50% probability level.

**Compound**	**db-7-006**
Formula	$C_{22}H_{18}FNO_3$
$D_{calc.}$/ g cm^{-3}	1.305
μ/mm^{-1}	0.772
Formula Weight	363.37
Colour	clear pale yellow
Shape	prism-shaped
Size/mm^3	0.49×0.10×0.08
T/K	200.00(12)
Crystal System	monoclinic
Space Group	$P2_1/c$
a/Å	6.32450(12)
b/Å	29.1905(6)
c/Å	30.0663(6)
α/°	90
β/°	90.6759(18)
γ/°	90
V/Å^3	5550.31(19)
Z	12
Z'	3
Wavelength/Å	1.54184
Radiation type	Cu K_α
Θ_{min}/°	2.940
Θ_{max}/°	72.409
Measured Refl's.	29026
Indep't Refl's	10638
Refl's I≥2 σ(I)	8045
R_{int}	0.0263
Parameters	989
Restraints	308
Largest Peak	0.242
Deepest Hole	-0.204
GooF	1.037
wR_2 (all data)	0.1334
wR_2	0.1184
R_1 (all data)	0.0623
R_1	0.0453

Experimental. Single clear pale yellow prism-shaped crystals of **db-7-006** were used as supplied. A suitable crystal with dimensions 0.49 × 0.10 × 0.08 mm^3 was selected and mounted on a SuperNova, Dual, Cu at home/near, Atlas diffractometer. The crystal was kept at a steady T = 200.00(12) K during data collection. The structure was solved with the **ShelXT** (Sheldrick, 2015) solution program using dual methods and by using **Olex2** 1.5 (Dolomanov et al., 2009) as the graphical interface. The model was refined with **ShelXL** 2018/3 (Sheldrick, 2015) using full matrix least squares minimisation on ***F*²**.

Crystal Data. $C_{22}H_{18}FNO_3$, M_r = 363.37, monoclinic, $P2_1/c$ (No. 14), a = 6.32450(12) Å, b = 29.1905(6) Å, c = 30.0663(6) Å, β = 90.6759(18)°, α = γ = 90°, V = 5550.31(19) Å^3, T = 200.00(12) K, Z = 12, Z' = 3, μ(Cu K_α) = 0.772, 29026 reflections measured, 10638 unique (R_{int} = 0.0263) which were used in all calculations. The final wR_2 was 0.1334 (all data) and R_1 was 0.0453 (I≥2 σ(I)).

Structure Quality Indicators

**Reflections:**	d min (Cu\a) 2Θ=144.8° 0.81	I/σ(I) 38.8	Rint 2.63%	Full 135.4° 97% to 144.8° 99.6
**Refinement:**	Shift -0.001	Max Peak 0.2	Min Peak -0.2	GooF 1.037

A clear pale yellow prism-shaped crystal with dimensions 0.49 × 0.10 × 0.08 mm³ was mounted. Data were collected using a SuperNova, Dual, Cu at home/near, Atlas diffractometer operating at T = 200.00(12) K.

Data were measured using ω scans with Cu K_α radiation. The diffraction pattern was indexed and the total number of runs and images was based on the strategy calculation from the program CrysAlisPro 1.171.42.83a (Rigaku OD, 2023). The maximum resolution that was achieved was Θ = 72.409° (0.81 Å).

The unit cell was refined using CrysAlisPro 1.171.42.83a (Rigaku OD, 2023) on 11795 reflections, 41% of the observed reflections.

Data reduction, scaling and absorption corrections were performed using CrysAlisPro 1.171.42.83a (Rigaku OD, 2023). The final completeness is 99.60 % out to 72.409° in Θ. A gaussian absorption correction was performed using CrysAlisPro 1.171.42.83a (Rigaku Oxford Diffraction, 2023). The numerical absorption correction was based on gaussian integration over a multifaceted crystal model. The empirical absorption correction was done using spherical harmonics, implemented in SCALE3 ABSPACK scaling algorithm. The absorption coefficient μ of this crystal is 0.772 mm^{-1} at this wavelength (λ = 1.54184Å) and the minimum and maximum transmissions are 0.599 and 1.000.

The structure was solved and the space group $P2_1/c$ (# 14) determined by the ShelXT (Sheldrick, 2015) structure solution program using dual methods and refined by full matrix least squares minimisation on $\boldsymbol{F^2}$ using version 2018/3 of **ShelXL** (Sheldrick, 2015). All non-hydrogen atoms were refined anisotropically. Most hydrogen atom positions were calculated geometrically and refined using the riding model, but some hydrogen atoms were refined freely.

The value of Z' is 3.

Data Plots: Diffraction Data

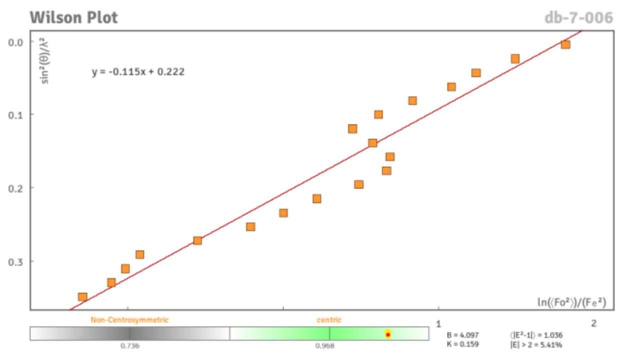

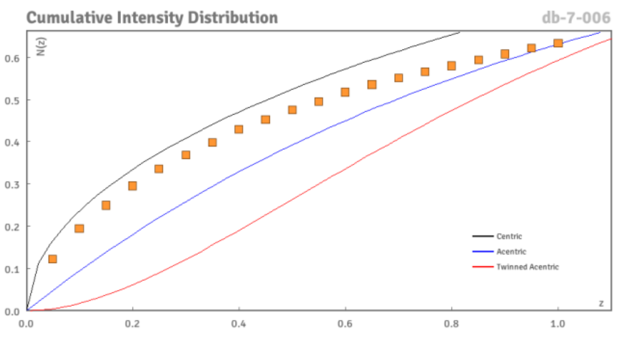

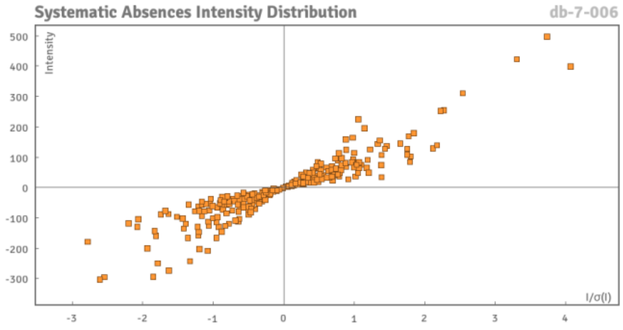

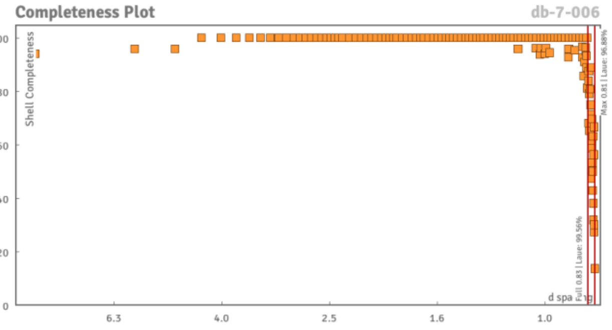

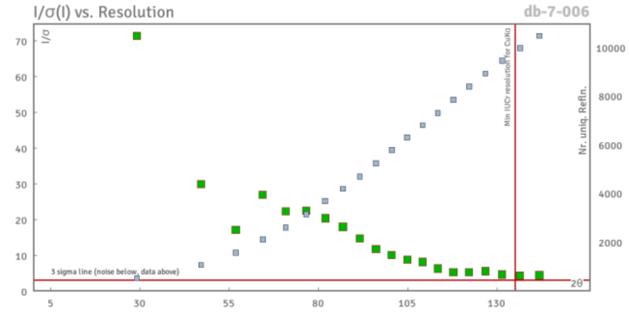

Data Plots: Refinement and Data

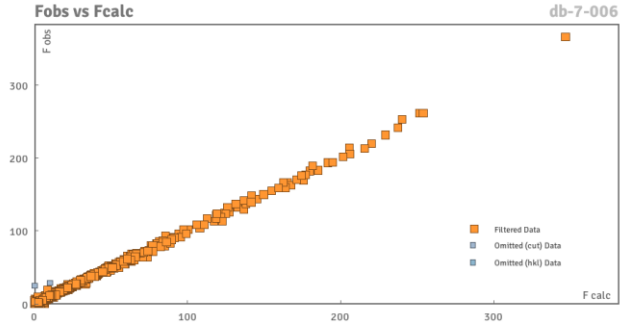

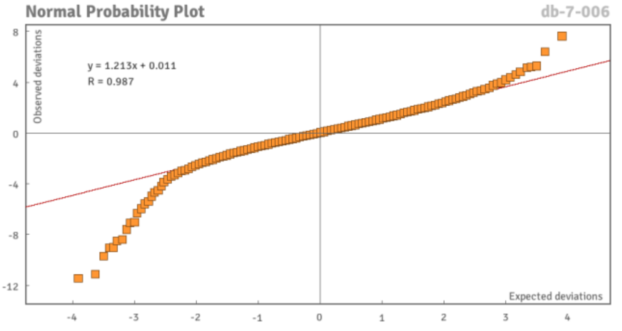

Reflection Statistics

Total reflections (after filtering)	29454	Unique reflections	10638
Completeness	0.969	Mean I/σ	16.18
hkl_{max} collected	(7, 34, 37)	hkl_{min} collected	(-6, -35, -36)
hkl_{max} used	(7, 35, 37)	hkl_{min} used	(-7, 0, 0)
Lim d_{max} collected	100.0	Lim d_{min} collected	0.77
d_{max} used	15.03	d_{min} used	0.81
Friedel pairs	2049	Friedel pairs merged	1
Inconsistent equivalents	25	R_{int}	0.0263
R_{sigma}	0.0258	Intensity transformed	0
Omitted reflections	0	Omitted by user (OMIT hkl)	6
Multiplicity	(12939, 4657, 1310, 440, 225, 62, 2)	Maximum multiplicity	11
Removed systematic absences	422	Filtered off (Shel/OMIT)	0

Table 1: Fractional Atomic Coordinates (×10^4) and Equivalent Isotropic Displacement Parameters (Å^2×10^3) for **db-7-006**. U_{eq} is defined as 1/3 of the trace of the orthogonalised U_{ij}.

Atom	x	y	z	U_{eq}
F1	6713(2)	8340.9(5)	8446.8(6)	87.2(5)
O1	4023(2)	6087.1(5)	7552.0(4)	52.3(3)

Atom	x	y	z	U_{eq}
O2	5734(3)	5874.0(7)	9622.9(6)	74.8(5)
O3	4061(4)	5491.7(8)	10118.0(6)	105.8(7)
N1	4195(4)	5664.7(7)	9746.3(6)	62.9(5)
C1	3566(3)	6384.1(7)	7911.8(6)	46.1(4)
C2	4516(3)	6213.3(7)	8347.9(6)	42.7(4)
C3	4072(3)	6533.1(6)	8732.0(6)	41.4(4)
C4	4820(3)	7013.1(6)	8671.3(5)	41.7(4)
C5	6850(3)	7111.2(8)	8522.8(7)	53.5(5)
C6	7489(4)	7558.1(9)	8447.7(7)	60.4(6)
C7	6094(4)	7904.2(7)	8519.6(7)	58.0(5)
C8	4074(4)	7827.4(8)	8662.7(7)	56.6(5)
C9	3458(3)	7379.7(7)	8735.4(6)	47.2(4)
C10	3016(3)	6397.1(6)	9097.0(6)	41.1(4)
C11	2702(3)	6703.4(6)	9488.6(5)	39.9(4)
C12	681(3)	6795.7(7)	9635.9(6)	47.5(4)
C13	361(3)	7105.3(8)	9977.5(7)	56.6(5)
C14	2049(3)	7314.1(7)	10186.1(7)	54.7(5)
C15	4064(3)	7214.4(8)	10054.8(7)	56.4(5)
C16	4392(3)	6912.6(8)	9707.6(6)	52.3(5)
C17	1928(3)	5944.5(6)	9124.1(6)	43.3(4)
C18	2389(3)	5608.2(7)	9442.6(6)	49.9(4)
C19	1216(4)	5211.5(7)	9485.1(7)	60.8(5)
C20	-479(4)	5137.1(8)	9205.8(8)	62.4(6)
C21	-993(3)	5458.7(7)	8884.0(8)	55.8(5)
C22	189(3)	5854.1(7)	8847.2(7)	48.6(4)
F2	6256(3)	4060.0(7)	5984.6(4)	94.9(5)
O4	11137(2)	5466.2(5)	7764.2(5)	56.7(3)
O5	14769(3)	4611.5(7)	8496.0(7)	92.0(6)
O6	14149(2)	4067.1(6)	8025.8(6)	69.0(4)
N2	13796(3)	4267.5(6)	8371.5(6)	58.2(4)
C23	11047(4)	4999.0(7)	7631.9(8)	56.8(5)
C24	9433(3)	4729.7(6)	7898.3(7)	45.3(4)
C25	9252(3)	4243.8(6)	7737.9(6)	40.7(4)
C26	8499(3)	4198.6(6)	7265.5(6)	42.3(4)
C27	9699(3)	3983.5(6)	6945.0(6)	46.8(4)
C28	8971(4)	3942.4(8)	6510.3(7)	56.4(5)
C29	7017(4)	4116.0(9)	6407.0(7)	63.8(6)
C30	5789(4)	4333.4(10)	6709.8(8)	69.9(7)
C31	6555(3)	4381.4(8)	7139.0(7)	57.8(5)
C32	9732(3)	3877.7(6)	7988.0(6)	40.3(4)
C33	9497(3)	3392.2(6)	7833.2(5)	41.4(4)
C34	11179(3)	3088.6(6)	7880.6(7)	49.7(4)
C35	10982(4)	2636.1(7)	7756.2(7)	57.7(5)
C36	9094(4)	2477.0(7)	7582.2(7)	59.4(5)
C37	7402(4)	2772.1(7)	7533.0(7)	57.3(5)
C38	7597(3)	3226.1(7)	7657.5(6)	49.0(4)
C39	10301(3)	3911.3(6)	8475.0(6)	42.6(4)
C40	12138(3)	4093.0(6)	8659.5(6)	47.6(4)
C41	12511(4)	4115.3(7)	9113.5(7)	54.0(5)
C42	11042(4)	3949.5(7)	9400.2(7)	60.8(5)
C43	9164(4)	3769.8(7)	9232.5(7)	59.0(5)
C44	8805(3)	3751.9(6)	8781.5(6)	48.0(4)
F3	5429(2)	8112.0(4)	6215.2(5)	74.1(4)
O7	8138(3)	5968.6(7)	7400.9(5)	75.1(5)
O8	12419(5)	6010.6(10)	6150.2(11)	65.1(9)
O9	12764(6)	5415.3(12)	6567.9(11)	77.5(10)
O10	5601(6)	5512.8(15)	5500.0(18)	102.4(15)
O11	6443(7)	4894.0(16)	5168.3(14)	99.8(14)
N3	12066(7)	5611.5(16)	6239.3(14)	54.3(11)
N4	6853(8)	5205.5(17)	5425.1(19)	63.6(13)
C45	8594(4)	6193.5(9)	6992.6(7)	63.2(6)

Atom	x	y	z	U_{eq}
C46	7358(4)	5995.0(8)	6604.7(7)	57.9(5)
C47	7670(3)	6254.6(7)	6175.2(7)	51.5(4)
C48	7061(3)	6749.5(6)	6189.3(6)	44.9(4)
C49	8491(3)	7085.9(6)	6065.9(6)	44.4(4)
C50	7961(3)	7545.0(7)	6074.1(6)	49.6(4)
C51	5965(3)	7662.0(7)	6210.9(6)	51.2(4)
C52	4522(3)	7347.1(8)	6345.1(7)	56.6(5)
C53	5089(3)	6888.6(8)	6337.8(7)	56.2(5)
C54	8472(3)	6061.5(6)	5806.9(7)	51.3(4)
C55	8691(3)	6305.0(6)	5372.6(6)	48.5(4)
C56	10614(4)	6311.4(8)	5158.4(7)	56.2(5)
C57	10854(4)	6533.8(9)	4756.7(8)	68.3(6)
C58	9170(5)	6748.9(9)	4557.9(7)	71.4(7)
C59	7227(5)	6739.8(9)	4760.5(8)	72.3(7)
C60	6980(4)	6521.7(9)	5165.9(8)	62.6(5)
C61	8927(5)	5551.5(7)	5739.3(10)	39.1(10)
C62	10699(5)	5361.8(9)	5946.1(8)	43.9(8)
C63	11207(5)	4904.6(10)	5874.5(12)	55.3(12)
C64	9944(6)	4637.2(8)	5596.2(12)	61.9(14)
C65	8173(5)	4827.0(9)	5389.3(11)	59.7(11)
C66	7664(5)	5284.2(10)	5460.9(11)	53.5(14)
C67	9688(5)	5592.9(9)	5858.8(13)	47.3(12)
C68	11647(6)	5566.1(10)	6071.7(14)	59.3(16)
C69	12795(5)	5160.3(12)	6060.8(13)	69.3(13)
C70	11984(6)	4781.2(10)	5837.0(14)	67.1(15)
C71	10025(6)	4808.0(9)	5624.1(13)	62.2(14)
C72	8877(4)	5213.9(10)	5635.0(11)	48.9(9)

Table 2: Anisotropic Displacement Parameters (×10[4]) for **db-7-006.** The anisotropic displacement factor exponent takes the form: $-2\pi^2[h^2a^{*2} \times U_{11} + ... + 2hka^* \times b^* \times U_{12}]$

Atom	U_{11}	U_{22}	U_{33}	U_{23}	U_{13}	U_{12}
F1	93.9(10)	63.3(8)	104.6(11)	19.0(8)	7.4(8)	-32.6(7)
O1	58.0(8)	59.4(8)	39.7(7)	-4.4(6)	5.9(6)	-9.6(7)
O2	64.4(10)	94.8(13)	65.0(10)	1.6(9)	-8.1(8)	15.6(9)
O3	171(2)	96.8(14)	48.8(9)	18.9(9)	-31.5(11)	-20.9(14)
N1	88.5(14)	56.0(10)	44.0(9)	-3.9(8)	-9.7(9)	15.0(10)
C1	51.9(11)	46.9(11)	39.6(9)	0.8(8)	1.3(8)	0.7(8)
C2	43.7(9)	45.2(10)	39.3(9)	0.8(7)	4.7(7)	3.5(8)
C3	41.2(8)	44.7(9)	38.4(8)	0.9(7)	2.1(7)	1.9(7)
C4	42.3(9)	48.3(10)	34.4(8)	-0.2(7)	0.6(7)	-4.0(7)
C5	44.5(10)	64.2(13)	51.9(11)	0.2(9)	6.7(8)	-3.7(9)
C6	48.6(11)	77.0(15)	55.8(12)	5.1(10)	5.5(9)	-20.7(11)
C7	67.0(12)	52.1(12)	54.9(11)	7.9(9)	-1.9(9)	-20.7(10)
C8	62.7(12)	46.6(11)	60.5(12)	4.9(9)	3.4(10)	-3.5(10)
C9	45.7(10)	46.8(10)	49.1(10)	3.3(8)	3.7(8)	-6.0(8)
C10	42.0(8)	43.5(9)	37.9(8)	1.9(7)	2.8(7)	3.2(7)
C11	42.9(9)	41.8(9)	34.9(8)	3.1(7)	3.9(7)	2.0(7)
C12	42.9(9)	54.8(11)	45.0(9)	-3.1(8)	3.7(8)	-0.3(8)
C13	49.0(11)	65.6(13)	55.4(11)	-10.0(10)	10.2(9)	6.8(9)
C14	64.3(12)	53.6(11)	46.5(10)	-10.0(9)	11.0(9)	-0.4(9)
C15	53.6(11)	68.1(13)	47.5(10)	-10.4(9)	0.9(9)	-8.9(10)
C16	41.5(10)	69.5(13)	45.9(10)	-8.6(9)	5.1(8)	-1.6(9)
C17	49.7(9)	41.2(9)	39.3(9)	-1.1(7)	9.0(7)	6.3(7)
C18	70.5(12)	42.7(10)	36.7(9)	-3.3(7)	5.7(8)	9.1(9)
C19	94.1(16)	40.1(10)	48.5(11)	1.7(9)	10.4(11)	3.1(10)
C20	79.9(15)	43.0(11)	64.7(13)	-0.7(10)	18.5(11)	-6.5(10)
C21	53.9(11)	47.8(11)	65.9(13)	-6.8(10)	6.1(10)	-2.1(9)

Atom	U_{11}	U_{22}	U_{33}	U_{23}	U_{13}	U_{12}
C22	51.2(10)	41.7(10)	52.9(10)	2.4(8)	1.0(8)	2.2(8)
F2	96.9(10)	145.4(15)	42.2(7)	1.3(8)	-12.5(7)	13.6(10)
O4	64.1(9)	38.9(7)	67.2(9)	5.9(6)	-3.7(7)	-5.4(6)
O5	80.6(11)	95.1(14)	100.3(14)	-7.7(11)	6.8(10)	-43.3(10)
O6	57.2(8)	84.7(11)	65.5(10)	-0.5(8)	13.4(7)	4.3(8)
N2	48.9(9)	62.1(11)	63.7(11)	3.3(9)	4.2(8)	-3.2(8)
C23	62.7(13)	40.2(10)	67.9(14)	4.6(9)	14.1(10)	1.3(9)
C24	52.0(10)	35.9(9)	48.1(10)	2.5(7)	6.4(8)	4.5(8)
C25	40.7(8)	38.9(9)	42.6(9)	2.0(7)	5.1(7)	4.4(7)
C26	47.5(9)	39.0(9)	40.5(9)	6.8(7)	6.2(7)	4.2(7)
C27	52.0(10)	43.4(10)	45.1(9)	2.8(8)	5.9(8)	7.1(8)
C28	66.8(12)	57.5(12)	44.9(10)	-0.6(9)	8.8(9)	5.6(10)
C29	73.5(14)	80.1(15)	37.7(10)	7.3(10)	-3.2(9)	2.5(12)
C30	59.0(13)	99.3(19)	51.3(12)	16.5(12)	-1.3(10)	22.3(12)
C31	55.2(11)	74.9(14)	43.5(10)	8.4(10)	7.2(8)	20.3(10)
C32	43.4(9)	36.3(9)	41.2(9)	0.4(7)	-0.3(7)	0.3(7)
C33	53.2(10)	37.3(9)	33.7(8)	3.6(7)	-0.8(7)	-0.2(7)
C34	57.7(11)	40.0(10)	51.2(10)	0.3(8)	-9.1(9)	3.1(8)
C35	72.5(13)	40.7(10)	59.9(12)	-3.0(9)	-9.8(10)	7.1(10)
C36	87.8(15)	40.6(11)	49.7(11)	-6.0(9)	-6.0(10)	-3.9(10)
C37	68.6(13)	51.5(12)	51.5(11)	-2.1(9)	-8.7(10)	-12.8(10)
C38	52.2(11)	48.4(11)	46.5(10)	2.7(8)	-4.3(8)	-2.4(9)
C39	52.0(9)	32.6(8)	43.3(9)	0.1(7)	0.5(7)	1.5(7)
C40	52.9(10)	41.4(10)	48.4(10)	-0.1(8)	0.3(8)	1.8(8)
C41	66.7(13)	46.4(11)	48.8(10)	-4.5(8)	-8.6(9)	0.9(9)
C42	89.2(16)	50.8(12)	42.3(10)	-1.0(9)	-4.3(10)	1.9(11)
C43	81.8(15)	48.1(11)	47.4(11)	3.6(9)	10.9(10)	-3.2(10)
C44	55.9(11)	42.3(10)	45.7(10)	4.7(8)	3.3(8)	-5.4(8)
F3	87.2(9)	48.5(7)	86.3(9)	-5.1(6)	-3.5(7)	21.1(6)
O7	65.8(10)	100.4(13)	59.6(9)	32.6(9)	14.9(7)	22.4(9)
O8	62.7(17)	46.8(17)	85(2)	-1.2(14)	-13.2(15)	-8.9(13)
O9	90(2)	75(2)	67(2)	1.0(17)	-33.8(18)	-3.2(17)
O10	57(2)	90(3)	160(4)	-38(3)	-22(2)	3.4(19)
O11	106(3)	98(3)	94(3)	-31(2)	-40(2)	2(2)
N3	52(2)	52(2)	58(3)	-4.1(19)	-9.4(19)	3.2(17)
N4	72(3)	55(3)	64(2)	-6(2)	0(2)	-15(2)
C45	67.2(14)	67.0(15)	55.7(12)	18.9(11)	9.3(10)	4.3(11)
C46	67.5(13)	47.9(12)	58.6(12)	11.9(9)	18.8(10)	2.7(10)
C47	59.4(11)	45.1(10)	50.4(10)	4.0(8)	12.6(8)	0.5(8)
C48	52.7(10)	45.2(10)	37.0(8)	-0.4(7)	4.7(7)	2.1(8)
C49	48.8(10)	45.9(10)	38.8(9)	0.4(7)	4.9(7)	2.6(8)
C50	59.7(11)	45.2(10)	43.9(10)	2.0(8)	3.1(8)	-2.0(9)
C51	62.1(11)	43.6(10)	47.7(10)	-3.3(8)	-5.5(9)	11.6(9)
C52	48.4(11)	60.8(13)	60.5(12)	-10.0(10)	1.8(9)	10.7(9)
C53	50.5(11)	55.9(12)	62.4(12)	-4.3(10)	11.9(9)	-2.8(9)
C54	58.6(11)	39.3(10)	56.3(11)	1.5(8)	10.7(9)	0.6(8)
C55	61.7(11)	40.8(10)	43.1(9)	-6.7(8)	3.7(8)	-0.9(8)
C56	63.9(12)	55.4(12)	49.4(11)	-0.6(9)	6.2(9)	1.7(10)
C57	83.2(16)	72.1(15)	50.0(12)	1.8(11)	14.4(11)	0.6(13)
C58	108(2)	65.3(14)	40.5(11)	-0.5(10)	4.4(12)	2.9(13)
C59	92.9(18)	71.0(16)	52.5(12)	-1.5(11)	-15.1(12)	13.0(13)
C60	61.9(13)	70.0(15)	55.8(12)	-2.1(11)	1.2(10)	4.3(11)
C61	44(2)	40(2)	34(2)	-0.1(16)	-2.0(17)	-2.5(17)
C62	49(2)	41(2)	42.3(18)	3.5(16)	-4.5(16)	6.0(17)
C63	76(3)	37(2)	53(2)	2.7(18)	-6(2)	10(2)
C64	99(3)	33(3)	54(2)	2(2)	3(2)	0(2)
C65	79(3)	46(2)	54(2)	-4.3(17)	-8(2)	-14.4(19)
C66	61(3)	49(3)	50(2)	2(2)	-9(2)	-13(2)
C67	52(3)	44(2)	46(3)	-1(2)	-2(2)	-14(2)
C68	46(3)	51(3)	81(4)	5(3)	-20(3)	-6(2)
C69	64(3)	56(3)	87(3)	11(2)	-8(2)	6(2)

Atom	U_{11}	U_{22}	U_{33}	U_{23}	U_{13}	U_{12}
C70	82(3)	46(3)	74(3)	7(2)	4(3)	19(2)
C71	91(3)	39(3)	57(3)	5(2)	11(3)	2(2)
C72	58(2)	42(2)	46(2)	0.2(17)	3.5(18)	-7.9(18)

Table 3: Bond Lengths in Å for **db-7-006**.

Atom	Atom	Length/Å
F1	C7	1.352(2)
O1	C1	1.419(2)
O2	N1	1.212(3)
O3	N1	1.230(3)
N1	C18	1.463(3)
C1	C2	1.520(3)
C2	C3	1.514(2)
C3	C4	1.491(3)
C3	C10	1.351(2)
C4	C5	1.394(3)
C4	C9	1.388(3)
C5	C6	1.385(3)
C6	C7	1.360(3)
C7	C8	1.371(3)
C8	C9	1.382(3)
C10	C11	1.494(2)
C10	C17	1.492(3)
C11	C12	1.384(2)
C11	C16	1.390(3)
C12	C13	1.384(3)
C13	C14	1.374(3)
C14	C15	1.370(3)
C15	C16	1.384(3)
C17	C18	1.400(3)
C17	C22	1.397(3)
C18	C19	1.382(3)
C19	C20	1.371(4)
C20	C21	1.384(3)
C21	C22	1.380(3)
F2	C29	1.363(2)
O4	C23	1.422(2)
O5	N2	1.234(2)
O6	N2	1.216(2)
N2	C40	1.459(3)
C23	C24	1.524(3)
C24	C25	1.502(3)
C25	C26	1.499(2)
C25	C32	1.339(2)
C26	C27	1.384(3)
C26	C31	1.389(3)
C27	C28	1.386(3)
C28	C29	1.368(3)
C29	C30	1.360(3)
C30	C31	1.380(3)
C32	C33	1.499(2)
C32	C39	1.507(2)
C33	C34	1.391(3)
C33	C38	1.394(3)
C34	C35	1.378(3)
C35	C36	1.378(3)
C36	C37	1.381(3)
C37	C38	1.382(3)
C39	C40	1.387(3)
C39	C44	1.408(3)
C40	C41	1.384(3)
C41	C42	1.364(3)
C42	C43	1.388(3)
C43	C44	1.373(3)
F3	C51	1.357(2)
O7	C45	1.425(3)
O8	N3	1.217(5)
O9	N3	1.220(5)
O10	N4	1.219(6)
O11	N4	1.219(6)
N3	C62	1.428(4)
N4	C72	1.421(5)
C45	C46	1.512(4)
C46	C47	1.512(3)
C47	C48	1.496(3)
C47	C54	1.347(3)
C48	C49	1.388(3)
C48	C53	1.390(3)
C49	C50	1.382(3)
C50	C51	1.375(3)
C51	C52	1.360(3)
C52	C53	1.386(3)
C54	C55	1.495(3)
C54	C61	1.530(3)
C54	C67	1.576(3)
C55	C56	1.383(3)
C55	C60	1.393(3)
C56	C57	1.381(3)
C57	C58	1.368(4)
C58	C59	1.379(4)
C59	C60	1.385(3)
C61	C62	1.3900
C61	C66	1.3900
C62	C63	1.3900
C63	C64	1.3900
C64	C65	1.3900
C65	C66	1.3900
C67	C68	1.3900
C67	C72	1.3900
C68	C69	1.3900
C69	C70	1.3900
C70	C71	1.3900
C71	C72	1.3900

Table 4: Bond Angles in ° for **db-7-006**.

Atom	Atom	Atom	Angle/°
O2	N1	O3	123.3(2)
O2	N1	C18	119.35(17)
O3	N1	C18	117.3(2)
O1	C1	C2	112.05(15)
C3	C2	C1	112.39(15)
C4	C3	C2	115.11(15)
C10	C3	C2	122.44(17)
C10	C3	C4	122.41(16)
C5	C4	C3	121.78(17)
C9	C4	C3	120.63(16)
C9	C4	C5	117.47(18)
C6	C5	C4	121.1(2)
C7	C6	C5	118.9(2)
F1	C7	C6	119.0(2)
F1	C7	C8	118.5(2)
C6	C7	C8	122.5(2)
C7	C8	C9	118.0(2)
C8	C9	C4	122.04(19)
C3	C10	C11	122.42(16)
C3	C10	C17	122.50(16)
C17	C10	C11	114.90(14)
C12	C11	C10	120.07(16)
C12	C11	C16	118.07(17)
C16	C11	C10	121.84(15)
C11	C12	C13	120.54(18)
C14	C13	C12	120.56(19)
C15	C14	C13	119.64(19)
C14	C15	C16	120.04(19)
C15	C16	C11	121.07(18)
C18	C17	C10	124.46(17)
C22	C17	C10	119.69(16)
C22	C17	C18	115.55(18)
C17	C18	N1	120.16(18)
C19	C18	N1	116.91(19)
C19	C18	C17	122.92(19)
C20	C19	C18	119.5(2)
C19	C20	C21	119.8(2)
C22	C21	C20	120.0(2)
C21	C22	C17	122.26(19)
O5	N2	C40	117.61(19)
O6	N2	O5	123.72(19)
O6	N2	C40	118.67(17)
O4	C23	C24	111.88(17)
C25	C24	C23	111.55(16)
C26	C25	C24	114.17(15)
C32	C25	C24	123.87(17)
C32	C25	C26	121.96(16)
C27	C26	C25	121.91(16)
C27	C26	C31	118.32(18)
C31	C26	C25	119.76(16)
C26	C27	C28	121.14(18)
C29	C28	C27	118.10(19)
F2	C29	C28	118.4(2)
C30	C29	F2	118.8(2)
C30	C29	C28	122.8(2)
C29	C30	C31	118.4(2)
C30	C31	C26	121.1(2)
C25	C32	C33	124.01(16)

Atom	Atom	Atom	Angle/°
C25	C32	C39	122.92(16)
C33	C32	C39	112.63(14)
C34	C33	C32	119.89(16)
C34	C33	C38	118.16(17)
C38	C33	C32	121.89(16)
C35	C34	C33	121.09(19)
C34	C35	C36	120.1(2)
C35	C36	C37	119.8(2)
C36	C37	C38	120.2(2)
C37	C38	C33	120.68(19)
C40	C39	C32	127.07(16)
C40	C39	C44	115.54(17)
C44	C39	C32	117.37(16)
C39	C40	N2	120.04(17)
C41	C40	N2	116.94(18)
C41	C40	C39	123.01(19)
C42	C41	C40	119.8(2)
C41	C42	C43	119.4(2)
C44	C43	C42	120.3(2)
C43	C44	C39	121.94(19)
O8	N3	O9	124.2(4)
O8	N3	C62	117.7(4)
O9	N3	C62	118.1(4)
O10	N4	C72	119.3(4)
O11	N4	O10	122.2(5)
O11	N4	C72	118.5(5)
O7	C45	C46	112.3(2)
C45	C46	C47	113.26(18)
C48	C47	C46	115.06(17)
C54	C47	C46	123.14(18)
C54	C47	C48	121.79(17)
C49	C48	C47	120.43(17)
C49	C48	C53	117.97(18)
C53	C48	C47	121.57(18)
C50	C49	C48	121.48(18)
C51	C50	C49	118.02(19)
F3	C51	C50	118.24(19)
F3	C51	C52	118.89(18)
C52	C51	C50	122.86(19)
C51	C52	C53	118.25(19)
C52	C53	C48	121.4(2)
C47	C54	C55	123.99(17)
C47	C54	C61	126.1(2)
C47	C54	C67	118.0(2)
C55	C54	C61	109.09(19)
C55	C54	C67	116.7(2)
C56	C55	C54	120.30(18)
C56	C55	C60	117.9(2)
C60	C55	C54	121.74(19)
C57	C56	C55	121.4(2)
C58	C57	C56	120.3(2)
C57	C58	C59	119.5(2)
C58	C59	C60	120.5(2)
C59	C60	C55	120.4(2)
C62	C61	C54	118.7(2)
C62	C61	C66	120.0
C66	C61	C54	121.2(2)
C61	C62	N3	123.5(3)
C61	C62	C63	120.0

Atom	Atom	Atom	Angle/°
C63	C62	N3	116.5(3)
C64	C63	C62	120.0
C63	C64	C65	120.0
C64	C65	C66	120.0
C65	C66	C61	120.0
C68	C67	C54	121.7(2)
C68	C67	C72	120.0
C72	C67	C54	117.8(2)
C69	C68	C67	120.0
C68	C69	C70	120.0
C71	C70	C69	120.0
C70	C71	C72	120.0
C67	C72	N4	123.7(3)
C71	C72	N4	116.3(3)
C71	C72	C67	120.0

Table 5: Torsion Angles in ° for **db-7-006.**

Atom	Atom	Atom	Atom	Angle/°
F1	C7	C8	C9	179.91(19)
O1	C1	C2	C3	178.87(16)
O2	N1	C18	C17	-30.7(3)
O2	N1	C18	C19	148.6(2)
O3	N1	C18	C17	149.6(2)
O3	N1	C18	C19	-31.1(3)
N1	C18	C19	C20	-179.17(19)
C1	C2	C3	C4	-57.3(2)
C1	C2	C3	C10	120.72(19)
C2	C3	C4	C5	-47.4(2)
C2	C3	C4	C9	128.57(18)
C2	C3	C10	C11	175.89(15)
C2	C3	C10	C17	-9.3(3)
C3	C4	C5	C6	176.88(18)
C3	C4	C9	C8	-177.07(18)
C3	C10	C11	C12	123.3(2)
C3	C10	C11	C16	-55.0(3)
C3	C10	C17	C18	120.9(2)
C3	C10	C17	C22	-65.7(2)
C4	C3	C10	C11	-6.3(3)
C4	C3	C10	C17	168.58(16)
C4	C5	C6	C7	-0.3(3)
C5	C4	C9	C8	-0.9(3)
C5	C6	C7	F1	179.96(19)
C5	C6	C7	C8	-0.2(3)
C6	C7	C8	C9	0.1(3)
C7	C8	C9	C4	0.5(3)
C9	C4	C5	C6	0.8(3)
C10	C3	C4	C5	134.63(19)
C10	C3	C4	C9	-49.4(2)
C10	C11	C12	C13	-175.23(18)
C10	C11	C16	C15	176.53(19)
C10	C17	C18	N1	-7.2(3)
C10	C17	C18	C19	173.58(18)
C10	C17	C22	C21	-174.20(18)
C11	C10	C17	C18	-63.9(2)
C11	C10	C17	C22	109.51(18)
C11	C12	C13	C14	-2.3(3)
C12	C11	C16	C15	-1.8(3)
C12	C13	C14	C15	0.0(3)
C13	C14	C15	C16	1.4(3)
C14	C15	C16	C11	-0.5(3)
C16	C11	C12	C13	3.1(3)
C17	C10	C11	C12	-51.9(2)
C17	C10	C11	C16	129.82(19)
C17	C18	C19	C20	0.1(3)
C18	C17	C22	C21	-0.2(3)

Atom	Atom	Atom	Atom	Angle/°
C18	C19	C20	C21	0.2(3)
C19	C20	C21	C22	-0.5(3)
C20	C21	C22	C17	0.5(3)
C22	C17	C18	N1	179.17(17)
C22	C17	C18	C19	-0.1(3)
F2	C29	C30	C31	178.8(2)
O4	C23	C24	C25	176.53(17)
O5	N2	C40	C39	143.1(2)
O5	N2	C40	C41	-37.6(3)
O6	N2	C40	C39	-37.2(3)
O6	N2	C40	C41	142.14(19)
N2	C40	C41	C42	-178.69(19)
C23	C24	C25	C26	-62.2(2)
C23	C24	C25	C32	117.2(2)
C24	C25	C26	C27	120.69(19)
C24	C25	C26	C31	-58.5(2)
C24	C25	C32	C33	178.25(16)
C24	C25	C32	C39	6.3(3)
C25	C26	C27	C28	179.61(18)
C25	C26	C31	C30	-178.0(2)
C25	C32	C33	C34	129.29(19)
C25	C32	C33	C38	-53.4(3)
C25	C32	C39	C40	-70.3(3)
C25	C32	C39	C44	108.1(2)
C26	C25	C32	C33	-2.3(3)
C26	C25	C32	C39	-174.27(16)
C26	C27	C28	C29	-0.8(3)
C27	C26	C31	C30	2.8(3)
C27	C28	C29	F2	-177.3(2)
C27	C28	C29	C30	1.3(4)
C28	C29	C30	C31	0.2(4)
C29	C30	C31	C26	-2.3(4)
C31	C26	C27	C28	-1.2(3)
C32	C25	C26	C27	-58.8(2)
C32	C25	C26	C31	122.1(2)
C32	C33	C34	C35	177.59(18)
C32	C33	C38	C37	-177.51(18)
C32	C39	C40	N2	-1.6(3)
C32	C39	C40	C41	179.04(18)
C32	C39	C44	C43	-179.58(18)
C33	C32	C39	C40	116.90(19)
C33	C32	C39	C44	-64.6(2)
C33	C34	C35	C36	-0.1(3)
C34	C33	C38	C37	-0.2(3)
C34	C35	C36	C37	0.0(3)
C35	C36	C37	C38	0.0(3)
C36	C37	C38	C33	0.1(3)
C38	C33	C34	C35	0.2(3)
C39	C32	C33	C34	-58.0(2)
C39	C32	C33	C38	119.22(18)
C39	C40	C41	C42	0.7(3)
C40	C39	C44	C43	-0.9(3)
C40	C41	C42	C43	-1.5(3)
C41	C42	C43	C44	1.1(3)
C42	C43	C44	C39	0.1(3)
C44	C39	C40	N2	179.89(17)
C44	C39	C40	C41	0.6(3)
F3	C51	C52	C53	179.84(18)
O7	C45	C46	C47	174.96(18)
O8	N3	C62	C61	-37.3(6)
O8	N3	C62	C63	143.2(4)

Atom	Atom	Atom	Atom	Angle/°
O9	N3	C62	C61	141.0(4)
O9	N3	C62	C63	-38.5(5)
O10	N4	C72	C67	-12.3(7)
O10	N4	C72	C71	165.0(5)
O11	N4	C72	C67	166.2(4)
O11	N4	C72	C71	-16.5(6)
N3	C62	C63	C64	179.5(3)
C45	C46	C47	C48	-59.0(3)
C45	C46	C47	C54	119.8(2)
C46	C47	C48	C49	126.1(2)
C46	C47	C48	C53	-51.9(3)
C46	C47	C54	C55	176.7(2)
C46	C47	C54	C61	8.1(4)
C46	C47	C54	C67	-16.8(3)
C47	C48	C49	C50	179.68(17)
C47	C48	C53	C52	-179.19(19)
C47	C54	C55	C56	127.6(2)
C47	C54	C55	C60	-53.9(3)
C47	C54	C61	C62	-75.7(3)
C47	C54	C61	C66	107.3(3)
C47	C54	C67	C68	-69.8(3)
C47	C54	C67	C72	118.4(3)
C48	C47	C54	C55	-4.5(3)
C48	C47	C54	C61	-173.1(2)
C48	C47	C54	C67	162.0(2)
C48	C49	C50	C51	0.3(3)
C49	C48	C53	C52	2.8(3)
C49	C50	C51	F3	-179.34(17)
C49	C50	C51	C52	1.3(3)
C50	C51	C52	C53	-0.8(3)
C51	C52	C53	C48	-1.3(3)
C53	C48	C49	C50	-2.3(3)
C54	C47	C48	C49	-52.8(3)
C54	C47	C48	C53	129.3(2)
C54	C55	C56	C57	179.9(2)
C54	C55	C60	C59	-179.2(2)
C54	C61	C62	N3	3.5(4)
C54	C61	C62	C63	-177.1(3)
C54	C61	C66	C65	177.0(3)
C54	C67	C68	C69	-171.6(3)
C54	C67	C72	N4	-10.8(5)
C54	C67	C72	C71	172.0(3)
C55	C54	C61	C62	114.3(2)
C55	C54	C61	C66	-62.7(2)
C55	C54	C67	C68	97.7(2)
C55	C54	C67	C72	-74.1(3)
C55	C56	C57	C58	-0.9(4)
C56	C55	C60	C59	-0.7(3)
C56	C57	C58	C59	-0.4(4)
C57	C58	C59	C60	1.1(4)
C58	C59	C60	C55	-0.6(4)
C60	C55	C56	C57	1.4(3)
C61	C54	C55	C56	-62.1(3)
C61	C54	C55	C60	116.4(2)
C61	C62	C63	C64	0.0
C62	C61	C66	C65	0.0
C62	C63	C64	C65	0.0
C63	C64	C65	C66	0.0
C64	C65	C66	C61	0.0
C66	C61	C62	N3	-179.4(3)
C66	C61	C62	C63	0.0

Atom	Atom	Atom	Atom	Angle/°
C67	C54	C55	C56	-39.0(3)
C67	C54	C55	C60	139.5(2)
C67	C68	C69	C70	0.0
C68	C67	C72	N4	177.2(4)
C68	C67	C72	C71	0.0
C68	C69	C70	C71	0.0
C69	C70	C71	C72	0.0
C70	C71	C72	N4	-177.4(4)
C70	C71	C72	C67	0.0
C72	C67	C68	C69	0.0

Table 6: Hydrogen Fractional Atomic Coordinates (×10[4]) and Equivalent Isotropic Displacement Parameters (Å[2]×10[3]) for **db-7-006.** U_{eq} is defined as 1/3 of the trace of the orthogonalised U_{ij}.

Atom	x	y	z	U_{eq}
H1	3280(40)	5854(9)	7590(8)	66(7)
H1A	2020(30)	6410(7)	7942(7)	46(5)
H1B	4150(30)	6686(8)	7838(7)	50(5)
H2A	6000(30)	6185(7)	8306(6)	44(5)
H2B	3970(30)	5912(7)	8405(7)	47(5)
H5	7820(40)	6867(8)	8467(7)	59(6)
H6	8740(40)	7620(9)	8344(9)	75(7)
H8	3170(40)	8068(9)	8708(8)	74(7)
H9	2060(30)	7330(7)	8819(7)	53(6)
H12	-540(40)	6656(8)	9487(8)	64(6)
H13	-1050(40)	7175(8)	10065(8)	63(6)
H14	1820(30)	7532(8)	10423(8)	60(6)
H15	5230(40)	7362(8)	10203(8)	65(6)
H16	5780(40)	6843(7)	9620(7)	55(6)
H19	1740(40)	5004(9)	9712(9)	71(7)
H20	-1270(40)	4862(9)	9245(8)	67(7)
H21	-2120(30)	5426(7)	8692(7)	51(6)
H22	-210(30)	6097(7)	8642(7)	48(5)
H4	9880(40)	5610(9)	7668(9)	74(7)
H23A	12470(40)	4882(9)	7686(9)	78(8)
H23B	10690(40)	4994(8)	7314(9)	65(7)
H24A	8040(30)	4882(7)	7861(7)	49(5)
H24B	9780(30)	4742(7)	8217(8)	55(6)
H27	11060(40)	3865(9)	7030(8)	68(7)
H28	9760(40)	3800(8)	6276(8)	68(7)
H30	4490(40)	4448(9)	6624(9)	81(8)
H31	5750(40)	4560(9)	7350(9)	70(7)
H34	12500(40)	3201(8)	7998(7)	57(6)
H35	12210(40)	2425(9)	7808(8)	70(7)
H36	8960(40)	2166(10)	7492(9)	77(7)
H37	6100(40)	2663(8)	7429(8)	66(7)
H38	6440(30)	3414(8)	7622(7)	54(6)
H41	13930(40)	4234(8)	9183(7)	59(6)
H42	11400(40)	3961(9)	9724(9)	78(7)
H43	7990(40)	3673(10)	9406(10)	87(8)
H44	7450(30)	3644(6)	8648(6)	33(4)
H7	6690(50)	5952(10)	7422(10)	96(10)
H45A	8310(40)	6545(10)	7006(9)	77(7)
H45B	10200(50)	6169(10)	6952(10)	89(8)
H46A	5850(40)	5990(8)	6681(8)	67(7)
H46B	7740(40)	5663(9)	6569(8)	68(7)
H49	9910(30)	6997(7)	5977(6)	43(5)
H50	8920(40)	7778(8)	5990(8)	60(6)

Atom	x	y	z	U_{eq}
H52	3130(30)	7440(7)	6438(7)	53(6)
H53	4100(30)	6662(7)	6418(6)	44(5)
H56	11810(40)	6153(9)	5293(9)	73(7)
H57	12180(50)	6532(10)	4630(10)	87(9)
H58	9400(40)	6905(9)	4278(9)	80(8)
H59	5990(40)	6865(10)	4628(10)	84(8)
H60	5810(40)	6499(8)	5303(8)	62(7)
H63	12417.61	4774.9	6015.89	66
H64	10291.32	4324.78	5547.24	74
H65	7309.4	4644.26	5199.09	72
H66	6453.75	5413.86	5319.57	64
H68	12201.28	5825.17	6224.69	71
H69	14133.58	5141.96	6206.29	83
H70	12768.12	4503.86	5829.49	81
H71	9470.34	4548.97	5471.09	75

Table 7: Hydrogen Bond information for **db-7-006**.

D	H	A	d(D-H)/Å	d(H-A)/Å	d(D-A)/Å	D-H-A/deg
O1	H1	O4[1]	0.84(3)	1.85(3)	2.656(2)	163(3)
O4	H4	O7	0.94(3)	1.71(3)	2.625(2)	162(2)
O7	H7	O1	0.92(3)	1.78(3)	2.669(2)	162(3)

----
[1]-1+x,+y,+z

Table 8: Atomic Occupancies for all atoms that are not fully occupied in **db-7-006**.

Atom	Occupancy	Atom	Occupancy	Atom	Occupancy	Atom	Occupancy
O8	0.508(2)	C62	0.508(2)	C66	0.508(2)	C70	0.492(2)
O9	0.508(2)	C63	0.508(2)	H66	0.508(2)	H70	0.492(2)
O10	0.492(2)	H63	0.508(2)	C67	0.492(2)	C71	0.492(2)
O11	0.492(2)	C64	0.508(2)	C68	0.492(2)	H71	0.492(2)
N3	0.508(2)	H64	0.508(2)	H68	0.492(2)	C72	0.492(2)
N4	0.492(2)	C65	0.508(2)	C69	0.492(2)		
C61	0.508(2)	H65	0.508(2)	H69	0.492(2)		

X-Ray crystallographic data for 4s

R_1=5.49%

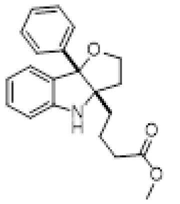

Solved by: **Rosario Scopelliti**

Crystal Data and Experimental

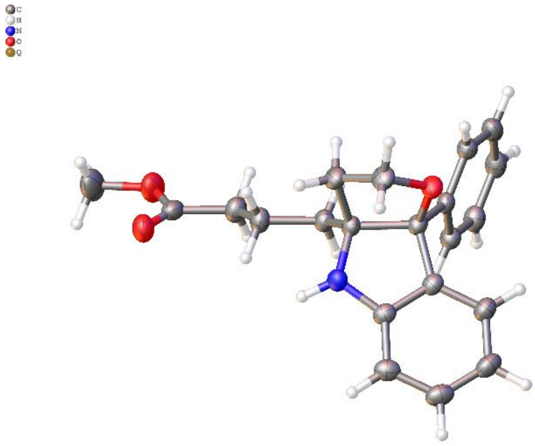

CCDC: 2523512
The ellipsoids are displayed at 50% probability level.

Experimental. Single clear light orange plate-shaped crystals of **db-7-118** were used as supplied. A suitable crystal with dimensions 0.21 × 0.18 × 0.08 mm was selected and mounted on a SuperNova, Dual, Cu at home/near, Atlas diffractometer. The crystal was kept at a steady T = 140.00(10) K during data collection. The structure was solved with the ShelXT (Sheldrick, 2015) solution program using dual methods and by using Olex2 1.5 (Dolomanov et al., 2009) as the graphical interface. The model was refined with ShelXL 2019/3 (Sheldrick, 2015) using full matrix least squares minimisation on $|F|^2$.

Crystal Data. $C_{21}H_{23}NO_3$, M_r = 337.40, monoclinic, $P2_1/c$ (No. 14), a = 9.3391(2) Å, b = 9.5034(3) Å, c = 20.1740(5) Å, β = 100.974(3)°, α = γ = 90°, V = 1757.76(8) Å^3, T = 140.00(10) K, Z = 4, Z' = 1, μ(Cu K_α) = 0.680, 9508 reflections measured, 3412 unique (R_{int} = 0.0329) which were used in all calculations. The final wR_2 was 0.1559 (all data) and R_1 was 0.0549 (I≥2σ(I)).

**Compound**	**db-7-118**
Formula	$C_{21}H_{23}NO_3$
$D_{calc.}$/ g cm^{-3}	1.275
μ/mm^{-1}	0.680
Formula Weight	337.40
Colour	clear light orange
Shape	plate-shaped
Size/mm	0.21×0.18×0.08
T/K	140.00(10)
Crystal System	monoclinic
Space Group	$P2_1/c$
a/Å	9.3391(2)
b/Å	9.5034(3)
c/Å	20.1740(5)
α/°	90
β/°	100.974(3)
γ/°	90
V/Å^3	1757.76(8)
Z	4
Z'	1
Wavelength/Å	1.54184
Radiation type	Cu K_α
Θ_{min}/°	4.465
Θ_{max}/°	72.542
Index range h	-10 ≤ h ≤ 11
Index range k	-10 ≤ k ≤ 11
Index range l	-23 ≤ l ≤ 24
Measured Refl's.	9508
Indep't Refl's	3412
Refl's I≥2σ(I)	2823
R_{int}	0.0329
Parameters	318
Restraints	0
Largest Peak/eÅ^3	0.422
Deepest Hole/eÅ^3	-0.269
GooF	1.073
R_1 (I≥2σ(I) / all)	0.0549 / 0.0646
wR_2 (I≥2σ(I) / all)	0.1434 / 0.1559

Structure Quality Indicators

**Reflections:**	d min (CuKα) 2Θ=145.1° 0.81	I/σ(I) 31.9	Rint m=2.92 3.29%	Full 135.4° 98% to 145.1° 99.9
**Refinement:**	Shift 0.000	Max Peak 0.4	Min Peak -0.3	GooF 1.073

A clear light orange plate-shaped crystal with dimensions 0.21 × 0.18 × 0.08 mm was mounted. Data were collected using a SuperNova, Dual, Cu at home/near, Atlas diffractometer operating at T = 140.00(10) K.

Data were measured using ω scans with Cu K_{α} radiation. The diffraction pattern was indexed and the total number of runs and images was based on the strategy calculation from the program CrysAlis^{Pro} 1.171.42.65a (Rigaku Oxford Diffraction, 2022). The maximum resolution achieved was Θ = 72.542° (0.81 Å).

The unit cell was refined using CrysAlis^{Pro} on 4668 reflections, 49% of the observed reflections.

Data reduction, scaling and absorption corrections were performed using CrysAlis^{Pro}. The final completeness is 99.90 % out to 72.542° in Θ. An analytical absorption correction was performed using CrysAlis^{Pro}. The absorption coefficient μ of this crystal is 0.680 mm^{-1} at this wavelength (λ = 1.54184Å) and the minimum and maximum transmissions are 0.907 and 0.961.

The structure was solved in the space group $P2_1/c$ (# 14) by ShelXT (Sheldrick, 2015) using dual methods. It was refined by full matrix least squares minimisation on $|F|^2$ using version 2019/3 of ShelXL 2019/3 (Sheldrick, 2015). All non-hydrogen atoms were refined anisotropically.

All hydrogen atoms were freely refined.

There is a single formula unit in the asymmetric unit, which is represented by the reported sum formula. In other words: Z is 4 and Z' is 1. The moiety formula is C21 H23 N O3.

Data Plots: Diffraction Data

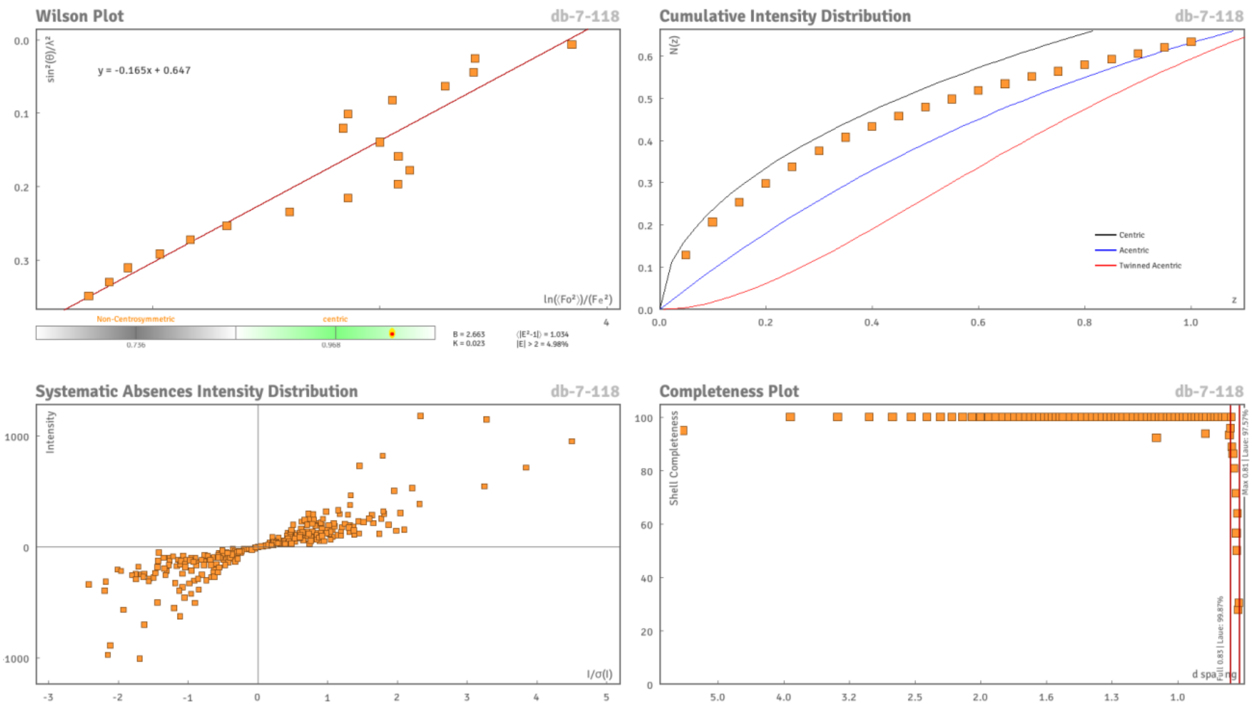

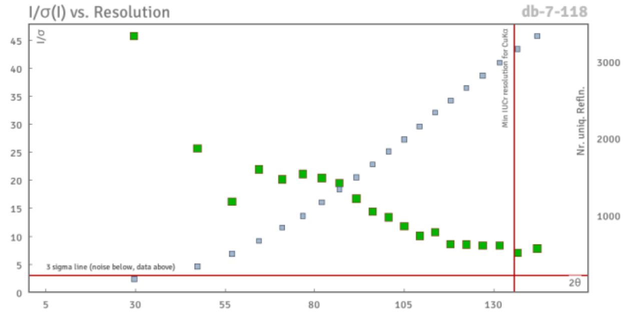

Data Plots: Refinement and Data

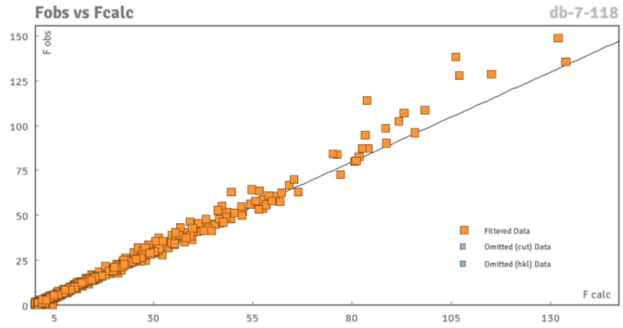

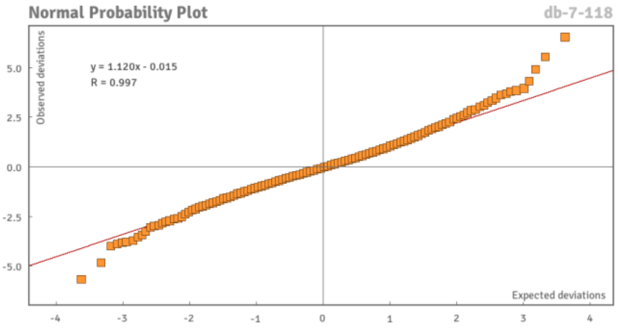

Reflection Statistics

Total reflections (after filtering)	9957	Unique reflections	3412
Completeness	0.976	Mean I/σ	16.01
hkl_{max} collected	(11, 11, 24)	hkl_{min} collected	(-10, -10, -23)
hkl_{max} used	(11, 11, 24)	hkl_{min} used	(-11, 0, 0)
Lim d_{max} collected	100.0	Lim d_{min} collected	0.77
d_{max} used	9.9	d_{min} used	0.81
Friedel pairs	1026	Friedel pairs merged	1
Inconsistent equivalents	3	R_{int}	0.0329
R_{sigma}	0.0313	Intensity transformed	0
Omitted reflections	0	Omitted by user (OMIT hkl)	0
Multiplicity	(4419, 1516, 513, 148, 63, 10)	Maximum multiplicity	11
Removed systematic absences	449	Filtered off (Shel/OMIT)	0

Table 9: Fractional Atomic Coordinates (×10[4]) and Equivalent Isotropic Displacement Parameters (Å^2×10^3) for **db-7-118**. U_{eq} is defined as 1/3 of the trace of the orthogonalised U_{ij}.

Atom	x	y	z	U_{eq}
O1	8024.3(12)	10717.0(13)	3529.9(6)	30.3(3)
O2	8381.0(16)	3230.0(16)	4831.4(7)	45.4(4)
O3	6100.6(17)	2882.3(16)	4979.5(8)	50.9(4)
N1	9125.4(15)	7633.4(17)	3232.6(7)	31.3(3)
C1	8030.3(17)	8174.5(18)	3607.5(8)	27.0(4)
C2	8807(2)	8870(2)	4264.7(8)	33.9(4)
C3	9252(2)	10271(2)	4024.4(9)	34.4(4)
C4	7361.8(17)	9496.3(17)	3168.8(8)	26.0(4)
C5	7924.6(17)	9305.3(18)	2520.8(8)	27.2(4)
C6	7603(2)	10068(2)	1926.2(8)	33.8(4)
C7	8313(2)	9716(2)	1396.8(9)	39.8(4)
C8	9314(2)	8612(2)	1472.3(9)	37.4(4)
C9	9643.4(18)	7842(2)	2066.4(9)	32.3(4)
C10	8932.1(17)	8212.6(18)	2591.9(8)	27.7(4)

Atom	x	y	z	U_{eq}
C11	6943.2(18)	7043.8(19)	3715.7(8)	30.0(4)
C12	7630(2)	5825(2)	4159.0(9)	34.3(4)
C13	6482(2)	4801(2)	4300.1(10)	37.5(4)
C14	7128(2)	3573(2)	4725.4(8)	34.7(4)
C15	6617(4)	1698(3)	5405.1(15)	62.9(7)
C16	5721.6(17)	9663.8(17)	3070.8(8)	25.5(3)
C17	5075.8(19)	10625.5(19)	3442.7(8)	30.4(4)
C18	3565(2)	10744(2)	3346.6(9)	35.8(4)
C19	2683.5(19)	9886(2)	2887.1(9)	35.8(4)
C20	3312.6(19)	8915(2)	2516.8(9)	33.7(4)
C21	4818.4(18)	8814.0(19)	2598.6(8)	29.7(4)

Table 10: Anisotropic Displacement Parameters (×10^4) for **db-7-118**. The anisotropic displacement factor exponent takes the form: $-2\pi^2[h^2a^{*2} \times U_{11} + ... + 2hka^* \times b^* \times U_{12}]$

Atom	U_{11}	U_{22}	U_{33}	U_{23}	U_{13}	U_{12}
O1	30.0(6)	29.6(6)	28.0(6)	-1.8(5)	-2.8(5)	-0.9(5)
O2	49.8(8)	40.9(8)	45.5(8)	10.5(6)	8.7(6)	10.6(6)
O3	61.2(9)	41.0(8)	56.3(9)	18.2(7)	25.8(7)	6.7(7)
N1	29.6(7)	34.5(8)	29.9(7)	3.3(6)	5.8(5)	7.2(6)
C1	25.8(7)	30.6(8)	23.4(7)	1.4(6)	1.6(6)	4.5(6)
C2	36.4(9)	37.2(10)	25.0(8)	-0.1(7)	-2.1(7)	4.0(8)
C3	31.3(9)	38.9(10)	28.4(8)	-2.7(7)	-6.1(7)	-0.4(7)
C4	26.8(8)	26.3(8)	23.3(7)	0.3(6)	0.8(6)	-0.7(6)
C5	24.1(7)	31.7(9)	24.7(8)	0.5(6)	1.7(6)	-3.2(6)
C6	36.2(9)	37.0(10)	27.4(8)	3.5(7)	3.8(7)	-0.8(8)
C7	47.3(10)	46.5(11)	26.1(8)	3.4(7)	8.3(7)	-6.9(8)
C8	37.9(9)	45.1(11)	31.8(9)	-5.1(7)	13.1(7)	-10.2(8)
C9	26.5(8)	37.1(10)	34.4(8)	-6.4(7)	8.4(6)	-5.4(7)
C10	23.9(7)	32.7(9)	26.0(7)	-2.0(6)	3.7(6)	-4.7(6)
C11	28.6(8)	31.7(9)	29.1(8)	3.4(7)	4.3(6)	3.5(7)
C12	34.5(9)	33.8(9)	33.8(9)	8.0(7)	4.3(7)	3.9(7)
C13	37.8(9)	39.0(10)	35.8(9)	8.4(8)	7.4(8)	2.9(8)
C14	47.4(10)	31.4(9)	26.4(8)	-0.3(7)	10.2(7)	2.2(8)
C15	84.1(19)	44.2(13)	68.8(16)	25.0(12)	36.2(15)	12.8(13)
C16	26.4(8)	25.2(8)	24.2(7)	4.4(6)	2.7(6)	2.4(6)
C17	32.8(9)	30.1(9)	28.0(8)	1.7(7)	4.7(7)	2.7(7)
C18	37.5(9)	34.7(10)	37.6(9)	6.2(7)	13.1(8)	9.5(7)
C19	26.0(8)	40.1(10)	41.3(9)	11.1(8)	6.4(7)	3.5(7)
C20	28.2(8)	35.3(10)	35.3(9)	4.8(7)	0.0(7)	-1.9(7)
C21	28.8(8)	30.2(9)	28.7(8)	0.8(6)	2.3(6)	1.2(7)

Table 11: Bond Lengths in Å for **db-7-118**.

Atom	Atom	Length/Å
O1	C3	1.433(2)
O1	C4	1.4448(19)
O2	C14	1.195(2)
O3	C14	1.342(2)
O3	C15	1.442(3)
N1	C1	1.475(2)
N1	C10	1.385(2)
C1	C2	1.535(2)
C1	C4	1.594(2)
C1	C11	1.522(2)

Atom	Atom	Length/Å
C2	C3	1.503(3)
C4	C5	1.510(2)
C4	C16	1.515(2)
C5	C6	1.385(2)
C5	C10	1.390(2)
C6	C7	1.401(3)
C7	C8	1.395(3)
C8	C9	1.388(3)
C9	C10	1.399(2)
C11	C12	1.528(2)

Atom	Atom	Length/Å
C12	C13	1.514(3)
C13	C14	1.505(3)
C16	C17	1.391(2)
C16	C21	1.402(2)

Atom	Atom	Length/Å
C17	C18	1.392(3)
C18	C19	1.383(3)
C19	C20	1.386(3)
C20	C21	1.388(2)

Table 12: Bond Angles in ° for **db-7-118**.

Atom	Atom	Atom	Angle/°
C3	O1	C4	108.83(12)
C14	O3	C15	115.28(19)
C10	N1	C1	111.10(13)
N1	C1	C2	109.45(13)
N1	C1	C4	103.09(12)
N1	C1	C11	111.95(14)
C2	C1	C4	102.20(13)
C11	C1	C2	113.49(14)
C11	C1	C4	115.75(13)
C3	C2	C1	102.54(13)
O1	C3	C2	104.64(14)
O1	C4	C1	105.55(12)
O1	C4	C5	110.29(13)
O1	C4	C16	107.86(13)
C5	C4	C1	102.82(13)
C5	C4	C16	114.17(13)
C16	C4	C1	115.76(13)
C6	C5	C4	129.30(16)
C6	C5	C10	120.75(16)
C10	C5	C4	109.92(14)
C5	C6	C7	118.66(18)

Atom	Atom	Atom	Angle/°
C8	C7	C6	120.08(17)
C9	C8	C7	121.67(17)
C8	C9	C10	117.55(17)
N1	C10	C5	111.02(14)
N1	C10	C9	127.65(16)
C5	C10	C9	121.30(16)
C1	C11	C12	113.69(14)
C13	C12	C11	111.33(15)
C14	C13	C12	112.62(16)
O2	C14	O3	123.04(17)
O2	C14	C13	125.89(18)
O3	C14	C13	111.07(16)
C17	C16	C4	122.01(15)
C17	C16	C21	118.59(15)
C21	C16	C4	119.39(15)
C16	C17	C18	120.68(16)
C19	C18	C17	120.26(17)
C18	C19	C20	119.66(16)
C19	C20	C21	120.38(17)
C20	C21	C16	120.40(16)

Table 13: Torsion Angles in ° for **db-7-118**.

Atom	Atom	Atom	Atom	Angle/°
O1	C4	C5	C6	-73.9(2)
O1	C4	C5	C10	103.99(15)
O1	C4	C16	C17	-16.4(2)
O1	C4	C16	C21	164.49(14)
N1	C1	C2	C3	78.10(17)
N1	C1	C4	O1	-102.65(14)
N1	C1	C4	C5	12.97(15)
N1	C1	C4	C16	138.16(14)
N1	C1	C11	C12	64.24(18)
C1	N1	C10	C5	9.83(19)
C1	N1	C10	C9	-172.14(16)
C1	C2	C3	O1	40.95(17)
C1	C4	C5	C6	173.98(17)
C1	C4	C5	C10	-8.17(17)
C1	C4	C16	C17	101.50(18)
C1	C4	C16	C21	-77.59(18)
C1	C11	C12	C13	174.94(15)
C2	C1	C4	O1	10.93(16)
C2	C1	C4	C5	126.55(13)
C2	C1	C4	C16	-108.26(15)
C2	C1	C11	C12	-60.3(2)
C3	O1	C4	C1	14.51(17)
C3	O1	C4	C5	-95.88(15)

Atom	Atom	Atom	Atom	Angle/°
C3	O1	C4	C16	138.82(14)
C4	O1	C3	C2	-35.15(18)
C4	C1	C2	C3	-30.69(16)
C4	C1	C11	C12	-178.02(14)
C4	C5	C6	C7	177.69(17)
C4	C5	C10	N1	-0.35(19)
C4	C5	C10	C9	-178.53(14)
C4	C16	C17	C18	-179.26(15)
C4	C16	C21	C20	177.77(15)
C5	C4	C16	C17	-139.38(16)
C5	C4	C16	C21	41.5(2)
C5	C6	C7	C8	0.4(3)
C6	C5	C10	N1	177.71(15)
C6	C5	C10	C9	-0.5(3)
C6	C7	C8	C9	-0.4(3)
C7	C8	C9	C10	0.0(3)
C8	C9	C10	N1	-177.42(16)
C8	C9	C10	C5	0.4(2)
C10	N1	C1	C2	-122.43(15)
C10	N1	C1	C4	-14.23(17)
C10	N1	C1	C11	110.84(15)
C10	C5	C6	C7	0.0(3)
C11	C1	C2	C3	-156.05(14)
C11	C1	C4	O1	134.79(14)
C11	C1	C4	C5	-109.59(15)
C11	C1	C4	C16	15.6(2)
C11	C12	C13	C14	178.94(15)
C12	C13	C14	O2	-15.4(3)
C12	C13	C14	O3	164.70(16)
C15	O3	C14	O2	1.1(3)
C15	O3	C14	C13	-179.0(2)
C16	C4	C5	C6	47.8(2)
C16	C4	C5	C10	-134.38(15)
C16	C17	C18	C19	1.2(3)
C17	C16	C21	C20	-1.3(2)
C17	C18	C19	C20	-0.7(3)
C18	C19	C20	C21	-0.9(3)
C19	C20	C21	C16	1.9(3)
C21	C16	C17	C18	-0.2(2)

Table 14: Hydrogen Fractional Atomic Coordinates (×10[4]) and Equivalent Isotropic Displacement Parameters (Å[2]×10[3]) for **db-7-118**. U_{eq} is defined as 1/3 of the trace of the orthogonalised U_{ij}.

Atom	x	y	z	U_{eq}
H1	9480(20)	6720(30)	3306(11)	32(5)
H2A	8050(20)	9010(20)	4554(11)	36(5)
H2B	9630(20)	8310(20)	4474(11)	32(5)
H3A	10140(30)	10210(20)	3821(12)	39(6)
H3B	9410(30)	11010(30)	4373(13)	46(6)
H6	6880(20)	10830(30)	1860(11)	38(6)
H7	8150(30)	10270(30)	968(13)	51(7)
H8	9790(30)	8350(30)	1099(12)	46(6)
H9	10350(20)	7030(20)	2110(10)	30(5)
H11A	6180(20)	7490(20)	3912(10)	28(5)
H11B	6440(20)	6640(20)	3268(12)	35(5)
H12A	8350(30)	5310(30)	3925(13)	50(7)
H12B	8220(20)	6180(20)	4579(11)	34(5)
H13A	5740(30)	5280(30)	4528(14)	57(7)

Atom	x	y	z	U_{eq}
H13B	5880(30)	4410(30)	3901(14)	52(7)
H15A	7120(30)	1020(40)	5144(16)	69(8)
H15B	5730(40)	1300(40)	5507(17)	79(10)
H15C	7370(40)	2110(40)	5779(19)	88(11)
H17	5700(20)	11180(20)	3737(11)	30(5)
H18	3140(20)	11450(30)	3602(12)	42(6)
H19	1640(20)	9990(20)	2818(11)	32(5)
H20	2720(30)	8320(30)	2201(13)	45(6)
H21	5260(20)	8090(20)	2349(11)	33(5)

Table 15: Hydrogen Bond information for **db-7-118**.

D	H	A	d(D-H)/Å	d(H-A)/Å	d(D-A)/Å	D-H-A/deg
C2	H2B	$O2^{1}$	0.97(2)	2.56(2)	3.518(2)	171.8(17)
C3	H3B	$O2^{2}$	0.98(3)	2.57(3)	3.424(3)	145.7(19)
C9	H9	$O1^{3}$	1.01(2)	2.51(2)	3.357(2)	141.8(16)

––––

^{1}2-x,1-y,1-z; ^{2}+x,1+y,+z; ^{3}2-x,-1/2+y,1/2-z

Citations

CrysAlis^{Pro} (Rigaku Oxford Diffraction), Rigaku Oxford Diffraction, Poland (2022).

O.V. Dolomanov and L.J. Bourhis and R.J. Gildea and J.A.K. Howard and H. Puschmann, Olex2: A complete structure solution, refinement and analysis program, *J. Appl. Cryst.*, (2009), **42**, 339-341.

Sheldrick, G.M., Crystal structure refinement with ShelXL, *Acta Cryst.*, (2015), **C71**, 3-8.

Sheldrick, G.M., ShelXT-Integrated space-group and crystal-structure determination, *Acta Cryst.*, (2015), **A71**, 3-8.

6) Copies of NMR spectra

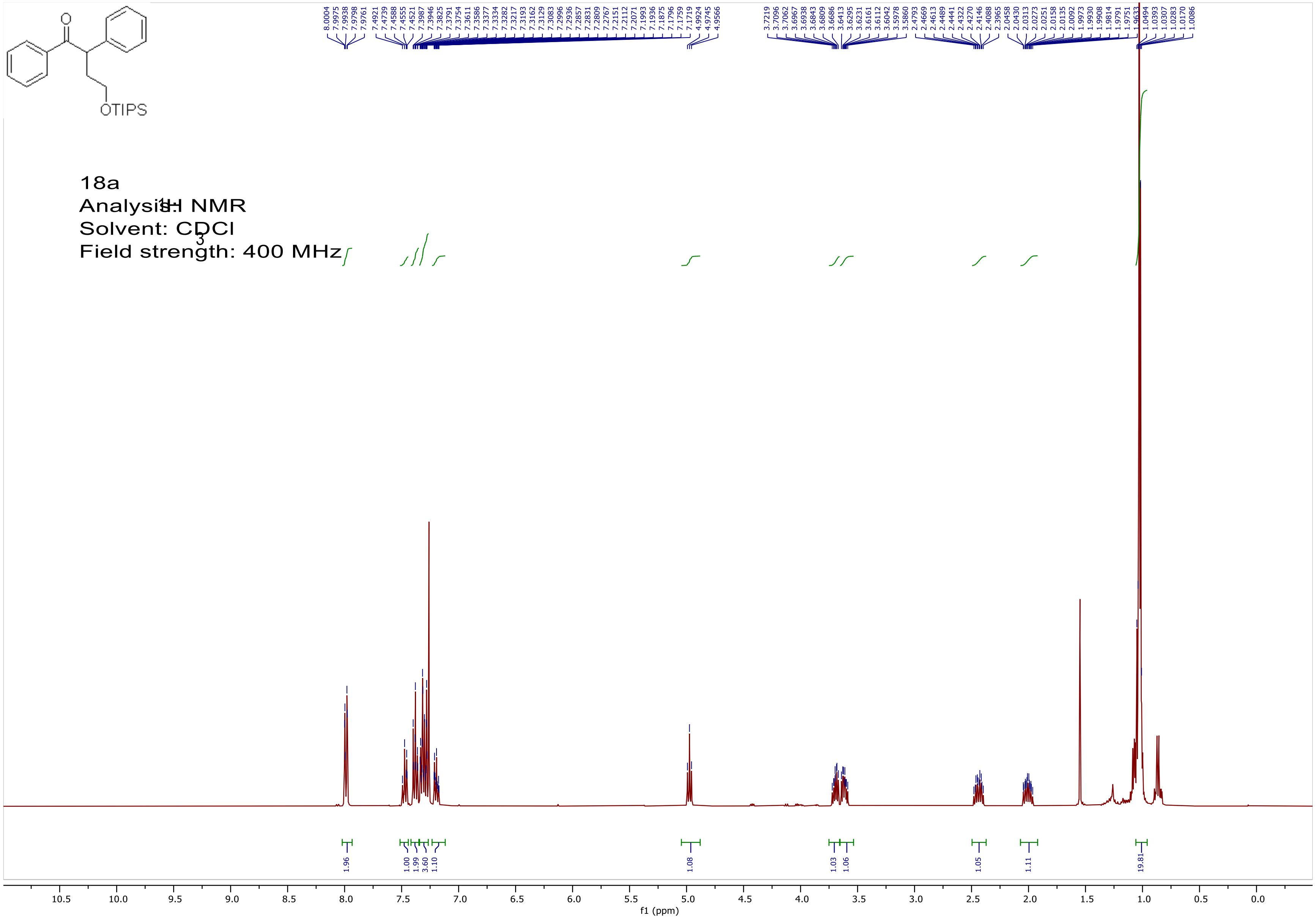
OTIPS
18a
Analysis: 1H NMR
Solvent: CDCl3
Field strength: 400 MHz
8.0004 7.9975 7.9938 7.9798 7.9761 7.4921 7.4739 7.4588 7.4555 7.4521 7.3987 7.3946 7.3825 7.3791 7.3754 7.3611 7.3586 7.3377 7.3334 7.3282 7.3217 7.3193 7.3162 7.3129 7.3083 7.2996 7.2936 7.2857 7.2831 7.2809 7.2767 7.2151 7.2112 7.2071 7.1993 7.1936 7.1875 7.1796 7.1759 7.1719 4.9924 4.9745 4.9566
3.7219 3.7096 3.7062 3.6967 3.6938 3.6843 3.6809 3.6686 3.6413 3.6295 3.6231 3.6161 3.6112 3.6042 3.5978 3.5860 2.4793 2.4669 2.4613 2.4489 2.4441 2.4322 2.4270 2.4146 2.4088 2.3965 2.0458 2.0430 2.0313 2.0273 2.0251 2.0158 2.0135 2.0092 1.9973 1.9930 1.9908 1.9814 1.9791 1.9751 1.0633 1.0494 1.0393 1.0307 1.0283 1.0170 1.0086
1.96 1.00 1.99 3.60 1.10 1.08 1.03 1.06 1.05 1.11 19.81
10.5 10.0 9.5 9.0 8.5 8.0 7.5 7.0 6.5 6.0 5.5 5.0 4.5 4.0 3.5 3.0 2.5 2.0 1.5 1.0 0.5 0.0
f1 (ppm)

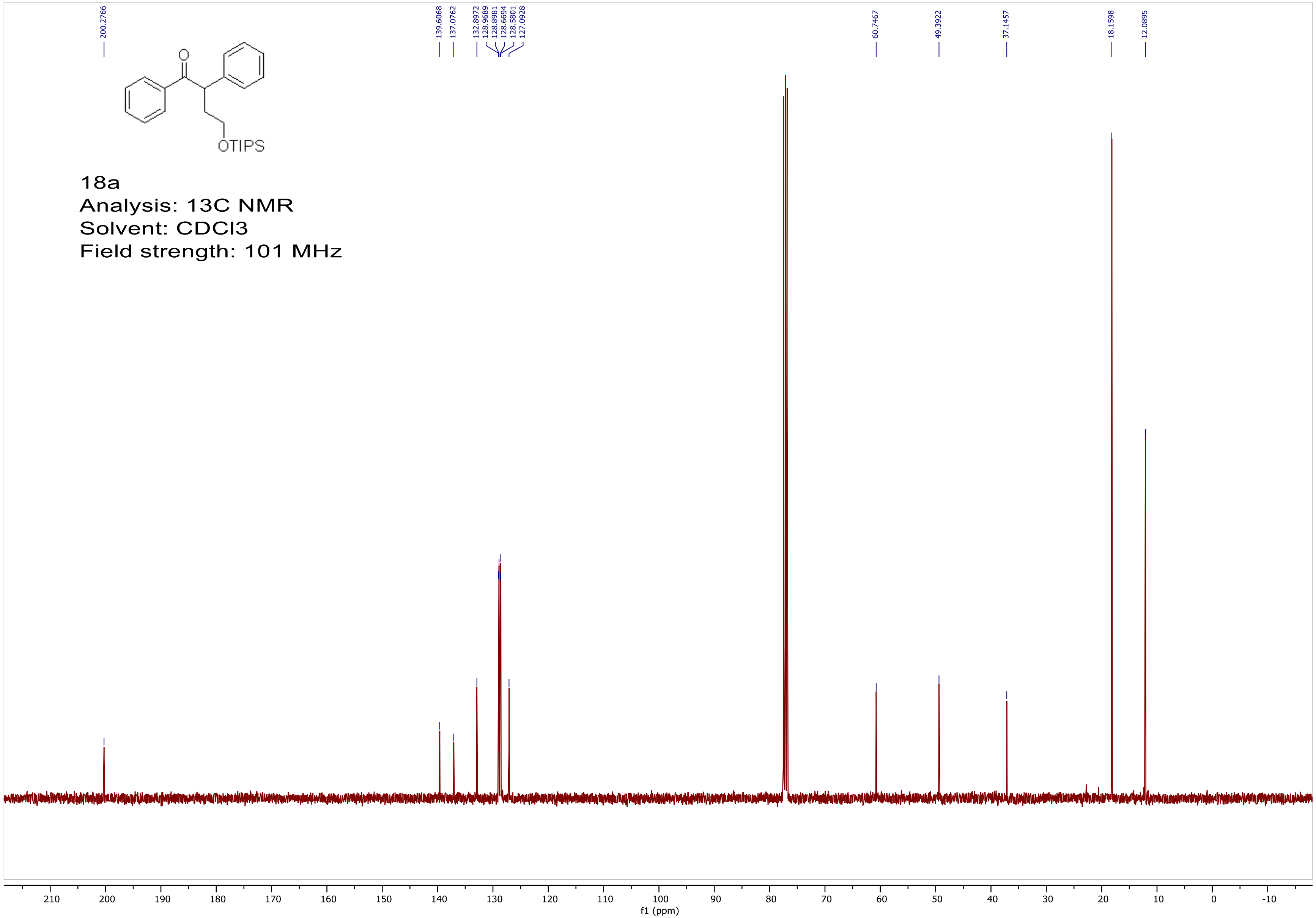

18a
Analysis: 13C NMR
Solvent: CDCl3
Field strength: 101 MHz

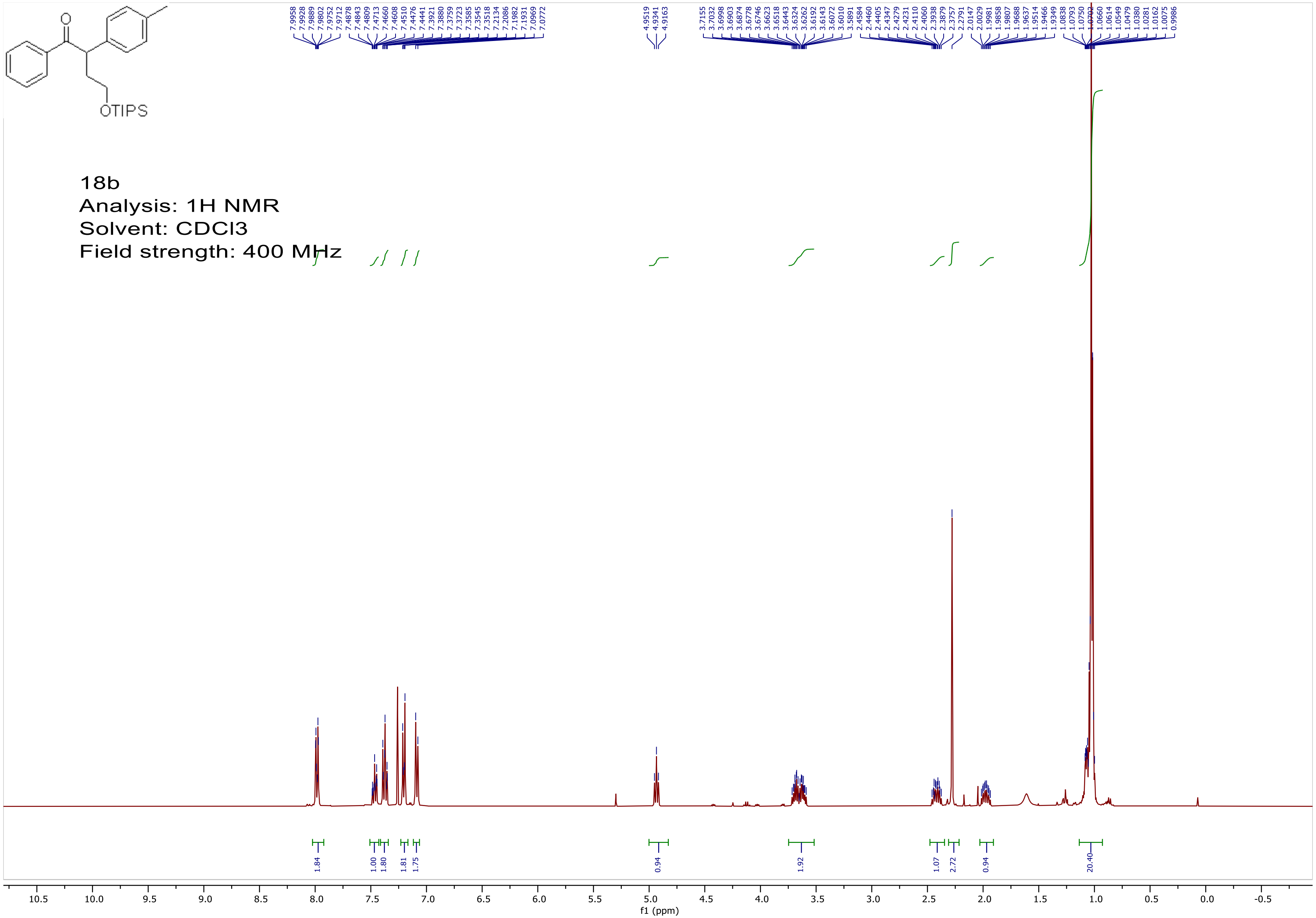

OTIPS
18b
Analysis: 1H NMR
Solvent: CDCl3
Field strength: 400 MHz
f1 (ppm)

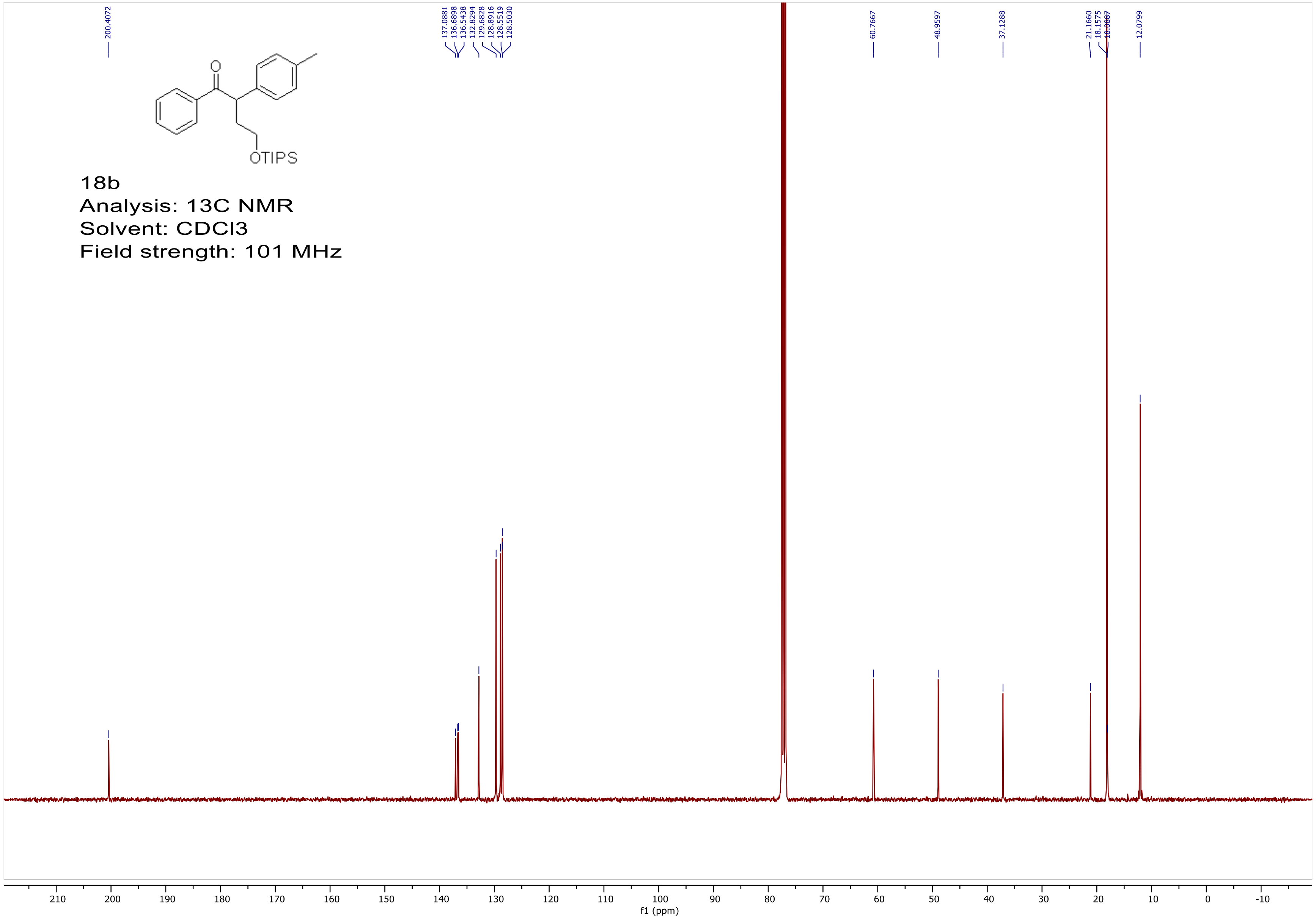

OTIPS
18b
Analysis: 13C NMR
Solvent: CDCl3
Field strength: 101 MHz
200.4072
137.0881
136.6898
136.5438
132.8294
129.6828
128.8916
128.5519
128.5030
60.7667
48.9597
37.1288
21.1660
18.1575
12.0799
210
200
190
180
170
160
150
140
130
120
110
100
90
80
70
60
50
40
30
20
10
0
-10
f1 (ppm)

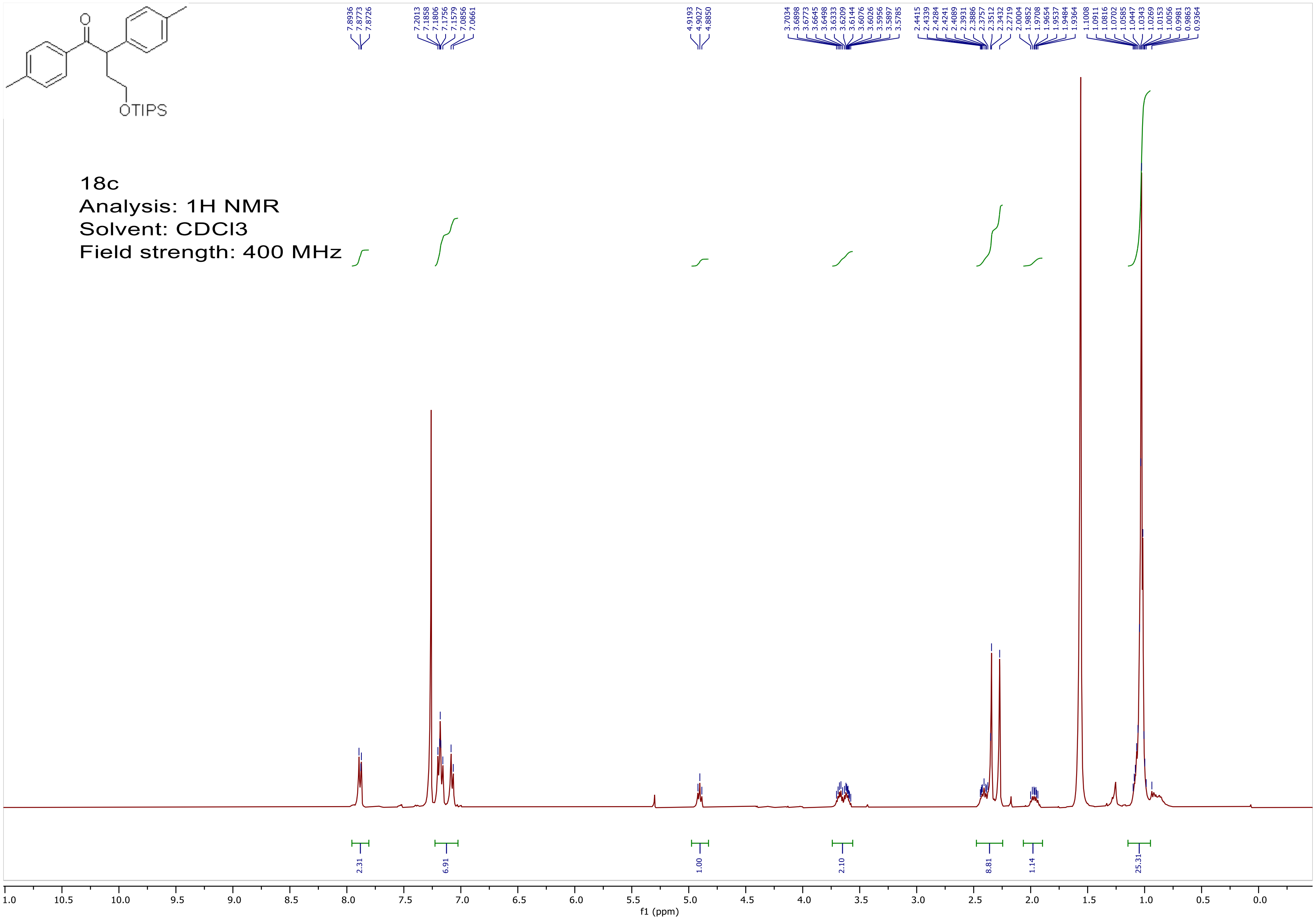
OTIPS
18c
Analysis: 1H NMR
Solvent: CDCl3
Field strength: 400 MHz
2.31
6.91
1.00
2.10
8.81
1.14
25.31
f1 (ppm)

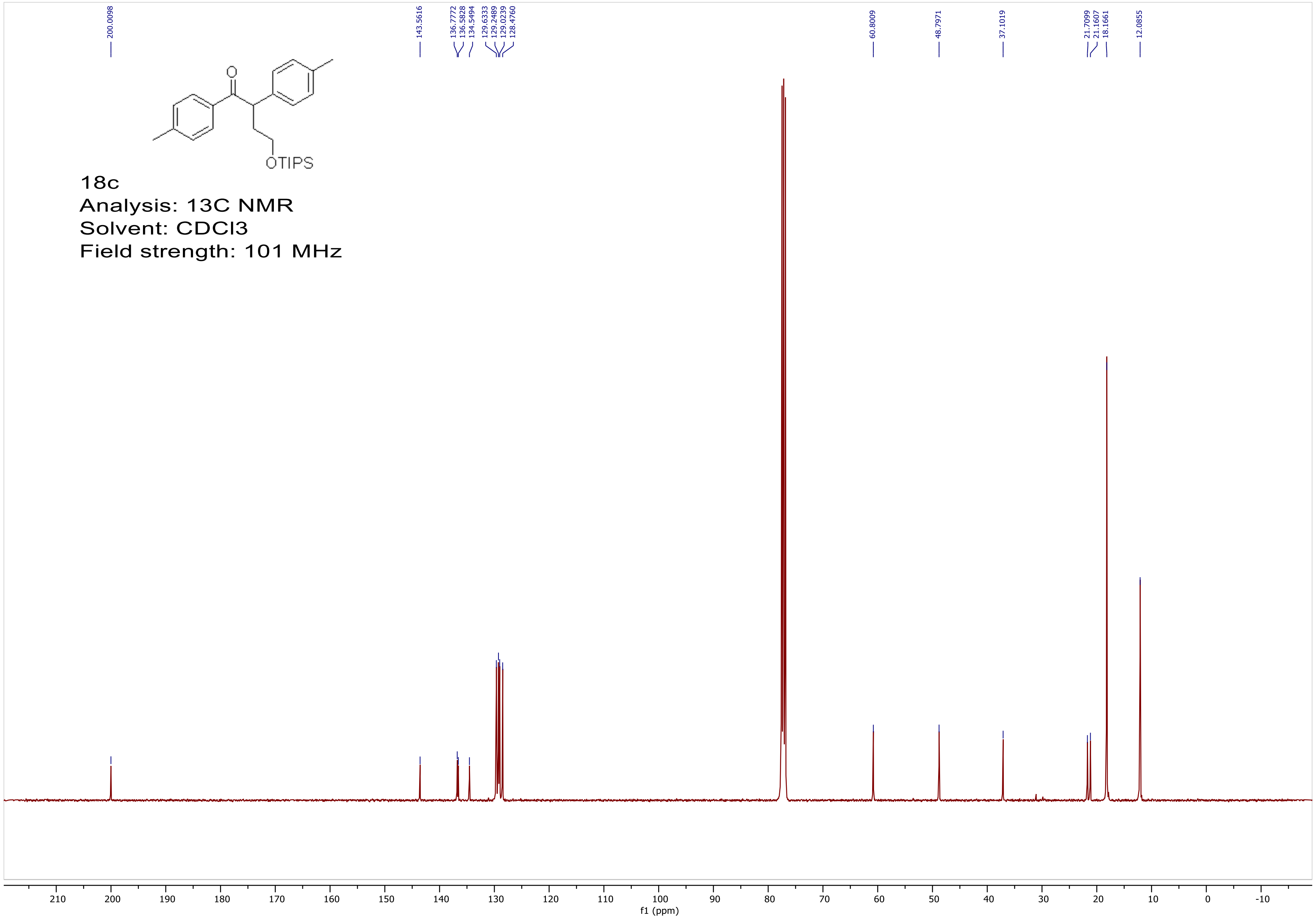
200.0098
143.5616
136.7772
136.5828
134.5494
129.6333
129.2489
129.0239
128.4760
60.8009
48.7971
37.1019
21.7099
21.1607
18.1661
12.0855
O
OTIPS
18c
Analysis: 13C NMR
Solvent: CDCl3
Field strength: 101 MHz
210
200
190
180
170
160
150
140
130
120
110
100
90
80
70
60
50
40
30
20
10
0
-10
f1 (ppm)

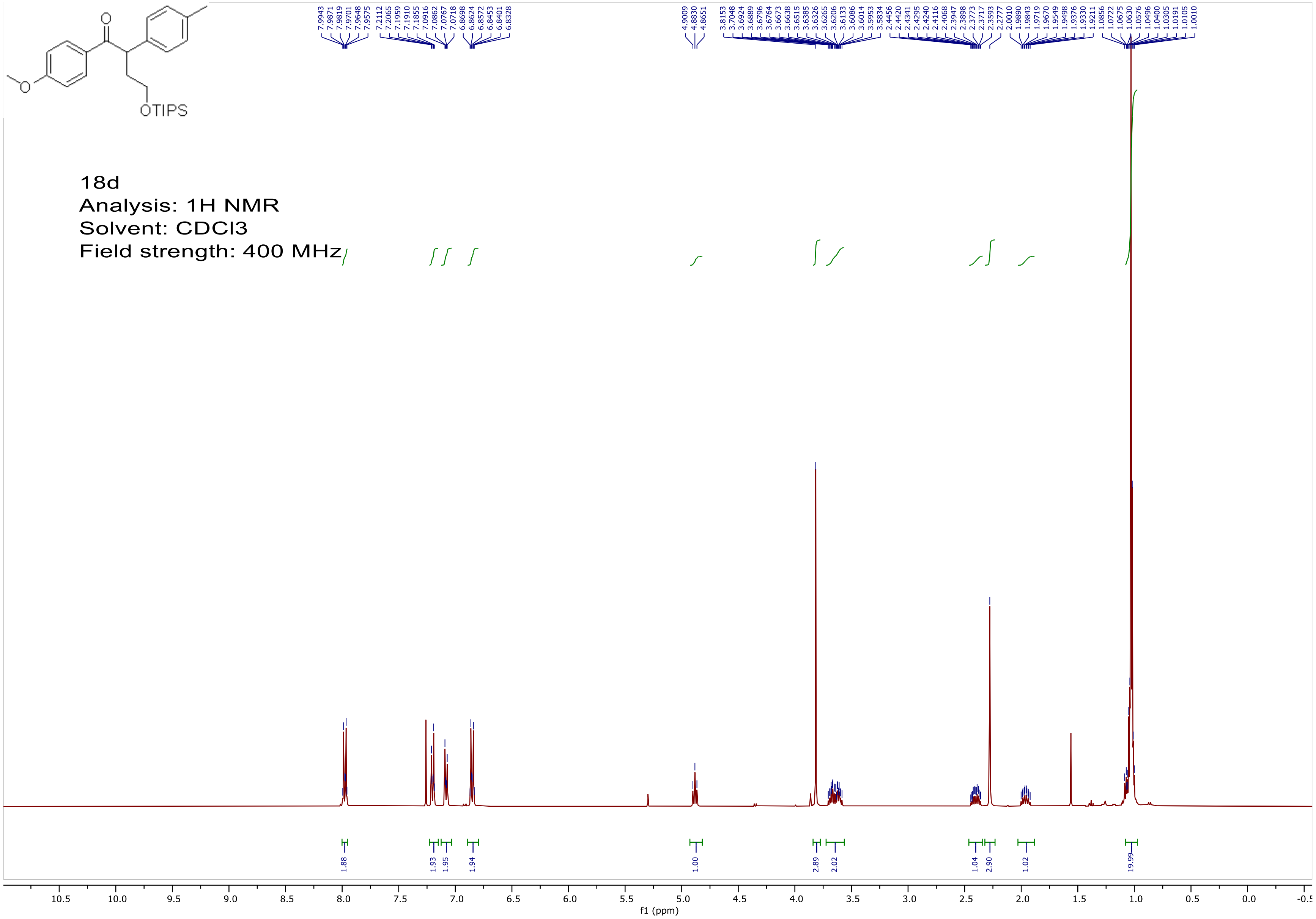
OTIPS
18d
Analysis: 1H NMR
Solvent: CDCl3
Field strength: 400 MHz
7.9943
7.9871
7.9819
7.9701
7.9648
7.9575
7.2112
7.2065
7.1959
7.1910
7.1855
7.0916
7.0862
7.0767
7.0718
6.8698
6.8624
6.8572
6.8453
6.8401
6.8328
4.9009
4.8830
4.8651
3.8153
3.7048
3.6924
3.6889
3.6796
3.6764
3.6673
3.6638
3.6515
3.6385
3.6326
3.6265
3.6206
3.6133
3.6086
3.6014
3.5953
3.5834
2.4456
2.4420
2.4341
2.4295
2.4240
2.4116
2.4068
2.3947
2.3898
2.3773
2.3717
2.3593
2.2777
2.0010
1.9890
1.9843
1.9719
1.9670
1.9549
1.9498
1.9376
1.9330
1.9211
1.0856
1.0722
1.0675
1.0630
1.0576
1.0496
1.0400
1.0305
1.0191
1.0105
1.0010
1.88
1.93
1.95
1.94
1.00
2.89
2.02
1.04
2.90
1.02
19.99
10.5
10.0
9.5
9.0
8.5
8.0
7.5
7.0
6.5
6.0
5.5
5.0
4.5
4.0
3.5
3.0
2.5
2.0
1.5
1.0
0.5
0.0
-0.5
f1 (ppm)

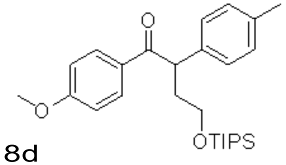

18d
Analysis: 13C NMR
Solvent: CDCl3
Field strength: 101 MHz

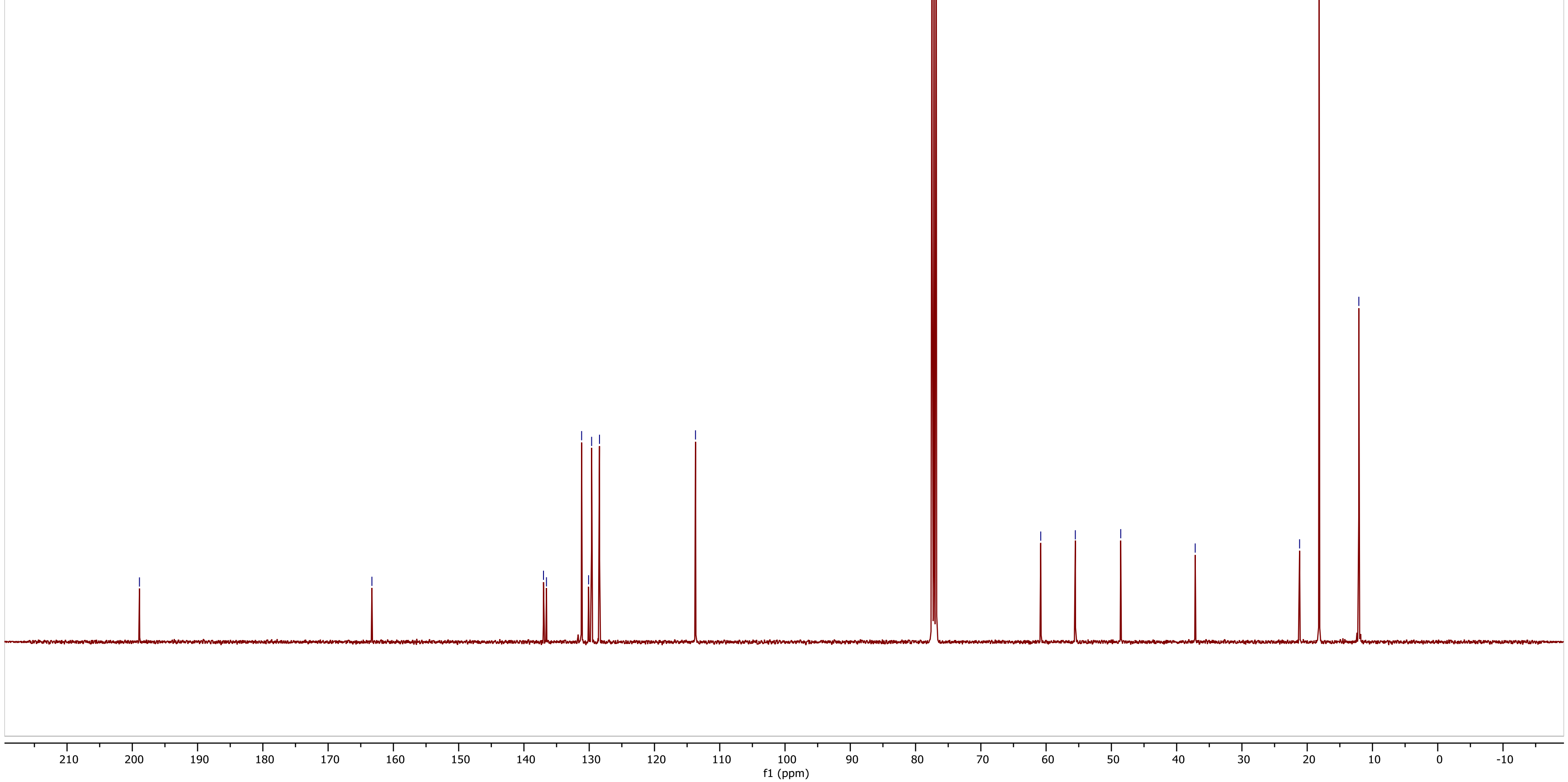

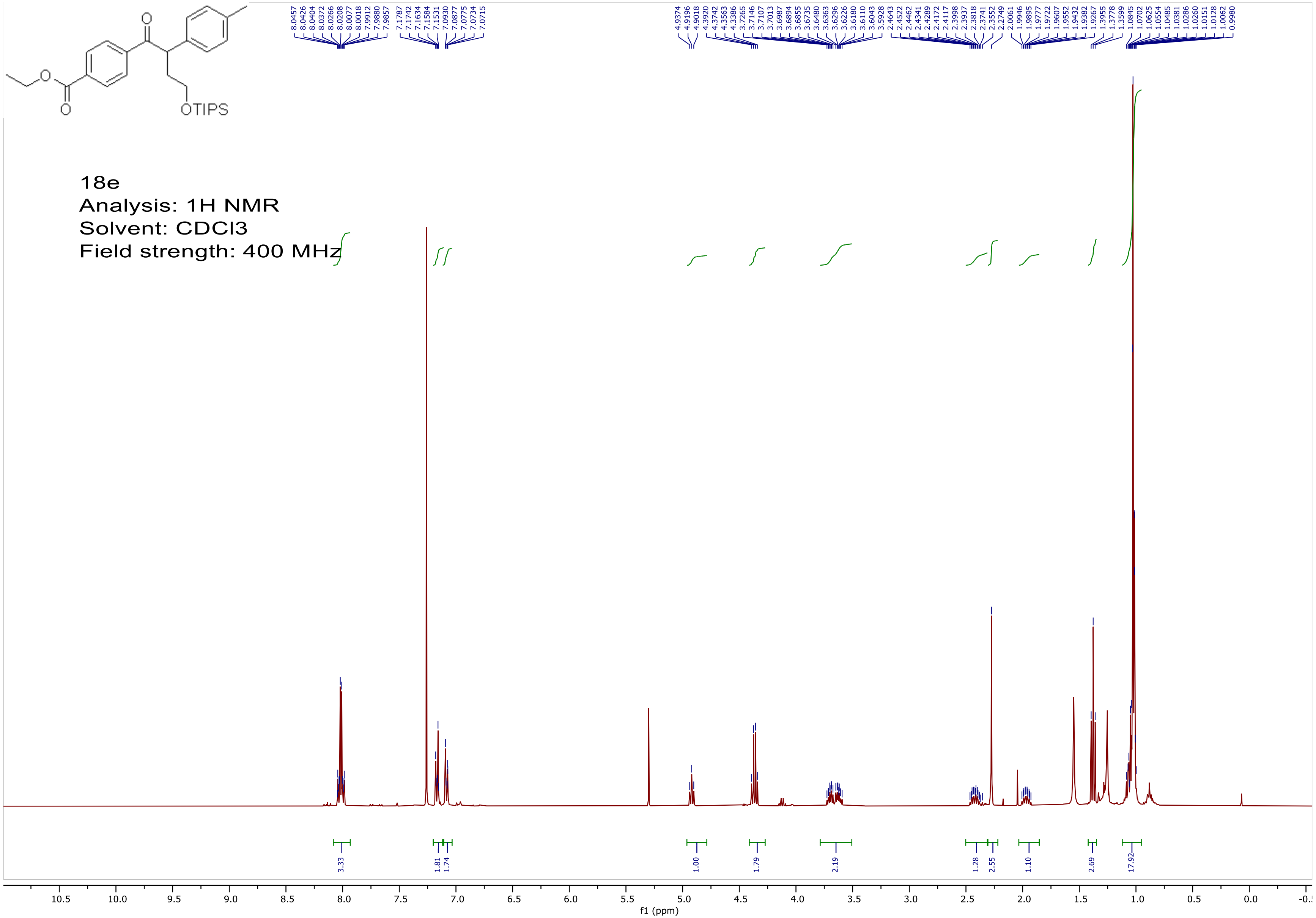
18e
Analysis: 1H NMR
Solvent: CDCl3
Field strength: 400 MHz
OTIPS
f1 (ppm)

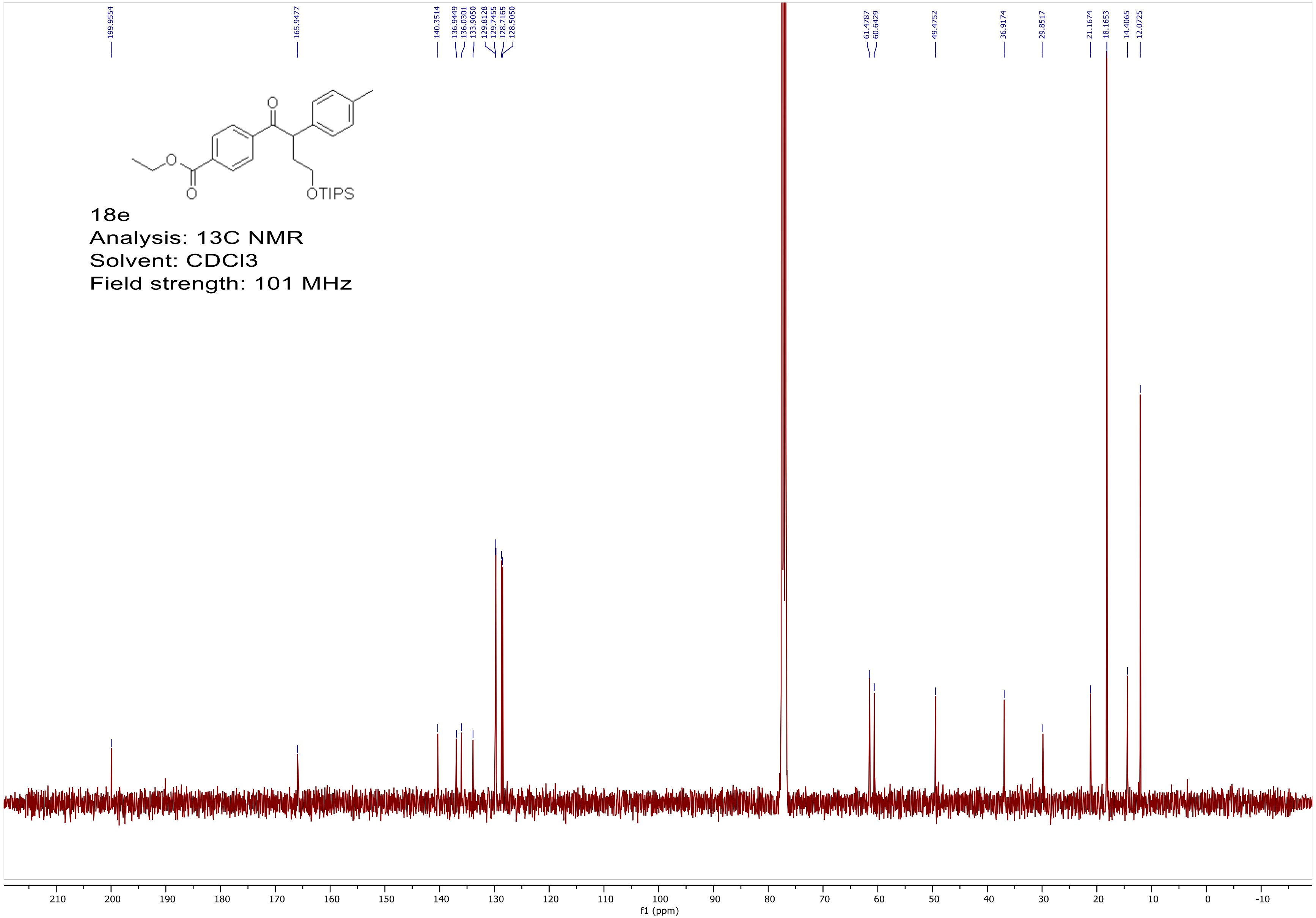
199.9554
165.9477
140.3514
136.9449
136.0301
133.9050
129.8128
129.7455
128.7165
128.5050
61.4787
60.6429
49.4752
36.9174
29.8517
21.1674
18.1653
14.4065
12.0725
OTIPS
18e
Analysis: 13C NMR
Solvent: CDCl3
Field strength: 101 MHz
f1 (ppm)

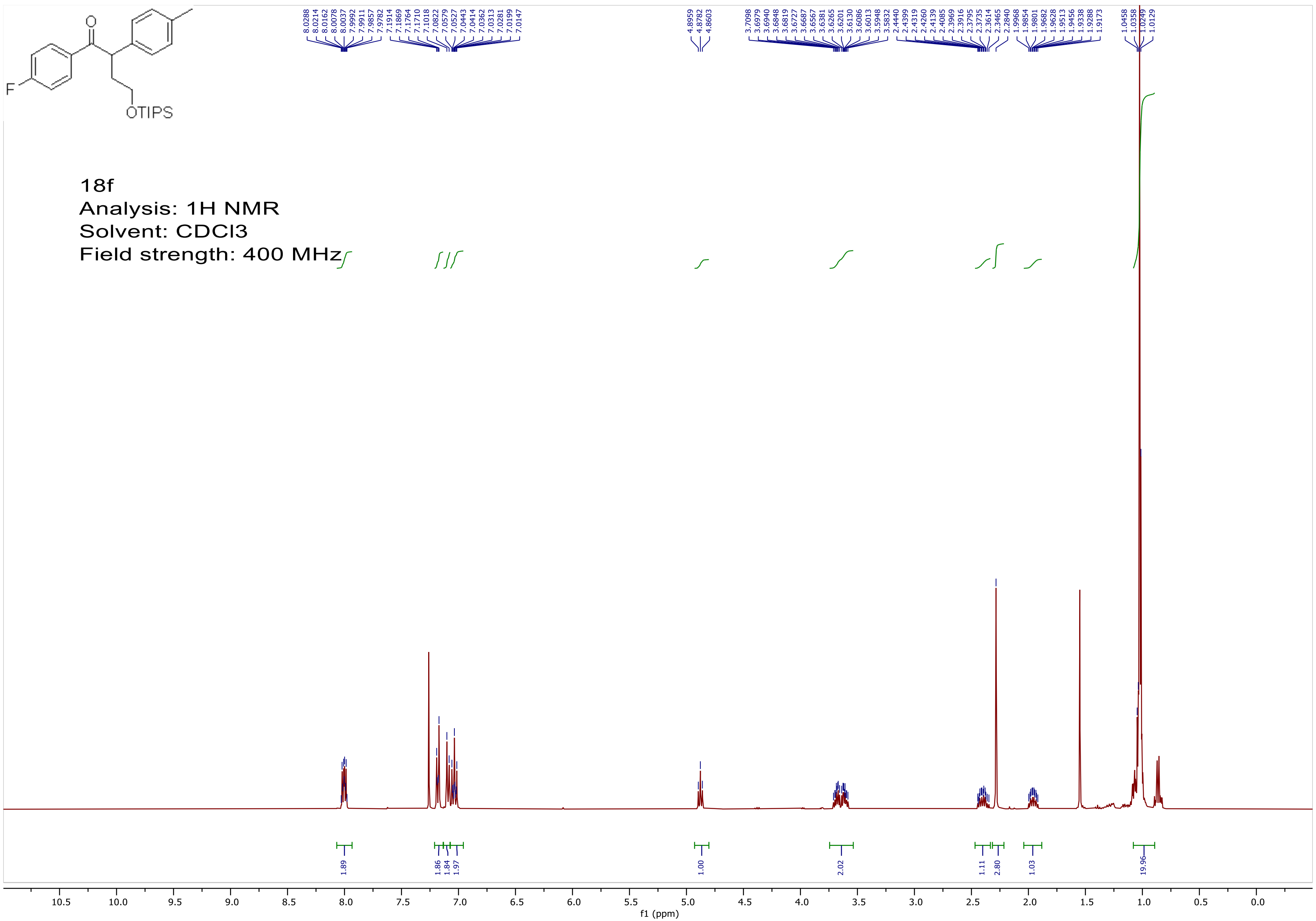
O
F
OTIPS
18f
Analysis: 1H NMR
Solvent: CDCl3
Field strength: 400 MHz
8.0288 8.0214 8.0162 8.0078 8.0037 7.9992 7.9911 7.9857 7.9782
7.1914 7.1869 7.1764 7.1710 7.1018 7.0822 7.0579 7.0527 7.0443 7.0414 7.0362 7.0313 7.0281 7.0199 7.0147
4.8959 4.8782 4.8603
3.7098 3.6979 3.6940 3.6848 3.6819 3.6727 3.6687 3.6567 3.6381 3.6265 3.6201 3.6130 3.6086 3.6013 3.5948 3.5832
2.4440 2.4399 2.4319 2.4260 2.4139 2.4085 2.3969 2.3916 2.3795 2.3735 2.3614 2.3465 2.2840
1.9968 1.9854 1.9801 1.9682 1.9628 1.9513 1.9456 1.9338 1.9288 1.9173
1.0458 1.0358 1.0249 1.0129
1.89
1.86
1.84
1.97
1.00
2.02
1.11
2.80
1.03
19.96
10.5 10.0 9.5 9.0 8.5 8.0 7.5 7.0 6.5 6.0 5.5 5.0 4.5 4.0 3.5 3.0 2.5 2.0 1.5 1.0 0.5 0.0
f1 (ppm)

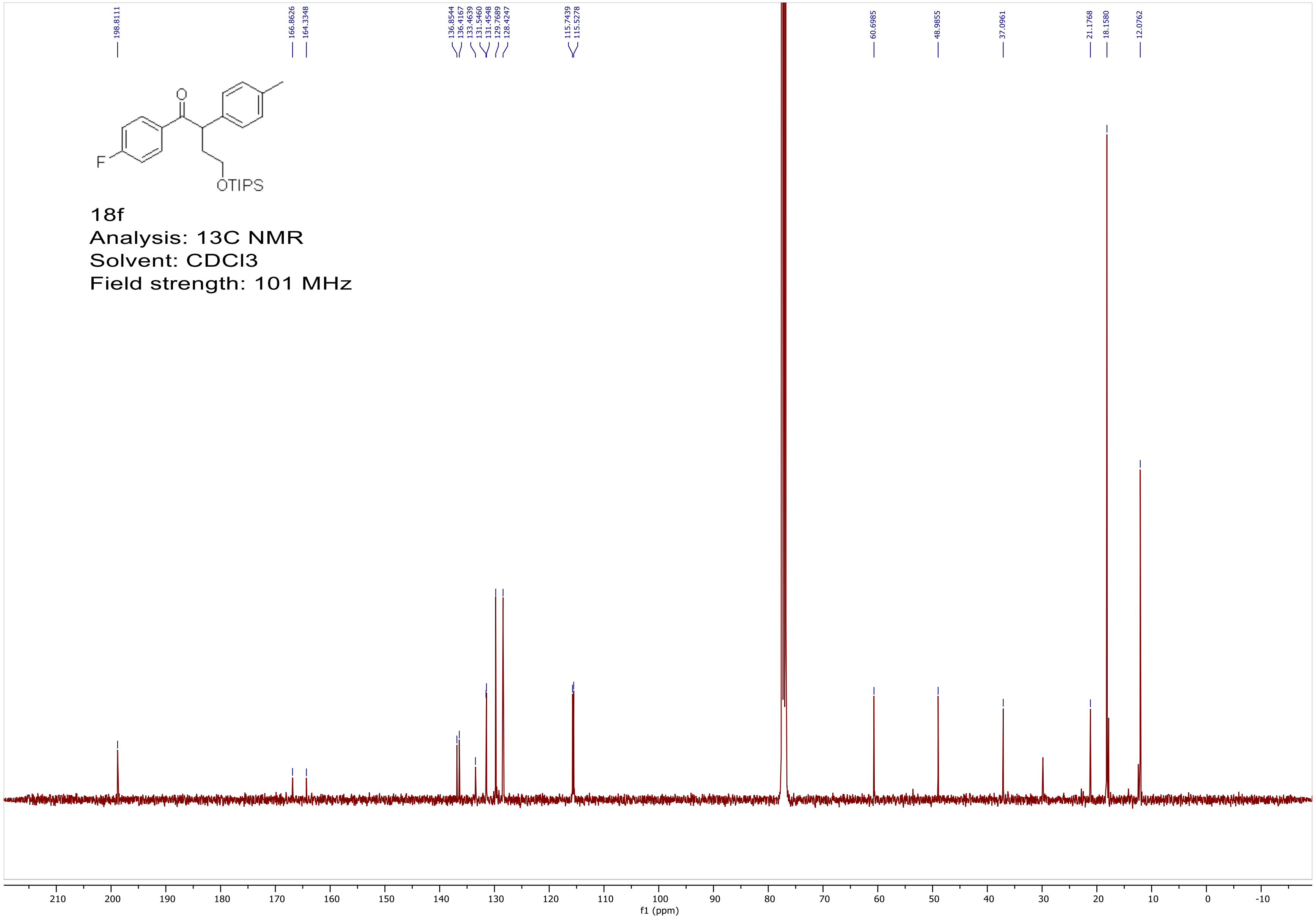
198.8111
166.8626
164.3348
136.8544
136.4167
133.4639
131.5460
131.4548
129.7689
128.4247
115.7439
115.5278
60.6985
48.9855
37.0961
21.1768
18.1580
12.0762
O
F
OTIPS
18f
Analysis: 13C NMR
Solvent: CDCl3
Field strength: 101 MHz
210
200
190
180
170
160
150
140
130
120
110
100
90
80
70
60
50
40
30
20
10
0
-10
f1 (ppm)

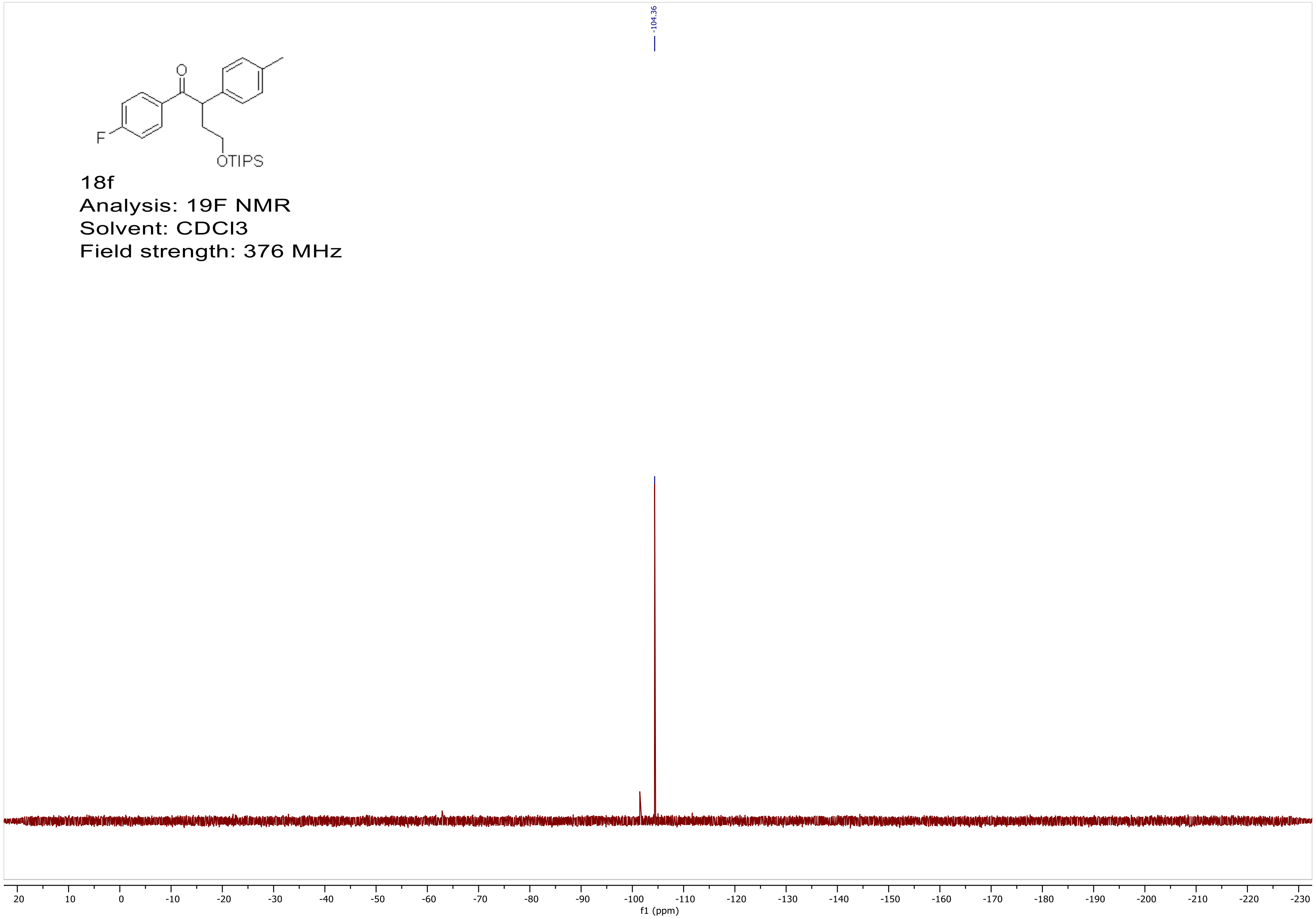
-104.36
O
F
OTIPS
18f
Analysis: 19F NMR
Solvent: CDCl3
Field strength: 376 MHz
20 10 0 -10 -20 -30 -40 -50 -60 -70 -80 -90 -100 -110 -120 -130 -140 -150 -160 -170 -180 -190 -200 -210 -220 -230
f1 (ppm)

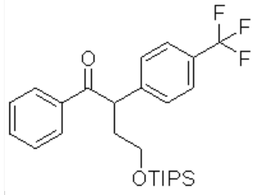

18g
Analysis: 1H NMR
Solvent: CDCl3
Field strength: 400 MHz

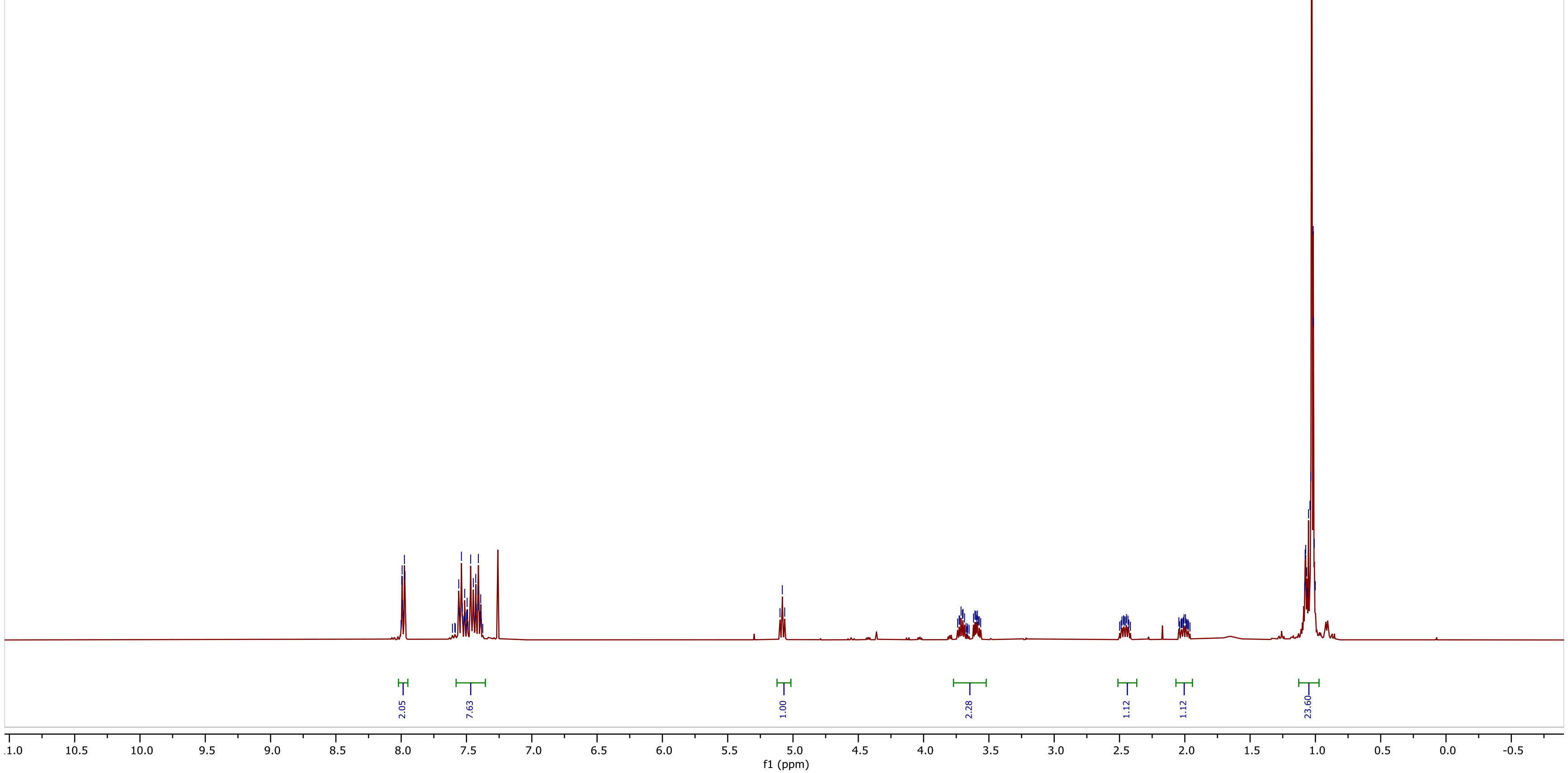

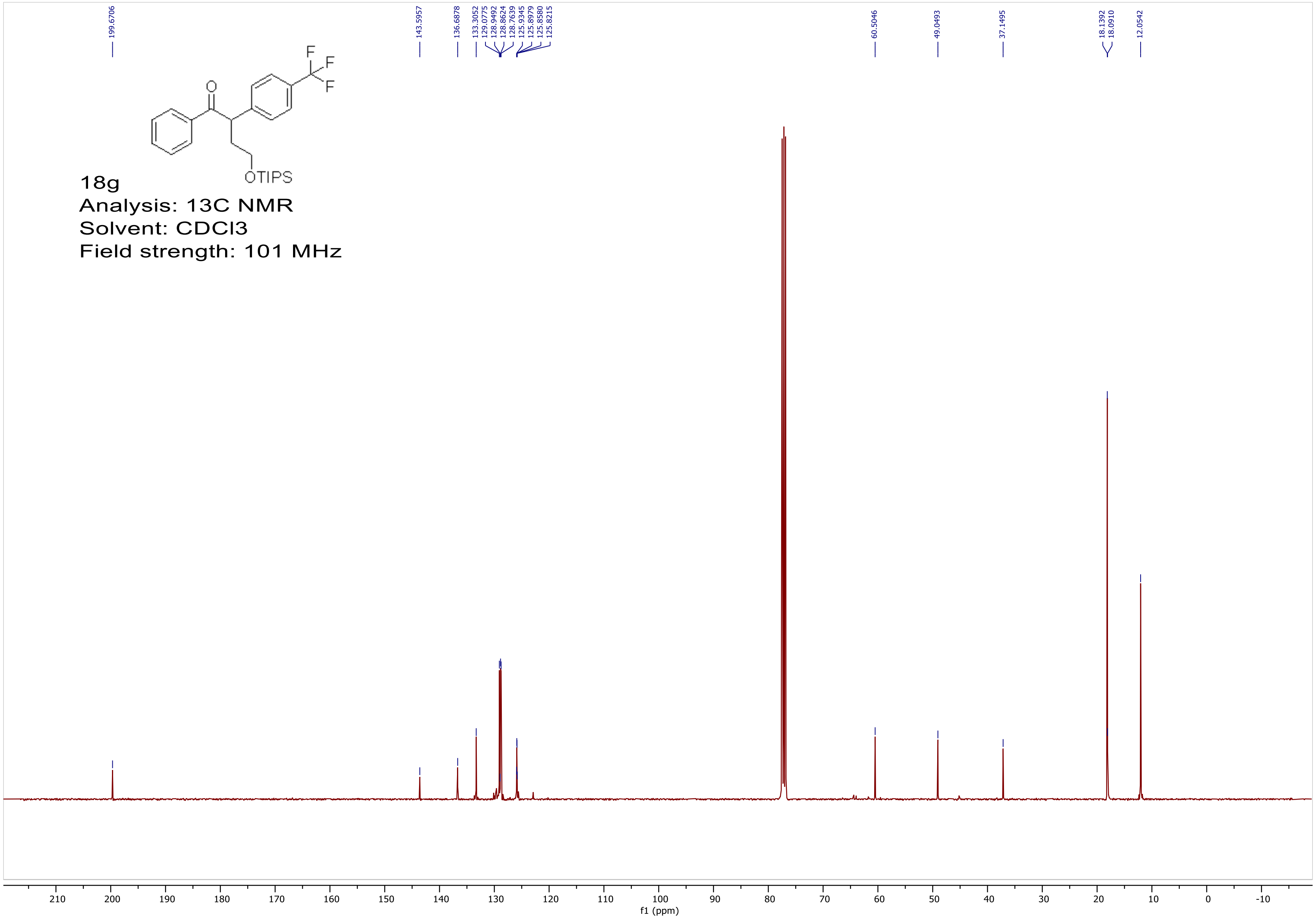

199.6706
143.5957
136.6878
133.3052
129.0775
128.9492
128.8624
128.7639
125.9345
125.8979
125.8580
125.8215
60.5046
49.0493
37.1495
18.1392
18.0910
12.0542
OTIPS
18g
Analysis: 13C NMR
Solvent: CDCl3
Field strength: 101 MHz
f1 (ppm)

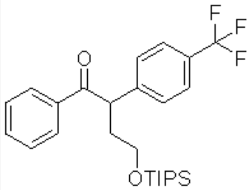

18g
Analysis: 19F NMR
Solvent: CDCl3
Field strength: 376 MHz

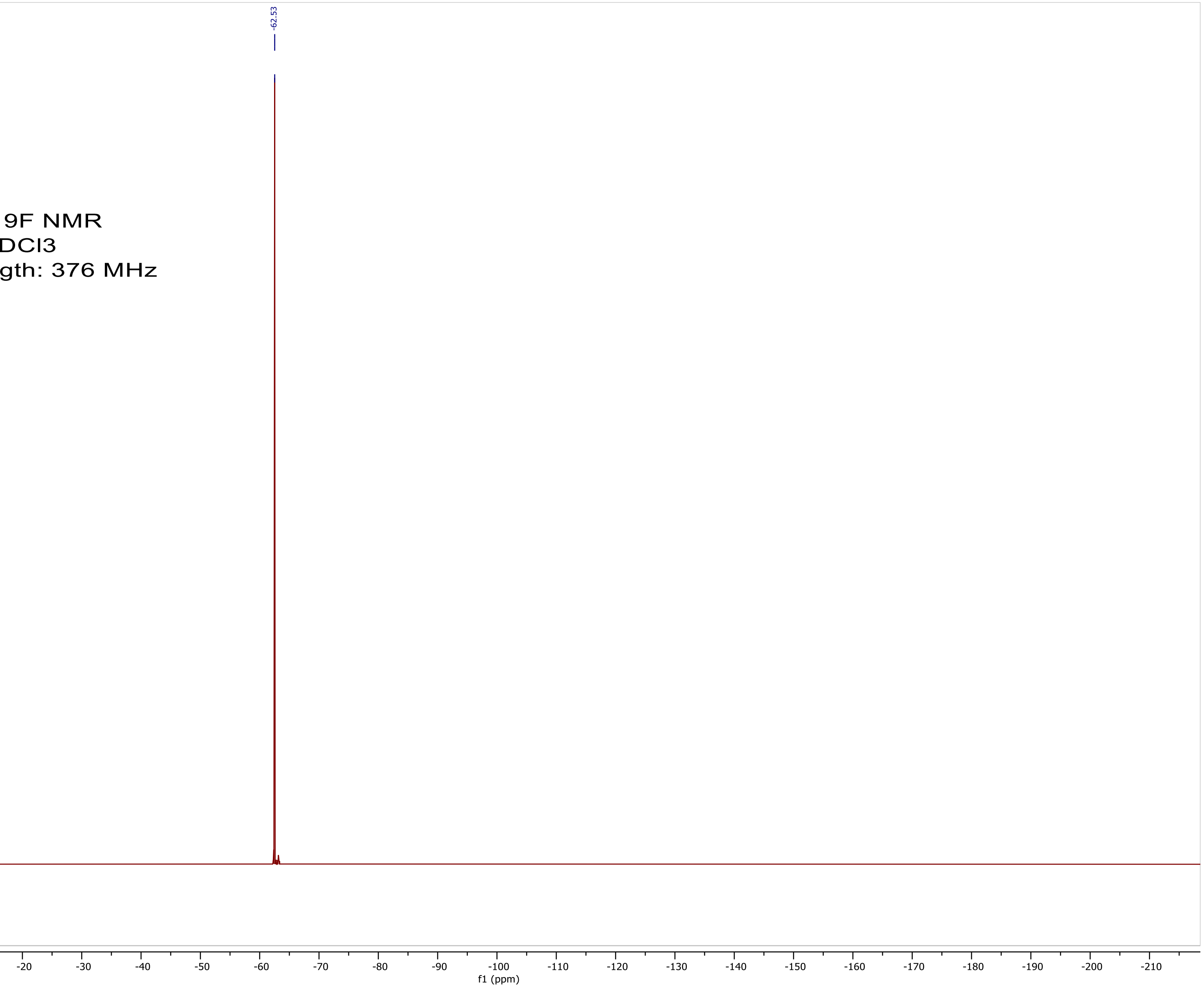

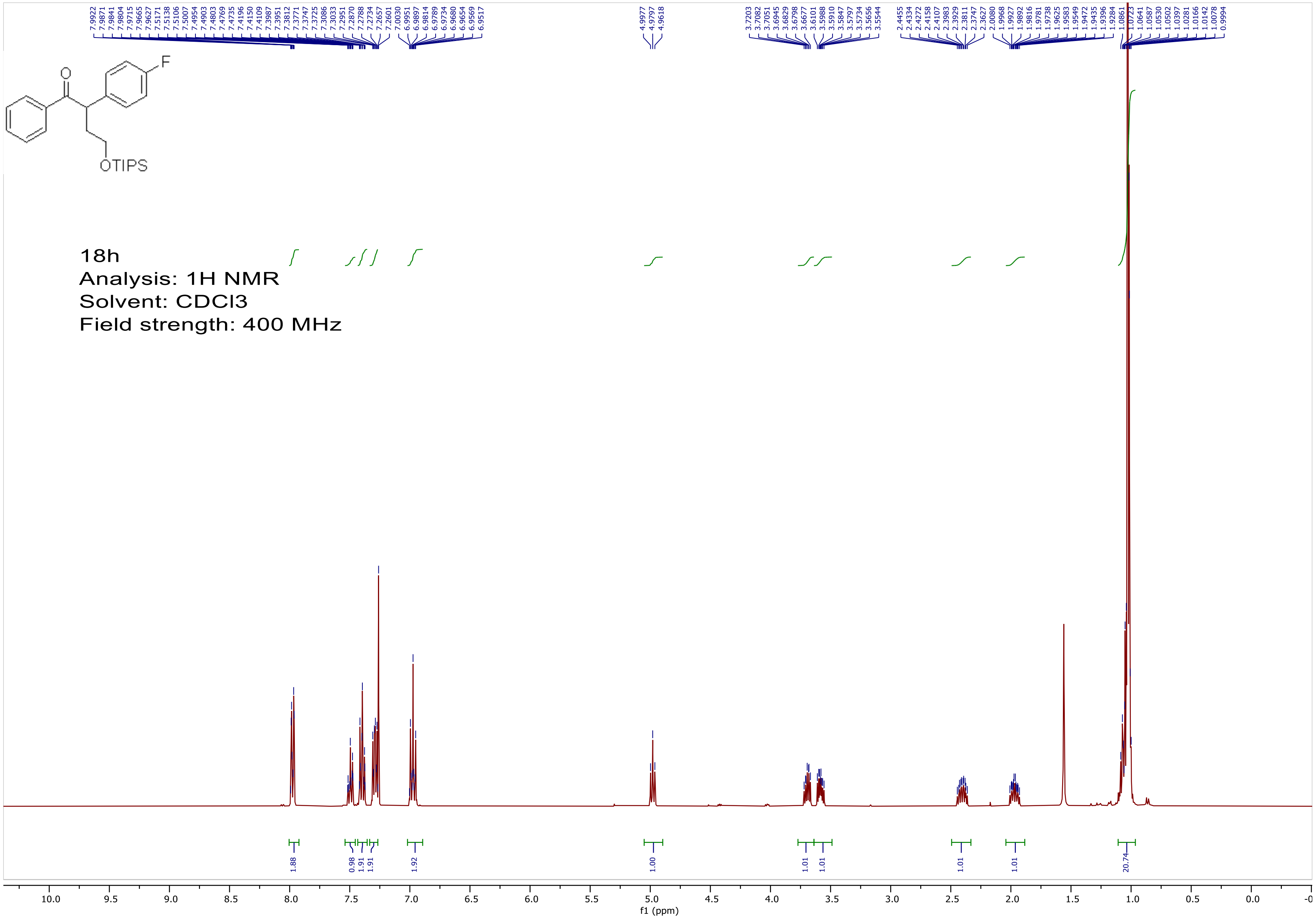

OTIPS
F
O
18h
Analysis: 1H NMR
Solvent: CDCl3
Field strength: 400 MHz
f1 (ppm)

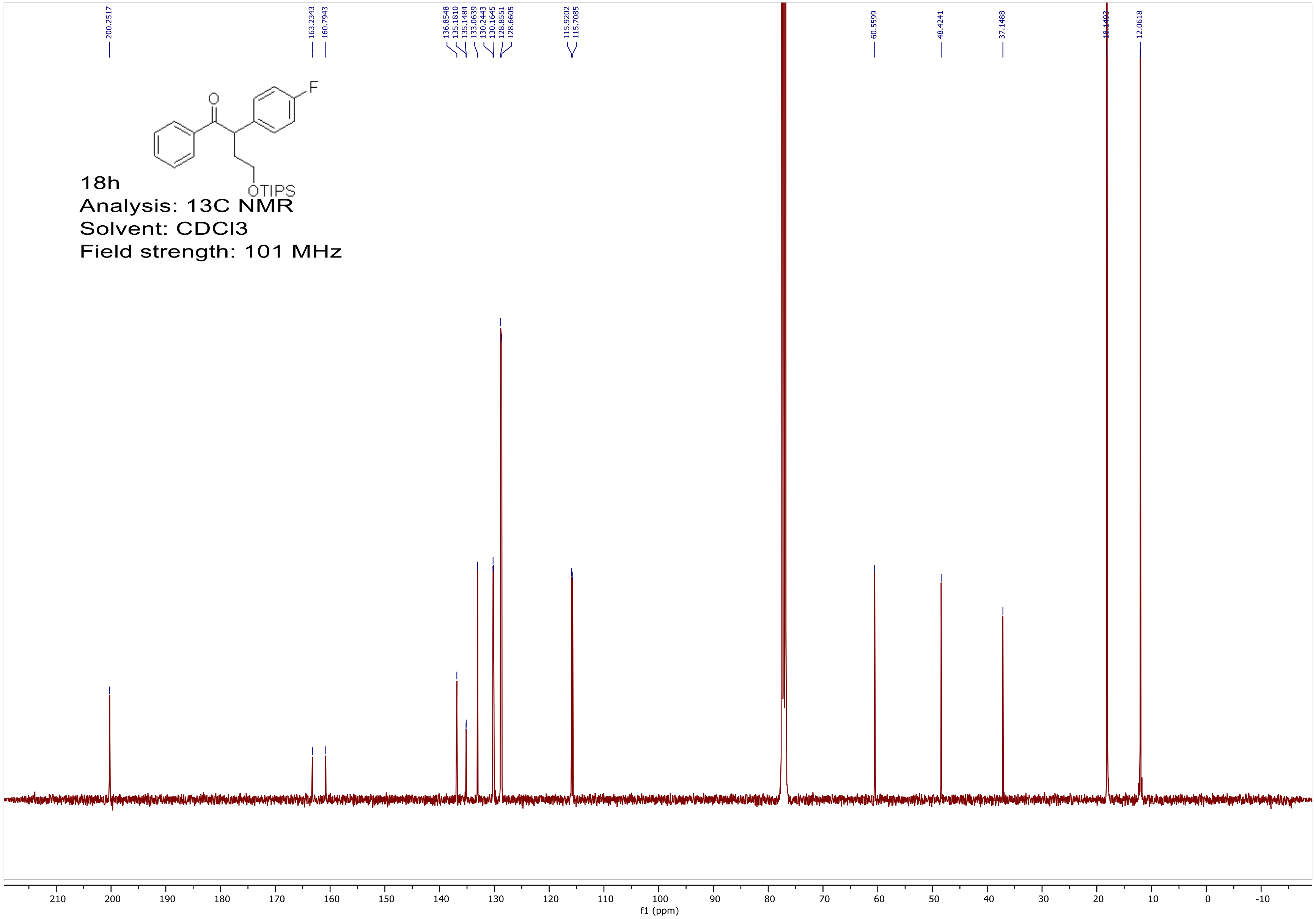

200.2517
163.2343
160.7943
136.8548
135.1810
135.1484
133.0639
130.2443
130.1645
128.8551
128.6605
115.9202
115.7085
60.5599
48.4241
37.1488
12.0618
F
O
OTIPS
18h
Analysis: 13C NMR
Solvent: CDCl3
Field strength: 101 MHz
210
200
190
180
170
160
150
140
130
120
110
100
90
80
70
60
50
40
30
20
10
0
-10
f1 (ppm)

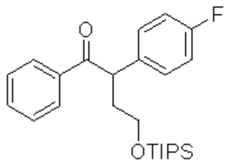

18h
Analysis: 19F NMR
Solvent: CDCl3
Field strength: 376 MHz

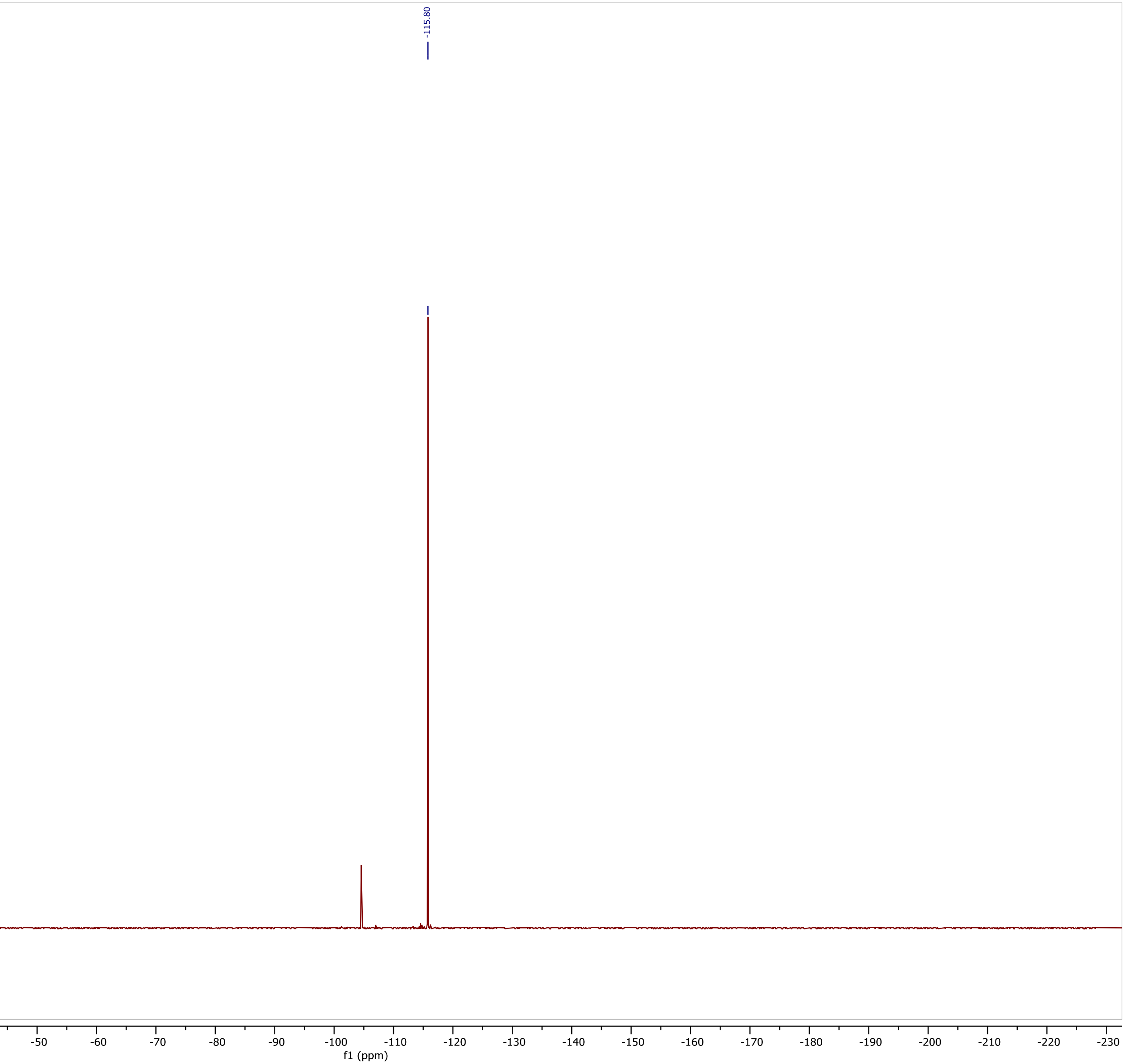

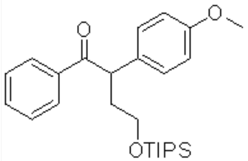

18i
Analysis: 1H NMR
Solvent: CDCl3
Field strength: 400 MHz

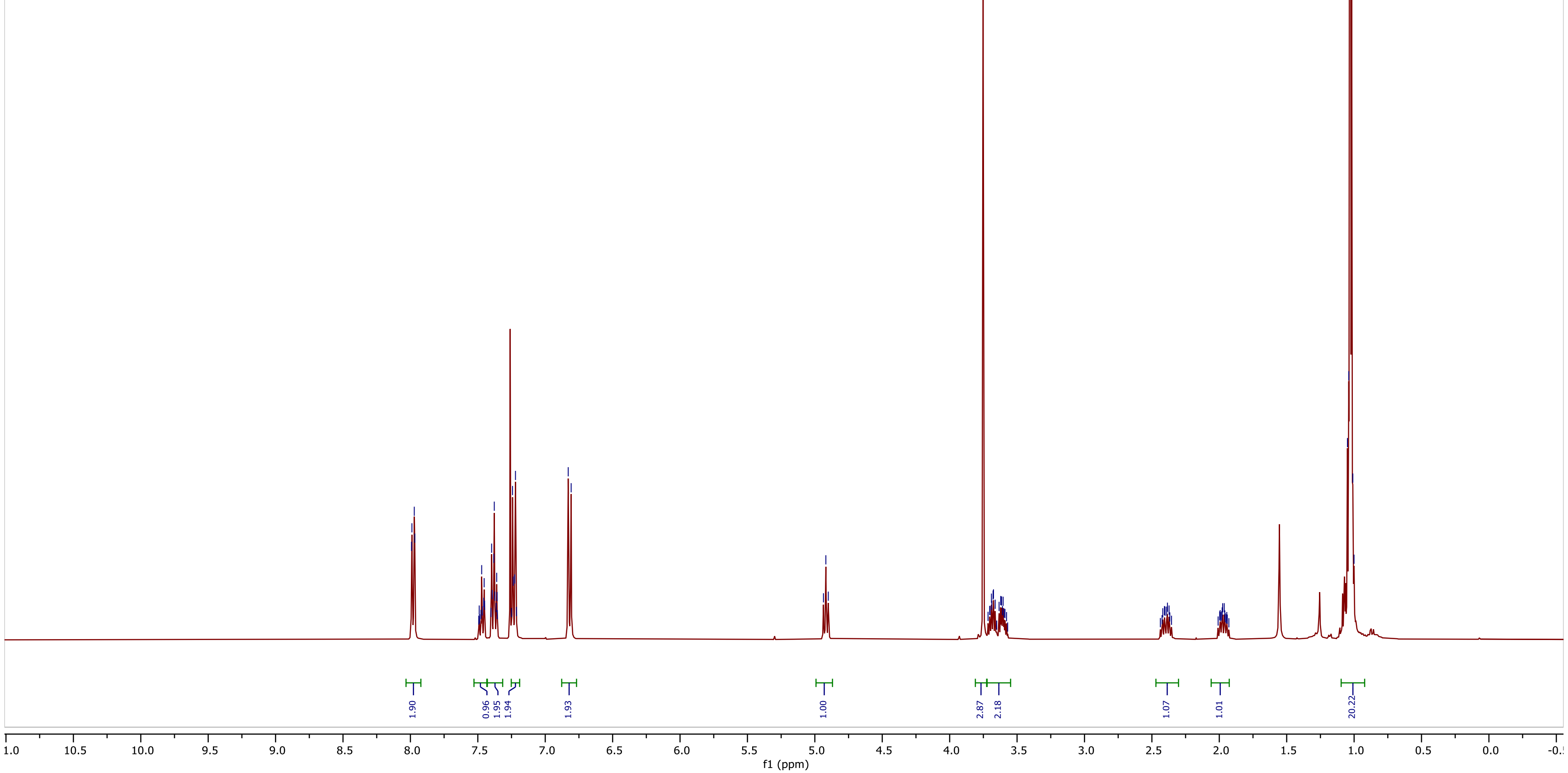

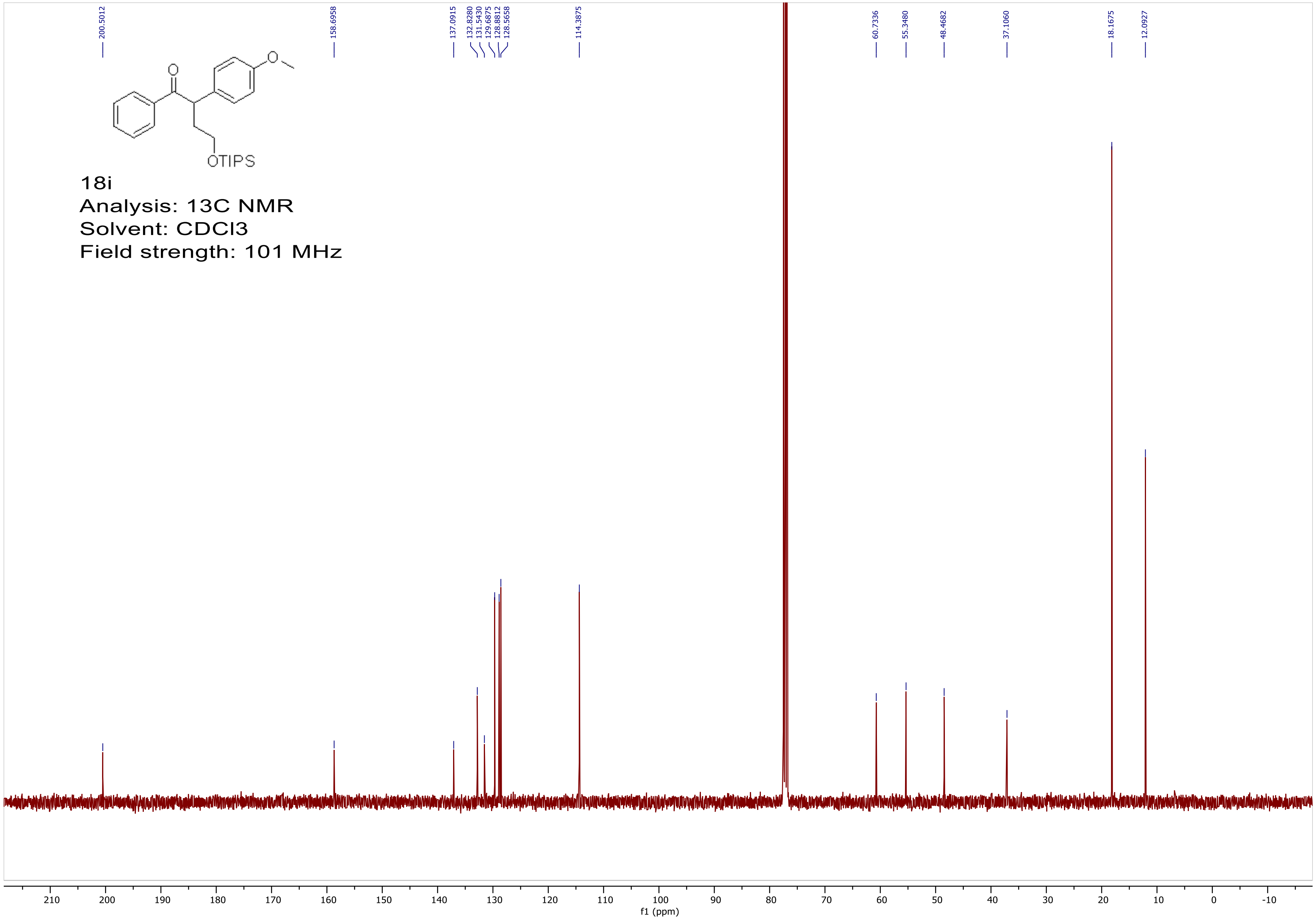

O
O
OTIPS
18i
Analysis: 13C NMR
Solvent: CDCl3
Field strength: 101 MHz
200.5012
158.6958
137.0915
132.8280
131.5430
129.6875
128.8812
128.5658
114.3875
60.7336
55.3480
48.4682
37.1060
18.1675
12.0927
210
200
190
180
170
160
150
140
130
120
110
100
90
80
70
60
50
40
30
20
10
0
-10
f1 (ppm)

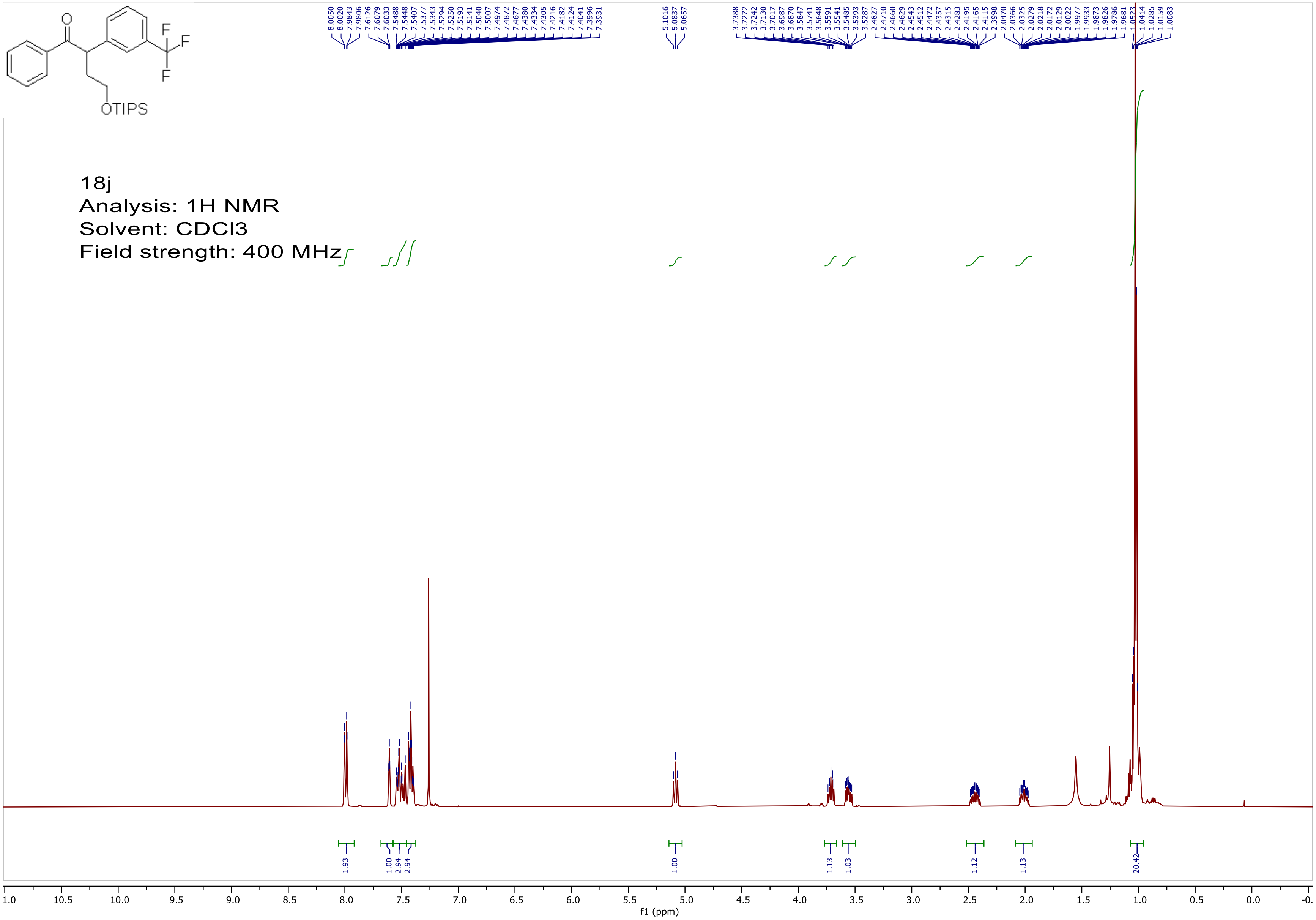
18j
Analysis: 1H NMR
Solvent: CDCl3
Field strength: 400 MHz
OTIPS
f1 (ppm)

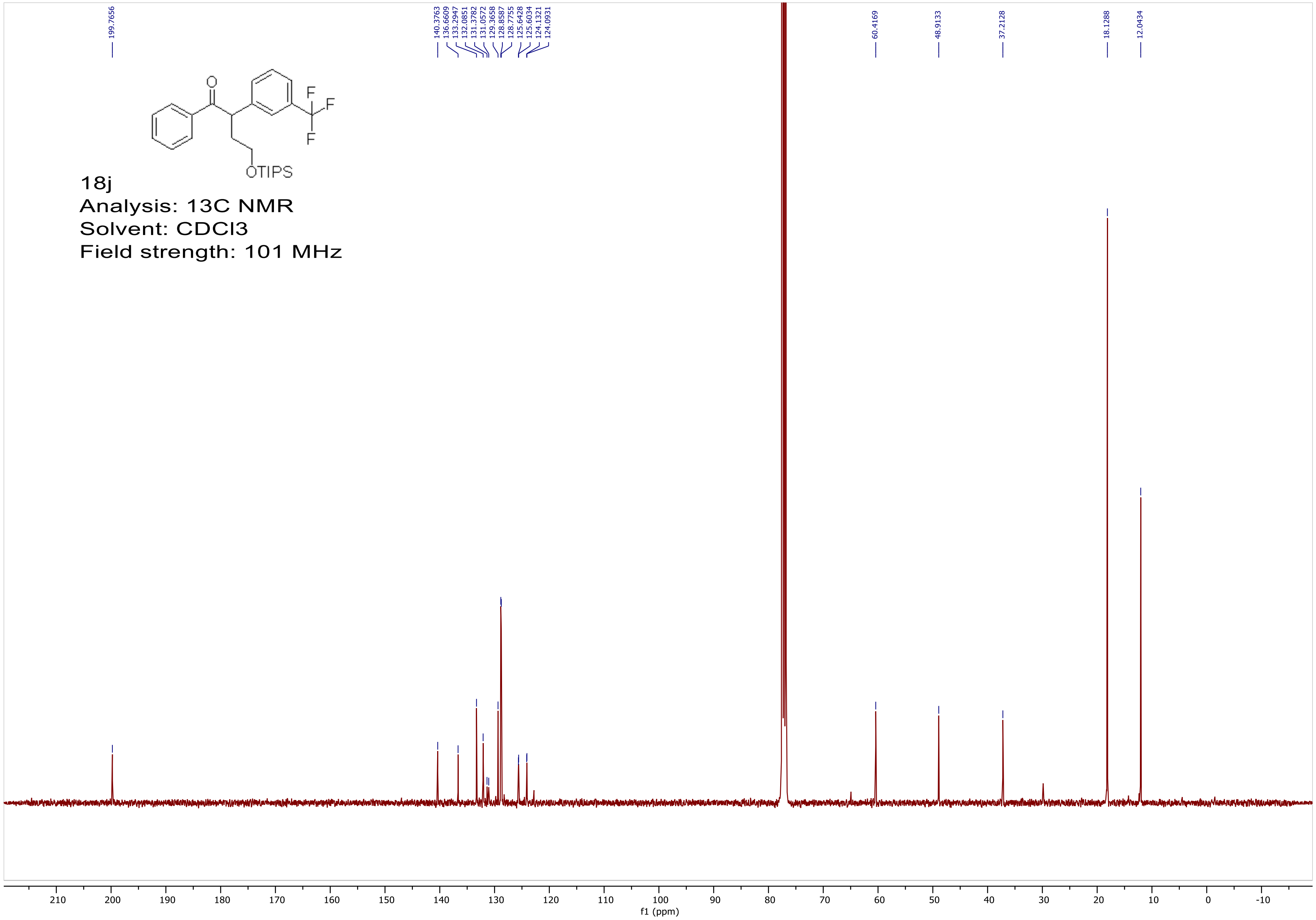
199.7656
140.3763
136.6609
133.2947
132.0851
131.3782
131.0572
129.3658
128.8587
128.7755
125.6428
125.6034
124.1321
124.0931
60.4169
48.9133
37.2128
18.1288
12.0434
F
F
F
O
OTIPS
18j
Analysis: 13C NMR
Solvent: CDCl3
Field strength: 101 MHz
210
200
190
180
170
160
150
140
130
120
110
100
90
80
70
60
50
40
30
20
10
0
-10
f1 (ppm)

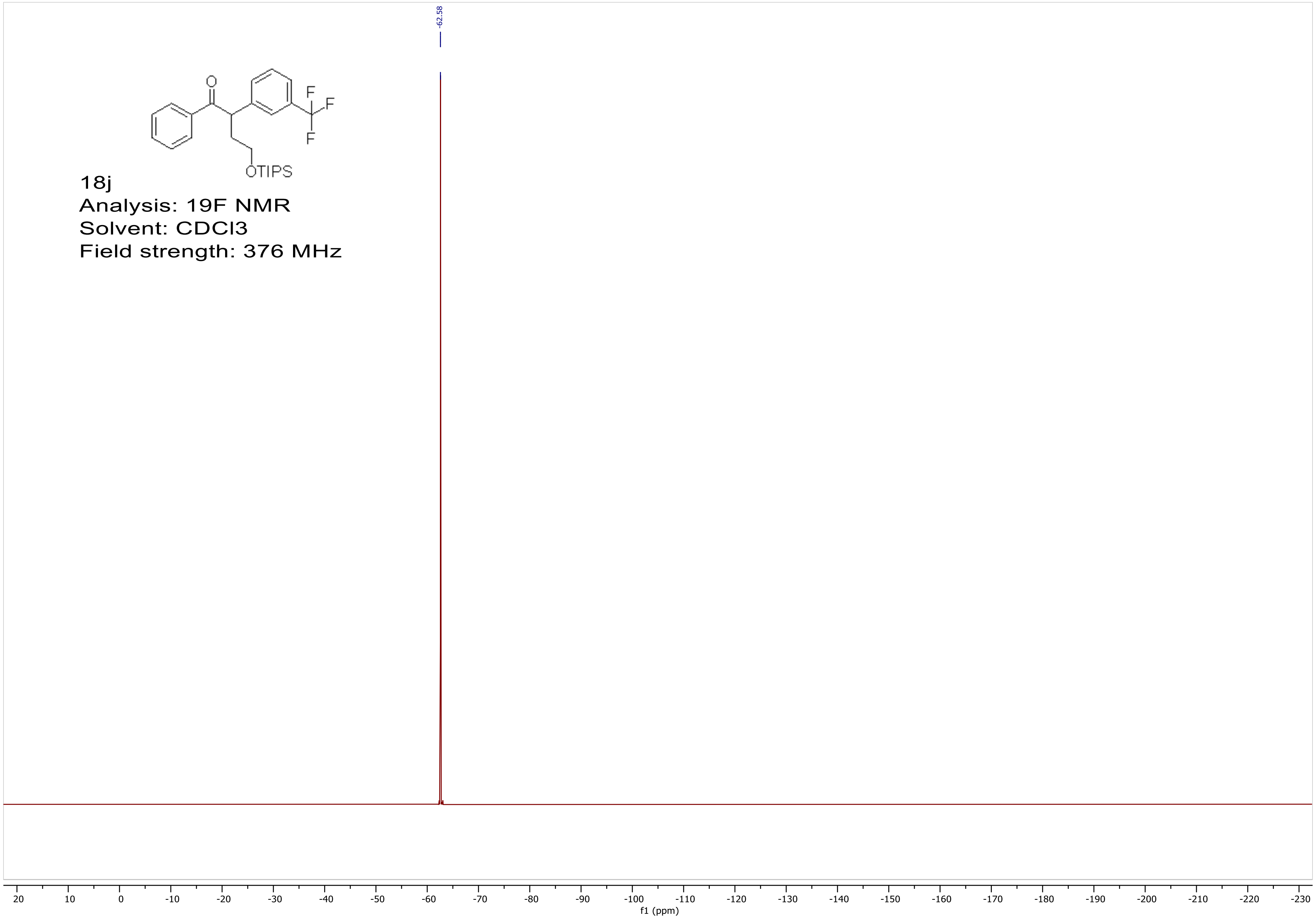
-62.58
O
F
F
F
OTIPS
18j
Analysis: 19F NMR
Solvent: CDCl3
Field strength: 376 MHz
20 10 0 -10 -20 -30 -40 -50 -60 -70 -80 -90 -100 -110 -120 -130 -140 -150 -160 -170 -180 -190 -200 -210 -220 -230
f1 (ppm)

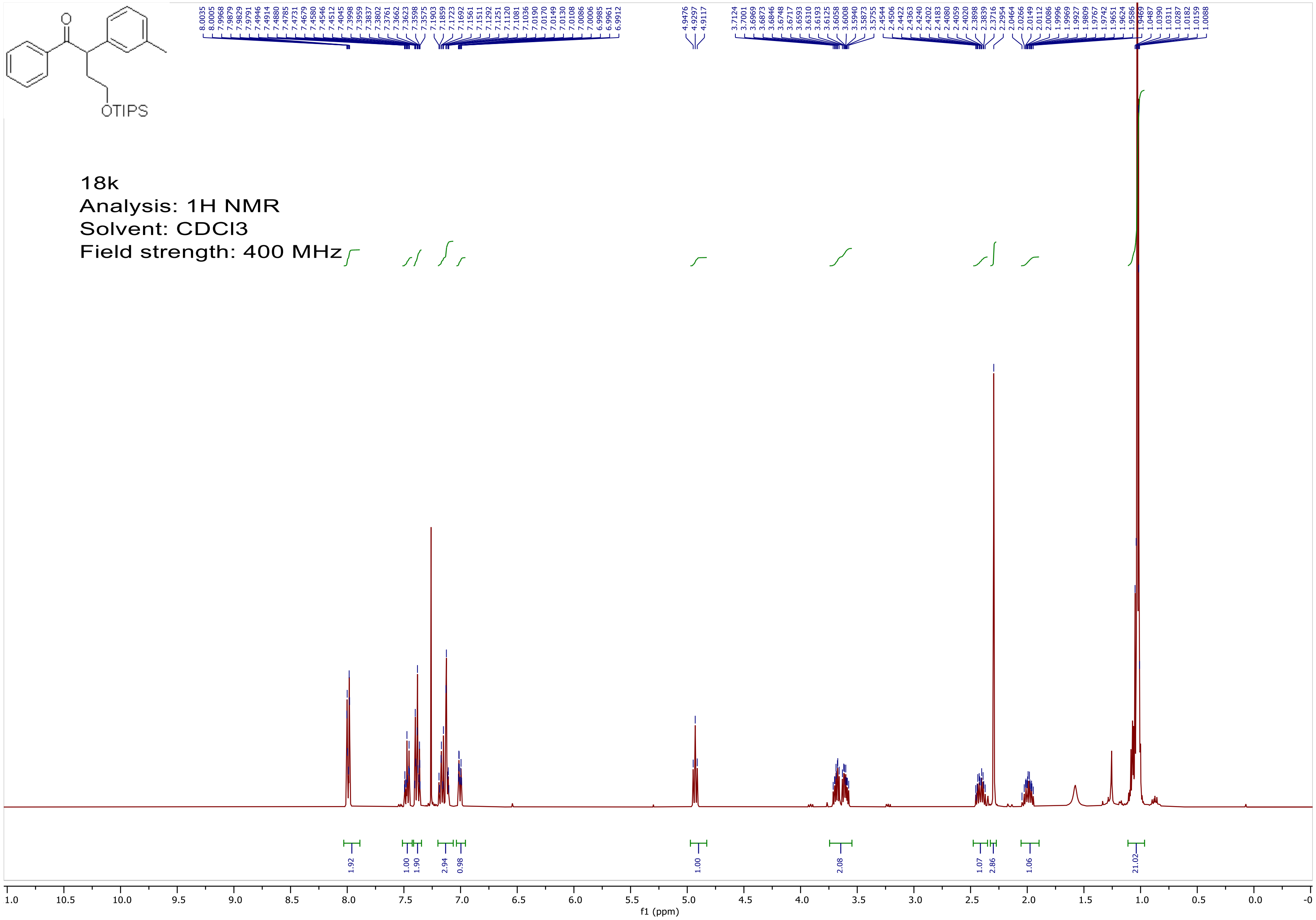
OTIPS
18k
Analysis: 1H NMR
Solvent: CDCl3
Field strength: 400 MHz
f1 (ppm)

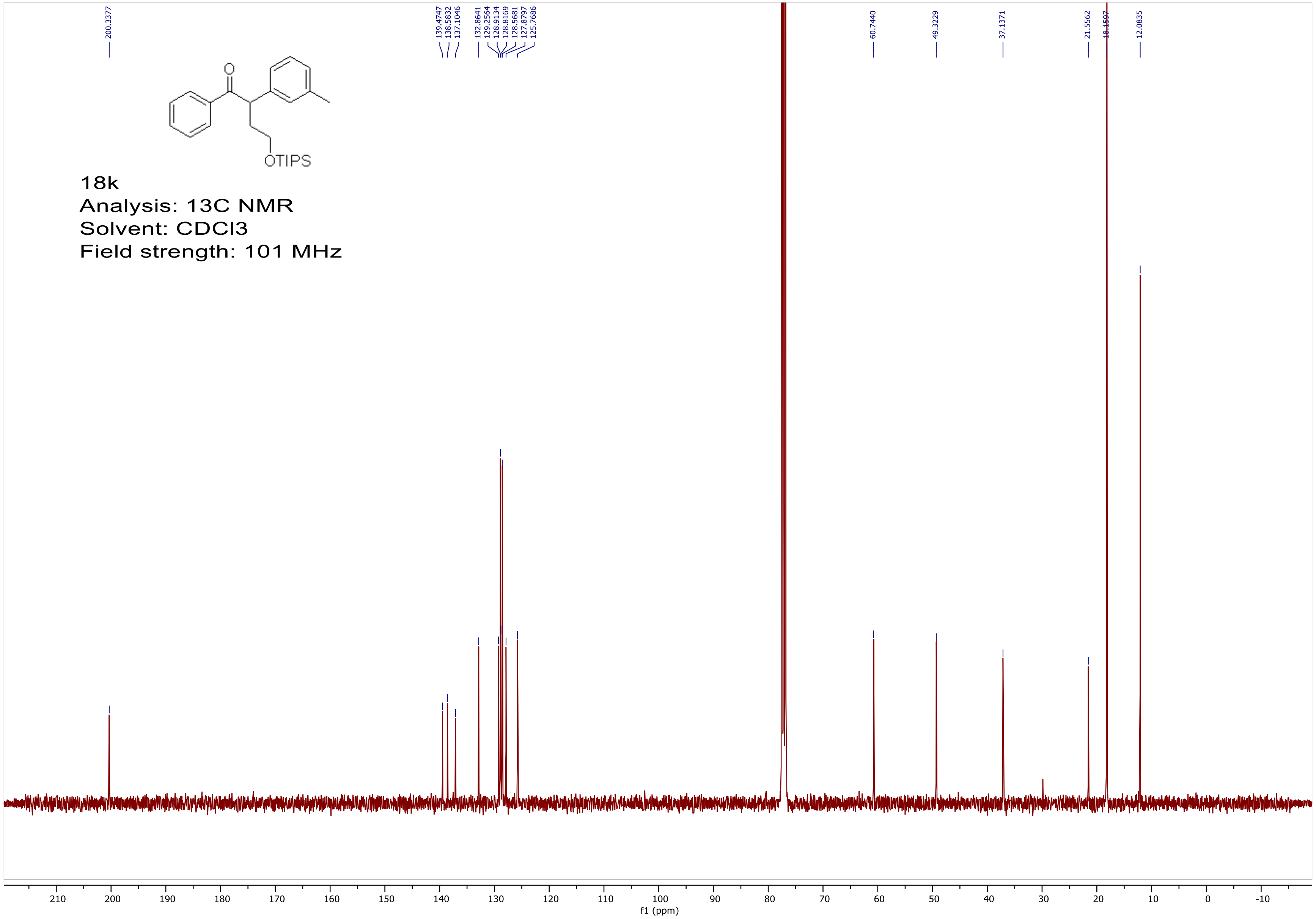
200.3377
139.4747
138.5832
137.1046
132.8641
129.2564
128.9134
128.8169
128.5681
127.8797
125.7666
60.7440
49.3229
37.1371
21.5562
18.1597
12.0835
OTIPS
18k
Analysis: 13C NMR
Solvent: CDCl3
Field strength: 101 MHz
210
200
190
180
170
160
150
140
130
120
110
100
90
80
70
60
50
40
30
20
10
0
-10
f1 (ppm)

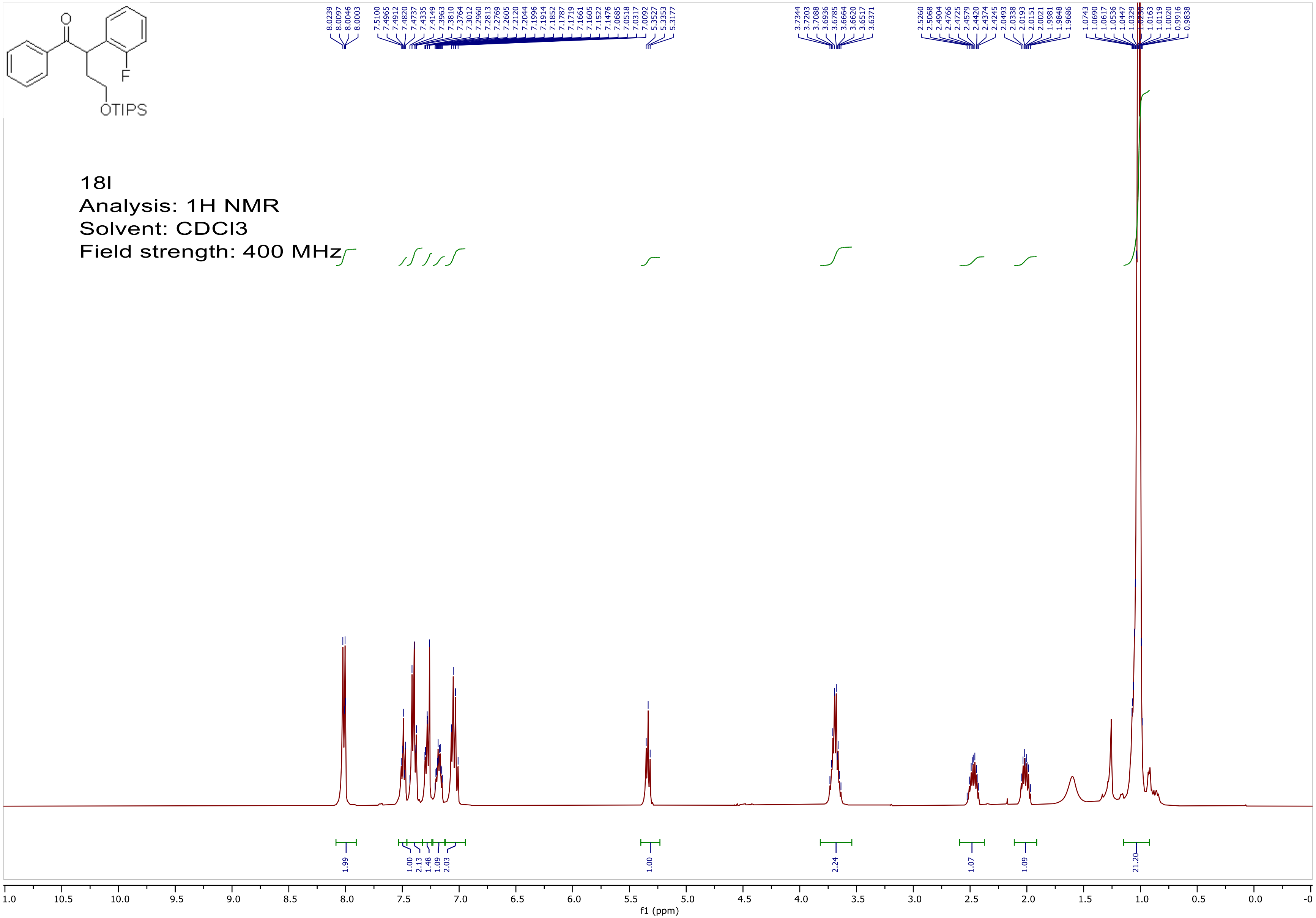
O
F
OTIPS
18l
Analysis: 1H NMR
Solvent: CDCl3
Field strength: 400 MHz
8.0239
8.0097
8.0046
8.0003
7.5100
7.4965
7.4912
7.4820
7.4737
7.4335
7.4149
7.3963
7.3810
7.3764
7.3012
7.2960
7.2813
7.2769
7.2605
7.2120
7.2044
7.1996
7.1914
7.1852
7.1787
7.1719
7.1661
7.1605
7.1522
7.1476
7.0685
7.0518
7.0317
7.0092
5.3527
5.3353
5.3177
3.7344
3.7203
3.7088
3.6936
3.6785
3.6664
3.6620
3.6517
3.6371
2.5260
2.5068
2.4904
2.4766
2.4725
2.4579
2.4420
2.4374
2.4245
2.0493
2.0338
2.0193
2.0151
2.0021
1.9981
1.9848
1.9686
1.0743
1.0690
1.0617
1.0536
1.0447
1.0329
1.0230
1.0163
1.0119
1.0020
0.9916
0.9838
1.99
1.00
2.13
1.48
1.09
2.03
1.00
2.24
1.07
1.09
21.20
f1 (ppm)

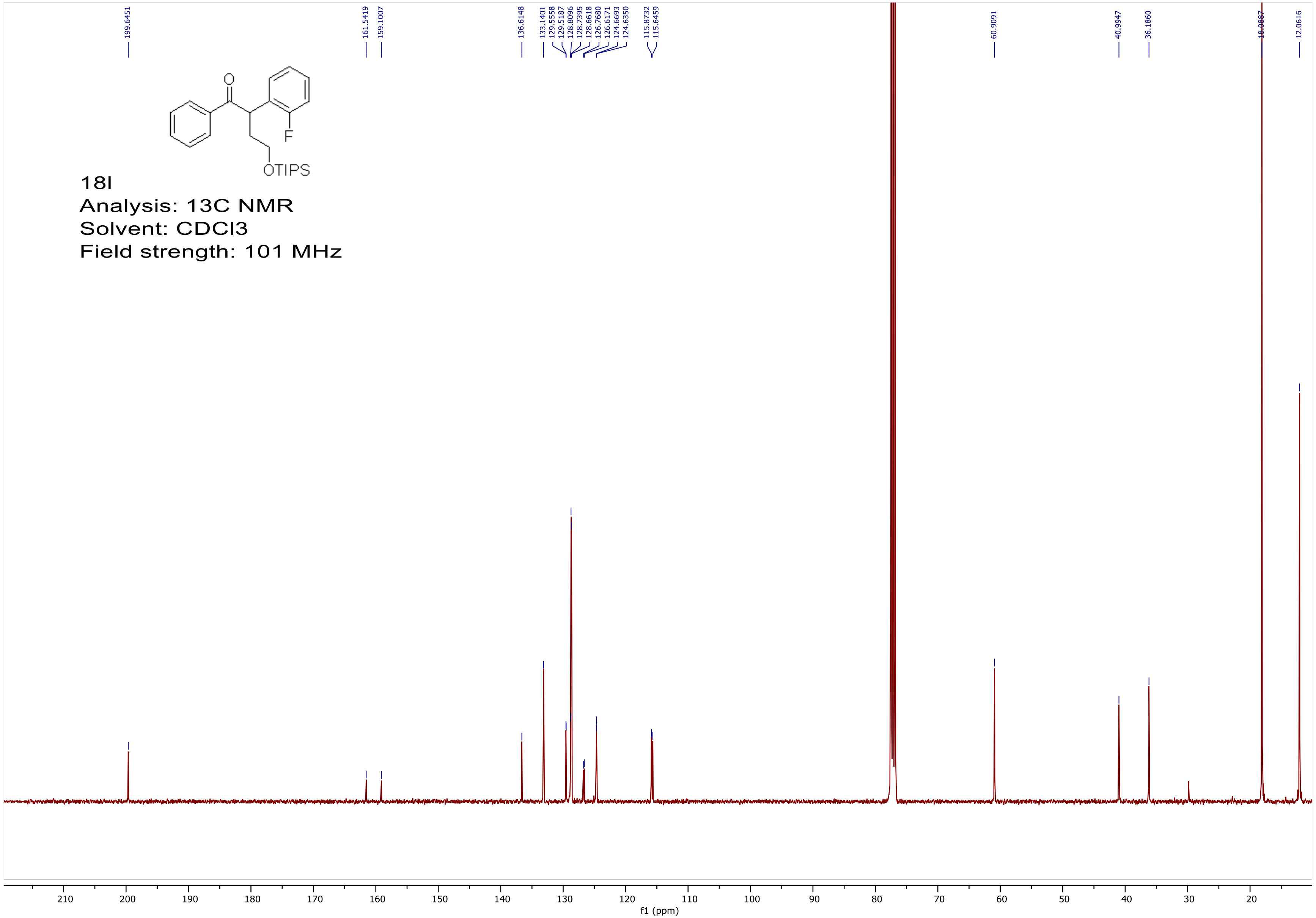
199.6451
161.5419
159.1007
136.6148
133.1401
129.5558
129.5187
128.8096
128.7395
128.6618
126.7680
126.6171
124.6693
124.6350
115.8732
115.6459
60.9091
40.9947
36.1860
18.0887
12.0616
O
F
OTIPS
18l
Analysis: 13C NMR
Solvent: CDCl3
Field strength: 101 MHz
210 200 190 180 170 160 150 140 130 120 110 100 90 80 70 60 50 40 30 20
f1 (ppm)

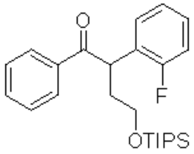

18l
Analysis: 19F NMR
Solvent: CDCl3
Field strength: 376 MHz

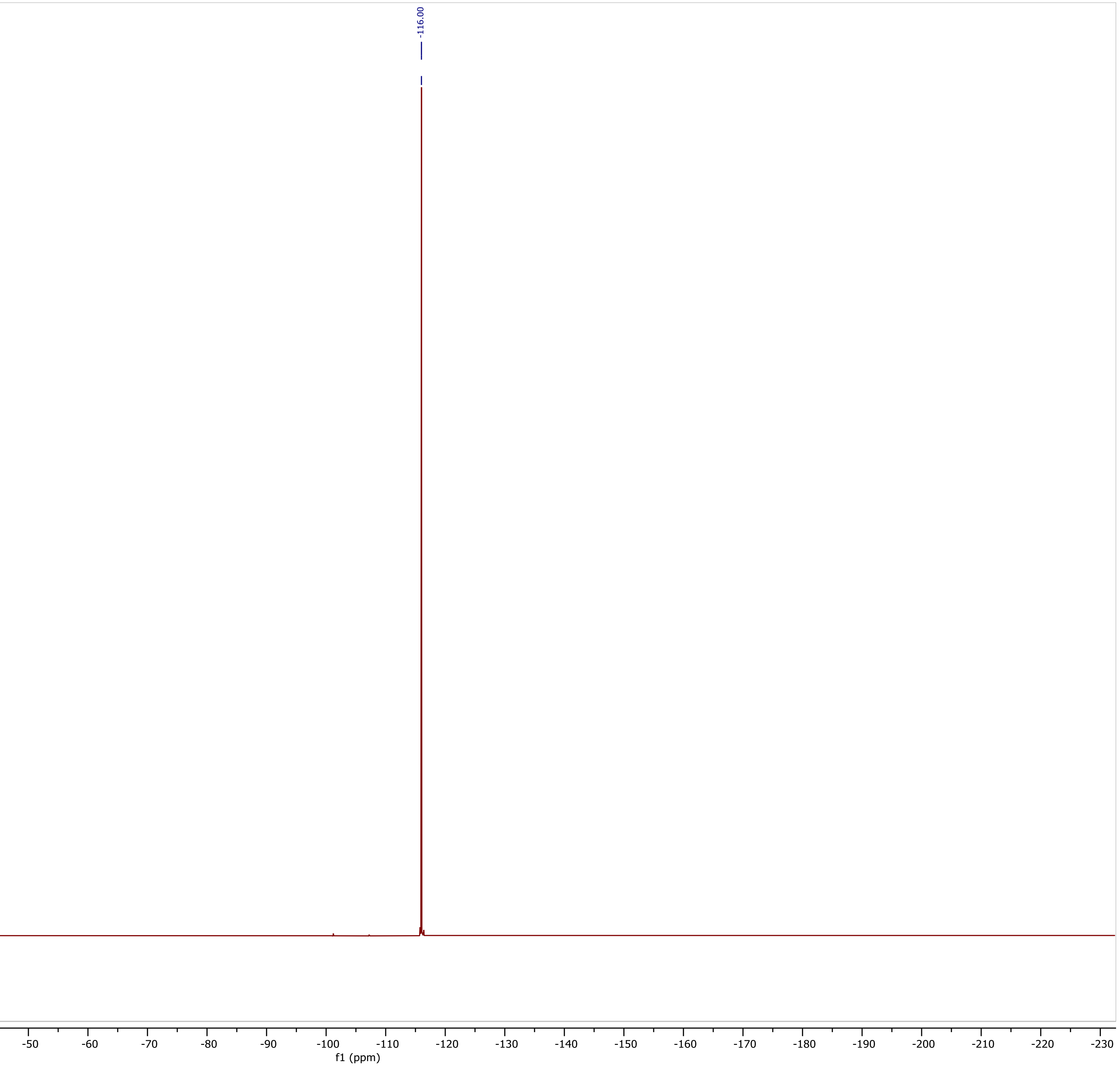

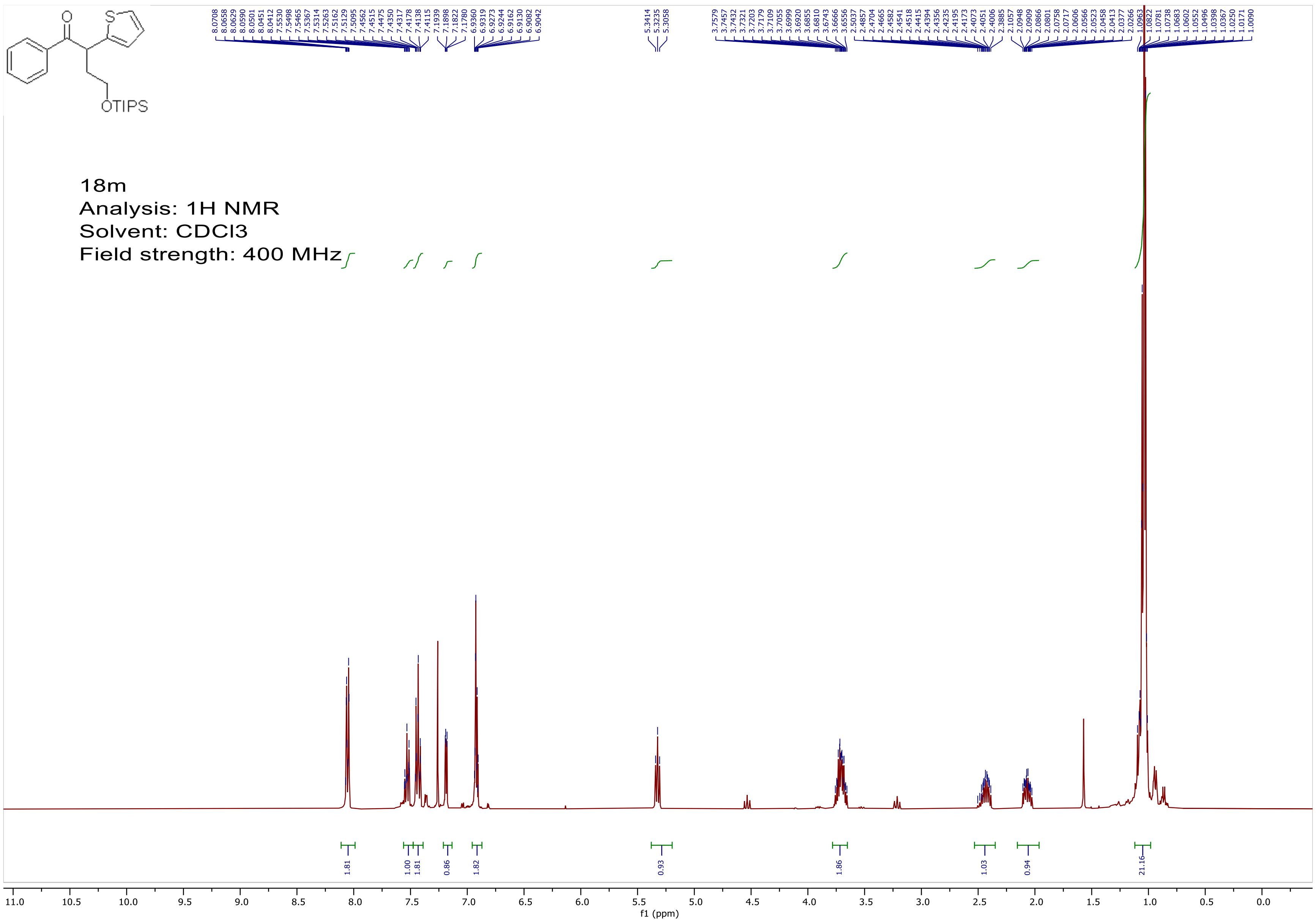
OTIPS
18m
Analysis: 1H NMR
Solvent: CDCl3
Field strength: 400 MHz
f1 (ppm)

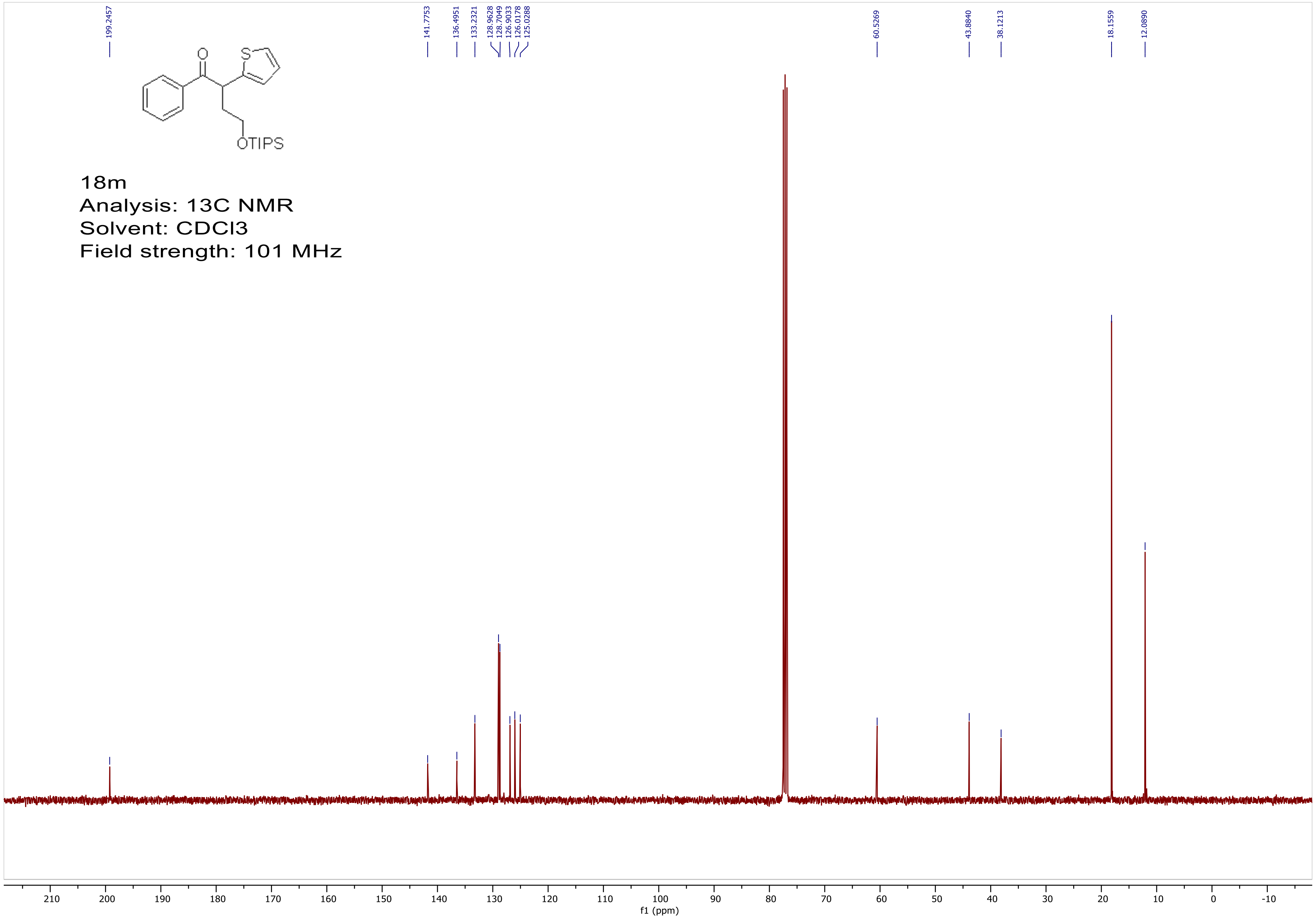
199.2457
141.7753
136.4951
133.2321
128.9628
128.7049
126.9033
126.0178
125.0288
60.5269
43.8840
38.1213
18.1559
12.0890
O
S
OTIPS
18m
Analysis: 13C NMR
Solvent: CDCl3
Field strength: 101 MHz
210 200 190 180 170 160 150 140 130 120 110 100 90 80 70 60 50 40 30 20 10 0 -10
f1 (ppm)

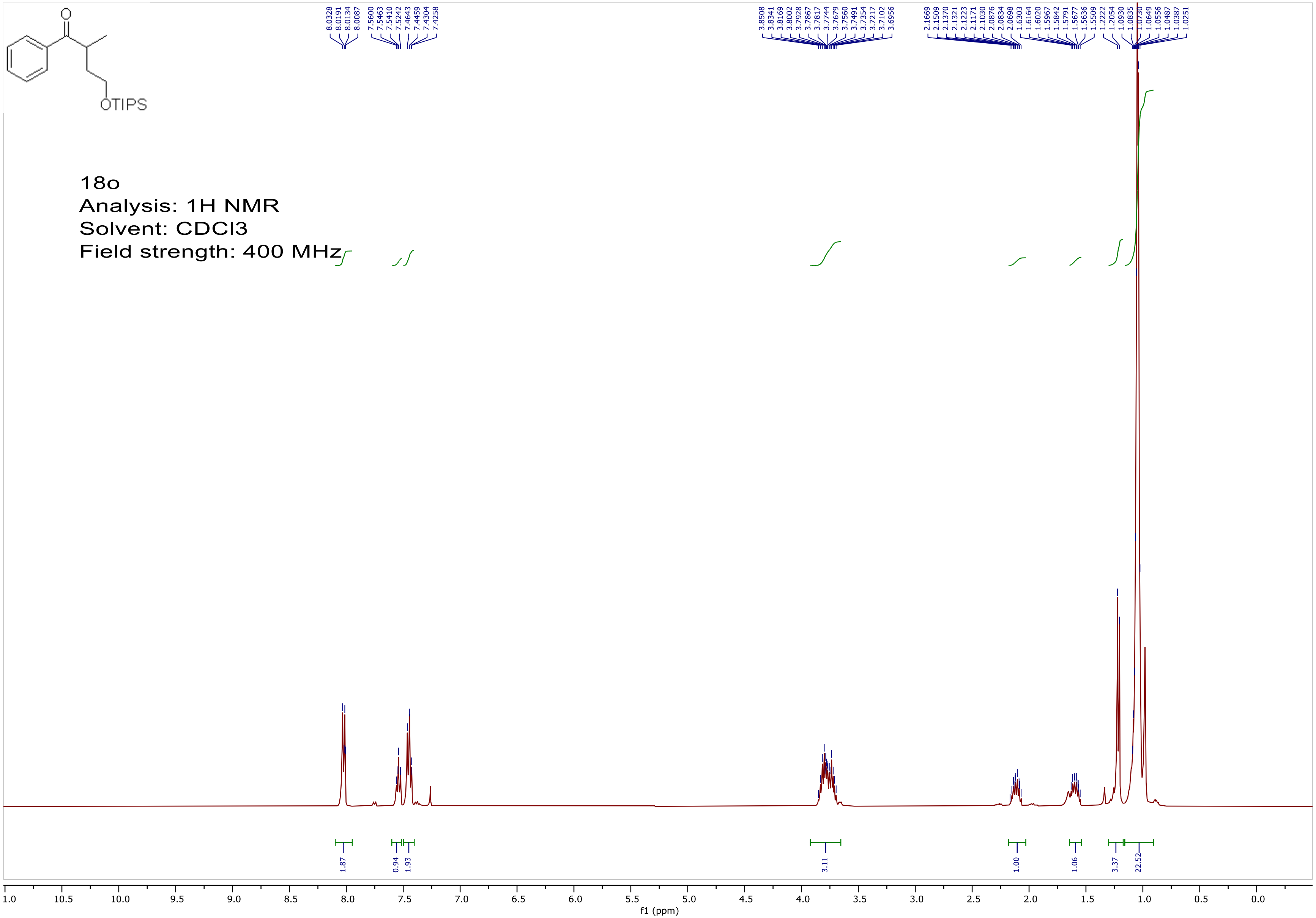
OTIPS
18o
Analysis: 1H NMR
Solvent: CDCl3
Field strength: 400 MHz
8.0328
8.0191
8.0134
8.0087
7.5600
7.5463
7.5410
7.5242
7.4643
7.4459
7.4304
7.4258
3.8508
3.8341
3.8169
3.8002
3.7928
3.7867
3.7817
3.7744
3.7679
3.7560
3.7491
3.7354
3.7217
3.7102
3.6956
2.1669
2.1509
2.1370
2.1321
2.1223
2.1171
2.1030
2.0876
2.0834
2.0698
1.6303
1.6164
1.6020
1.5967
1.5842
1.5791
1.5677
1.5626
1.5509
1.2222
1.2054
1.0930
1.0835
1.0730
1.0649
1.0556
1.0487
1.0387
1.0251
1.87
0.94
1.93
3.11
1.00
1.06
3.37
22.52
f1 (ppm)

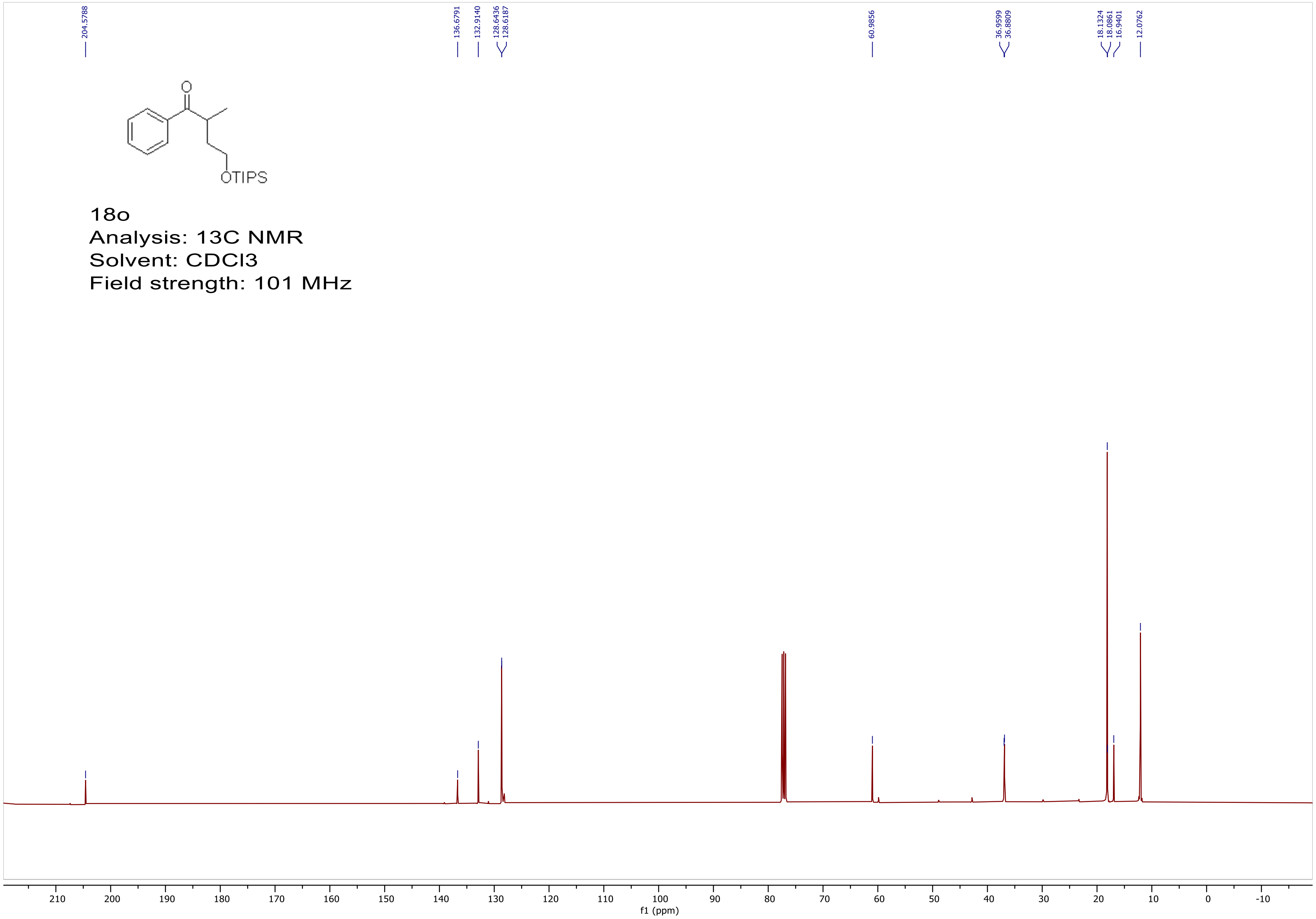
204.5788
136.6791
132.9140
128.6436
128.6187
60.9856
36.9599
36.8809
18.1324
18.0861
16.9401
12.0762
OTIPS
18o
Analysis: 13C NMR
Solvent: CDCl3
Field strength: 101 MHz
210 200 190 180 170 160 150 140 130 120 110 100 90 80 70 60 50 40 30 20 10 0 -10
f1 (ppm)

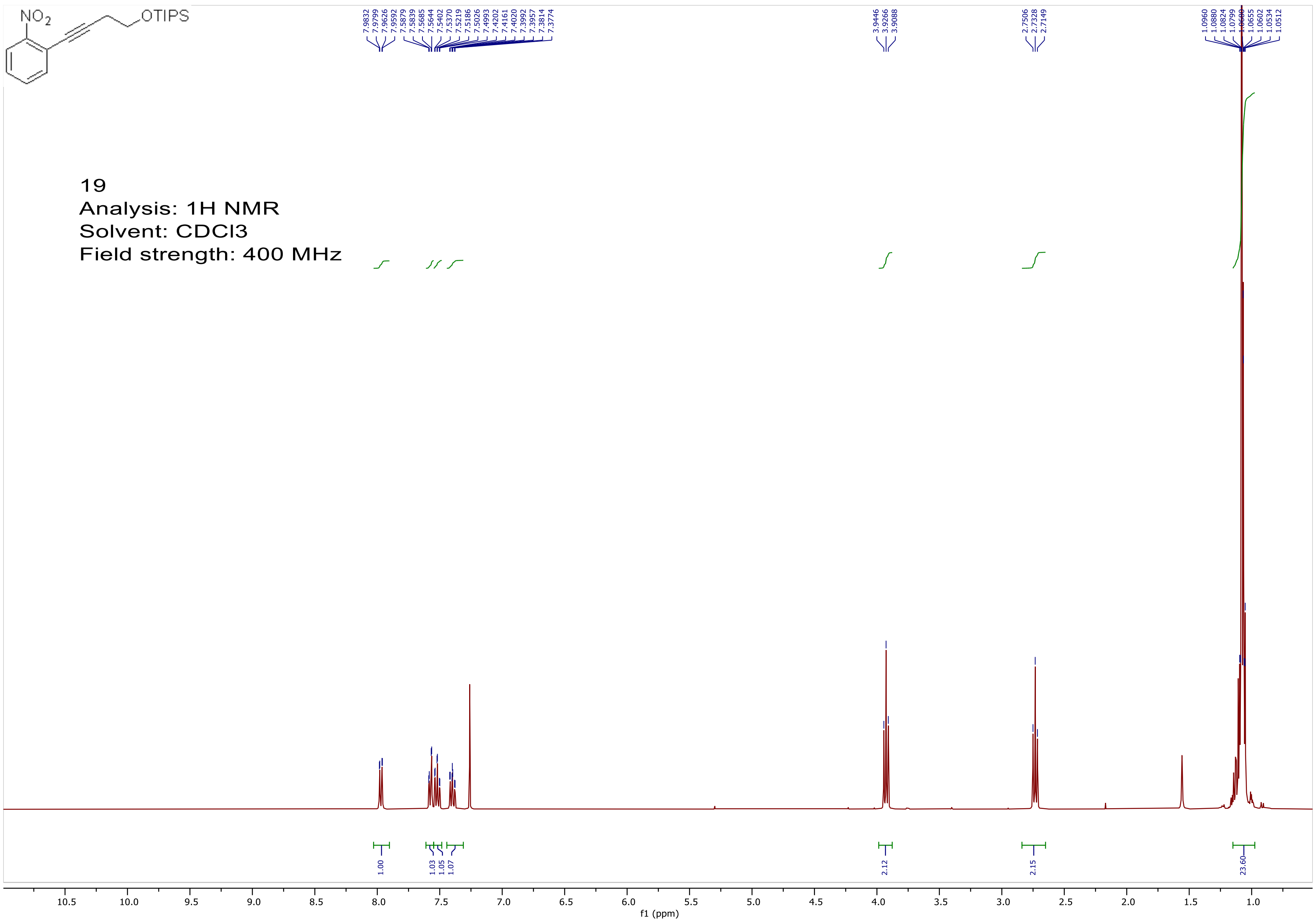
19
Analysis: 1H NMR
Solvent: CDCl3
Field strength: 400 MHz
f1 (ppm)

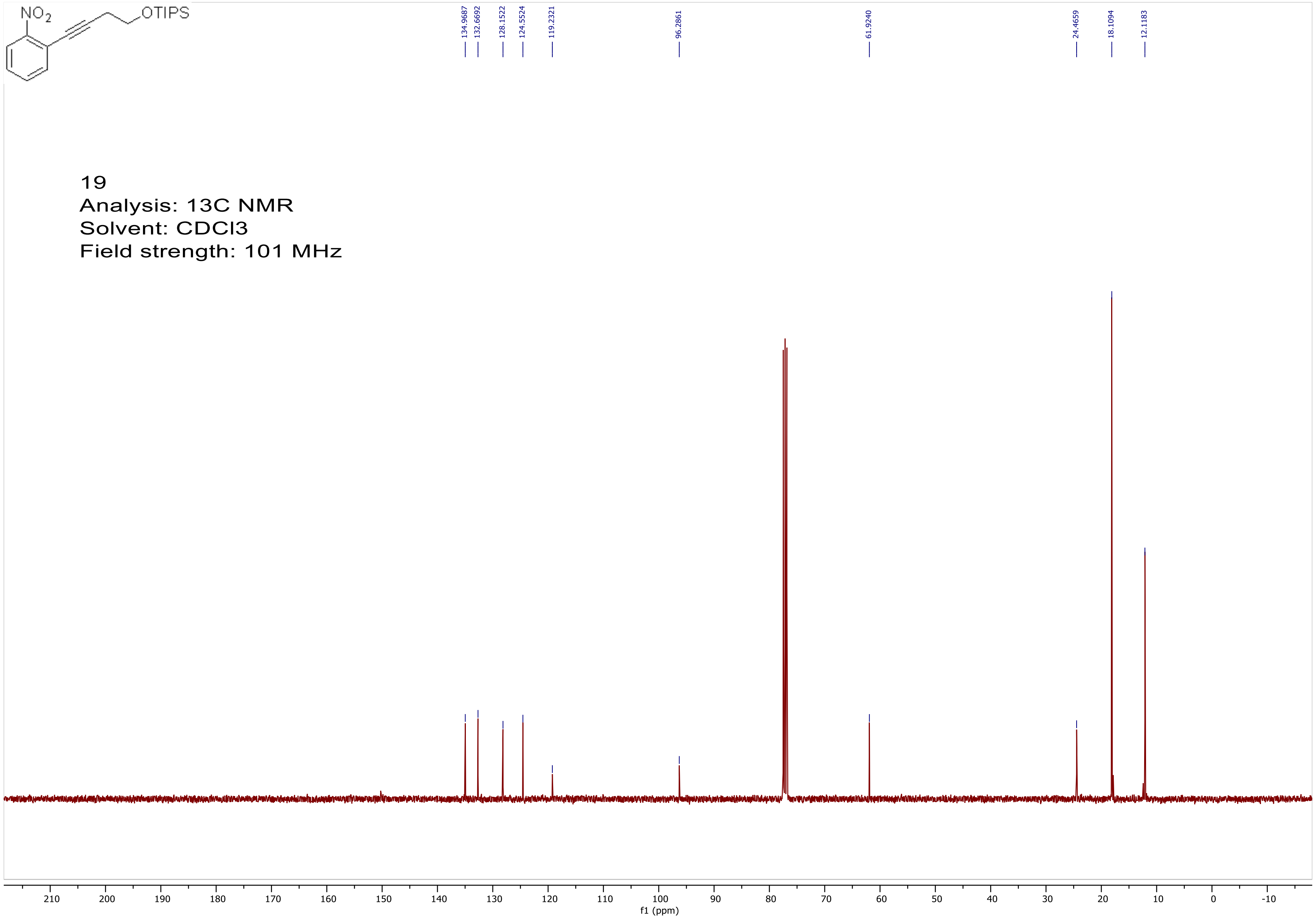
NO2
OTIPS
134.9687
132.6692
128.1522
124.5524
119.2321
96.2861
61.9240
24.4659
18.1094
12.1183
19
Analysis: 13C NMR
Solvent: CDCl3
Field strength: 101 MHz
210 200 190 180 170 160 150 140 130 120 110 100 90 80 70 60 50 40 30 20 10 0 -10
f1 (ppm)

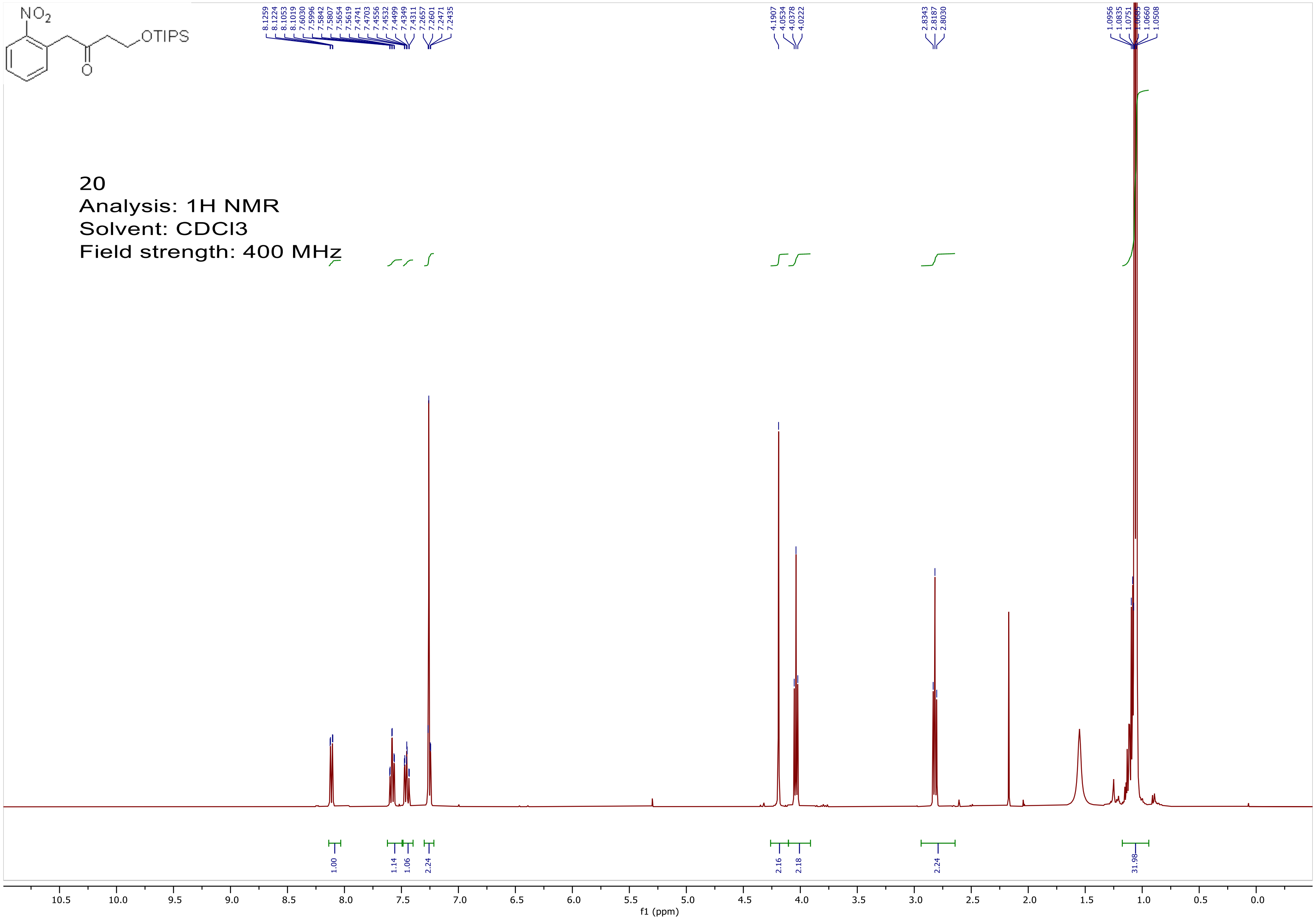
NO2
OTIPS
O
20
Analysis: 1H NMR
Solvent: CDCl3
Field strength: 400 MHz
8.1259 8.1224 8.1063 8.1019 7.6030 7.5996 7.5842 7.5807 7.5654 7.5619 7.4741 7.4703 7.4556 7.4532 7.4499 7.4349 7.4311 7.2657 7.2601 7.2471 7.2435
4.1907 4.0534 4.0378 4.0222
2.8343 2.8187 2.8030
1.0956 1.0835 1.0751 1.0689 1.0660 1.0508
1.00 1.14 1.06 2.24 2.16 2.18 2.24 31.98
f1 (ppm)

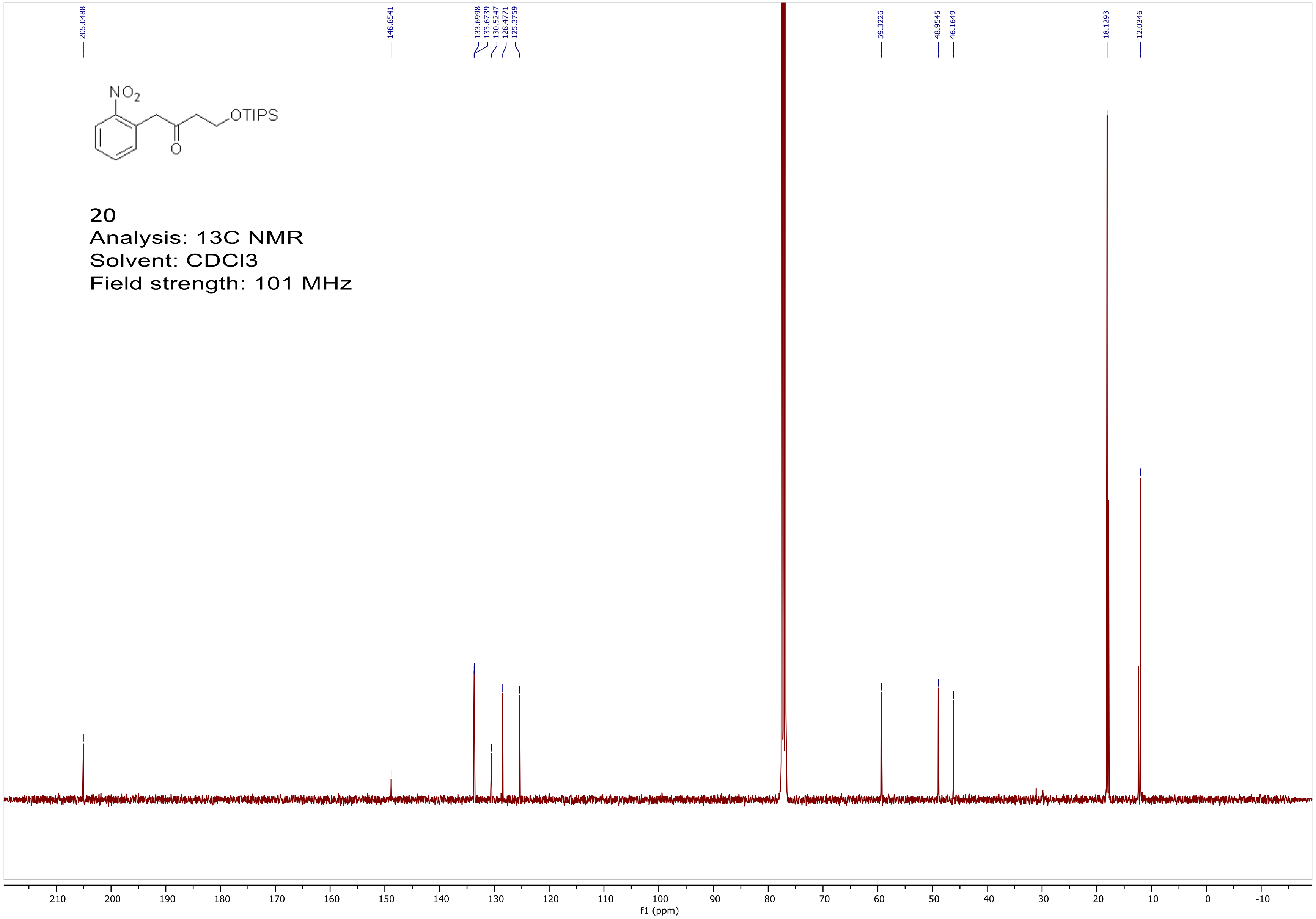

205.0488
148.8541
133.6998
133.6739
130.5247
128.4771
125.3759
59.3226
48.9545
46.1649
18.1293
12.0346
NO2
OTIPS
O
20
Analysis: 13C NMR
Solvent: CDCl3
Field strength: 101 MHz
210 200 190 180 170 160 150 140 130 120 110 100 90 80 70 60 50 40 30 20 10 0 -10
f1 (ppm)

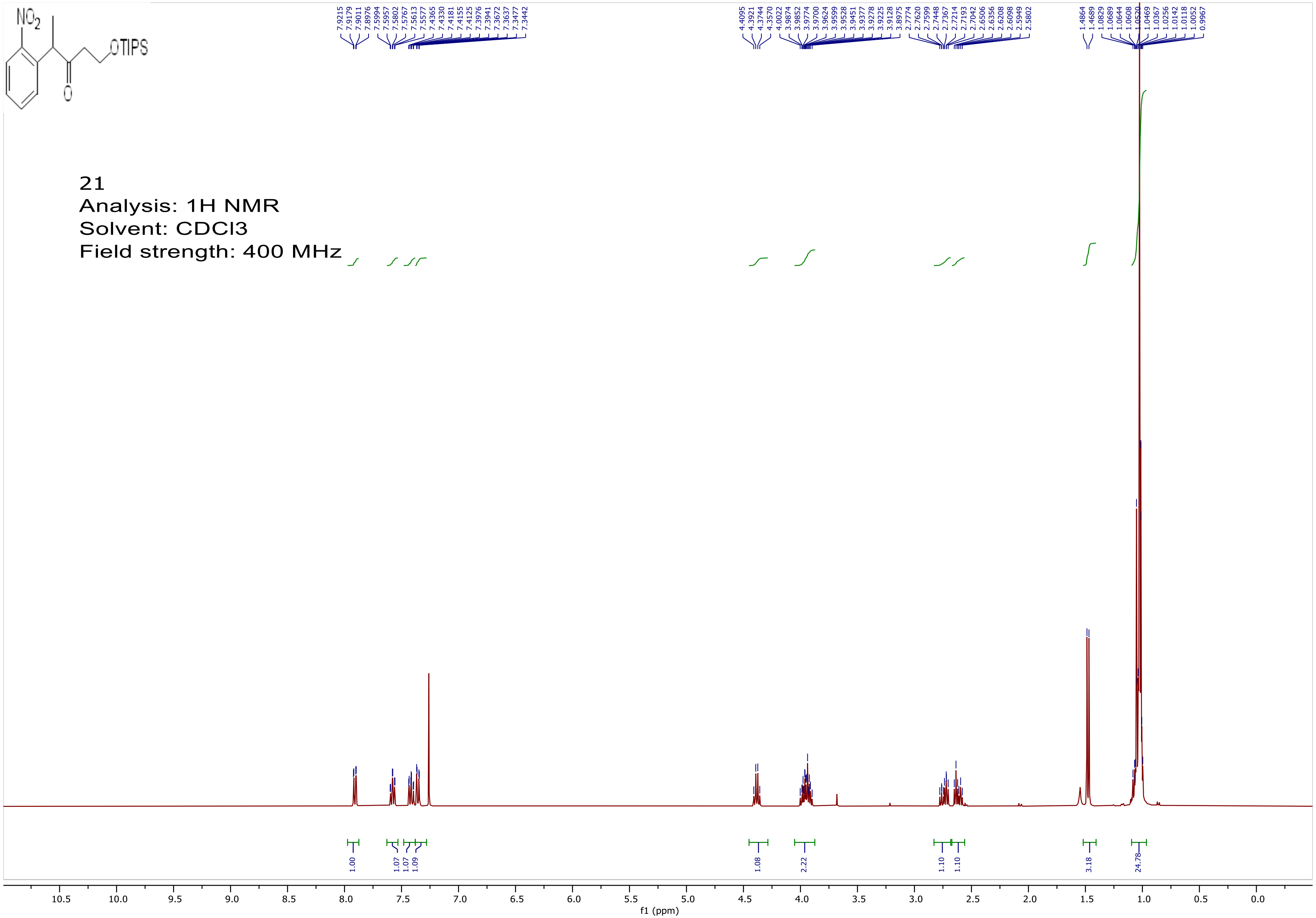
NO2
OTIPS
O
21
Analysis: 1H NMR
Solvent: CDCl3
Field strength: 400 MHz
7.9215 7.9179 7.9011 7.8976 7.5994 7.5957 7.5802 7.5767 7.5613 7.5577 7.4365 7.4330 7.4181 7.4155 7.4125 7.3976 7.3941 7.3672 7.3637 7.3477 7.3442
4.4095 4.3921 4.3744 4.3570 4.0022 3.9874 3.9852 3.9774 3.9700 3.9624 3.9599 3.9528 3.9451 3.9377 3.9278 3.9225 3.9128 3.8975 2.7774 2.7620 2.7599 2.7448 2.7367 2.7214 2.7193 2.7042 2.6506 2.6356 2.6208 2.6098 2.5949 2.5802
1.4864 1.4689 1.0829 1.0689 1.0644 1.0608 1.0520 1.0469 1.0367 1.0256 1.0142 1.0118 1.0052 0.9967
1.00 1.07 1.07 1.09 1.08 2.22 1.10 1.10 3.18 24.78
10.5 10.0 9.5 9.0 8.5 8.0 7.5 7.0 6.5 6.0 5.5 5.0 4.5 4.0 3.5 3.0 2.5 2.0 1.5 1.0 0.5 0.0
f1 (ppm)

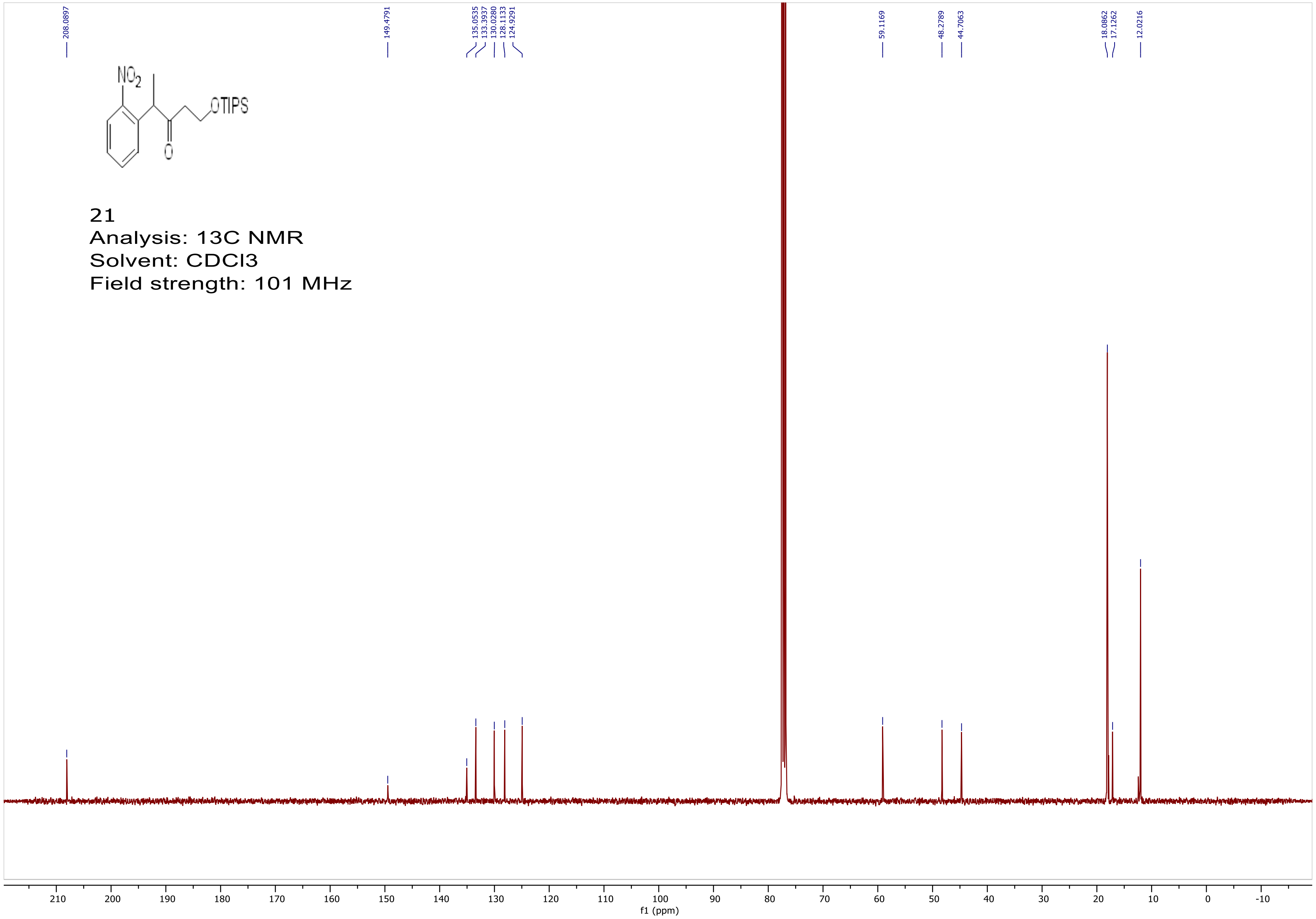

21
Analysis: 13C NMR
Solvent: CDCl3
Field strength: 101 MHz

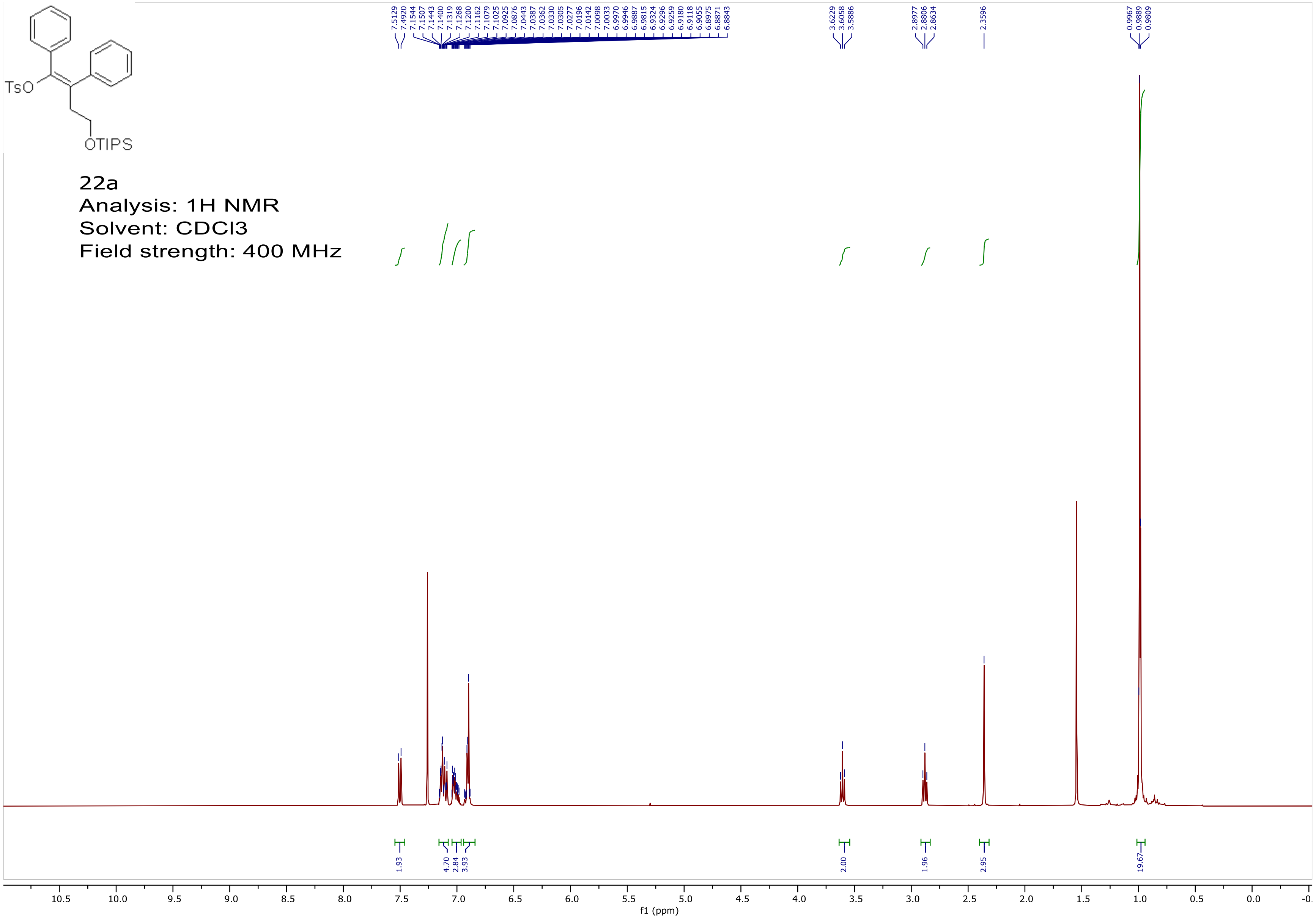
TsO
OTIPS
22a
Analysis: 1H NMR
Solvent: CDCl3
Field strength: 400 MHz
f1 (ppm)

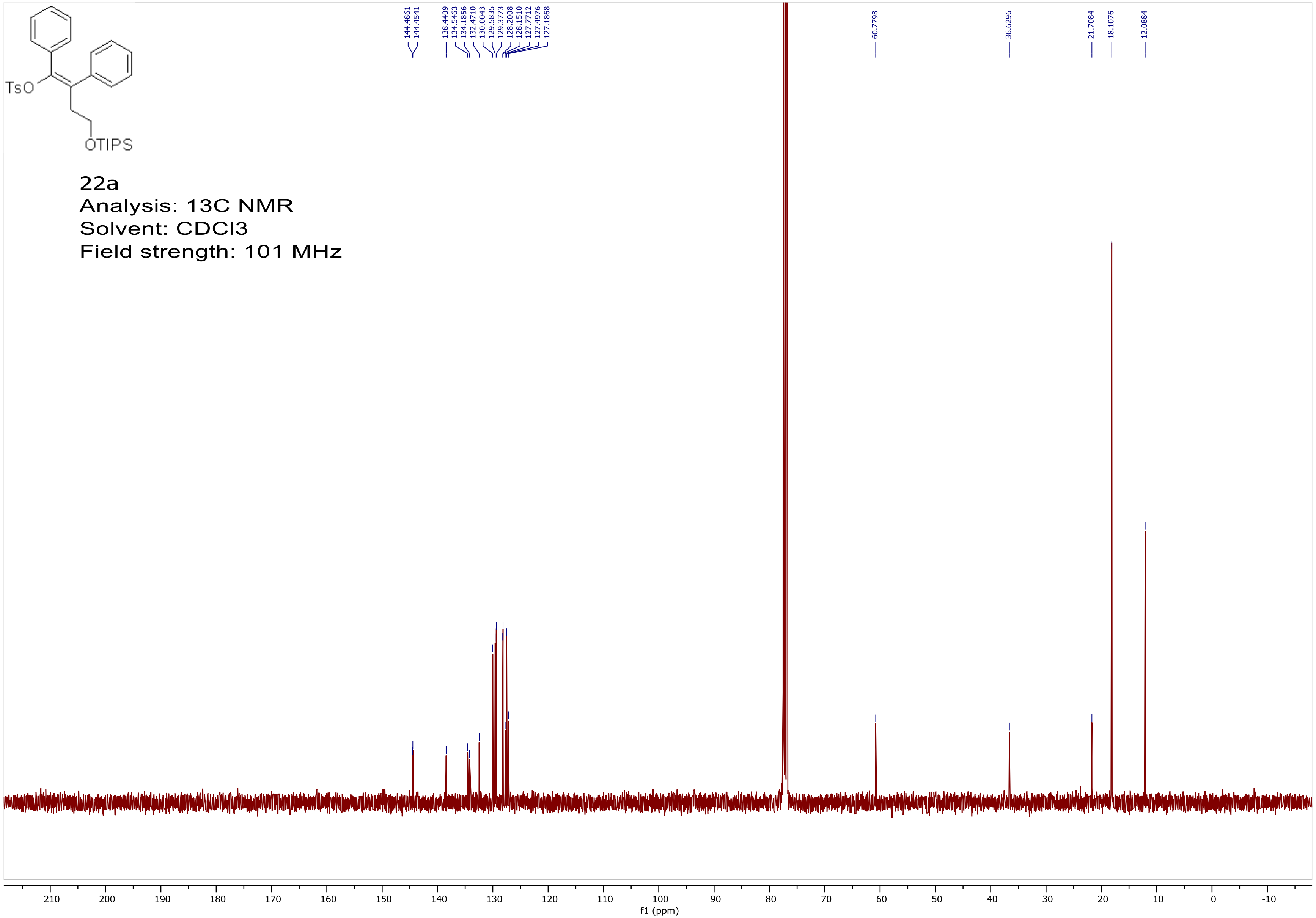
144.4861
144.4541
138.4409
134.5463
134.1856
132.4710
130.0043
129.5835
129.3773
128.2008
128.1510
127.7712
127.4976
127.1868
60.7798
36.6296
21.7084
18.1076
12.0884
TsO
OTIPS
22a
Analysis: 13C NMR
Solvent: CDCl3
Field strength: 101 MHz
210 200 190 180 170 160 150 140 130 120 110 100 90 80 70 60 50 40 30 20 10 0 -10
f1 (ppm)

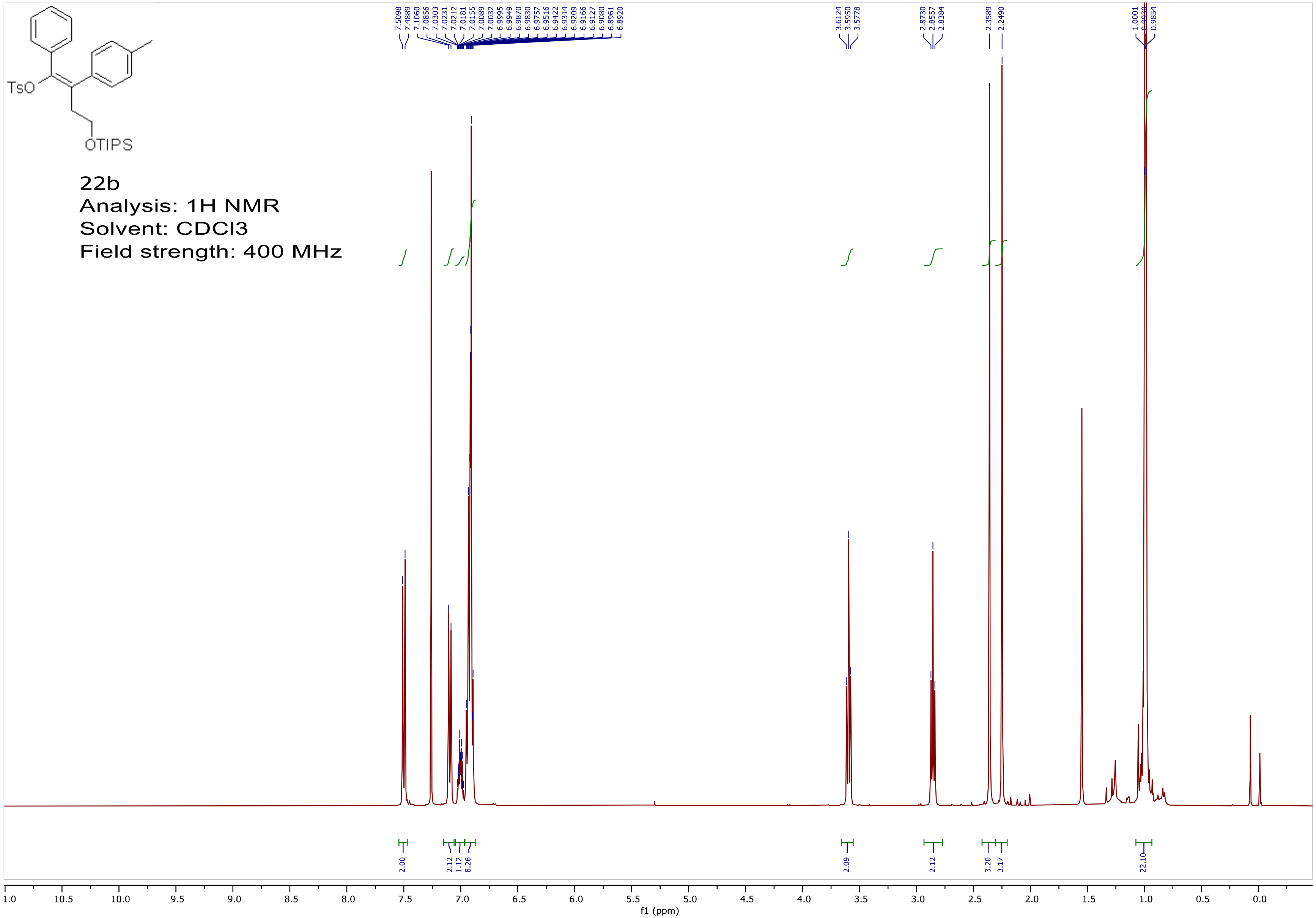
TsO
OTIPS
22b
Analysis: 1H NMR
Solvent: CDCl3
Field strength: 400 MHz
7.5098
7.4889
7.1060
7.0856
7.0303
7.0231
7.0212
7.0181
7.0155
7.0089
7.0032
6.9995
6.9949
6.9870
6.9830
6.9757
6.9516
6.9422
6.9314
6.9209
6.9166
6.9127
6.9080
6.8961
6.8920
3.6124
3.5950
3.5778
2.8730
2.8557
2.8384
2.3589
2.2490
1.0001
0.9854
2.00
2.12
1.12
8.26
2.09
2.12
3.20
3.17
22.10
f1 (ppm)

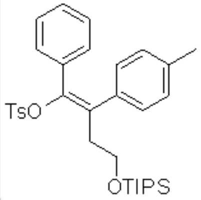

22b
Analysis: 13C NMR
Solvent: CDCl3
Field strength: 101 MHz

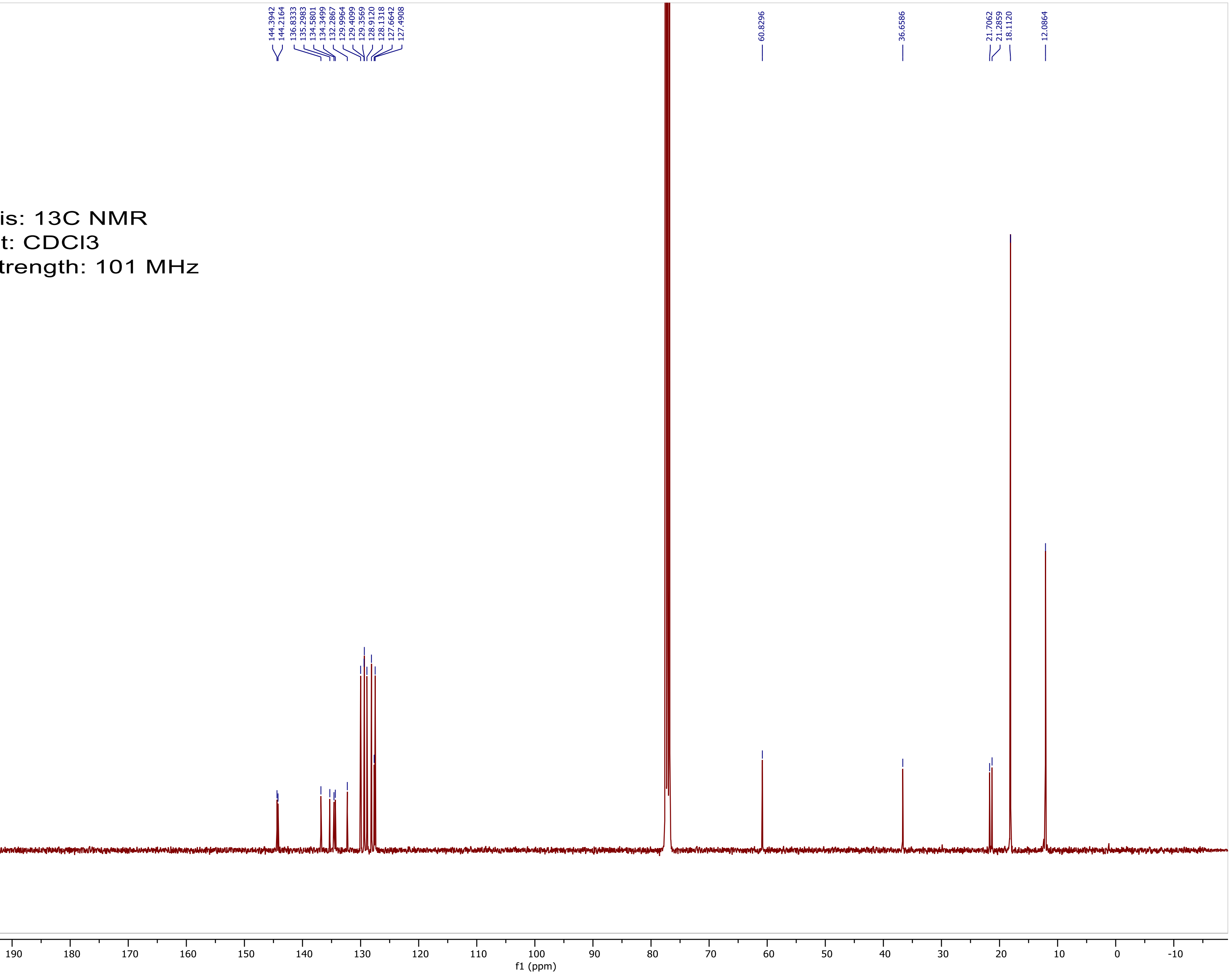

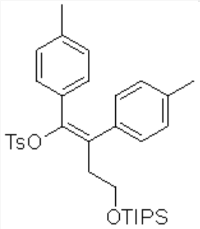

22c
Analysis: 1H NMR
Solvent: CDCl3
Field strength: 400 MHz

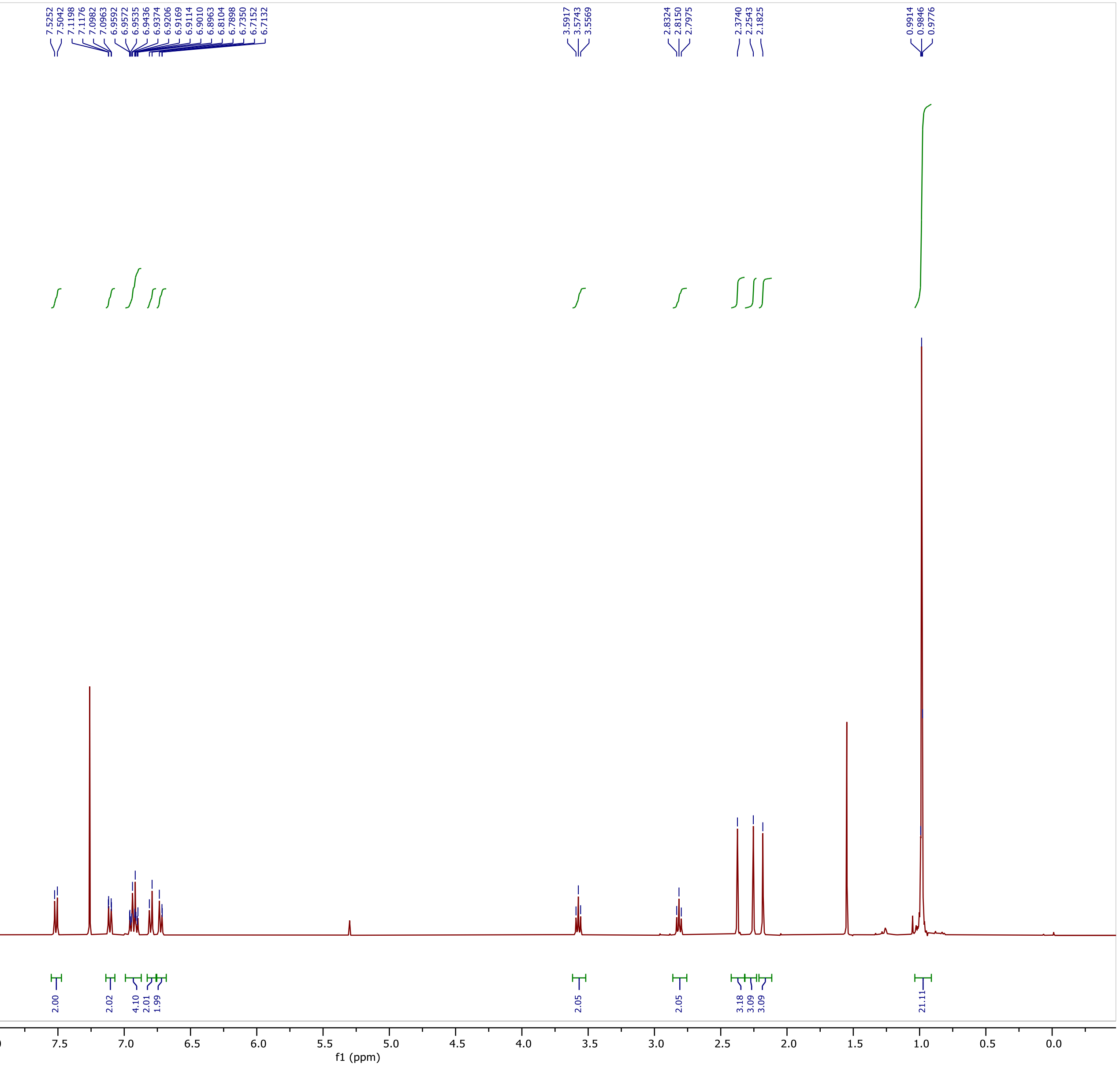

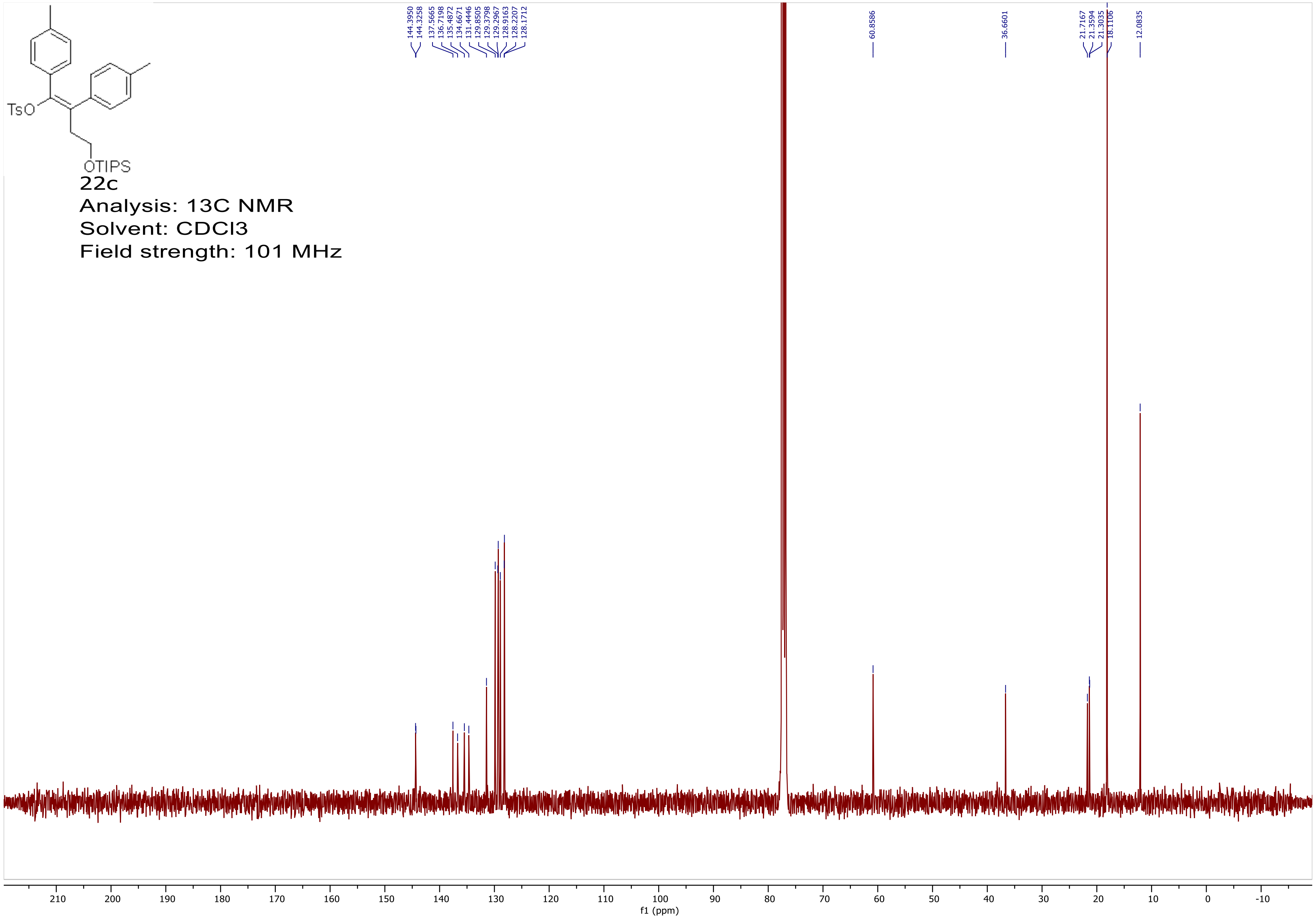
22c
Analysis: 13C NMR
Solvent: CDCl3
Field strength: 101 MHz
144.3950
144.3258
137.5665
136.7198
135.4872
134.6671
131.4446
129.8505
129.3798
129.2967
128.9163
128.2207
128.1712
60.8586
36.6601
21.7167
21.3594
21.3035
18.1106
12.0835
f1 (ppm)

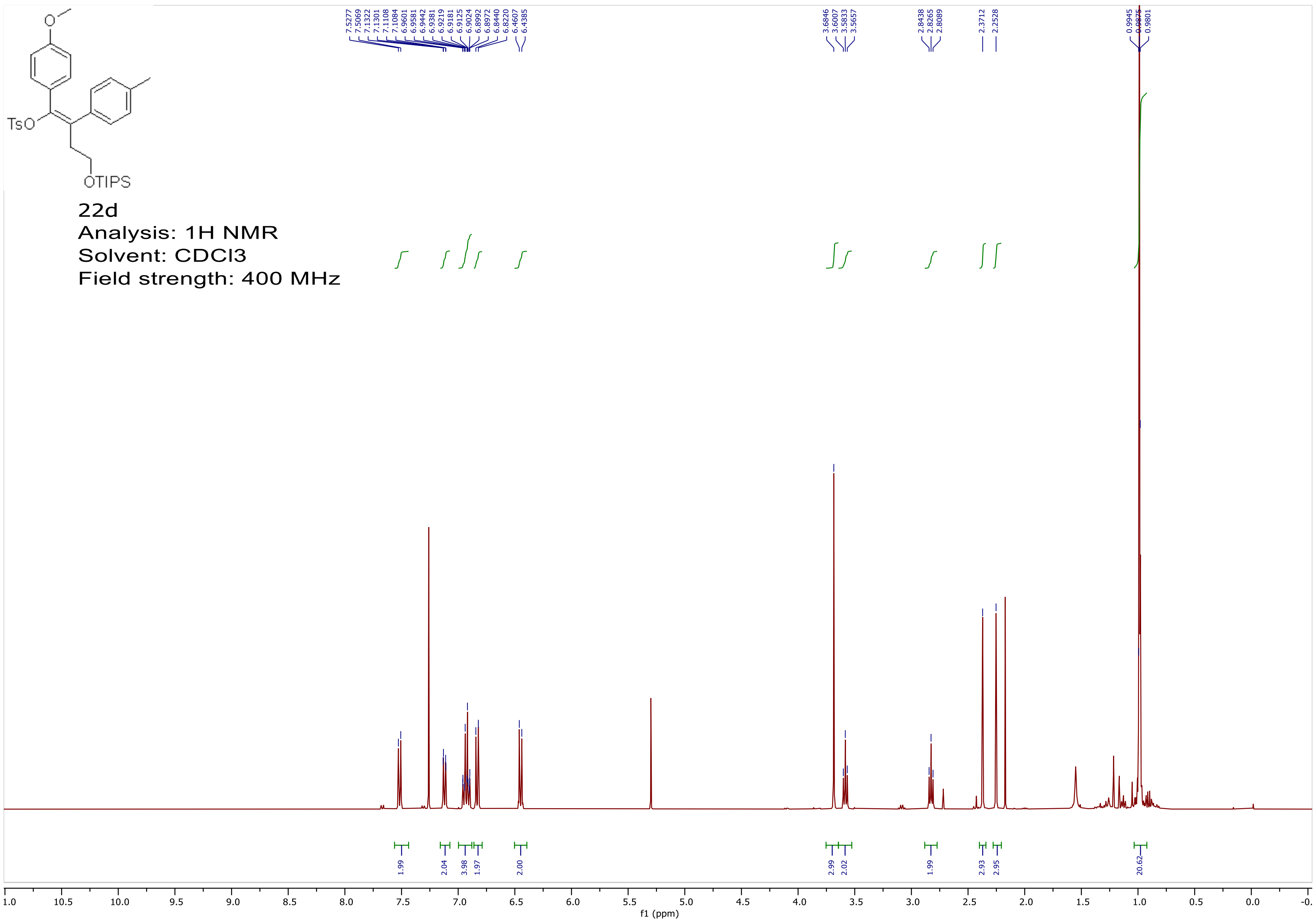
TsO
OTIPS
22d
Analysis: 1H NMR
Solvent: CDCl3
Field strength: 400 MHz
7.5277
7.5069
7.1322
7.1301
7.1108
7.1084
6.9601
6.9581
6.9442
6.9381
6.9219
6.9181
6.9125
6.9024
6.8992
6.8972
6.8440
6.8220
6.4607
6.4385
3.6846
3.6007
3.5833
3.5657
2.8438
2.8265
2.8089
2.3712
2.2528
0.9945
0.9801
1.99
2.04
3.98
1.97
2.00
2.99
2.02
1.99
2.93
2.95
20.62
f1 (ppm)

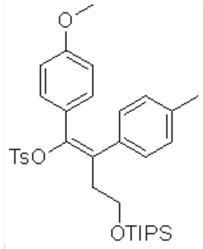

22d
Analysis: 13C NMR
Solvent: CDCl3
Field strength: 101 MHz

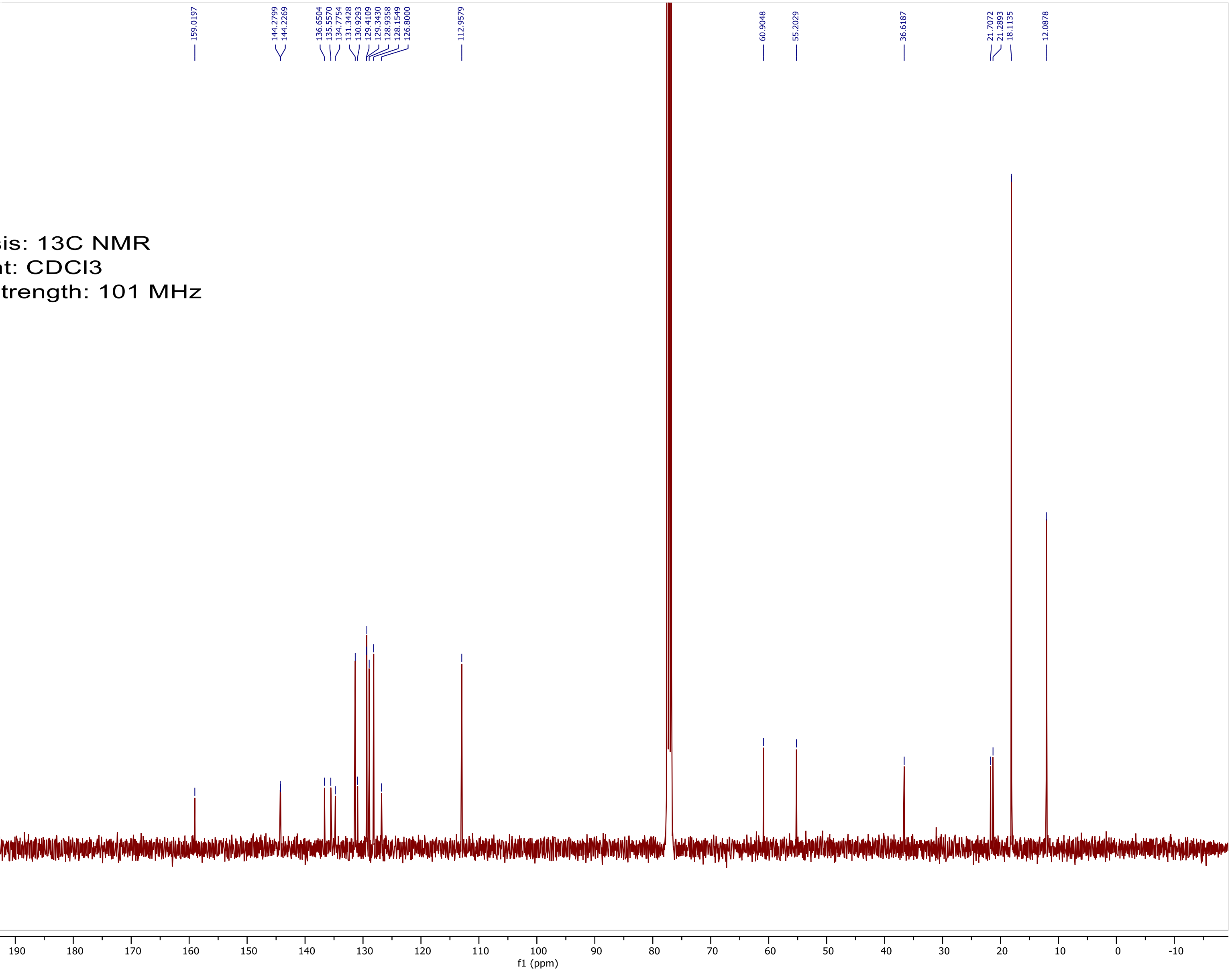

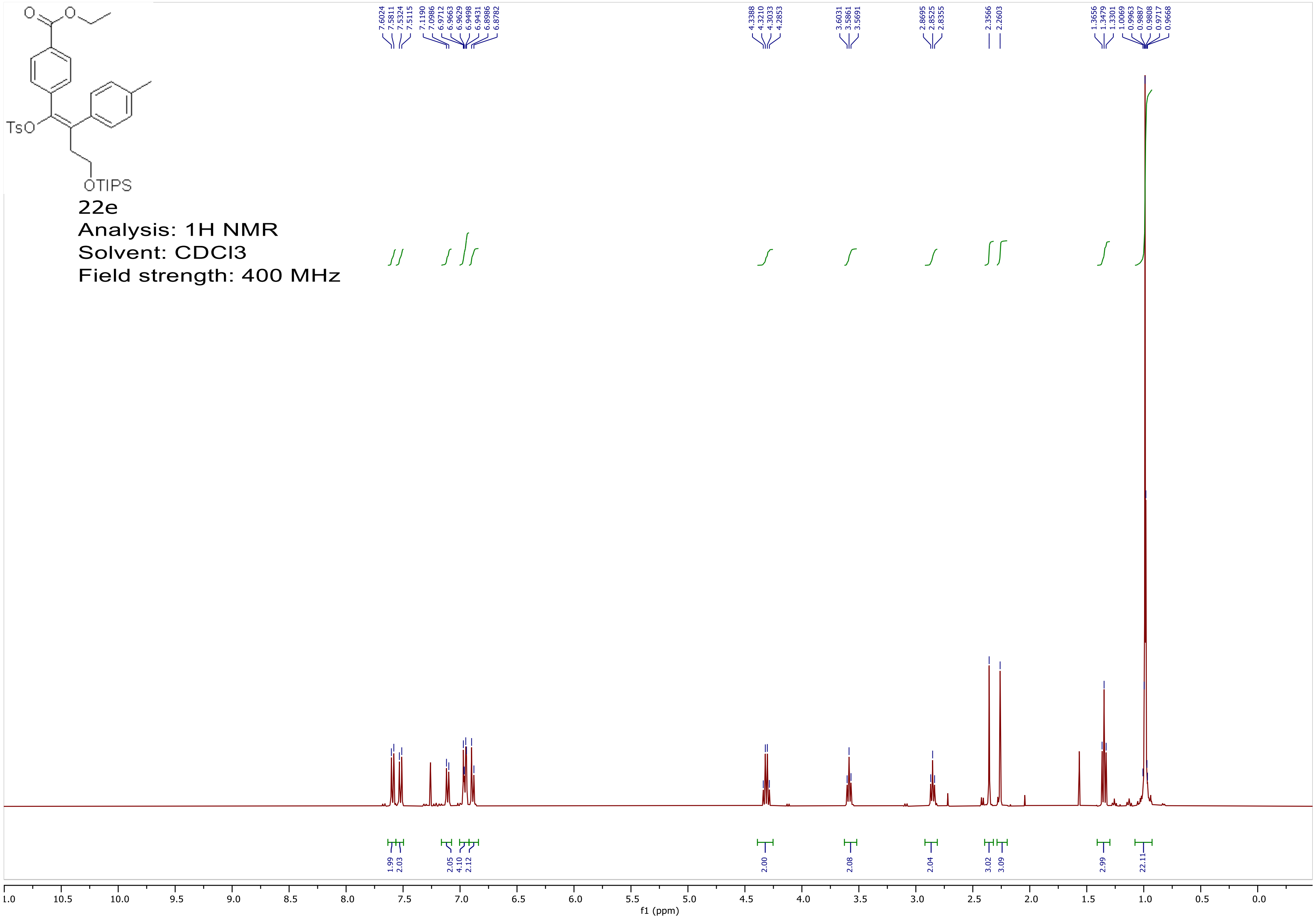

O
O
TsO
OTIPS
22e
Analysis: 1H NMR
Solvent: CDCl3
Field strength: 400 MHz
7.6024
7.5811
7.5324
7.5115
7.1190
7.0986
6.9712
6.9663
6.9629
6.9498
6.9431
6.8986
6.8782
4.3388
4.3210
4.3033
4.2853
3.6031
3.5861
3.5691
2.8695
2.8525
2.8355
2.3566
2.2603
1.3656
1.3479
1.3301
1.0069
0.9963
0.9887
0.9808
0.9717
0.9668
1.99
2.03
2.05
4.10
2.12
2.00
2.08
2.04
3.02
3.09
2.99
22.11
f1 (ppm)

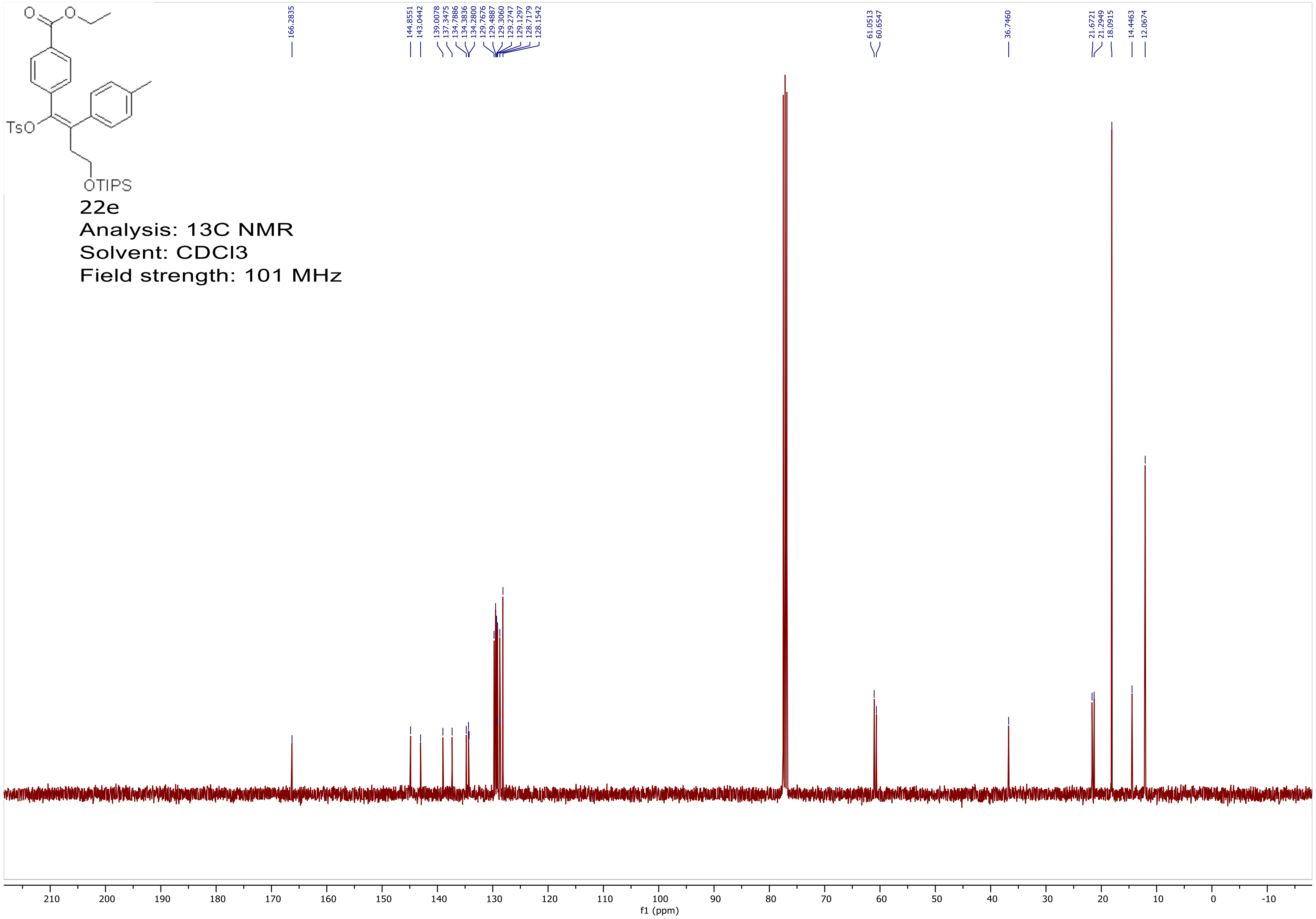
TsO
OTIPS
22e
Analysis: 13C NMR
Solvent: CDCl3
Field strength: 101 MHz
166.2835
144.8551
143.0442
139.0078
137.3475
134.7886
134.3836
134.2800
129.7676
129.4867
129.3060
129.2747
129.1297
128.7179
128.1542
61.0513
60.6547
36.7460
21.6721
21.2949
18.0915
14.4463
12.0674
210 200 190 180 170 160 150 140 130 120 110 100 90 80 70 60 50 40 30 20 10 0 -10
f1 (ppm)

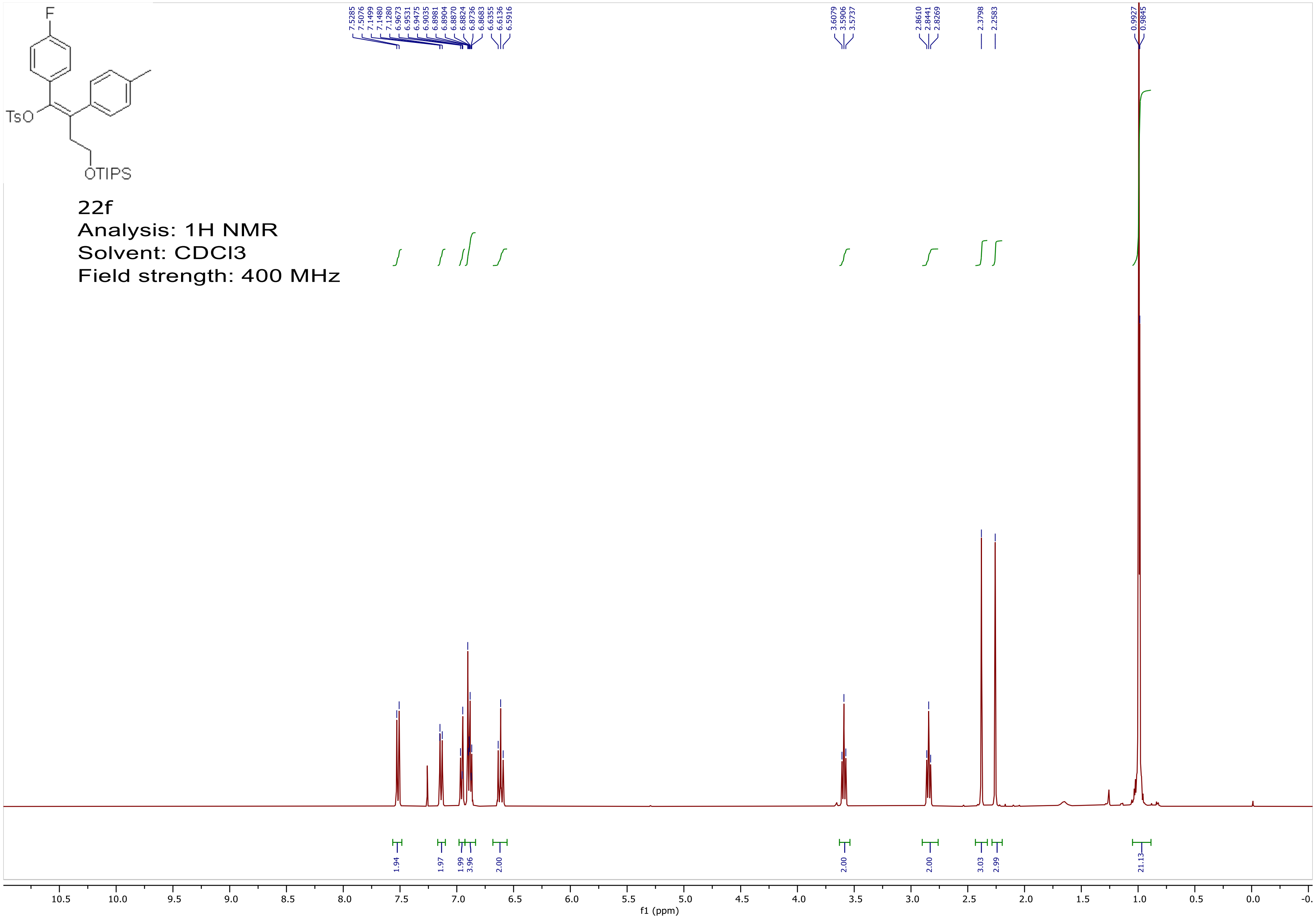

22f
Analysis: 1H NMR
Solvent: CDCl3
Field strength: 400 MHz

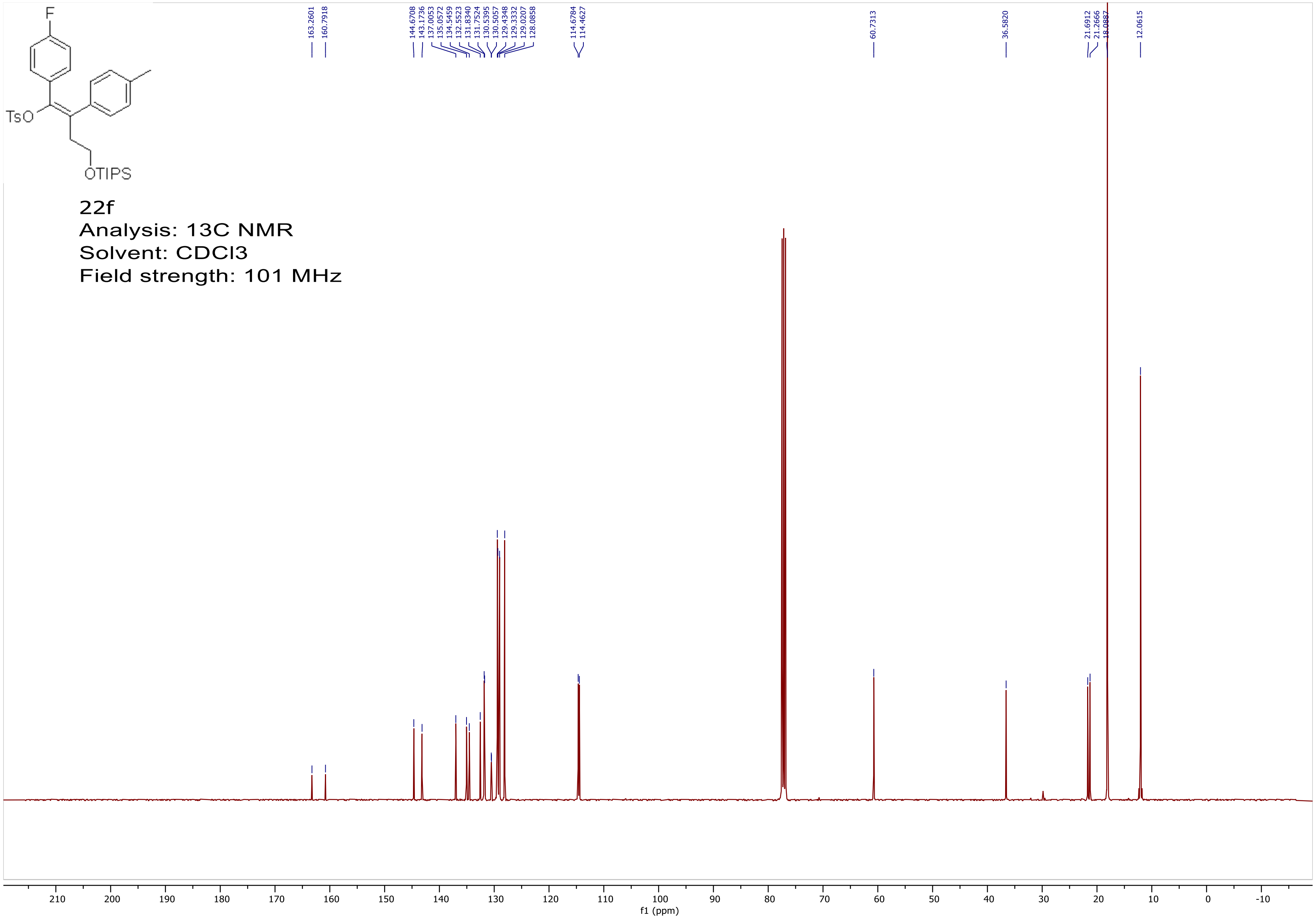

F
TsO
OTIPS
22f
Analysis: 13C NMR
Solvent: CDCl3
Field strength: 101 MHz
163.2601
160.7918
144.6708
143.1736
137.0053
135.0572
134.5459
132.5523
131.8340
131.7524
130.5395
130.5057
129.4348
129.3332
129.0207
128.0858
114.6784
114.4627
60.7313
36.5820
21.6912
21.2666
18.0887
12.0615
210 200 190 180 170 160 150 140 130 120 110 100 90 80 70 60 50 40 30 20 10 0 -10
f1 (ppm)

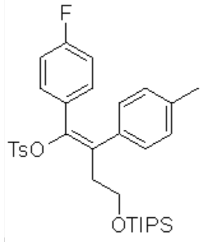

22f
Analysis: 19F NMR
Solvent: CDCl3
Field strength: 376 MHz

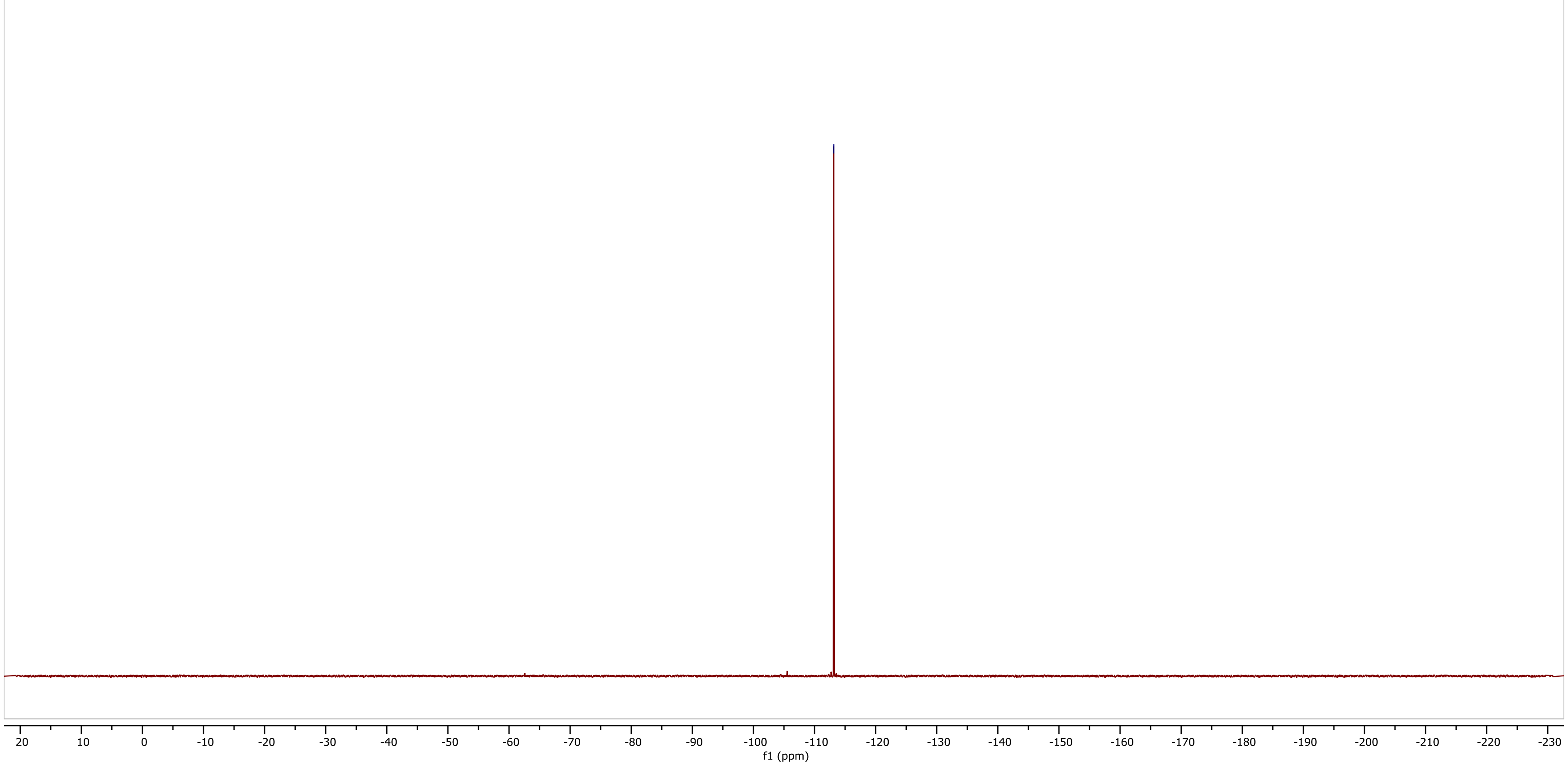

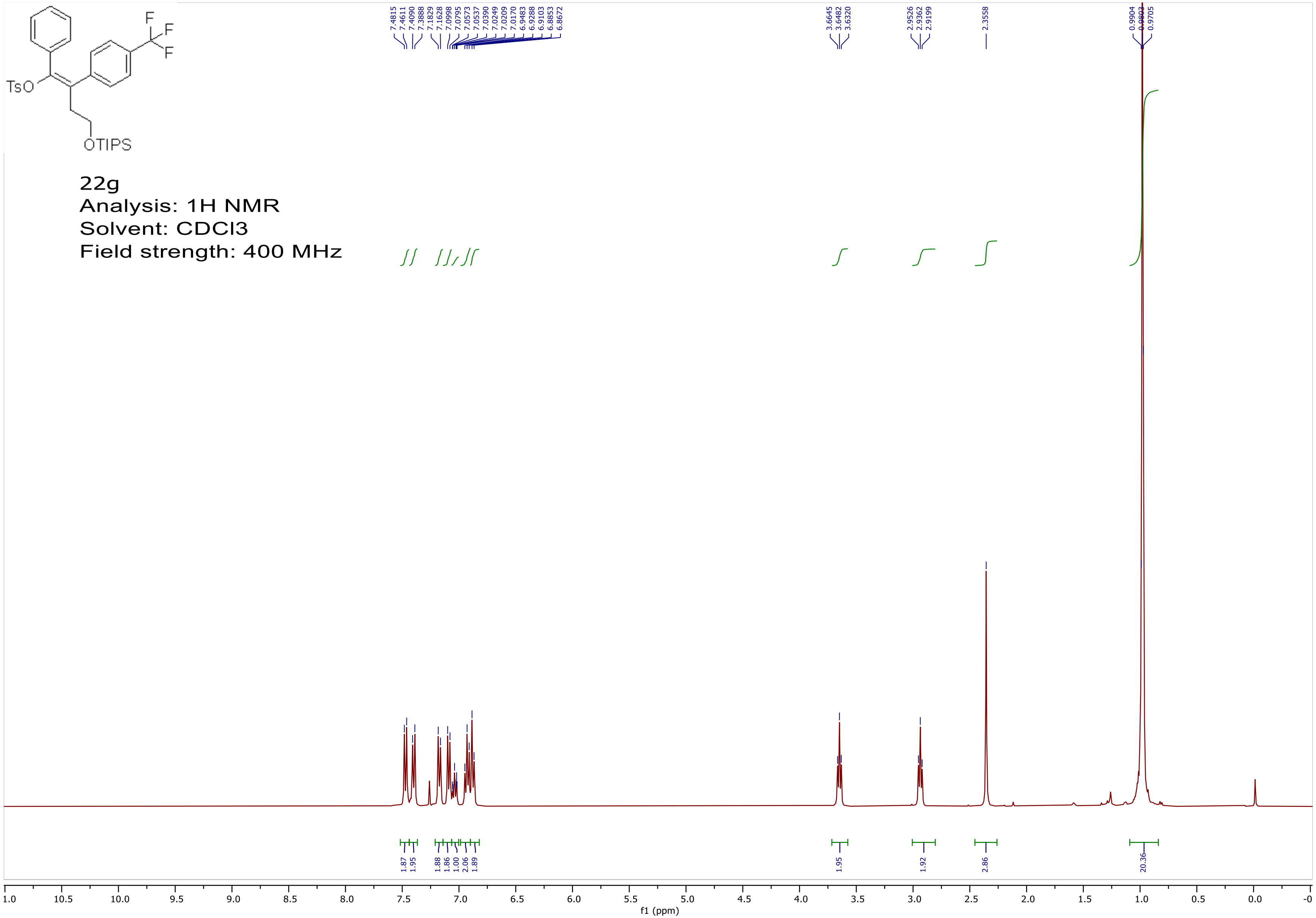

TsO
OTIPS
F
F
F
22g
Analysis: 1H NMR
Solvent: CDCl3
Field strength: 400 MHz
7.4815
7.4611
7.4090
7.3888
7.1829
7.1628
7.0998
7.0795
7.0573
7.0537
7.0390
7.0249
7.0209
7.0170
6.9483
6.9288
6.9103
6.8853
6.8672
3.6645
3.6482
3.6320
2.9526
2.9362
2.9199
2.3558
0.9904
0.9705
1.87
1.95
1.88
1.86
1.00
2.06
1.89
1.95
1.92
2.86
20.36
f1 (ppm)

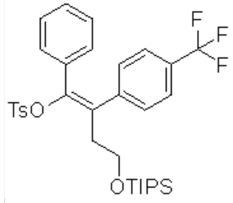

22g
Analysis: 13C NMR
Solvent: CDCl3
Field strength: 101 MHz

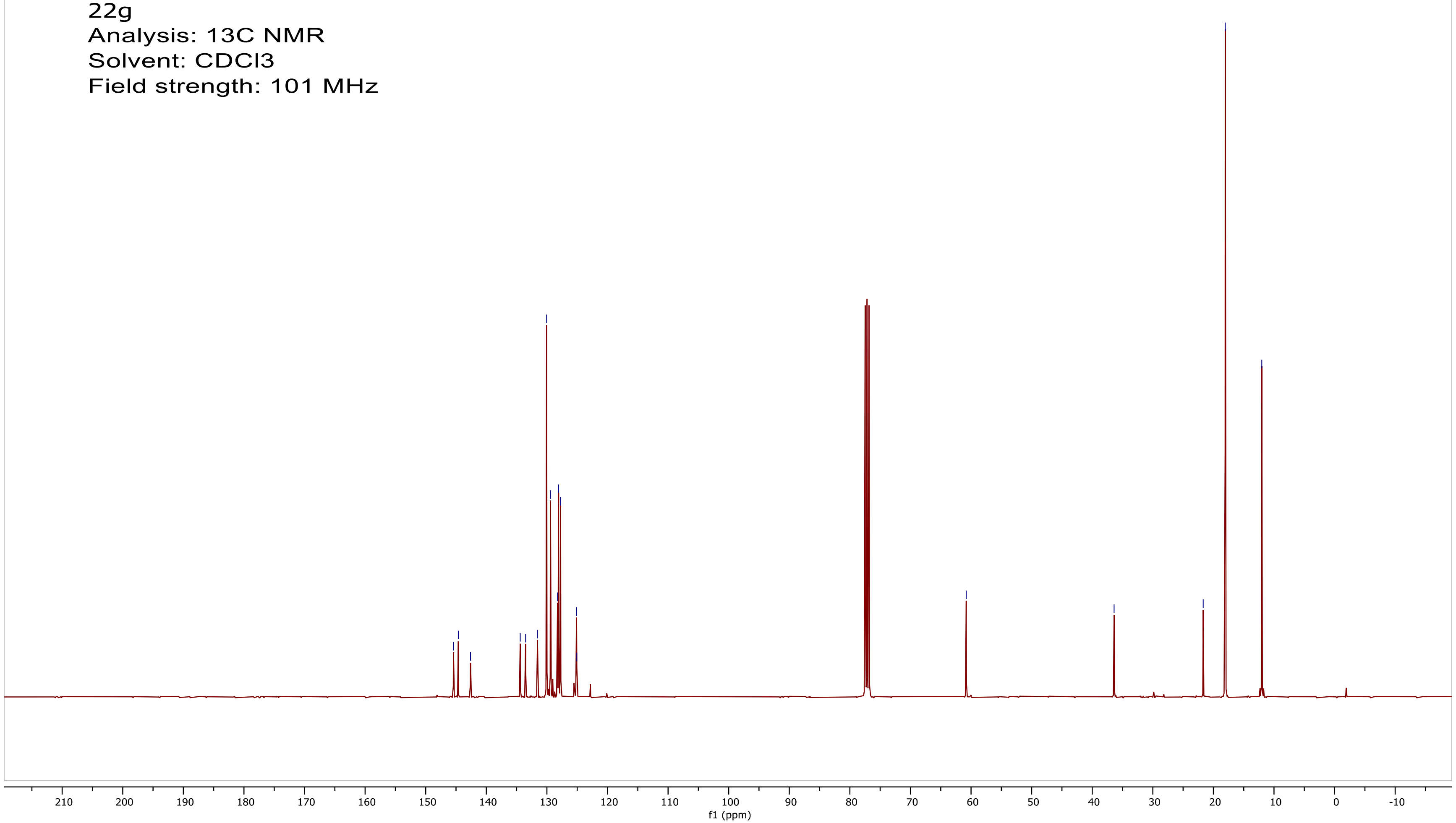

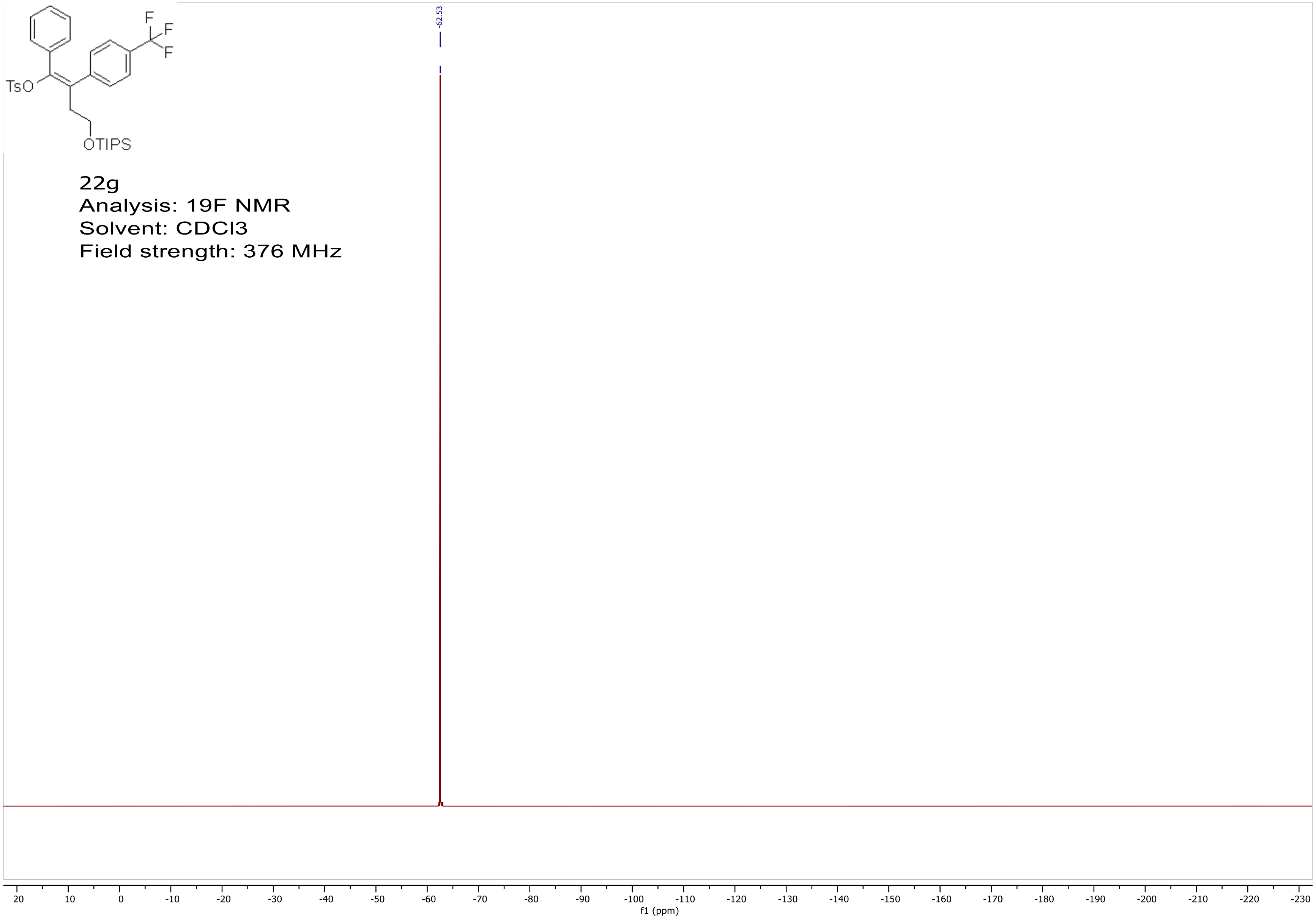
TsO
OTIPS
F
F
F
22g
Analysis: 19F NMR
Solvent: CDCl3
Field strength: 376 MHz
-62.53
20 10 0 -10 -20 -30 -40 -50 -60 -70 -80 -90 -100 -110 -120 -130 -140 -150 -160 -170 -180 -190 -200 -210 -220 -230
f1 (ppm)

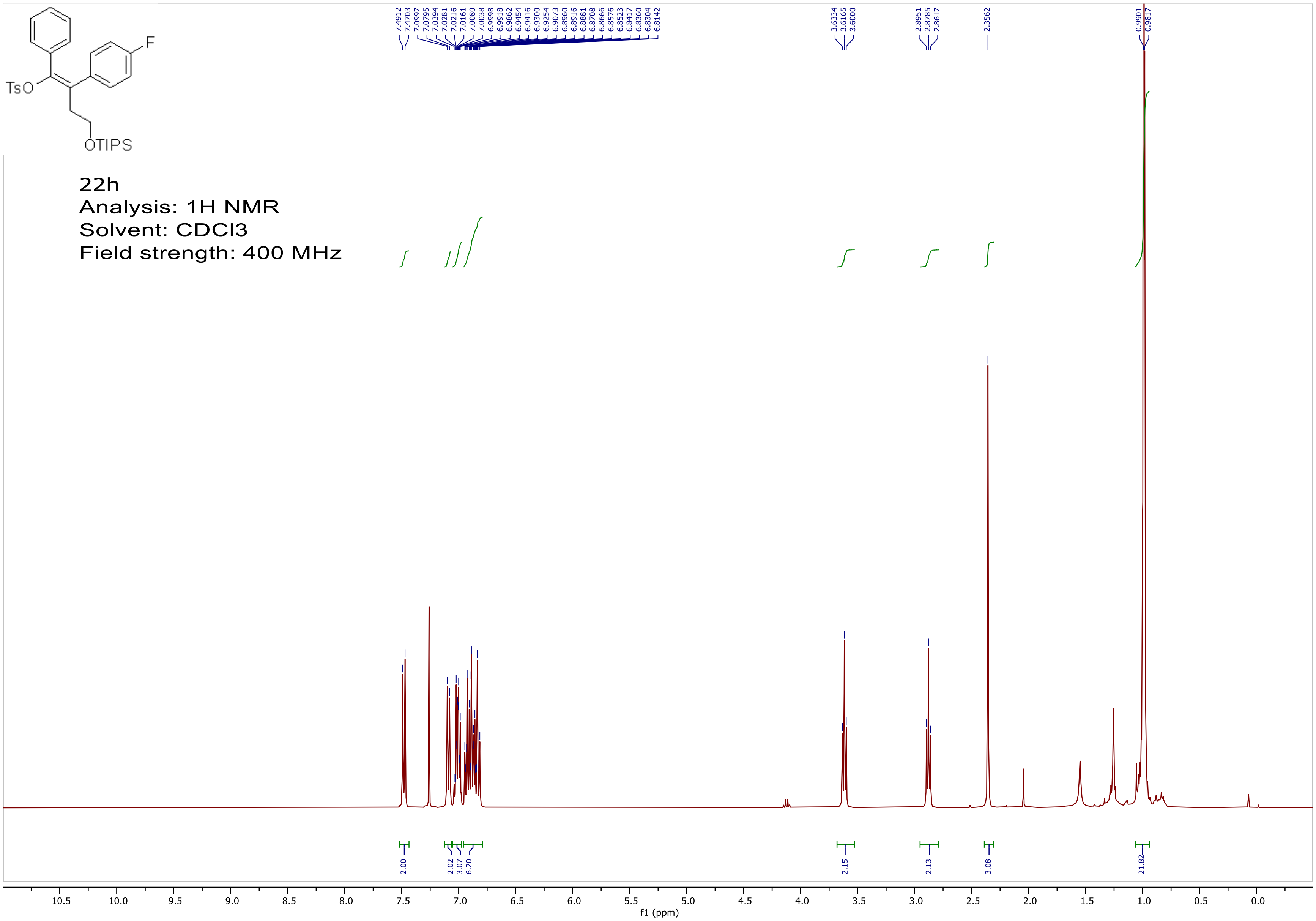
TsO
F
OTIPS
22h
Analysis: 1H NMR
Solvent: CDCl3
Field strength: 400 MHz
f1 (ppm)

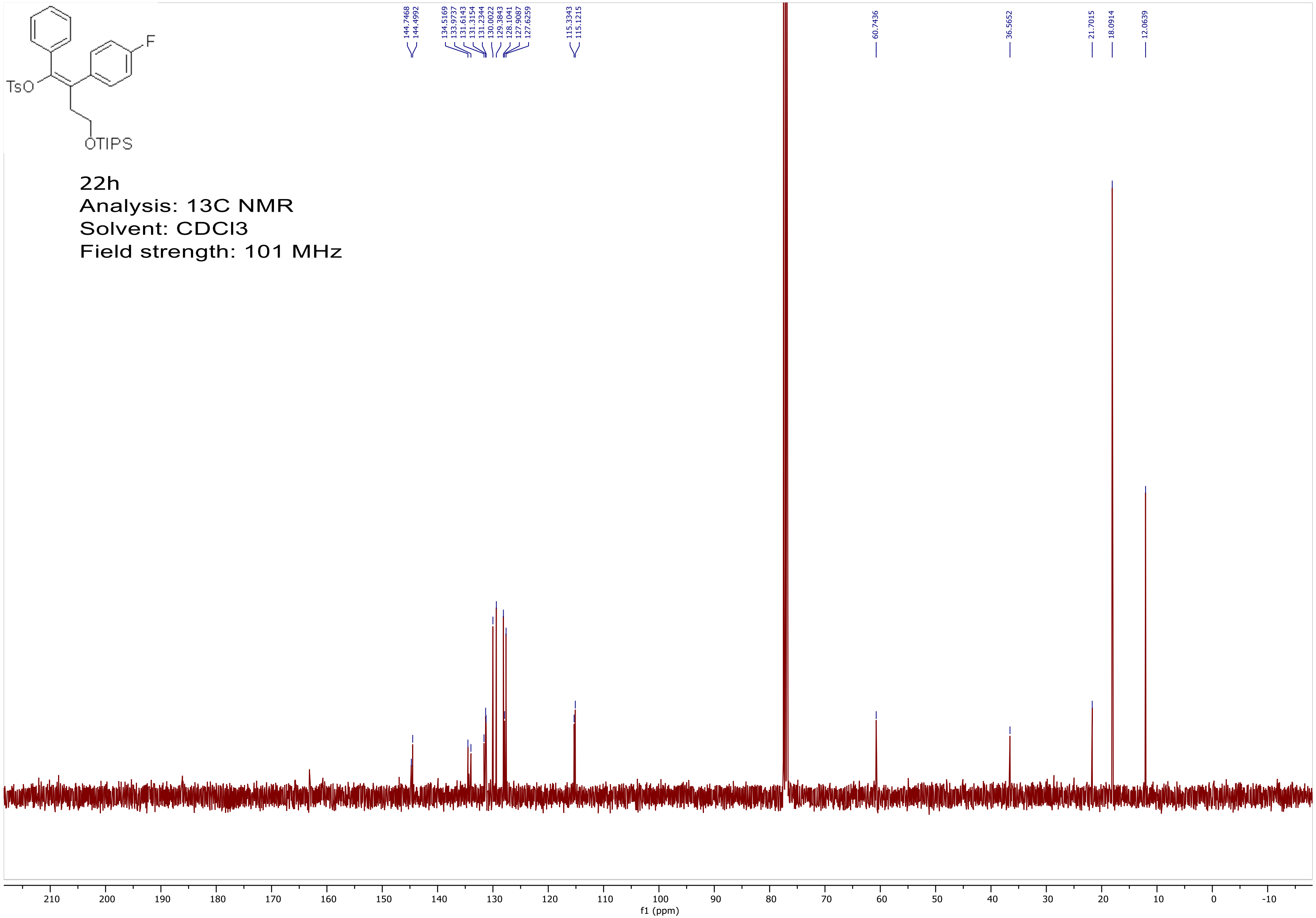

22h
Analysis: 13C NMR
Solvent: CDCl3
Field strength: 101 MHz

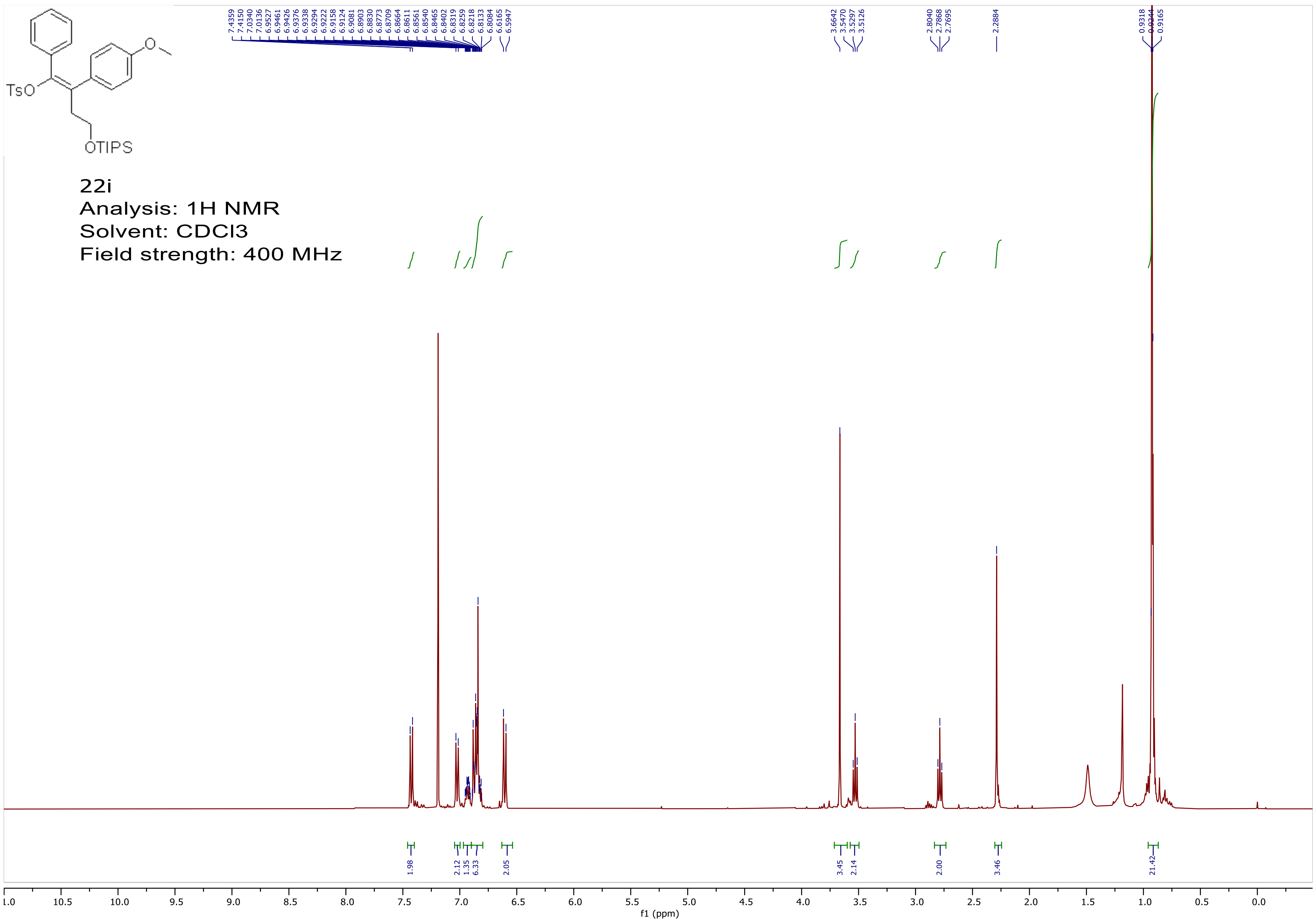

TsO
OTIPS
22i
Analysis: 1H NMR
Solvent: CDCl3
Field strength: 400 MHz
f1 (ppm)

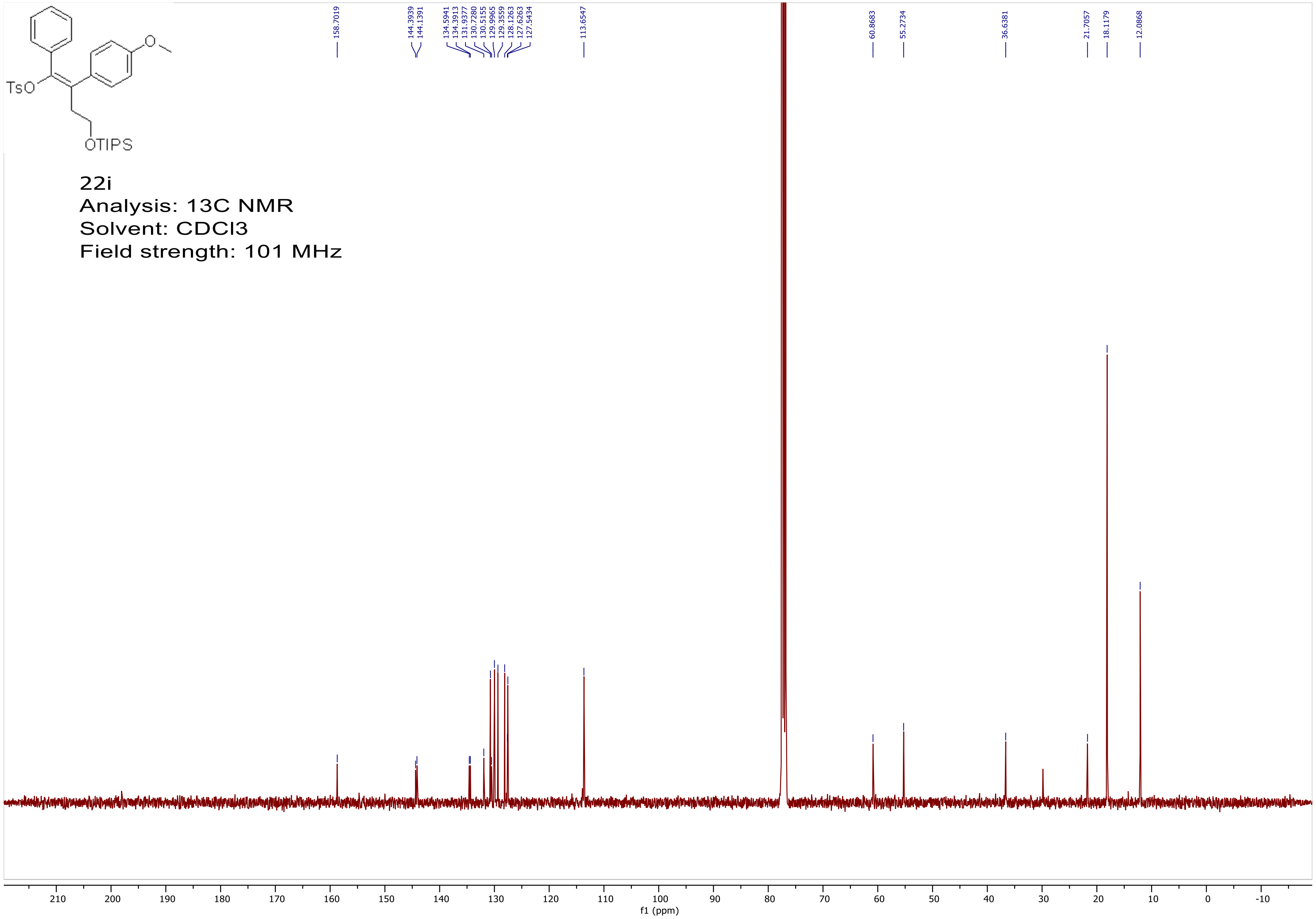

22i
Analysis: 13C NMR
Solvent: CDCl3
Field strength: 101 MHz

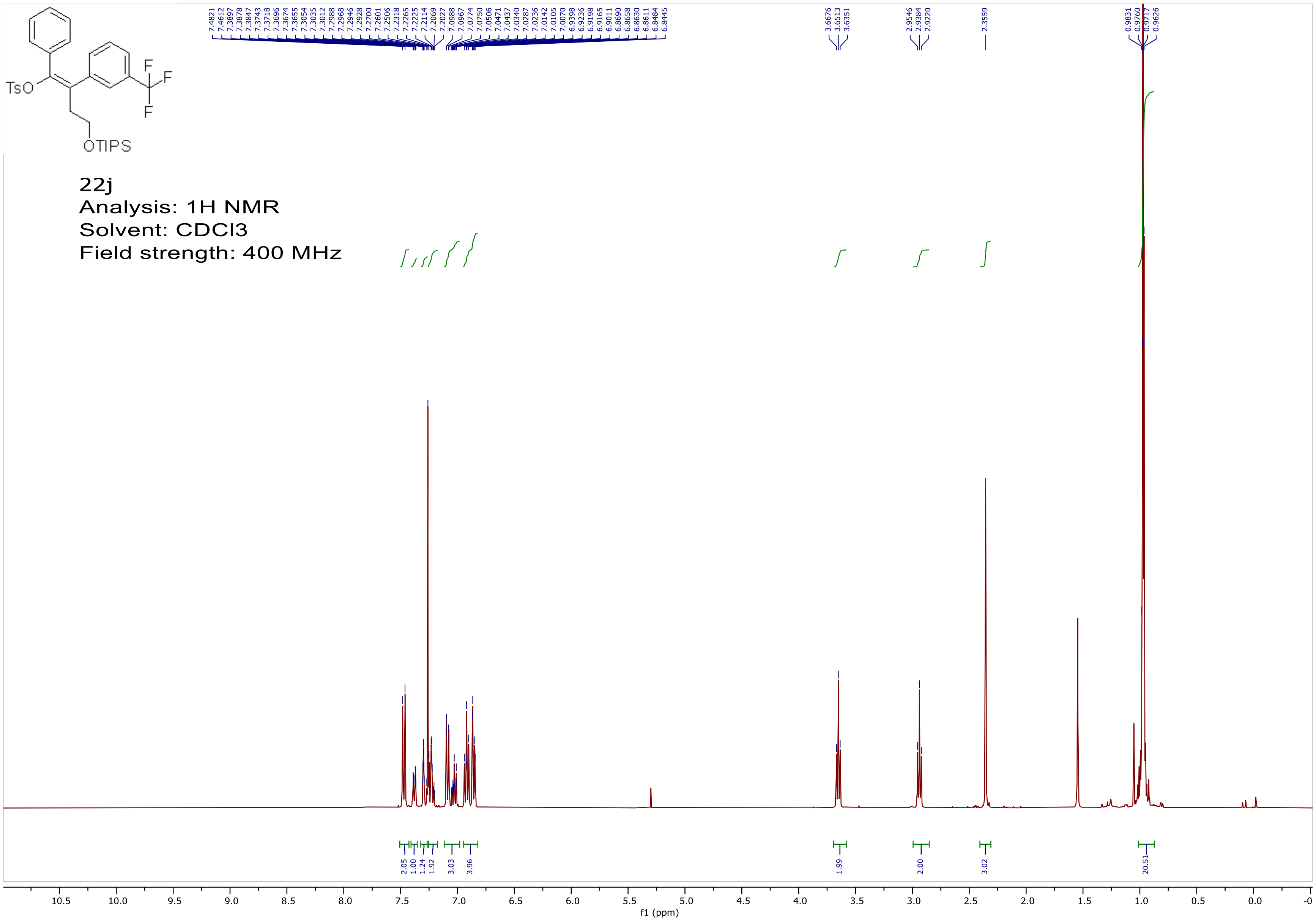
TsO
F
F
F
OTIPS
22j
Analysis: 1H NMR
Solvent: CDCl3
Field strength: 400 MHz
2.05
1.00
1.24
1.92
3.03
3.96
1.99
2.00
3.02
20.51
f1 (ppm)

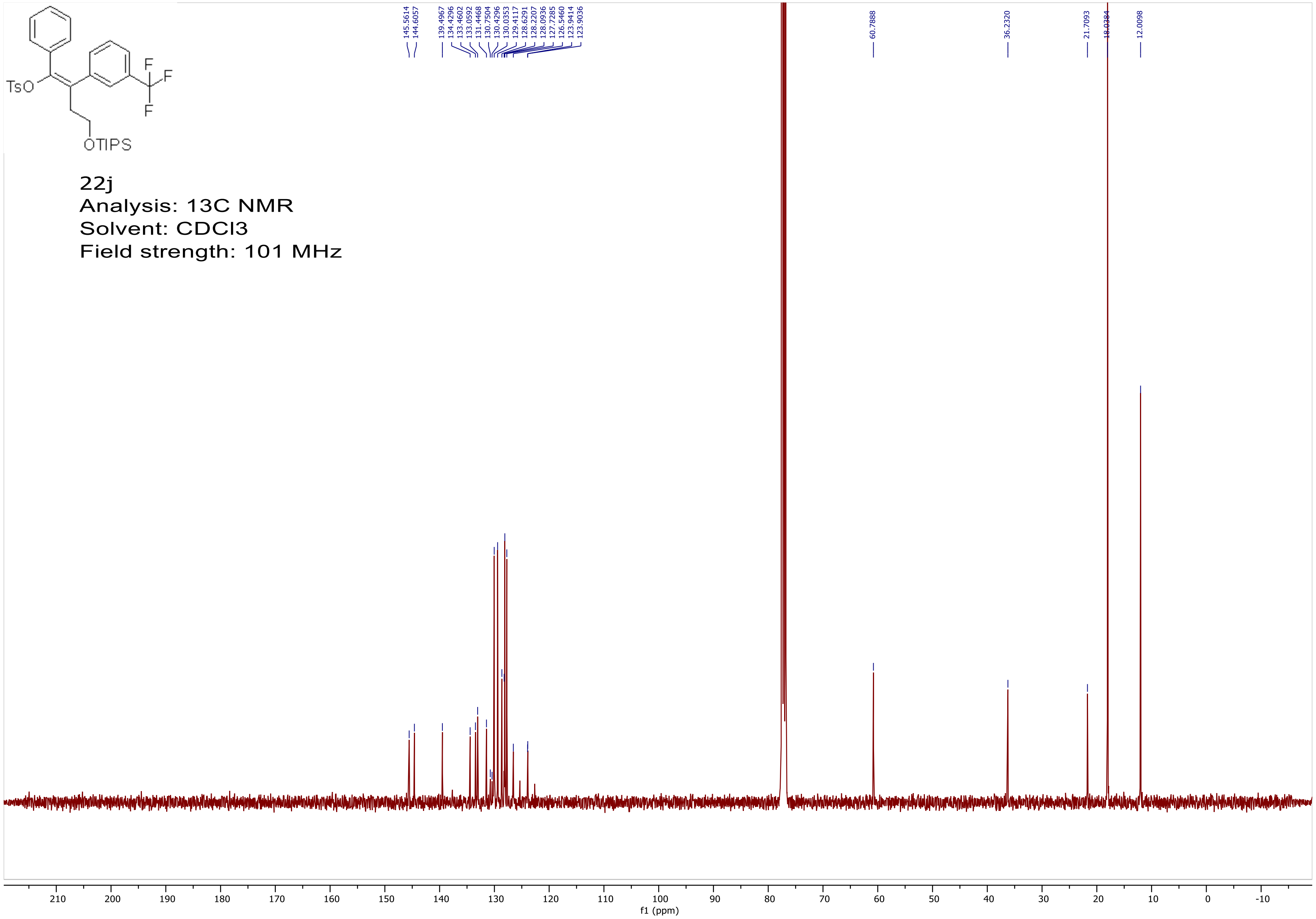

TsO
F
F
F
OTIPS
22j
Analysis: 13C NMR
Solvent: CDCl3
Field strength: 101 MHz
145.5614
144.6057
139.4967
134.4296
133.4602
133.0592
131.4468
130.7504
130.4296
130.0353
129.4117
128.6291
128.2207
128.0936
127.7285
126.5460
123.9414
123.9036
60.7888
36.2320
21.7093
18.0384
12.0098
210
200
190
180
170
160
150
140
130
120
110
100
90
80
70
60
50
40
30
20
10
0
-10
f1 (ppm)

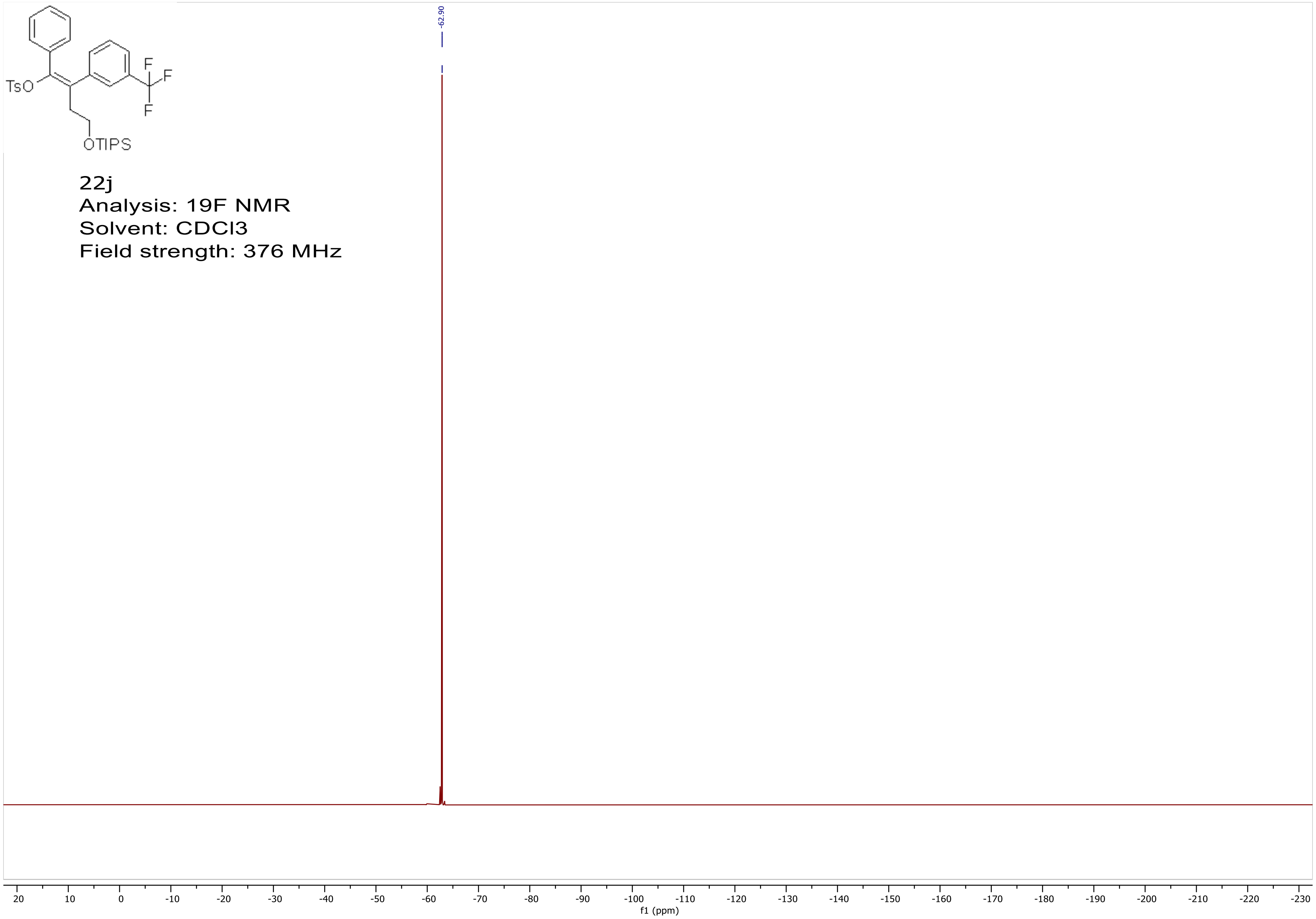
-62.90
TsO
F
F
F
OTIPS
22j
Analysis: 19F NMR
Solvent: CDCl3
Field strength: 376 MHz
20 10 0 -10 -20 -30 -40 -50 -60 -70 -80 -90 -100 -110 -120 -130 -140 -150 -160 -170 -180 -190 -200 -210 -220 -230
f1 (ppm)

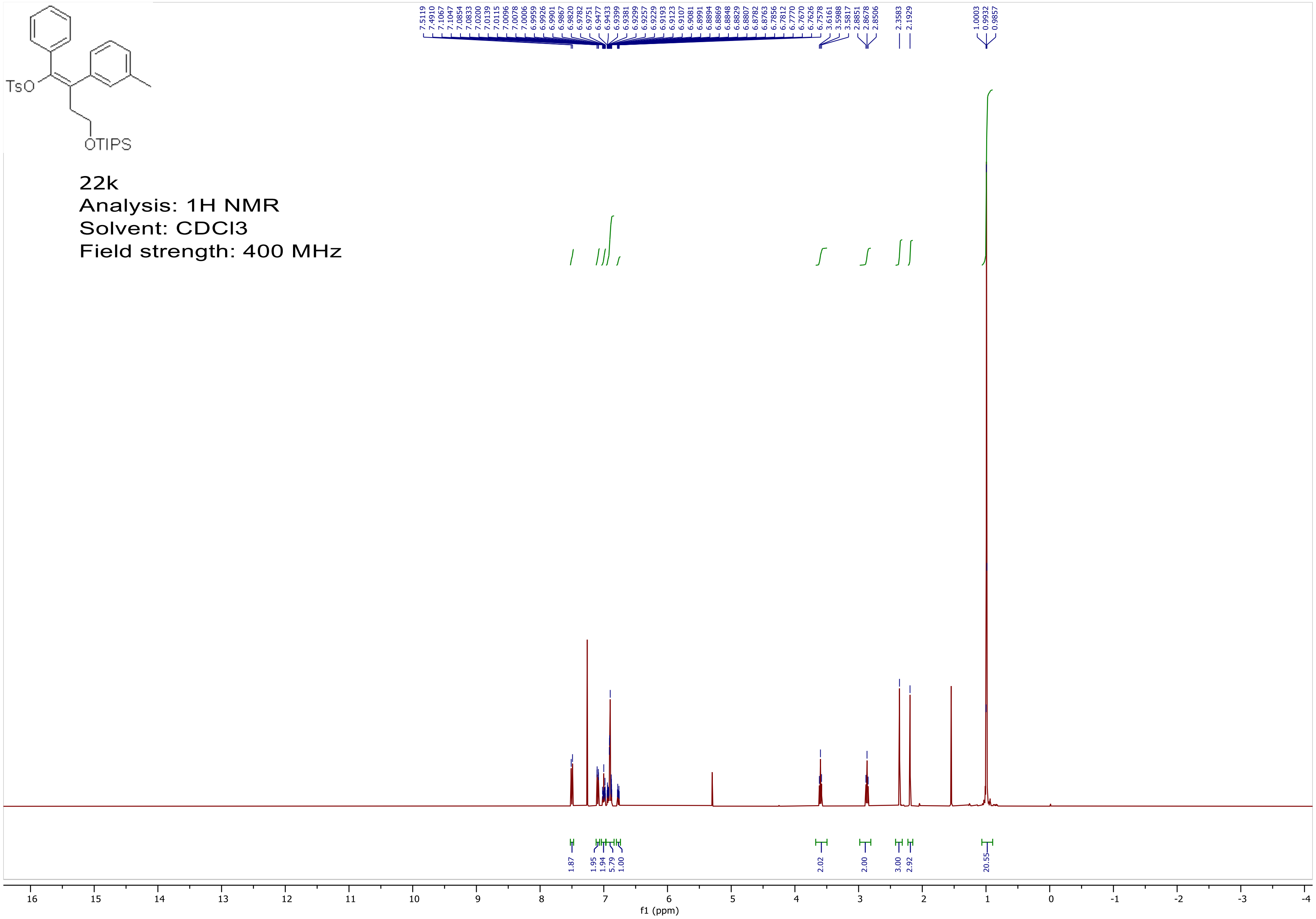
TsO
OTIPS
22k
Analysis: 1H NMR
Solvent: CDCl3
Field strength: 400 MHz
f1 (ppm)

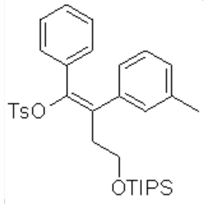

22k
Analysis: 13C NMR
Solvent: CDCl3
Field strength: 101 MHz

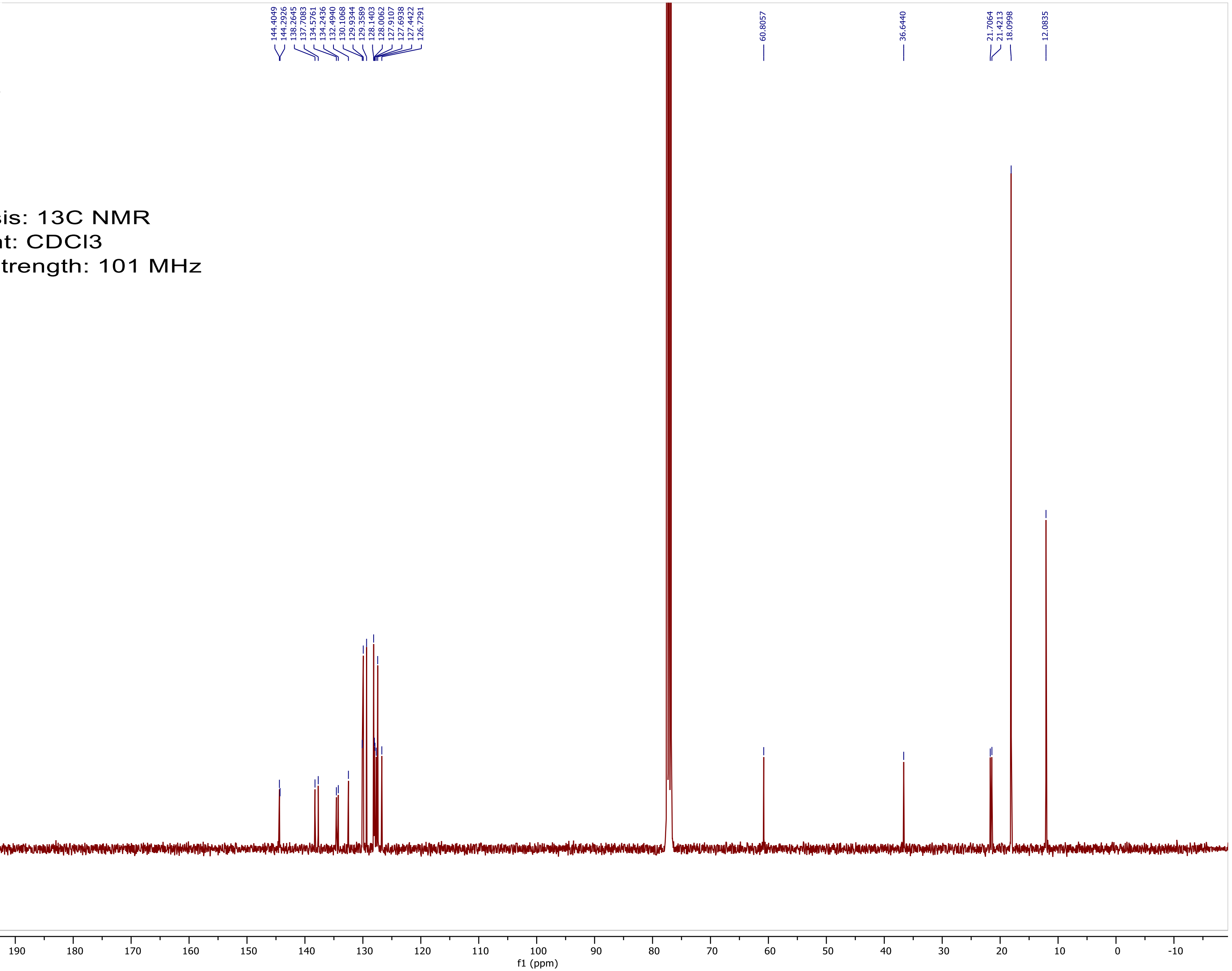

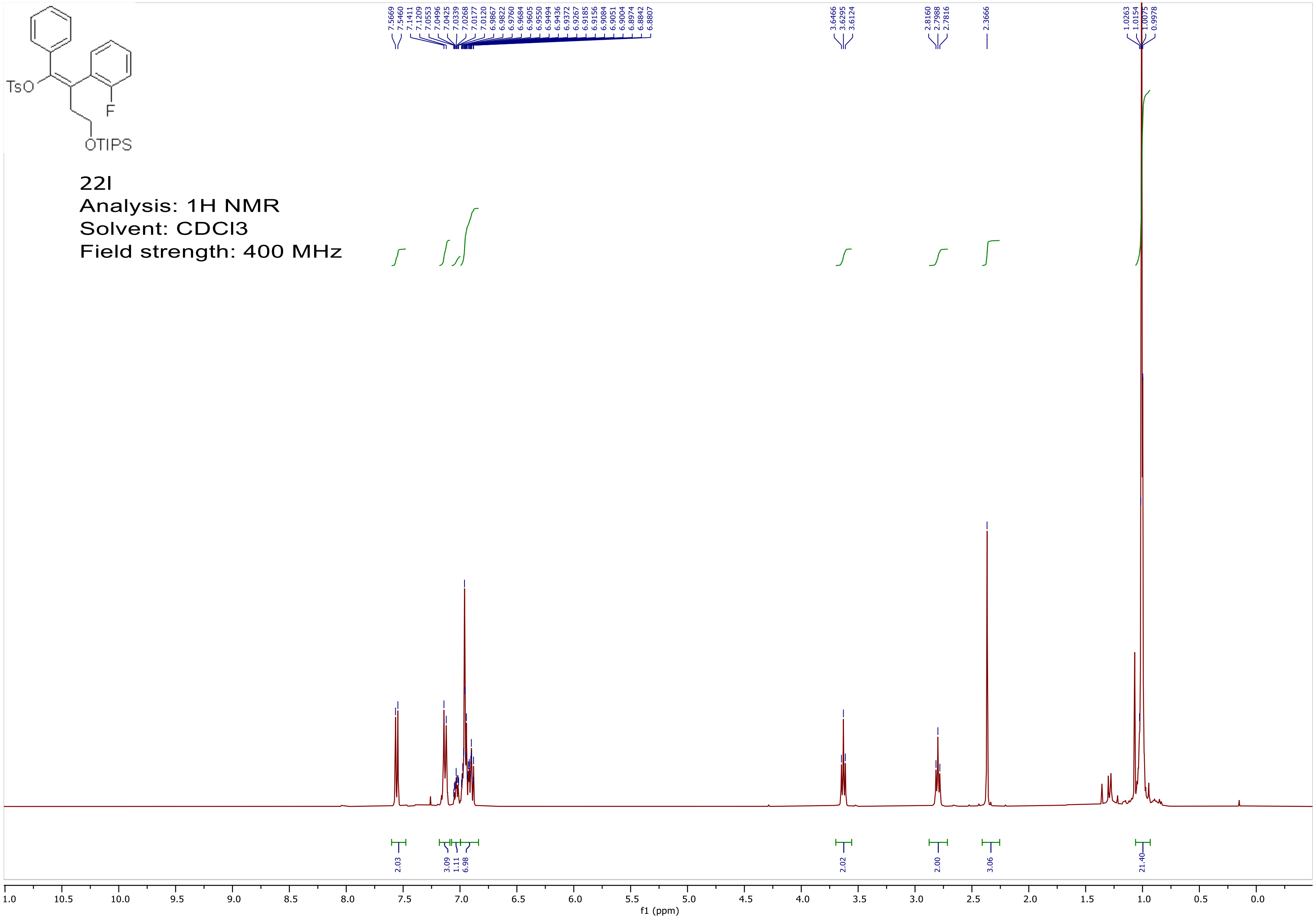
TsO
F
OTIPS
22l
Analysis: 1H NMR
Solvent: CDCl3
Field strength: 400 MHz
f1 (ppm)

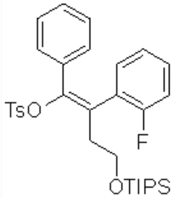

22l
Analysis: 13C NMR
Solvent: CDCl3
Field strength: 101 MHz

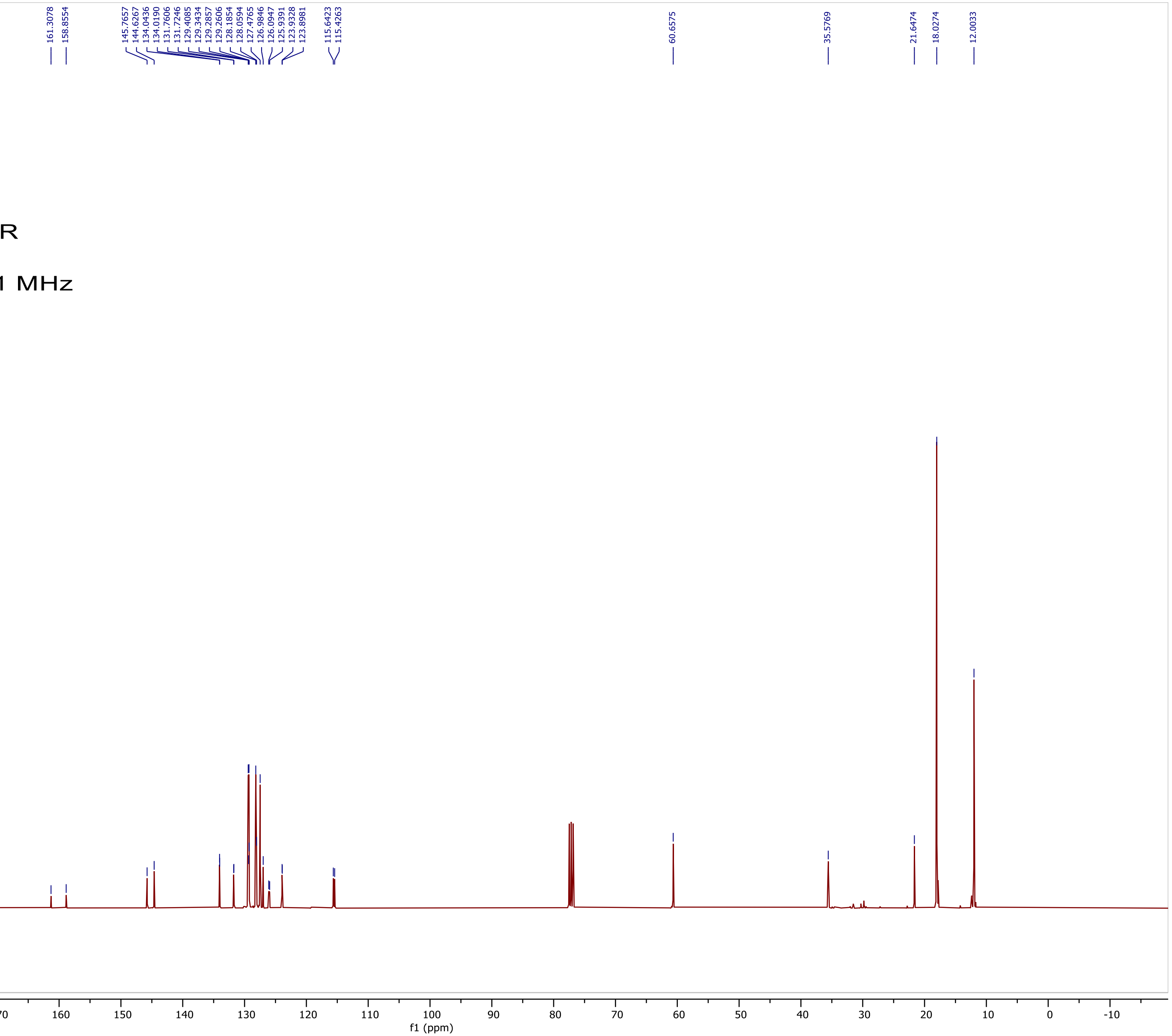

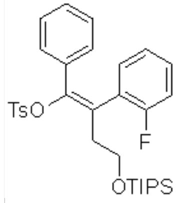

22l
Analysis: 19F NMR
Solvent: CDCl3
Field strength: 376 MHz

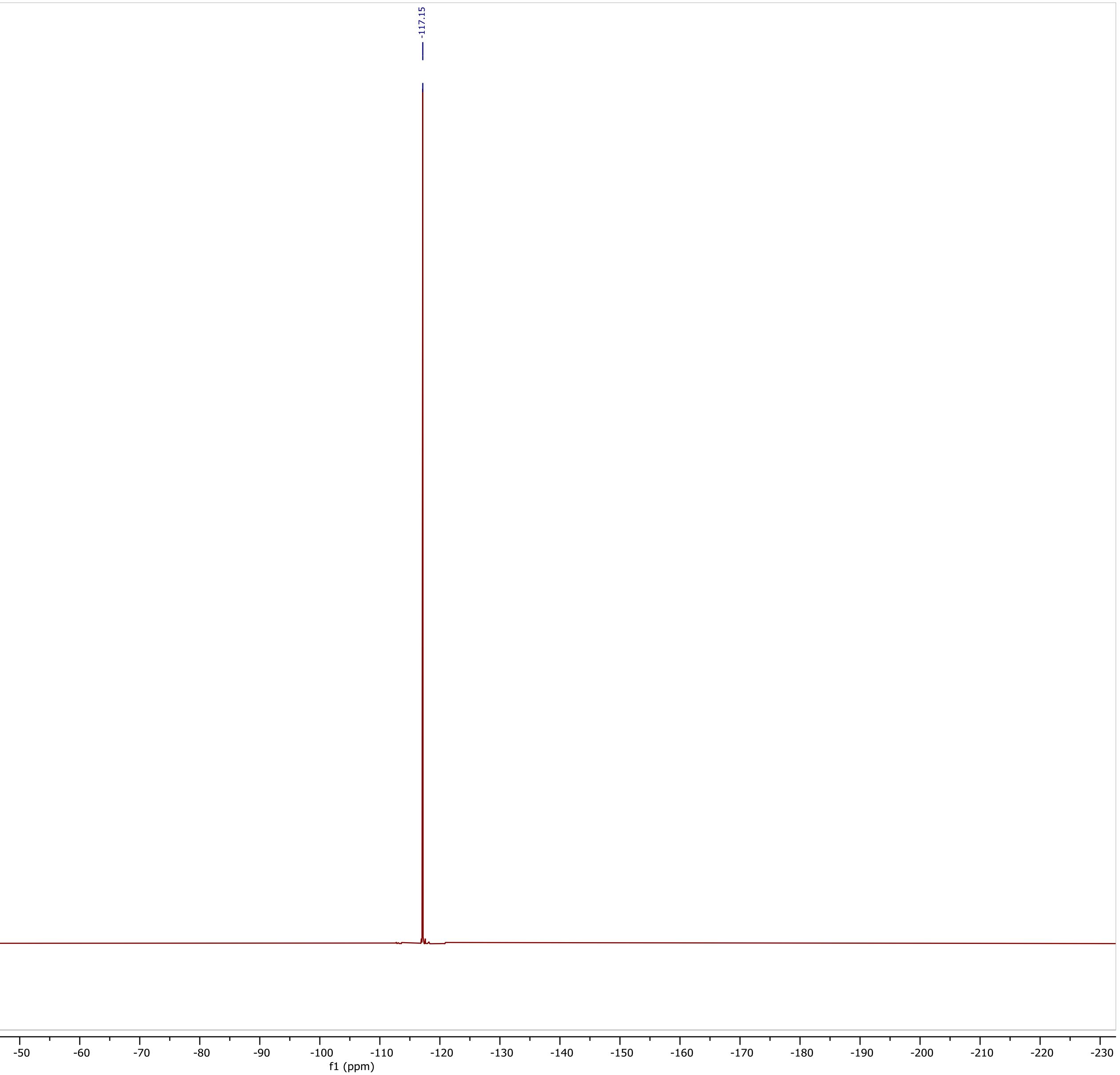

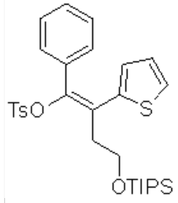

22m (mix of isomers 7:1)
Analysis: 1H NMR
Solvent: CDCl3
Field strength: 400 MHz

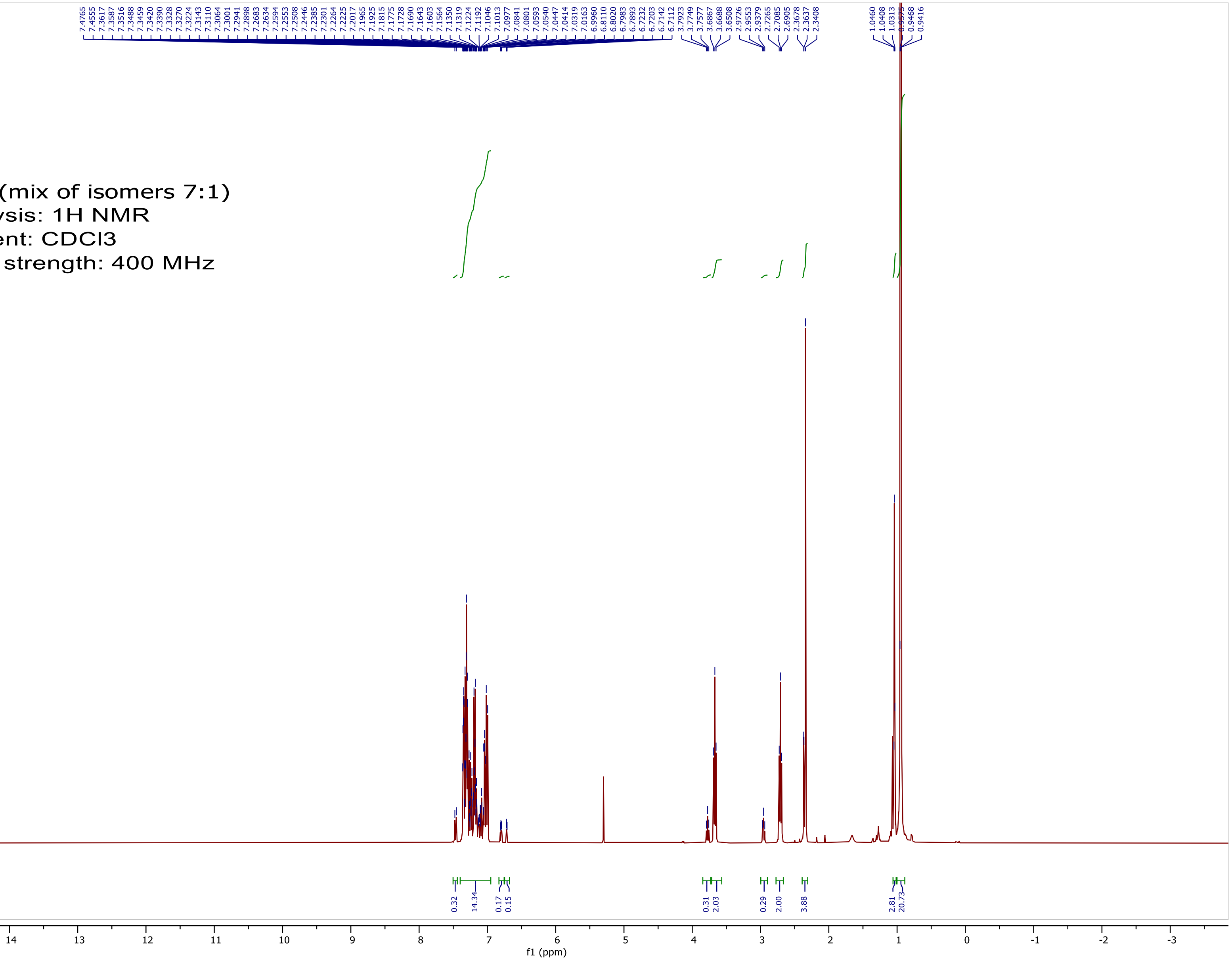

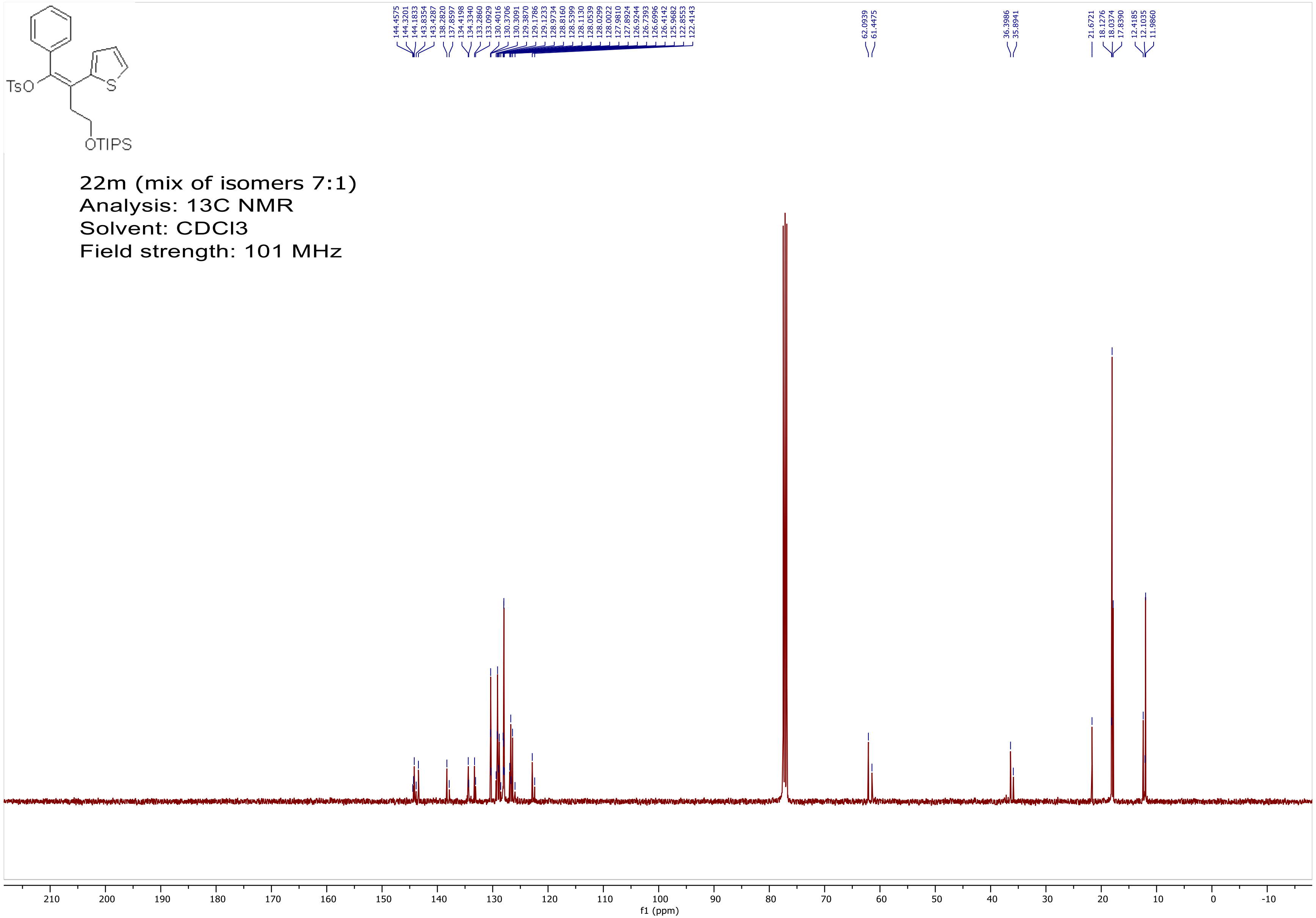
TsO
S
OTIPS
22m (mix of isomers 7:1)
Analysis: 13C NMR
Solvent: CDCl3
Field strength: 101 MHz
144.4575
144.3201
144.1833
143.8354
143.4287
138.2820
137.8597
134.4198
134.3340
133.2860
133.0929
130.4016
130.3706
130.3091
129.3870
129.1786
129.1233
128.9734
128.8160
128.5399
128.1130
128.0539
128.0299
128.0022
127.9810
127.8924
126.9244
126.7393
126.6996
126.4142
125.9682
122.8553
122.4143
62.0939
61.4475
36.3986
35.8941
21.6721
18.1276
18.0374
17.8390
12.4185
12.1035
11.9860
210
200
190
180
170
160
150
140
130
120
110
100
90
80
70
60
50
40
30
20
10
0
-10
f1 (ppm)

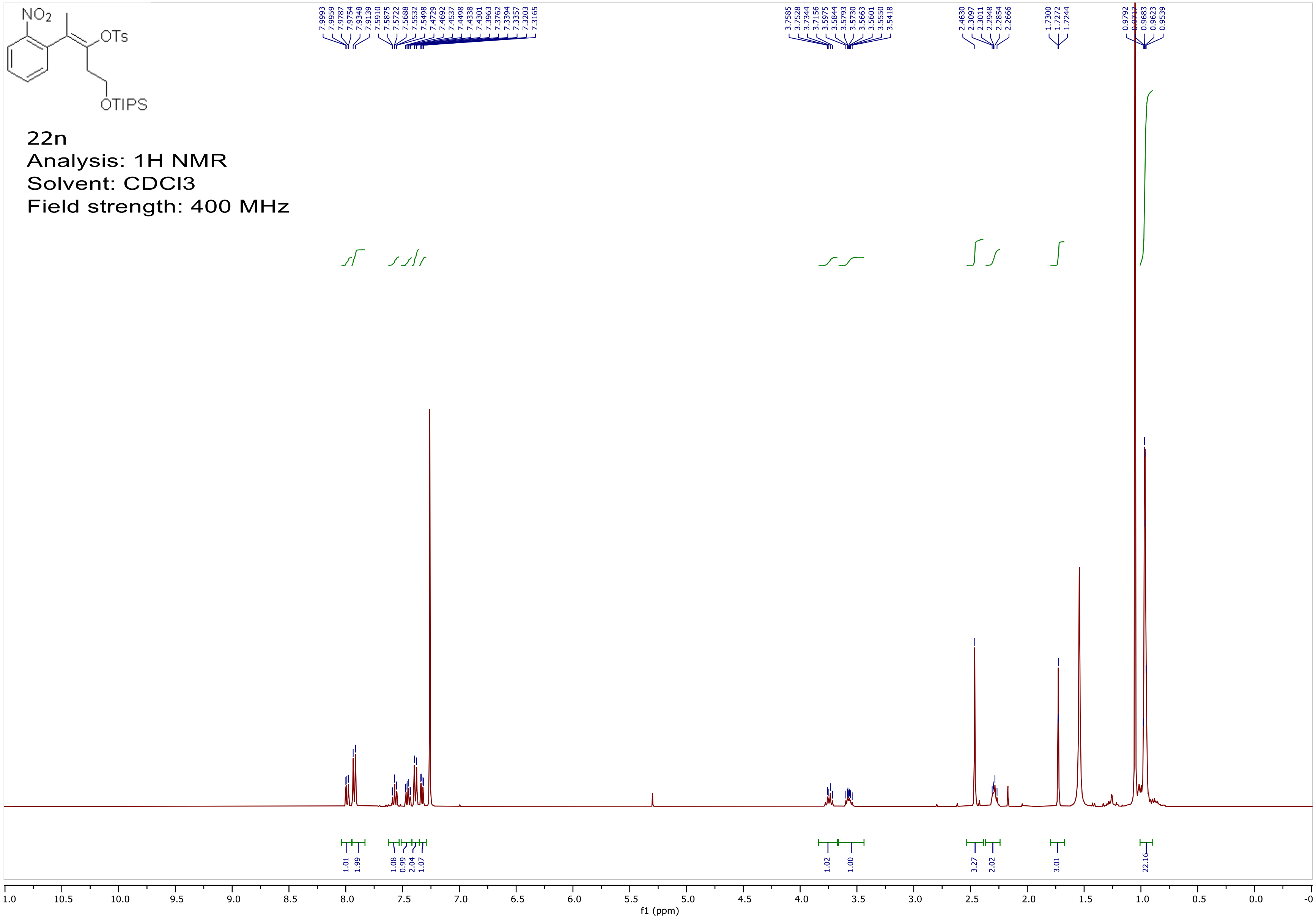
NO2
OTs
OTIPS
22n
Analysis: 1H NMR
Solvent: CDCl3
Field strength: 400 MHz
7.9993 7.9959 7.9787 7.9754 7.9348 7.9139 7.5910 7.5875 7.5722 7.5688 7.5532 7.5498 7.4729 7.4692 7.4537 7.4498 7.4338 7.4301 7.3963 7.3762 7.3394 7.3357 7.3203 7.3165
3.7585 3.7528 3.7344 3.7156 3.5975 3.5844 3.5793 3.5730 3.5663 3.5601 3.5550 3.5418
2.4630 2.3097 2.3011 2.2948 2.2854 2.2666
1.7300 1.7272 1.7244
0.9792 0.9717 0.9683 0.9623 0.9539
1.01 1.99 1.08 0.99 2.04 1.07 1.02 1.00 3.27 2.02 3.01 22.16
f1 (ppm)

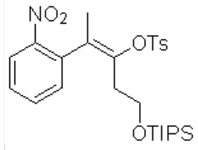

22n
Analysis: 13C NMR
Solvent: CDCl3
Field strength: 101 MHz

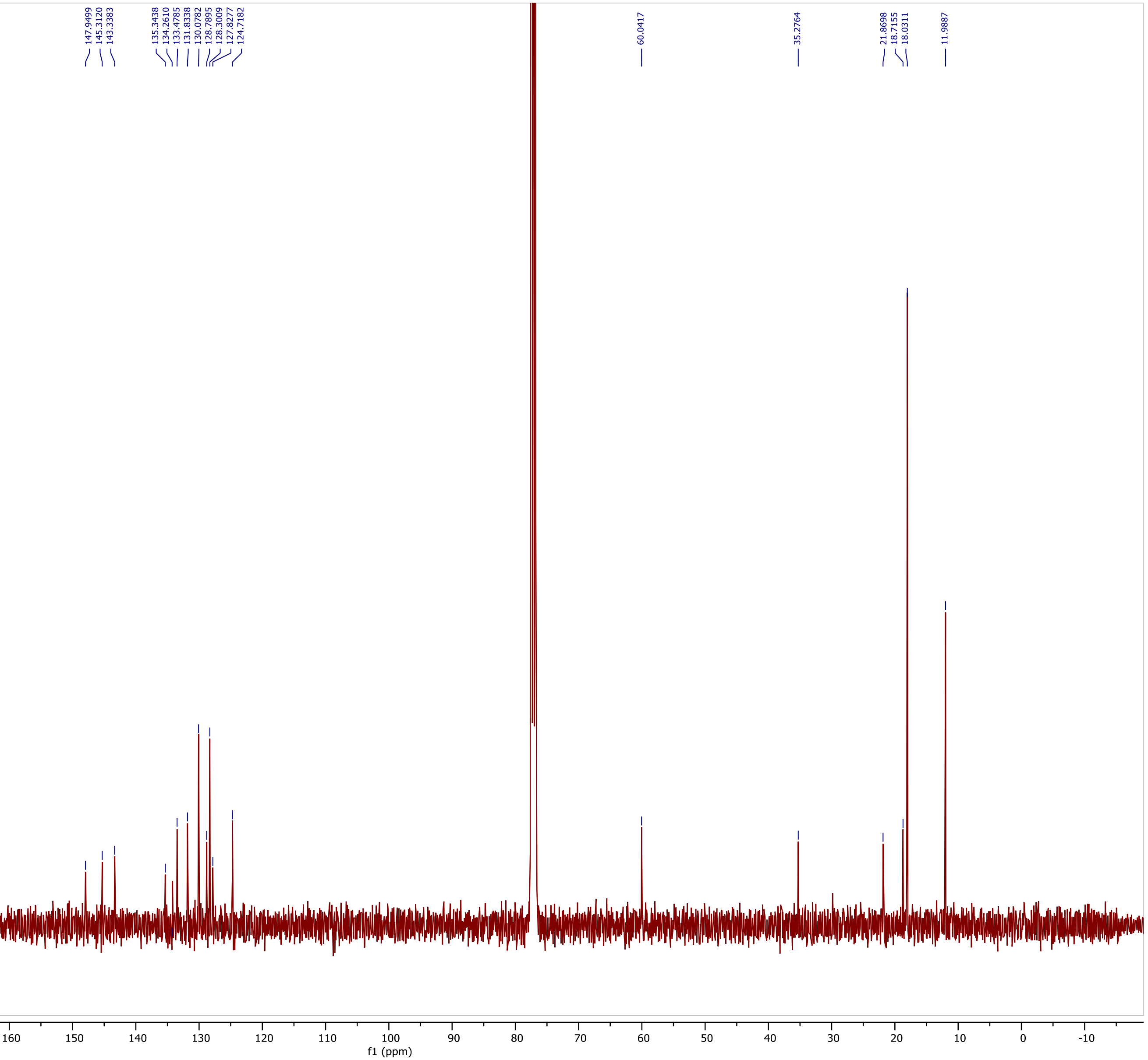

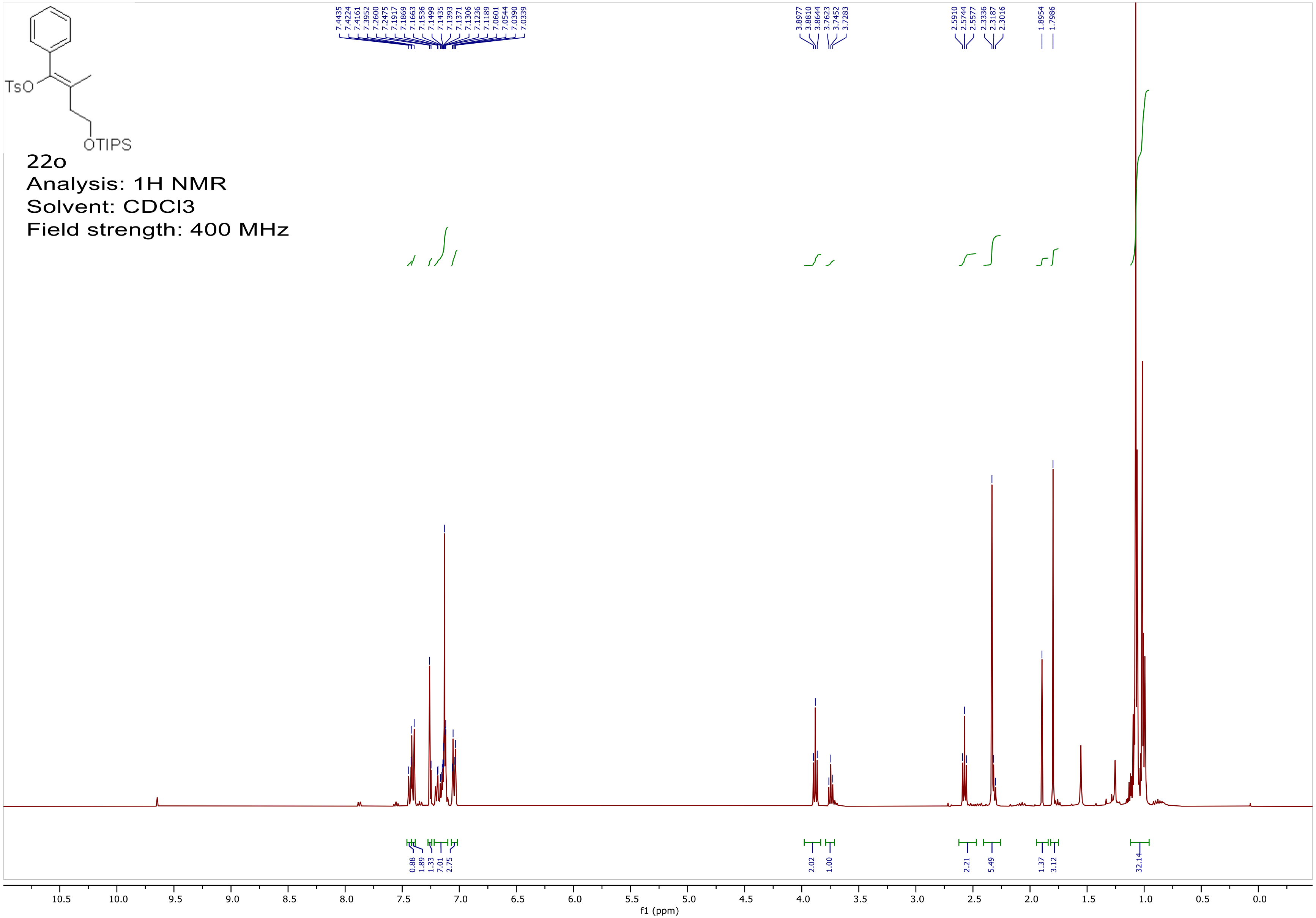
TsO
OTIPS
22o
Analysis: 1H NMR
Solvent: CDCl3
Field strength: 400 MHz
7.4435 7.4224 7.4161 7.3952 7.2600 7.2475 7.1917 7.1869 7.1663 7.1536 7.1499 7.1435 7.1393 7.1371 7.1306 7.1236 7.1189 7.0601 7.0544 7.0390 7.0339
3.8977 3.8810 3.8644 3.7623 3.7452 3.7283
2.5910 2.5744 2.5577 2.3336 2.3187 2.3016
1.8954 1.7986
0.88 1.89 1.33 7.01 2.75 2.02 1.00 2.21 5.49 1.37 3.12 32.14
f1 (ppm)

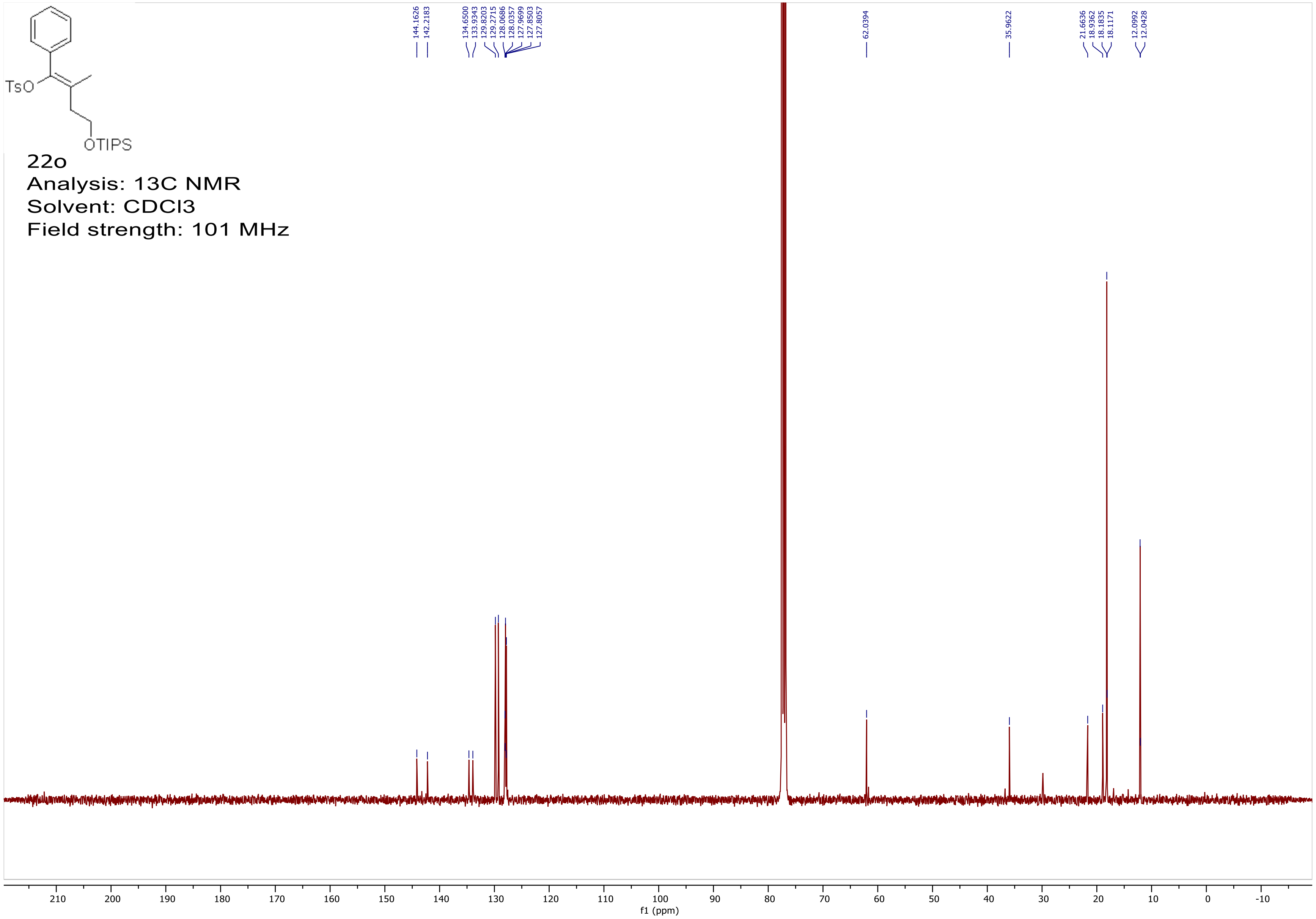
TsO
OTIPS
22o
Analysis: 13C NMR
Solvent: CDCl3
Field strength: 101 MHz
144.1626
142.2183
134.6500
133.9343
129.8203
129.2715
128.0686
128.0357
127.9699
127.8503
127.8057
62.0394
35.9622
21.6636
18.9362
18.1835
18.1171
12.0992
12.0428
210 200 190 180 170 160 150 140 130 120 110 100 90 80 70 60 50 40 30 20 10 0 -10
f1 (ppm)

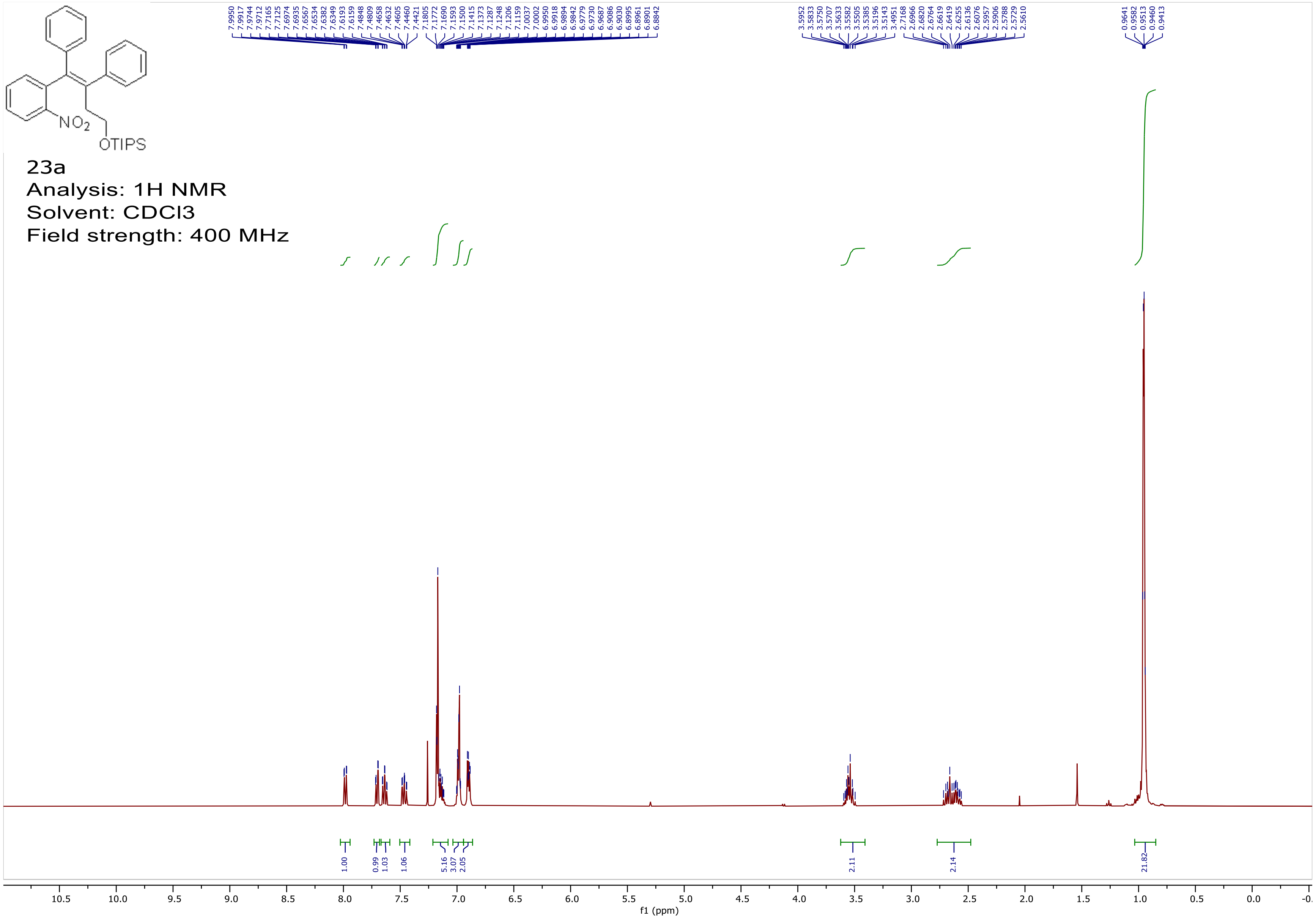

23a
Analysis: 1H NMR
Solvent: CDCl3
Field strength: 400 MHz
f1 (ppm)

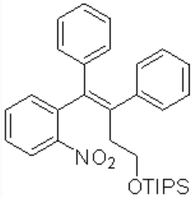

23a
Analysis: 13C NMR
Solvent: CDCl3
Field strength: 101 MHz

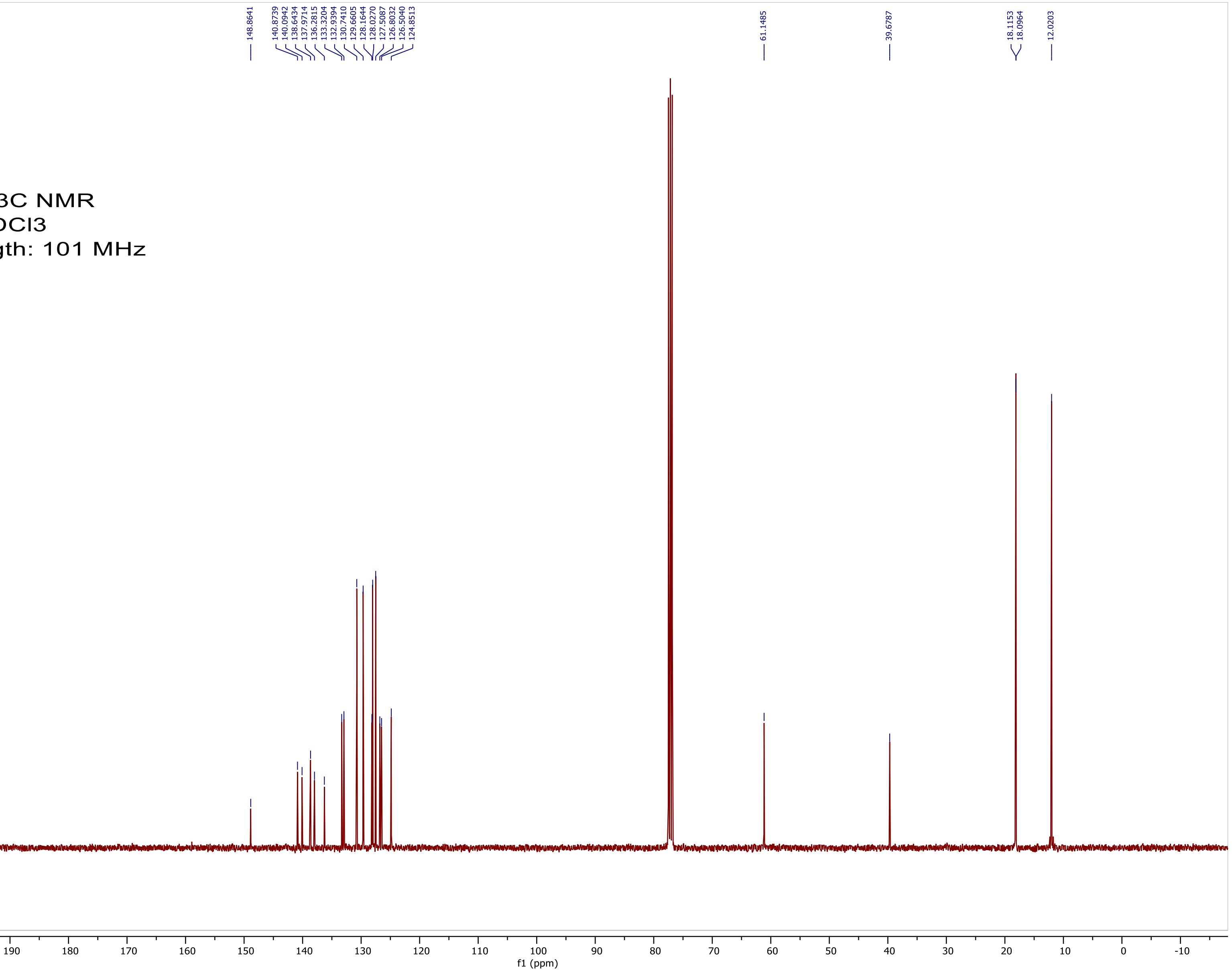

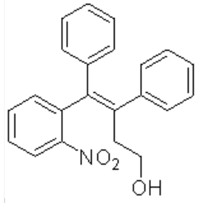

7a
Analysis: 1H NMR
Solvent: CDCl3
Field strength: 400 MHz

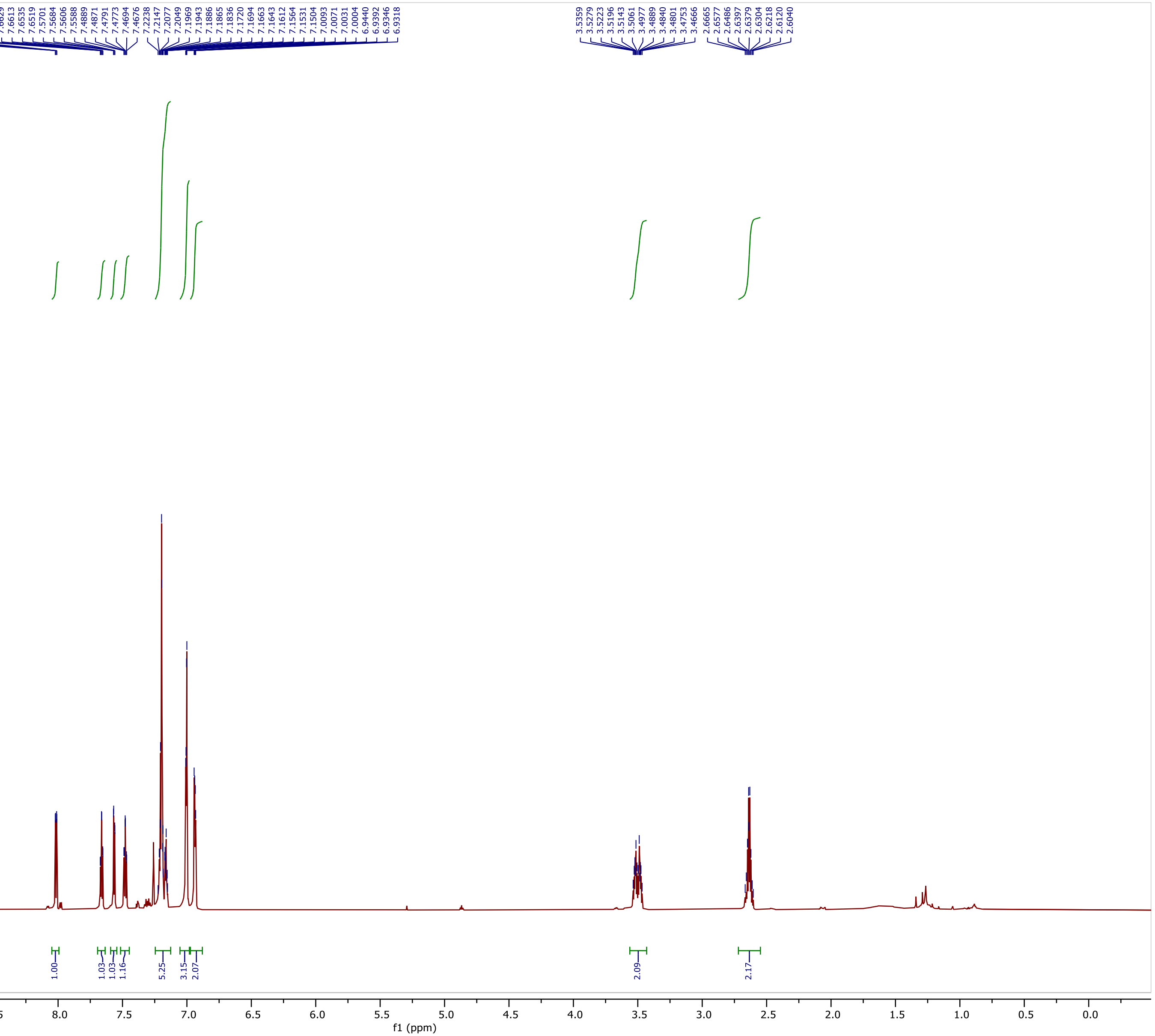

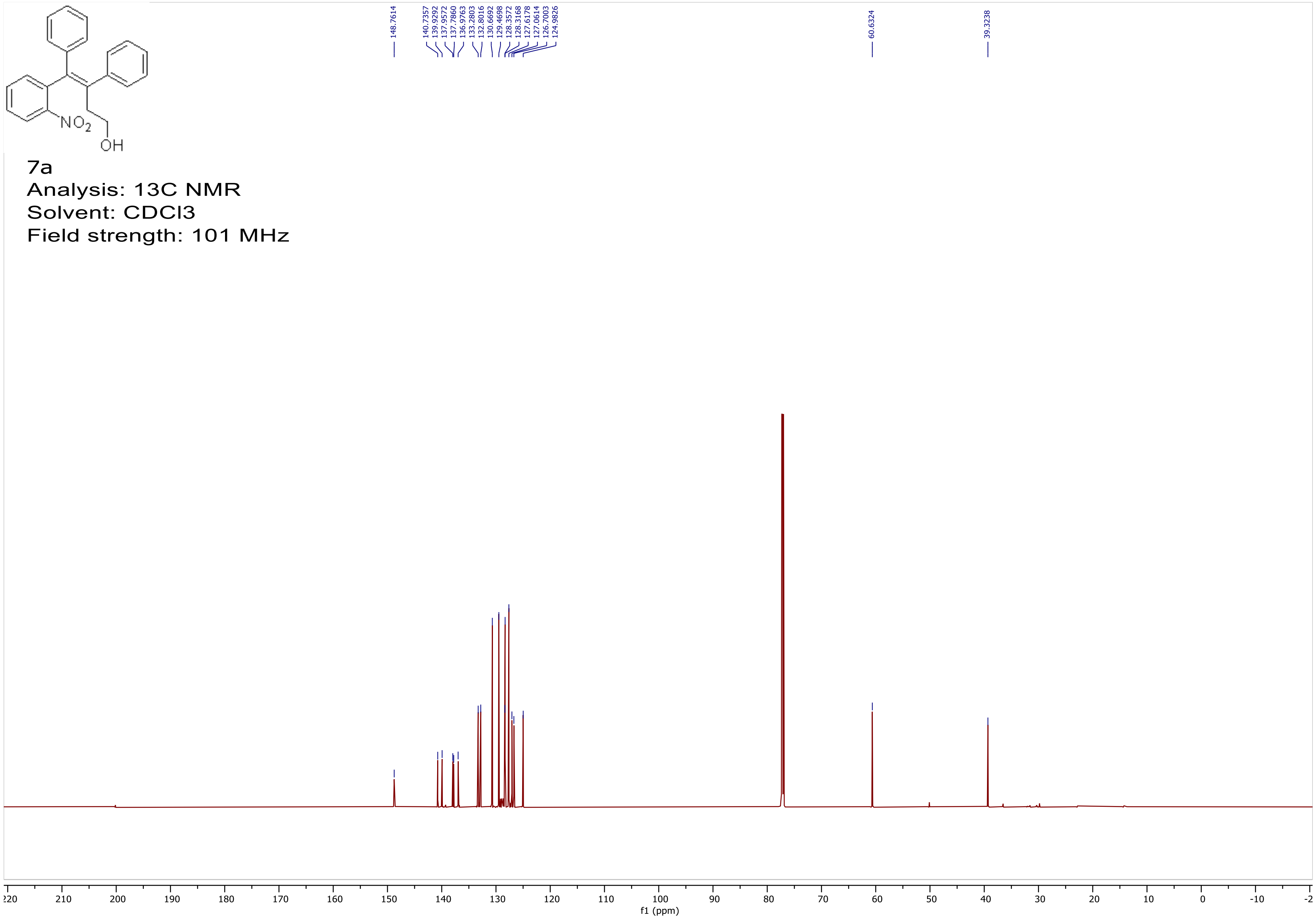
NO2
OH
7a
Analysis: 13C NMR
Solvent: CDCl3
Field strength: 101 MHz
148.7614
140.7357
139.9292
137.9572
137.7860
136.9763
133.2803
132.8016
130.6692
129.4698
128.3572
128.3168
127.6178
127.0614
126.7003
124.9826
60.6324
39.3238
f1 (ppm)

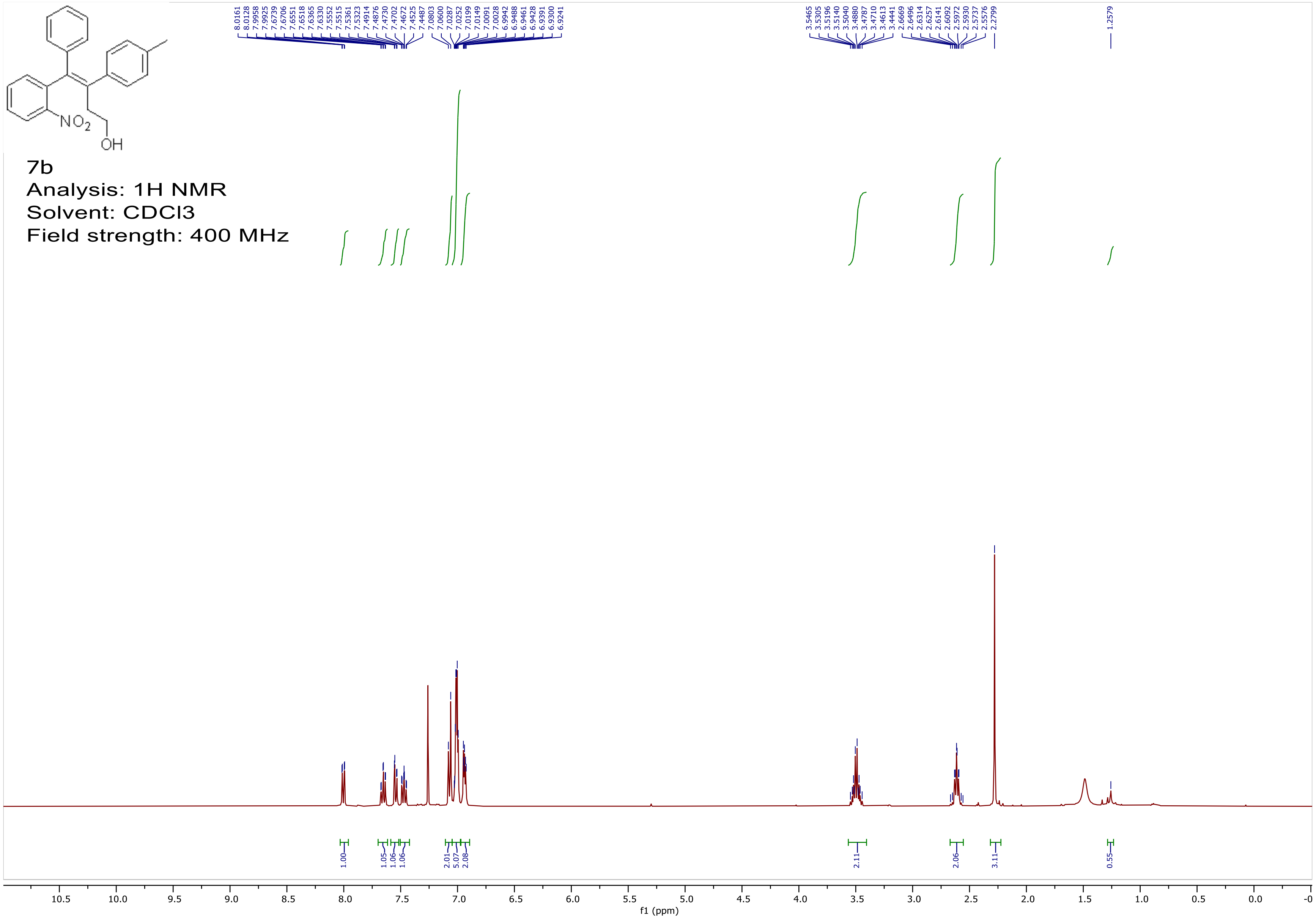
7b
Analysis: 1H NMR
Solvent: CDCl3
Field strength: 400 MHz
f1 (ppm)

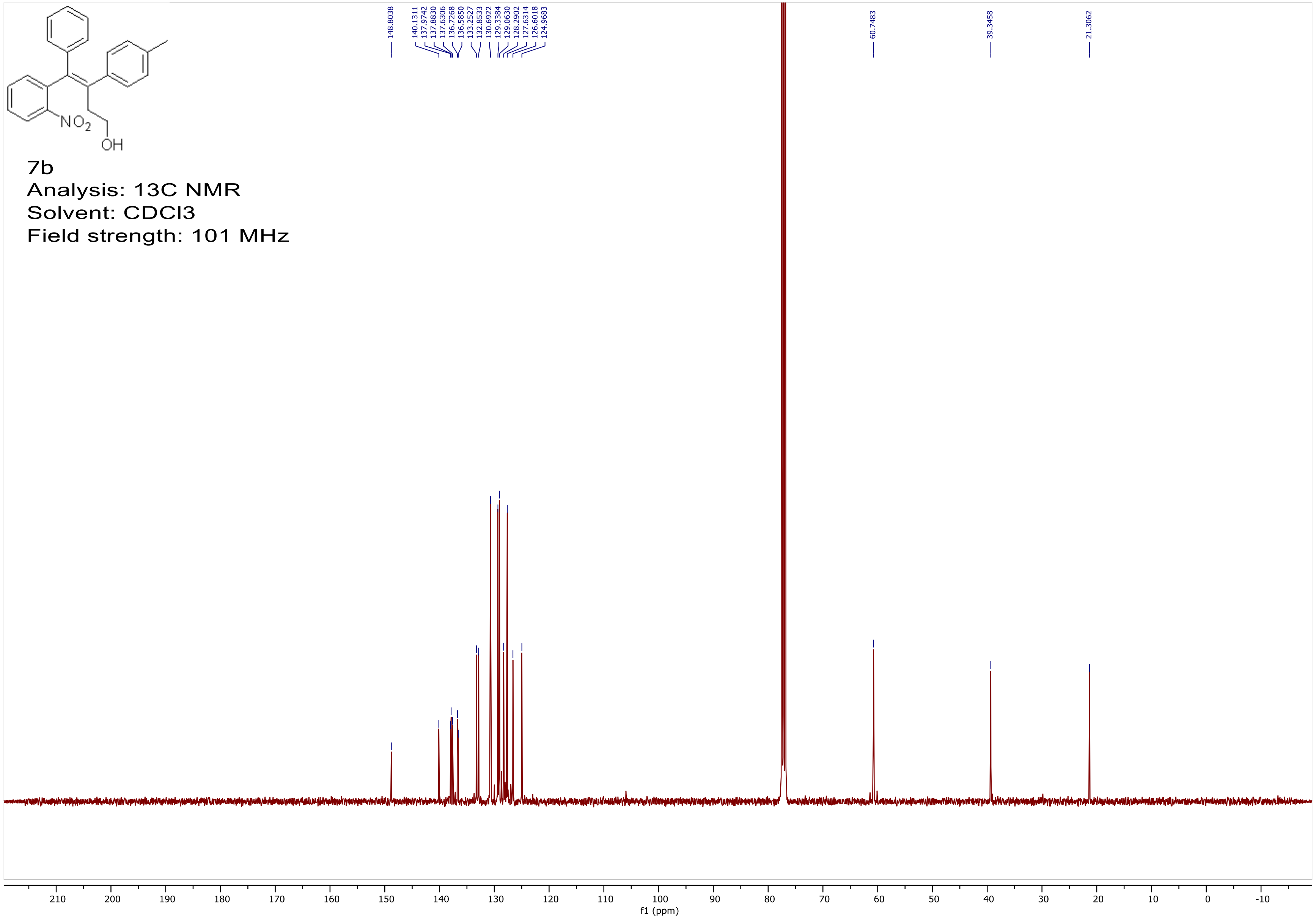
NO2
OH
7b
Analysis: 13C NMR
Solvent: CDCl3
Field strength: 101 MHz
148.8038
140.1311
137.9742
137.8830
137.6306
136.7268
136.5850
133.2527
132.8533
130.6922
129.3384
129.0630
128.2902
127.6314
126.6018
124.9683
60.7483
39.3458
21.3062
210
200
190
180
170
160
150
140
130
120
110
100
90
80
70
60
50
40
30
20
10
0
-10
f1 (ppm)

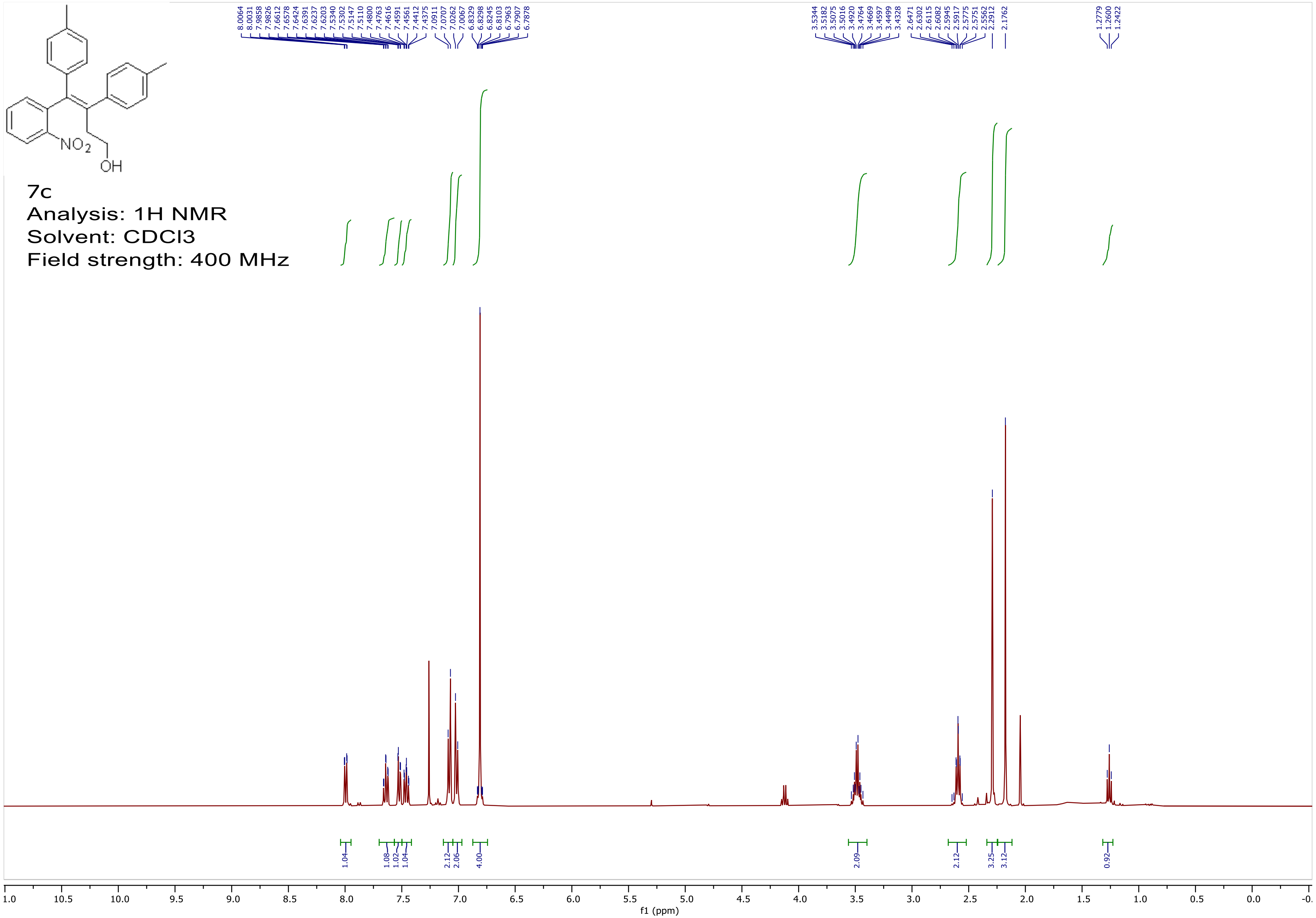
NO2
OH
7c
Analysis: 1H NMR
Solvent: CDCl3
Field strength: 400 MHz
8.0064 8.0031 7.9858 7.9826 7.6612 7.6578 7.6424 7.6391 7.6237 7.6203 7.5340 7.5302 7.5147 7.5110 7.4800 7.4763 7.4616 7.4591 7.4561 7.4412 7.4375 7.0911 7.0707 7.0262 7.0067 6.8329 6.8298 6.8245 6.8103 6.7963 6.7907 6.7878
3.5344 3.5182 3.5075 3.5016 3.4920 3.4764 3.4669 3.4597 3.4499 3.4328 2.6471 2.6302 2.6115 2.6082 2.5945 2.5917 2.5775 2.5751 2.5562 2.2912 2.1762
1.2779 1.2600 1.2422
1.04 1.08 1.02 1.04 2.12 2.06 4.00 2.09 2.12 3.25 3.12 0.92
f1 (ppm)

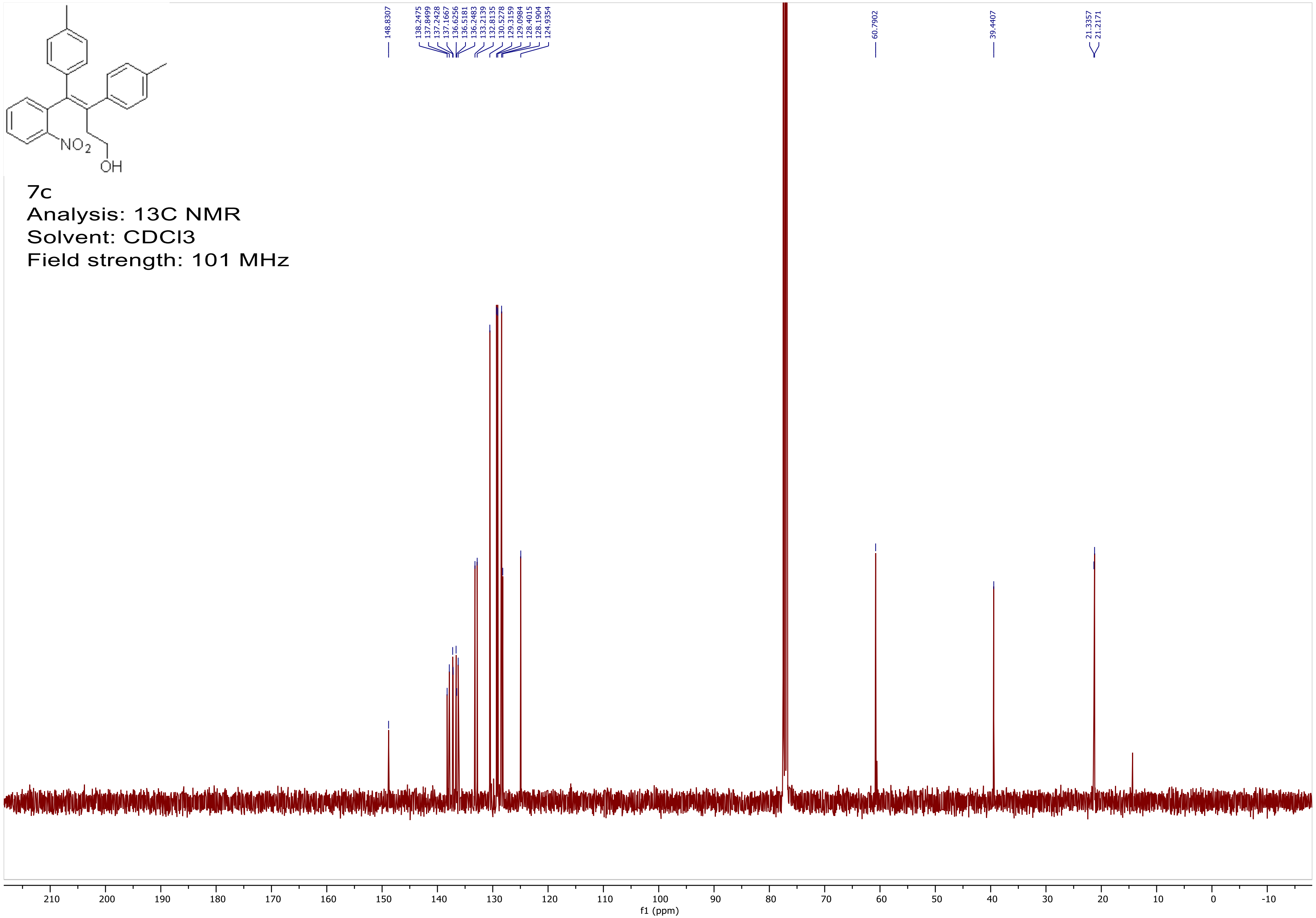
NO2
OH
7c
Analysis: 13C NMR
Solvent: CDCl3
Field strength: 101 MHz
148.8307
138.2475
137.8499
137.2428
137.1667
136.6256
136.5181
136.2483
133.2139
132.8135
130.5278
129.3159
129.0984
128.4015
128.1904
124.9354
60.7902
39.4407
21.3357
21.2171
210
200
190
180
170
160
150
140
130
120
110
100
90
80
70
60
50
40
30
20
10
0
-10
f1 (ppm)

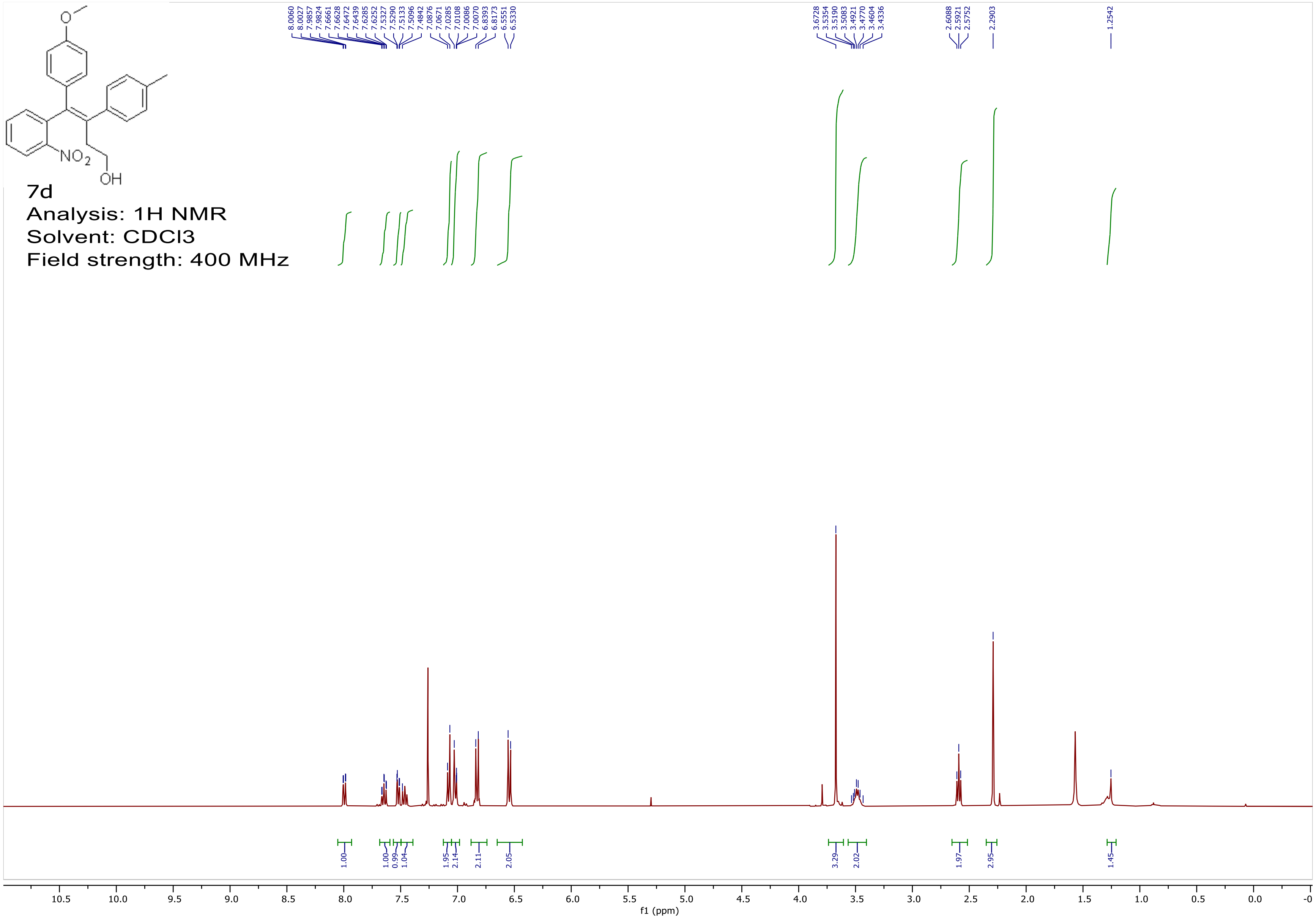
NO2
OH
7d
Analysis: 1H NMR
Solvent: CDCl3
Field strength: 400 MHz
8.0060
8.0027
7.9857
7.9824
7.6661
7.6628
7.6472
7.6439
7.6285
7.6252
7.5327
7.5290
7.5133
7.5096
7.4842
7.0876
7.0671
7.0285
7.0108
7.0086
7.0070
6.8393
6.8173
6.5551
6.5330
3.6728
3.5354
3.5190
3.5083
3.4921
3.4770
3.4604
3.4336
2.6088
2.5921
2.5752
2.2903
1.2542
1.00
1.00
0.99
1.04
1.95
2.14
2.11
2.05
3.29
2.02
1.97
2.95
1.45
10.5
10.0
9.5
9.0
8.5
8.0
7.5
7.0
6.5
6.0
5.5
5.0
4.5
4.0
3.5
3.0
2.5
2.0
1.5
1.0
0.5
0.0
-0
f1 (ppm)

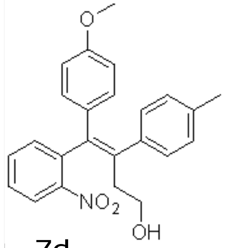

7d
Analysis: 13C NMR
Solvent: CDCl3
Field strength: 101 MHz

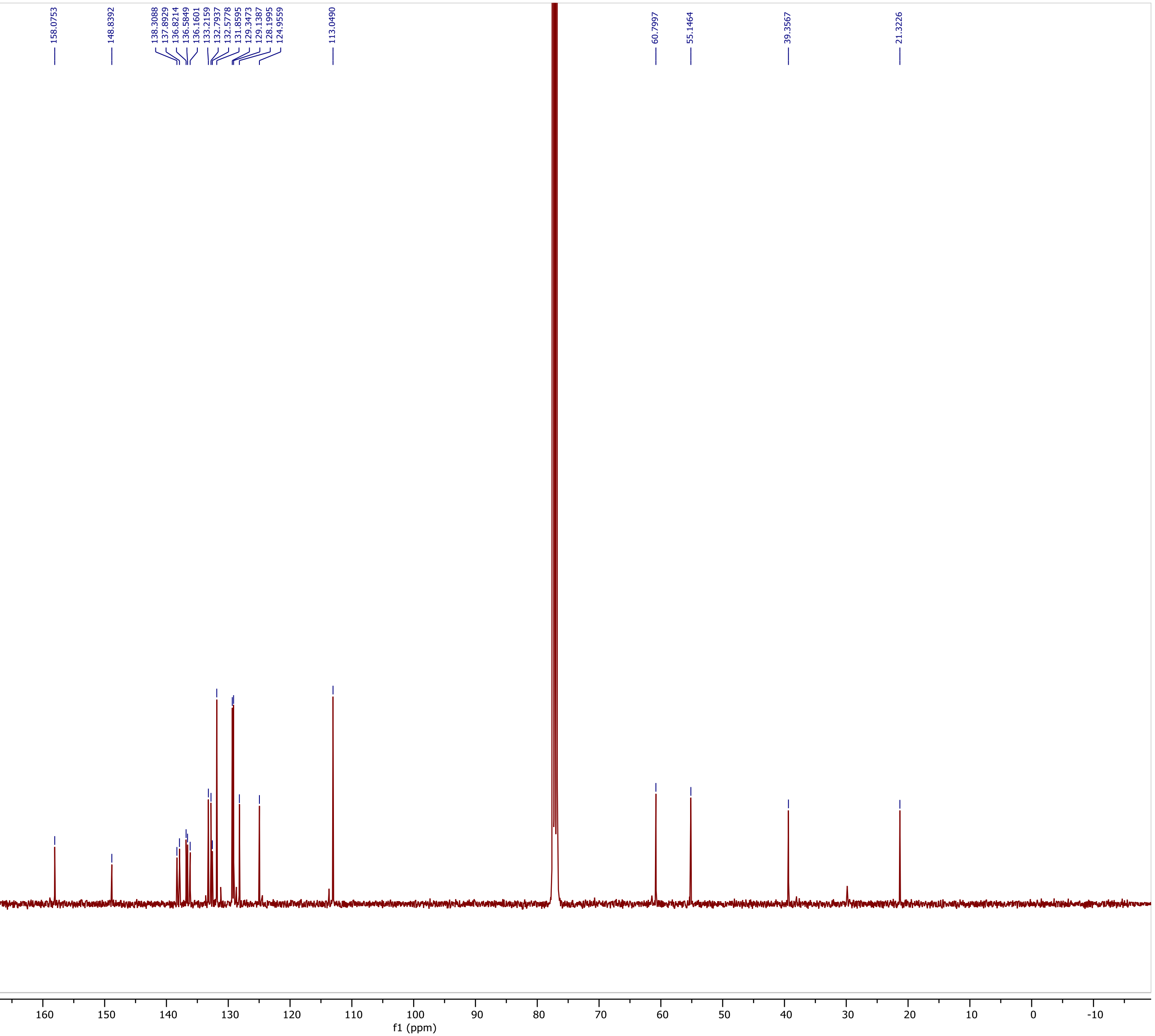

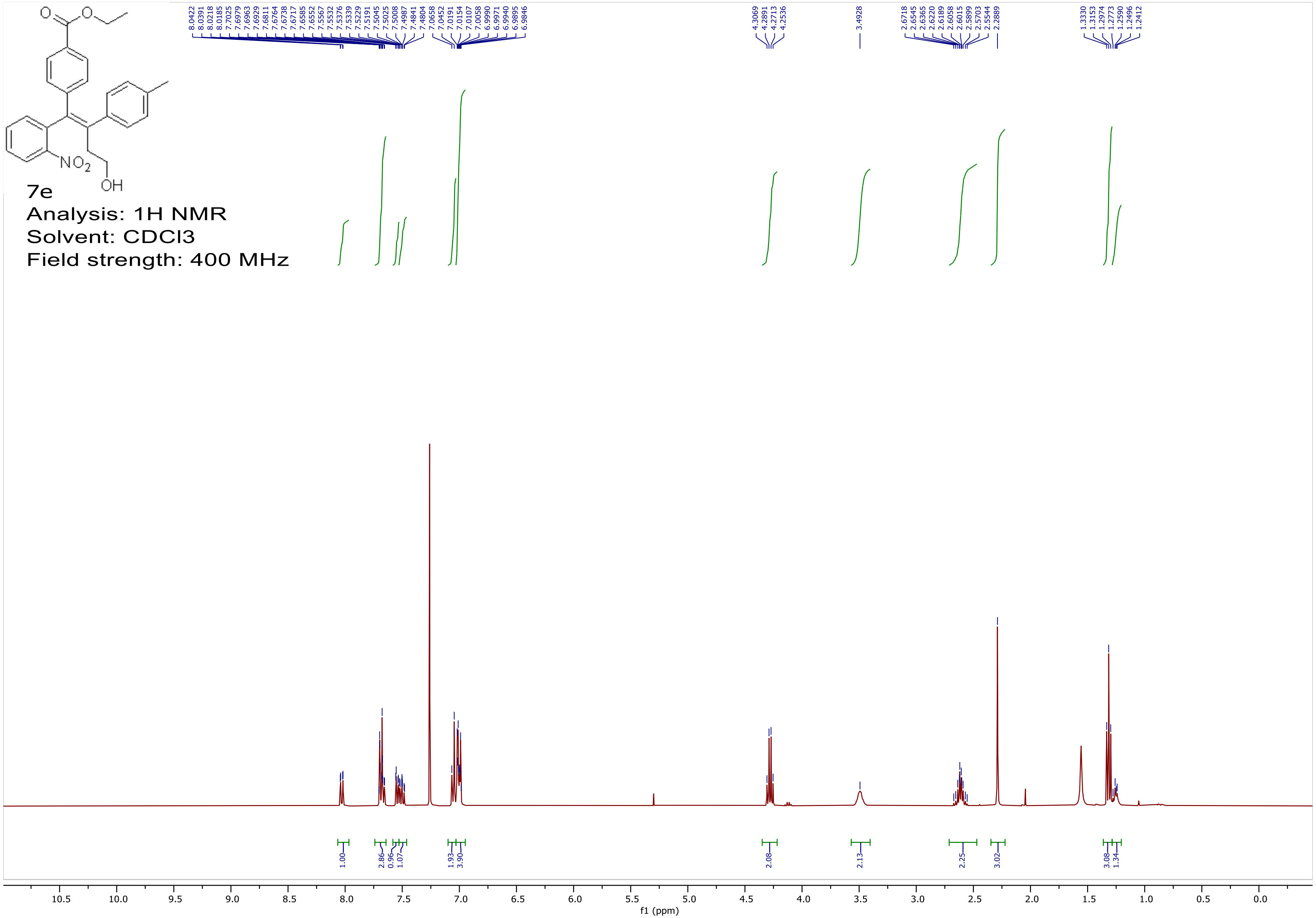
7e
Analysis: 1H NMR
Solvent: CDCl3
Field strength: 400 MHz
8.0422 8.0391 8.0218 8.0185 7.7025 7.6979 7.6963 7.6929 7.6811 7.6764 7.6738 7.6717 7.6585 7.6552 7.5567 7.5532 7.5376 7.5339 7.5229 7.5191 7.5045 7.5025 7.5008 7.4987 7.4841 7.4804 7.0658 7.0452 7.0191 7.0154 7.0107 7.0058 6.9990 6.9971 6.9940 6.9895 6.9846
4.3069 4.2891 4.2713 4.2536
3.4928
2.6718 2.6545 2.6365 2.6220 2.6189 2.6058 2.6015 2.5899 2.5703 2.5544 2.2889
1.3330 1.3153 1.2974 1.2773 1.2590 1.2496 1.2412
1.00 2.86 0.96 1.07 1.93 3.90 2.08 2.13 2.25 3.02 3.08 1.34
f1 (ppm)

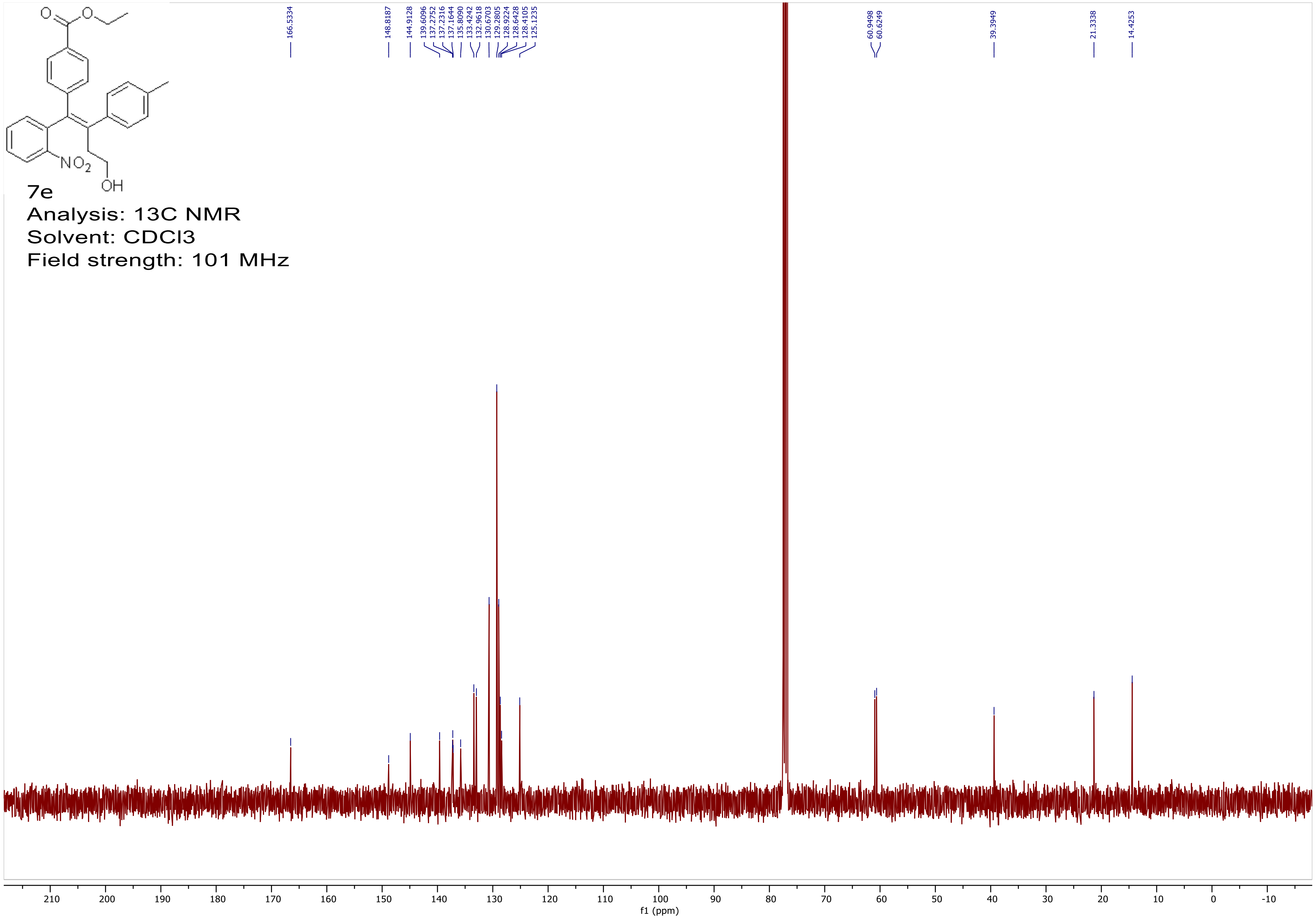
NO2
OH
7e
Analysis: 13C NMR
Solvent: CDCl3
Field strength: 101 MHz
166.5334
148.8187
144.9128
139.6096
137.2752
137.2316
137.1644
135.8090
133.4242
132.9618
130.6703
129.2805
128.9224
128.6428
128.4105
125.1235
60.9498
60.6249
39.3949
21.3338
14.4253
210
200
190
180
170
160
150
140
130
120
110
100
90
80
70
60
50
40
30
20
10
0
-10
f1 (ppm)

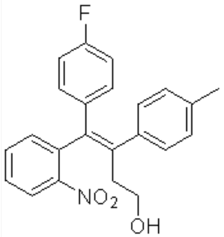

7f
Analysis: 1H NMR
Solvent: CDCl3
Field strength: 400 MHz

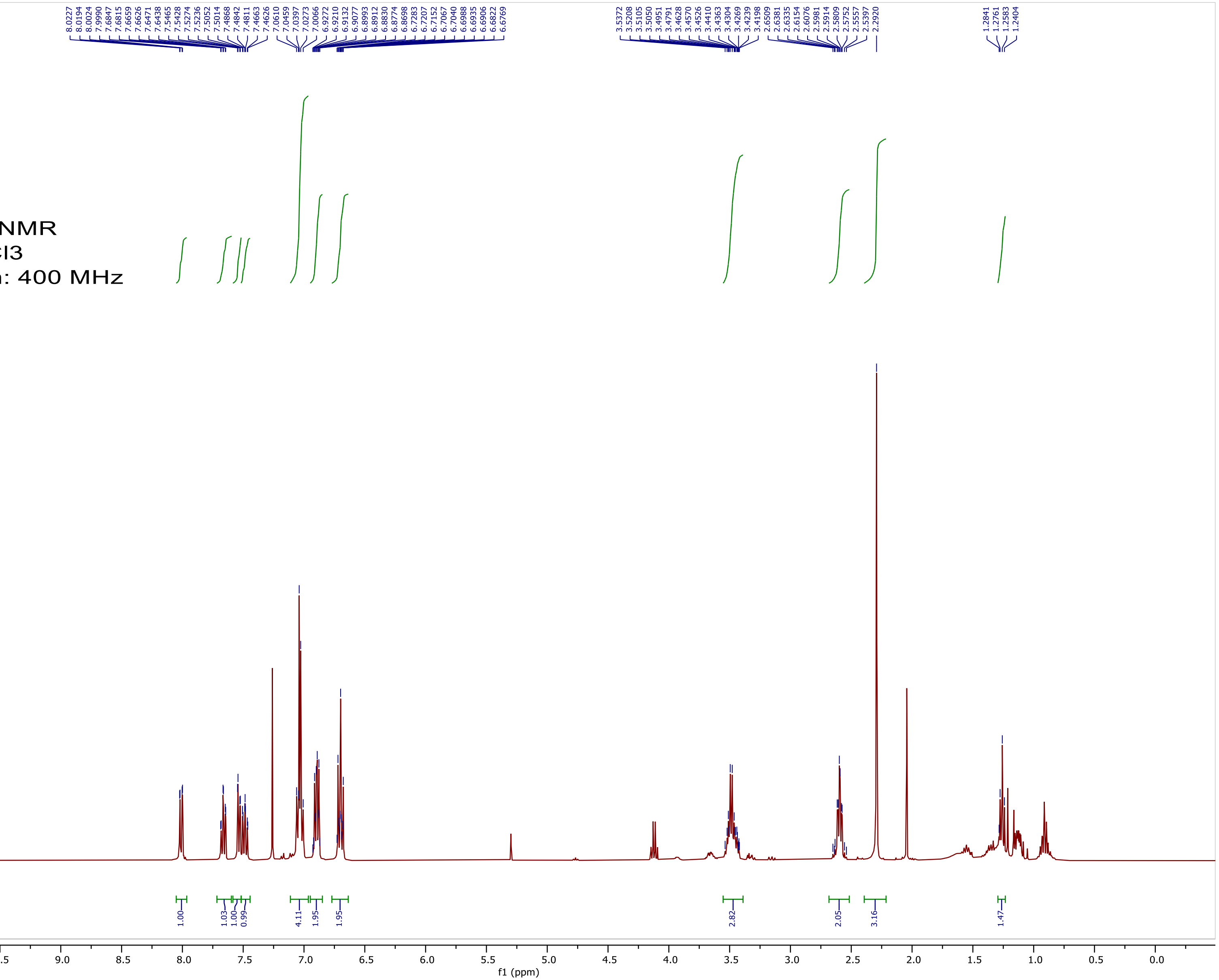

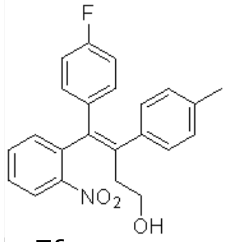

7f
Analysis: 13C NMR
Solvent: CDCl3
Field strength: 101 MHz

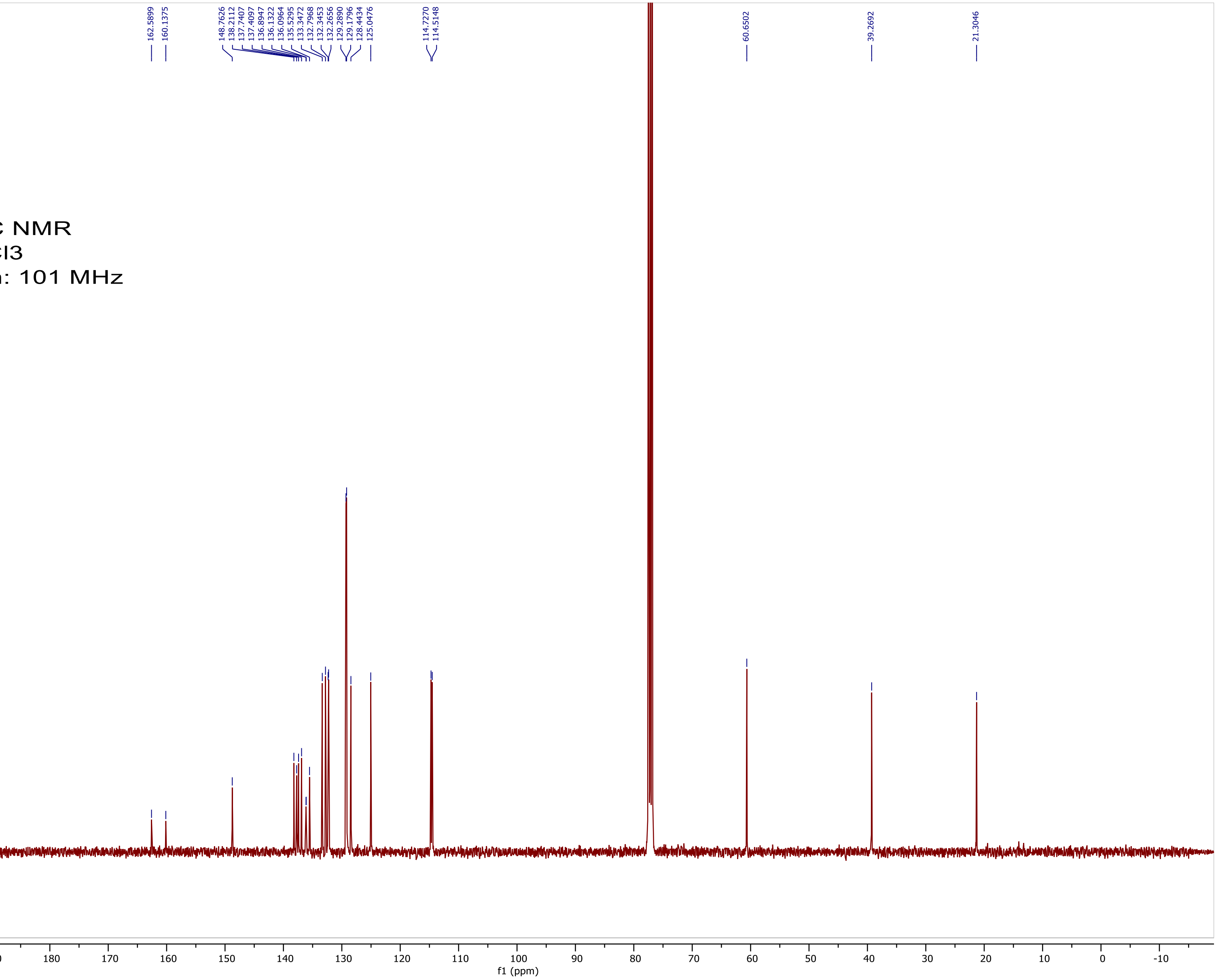

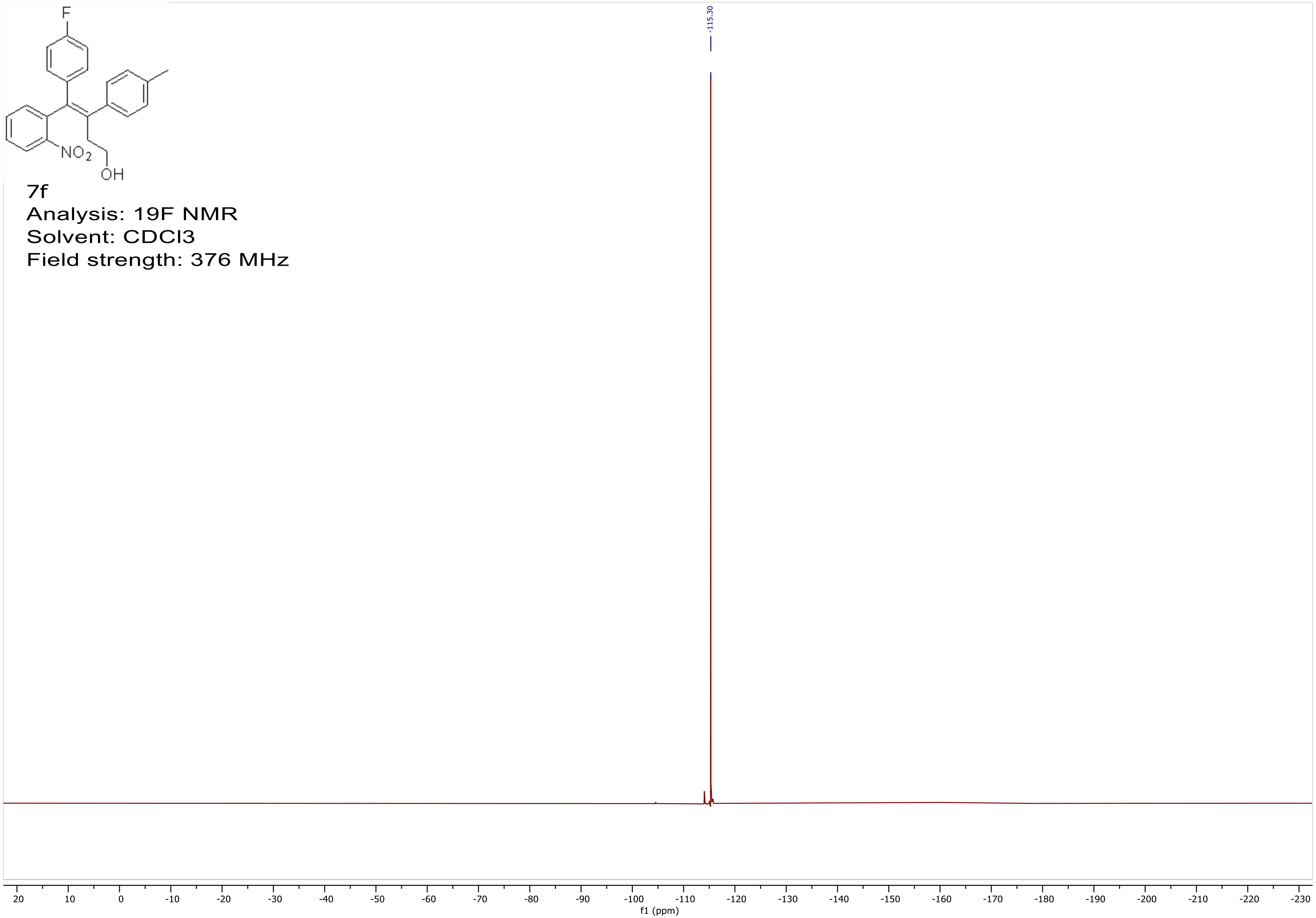

F
NO_2
OH
-115.30
7f
Analysis: 19F NMR
Solvent: CDCl3
Field strength: 376 MHz
20 10 0 -10 -20 -30 -40 -50 -60 -70 -80 -90 -100 -110 -120 -130 -140 -150 -160 -170 -180 -190 -200 -210 -220 -230
f1 (ppm)

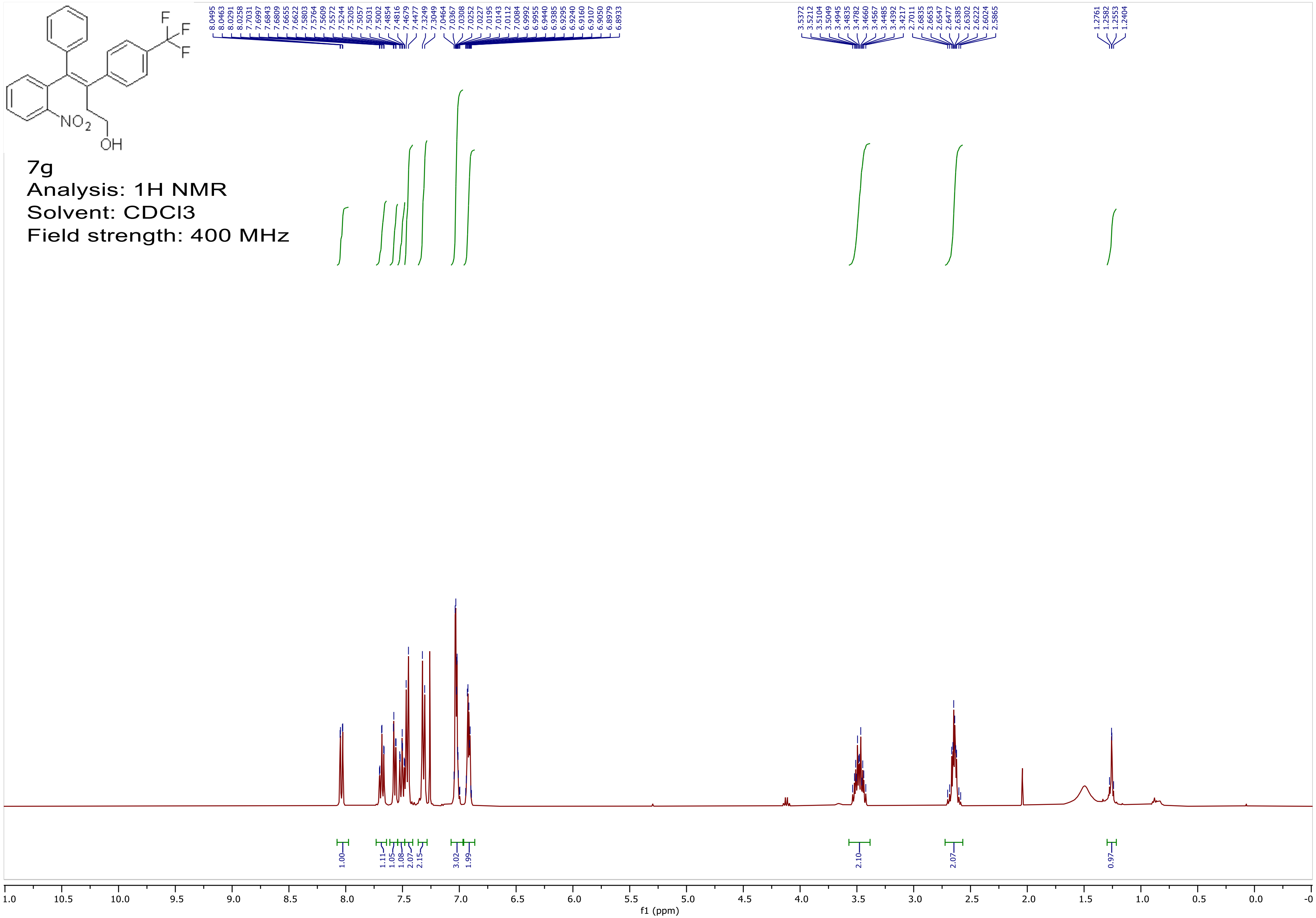

7g
Analysis: 1H NMR
Solvent: CDCl3
Field strength: 400 MHz
NO2
OH
F
F
F
8.0495 8.0463 8.0291 8.0258 7.7031 7.6997 7.6843 7.6809 7.6655 7.6622 7.5803 7.5764 7.5609 7.5572 7.5244 7.5205 7.5057 7.5031 7.5002 7.4854 7.4816 7.4679 7.4477 7.3249 7.3049 7.0464 7.0367 7.0308 7.0252 7.0227 7.0195 7.0143 7.0112 7.0084 6.9992 6.9955 6.9440 6.9385 6.9295 6.9240 6.9160 6.9107 6.9050 6.8979 6.8933
3.5372 3.5212 3.5104 3.5049 3.4945 3.4835 3.4782 3.4660 3.4567 3.4485 3.4392 3.4217 2.7011 2.6835 2.6653 2.6547 2.6477 2.6385 2.6302 2.6222 2.6024 2.5865
1.2761 1.2582 1.2553 1.2404
1.00 1.11 1.05 1.08 2.07 2.15 3.02 1.99 2.10 2.07 0.97
1.0 10.5 10.0 9.5 9.0 8.5 8.0 7.5 7.0 6.5 6.0 5.5 5.0 4.5 4.0 3.5 3.0 2.5 2.0 1.5 1.0 0.5 0.0 -0
f1 (ppm)

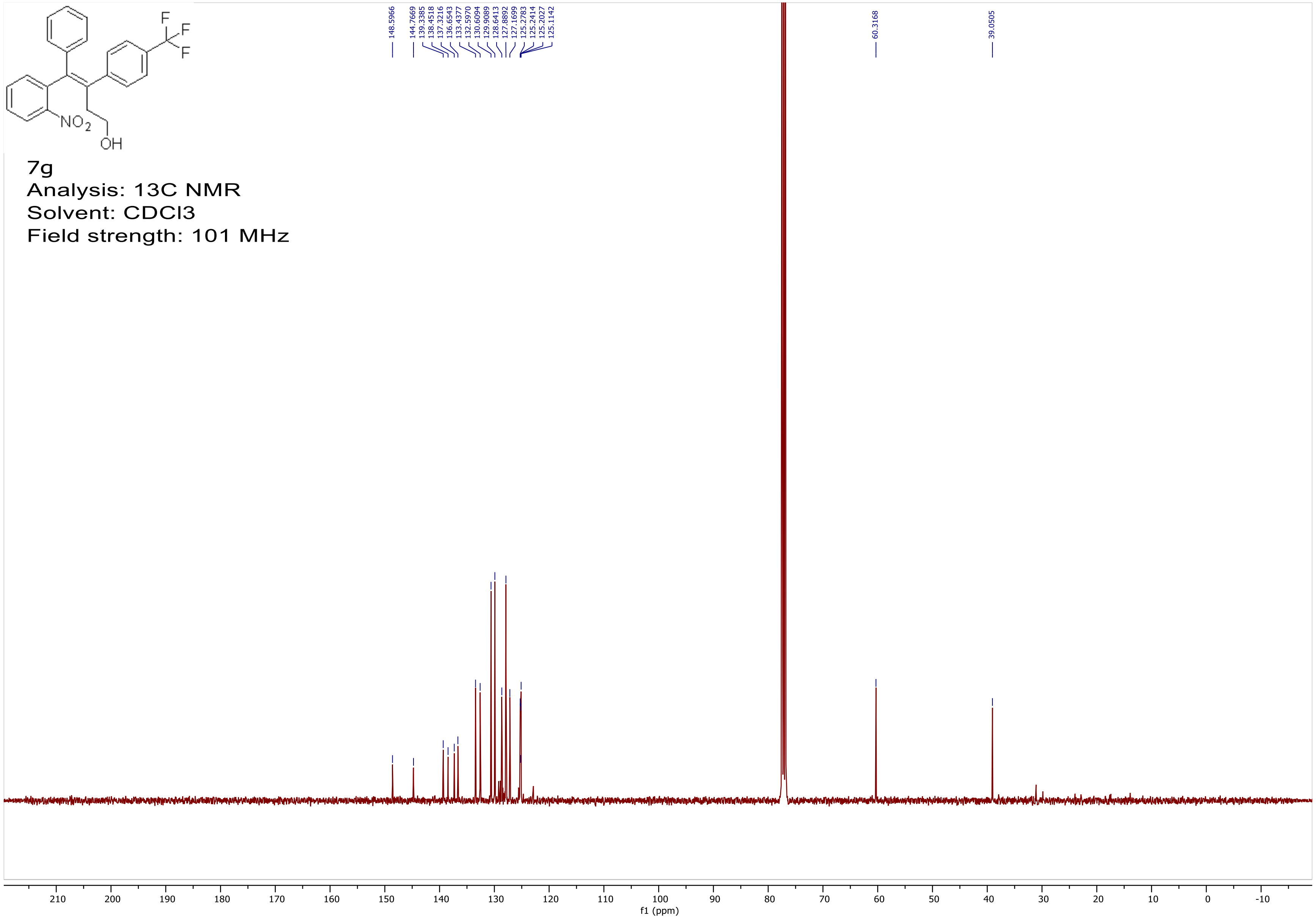
F
F
F
NO2
OH
7g
Analysis: 13C NMR
Solvent: CDCl3
Field strength: 101 MHz
148.5966
144.7669
139.3385
138.4518
137.3216
136.6543
133.4377
132.5970
130.6094
129.9089
128.6413
127.8892
127.1699
125.2783
125.2414
125.2027
125.1142
60.3168
39.0505
210
200
190
180
170
160
150
140
130
120
110
100
90
80
70
60
50
40
30
20
10
0
-10
f1 (ppm)

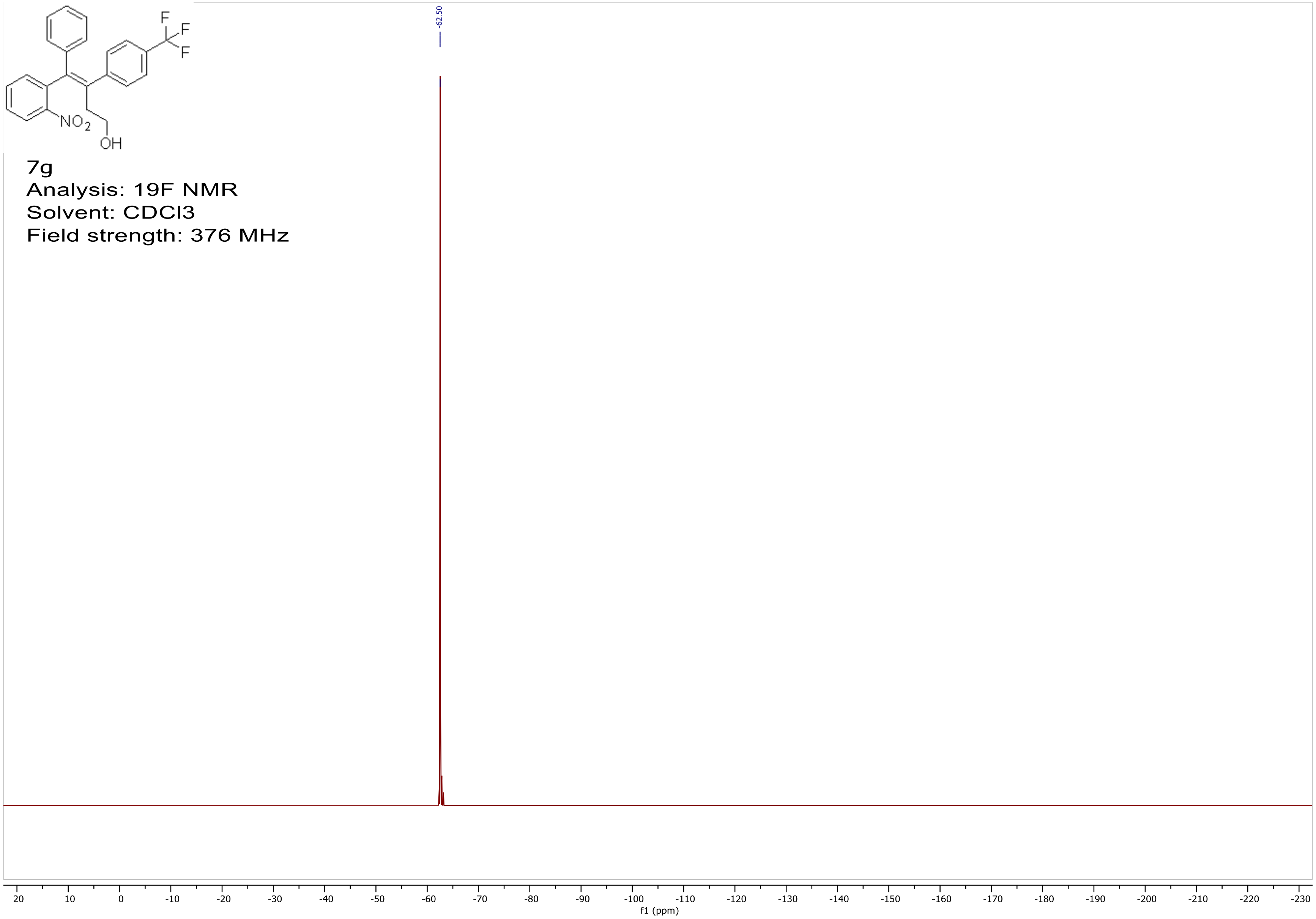
F
F
F
NO_2
OH
7g
Analysis: 19F NMR
Solvent: CDCl3
Field strength: 376 MHz
-62.50
20 10 0 -10 -20 -30 -40 -50 -60 -70 -80 -90 -100 -110 -120 -130 -140 -150 -160 -170 -180 -190 -200 -210 -220 -230
f1 (ppm)

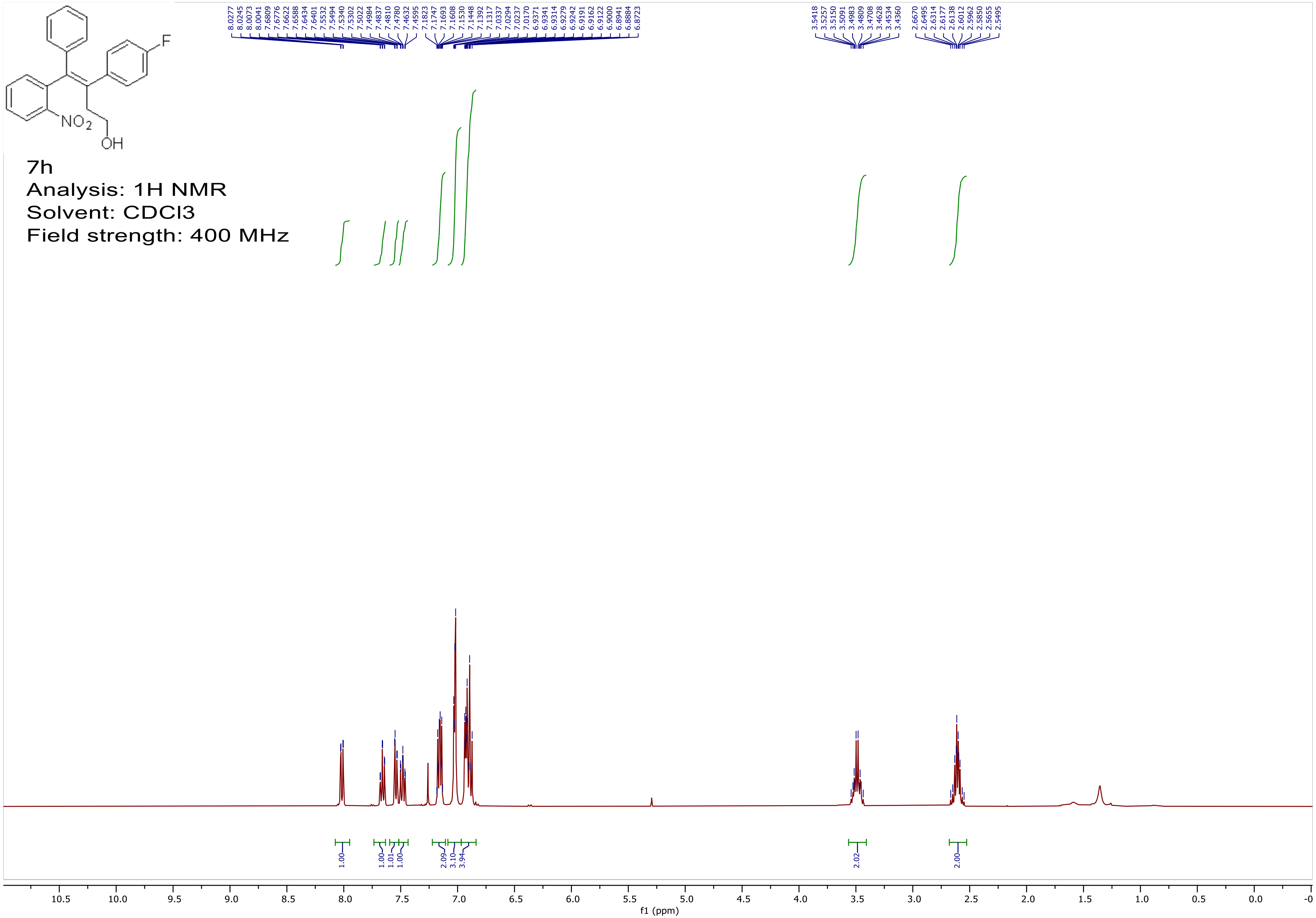
7h
Analysis: 1H NMR
Solvent: CDCl3
Field strength: 400 MHz
f1 (ppm)

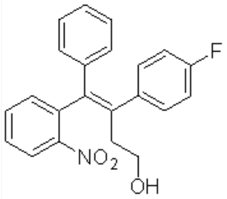

7h
Analysis: 13C NMR
Solvent: CDCl3
Field strength: 101 MHz

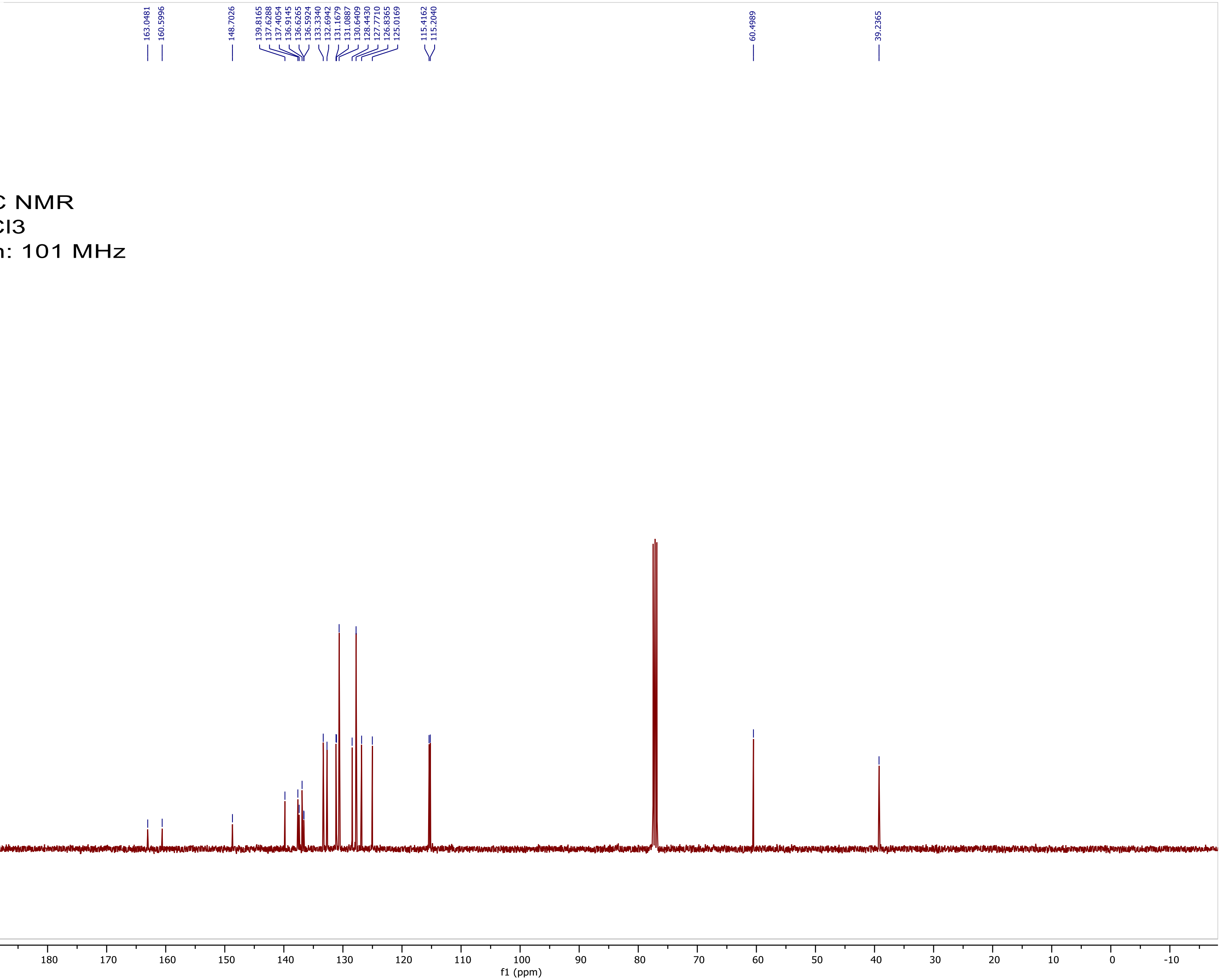

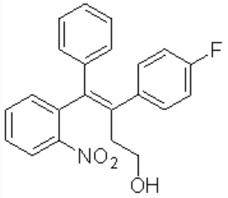

7h
Analysis: 19F NMR
Solvent: CDCl3
Field strength: 376 MHz

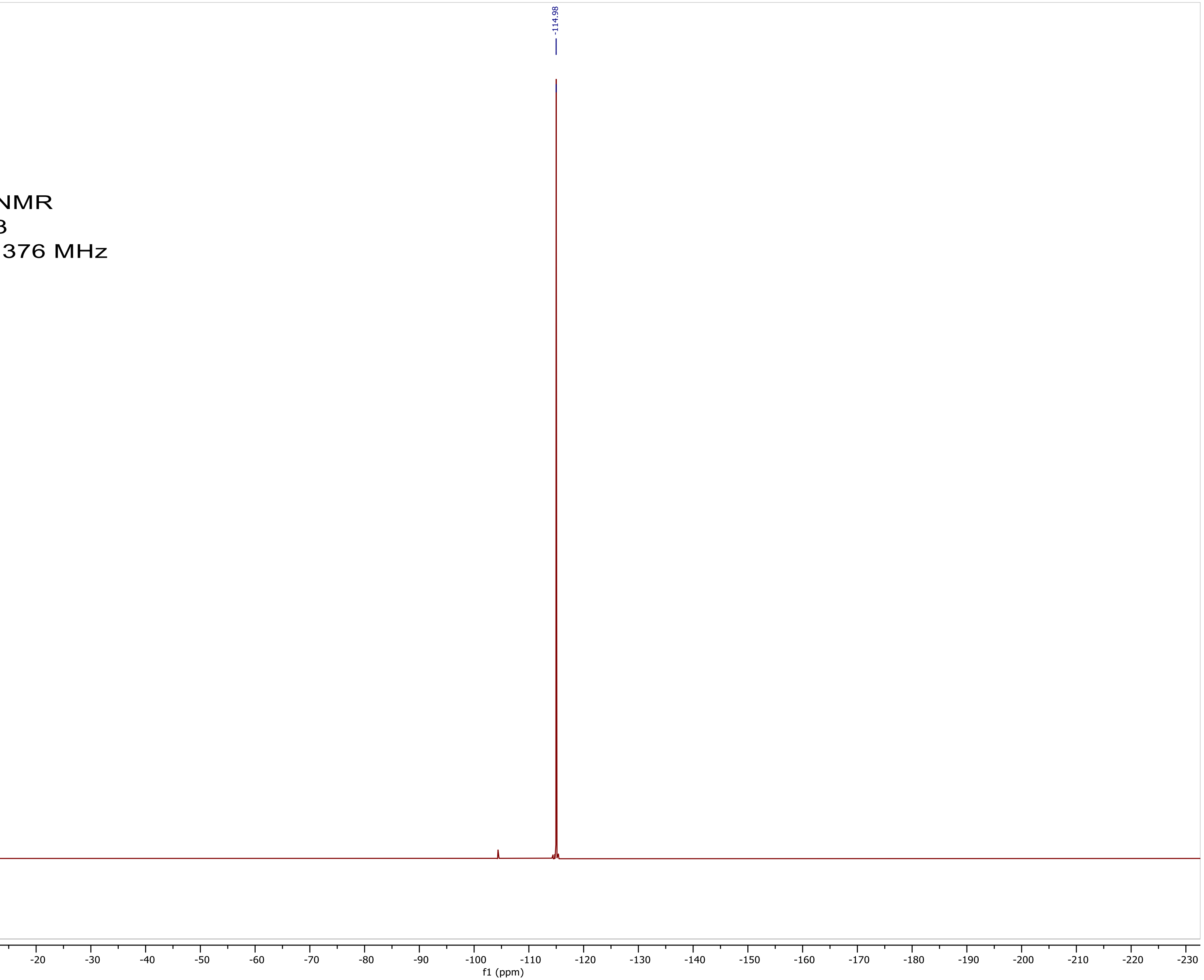

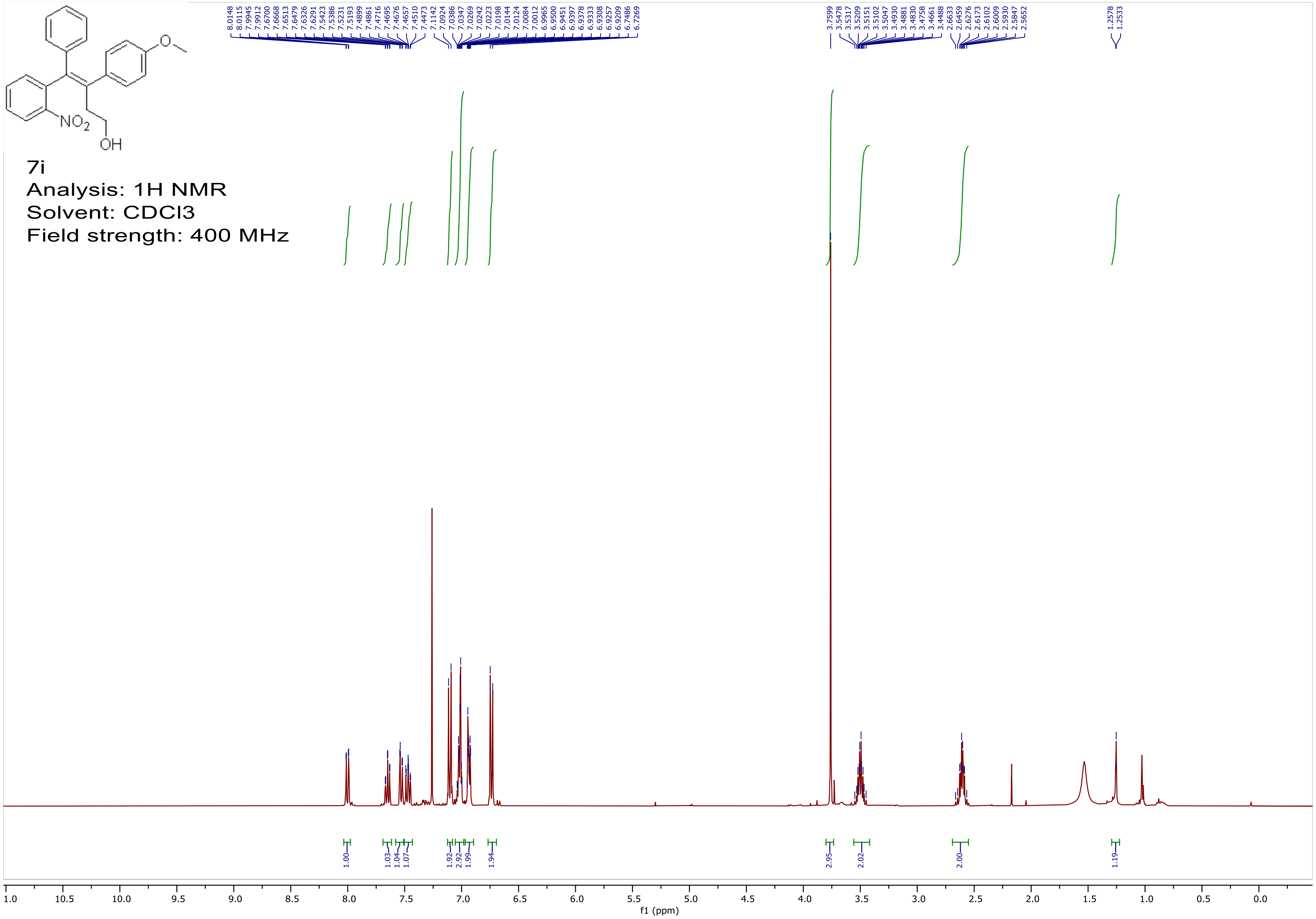
NO2
OH
7i
Analysis: 1H NMR
Solvent: CDCl3
Field strength: 400 MHz
8.0148 8.0115 7.9945 7.9912 7.6700 7.6668 7.6513 7.6479 7.6326 7.6291 7.5423 7.5386 7.5231 7.5193 7.4899 7.4861 7.4716 7.4695 7.4676 7.4657 7.4510 7.4473 7.1142 7.0924 7.0386 7.0347 7.0269 7.0242 7.0223 7.0198 7.0144 7.0124 7.0084 7.0012 6.9965 6.9500 6.9451 6.9397 6.9378 6.9333 6.9308 6.9257 6.9209 6.7486 6.7269
3.7599 3.5478 3.5317 3.5209 3.5151 3.5102 3.5047 3.4930 3.4881 3.4830 3.4758 3.4661 3.4488 2.6633 2.6459 2.6276 2.6173 2.6102 2.6009 2.5930 2.5847 2.5652
1.2578 1.2533
1.00 1.03 1.04 1.07 1.92 2.92 1.99 1.94 2.95 2.02 2.00 1.19
1.0 10.5 10.0 9.5 9.0 8.5 8.0 7.5 7.0 6.5 6.0 5.5 5.0 4.5 4.0 3.5 3.0 2.5 2.0 1.5 1.0 0.5 0.0
f1 (ppm)

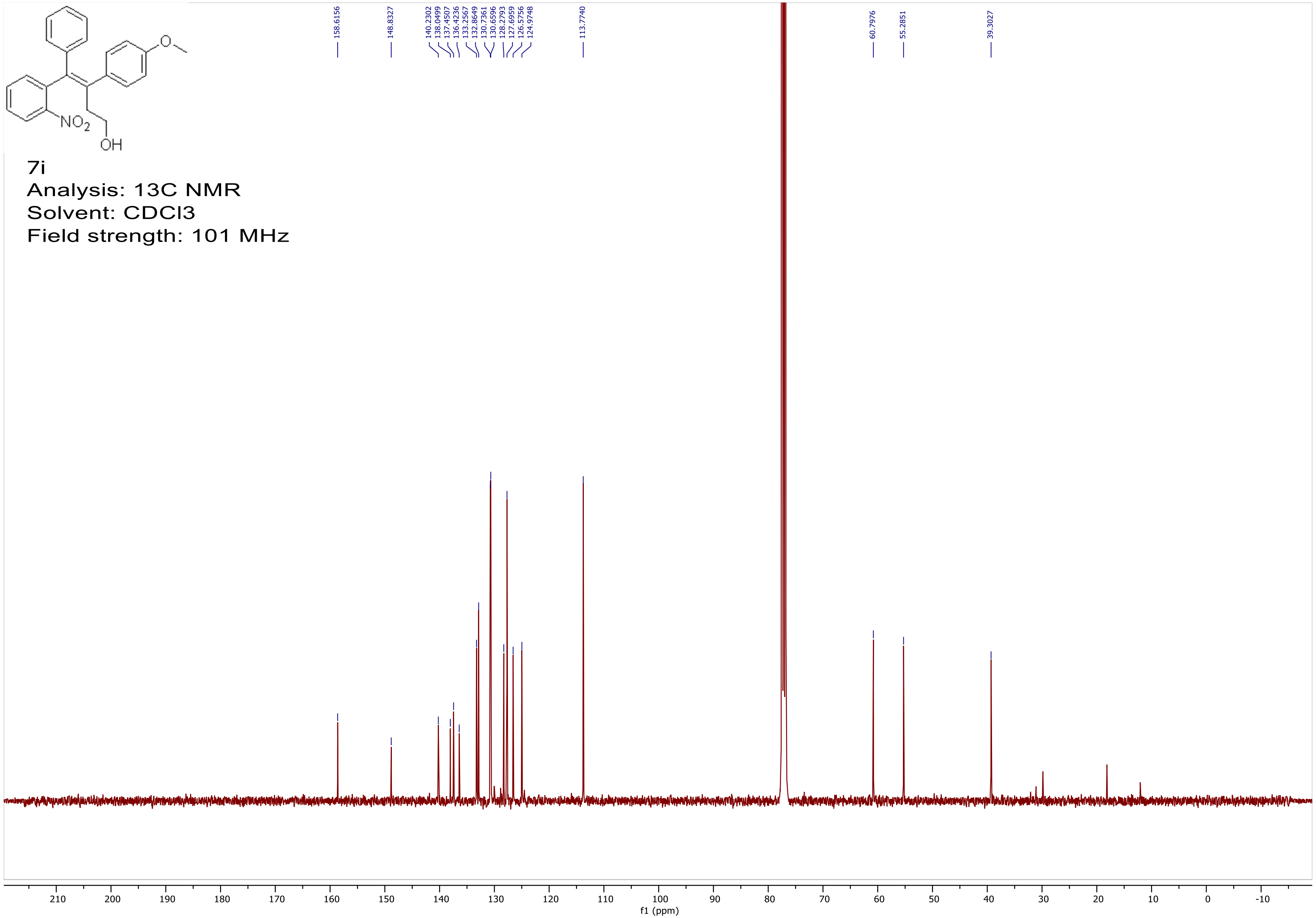
O
NO2
OH
7i
Analysis: 13C NMR
Solvent: CDCl3
Field strength: 101 MHz
158.6156
148.8327
140.2302
138.0499
137.4507
136.4236
133.2567
132.8649
130.7361
130.6596
128.2793
127.6959
126.5756
124.9748
113.7740
60.7976
55.2851
39.3027
210 200 190 180 170 160 150 140 130 120 110 100 90 80 70 60 50 40 30 20 10 0 -10
f1 (ppm)

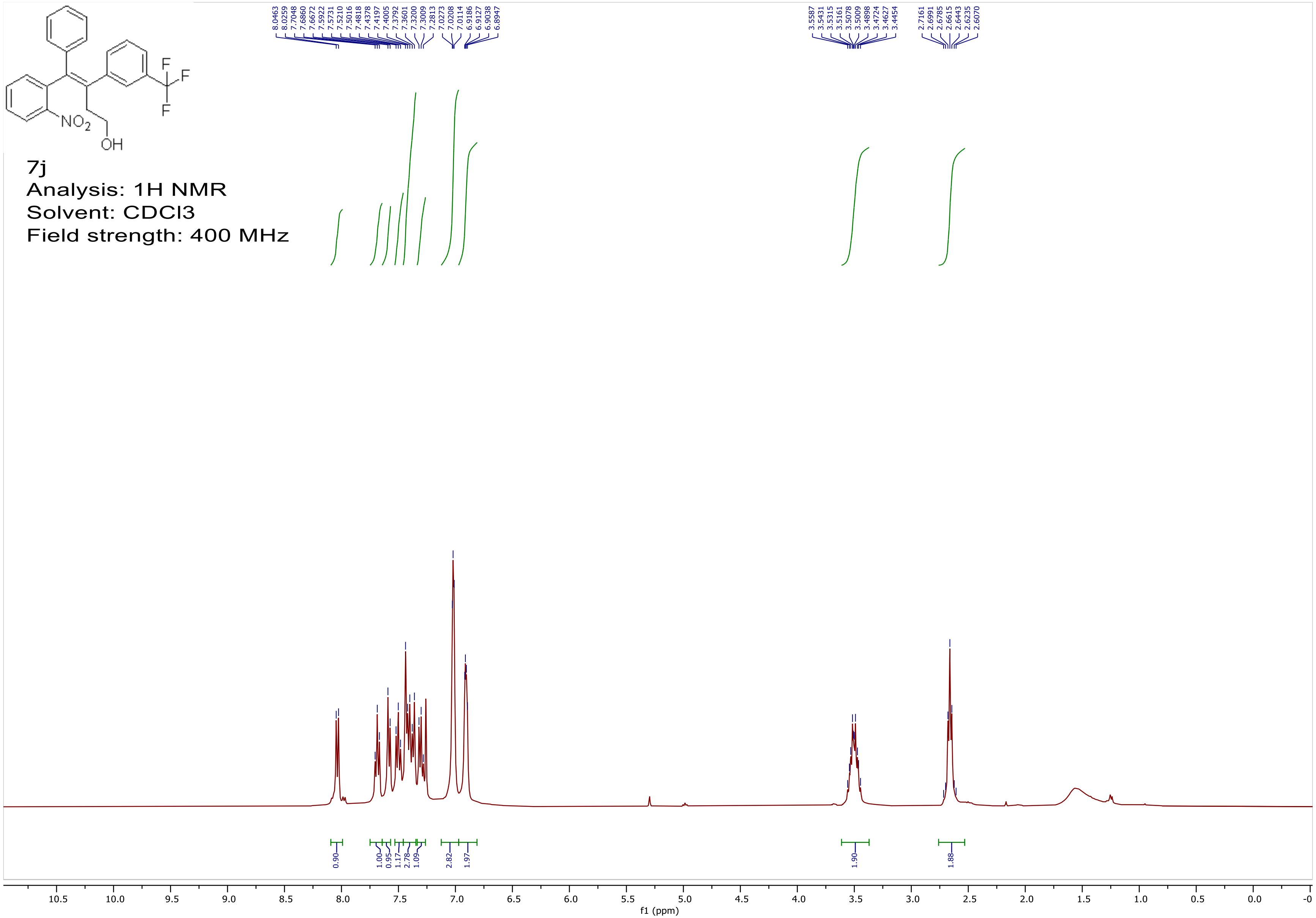

F
F
F
NO2
OH
7j
Analysis: 1H NMR
Solvent: CDCl3
Field strength: 400 MHz
8.0463
8.0259
7.7048
7.6860
7.6672
7.5922
7.5731
7.5210
7.5016
7.4818
7.4378
7.4197
7.4005
7.3792
7.3601
7.3200
7.3009
7.2813
7.0273
7.0208
7.0114
6.9186
6.9127
6.9038
6.8947
3.5587
3.5431
3.5315
3.5161
3.5078
3.5009
3.4898
3.4724
3.4627
3.4454
2.7161
2.6991
2.6785
2.6615
2.6443
2.6235
2.6070
0.90
1.00
0.95
1.17
2.78
1.09
2.82
1.97
1.90
1.88
10.5
10.0
9.5
9.0
8.5
8.0
7.5
7.0
6.5
6.0
5.5
5.0
4.5
4.0
3.5
3.0
2.5
2.0
1.5
1.0
0.5
0.0
-0
f1 (ppm)

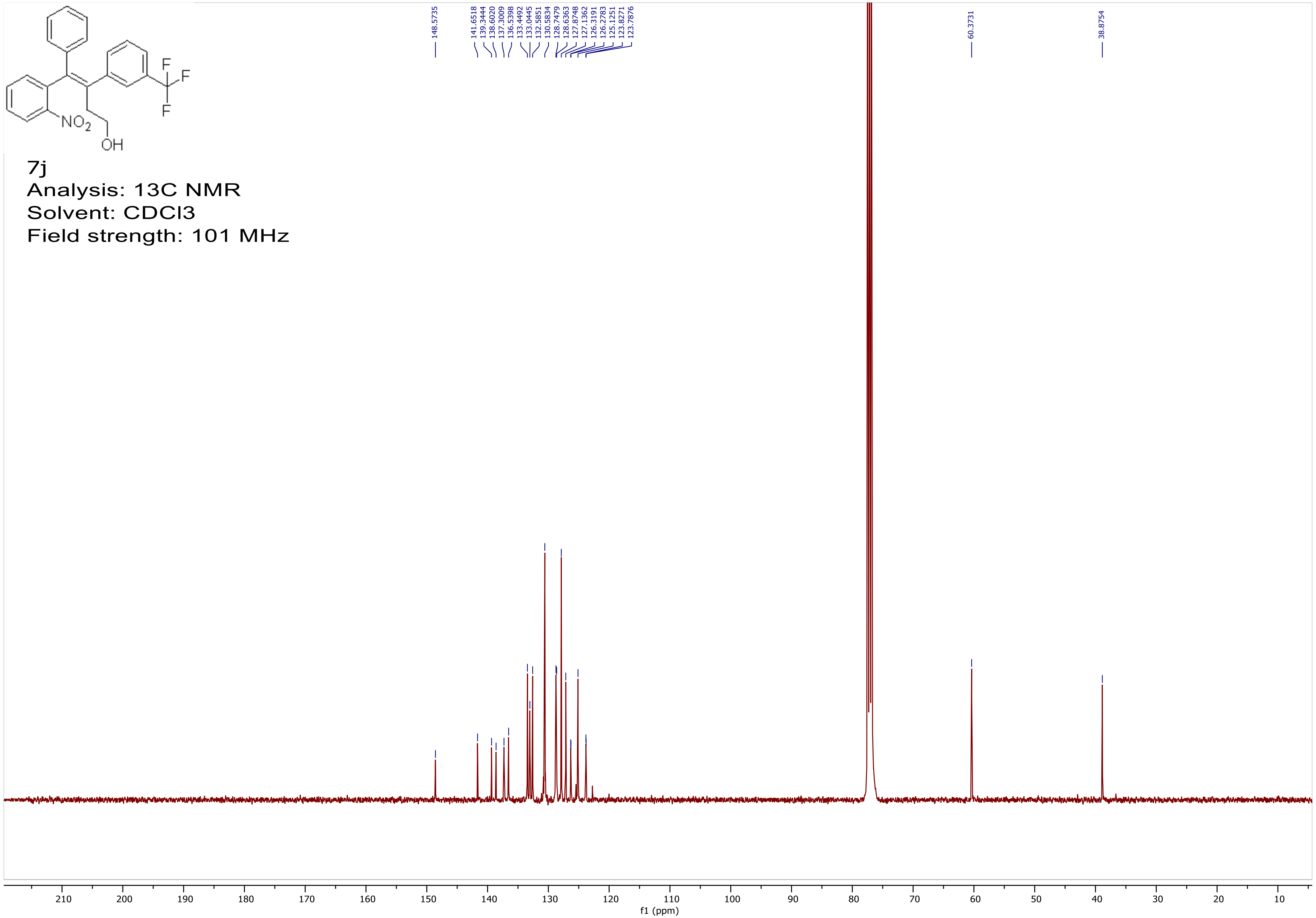
F
F
F
NO2
OH
7j
Analysis: 13C NMR
Solvent: CDCl3
Field strength: 101 MHz
148.5735
141.6518
139.3444
138.6020
137.3009
136.5398
133.4492
133.0445
132.5851
130.5834
128.7479
128.6363
127.8748
127.1362
126.3191
126.2783
125.1251
123.8271
123.7876
60.3731
38.8754
210
200
190
180
170
160
150
140
130
120
110
100
90
80
70
60
50
40
30
20
10
f1 (ppm)

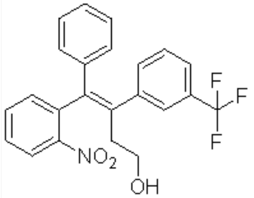

7j
Analysis: 19F NMR
Solvent: CDCl3
Field strength: 376 MHz

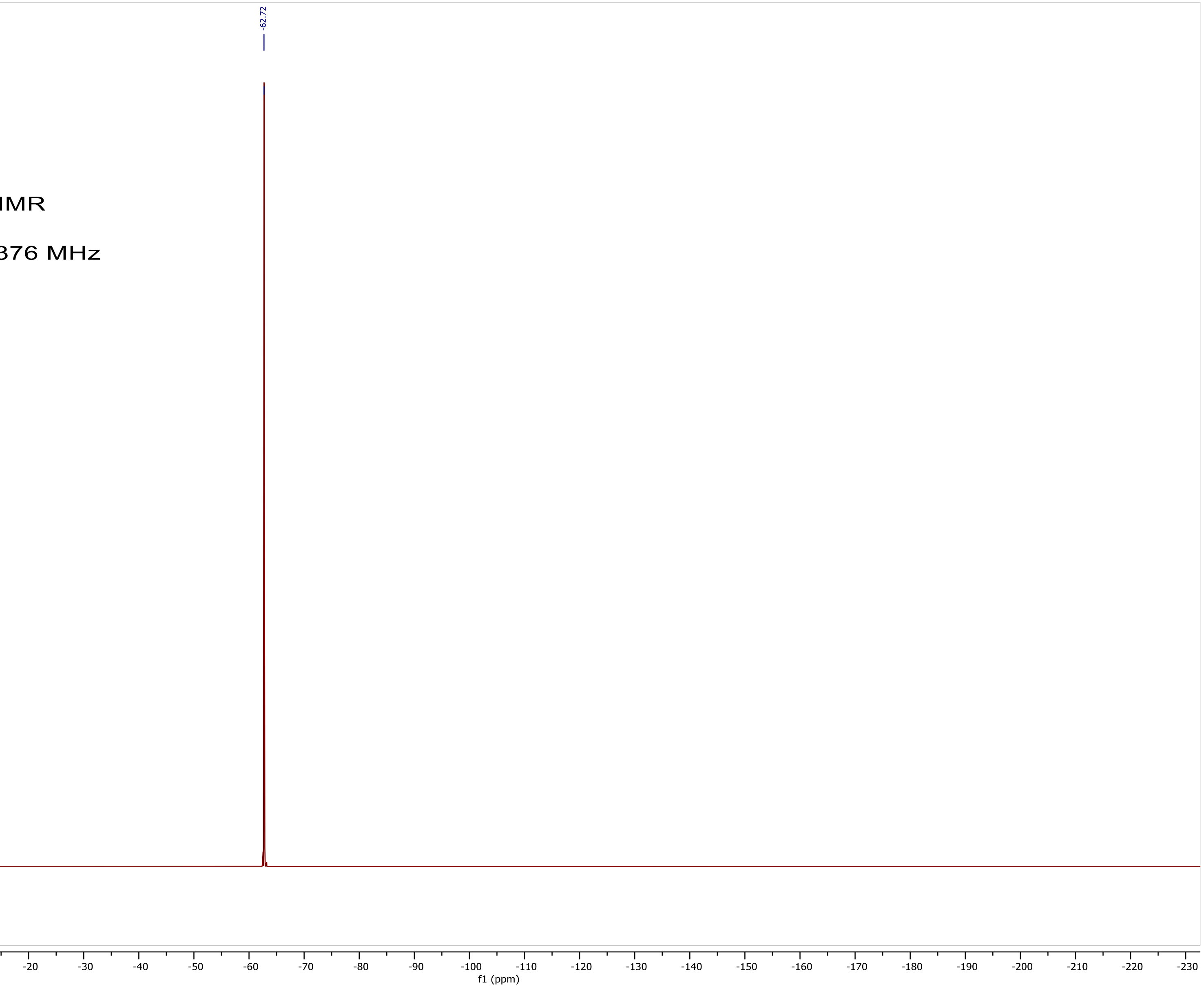

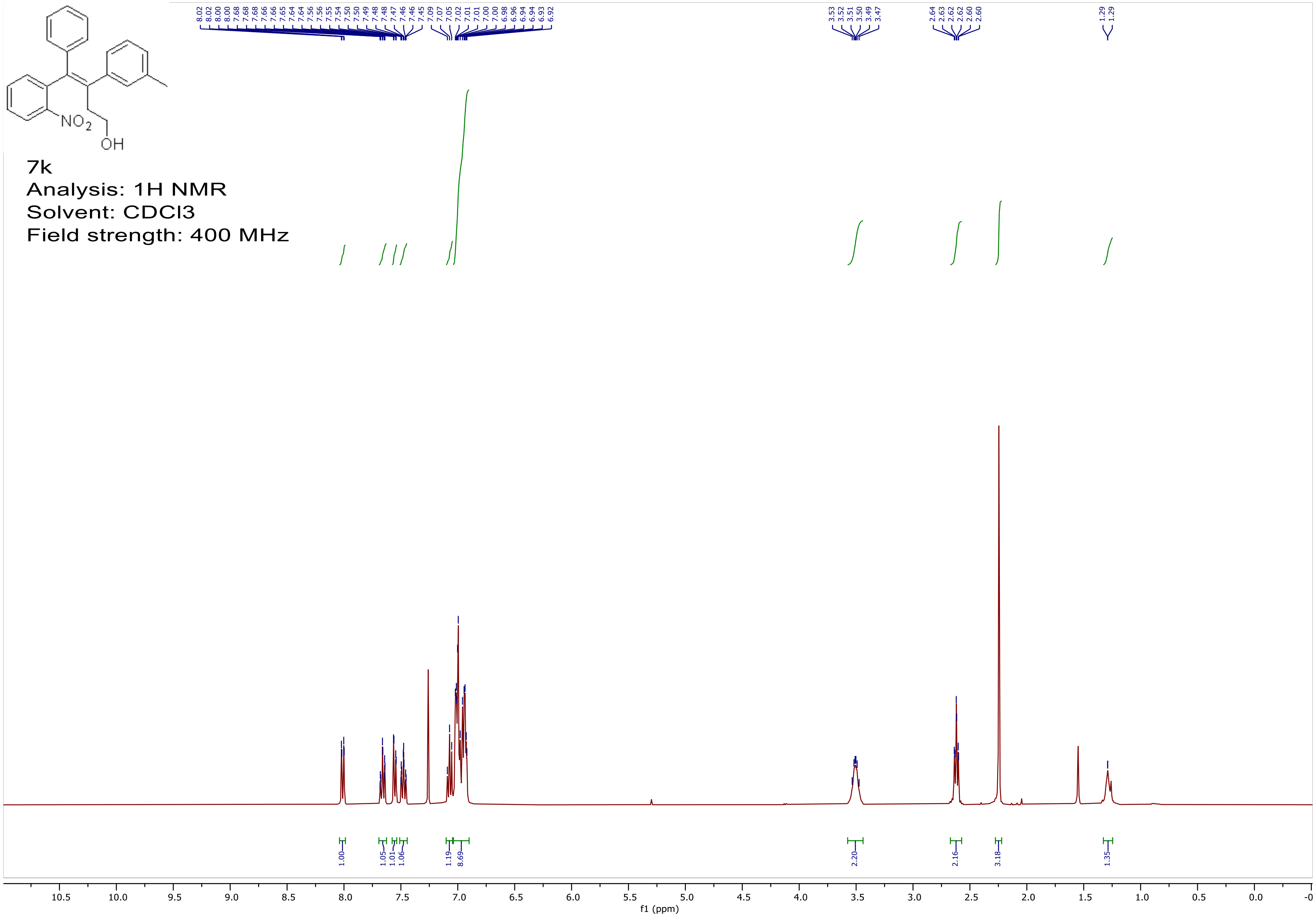
7k
Analysis: 1H NMR
Solvent: CDCl3
Field strength: 400 MHz
NO2
OH
f1 (ppm)

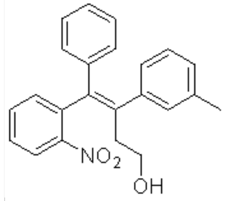

7k
Analysis: 13C NMR
Solvent: CDCl3
Field strength: 101 MHz

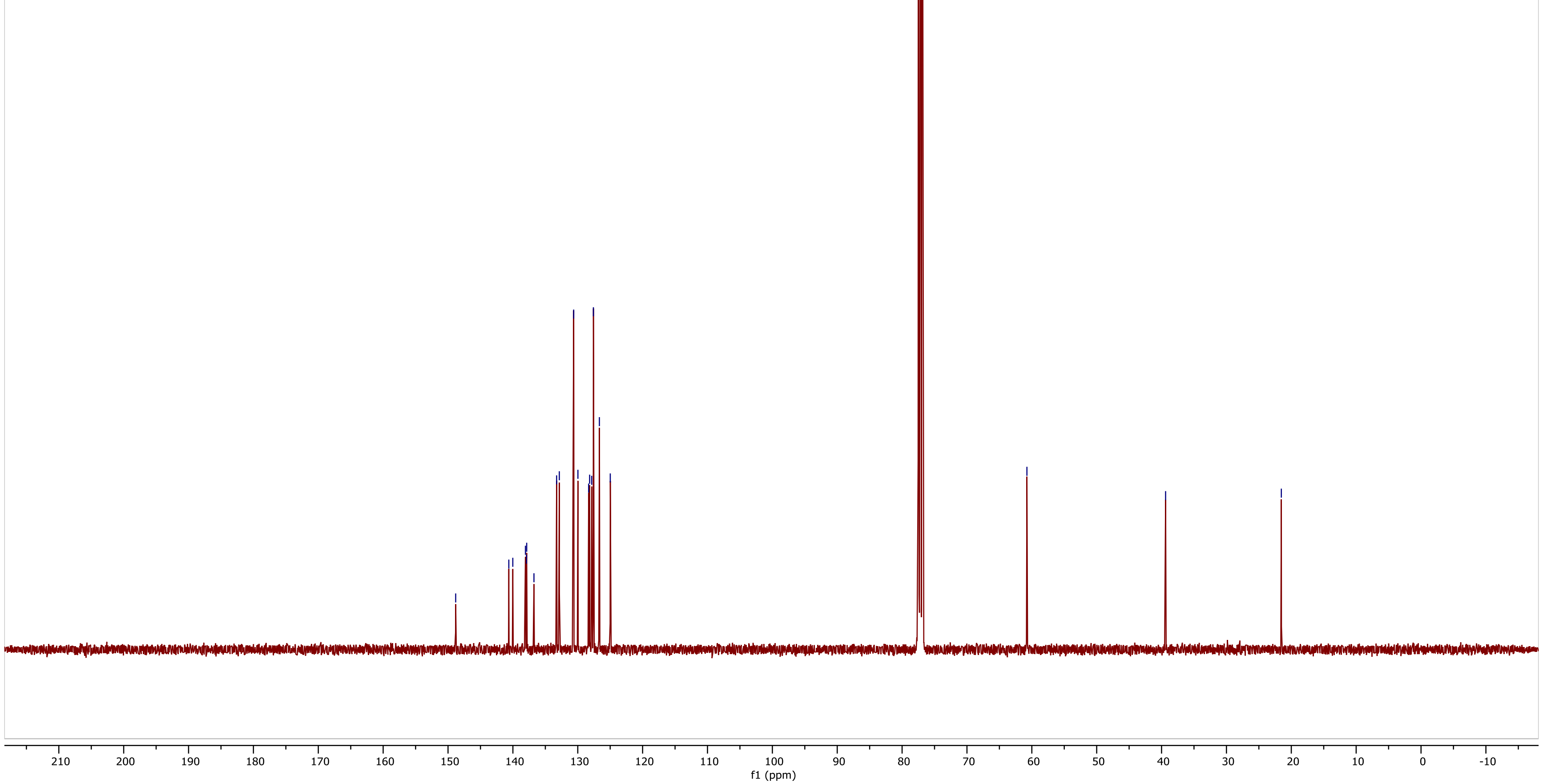

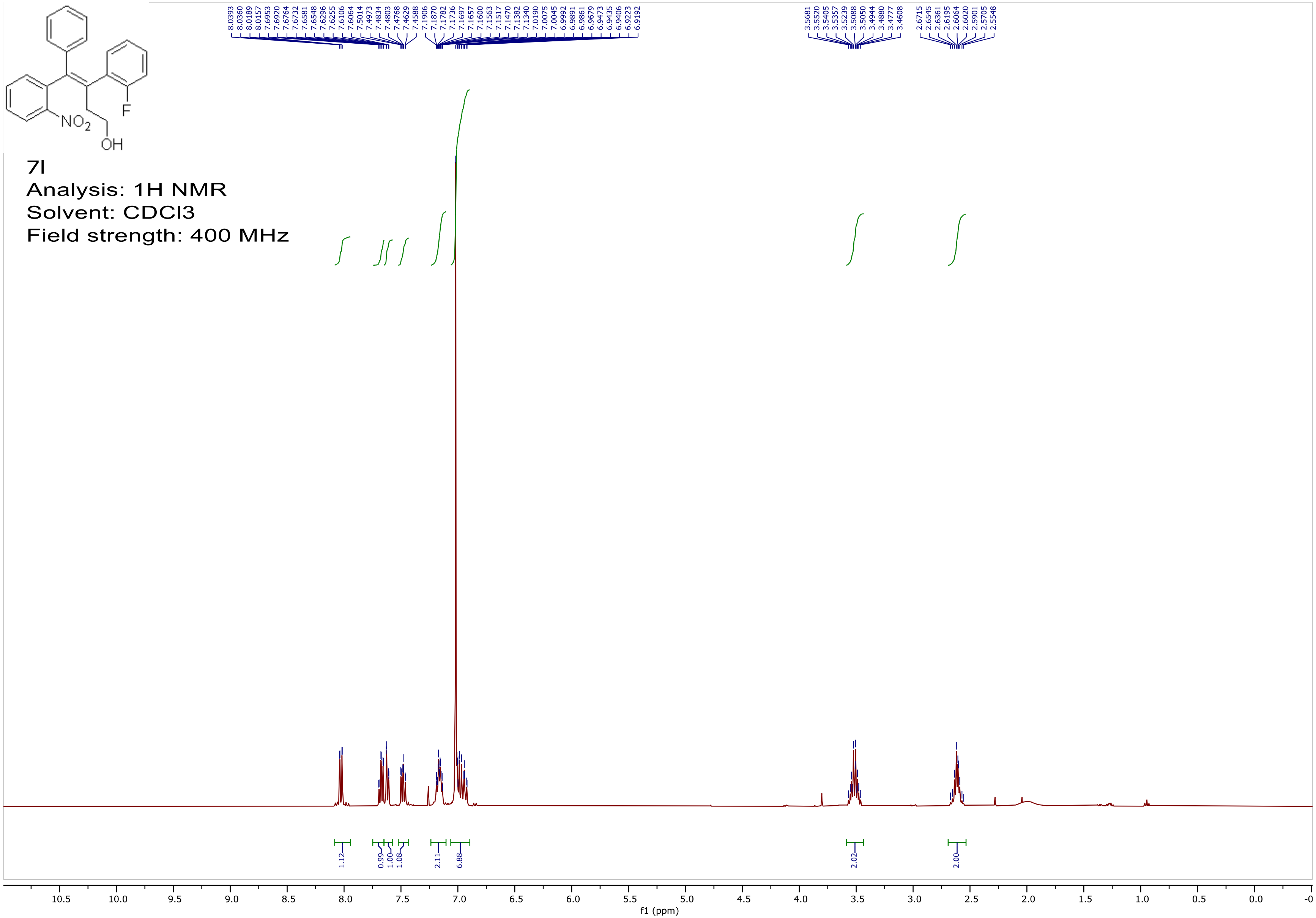
7l
Analysis: 1H NMR
Solvent: CDCl3
Field strength: 400 MHz
NO2
F
OH
1.12
0.99
1.00
1.08
2.11
6.88
2.02
2.00
f1 (ppm)

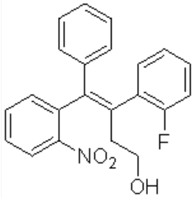

7l
Analysis: 13C NMR
Solvent: CDCl3
Field strength: 101 MHz

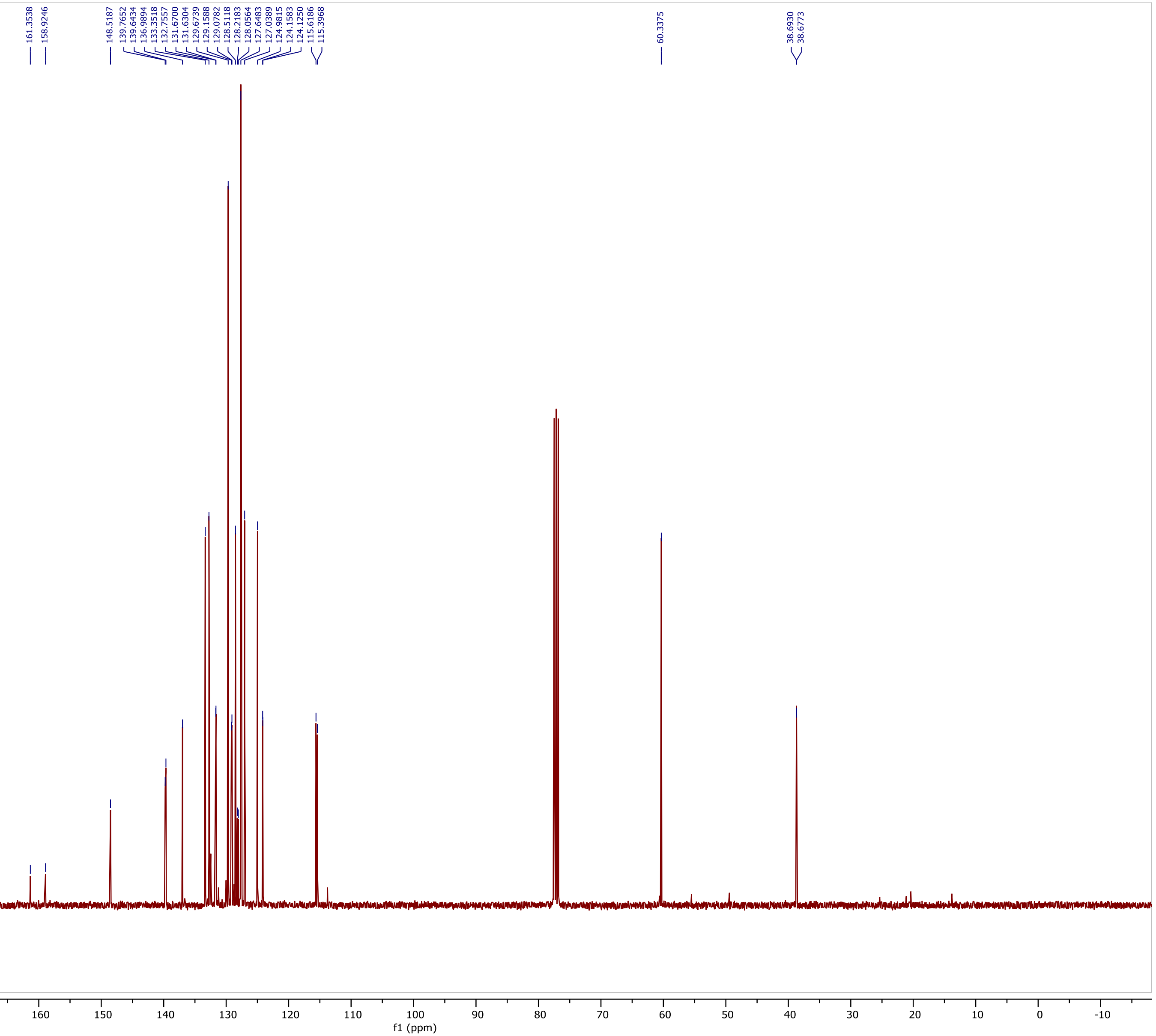

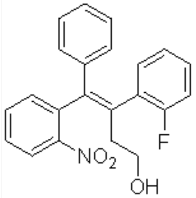

7l
Analysis: 19F NMR
Solvent: CDCl3
Field strength: 376 MHz

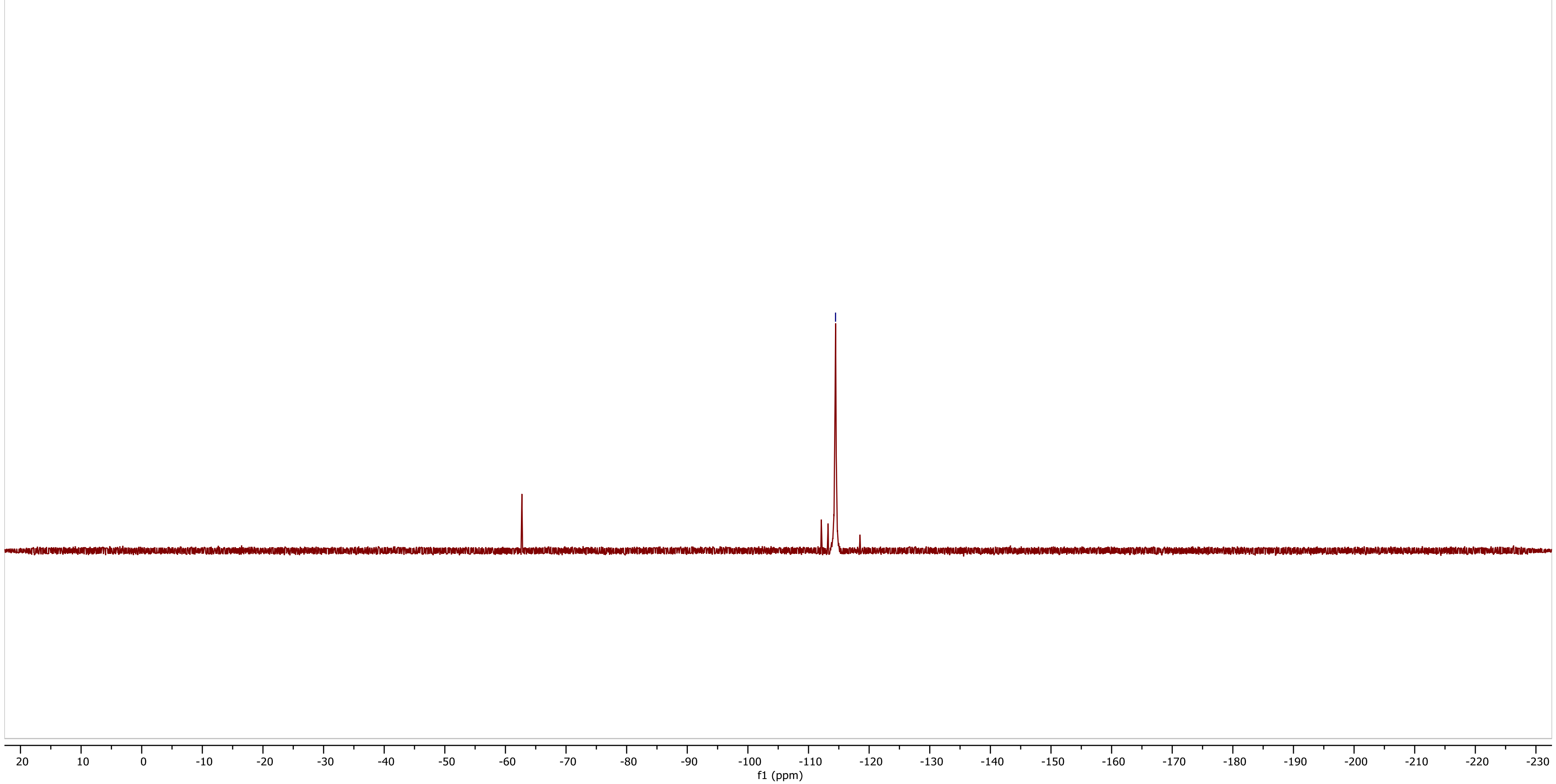

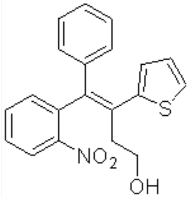

7m (isomer 1)
Analysis: 1H NMR
Solvent: CDCl3
Field strength: 400 MHz

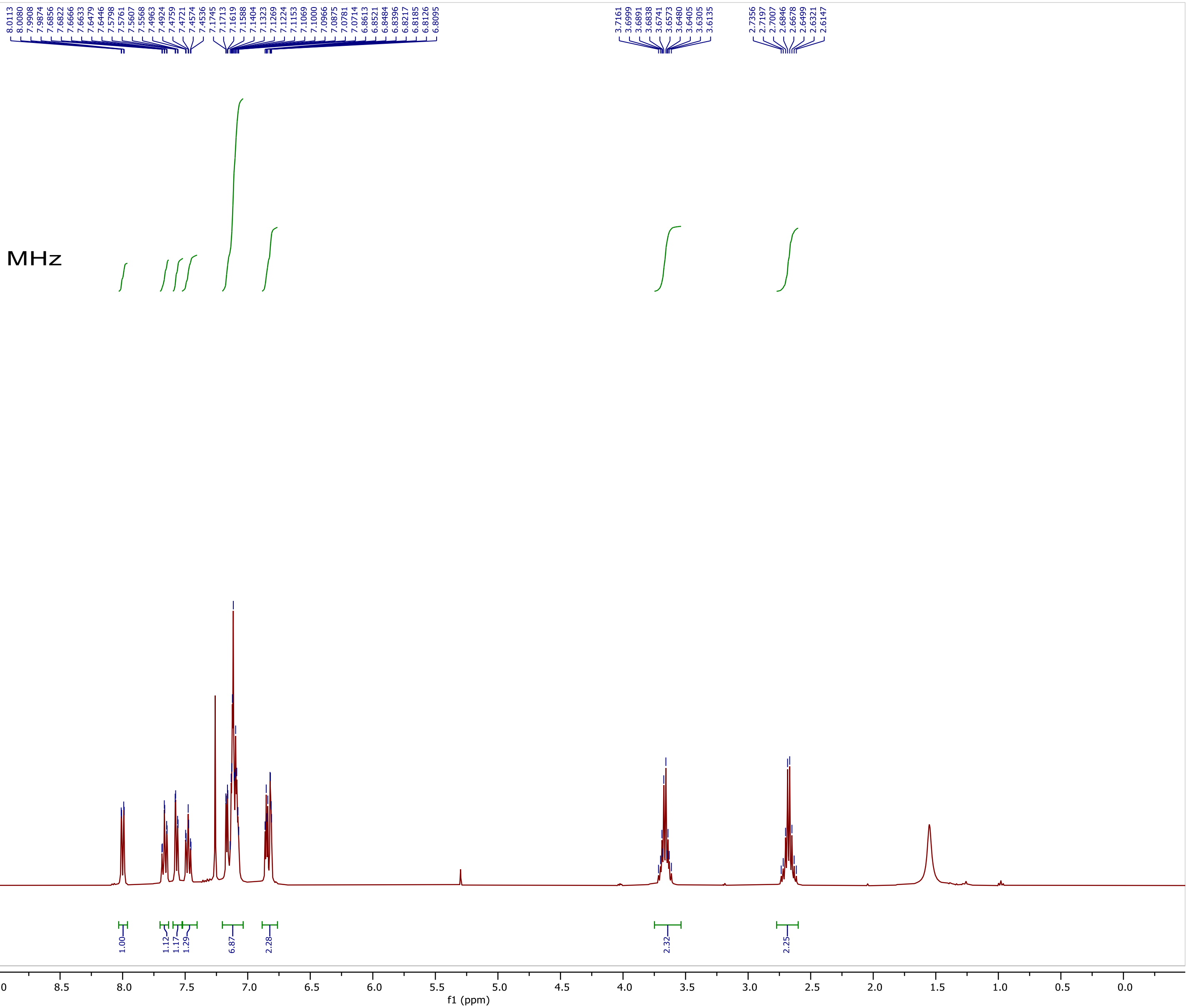

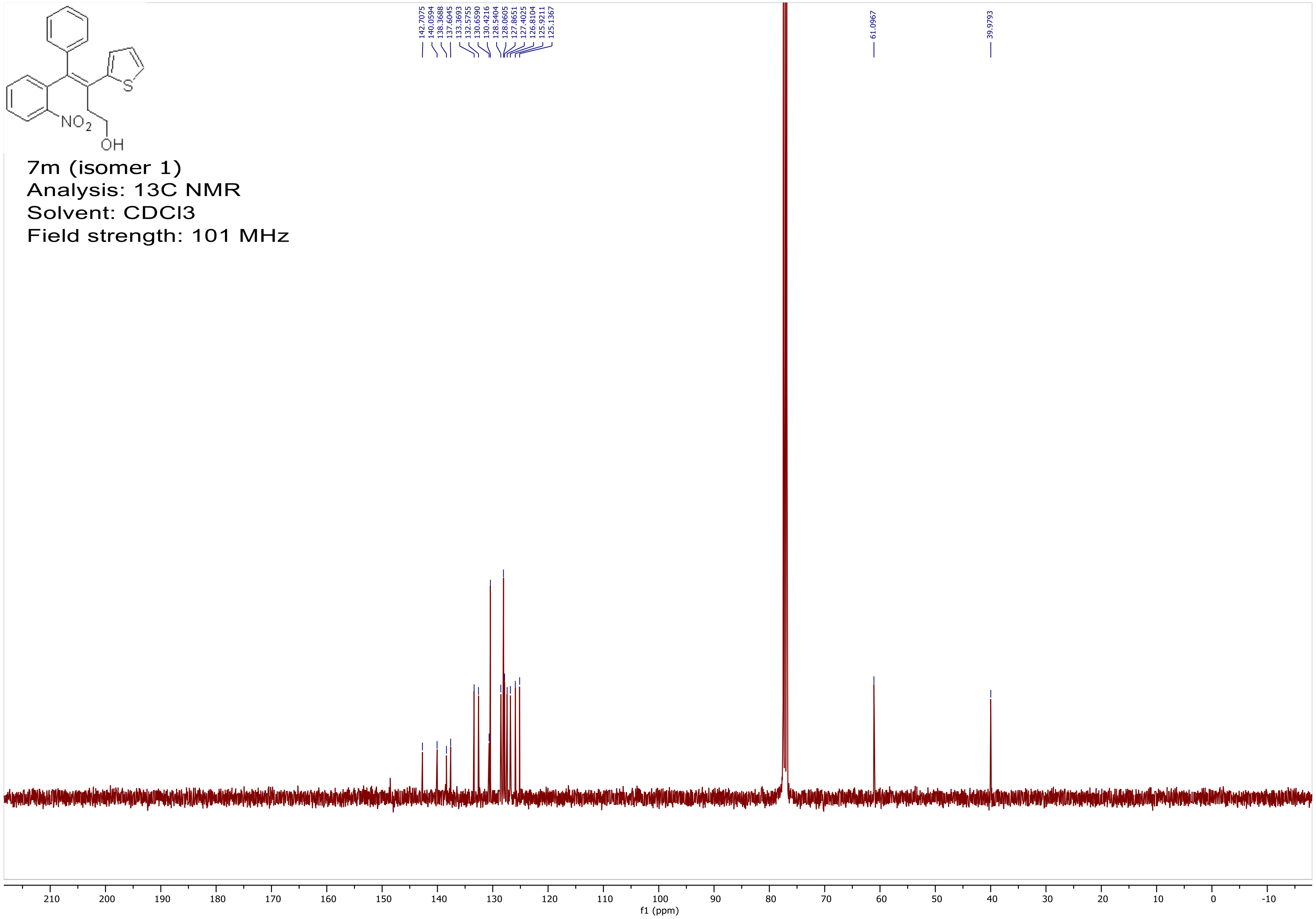
NO2
OH
S
7m (isomer 1)
Analysis: 13C NMR
Solvent: CDCl3
Field strength: 101 MHz
142.7075
140.0594
138.3688
137.6045
133.3693
132.5755
130.6590
130.4216
128.5404
128.0605
127.8651
127.4025
126.8104
125.9211
125.1367
61.0967
39.9793
210 200 190 180 170 160 150 140 130 120 110 100 90 80 70 60 50 40 30 20 10 0 -10
f1 (ppm)

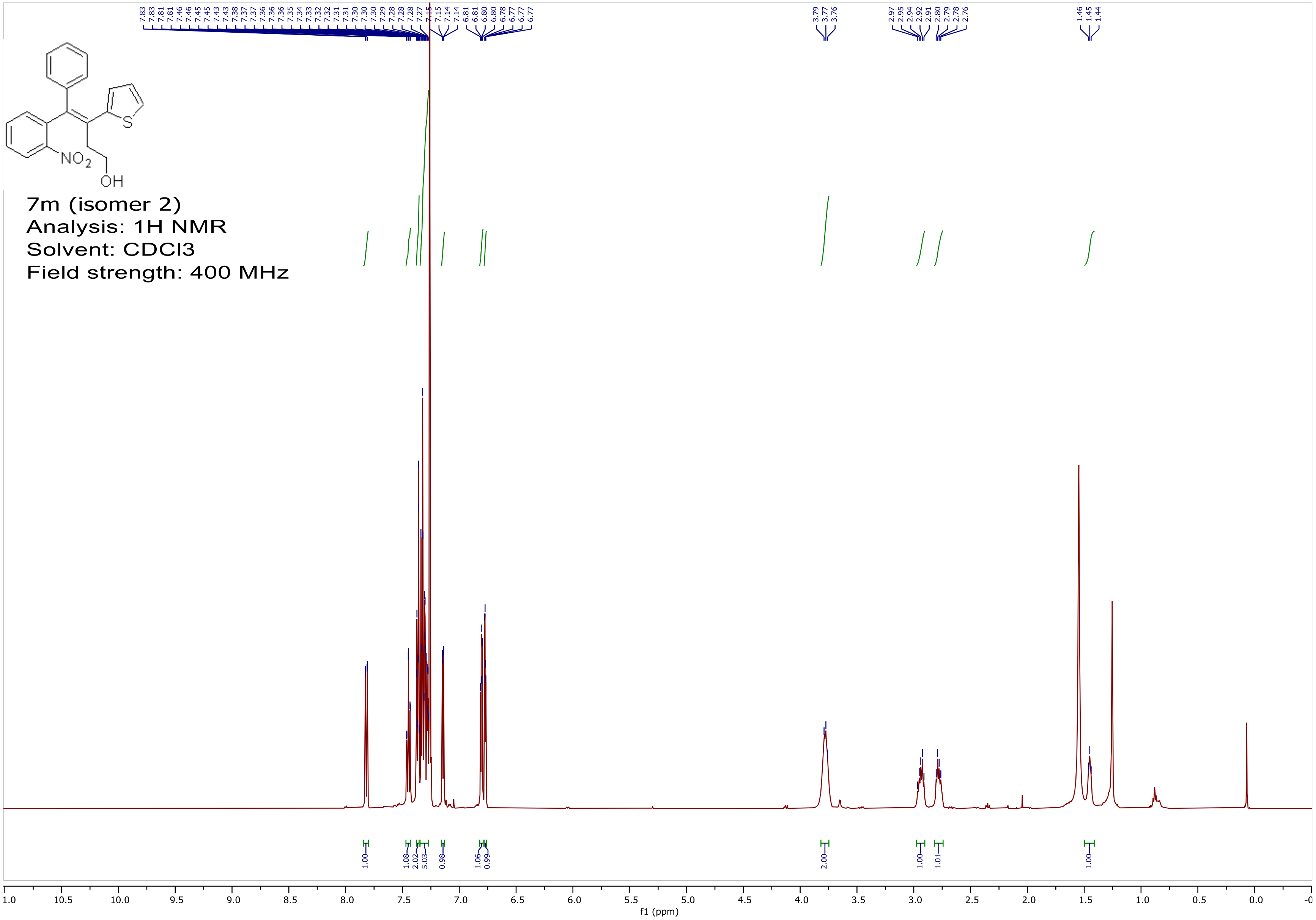
NO2
OH
S
7m (isomer 2)
Analysis: 1H NMR
Solvent: CDCl3
Field strength: 400 MHz
f1 (ppm)

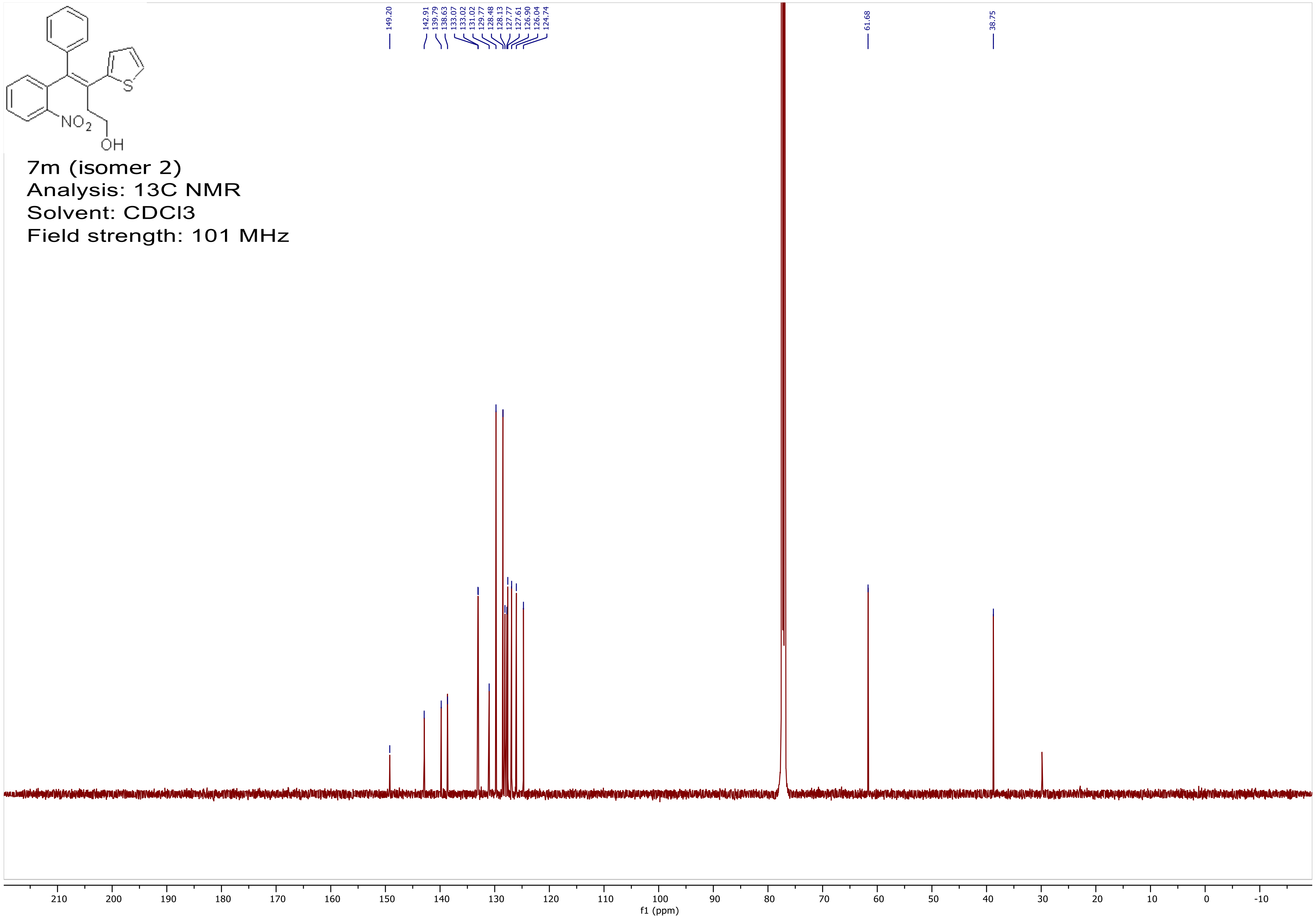

NO2
OH
S
7m (isomer 2)
Analysis: 13C NMR
Solvent: CDCl3
Field strength: 101 MHz
149.20
142.91
139.79
138.63
133.07
133.02
131.02
129.77
128.48
128.13
127.77
127.61
126.90
126.04
124.74
61.68
38.75
210
200
190
180
170
160
150
140
130
120
110
100
90
80
70
60
50
40
30
20
10
0
-10
f1 (ppm)

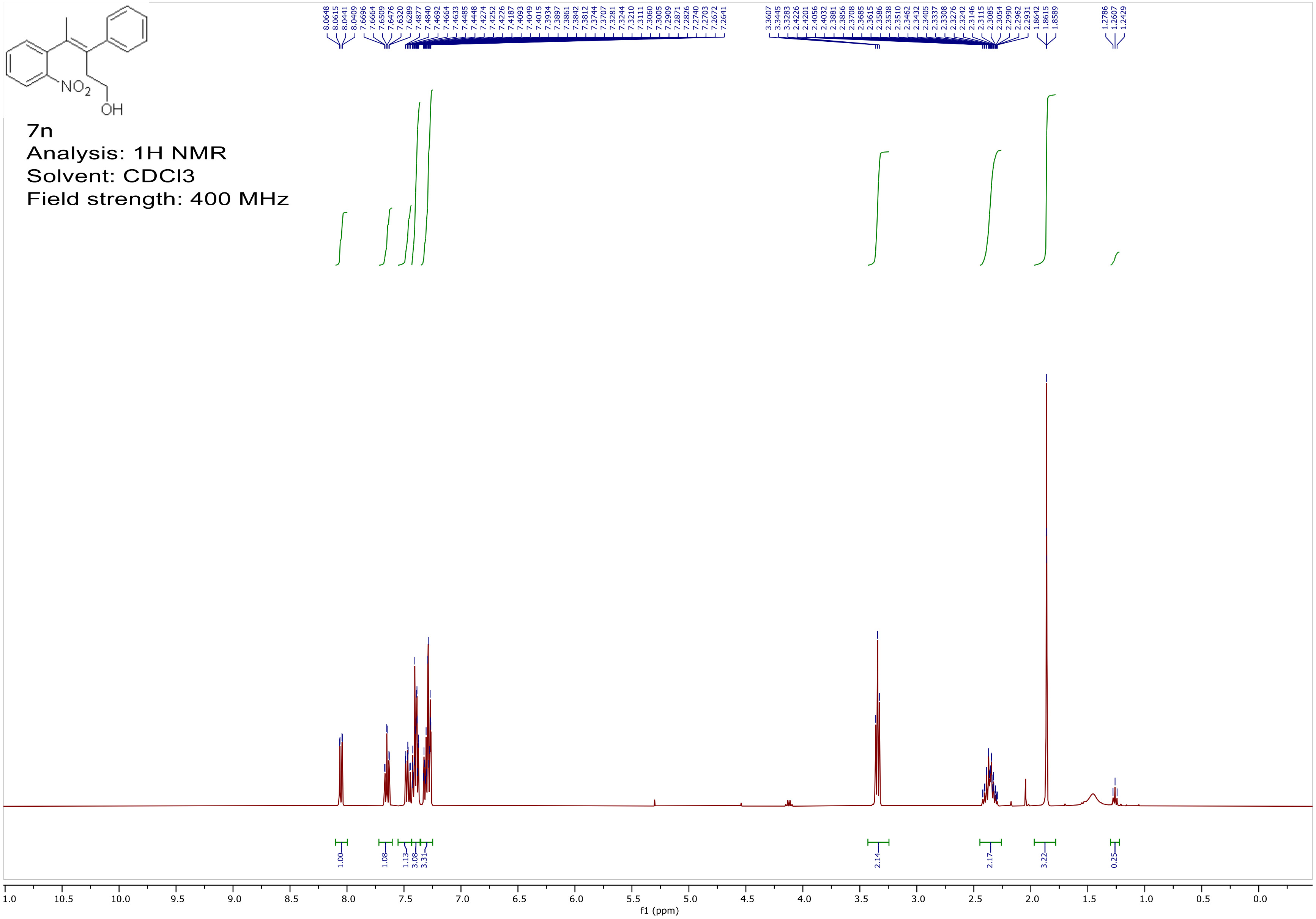

NO2
OH
7n
Analysis: 1H NMR
Solvent: CDCl3
Field strength: 400 MHz
8.0648 8.0615 8.0441 8.0409 7.6696 7.6664 7.6509 7.6476 7.6320 7.6289 7.4877 7.4840 7.4692 7.4664 7.4633 7.4485 7.4448 7.4274 7.4252 7.4226 7.4187 7.4093 7.4049 7.4015 7.3934 7.3897 7.3861 7.3842 7.3812 7.3744 7.3707 7.3281 7.3244 7.3210 7.3111 7.3060 7.3005 7.2909 7.2871 7.2826 7.2740 7.2703 7.2672 7.2641
3.3607 3.3445 3.3283 2.4226 2.4201 2.4056 2.4032 2.3881 2.3856 2.3708 2.3685 2.3615 2.3586 2.3538 2.3510 2.3462 2.3432 2.3405 2.3337 2.3308 2.3276 2.3242 2.3146 2.3115 2.3085 2.3054 2.2990 2.2962 2.2931 1.8642 1.8615 1.8589
1.2786 1.2607 1.2429
1.00 1.08 1.13 3.08 3.31 2.14 2.17 3.22 0.25
1.0 10.5 10.0 9.5 9.0 8.5 8.0 7.5 7.0 6.5 6.0 5.5 5.0 4.5 4.0 3.5 3.0 2.5 2.0 1.5 1.0 0.5 0.0
f1 (ppm)

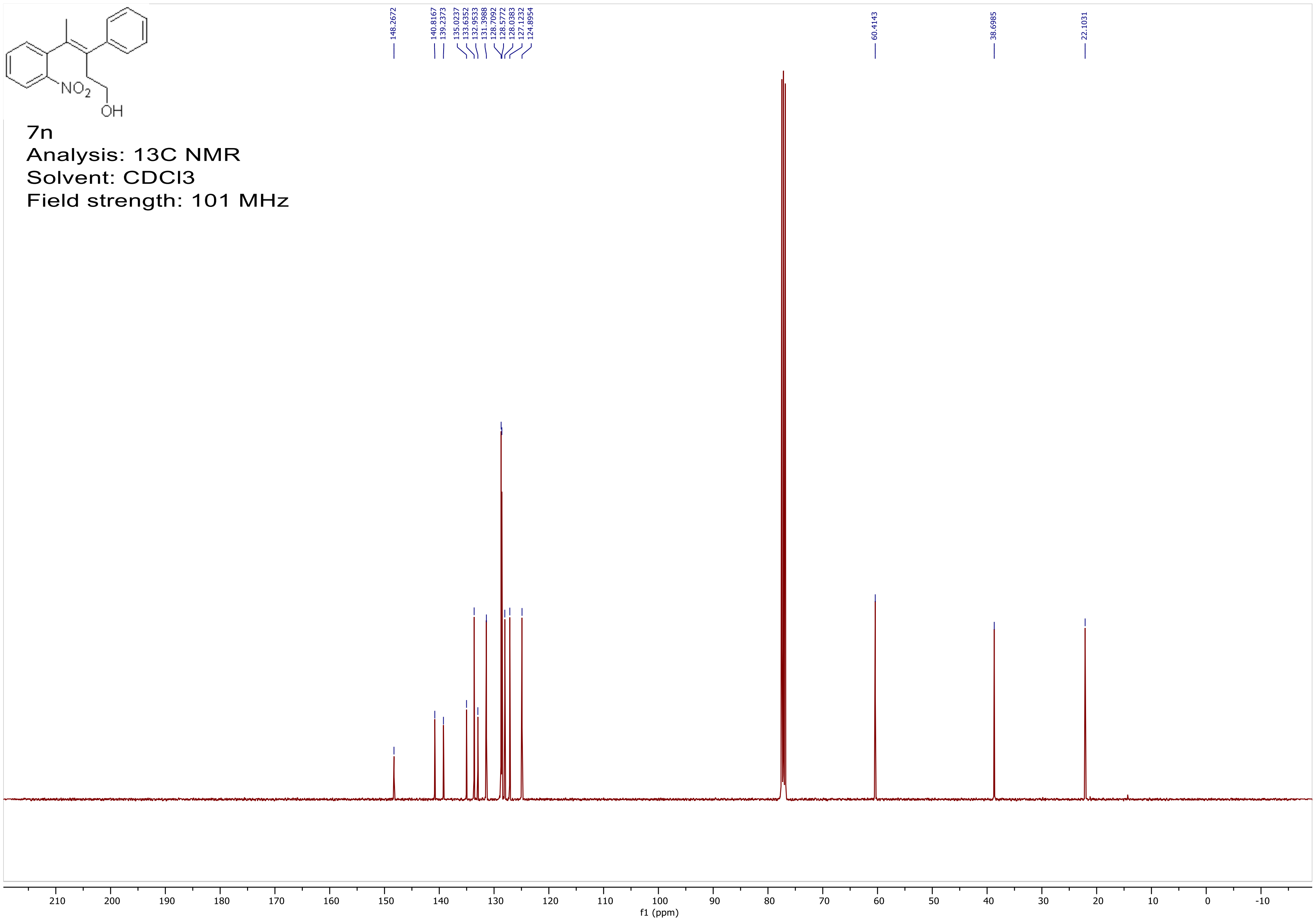
NO2
OH
7n
Analysis: 13C NMR
Solvent: CDCl3
Field strength: 101 MHz
148.2672
140.8167
139.2373
135.0237
133.6352
132.9533
131.3988
128.7092
128.5772
128.0383
127.1232
124.8954
60.4143
38.6985
22.1031
210 200 190 180 170 160 150 140 130 120 110 100 90 80 70 60 50 40 30 20 10 0 -10
f1 (ppm)

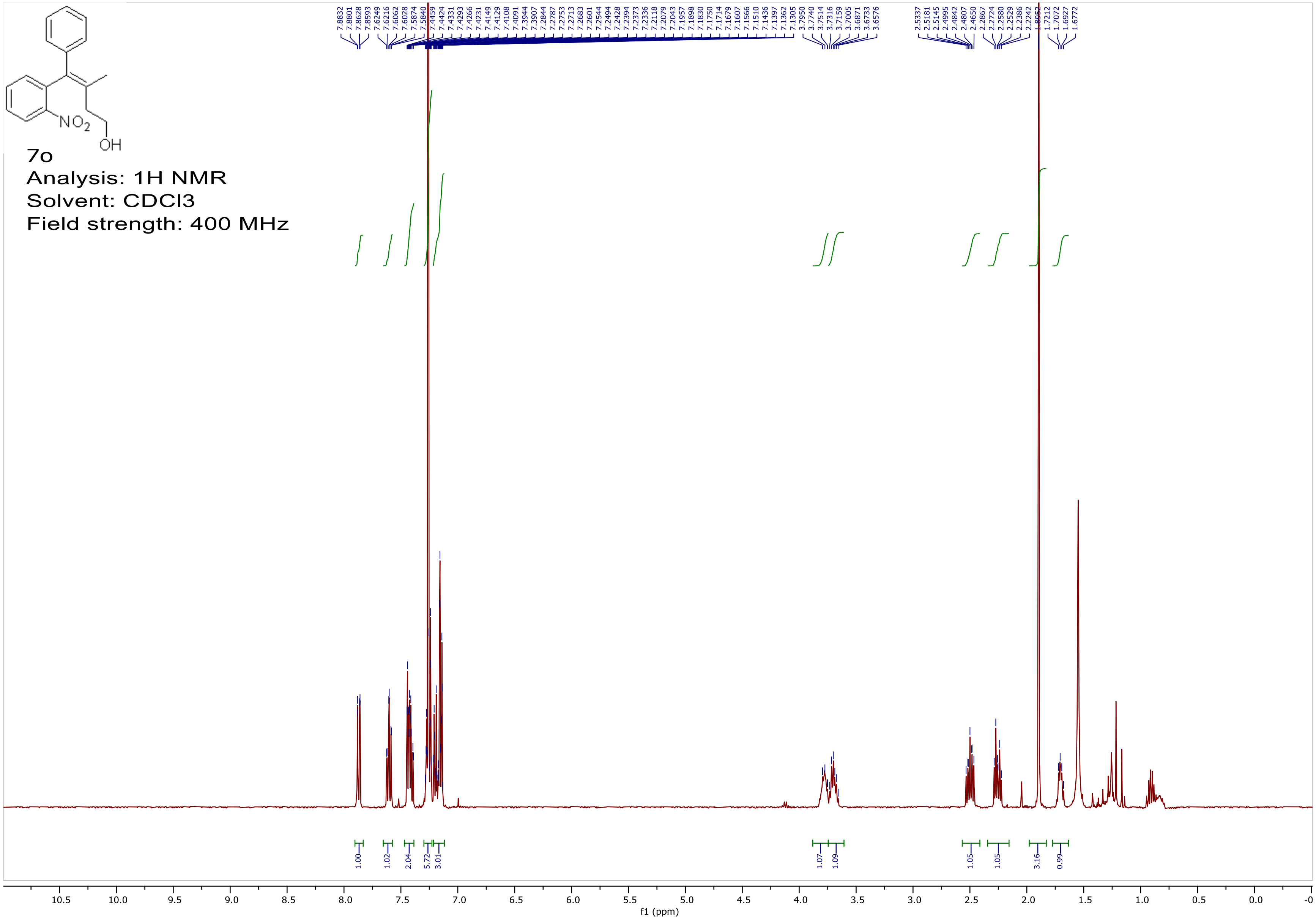

NO2
OH
7o
Analysis: 1H NMR
Solvent: CDCl3
Field strength: 400 MHz
f1 (ppm)

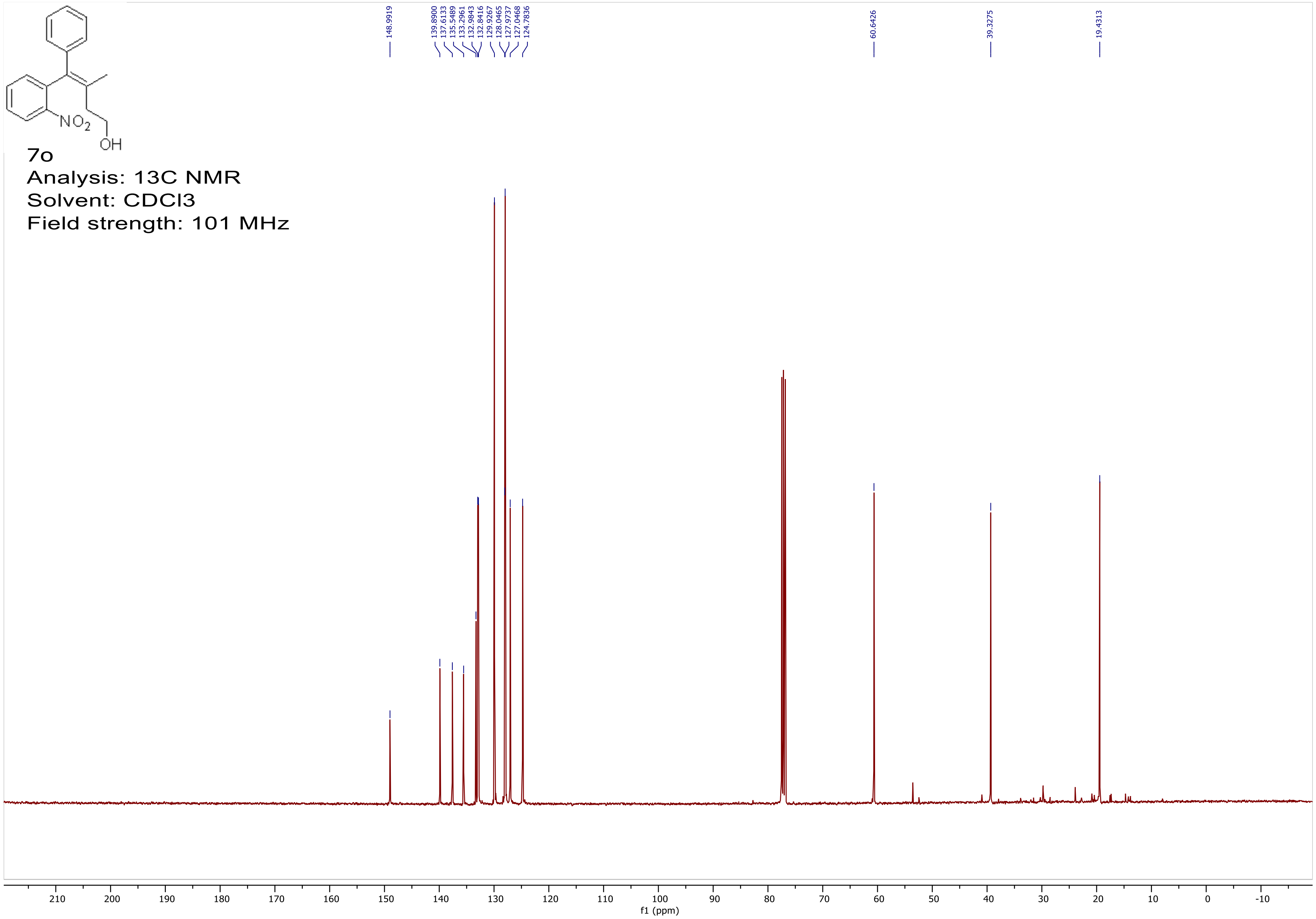

NO2
OH
7o
Analysis: 13C NMR
Solvent: CDCl3
Field strength: 101 MHz
148.9919
139.8900
137.6133
135.5489
133.2961
132.9843
132.8416
129.9267
128.0465
127.9737
127.0468
124.7836
60.6426
39.3275
19.4313
210
200
190
180
170
160
150
140
130
120
110
100
90
80
70
60
50
40
30
20
10
0
-10
f1 (ppm)

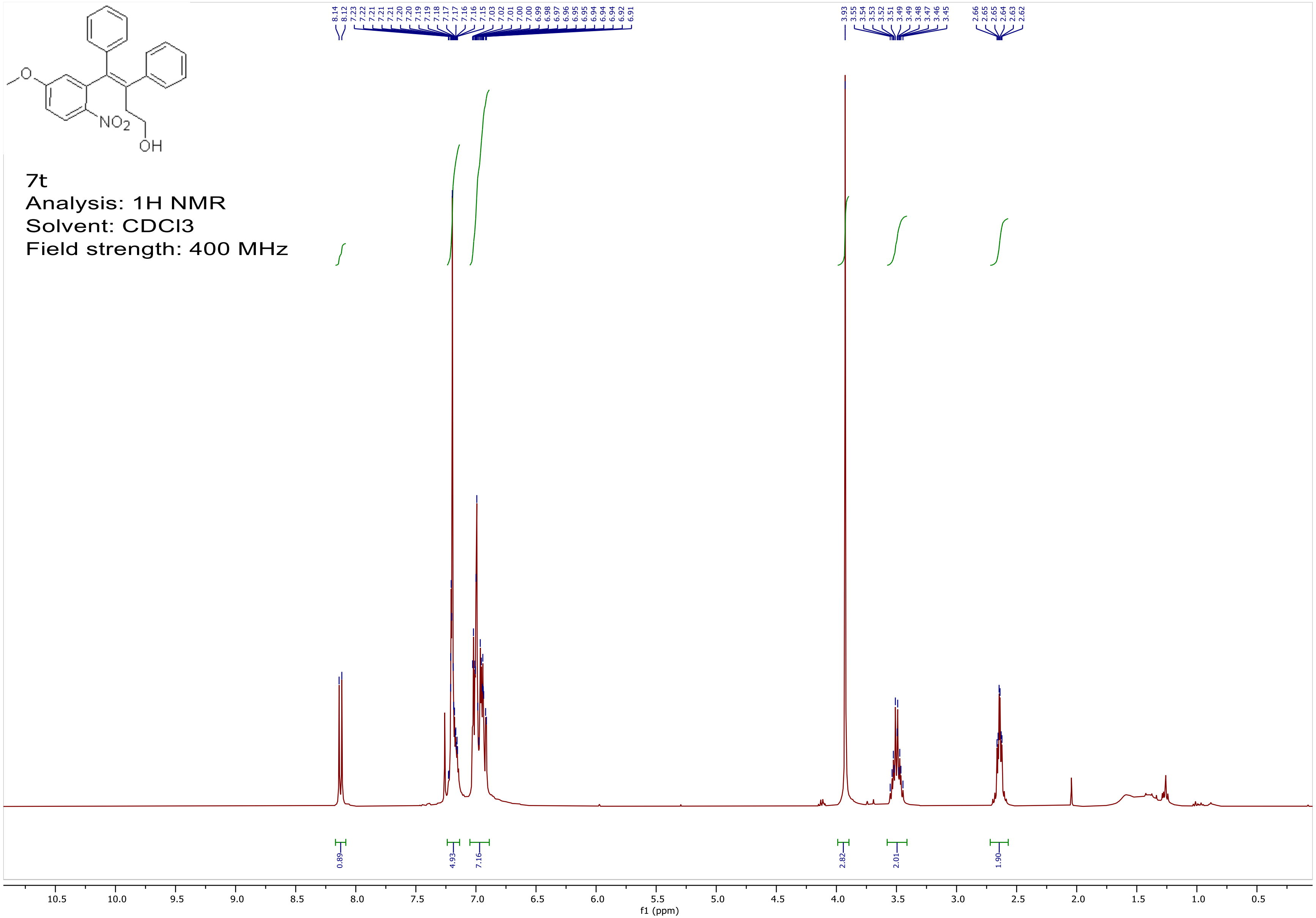

NO2
OH
7t
Analysis: 1H NMR
Solvent: CDCl3
Field strength: 400 MHz
f1 (ppm)
0.89
4.93
7.16
2.82
2.01
1.90

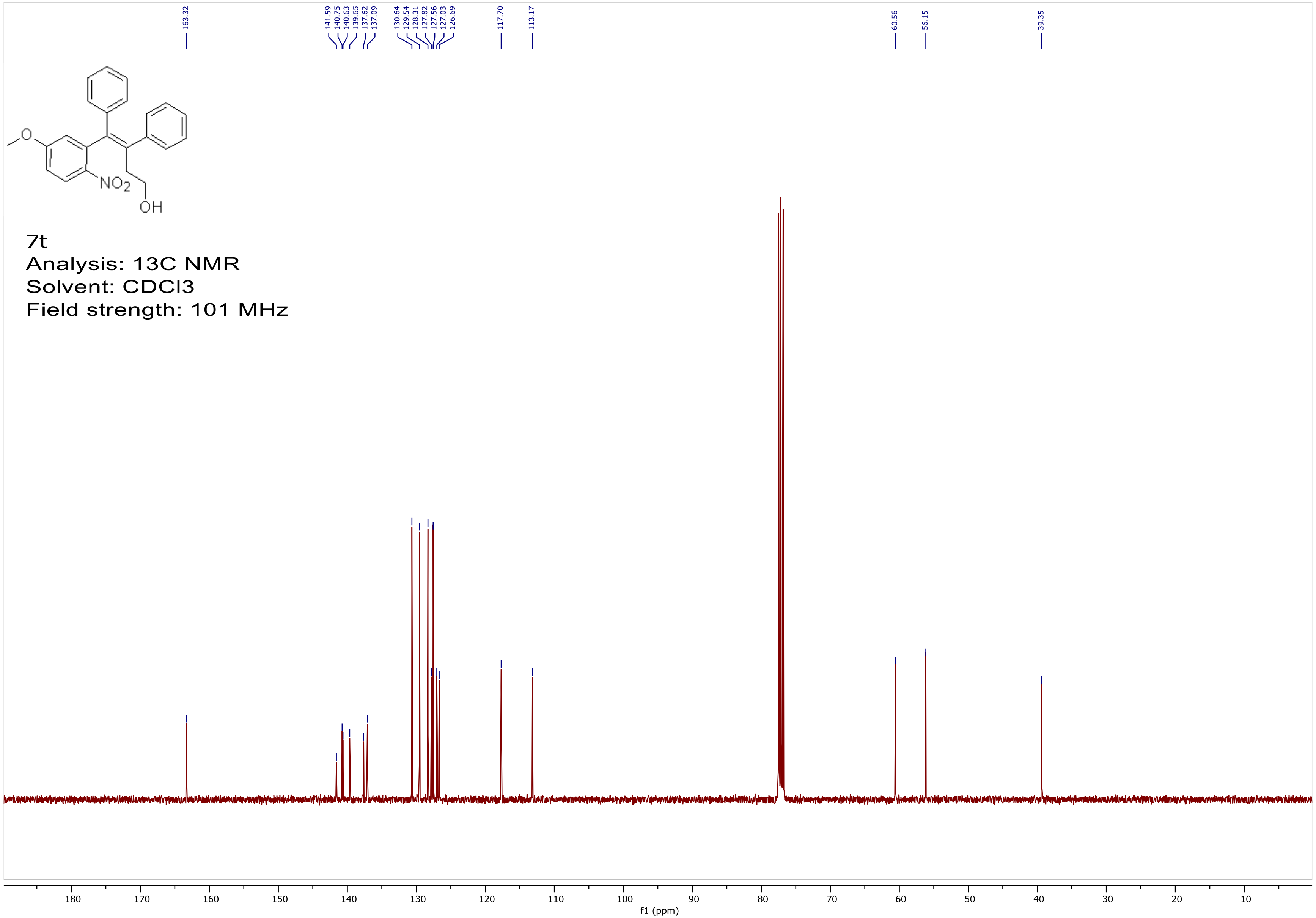
163.32
141.59
140.75
140.63
139.65
137.62
137.09
130.64
129.54
128.31
127.82
127.56
127.03
126.69
117.70
113.17
60.56
56.15
39.35
NO2
OH
O
7t
Analysis: 13C NMR
Solvent: CDCl3
Field strength: 101 MHz
180
170
160
150
140
130
120
110
100
90
80
70
60
50
40
30
20
10
f1 (ppm)

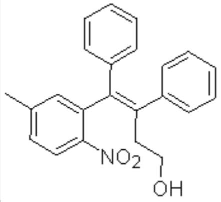

7u
Analysis: 1H NMR
Solvent: CDCl3
Field strength: 400 MHz

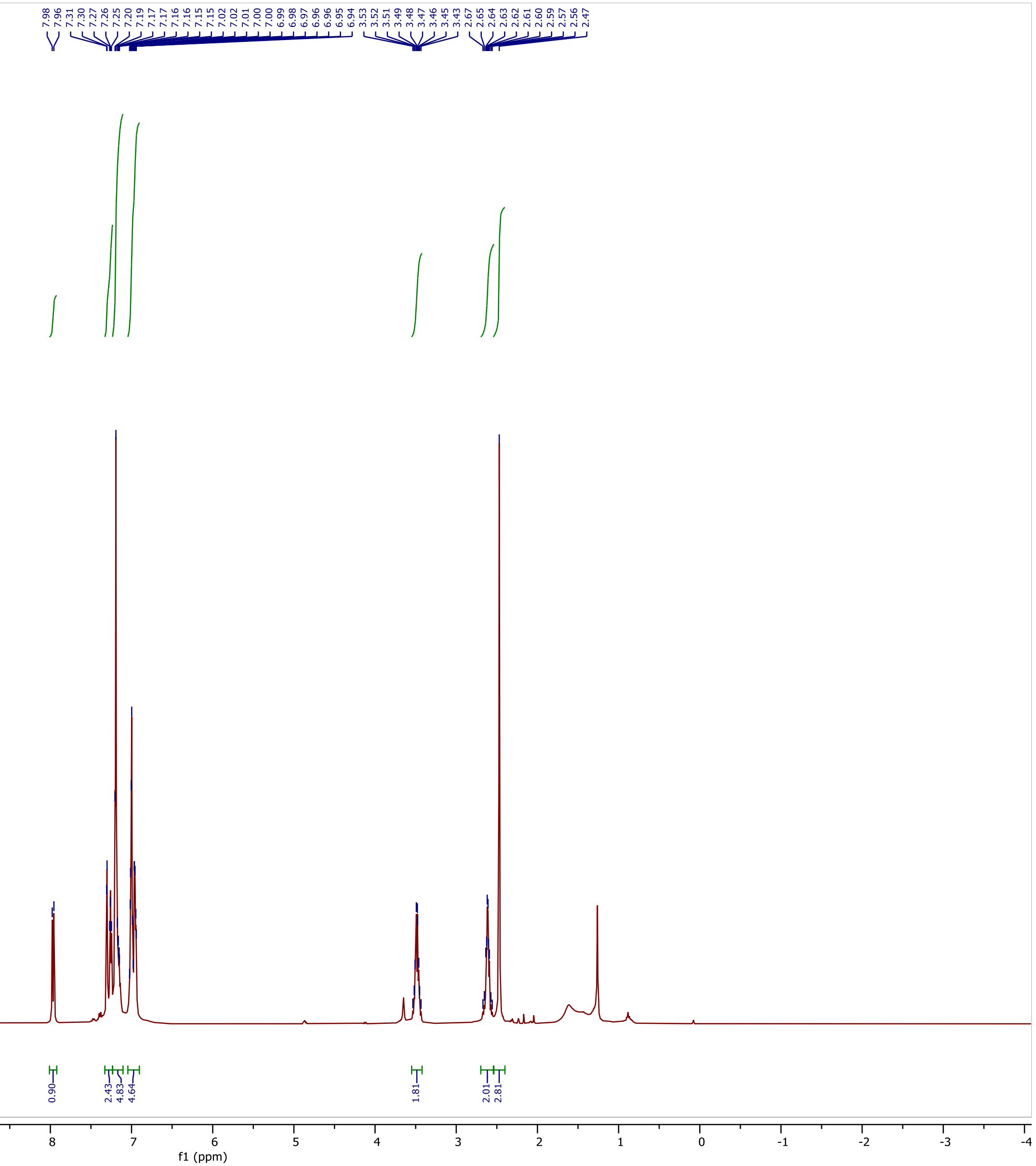

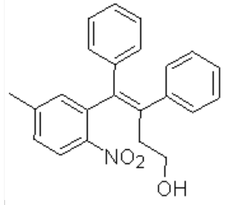

7u
Analysis: 13C NMR
Solvent: CDCl3
Field strength: 101 MHz

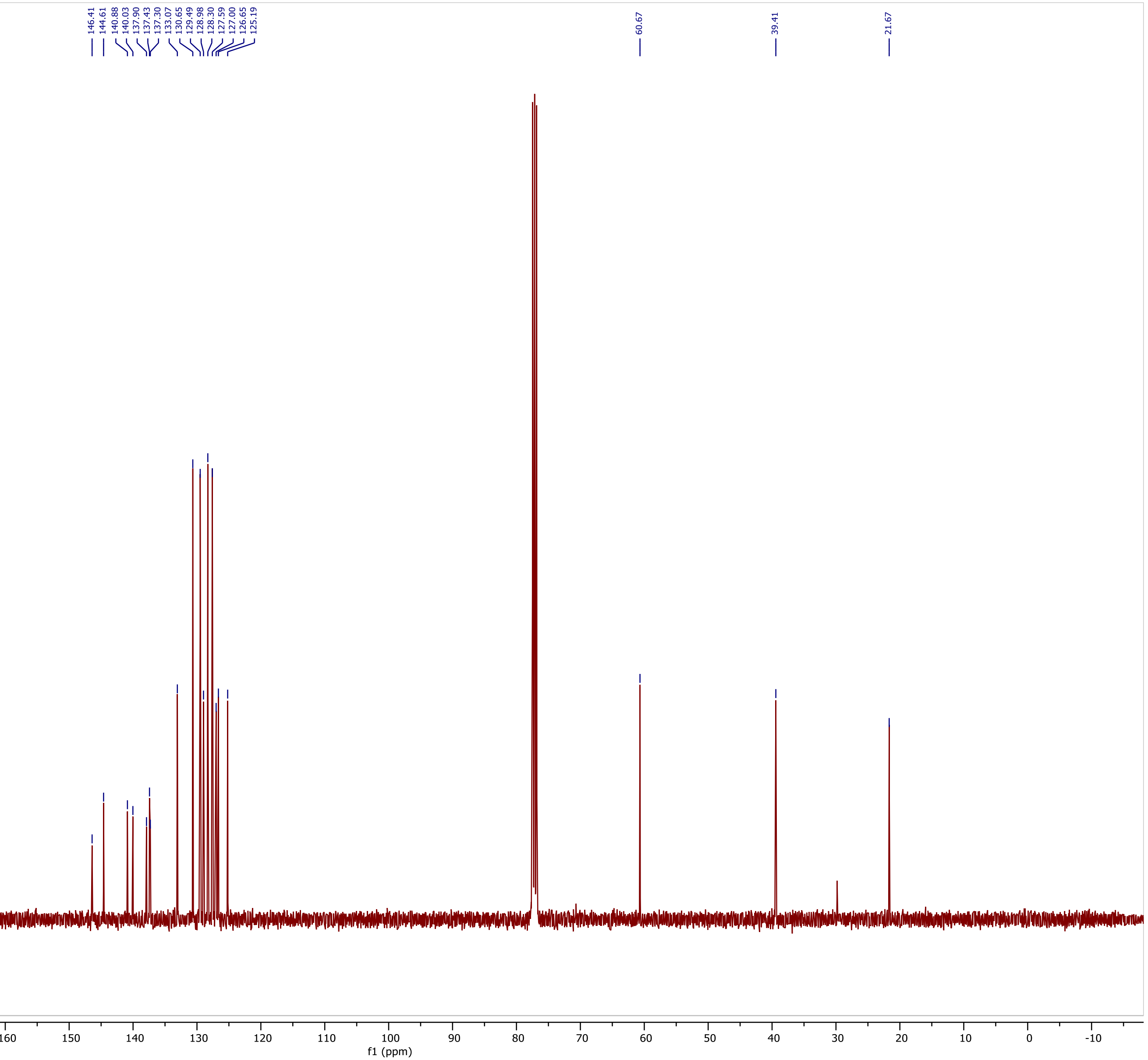

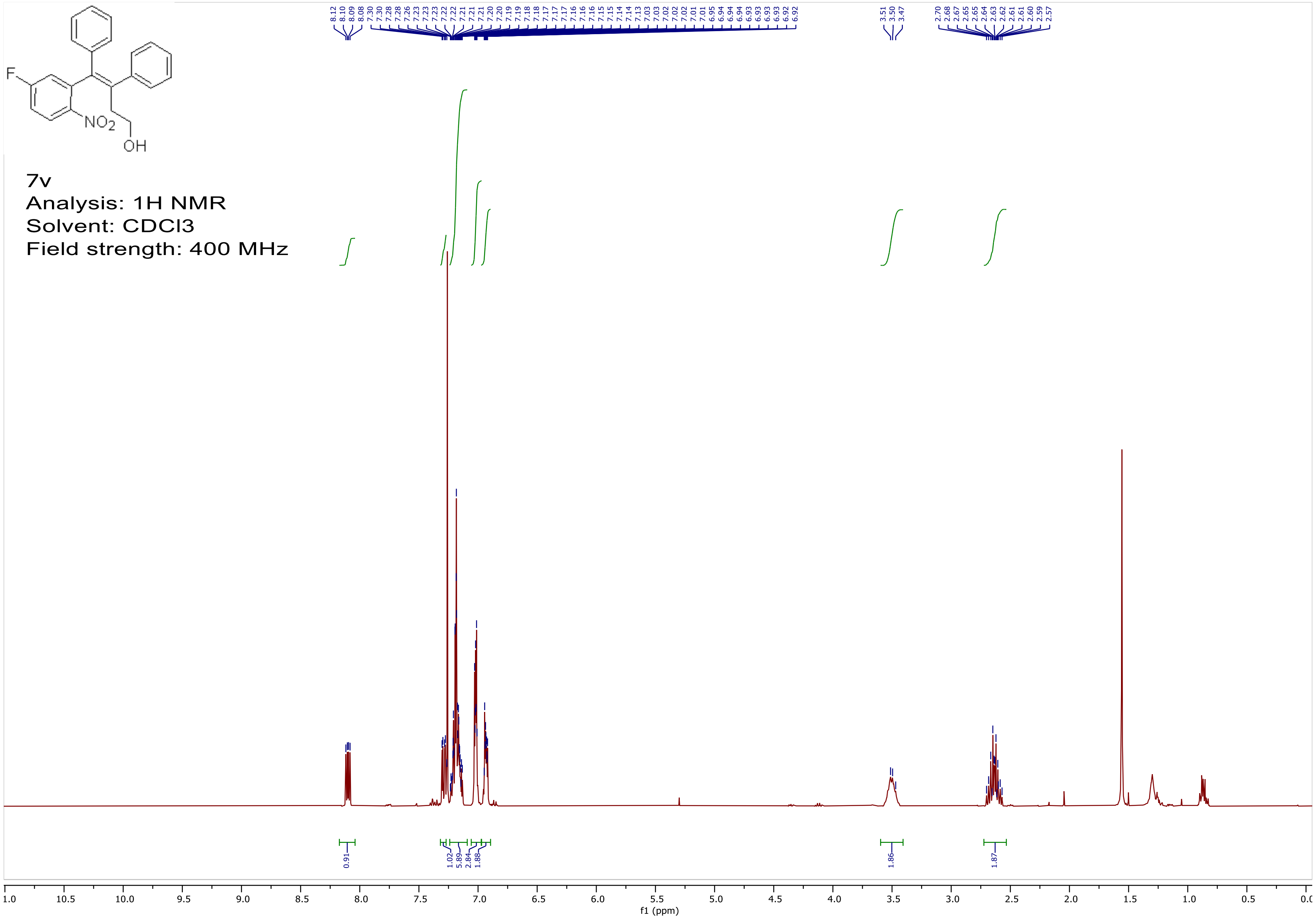
F
NO2
OH
7v
Analysis: 1H NMR
Solvent: CDCl3
Field strength: 400 MHz
8.12 8.10 8.09 8.08
7.30 7.30 7.28 7.28 7.26 7.23 7.23 7.22 7.22 7.21 7.21 7.21 7.20 7.20 7.19 7.19 7.18 7.18 7.17 7.17 7.17 7.16 7.16 7.15 7.15 7.14 7.14 7.13 7.03 7.02 7.02 7.02 7.01 7.01 6.95 6.94 6.94 6.94 6.93 6.93 6.93 6.92 6.92
3.51 3.50 3.47
2.70 2.68 2.67 2.65 2.65 2.64 2.63 2.62 2.61 2.61 2.60 2.59 2.57
0.91 1.02 5.89 2.84 1.88 1.86 1.87
1.0 10.5 10.0 9.5 9.0 8.5 8.0 7.5 7.0 6.5 6.0 5.5 5.0 4.5 4.0 3.5 3.0 2.5 2.0 1.5 1.0 0.5 0.0
f1 (ppm)

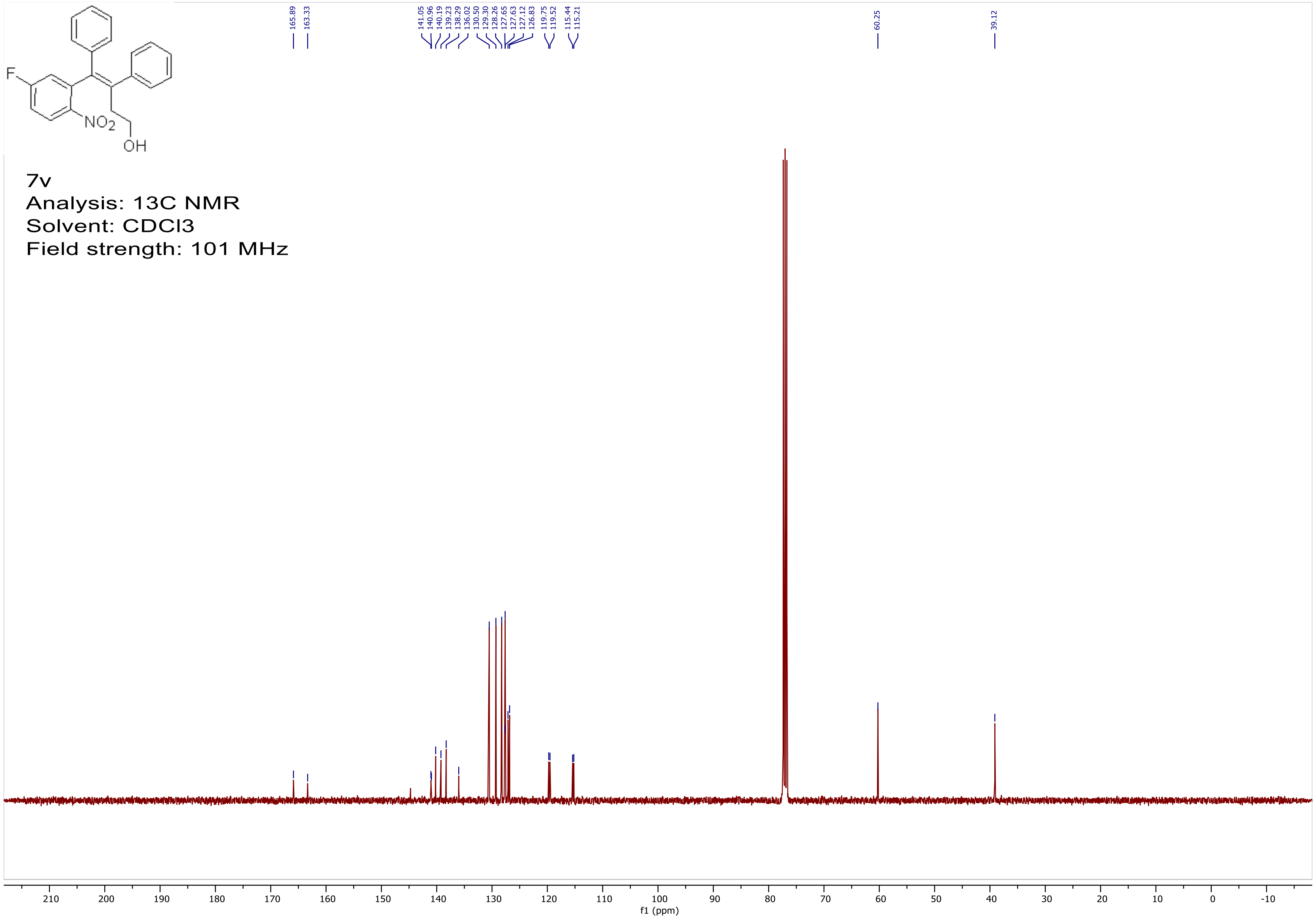
NO2
OH
F
7v
Analysis: 13C NMR
Solvent: CDCl3
Field strength: 101 MHz
165.89
163.33
141.05
140.96
140.19
139.23
138.29
136.02
130.50
129.30
128.26
127.65
127.63
127.12
126.83
119.75
119.52
115.44
115.21
60.25
39.12
210 200 190 180 170 160 150 140 130 120 110 100 90 80 70 60 50 40 30 20 10 0 -10
f1 (ppm)

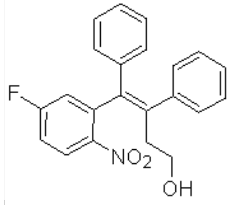

7v
Analysis: 19F NMR
Solvent: CDCl3
Field strength: 376 MHz

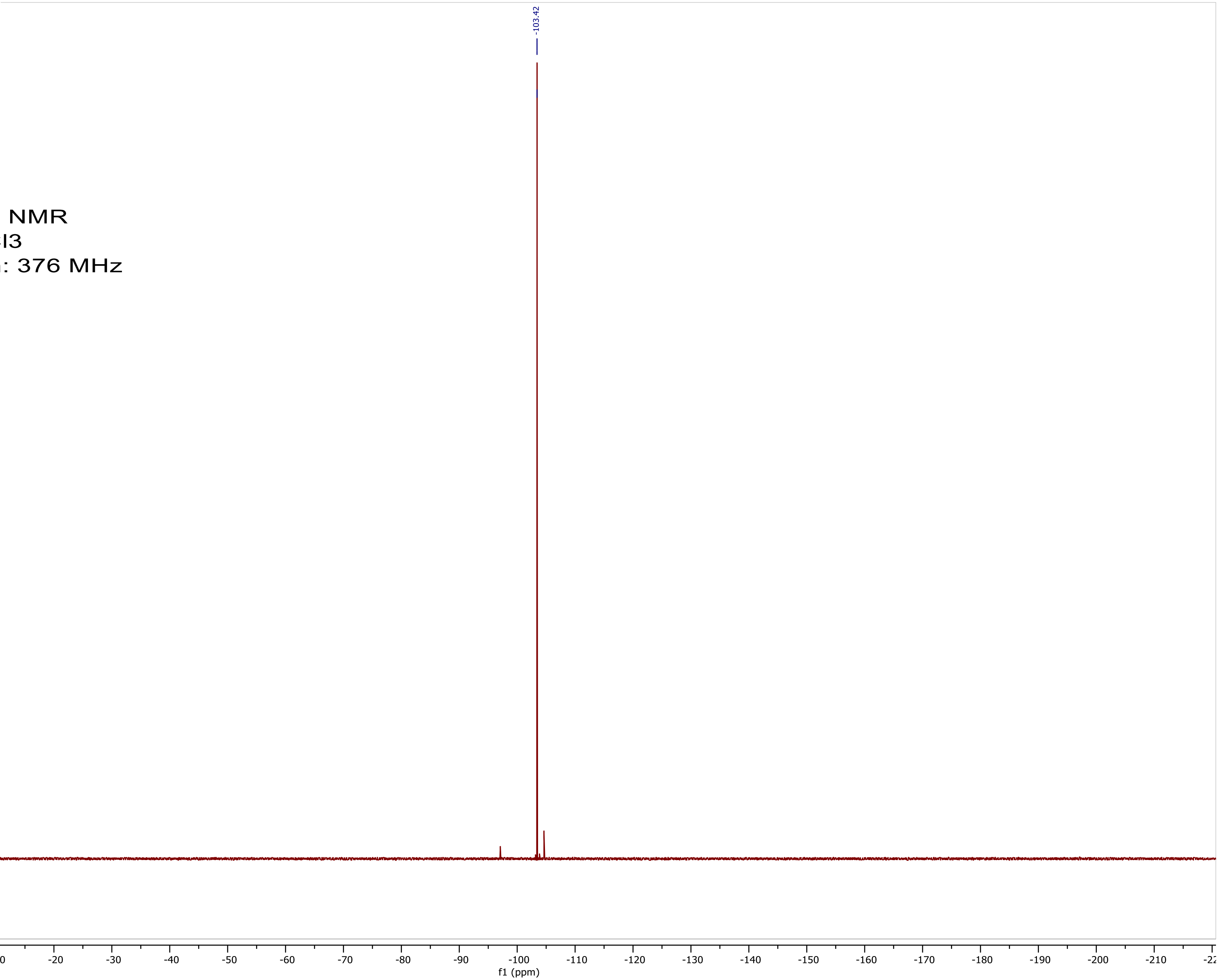

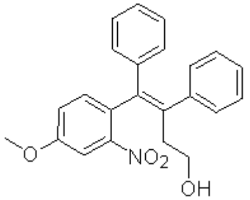

7w
Analysis: 1H NMR
Solvent: CDCl3
Field strength: 400 MHz

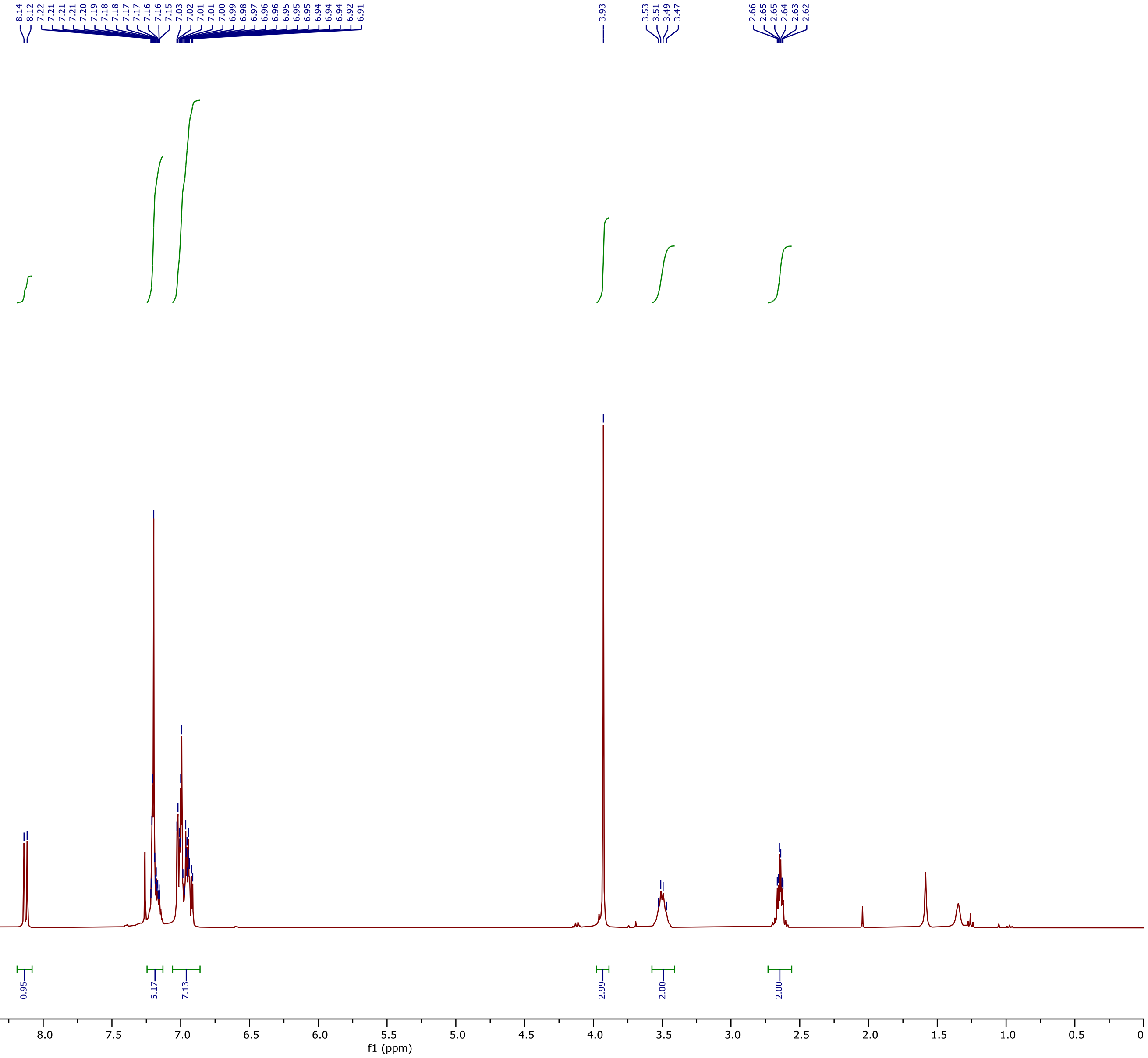

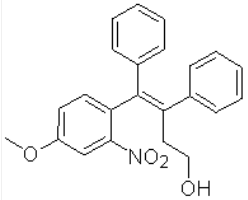

7w
Analysis: 13C NMR
Solvent: CDCl3
Field strength: 101 MHz

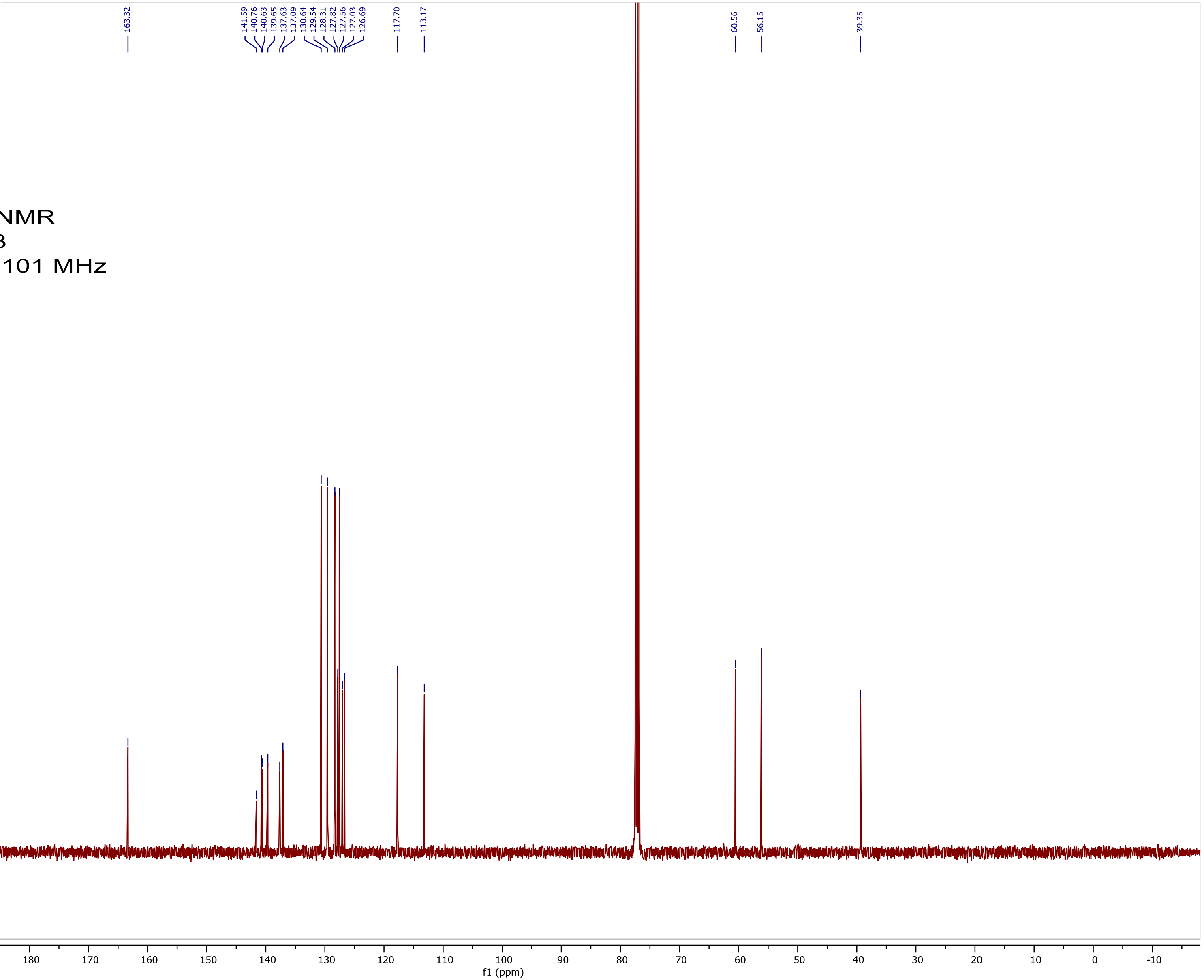

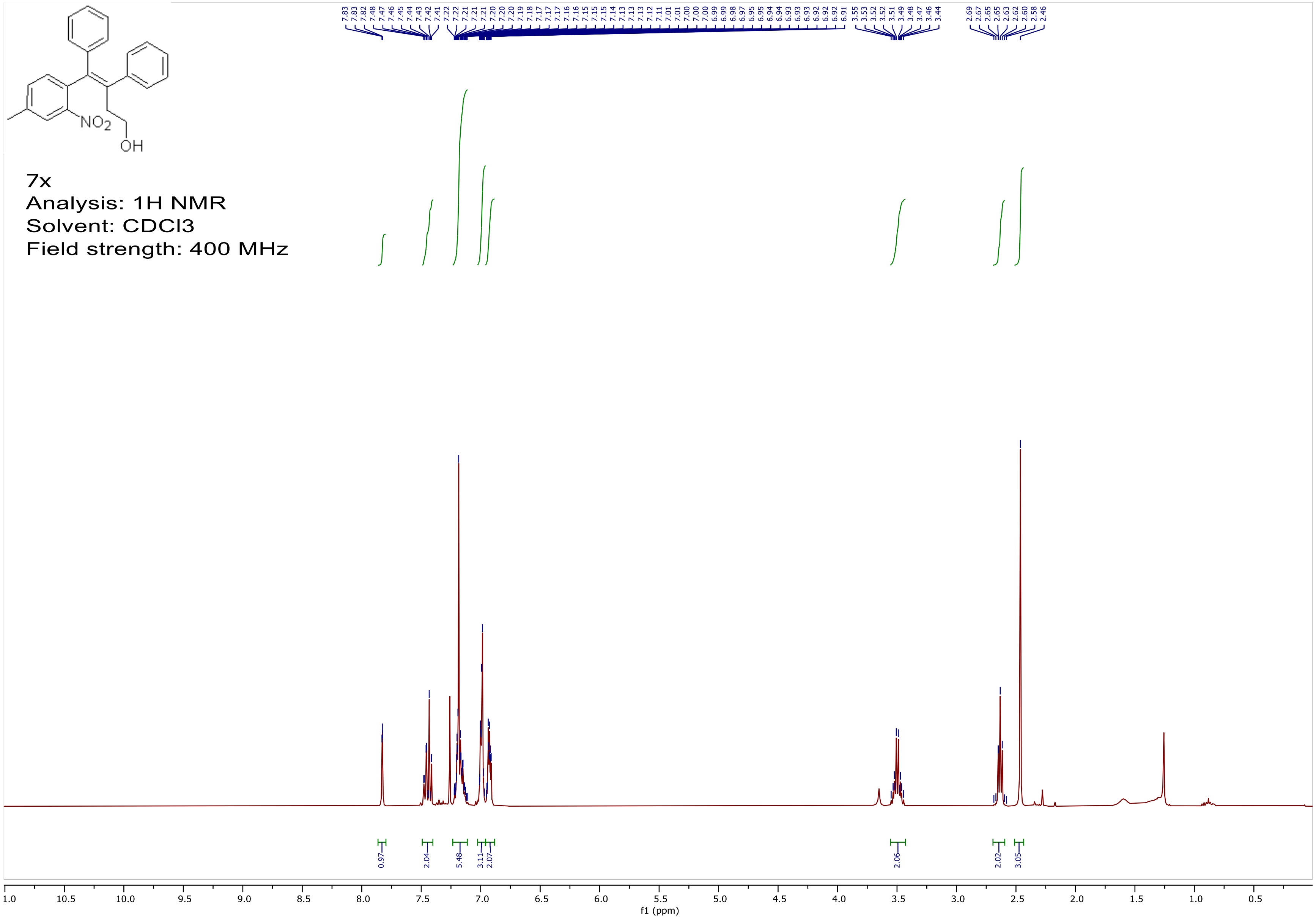
NO2
OH
7x
Analysis: 1H NMR
Solvent: CDCl3
Field strength: 400 MHz
f1 (ppm)

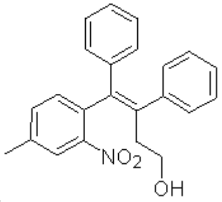

7x
Analysis: 13C NMR
Solvent: CDCl3
Field strength: 101 MHz

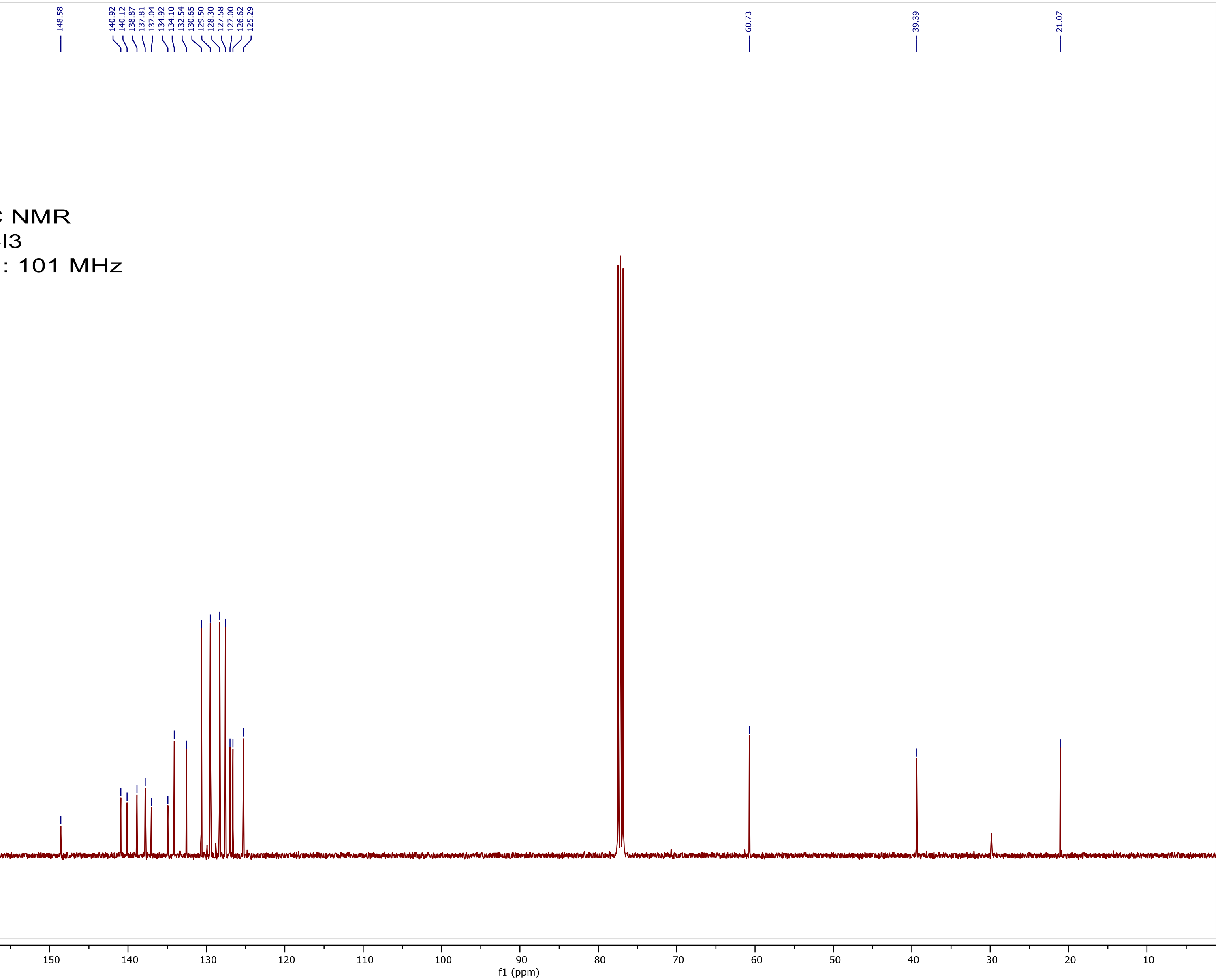

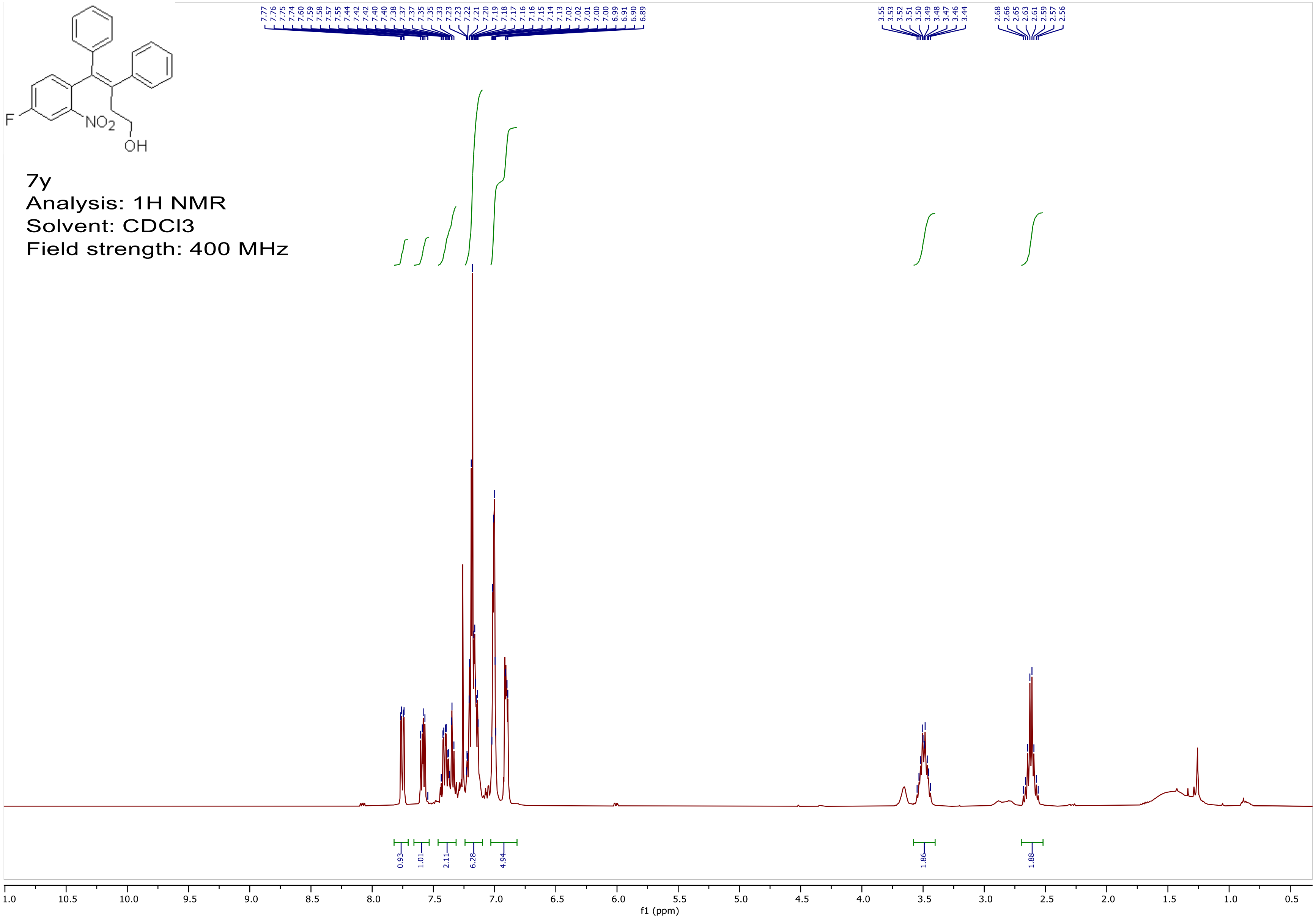
F
NO2
OH
7y
Analysis: 1H NMR
Solvent: CDCl3
Field strength: 400 MHz
7.77 7.76 7.75 7.74 7.60 7.59 7.58 7.57 7.55 7.44 7.42 7.42 7.40 7.40 7.38 7.37 7.37 7.35 7.35 7.33 7.23 7.23 7.22 7.21 7.20 7.19 7.18 7.17 7.16 7.16 7.15 7.14 7.13 7.02 7.02 7.01 7.00 7.00 6.99 6.91 6.90 6.89
3.55 3.53 3.52 3.51 3.50 3.49 3.48 3.47 3.46 3.44
2.68 2.66 2.65 2.63 2.61 2.59 2.57 2.56
0.93
1.01
2.11
6.28
4.94
1.86
1.88
f1 (ppm)

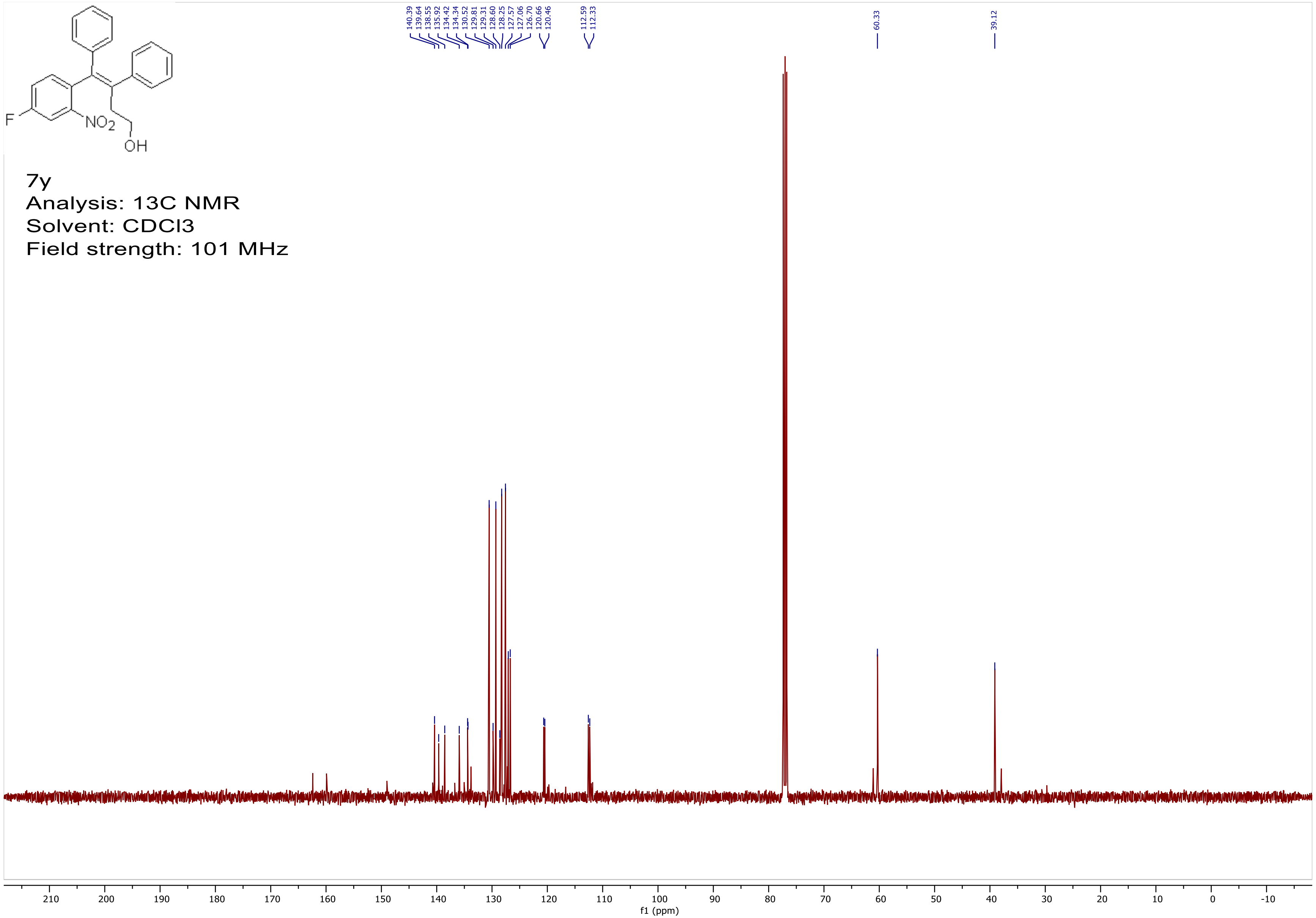
F
NO2
OH
7y
Analysis: 13C NMR
Solvent: CDCl3
Field strength: 101 MHz
140.39
139.64
138.55
135.92
134.42
134.34
130.52
129.81
129.31
128.60
128.25
127.57
127.06
126.70
120.66
120.46
112.59
112.33
60.33
39.12
f1 (ppm)

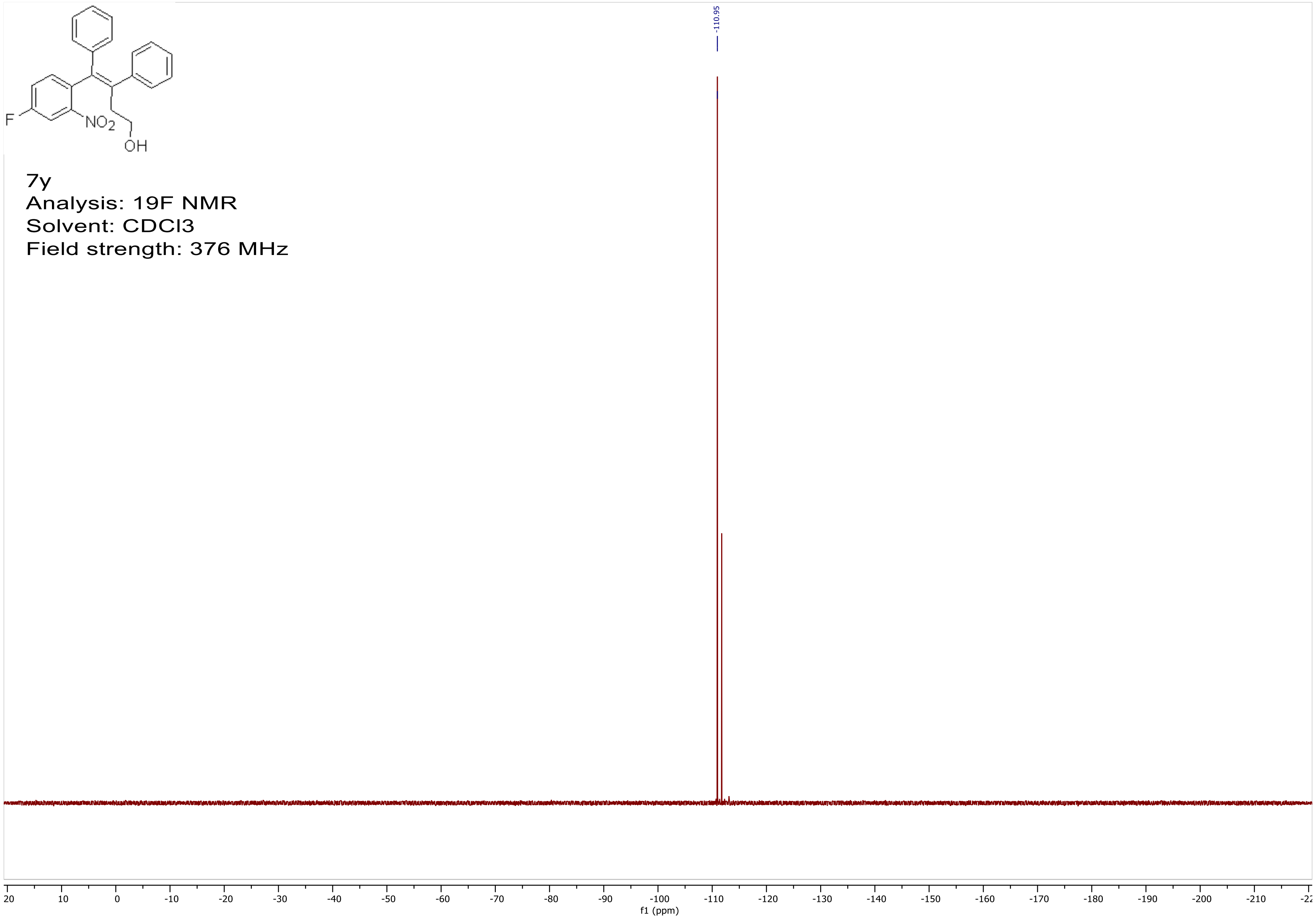

F
NO2
OH
7y
Analysis: 19F NMR
Solvent: CDCl3
Field strength: 376 MHz
-110.95
f1 (ppm)

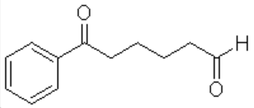

24
Analysis: 1H NMR
Solvent: CDCl3
Field strength: 400 MHz

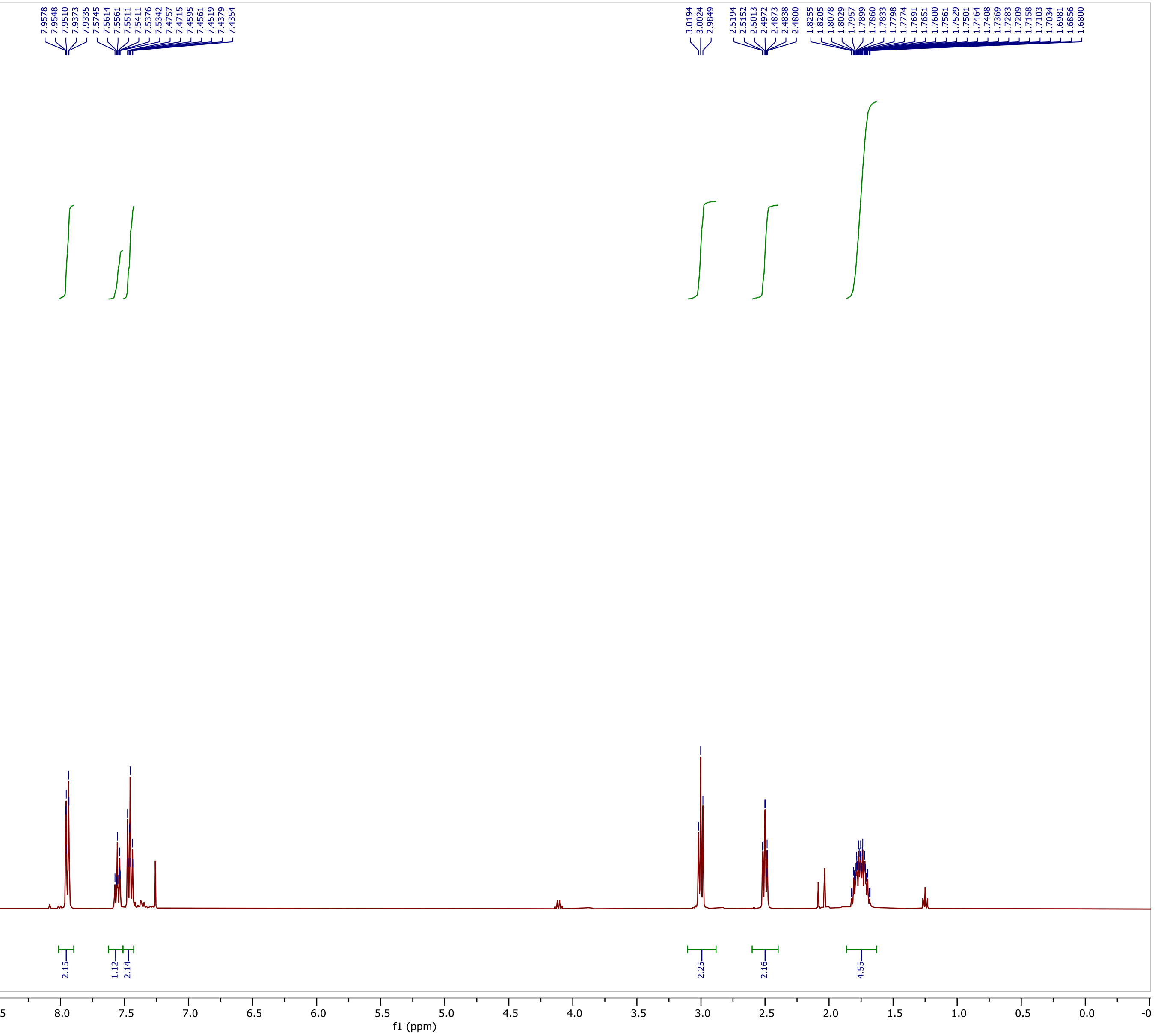

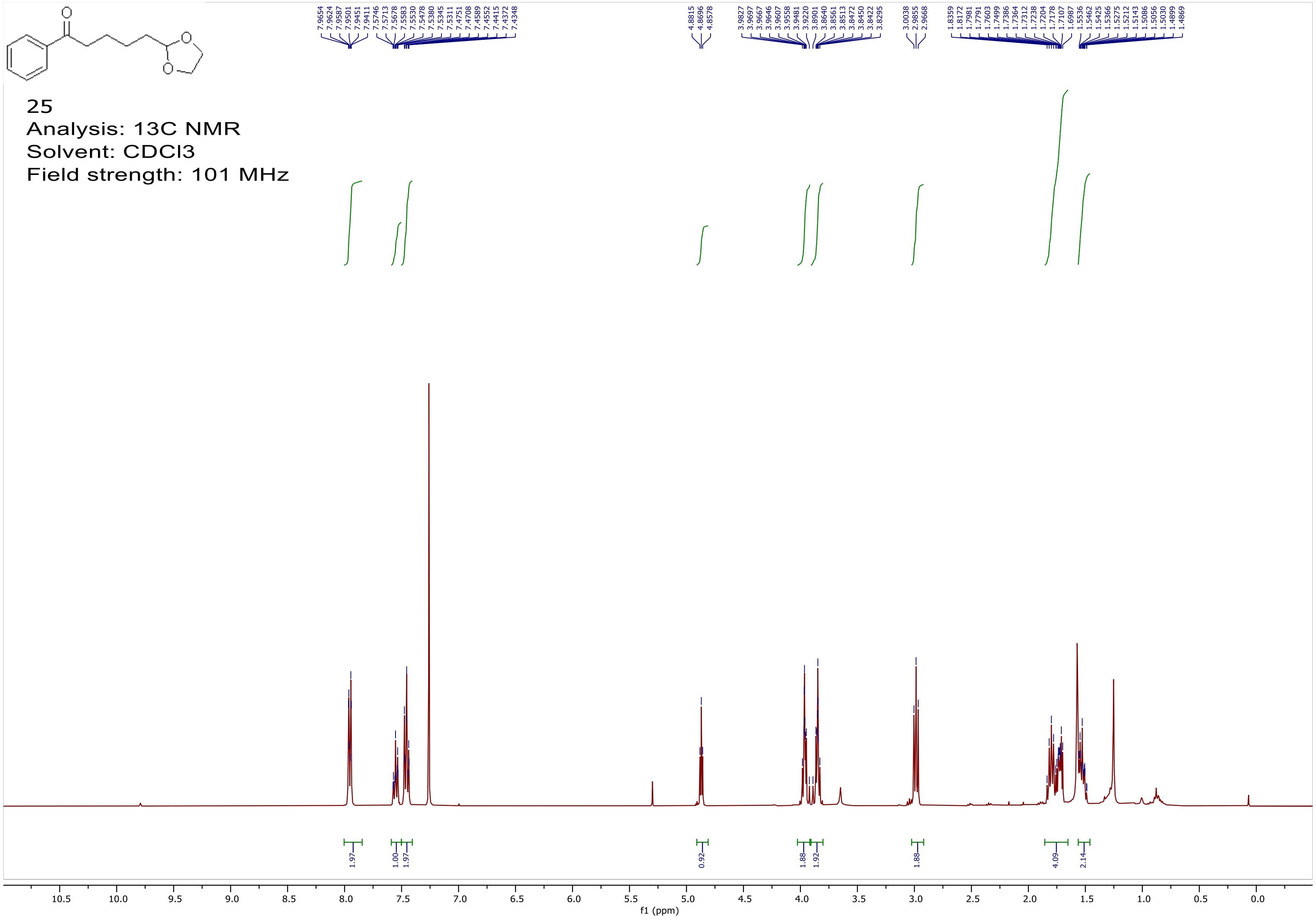
25
Analysis: 13C NMR
Solvent: CDCl3
Field strength: 101 MHz
f1 (ppm)

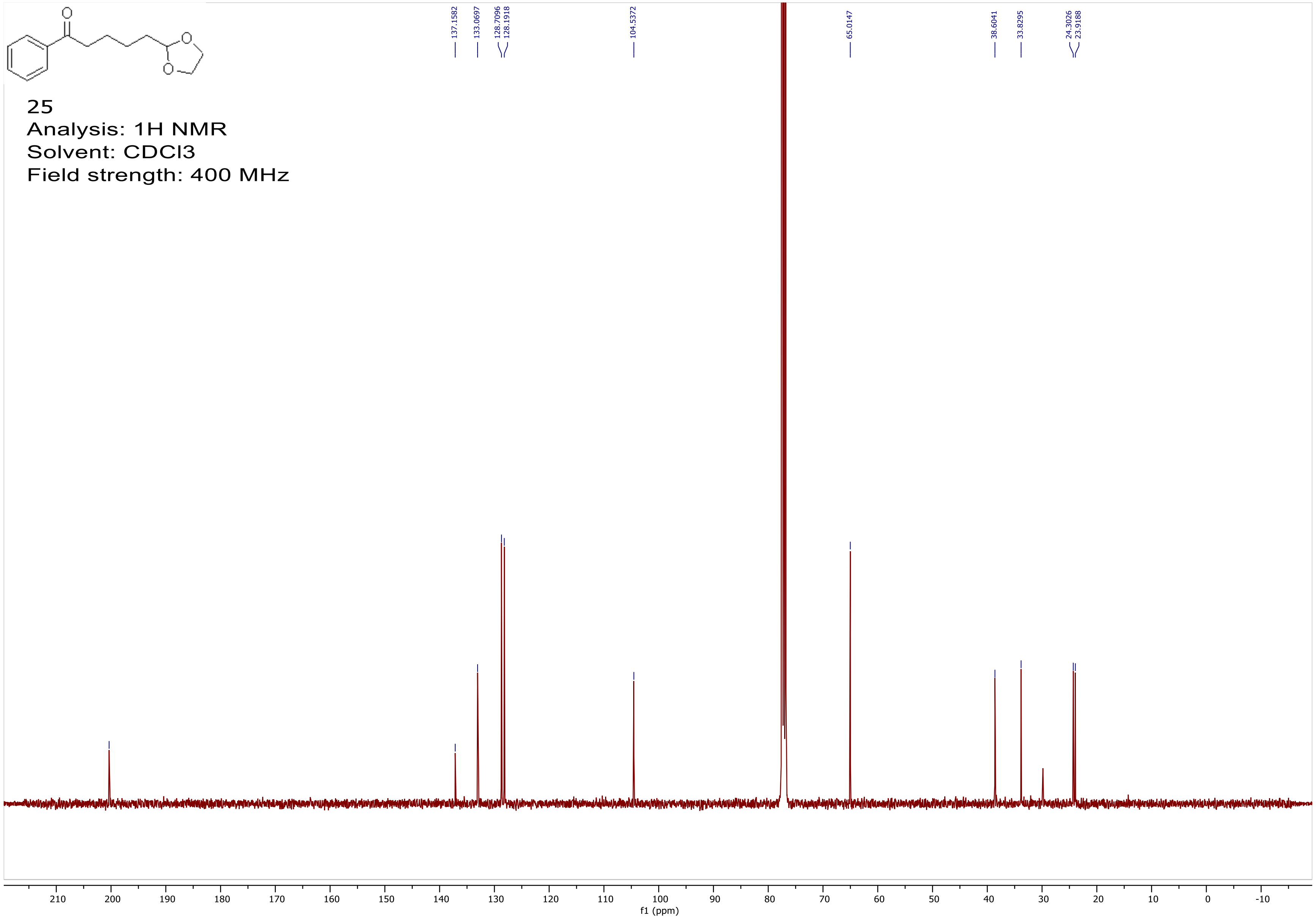

25
Analysis: 1H NMR
Solvent: CDCl3
Field strength: 400 MHz
137.1582
133.0697
128.7096
128.1918
104.5372
65.0147
38.6041
33.8295
24.3026
23.9188
210 200 190 180 170 160 150 140 130 120 110 100 90 80 70 60 50 40 30 20 10 0 -10
f1 (ppm)

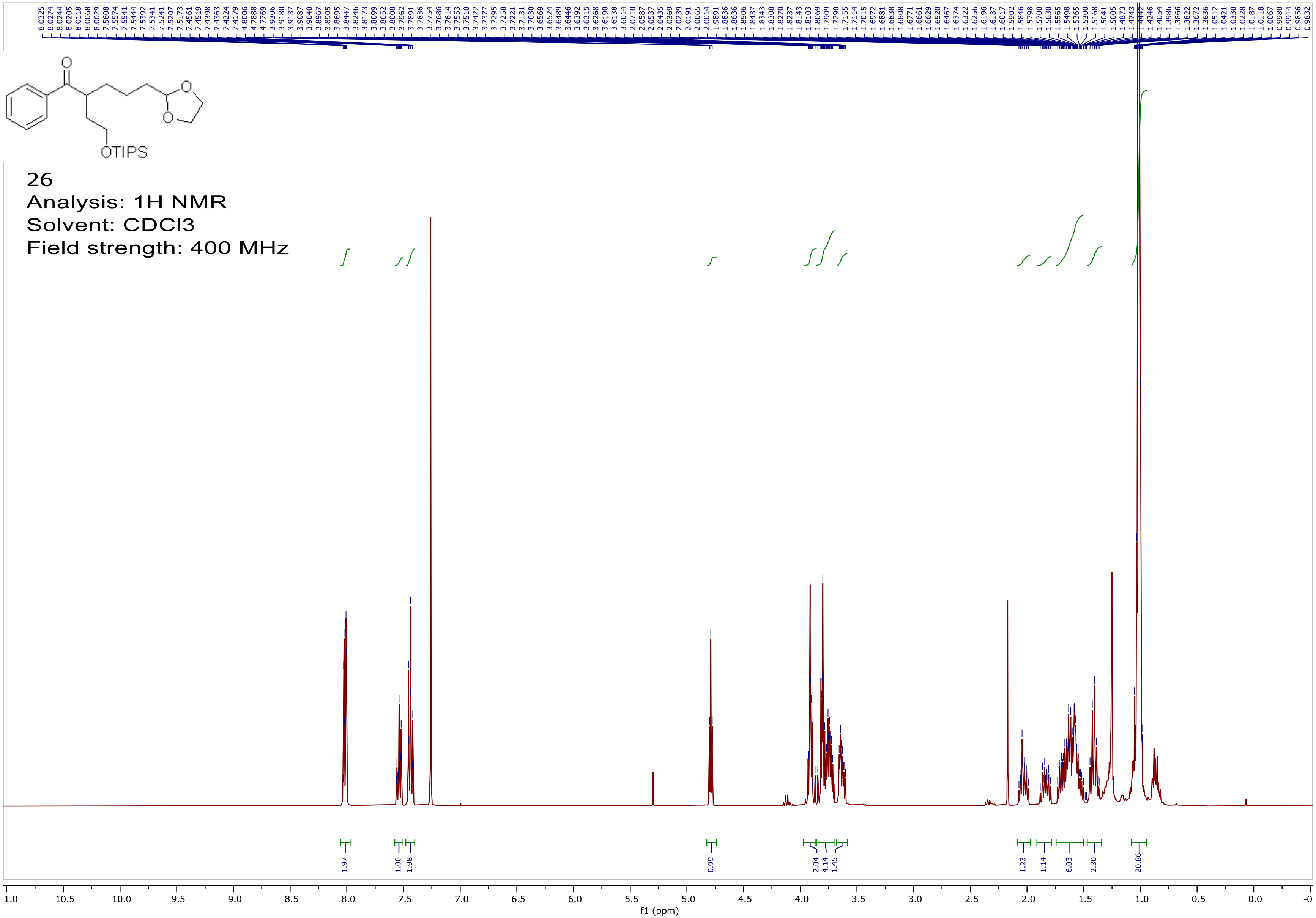
OTIPS
26
Analysis: 1H NMR
Solvent: CDCl3
Field strength: 400 MHz
f1 (ppm)

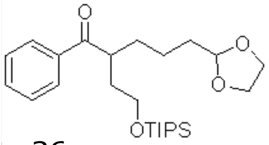

26
Analysis: 13C NMR
Solvent: CDCl3
Field strength: 101 MHz

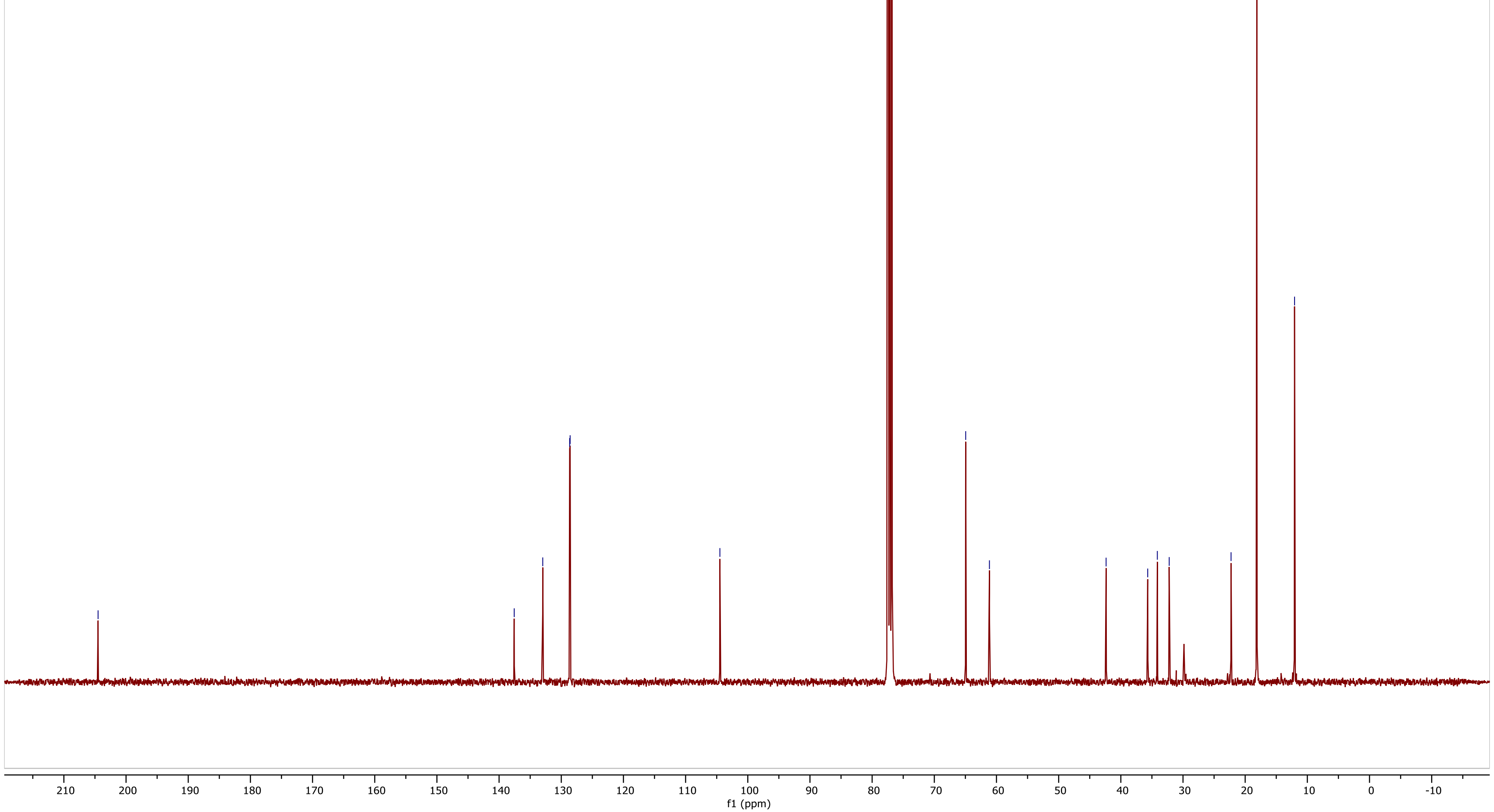

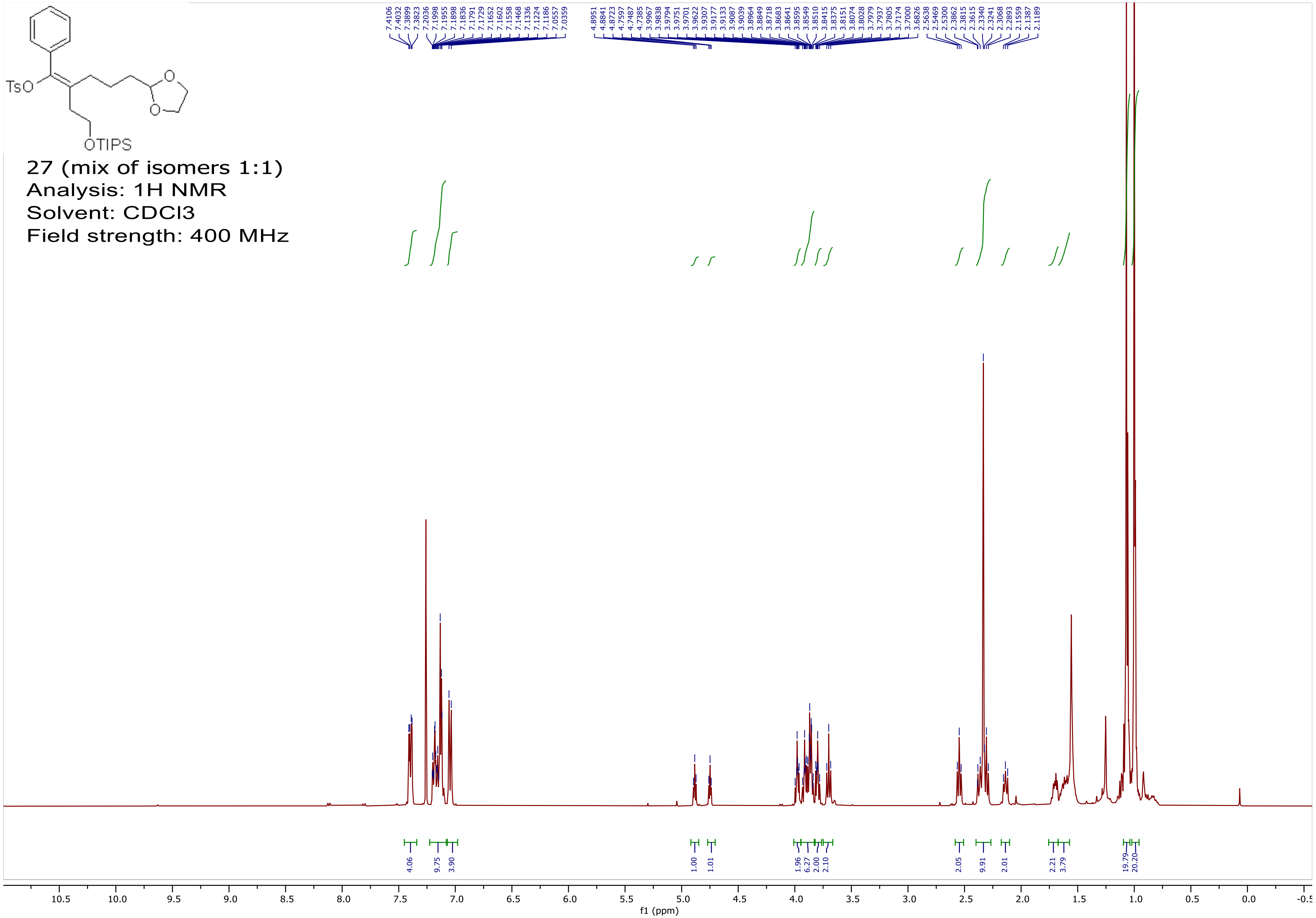
TsO
OTIPS
27 (mix of isomers 1:1)
Analysis: 1H NMR
Solvent: CDCl3
Field strength: 400 MHz
f1 (ppm)

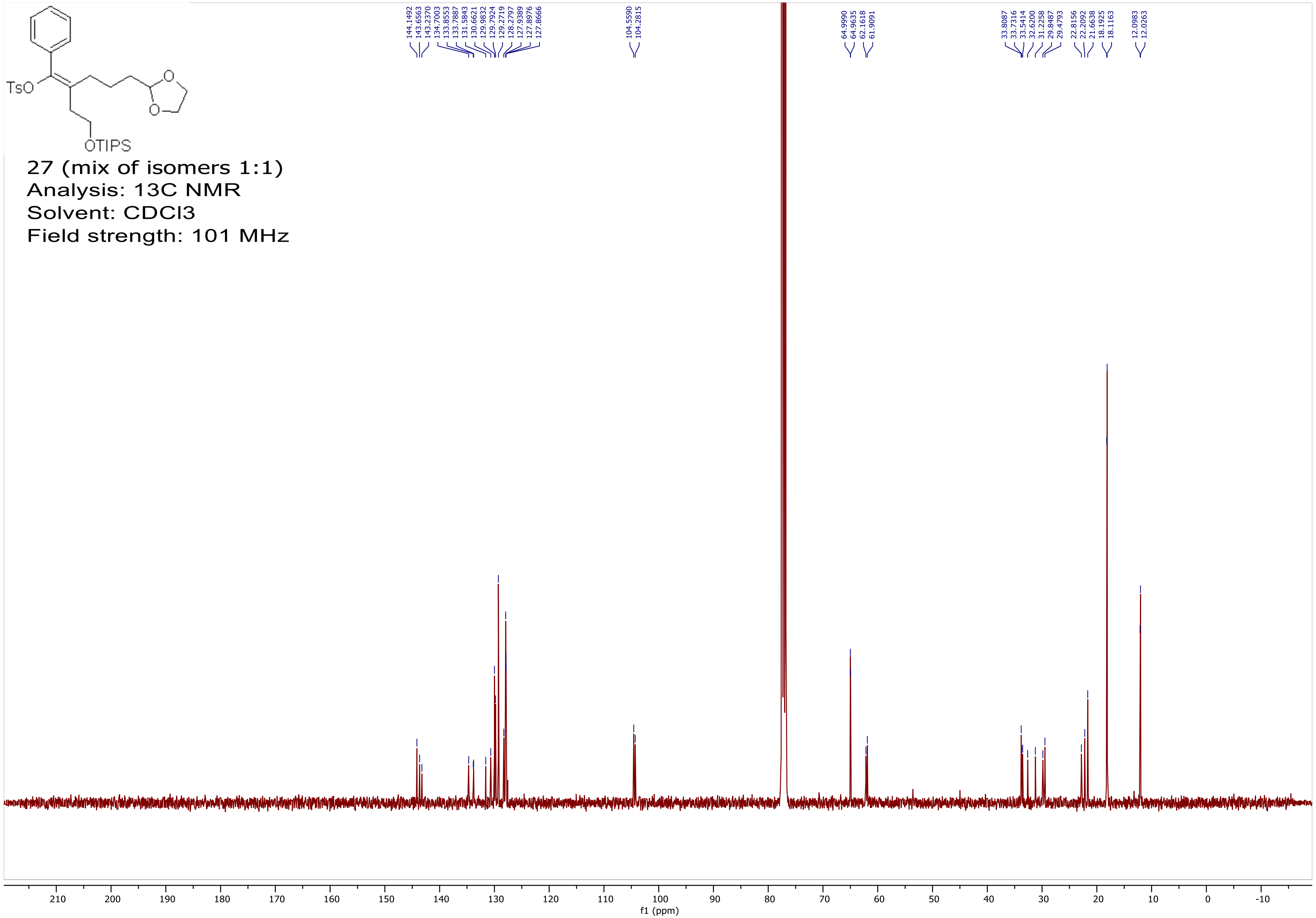
TsO
OTIPS
O
O
27 (mix of isomers 1:1)
Analysis: 13C NMR
Solvent: CDCl3
Field strength: 101 MHz
144.1492
143.6563
143.2370
134.7003
133.8553
133.7887
131.5843
130.6621
129.9832
129.7924
129.2719
128.2797
127.9389
127.8976
127.8666
104.5590
104.2815
64.9990
64.9635
62.1618
61.9091
33.8087
33.7316
33.5414
32.6200
31.2258
29.8487
29.4793
22.8156
22.2092
21.6638
18.1925
18.1163
12.0983
12.0263
210 200 190 180 170 160 150 140 130 120 110 100 90 80 70 60 50 40 30 20 10 0 -10
f1 (ppm)

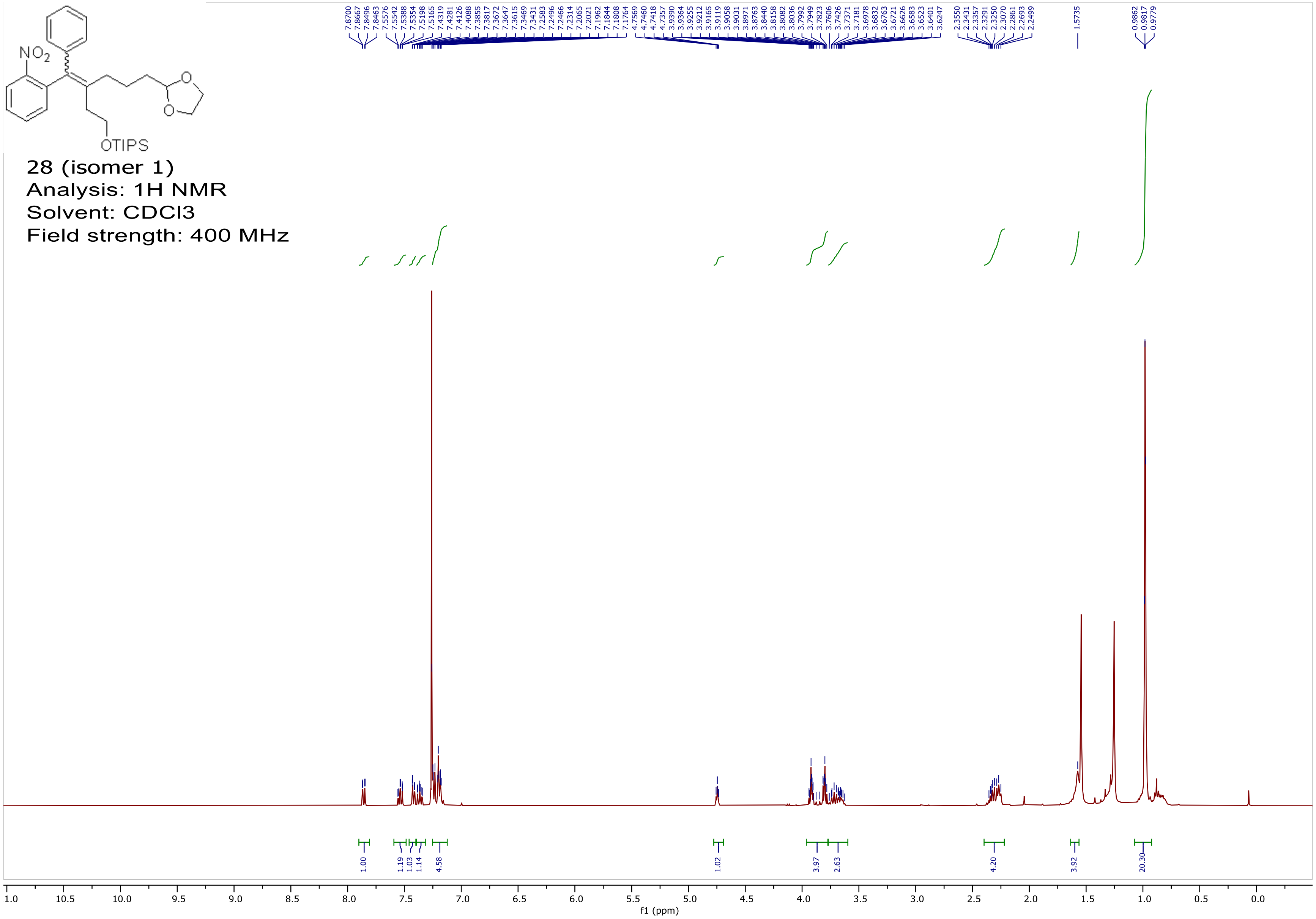

NO2
OTIPS
28 (isomer 1)
Analysis: 1H NMR
Solvent: CDCl3
Field strength: 400 MHz
1.00
1.19
1.03
1.14
4.58
1.02
3.97
2.63
4.20
3.92
20.30
f1 (ppm)

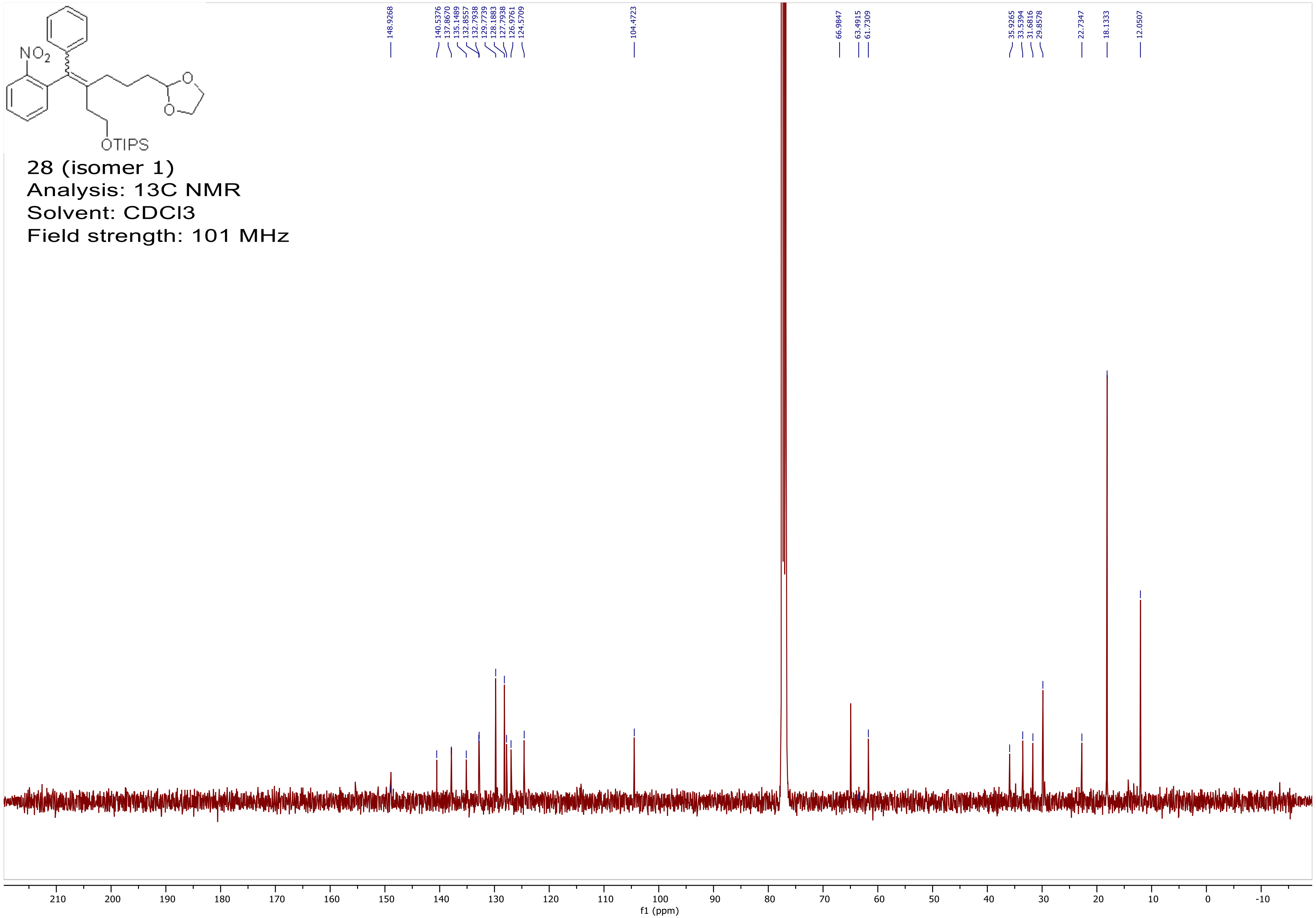

28 (isomer 1)
Analysis: 13C NMR
Solvent: CDCl3
Field strength: 101 MHz

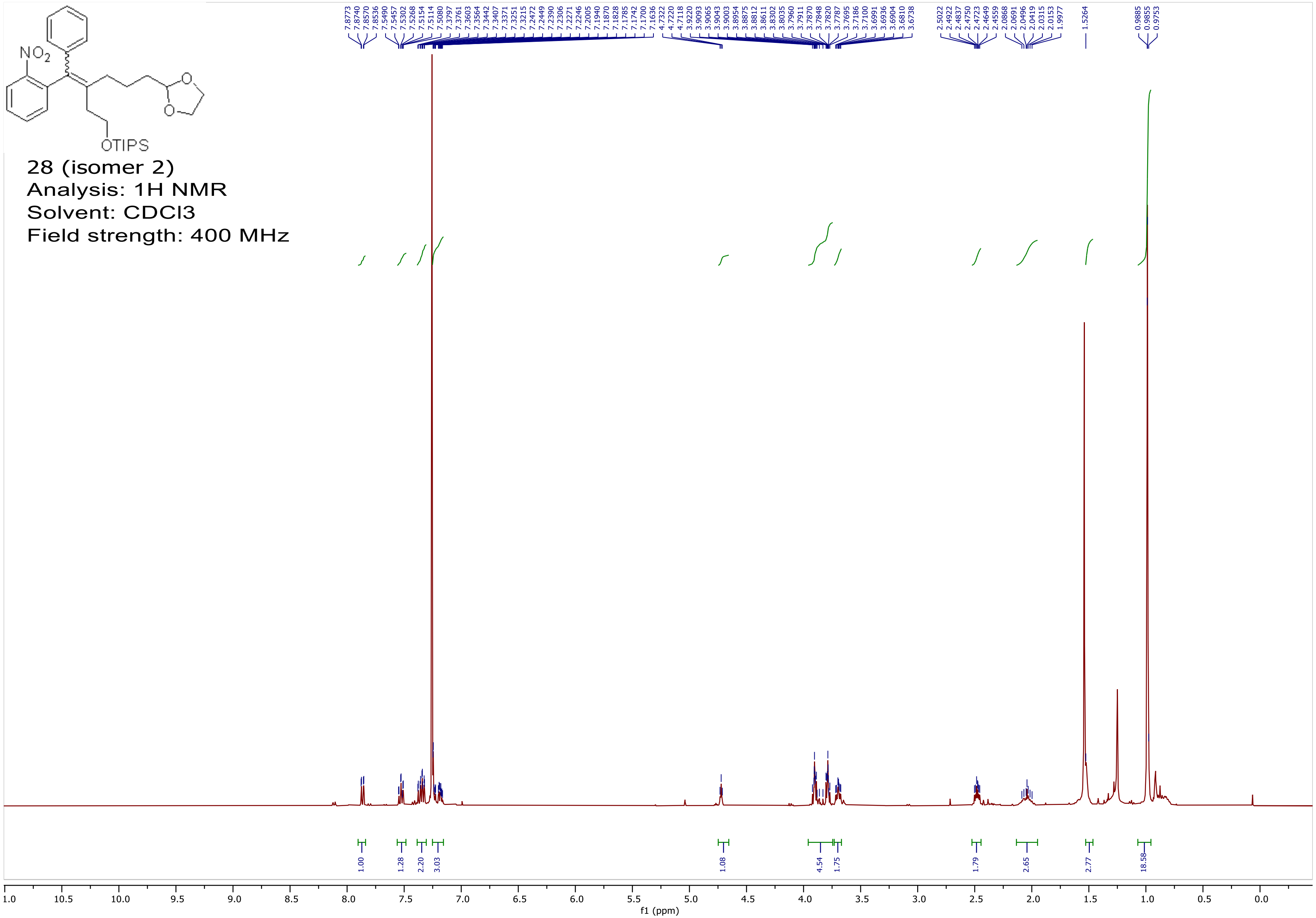

28 (isomer 2)
Analysis: 1H NMR
Solvent: CDCl3
Field strength: 400 MHz

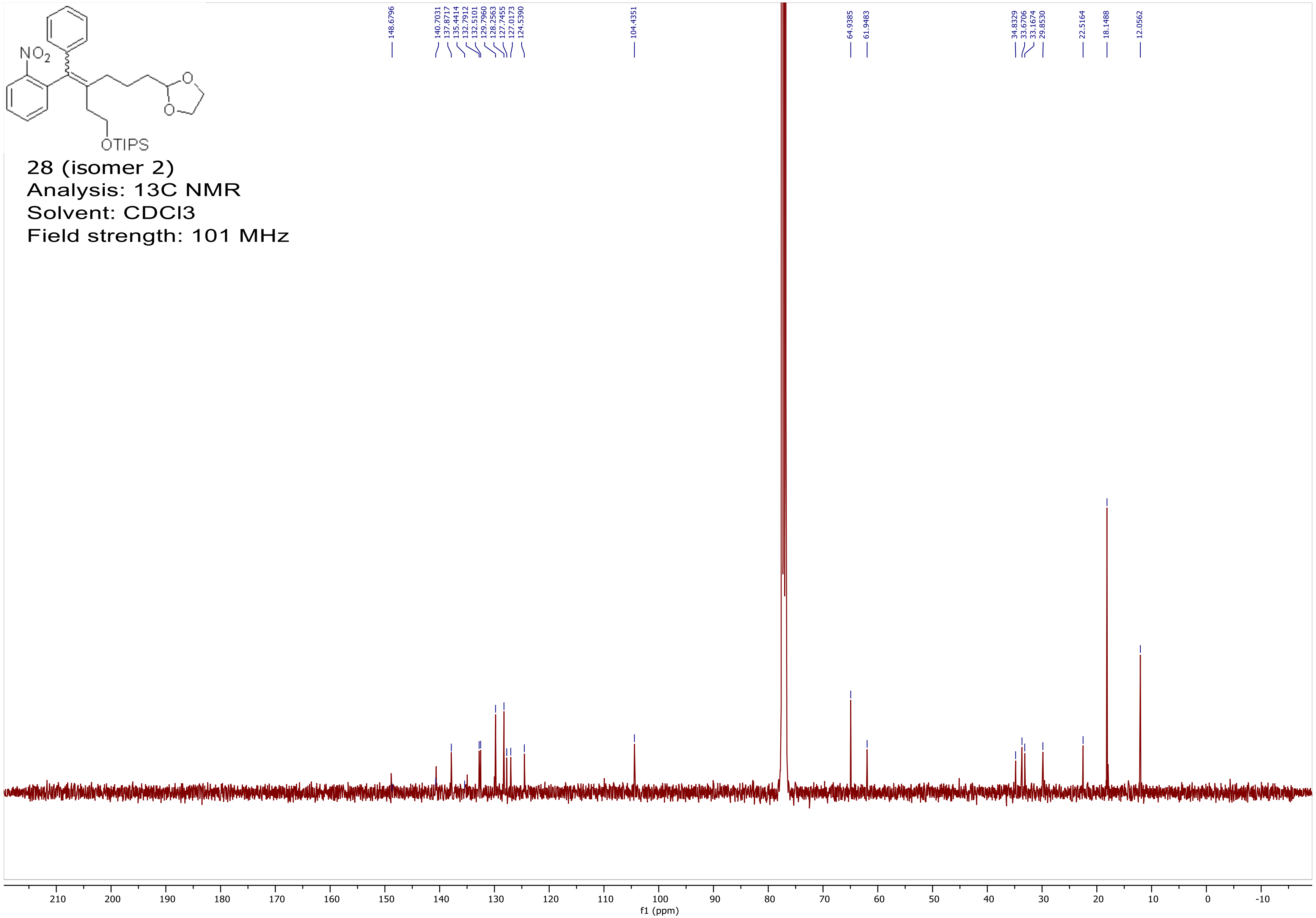

28 (isomer 2)
Analysis: 13C NMR
Solvent: CDCl3
Field strength: 101 MHz

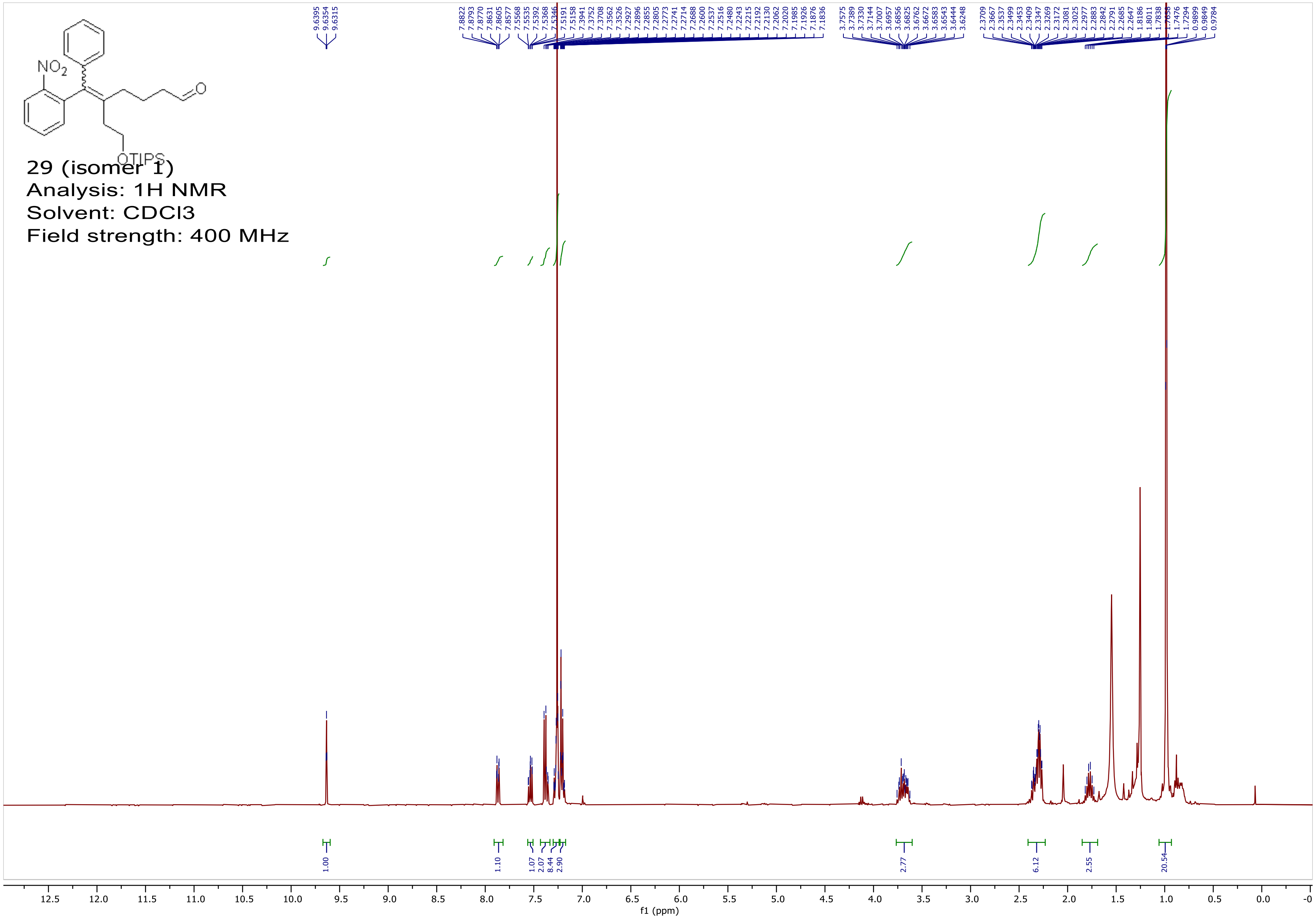
NO2
OTIPS
29 (isomer 1)
Analysis: 1H NMR
Solvent: CDCl3
Field strength: 400 MHz
f1 (ppm)

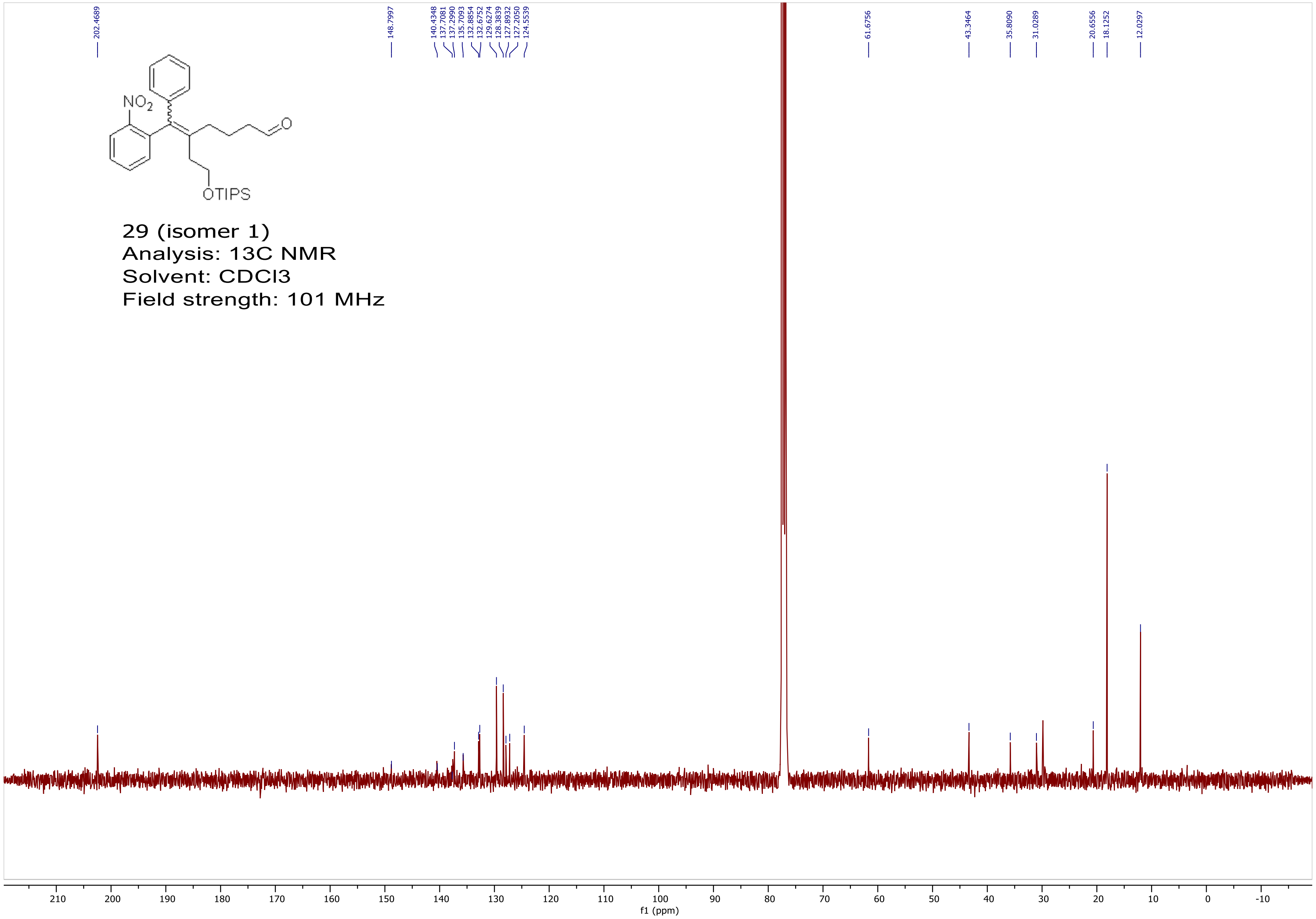

29 (isomer 1)
Analysis: 13C NMR
Solvent: CDCl3
Field strength: 101 MHz

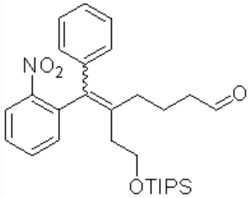

29 (isomer 2)
Analysis: 1H NMR
Solvent: CDCl3
Field strength: 400 MHz

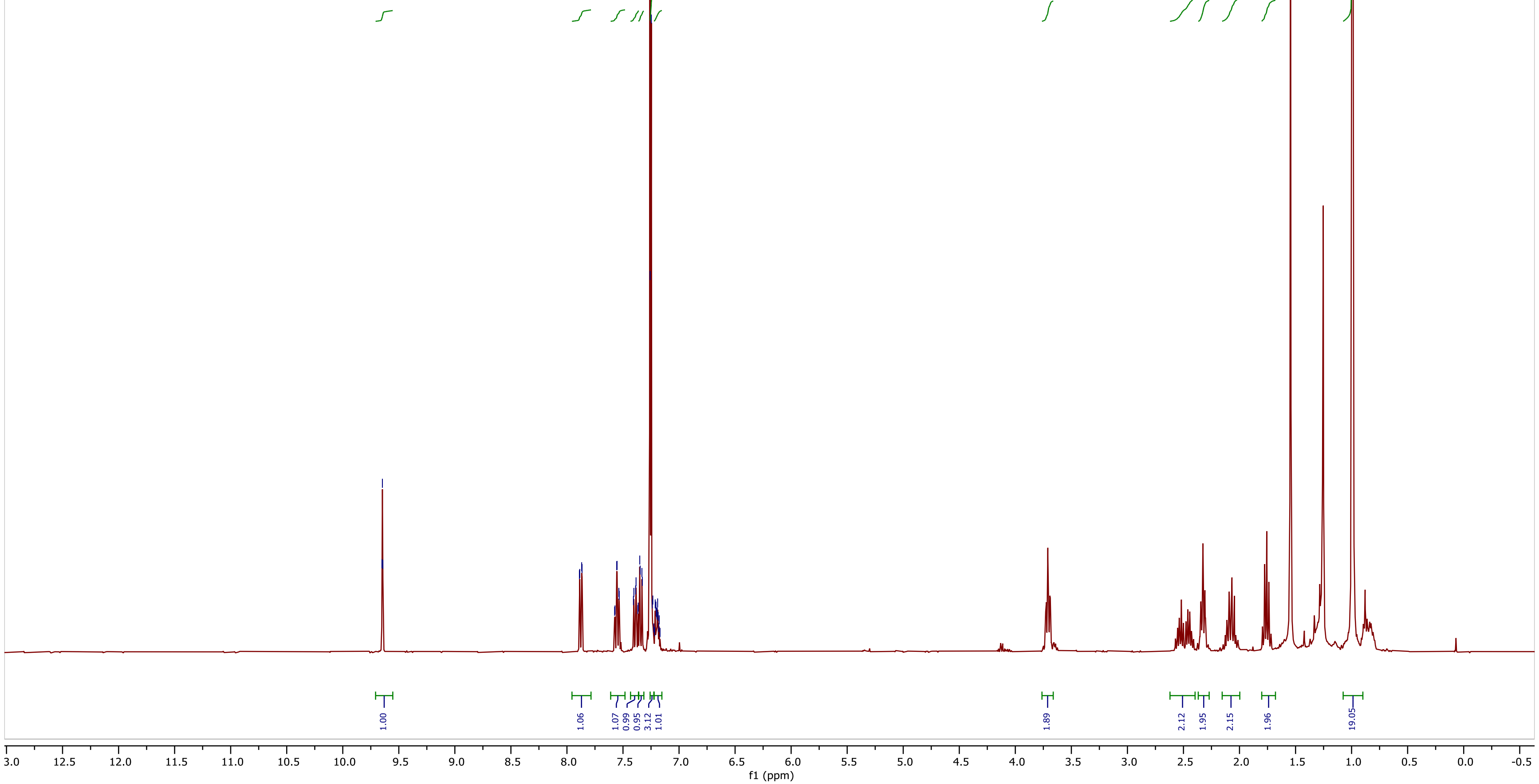

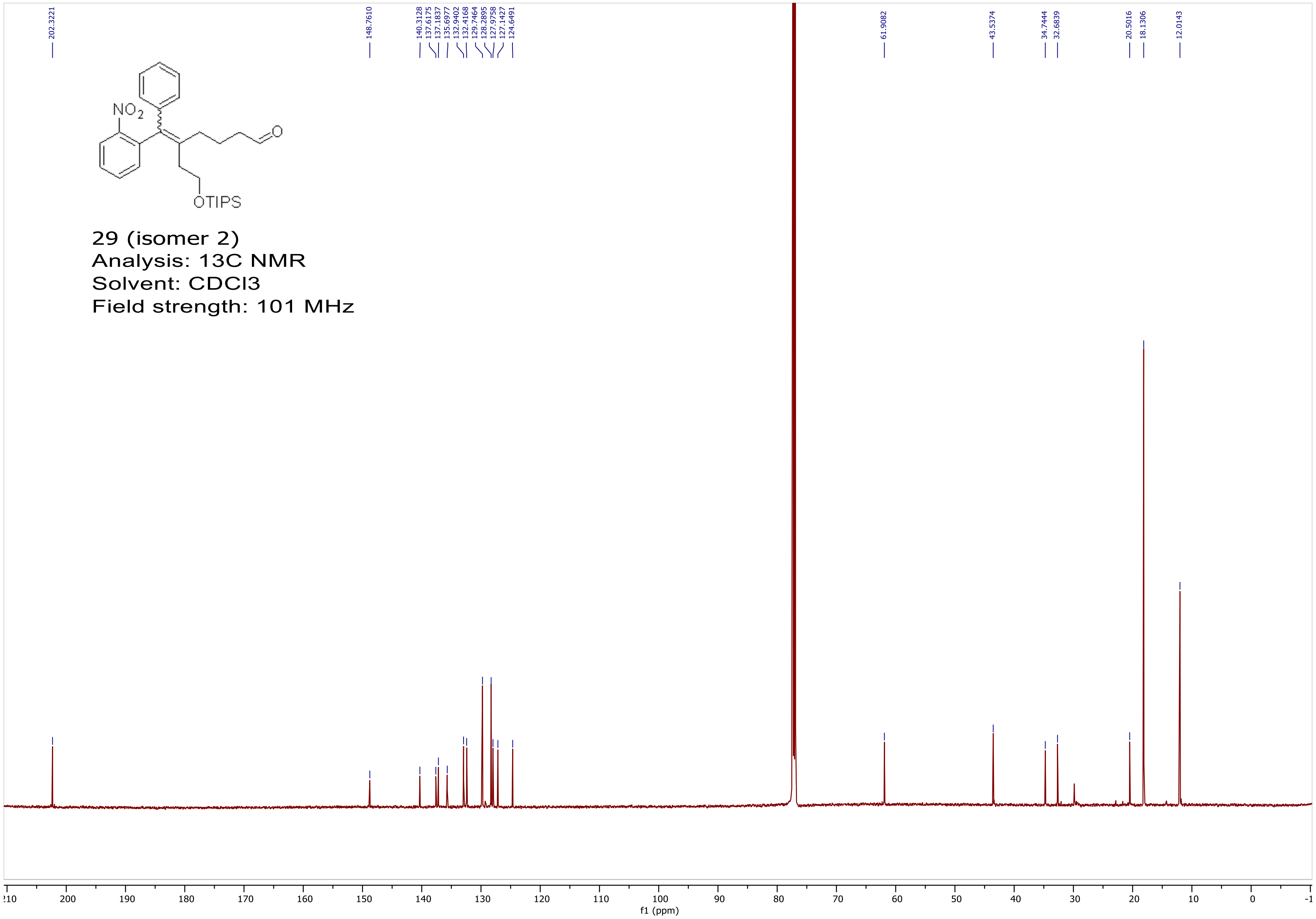
202.3221
148.7610
140.3128
137.6175
137.1837
135.6977
132.9402
132.4168
129.7464
128.2895
127.9758
127.1427
124.6491
61.9082
43.5374
34.7444
32.6839
20.5016
18.1306
12.0143
NO2
O
OTIPS
29 (isomer 2)
Analysis: 13C NMR
Solvent: CDCl3
Field strength: 101 MHz
f1 (ppm)

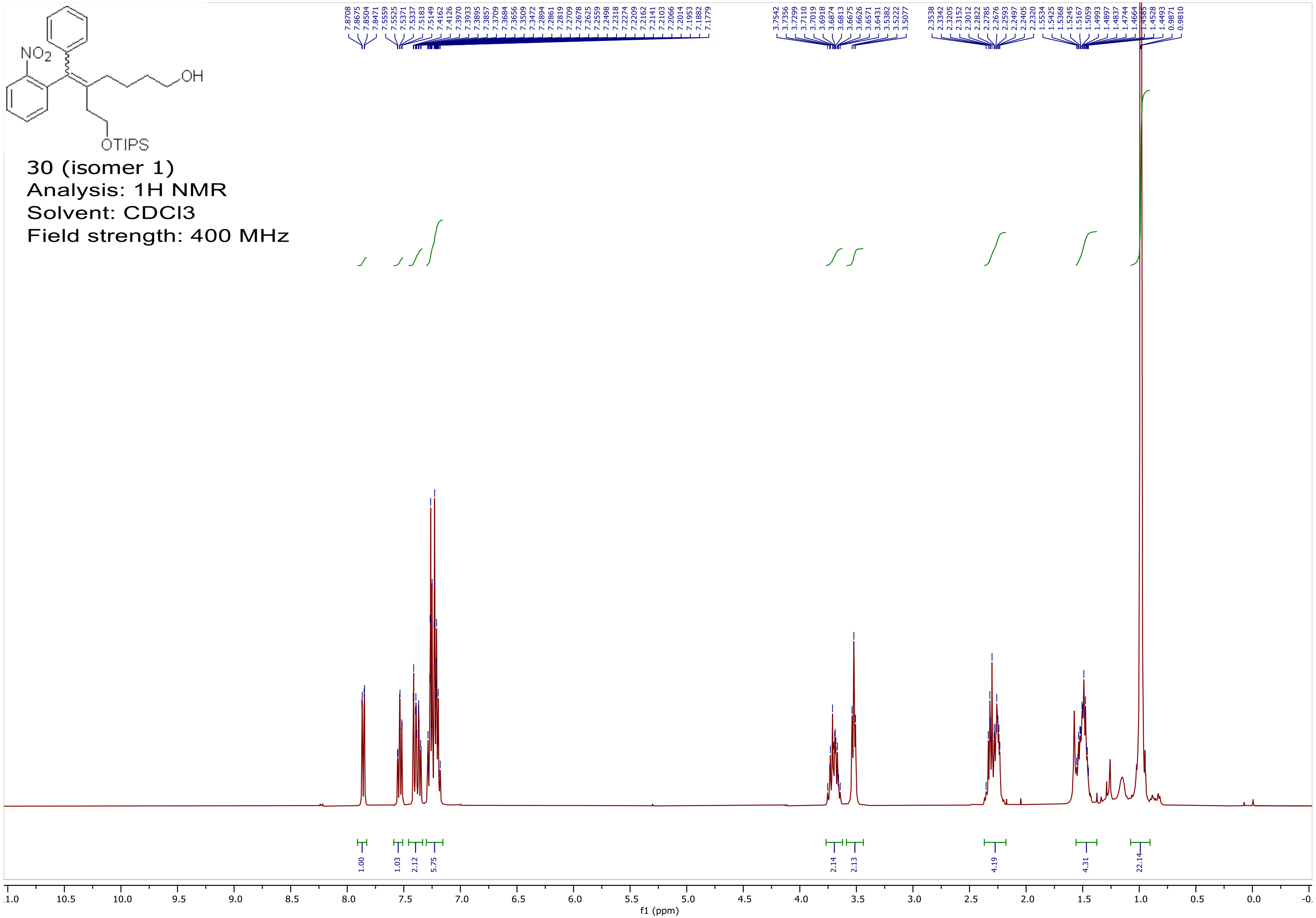

NO2
OH
OTIPS
30 (isomer 1)
Analysis: 1H NMR
Solvent: CDCl3
Field strength: 400 MHz
1.00
1.03
2.12
5.75
2.14
2.13
4.19
4.31
22.14
f1 (ppm)

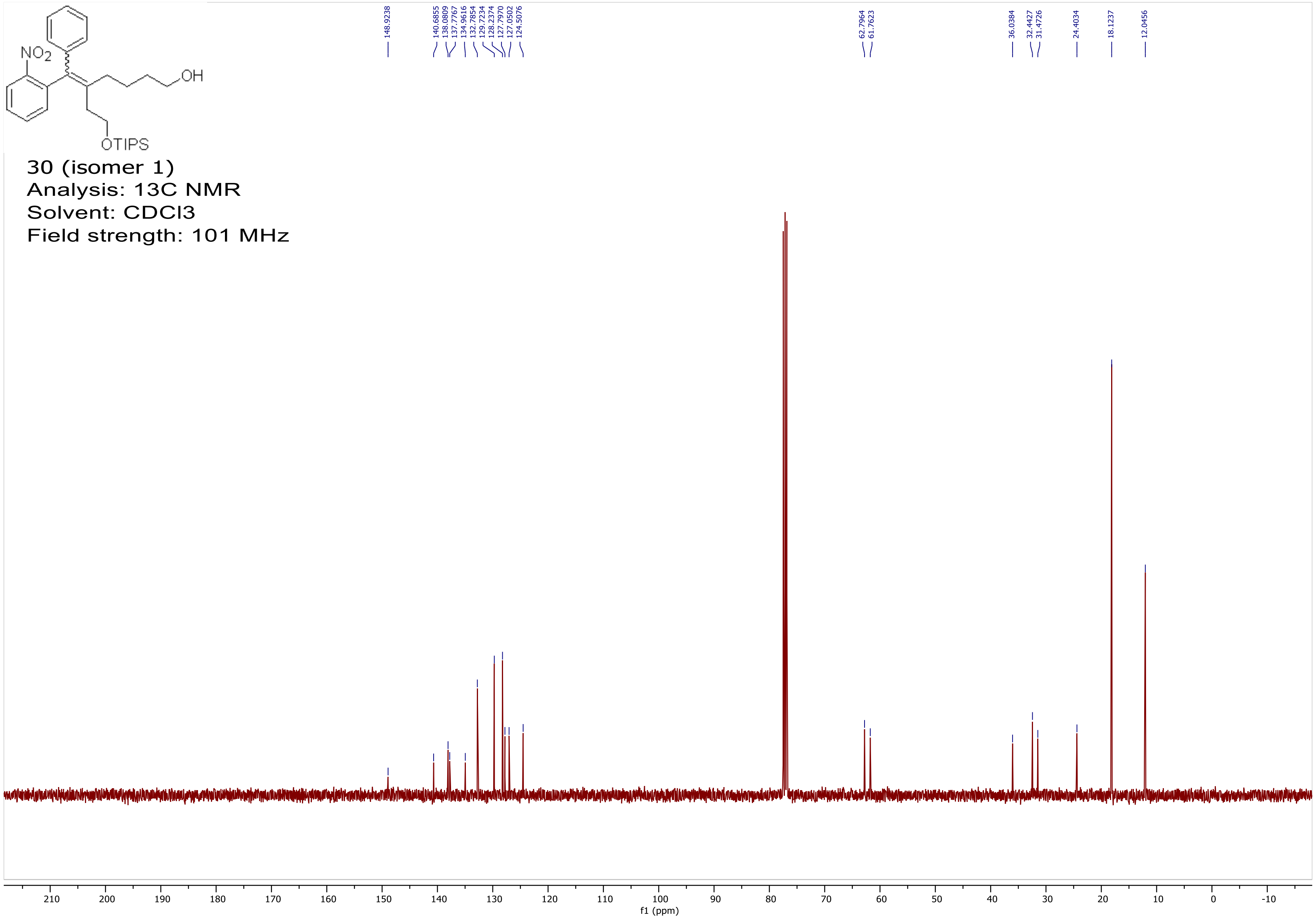

30 (isomer 1)
Analysis: 13C NMR
Solvent: CDCl3
Field strength: 101 MHz

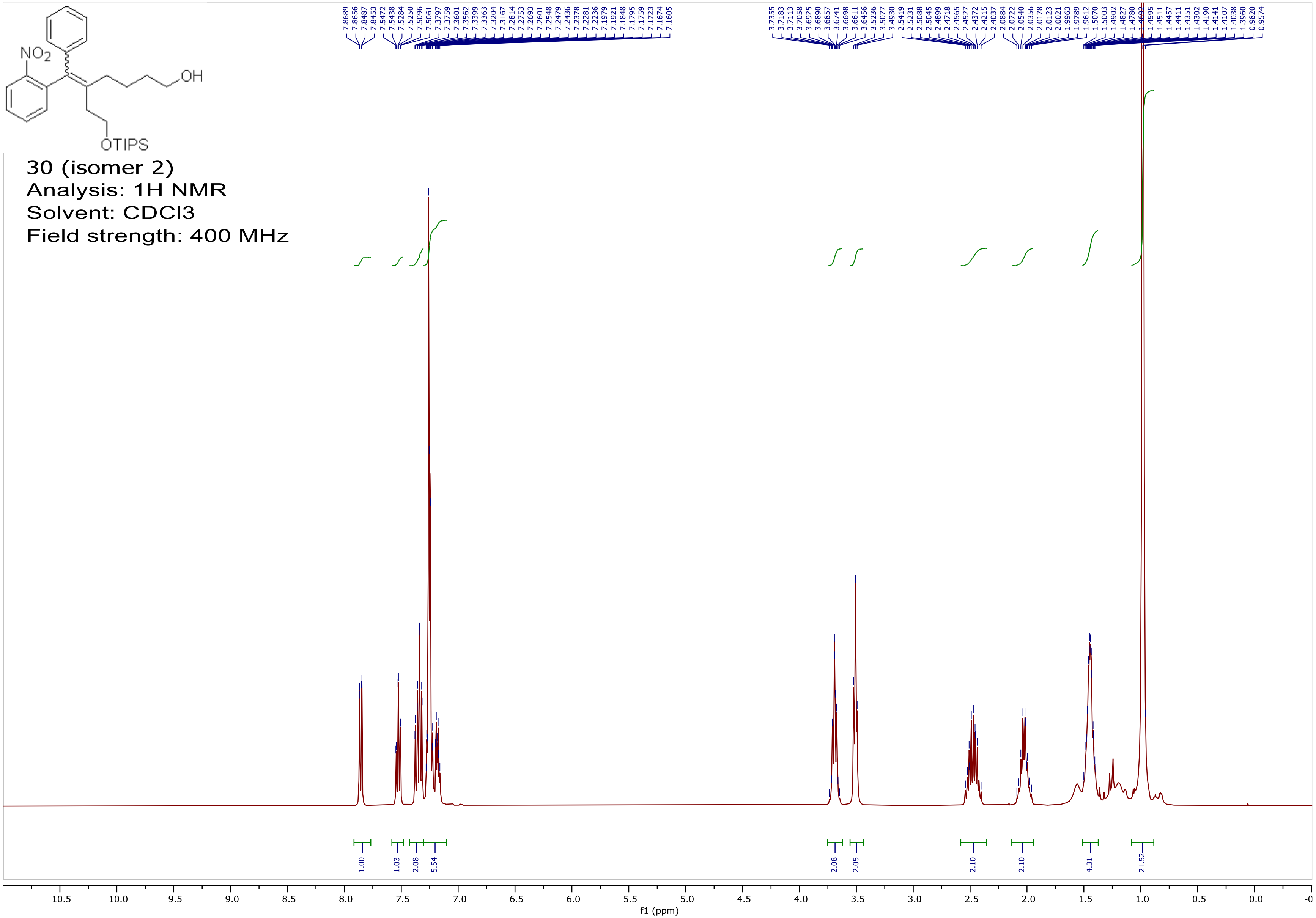
NO2
OH
OTIPS
30 (isomer 2)
Analysis: 1H NMR
Solvent: CDCl3
Field strength: 400 MHz
7.8689 7.8656 7.8487 7.8453 7.5472 7.5438 7.5284 7.5250 7.5096 7.5061 7.3797 7.3759 7.3601 7.3562 7.3399 7.3363 7.3204 7.3167 7.2814 7.2753 7.2693 7.2601 7.2548 7.2479 7.2436 7.2378 7.2281 7.2236 7.1979 7.1921 7.1848 7.1795 7.1759 7.1723 7.1674 7.1605
3.7355 3.7183 3.7113 3.7058 3.6925 3.6890 3.6857 3.6741 3.6698 3.6611 3.6456 3.5236 3.5077 3.4930 2.5419 2.5231 2.5088 2.5045 2.4899 2.4718 2.4565 2.4527 2.4372 2.4215 2.4037 2.0884 2.0722 2.0540 2.0356 2.0178 2.0123 2.0021 1.9963 1.9789 1.9612 1.5070 1.5003 1.4902 1.4827 1.4780 1.4692 1.4595 1.4511 1.4457 1.4411 1.4351 1.4302 1.4190 1.4141 1.4107 1.4038 1.3960 0.9820 0.9574
1.00
1.03
2.08
5.54
2.08
2.05
2.10
2.10
4.31
21.52
10.5 10.0 9.5 9.0 8.5 8.0 7.5 7.0 6.5 6.0 5.5 5.0 4.5 4.0 3.5 3.0 2.5 2.0 1.5 1.0 0.5 0.0
f1 (ppm)

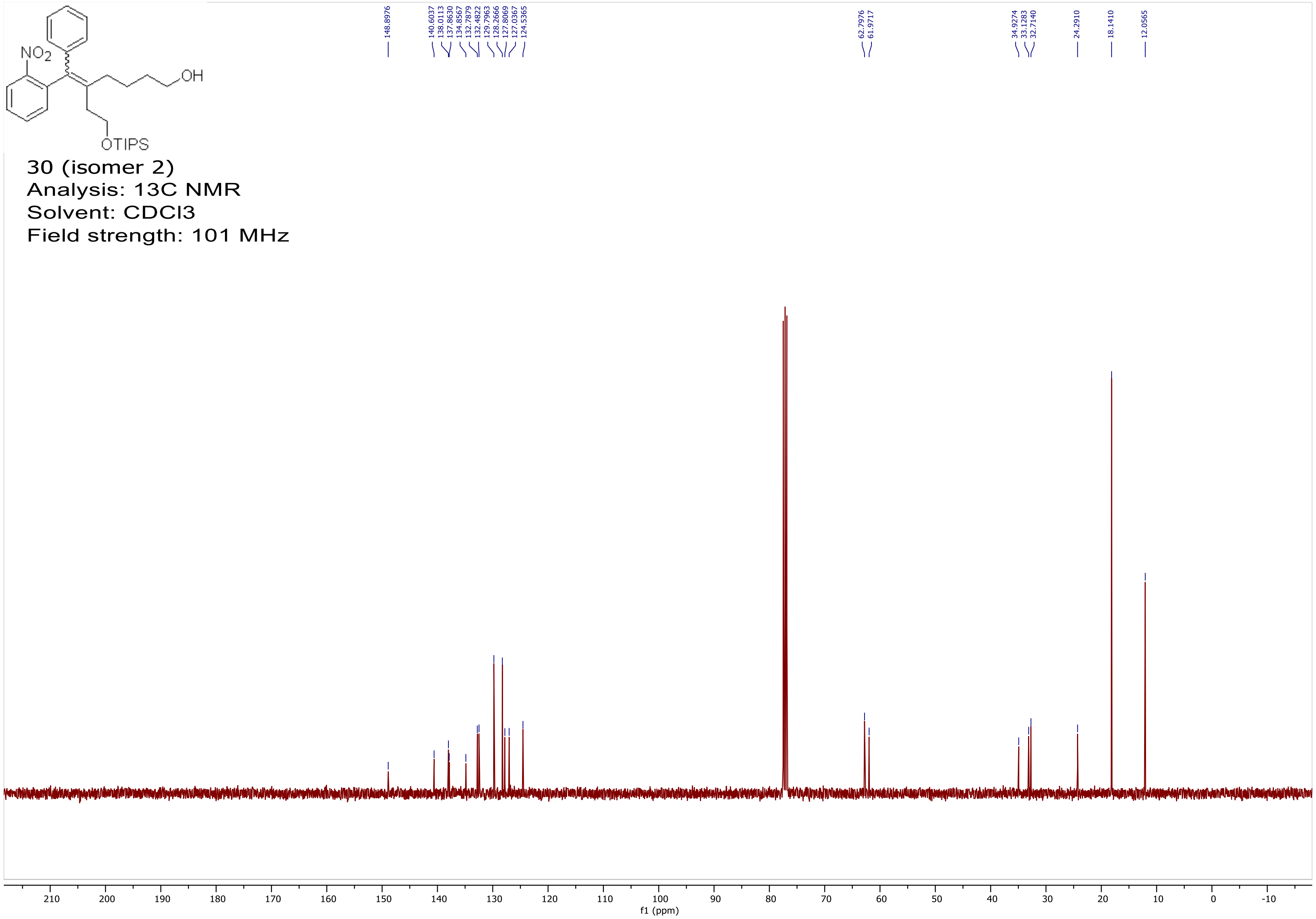

NO2
OH
OTIPS
30 (isomer 2)
Analysis: 13C NMR
Solvent: CDCl3
Field strength: 101 MHz
148.8976
140.6037
138.0113
137.8630
134.8567
132.7879
132.4822
129.7963
128.2666
127.8069
127.0367
124.5365
62.7976
61.9717
34.9274
33.1283
32.7140
24.2910
18.1410
12.0565
210 200 190 180 170 160 150 140 130 120 110 100 90 80 70 60 50 40 30 20 10 0 -10
f1 (ppm)

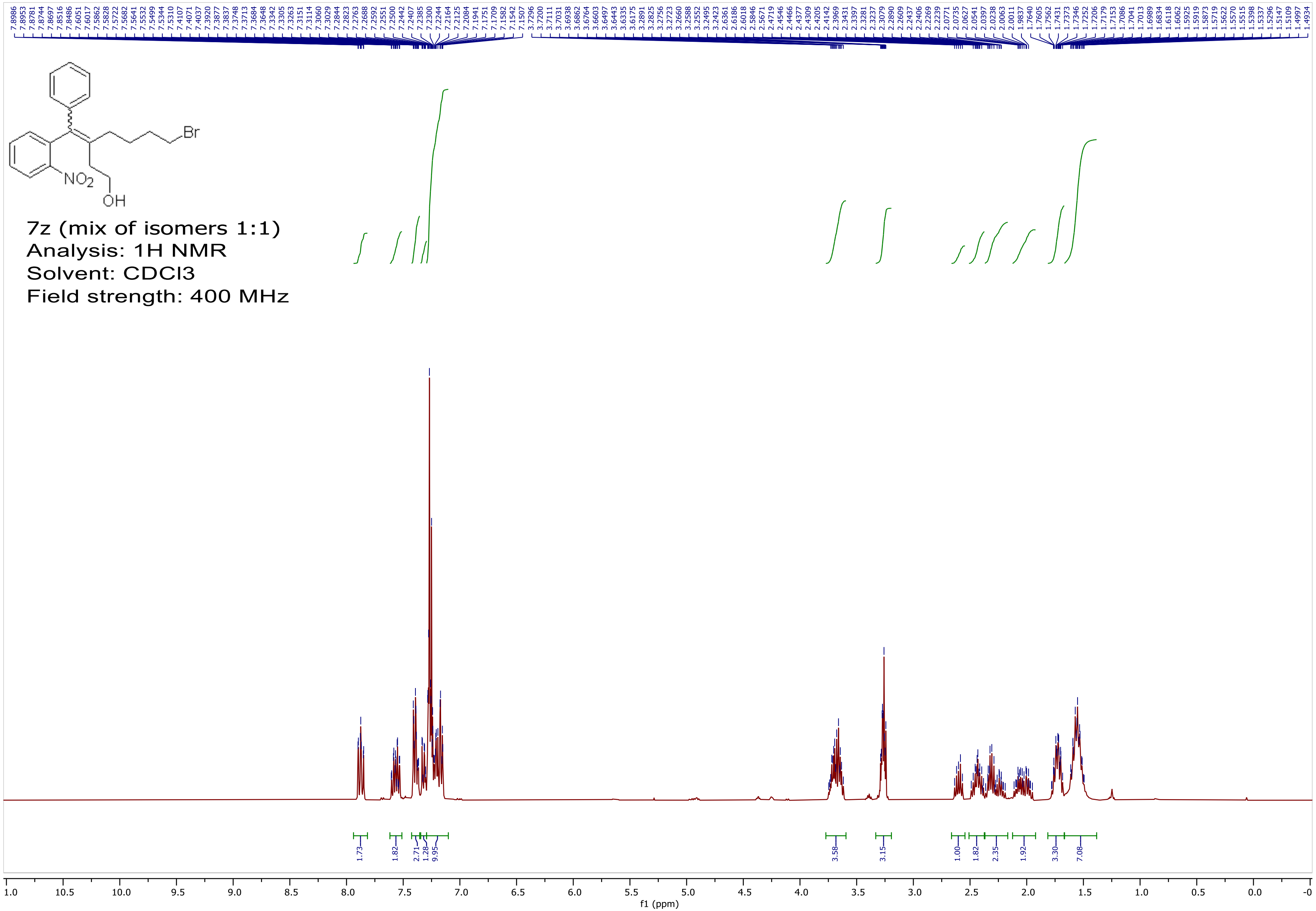
Br
NO2
OH
7z (mix of isomers 1:1)
Analysis: 1H NMR
Solvent: CDCl3
Field strength: 400 MHz
1.73
1.82
2.71
1.28
9.95
3.58
3.15
1.00
1.82
2.35
1.92
3.30
7.08
f1 (ppm)

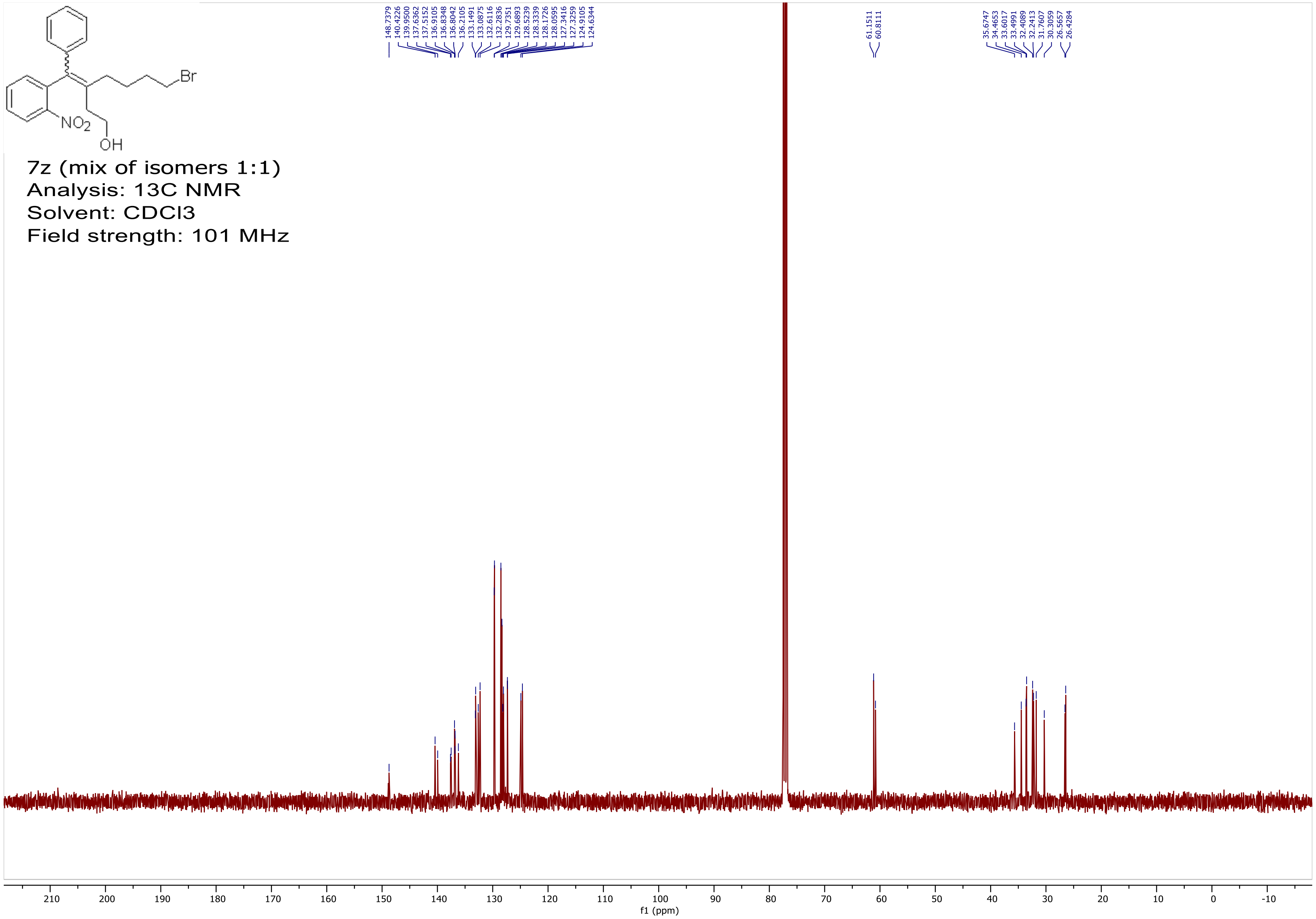
Br
NO_2
OH
7z (mix of isomers 1:1)
Analysis: 13C NMR
Solvent: CDCl3
Field strength: 101 MHz
148.7379
140.4226
139.9500
137.6362
137.5152
136.9105
136.8348
136.8042
136.2105
133.1491
133.0875
132.6116
132.2836
129.7351
129.6893
128.5239
128.3339
128.1726
128.0595
127.3416
127.3259
124.9105
124.6344
61.1511
60.8111
35.6747
34.4653
33.6017
33.4991
32.4089
32.2413
31.7607
30.3059
26.5657
26.4284
210
200
190
180
170
160
150
140
130
120
110
100
90
80
70
60
50
40
30
20
10
0
-10
f1 (ppm)

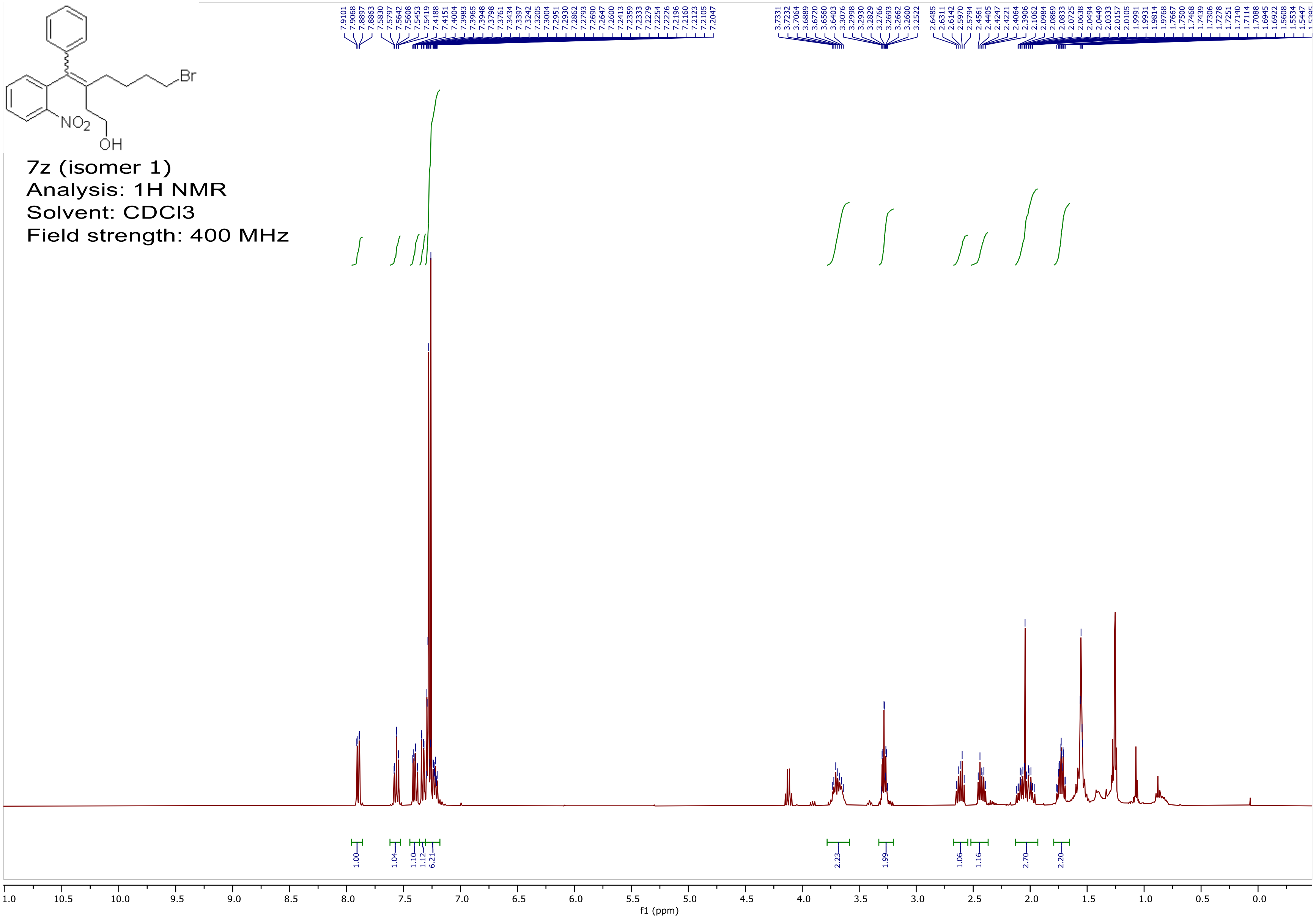
Br
NO2
OH
7z (isomer 1)
Analysis: 1H NMR
Solvent: CDCl3
Field strength: 400 MHz
f1 (ppm)

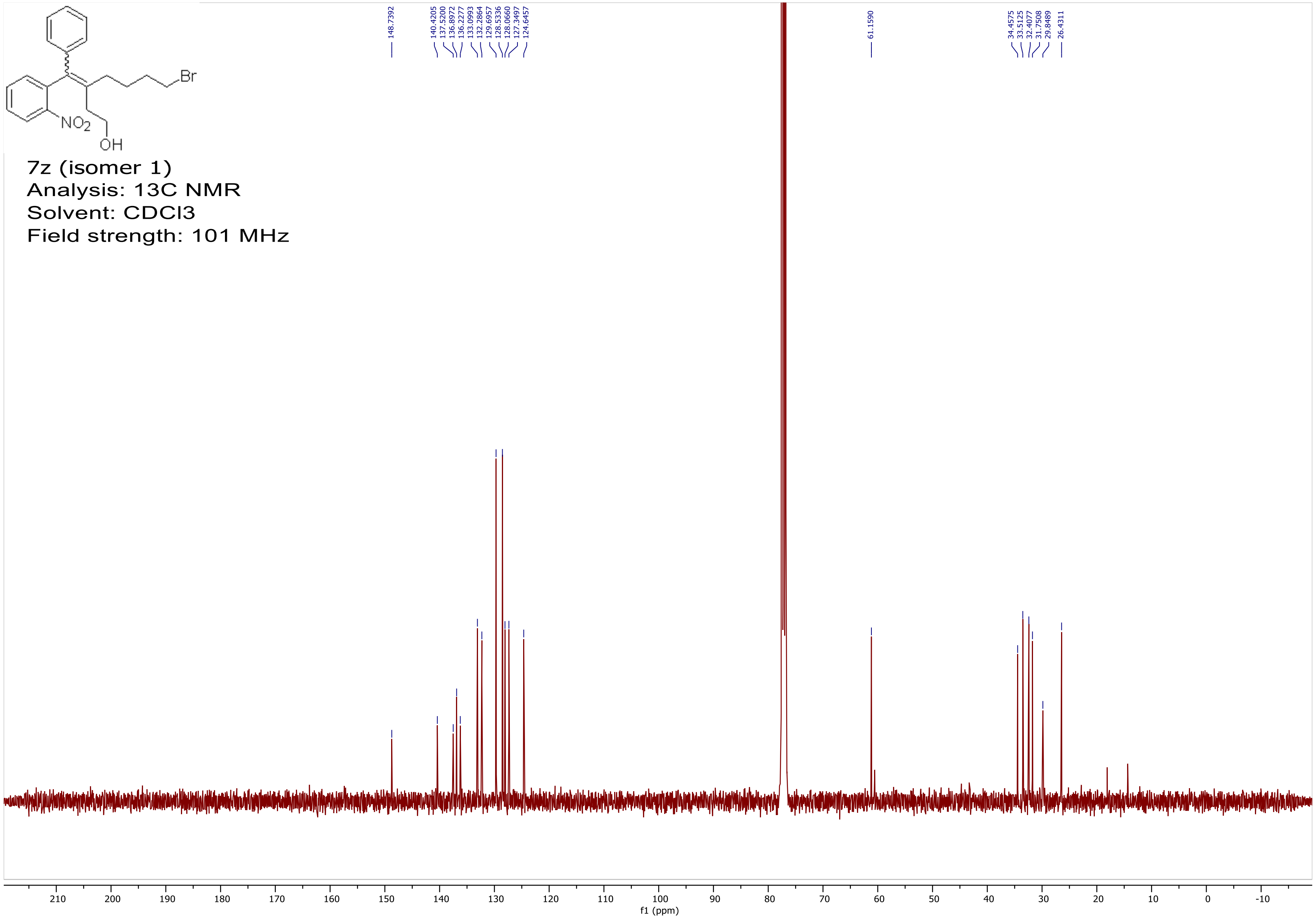

7z (isomer 1)
Analysis: 13C NMR
Solvent: CDCl3
Field strength: 101 MHz

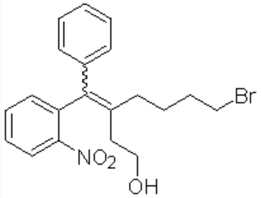

7z (isomer 2)
Analysis: 1H NMR
Solvent: CDCl3
Field strength: 400 MHz

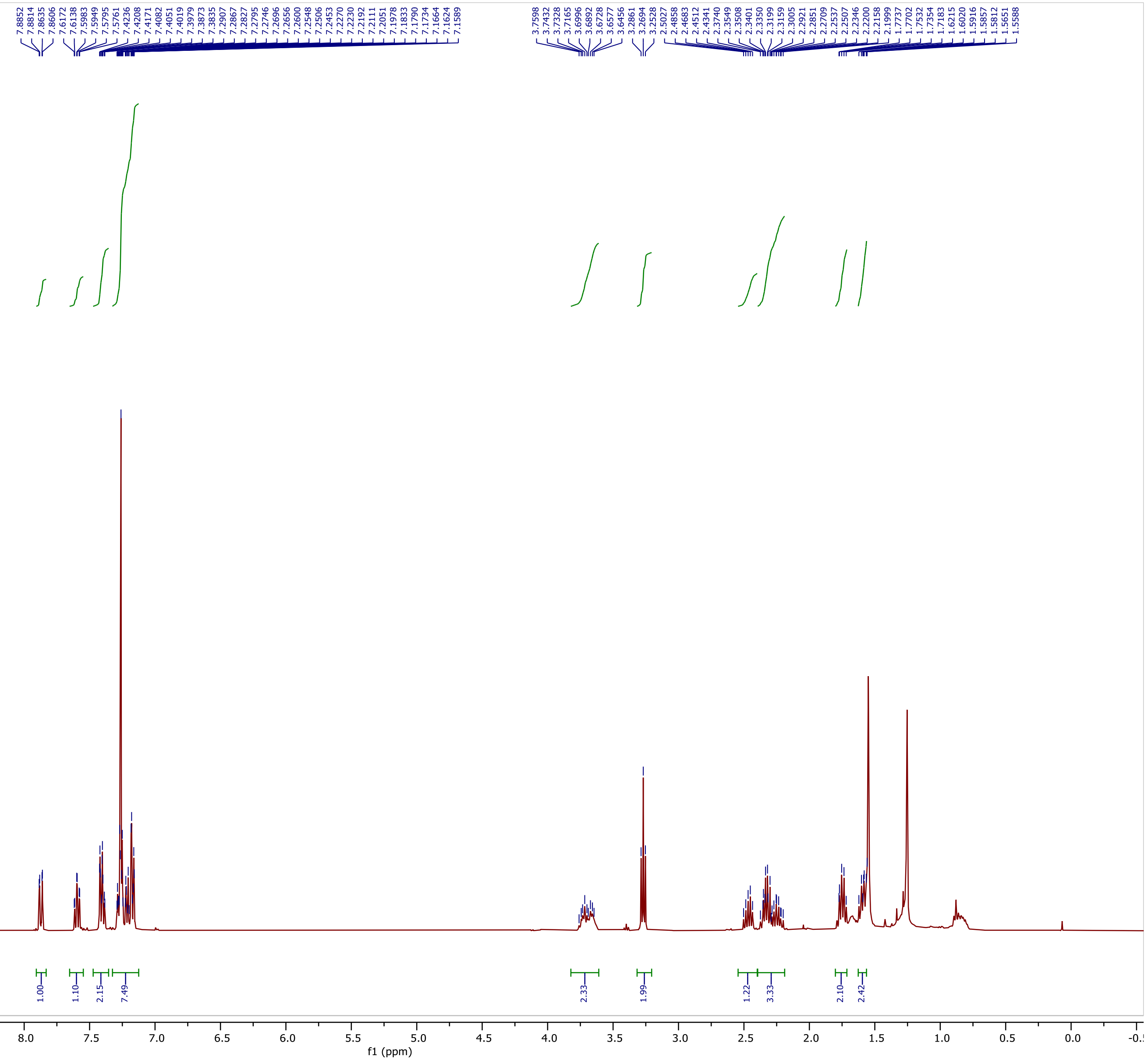

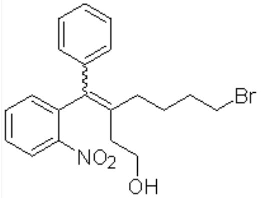

7z (isomer 2)
Analysis: 13C NMR
Solvent: CDCl3
Field strength: 101 MHz

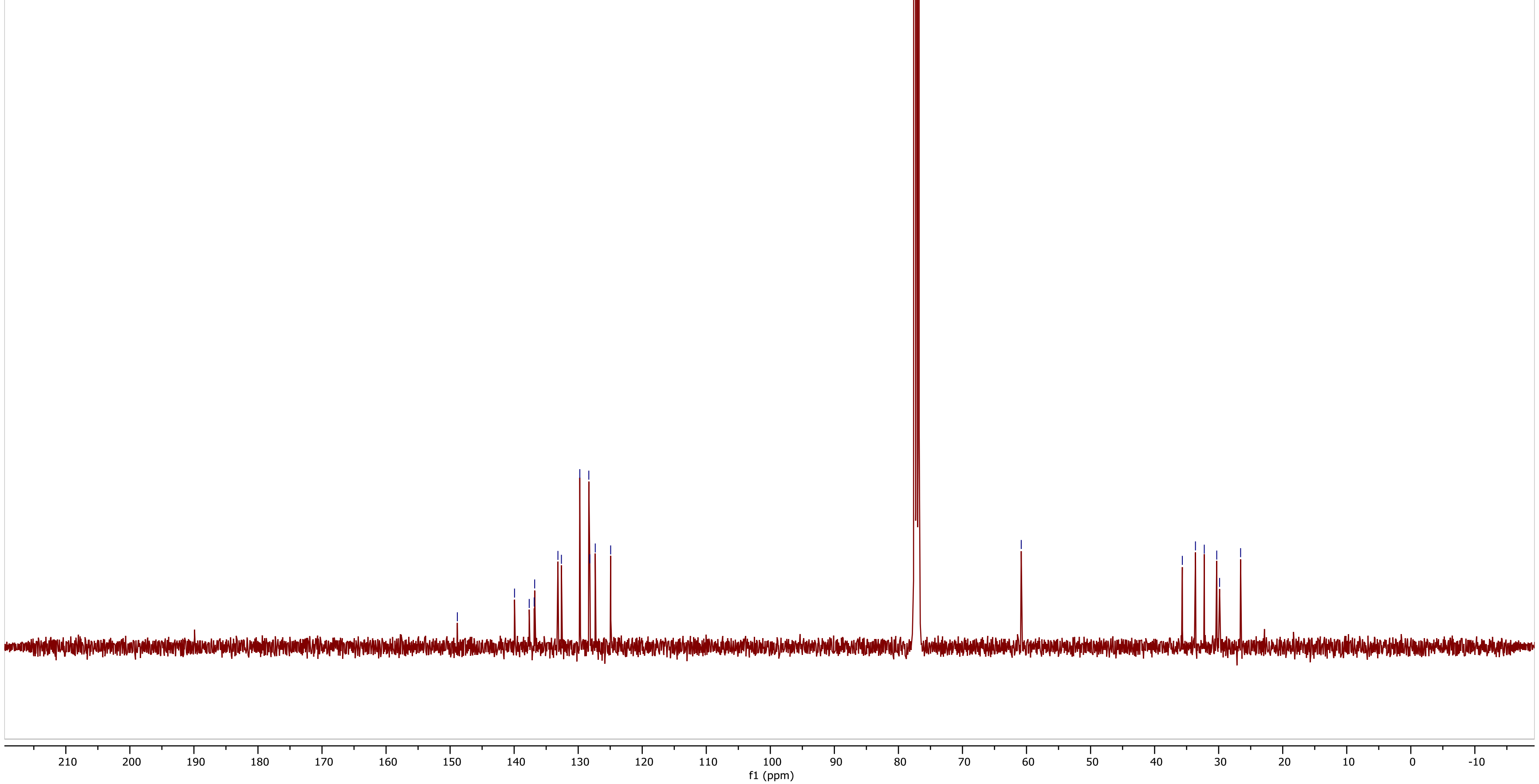

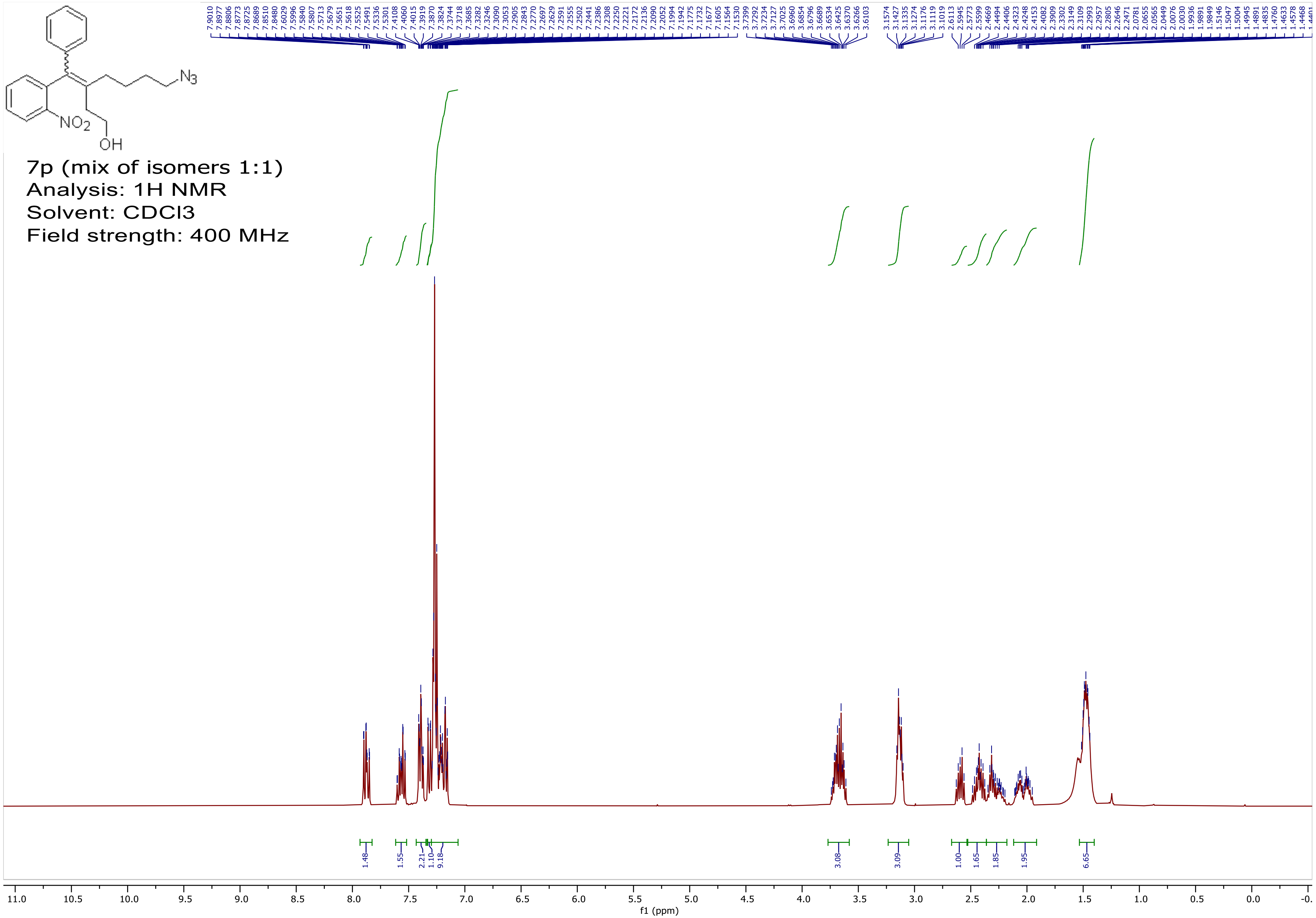
N3
NO2
OH
7p (mix of isomers 1:1)
Analysis: 1H NMR
Solvent: CDCl3
Field strength: 400 MHz
1.48
1.55
2.21
1.10
9.18
3.08
3.09
1.00
1.65
1.85
1.95
6.65
f1 (ppm)

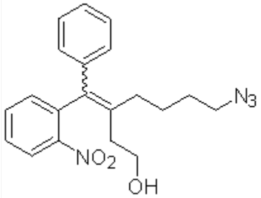

7p (mix of isomers 1:1)
Analysis: 13C NMR
Solvent: CDCl3
Field strength: 101 MHz

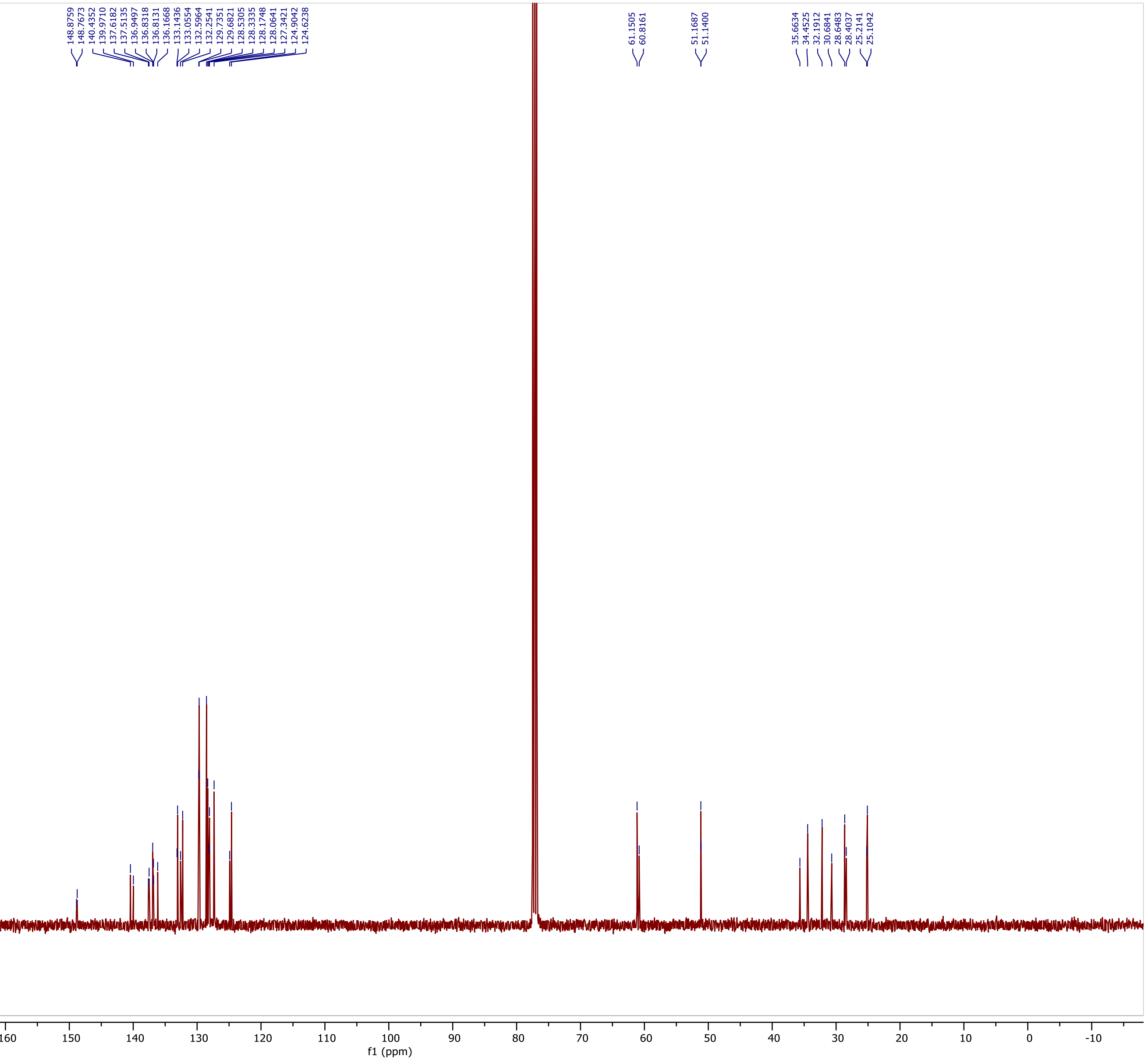

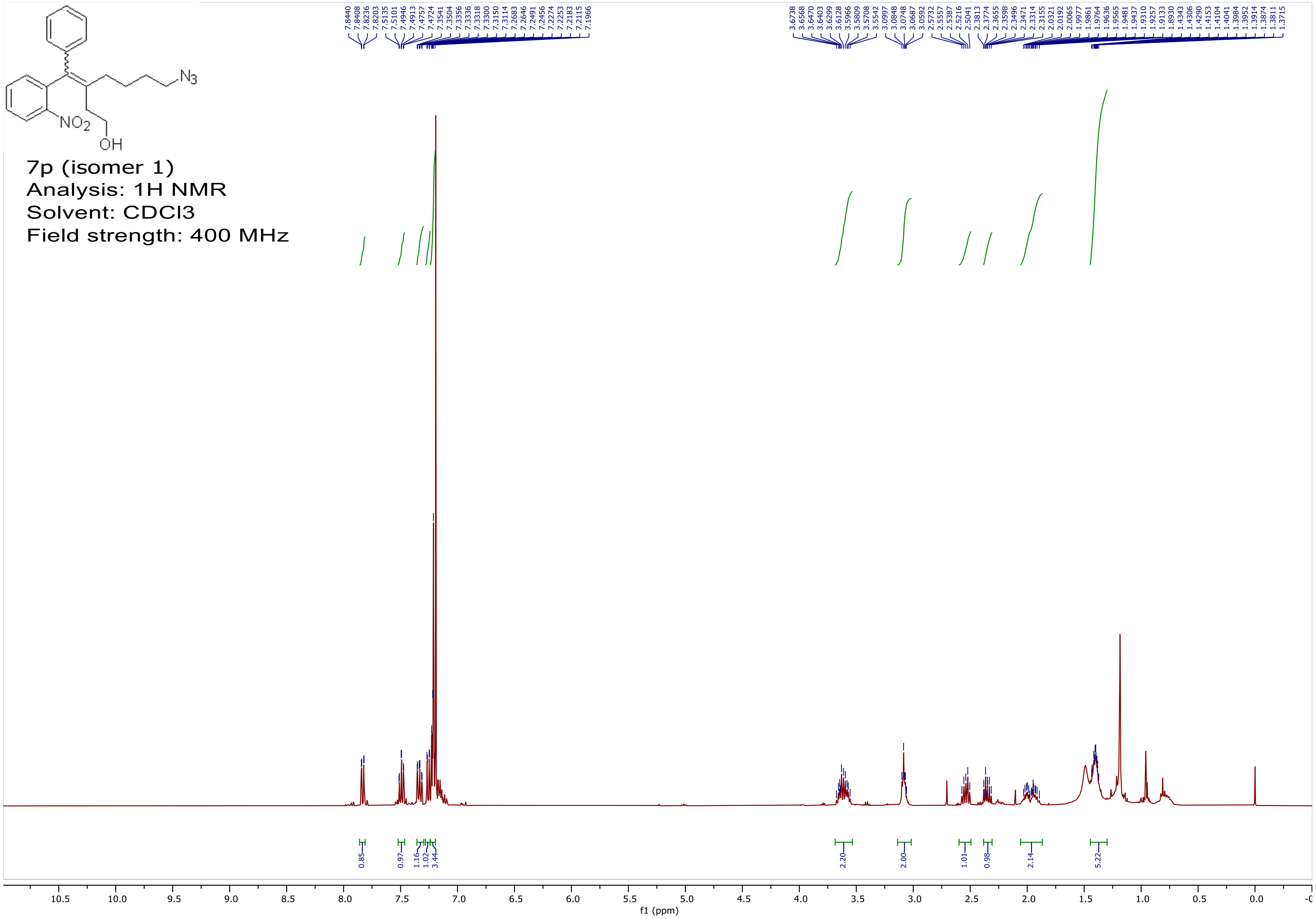

N3
NO2
OH
7p (isomer 1)
Analysis: 1H NMR
Solvent: CDCl3
Field strength: 400 MHz
7.8440 7.8408 7.8236 7.8203 7.5135 7.5101 7.4946 7.4913 7.4757 7.4724 7.3541 7.3504 7.3356 7.3336 7.3318 7.3300 7.3150 7.3114 7.2683 7.2646 7.2491 7.2456 7.2274 7.2253 7.2183 7.2115 7.1966
3.6738 3.6568 3.6470 3.6403 3.6299 3.6128 3.5966 3.5809 3.5708 3.5542 3.0997 3.0848 3.0748 3.0687 3.0592 2.5732 2.5557 2.5387 2.5216 2.5041 2.3813 2.3774 2.3655 2.3598 2.3496 2.3471 2.3314 2.3155 2.0321 2.0192 2.0065 1.9977 1.9861 1.9764 1.9636 1.9565 1.9481 1.9437 1.9310 1.9257 1.9133 1.8930 1.4343 1.4306 1.4290 1.4153 1.4104 1.4041 1.3984 1.3952 1.3914 1.3874 1.3811 1.3715
0.85 0.97 1.16 1.02 3.44 2.20 2.00 1.01 0.98 2.14 5.22
10.5 10.0 9.5 9.0 8.5 8.0 7.5 7.0 6.5 6.0 5.5 5.0 4.5 4.0 3.5 3.0 2.5 2.0 1.5 1.0 0.5 0.0
f1 (ppm)

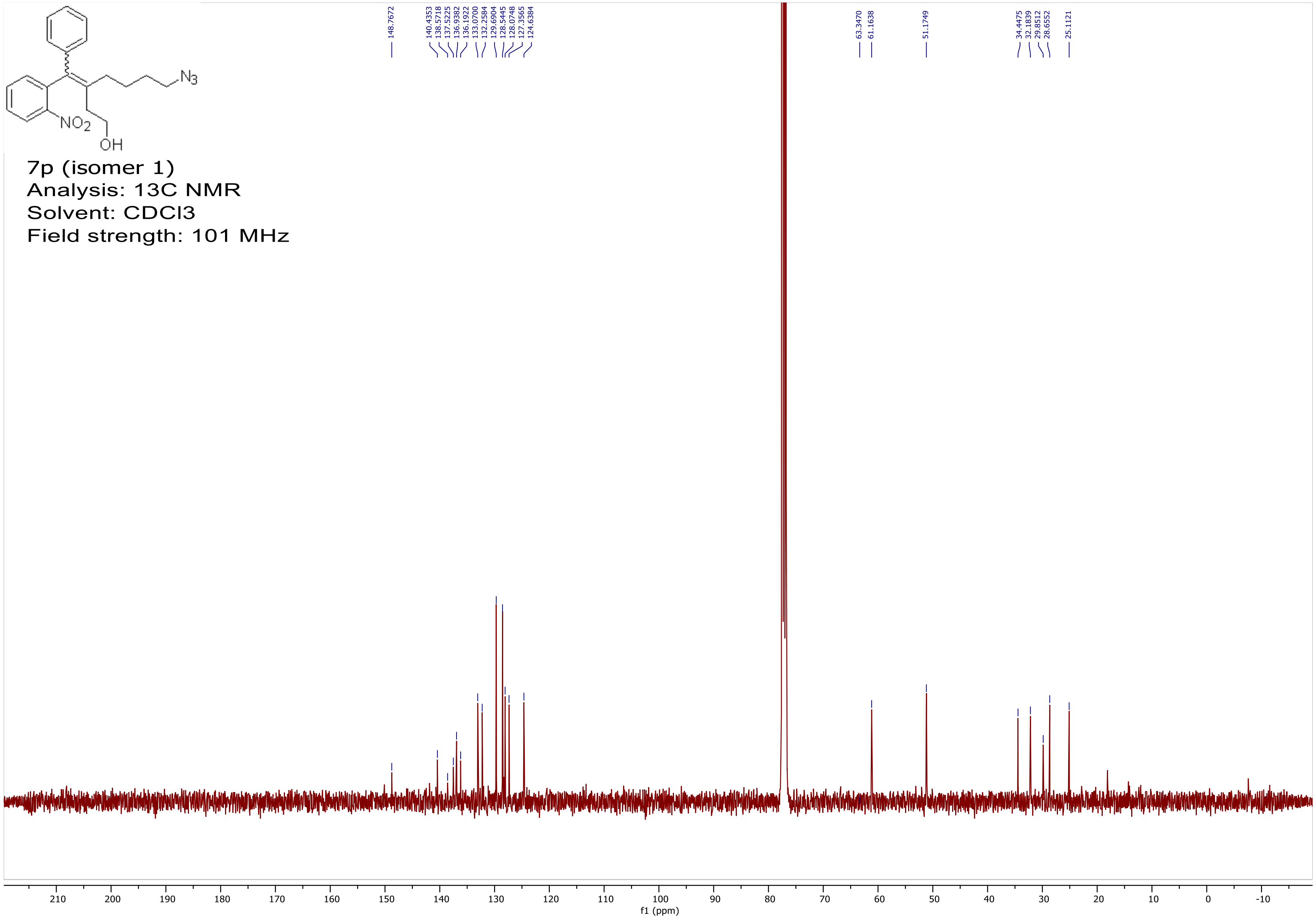

7p (isomer 1)
Analysis: 13C NMR
Solvent: CDCl3
Field strength: 101 MHz

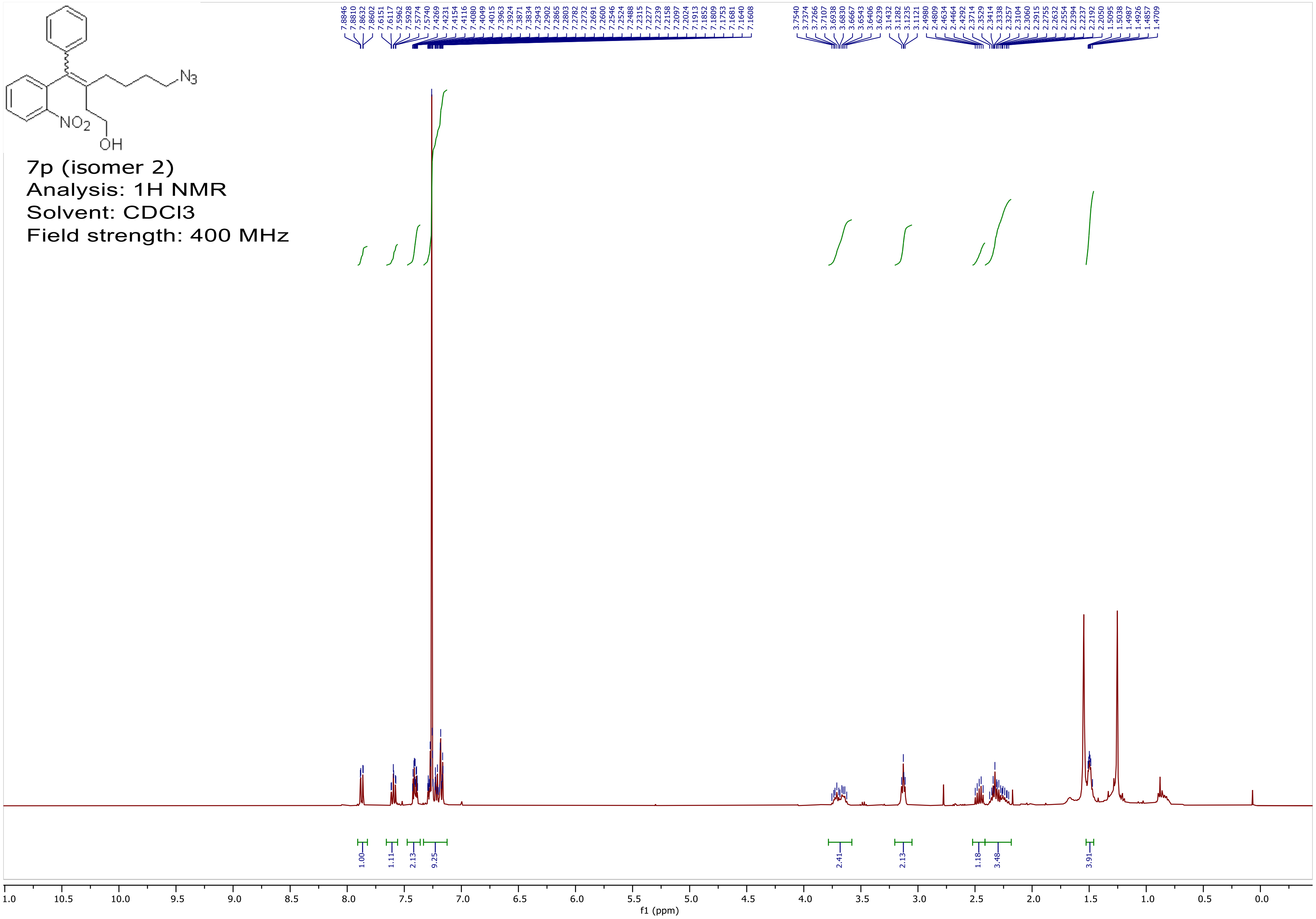
NO2
OH
N3
7p (isomer 2)
Analysis: 1H NMR
Solvent: CDCl3
Field strength: 400 MHz
f1 (ppm)

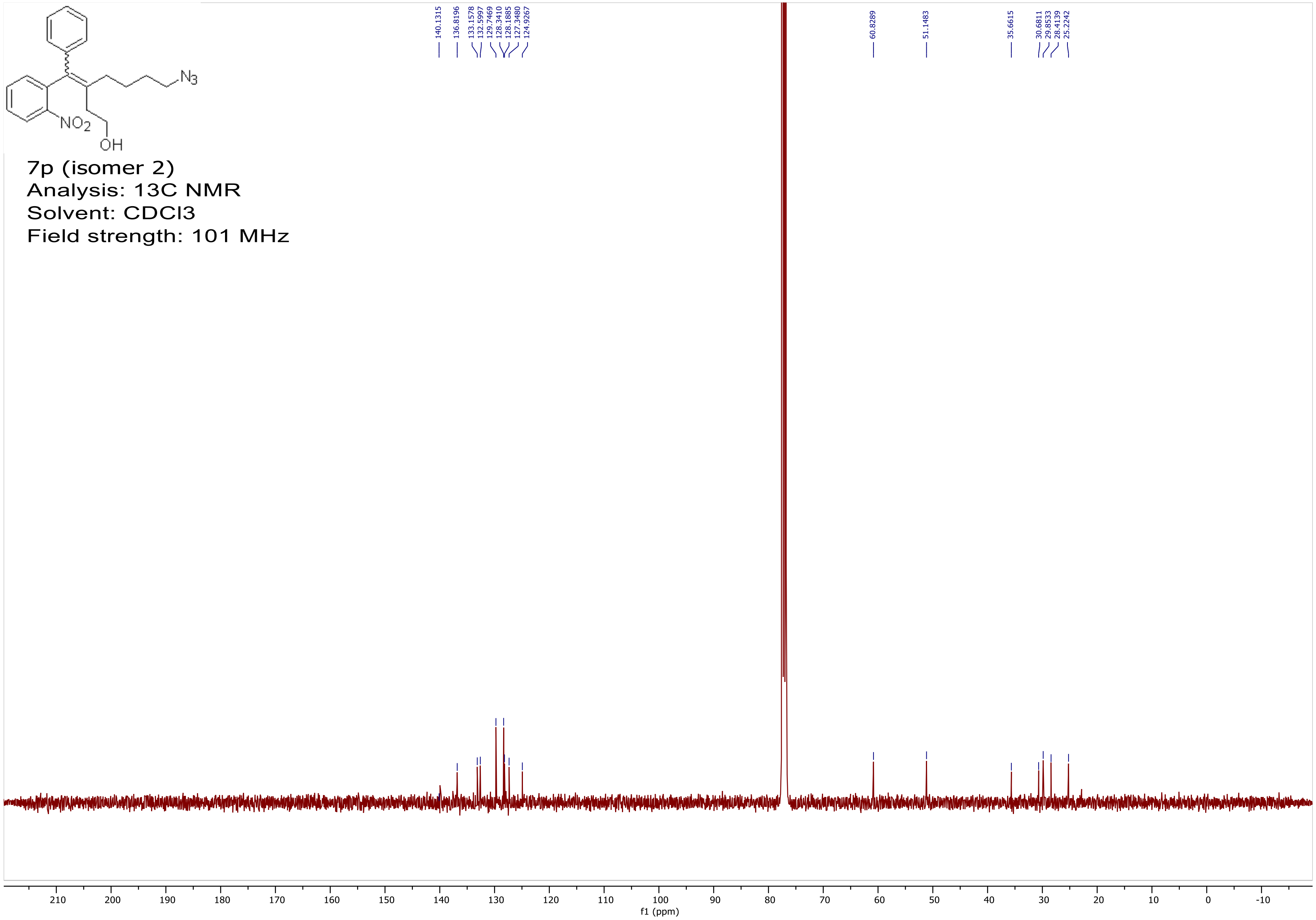
N3
NO2
OH
7p (isomer 2)
Analysis: 13C NMR
Solvent: CDCl3
Field strength: 101 MHz
140.1315
136.8196
133.1578
132.5997
129.7469
128.3410
128.1885
127.3480
124.9267
60.8289
51.1483
35.6615
30.6811
29.8533
28.4139
25.2242
210 200 190 180 170 160 150 140 130 120 110 100 90 80 70 60 50 40 30 20 10 0 -10
f1 (ppm)

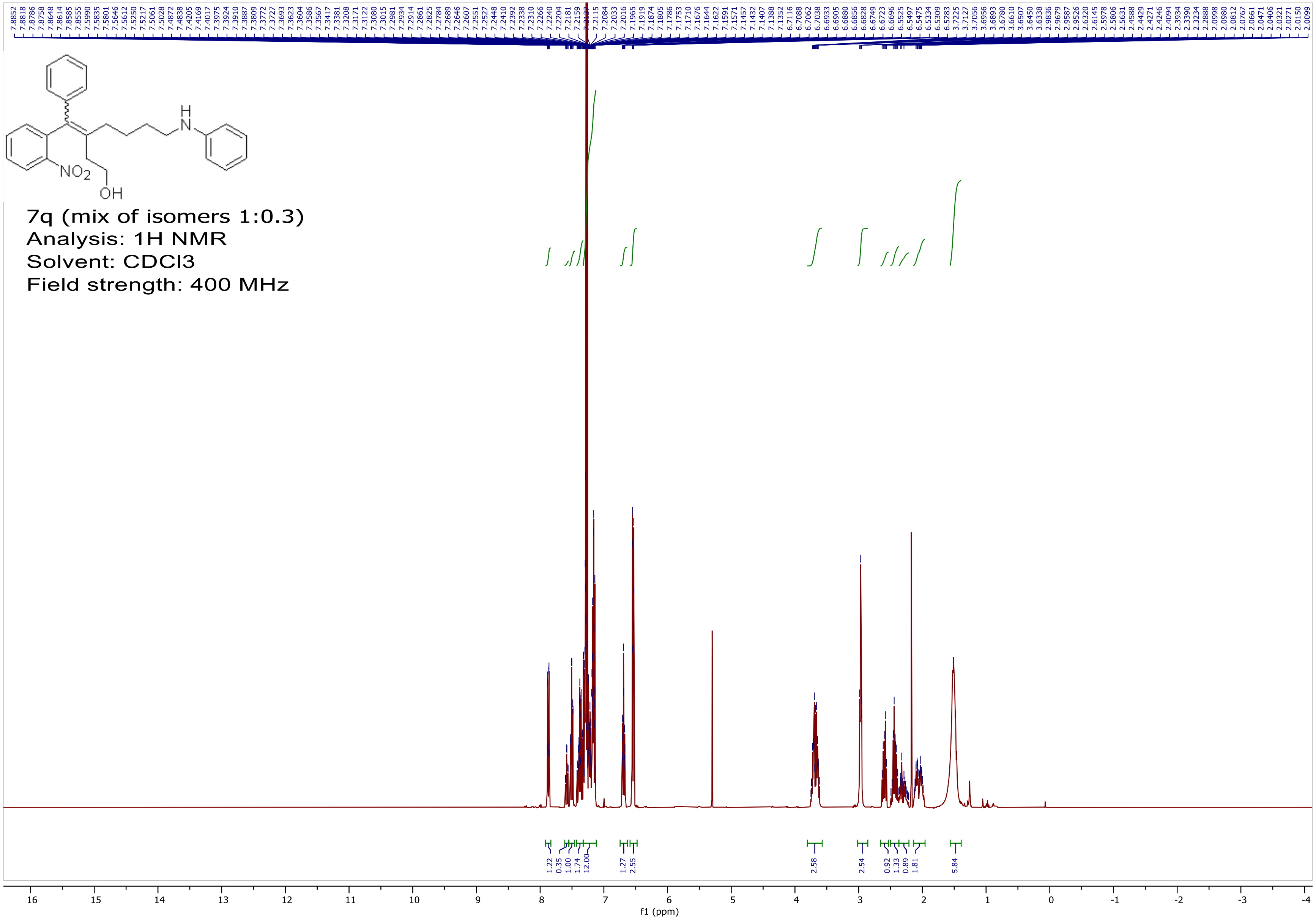
NO2
OH
H
N
7q (mix of isomers 1:0.3)
Analysis: 1H NMR
Solvent: CDCl3
Field strength: 400 MHz
f1 (ppm)

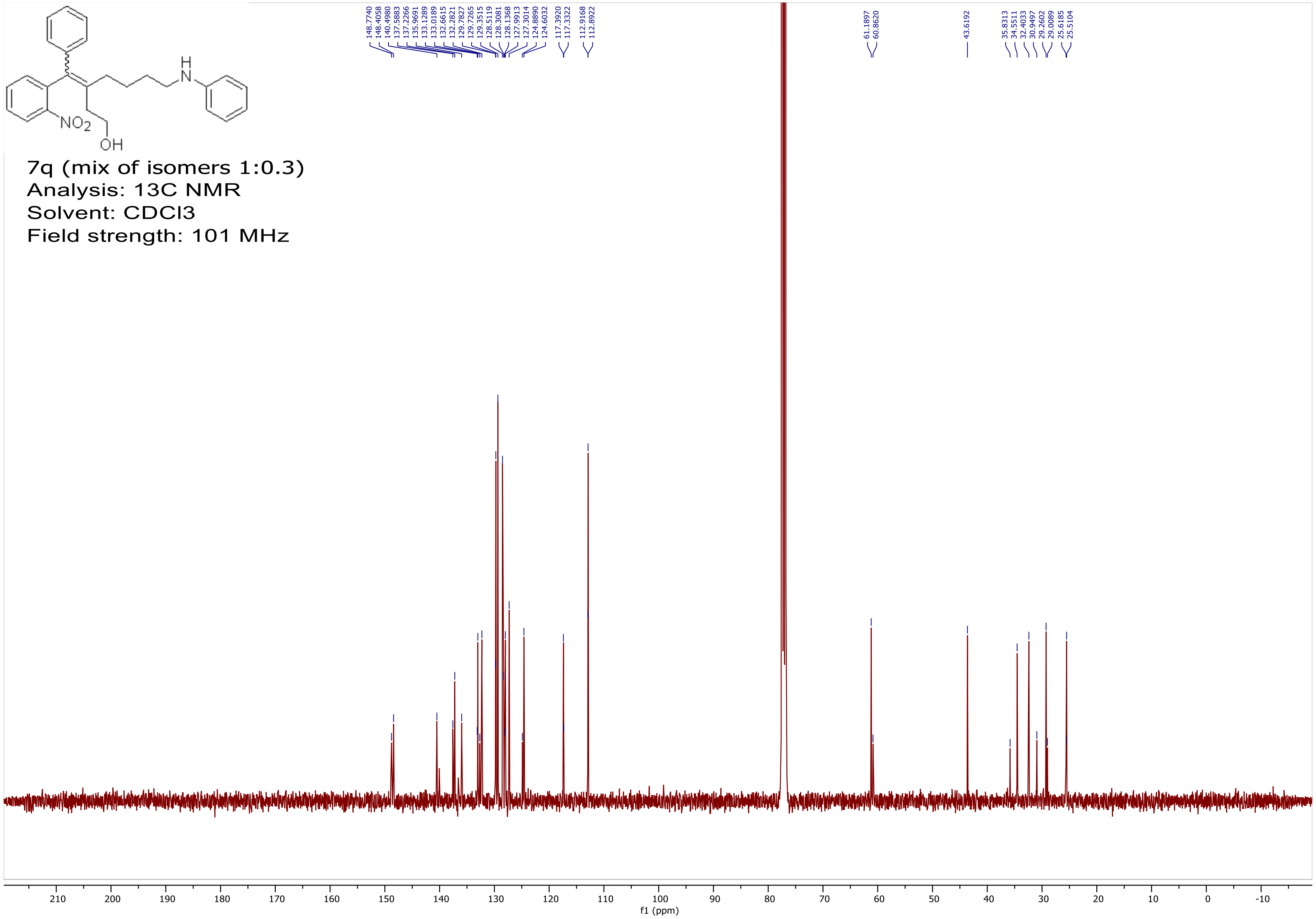

NO2
OH
H
N
7q (mix of isomers 1:0.3)
Analysis: 13C NMR
Solvent: CDCl3
Field strength: 101 MHz
148.7740
148.4058
140.4980
137.5883
137.2266
135.9691
133.1289
133.0189
132.6615
132.2821
129.7827
129.7265
129.3515
128.5119
128.3081
128.1368
127.9913
127.3014
124.8890
124.6032
117.3920
117.3322
112.9168
112.8922
61.1897
60.8620
43.6192
35.8313
34.5511
32.4033
30.9497
29.2602
29.0089
25.6185
25.5104
210 200 190 180 170 160 150 140 130 120 110 100 90 80 70 60 50 40 30 20 10 0 -10
f1 (ppm)

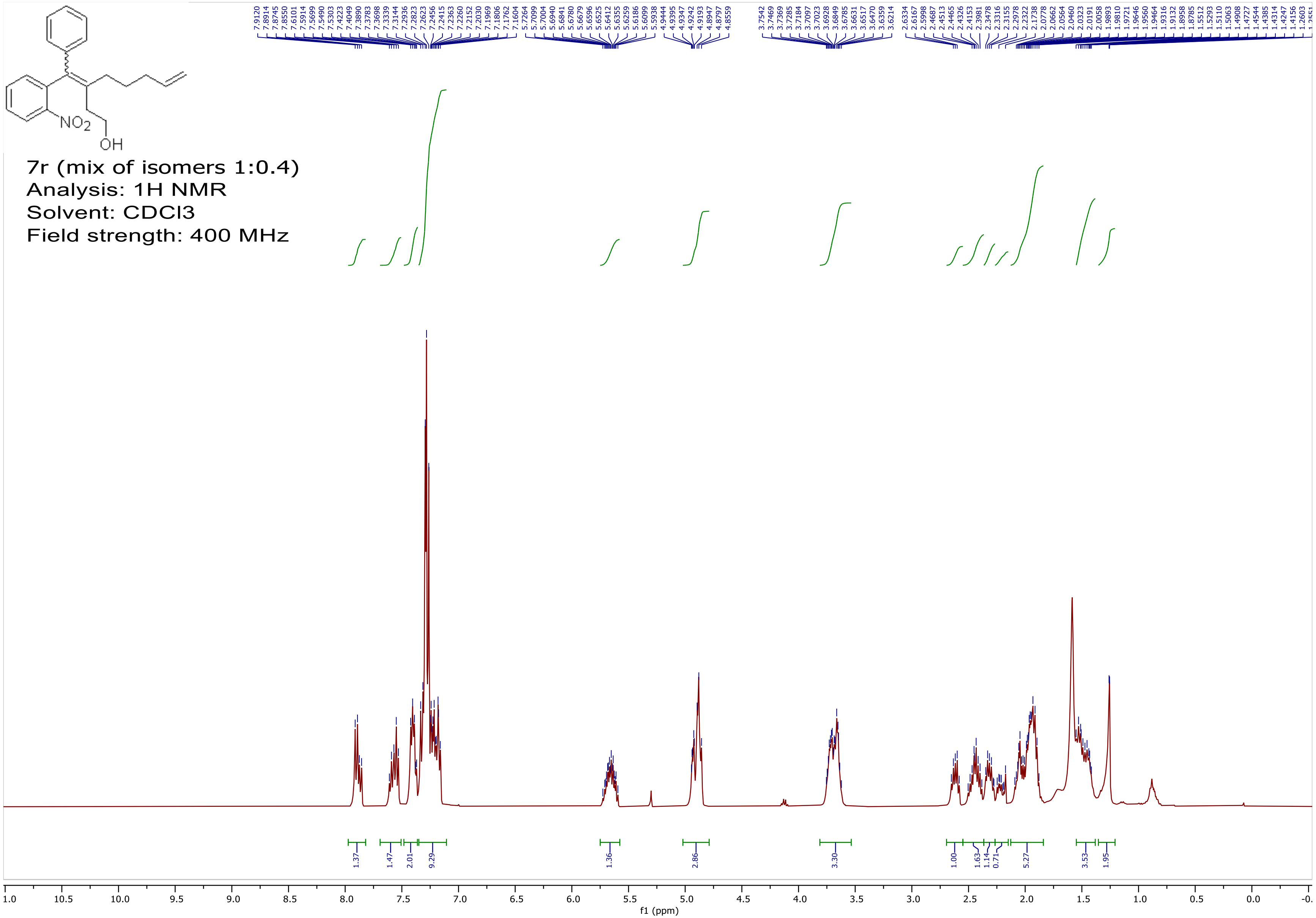
NO2
OH
7r (mix of isomers 1:0.4)
Analysis: 1H NMR
Solvent: CDCl3
Field strength: 400 MHz
f1 (ppm)

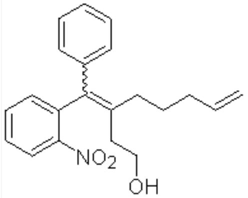

7r (mix of isomers 1:0.4)
Analysis: 13C NMR
Solvent: CDCl3
Field strength: 101 MHz

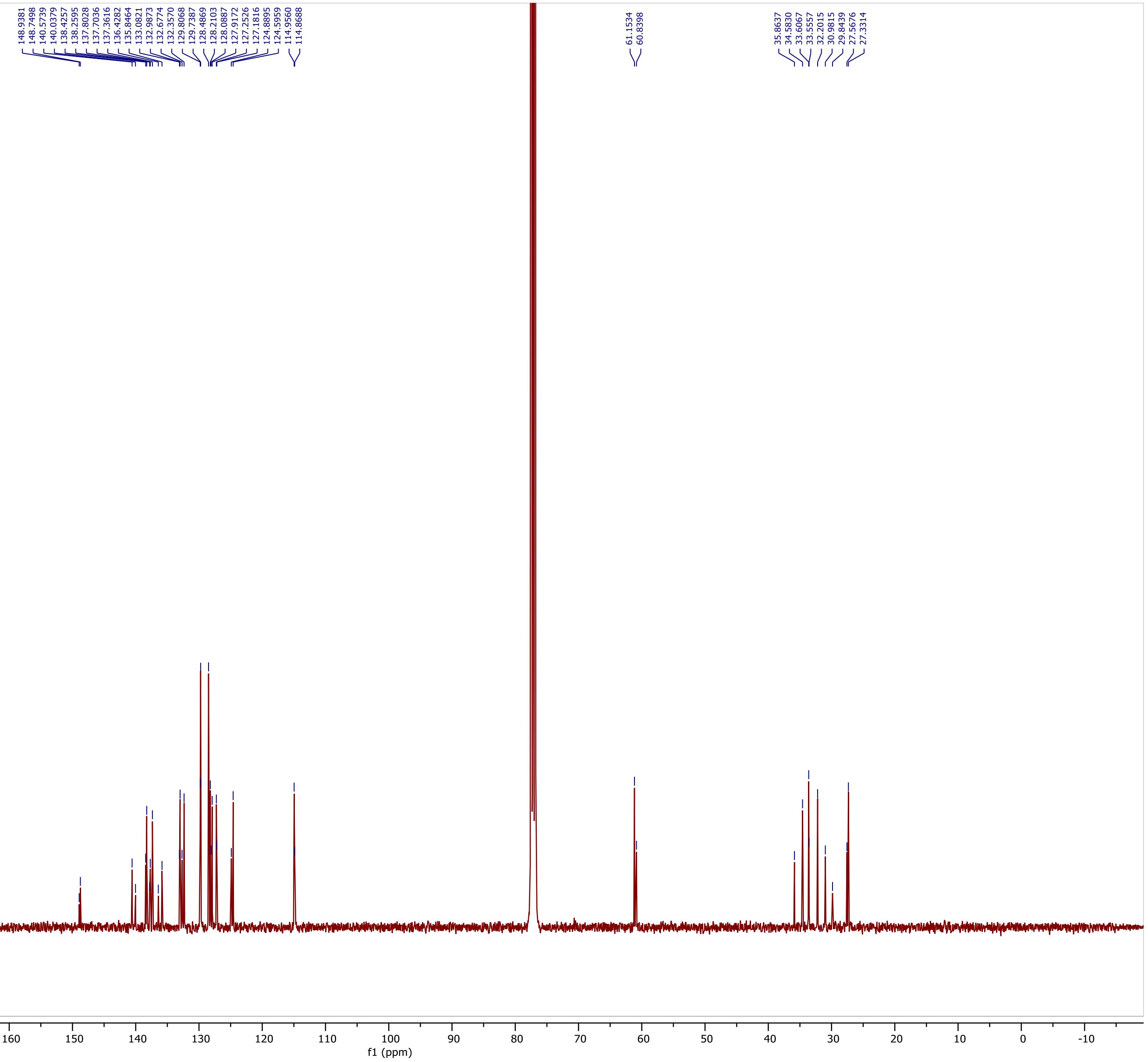

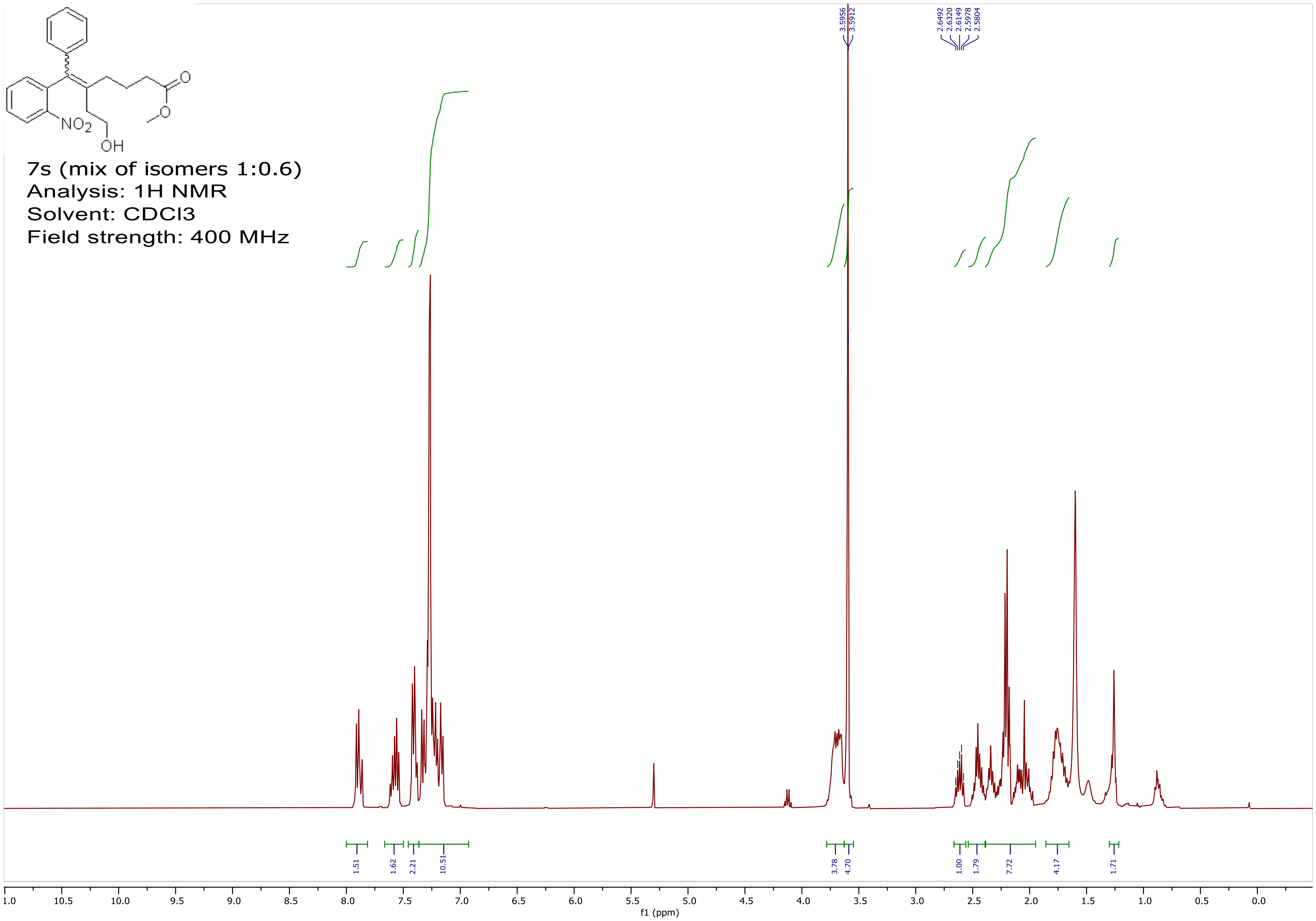

NO2
OH
O
O
7s (mix of isomers 1:0.6)
Analysis: 1H NMR
Solvent: CDCl3
Field strength: 400 MHz
3.5956
3.5912
2.6492
2.6320
2.6149
2.5978
2.5804
1.51
1.62
2.21
10.51
3.78
4.70
1.00
1.79
7.72
4.17
1.71
f1 (ppm)

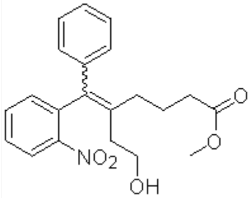

7s (mix of isomers 1:0.6)
Analysis: 13C NMR
Solvent: CDCl3
Field strength: 101 MHz

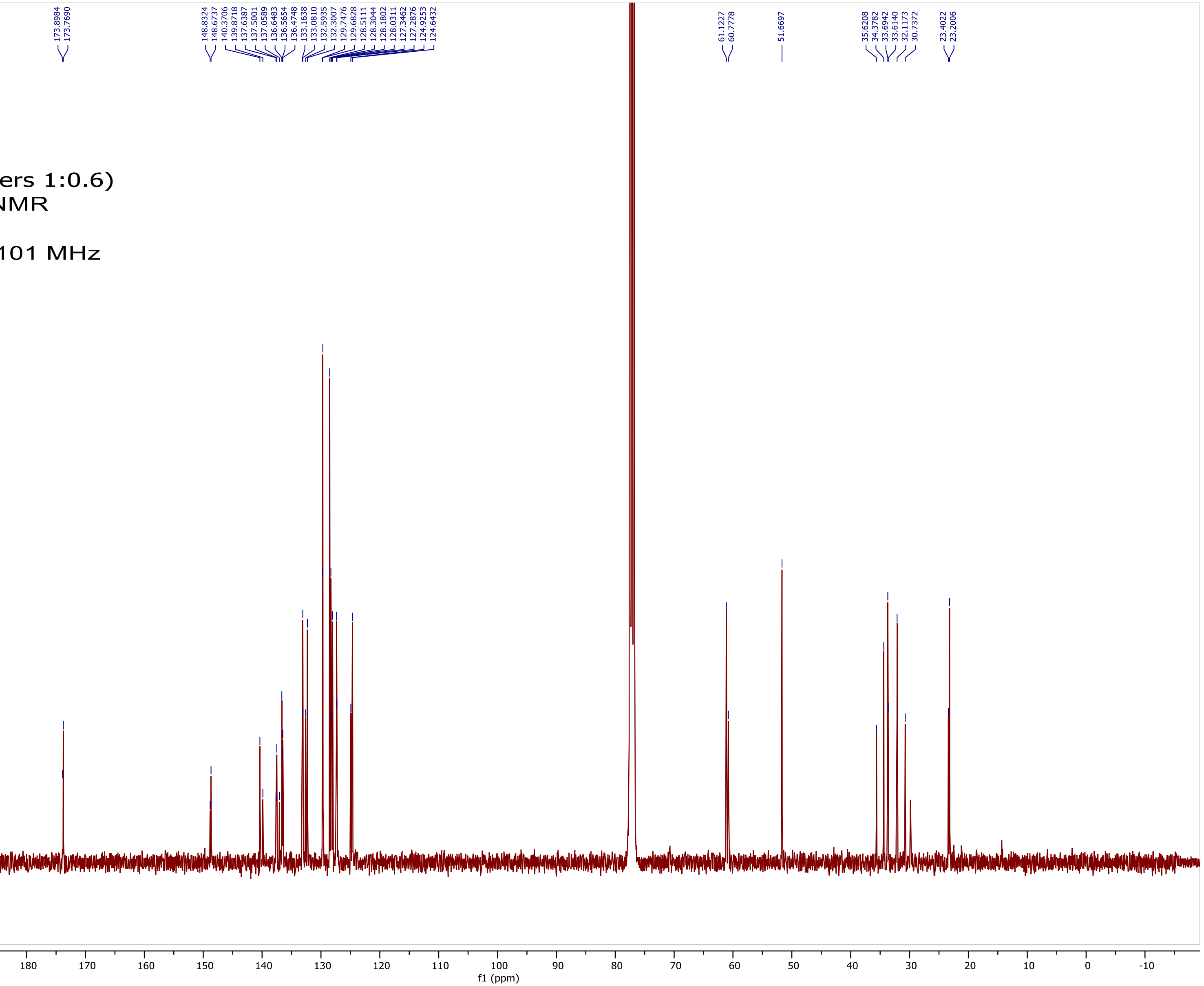

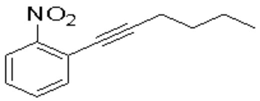

31

Analysis: 1H NMR

Solvent: CDCl3

Field strength: 400 MHz

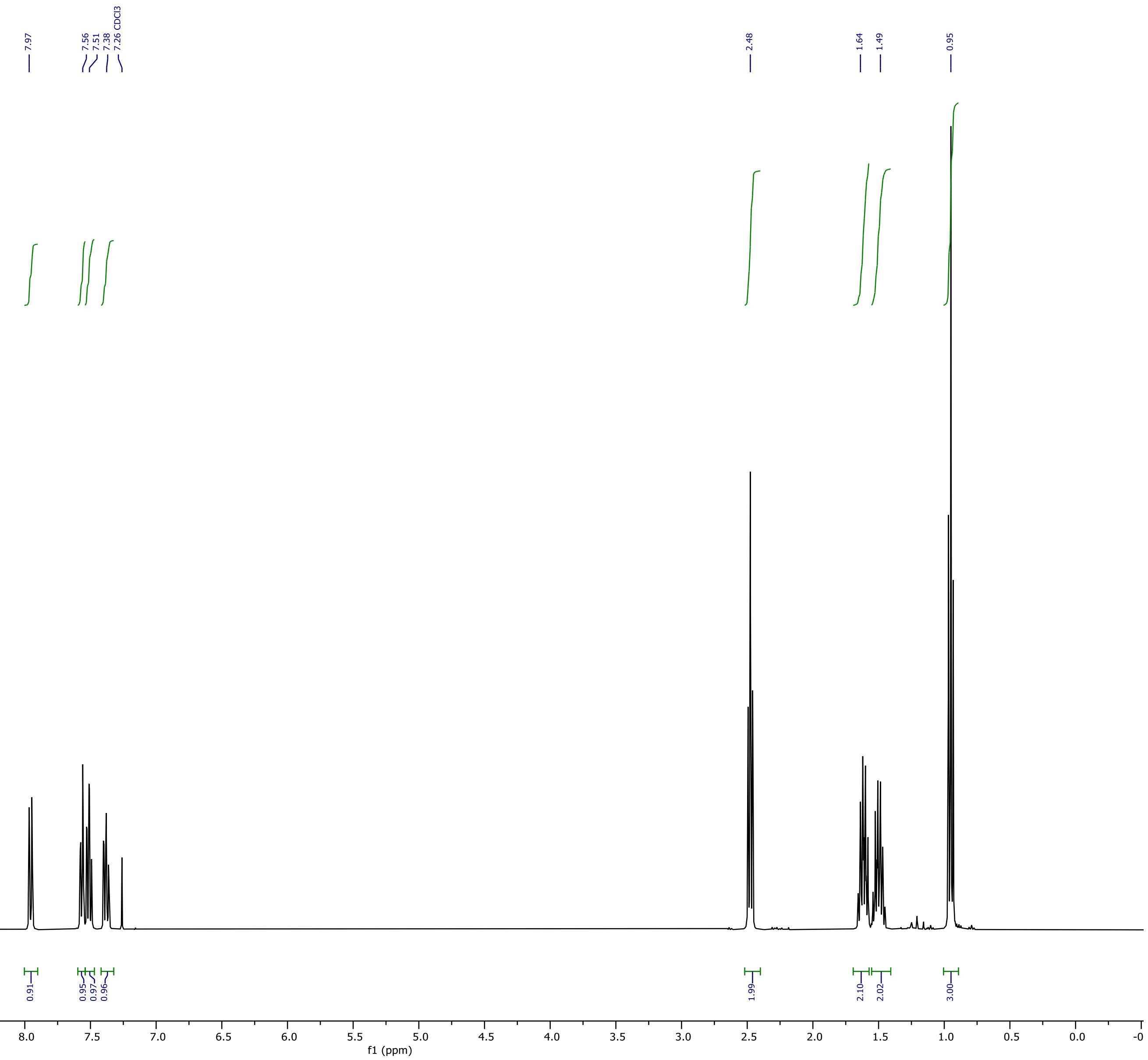

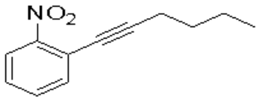

31
Analysis: 13C NMR
Solvent: CDCl3
Field strength: 101 MHz

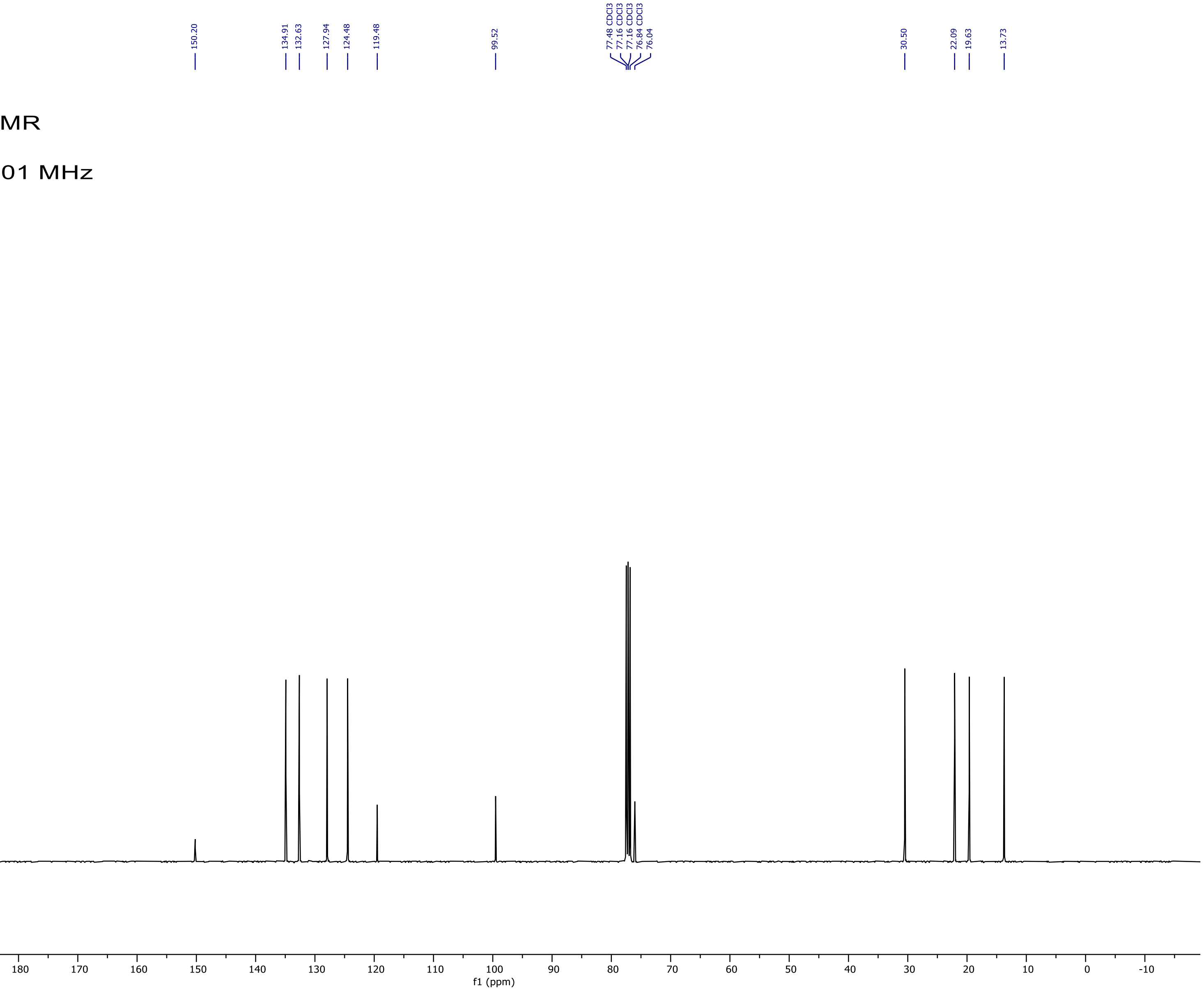

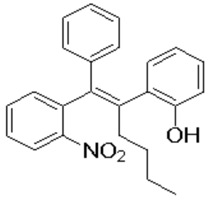

8a
Analysis: 1H NMR
Solvent: CDCl3
Field strength: 600 MHz

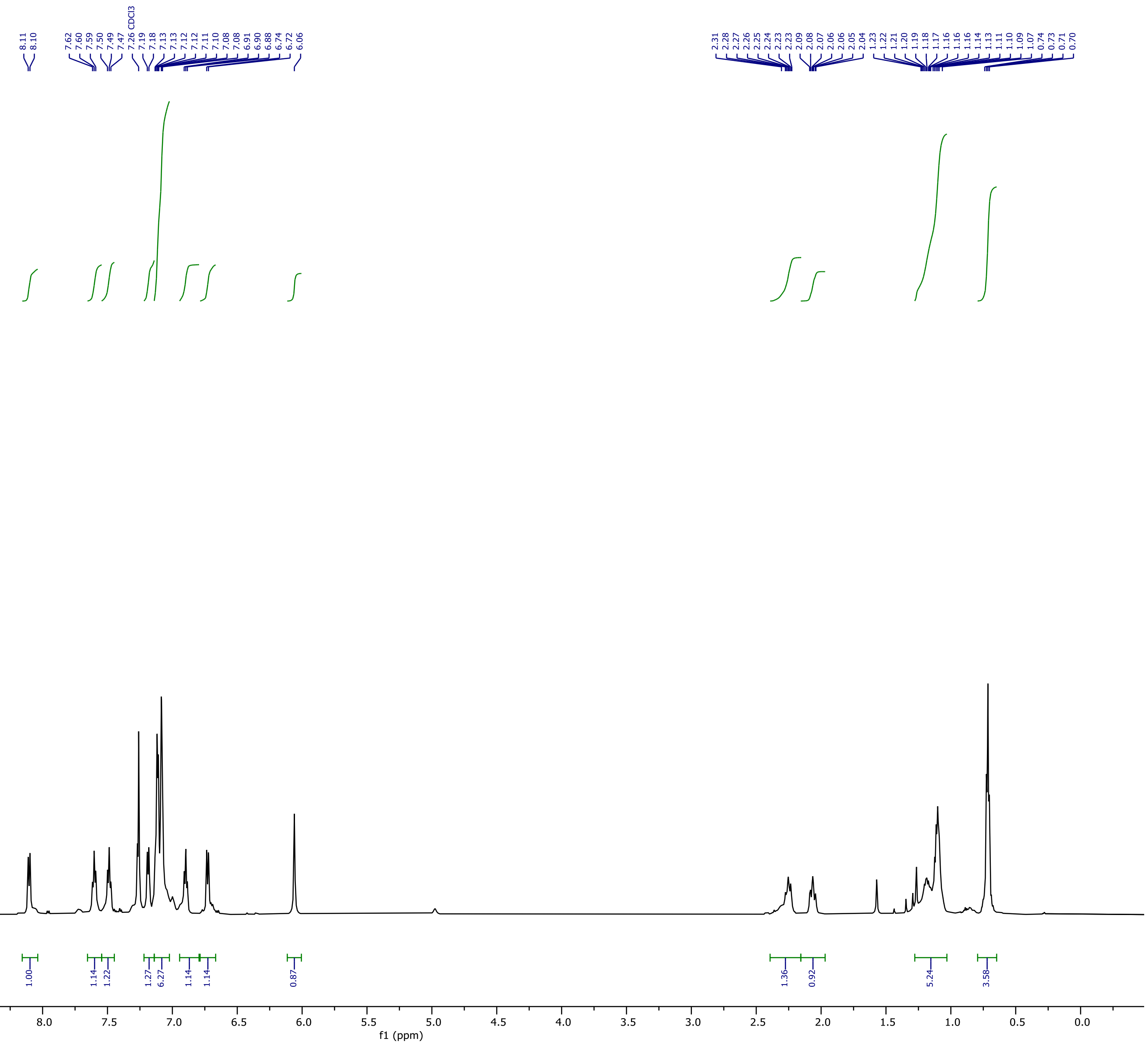

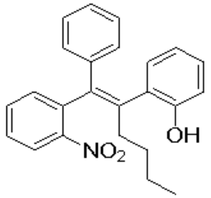

8a
Analysis: 13C NMR
Solvent: CDCl3
Field strength: 151 MHz

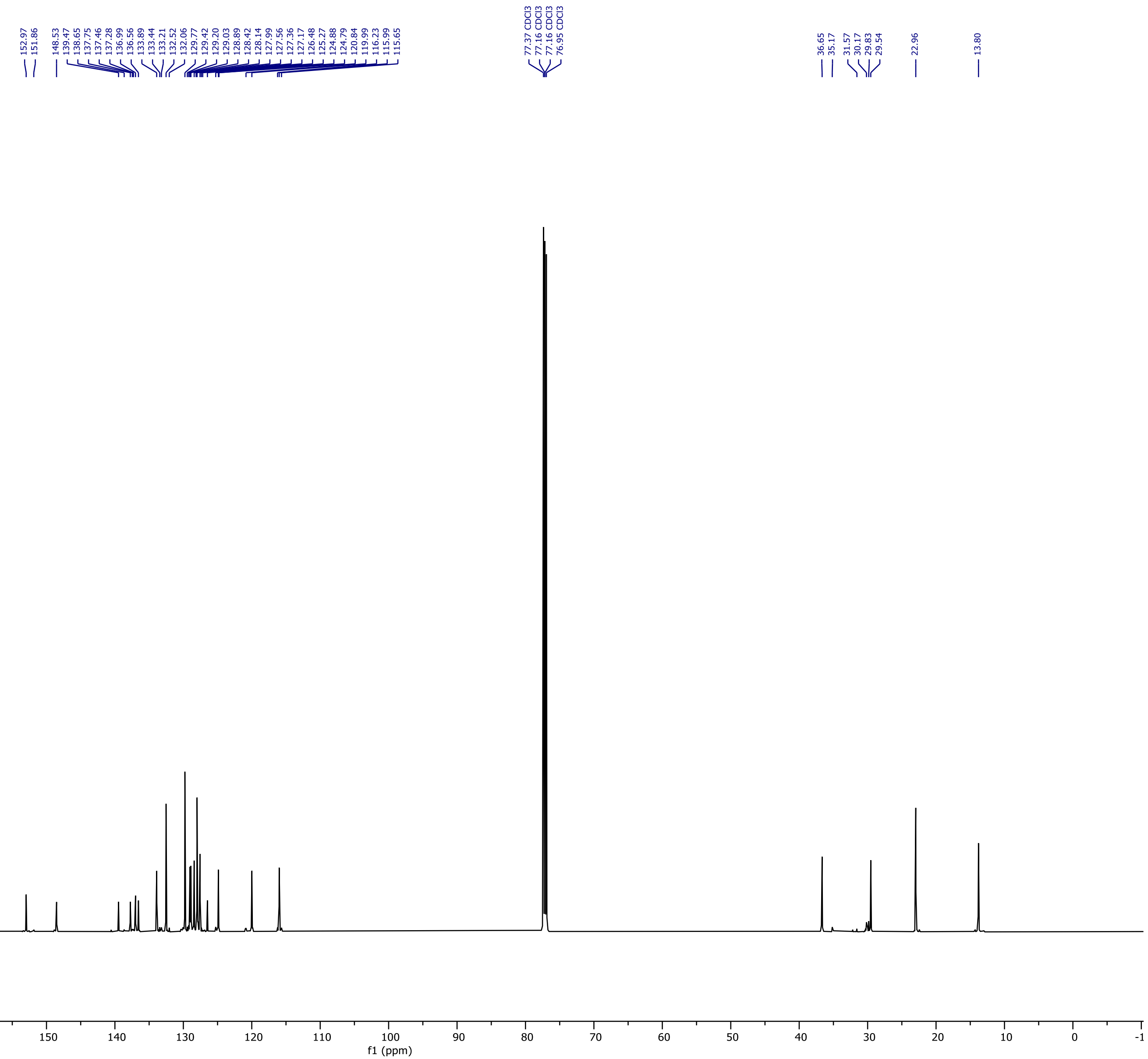

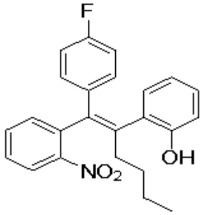

8b
Analysis: 1H NMR
Solvent: CDCl3
Field strength: 400 MHz

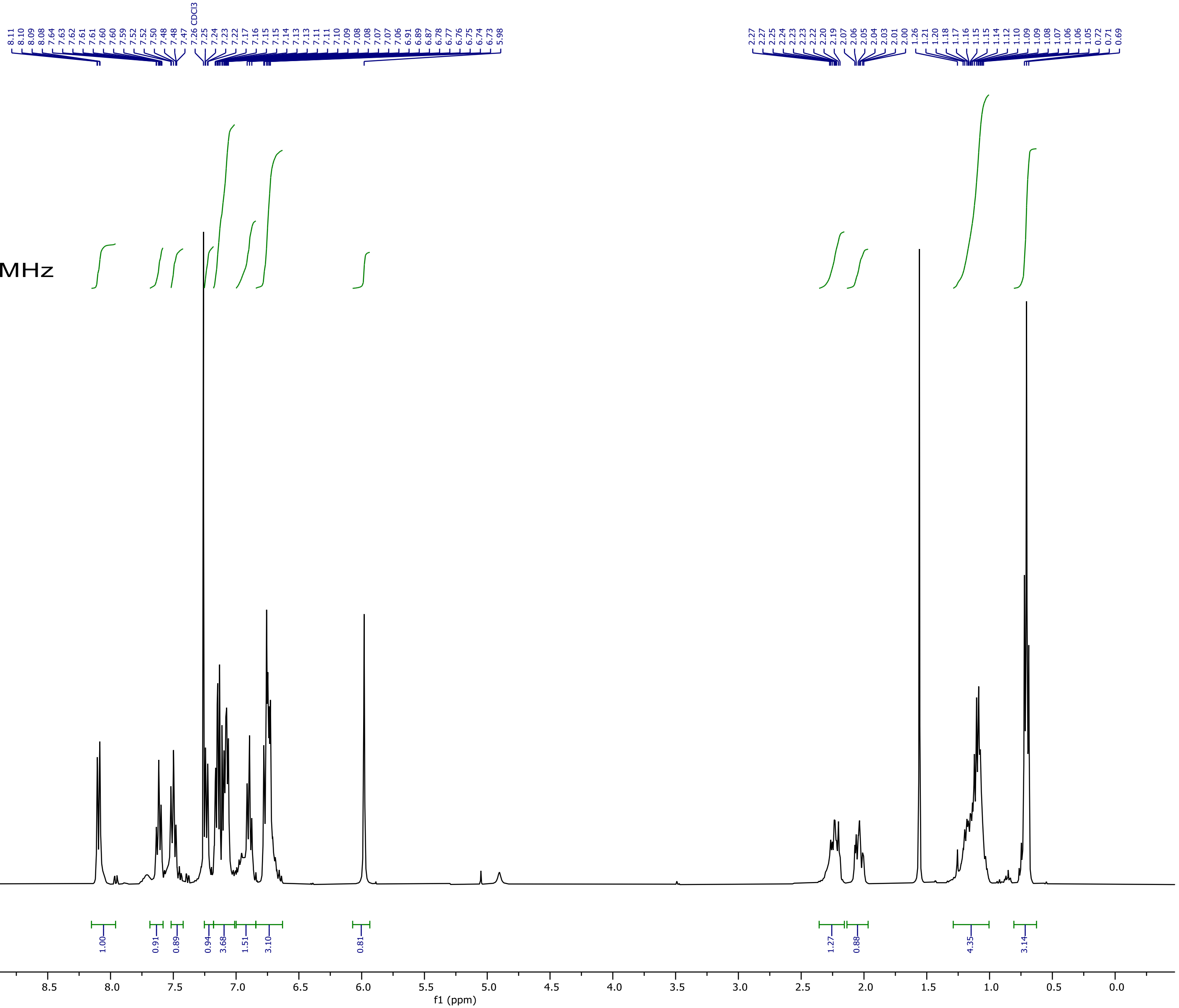

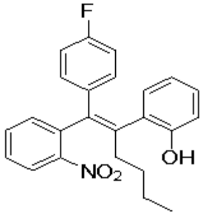

8b
Analysis: 13C NMR
Solvent: CDCl3
Field strength: 101 MHz

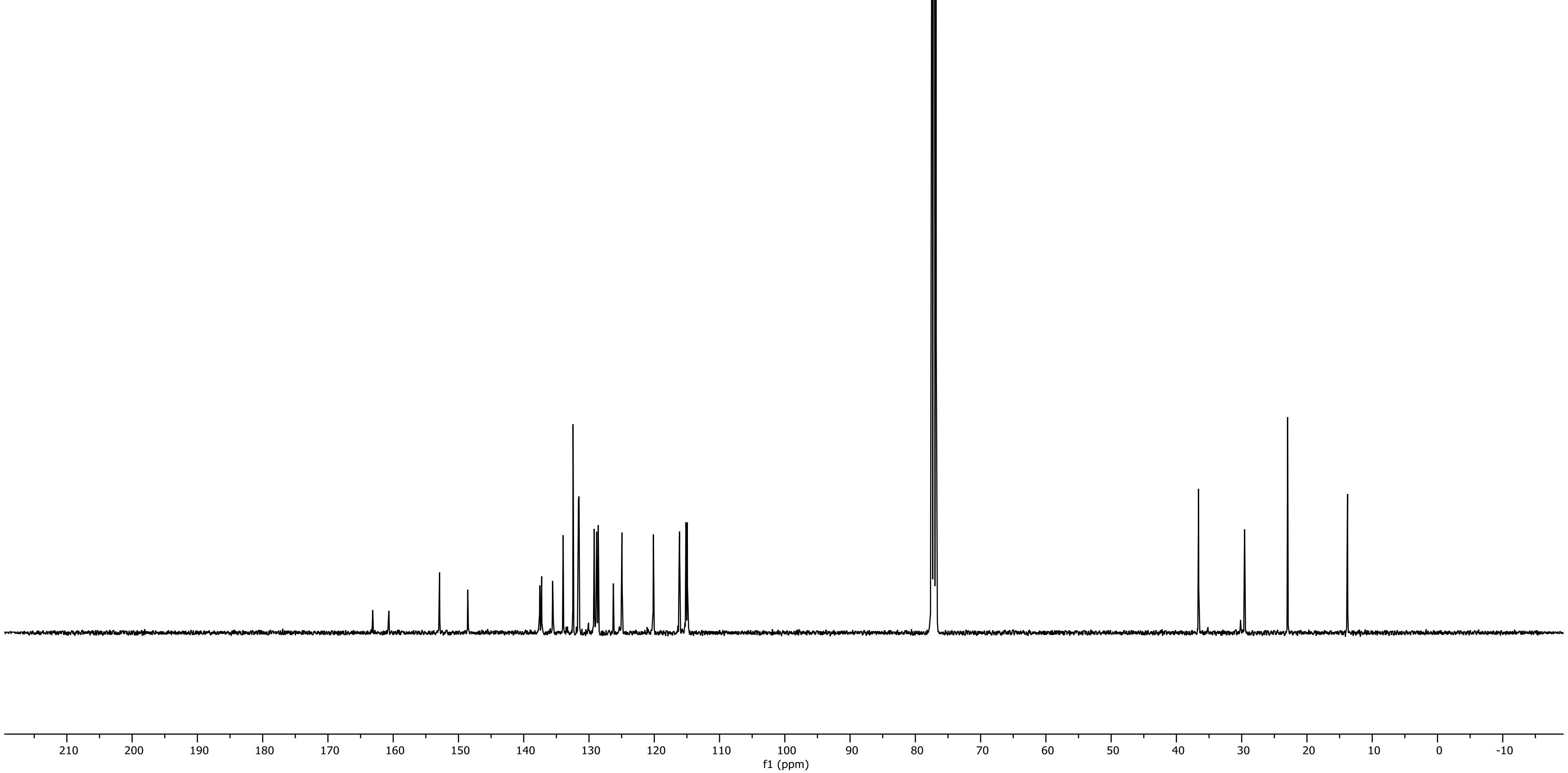

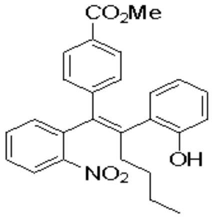

8c
Analysis: 1H NMR
Solvent: CDCl3
Field strength: 400 MHz

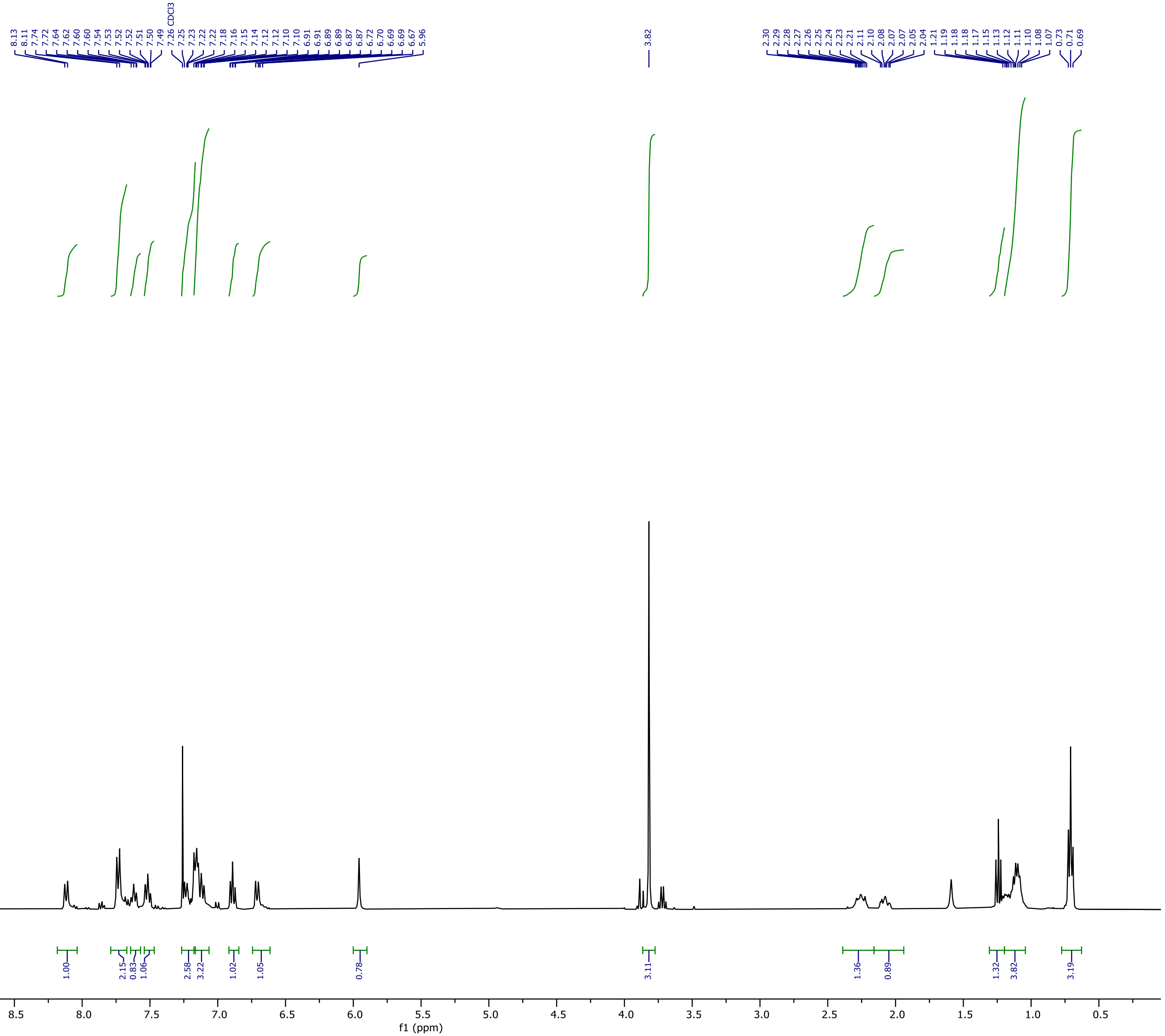

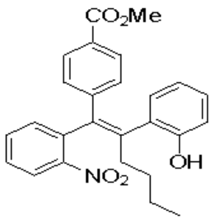

8c
Analysis: 13C NMR
Solvent: CDCl3
Field strength: 101 MHz

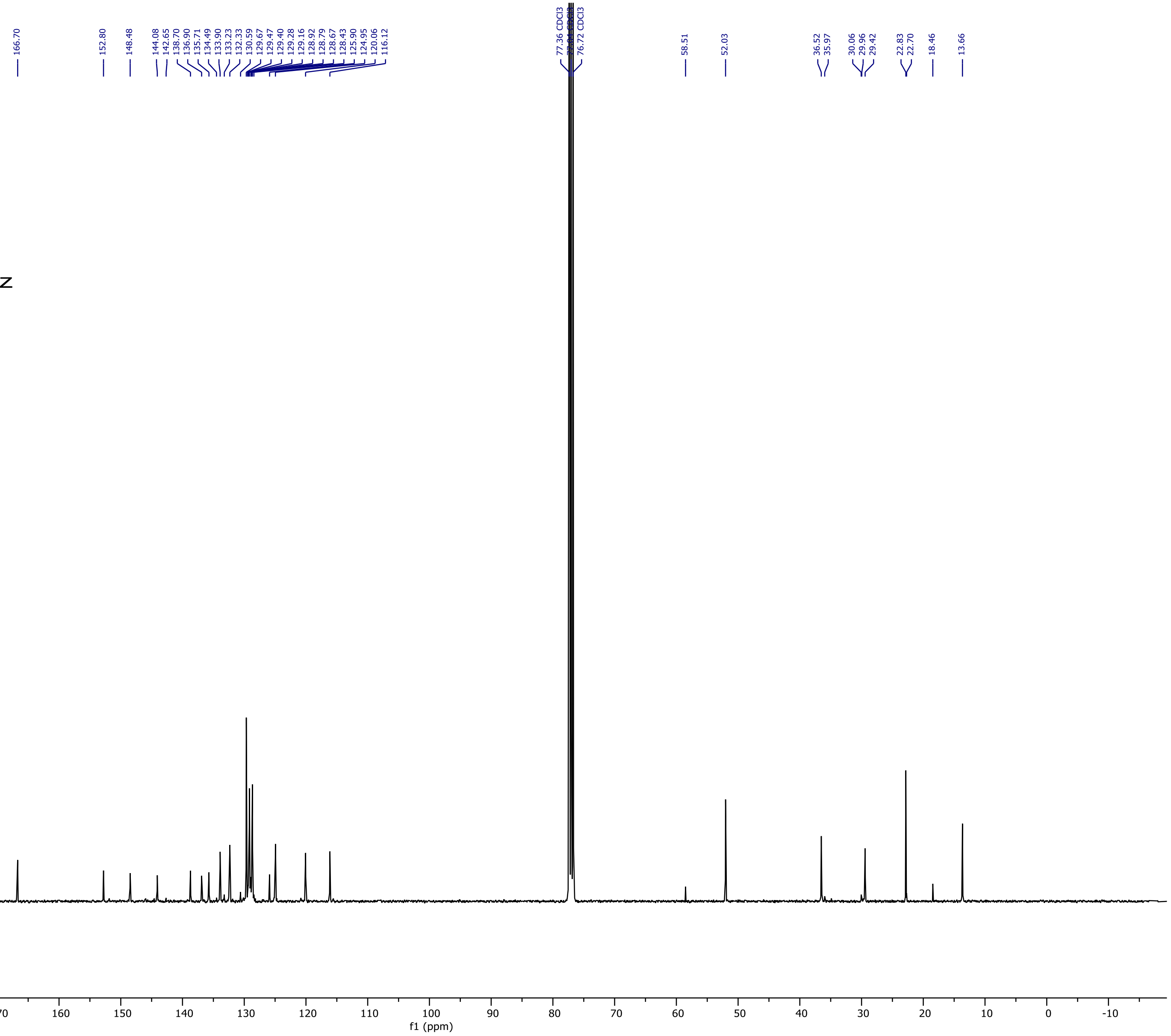

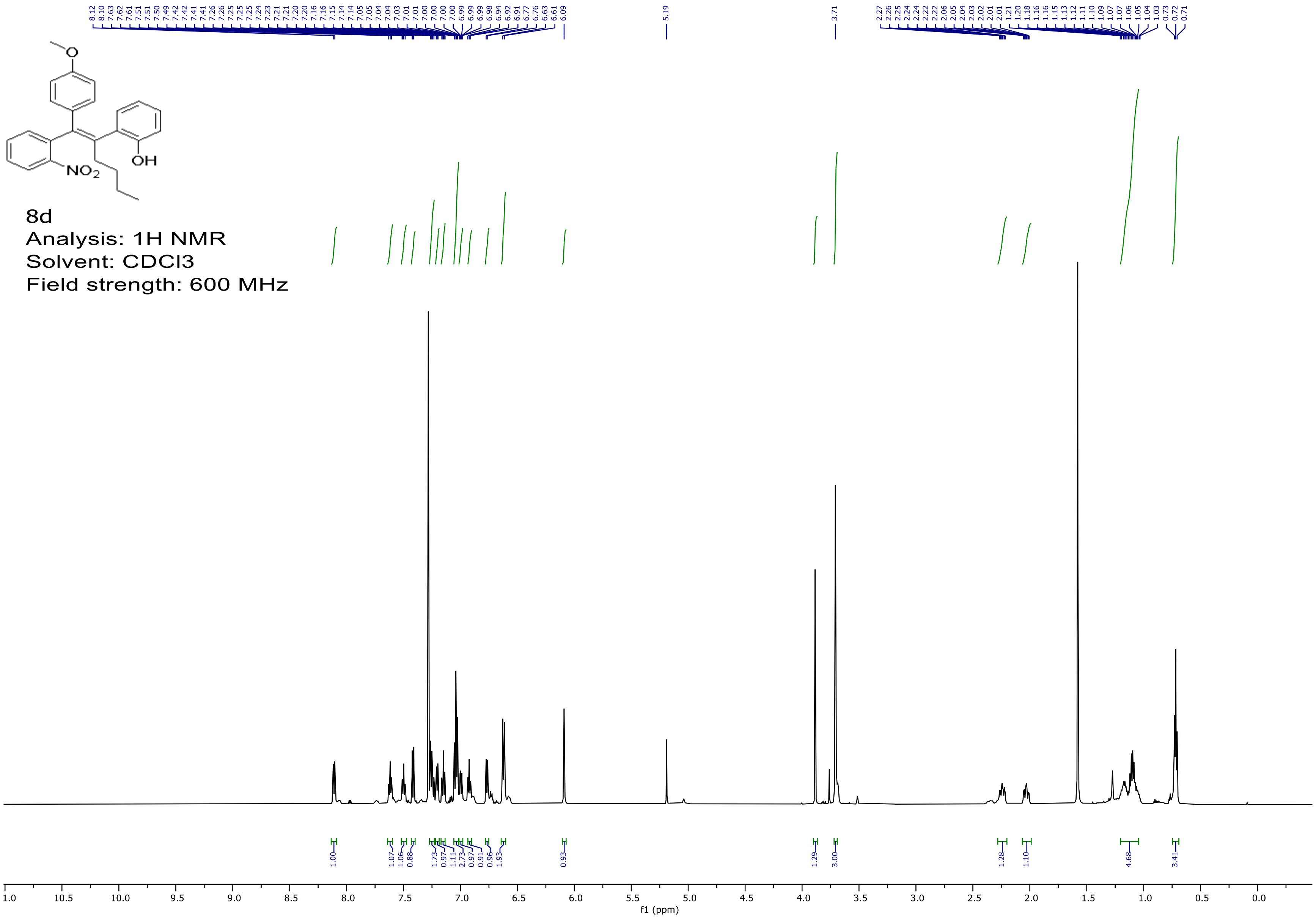

NO2
OH
8d
Analysis: 1H NMR
Solvent: CDCl3
Field strength: 600 MHz
f1 (ppm)

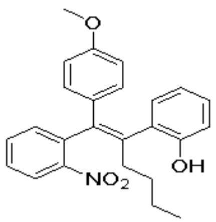

8d
Analysis: 13C NMR
Solvent: CDCl3
Field strength: 151 MHz

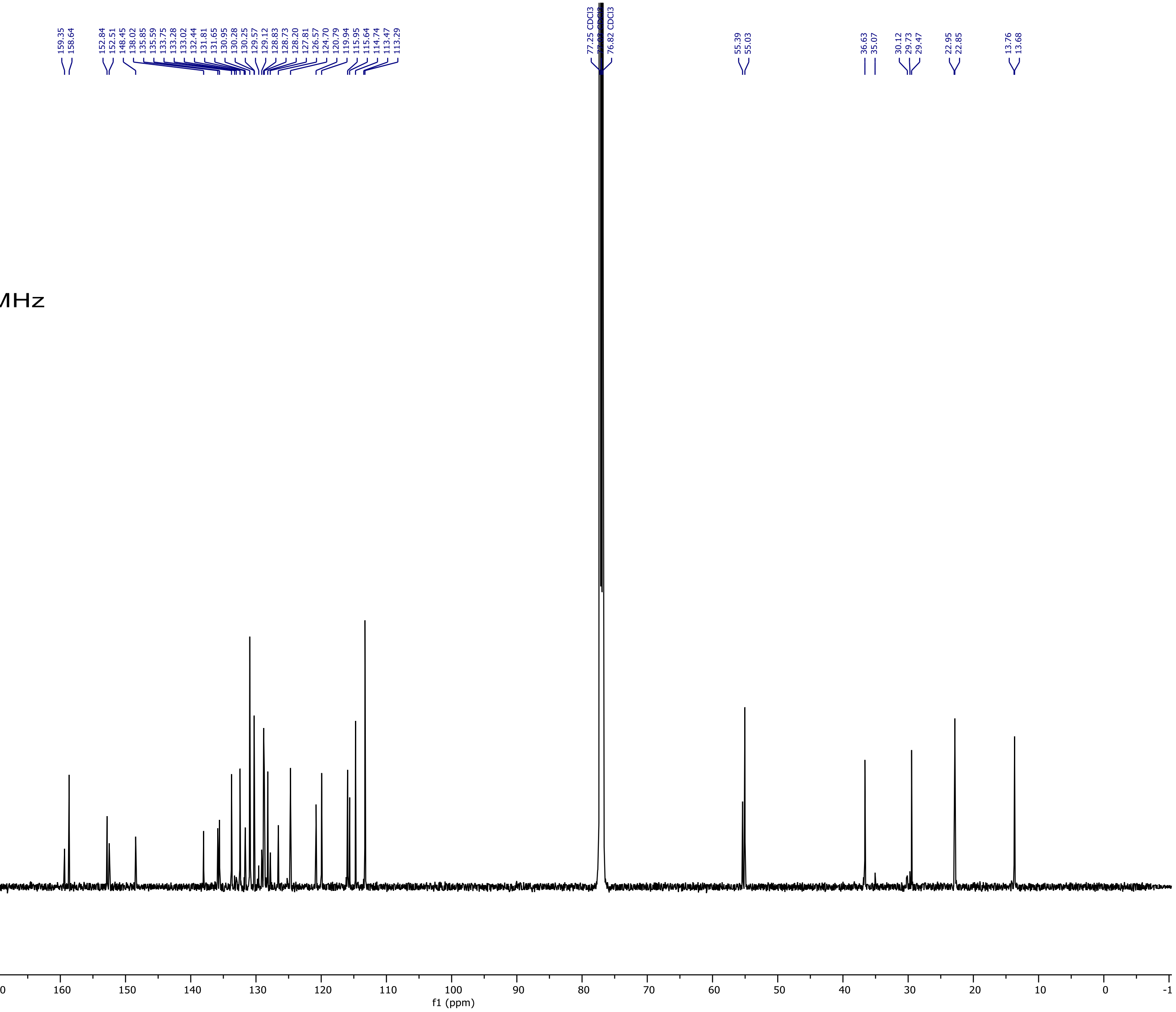

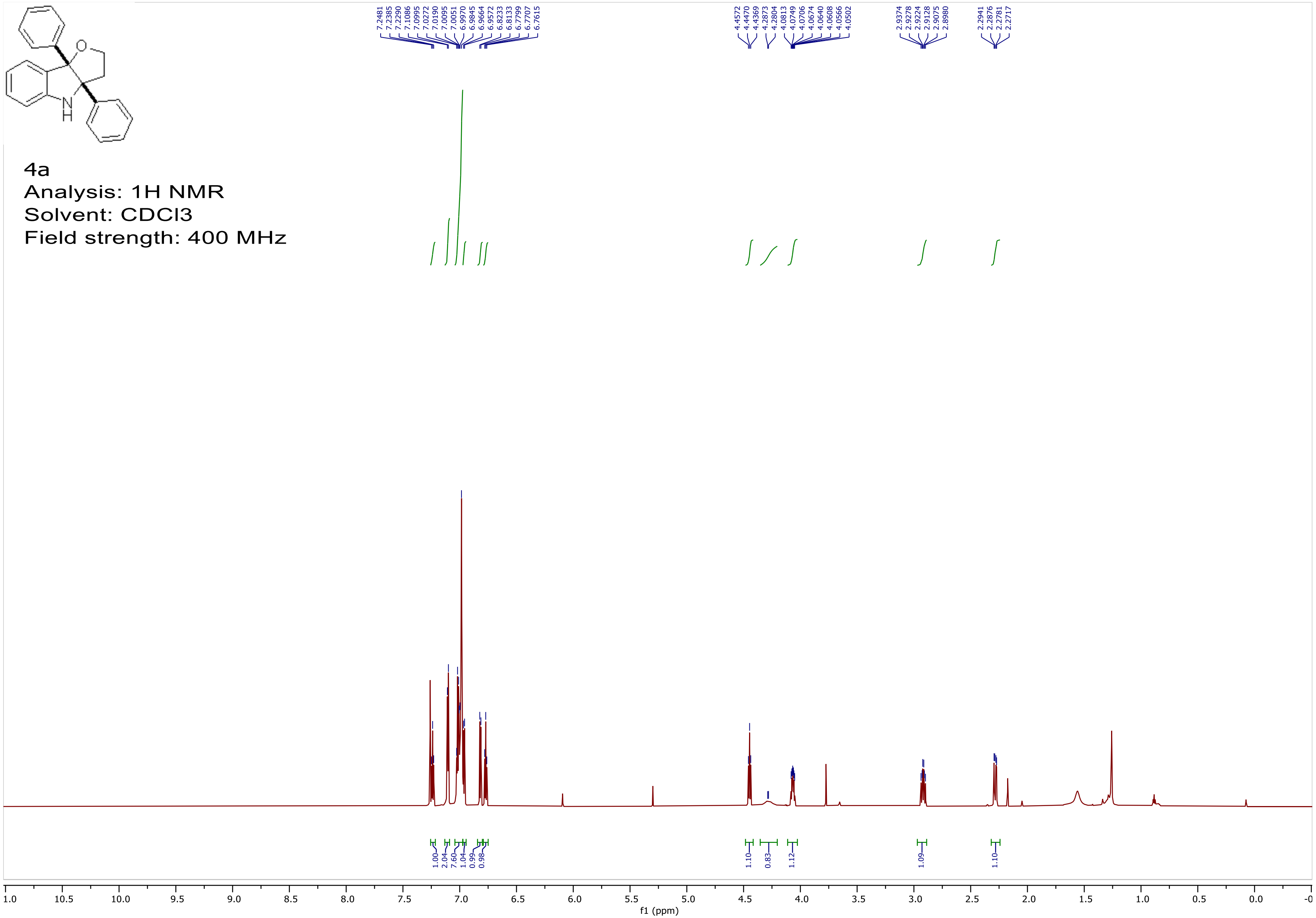

4a
Analysis: 1H NMR
Solvent: CDCl3
Field strength: 400 MHz
7.2481
7.2385
7.2290
7.1086
7.0995
7.0272
7.0190
7.0095
7.0051
6.9970
6.9845
6.9664
6.9572
6.8233
6.8133
6.7799
6.7707
6.7615
4.4572
4.4470
4.4369
4.2873
4.2804
4.0813
4.0749
4.0706
4.0674
4.0640
4.0608
4.0566
4.0502
2.9374
2.9278
2.9224
2.9128
2.9075
2.8980
2.2941
2.2876
2.2781
2.2717
1.00
2.04
7.60
1.04
0.99
0.98
1.10
0.83
1.12
1.09
1.10
f1 (ppm)

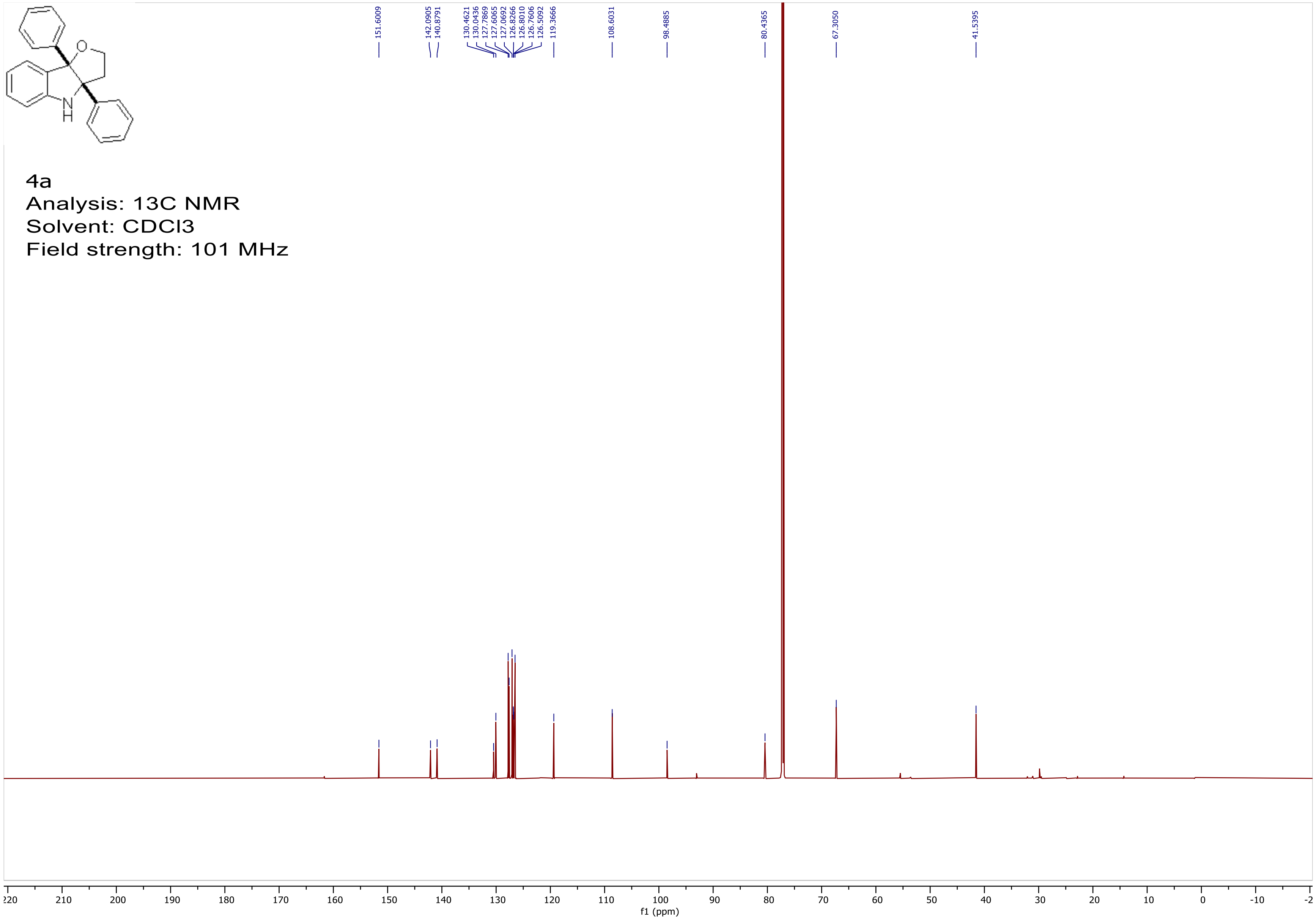

4a
Analysis: 13C NMR
Solvent: CDCl3
Field strength: 101 MHz
151.6009
142.0905
140.8791
130.4621
130.0436
127.7869
127.6065
127.0692
126.8266
126.8010
126.7606
126.5092
119.3666
108.6031
98.4885
80.4365
67.3050
41.5395
f1 (ppm)

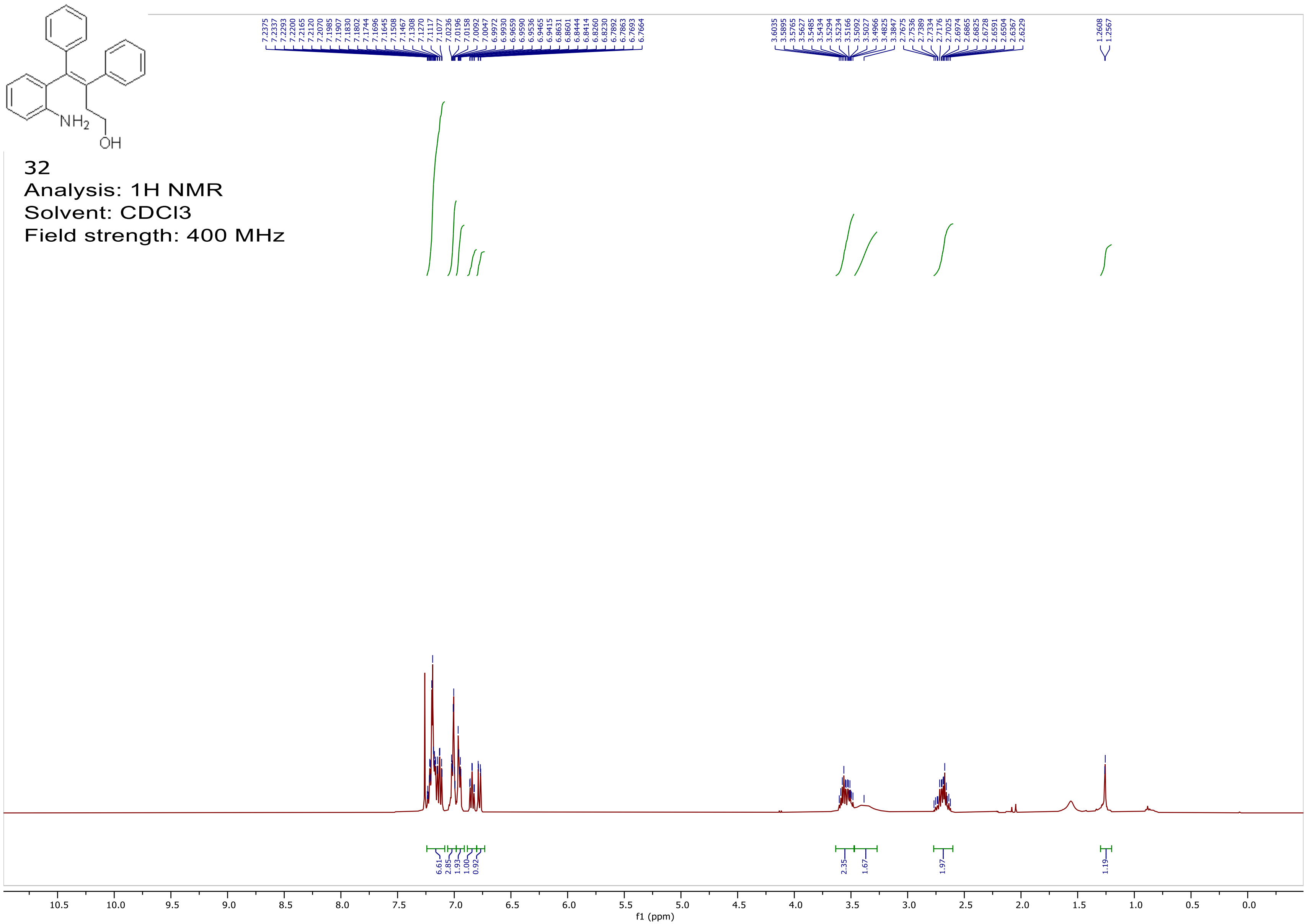
32
Analysis: 1H NMR
Solvent: CDCl3
Field strength: 400 MHz
NH2
OH
f1 (ppm)

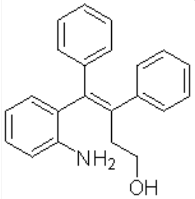

32
Analysis: 13C NMR
Solvent: CDCl3
Field strength: 101 MHz

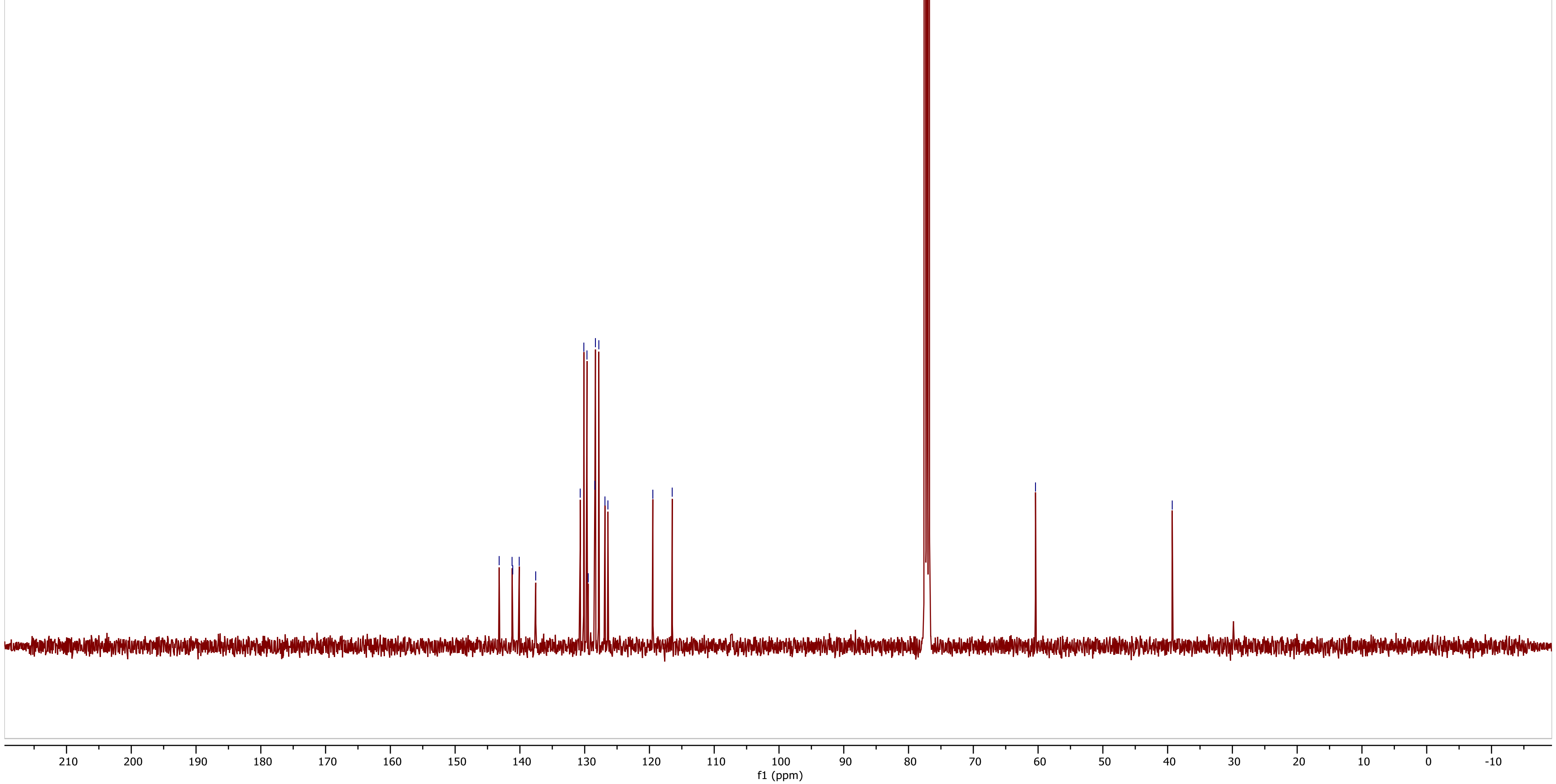

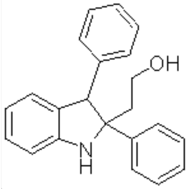

14
Analysis: 1H NMR
Solvent: CDCl3
Field strength: 400 MHz

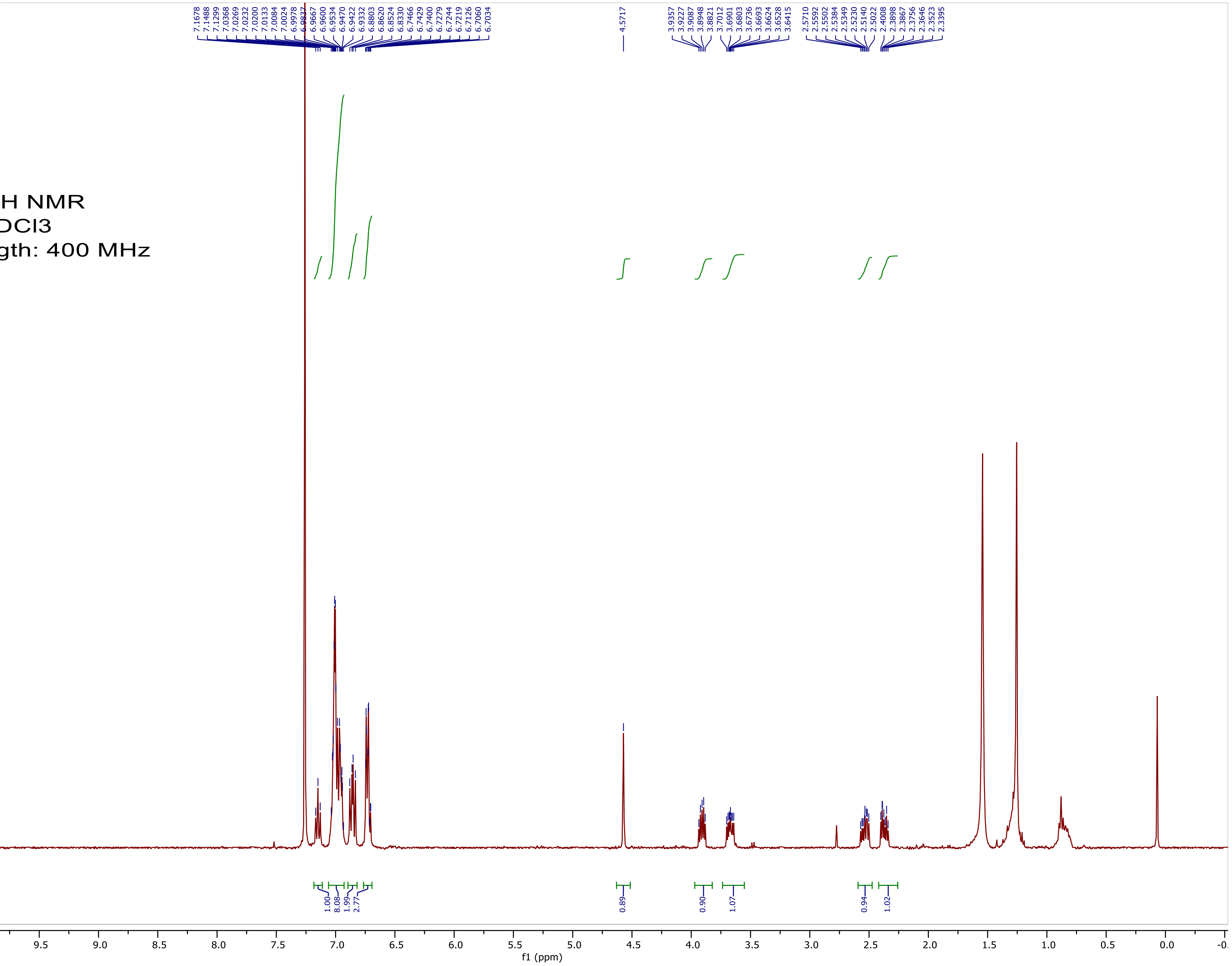

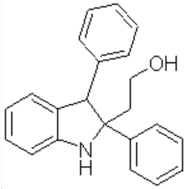

14
Analysis: 13C NMR
Solvent: CDCl3
Field strength: 101 MHz

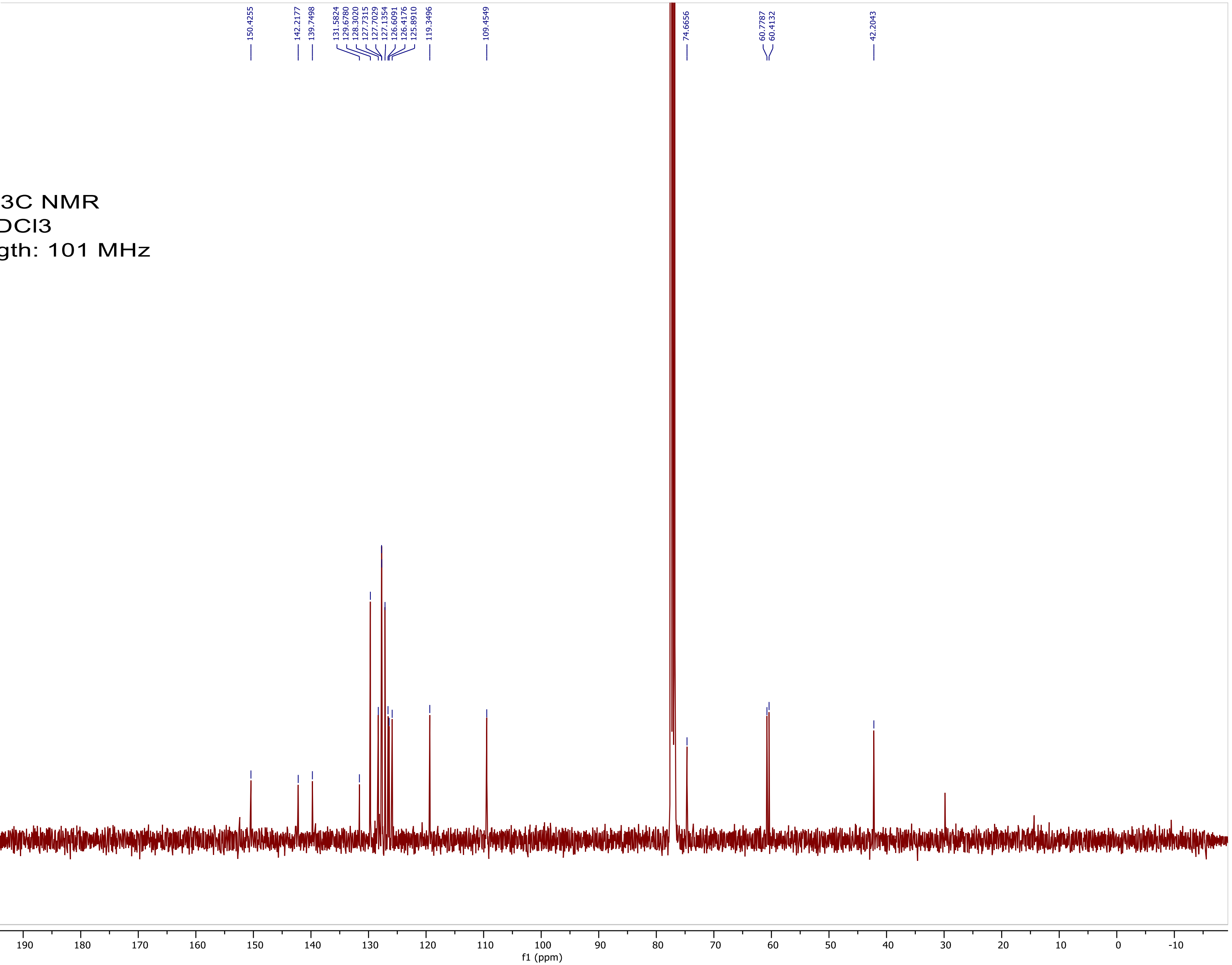

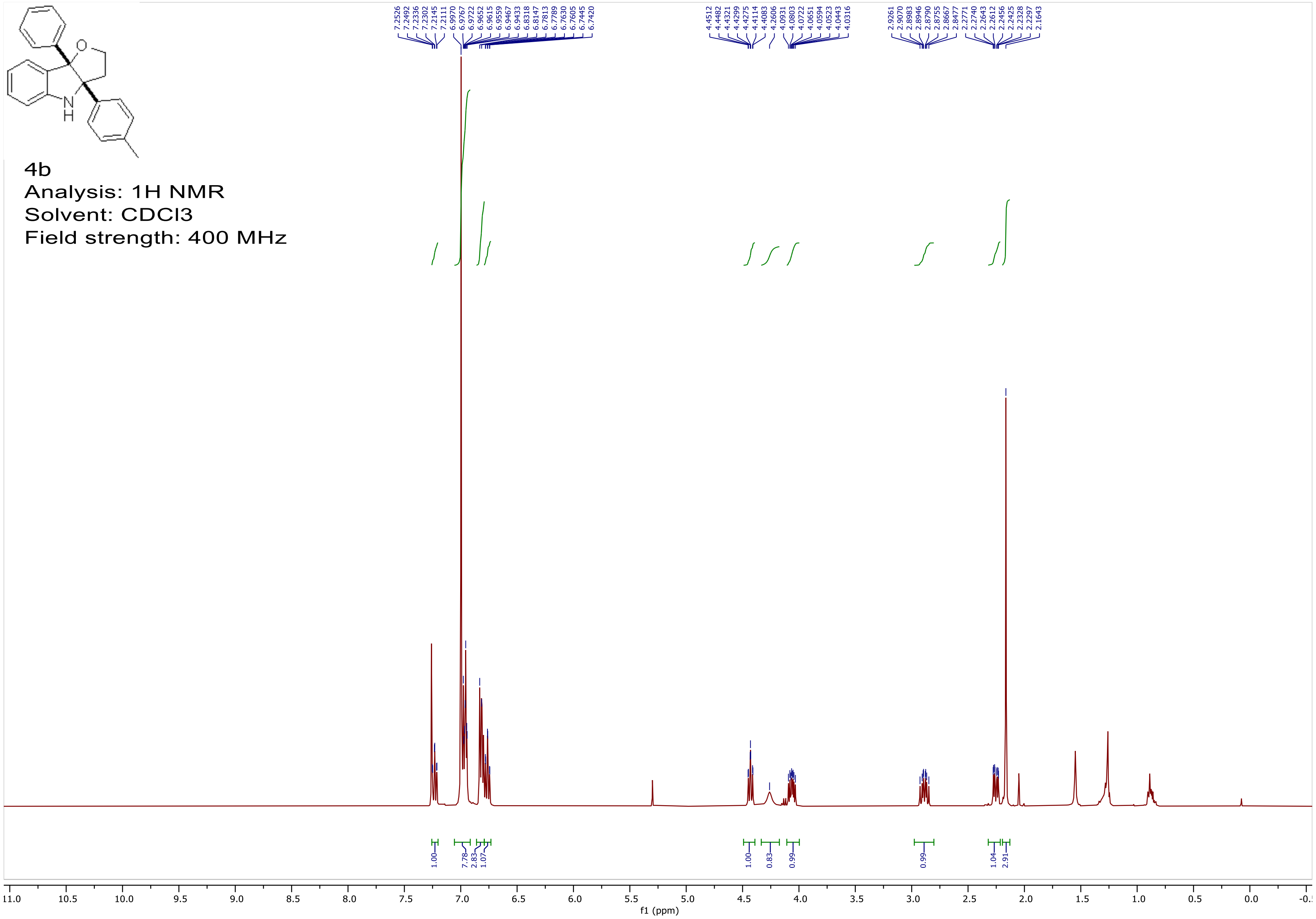
4b
Analysis: 1H NMR
Solvent: CDCl3
Field strength: 400 MHz
f1 (ppm)

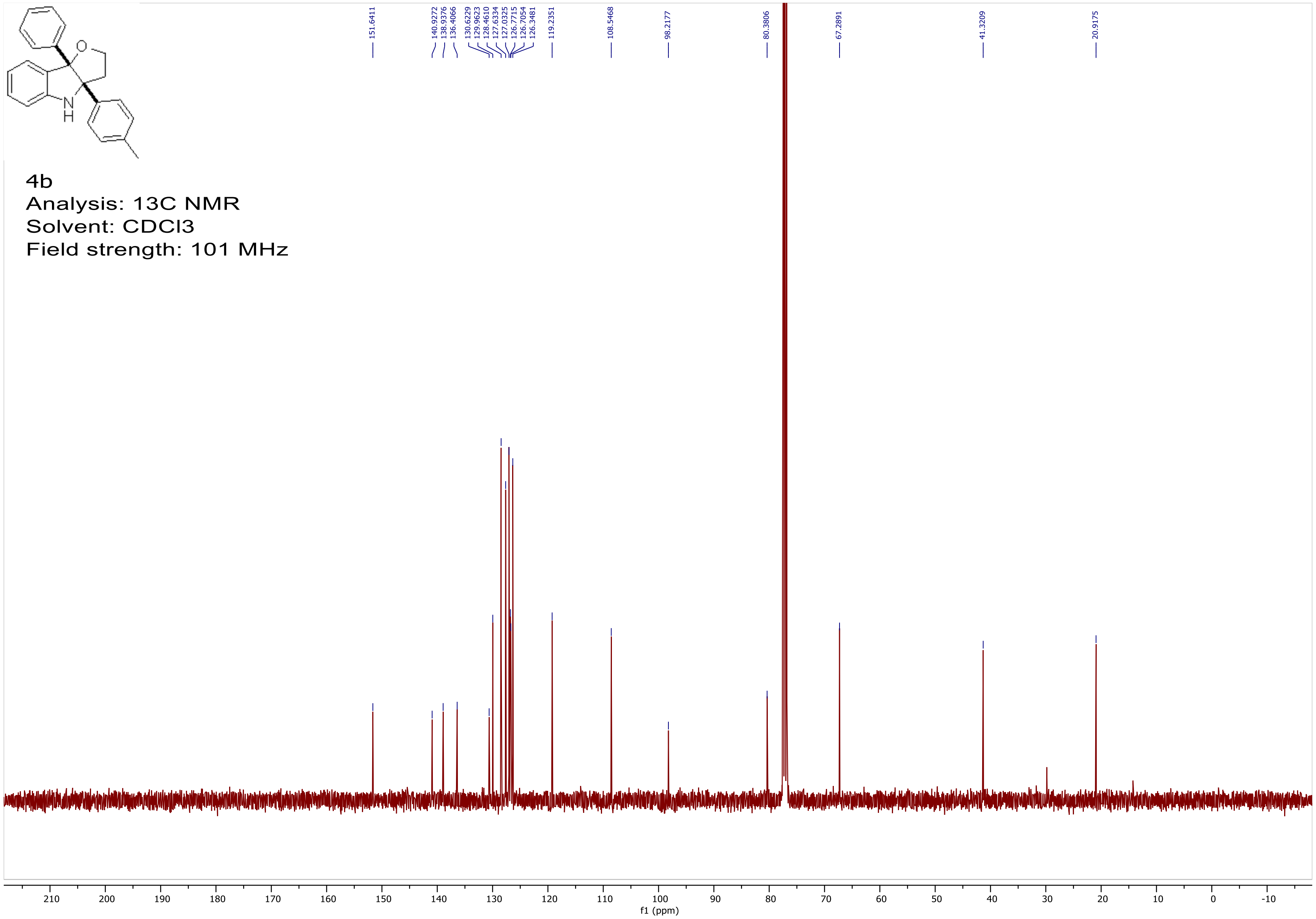

4b
Analysis: 13C NMR
Solvent: CDCl3
Field strength: 101 MHz
151.6411
140.9272
138.9376
136.4066
130.6229
129.9623
128.4610
127.6334
127.0325
126.7715
126.7054
126.3481
119.2351
108.5468
98.2177
80.3806
67.2891
41.3209
20.9175
210 200 190 180 170 160 150 140 130 120 110 100 90 80 70 60 50 40 30 20 10 0 -10
f1 (ppm)

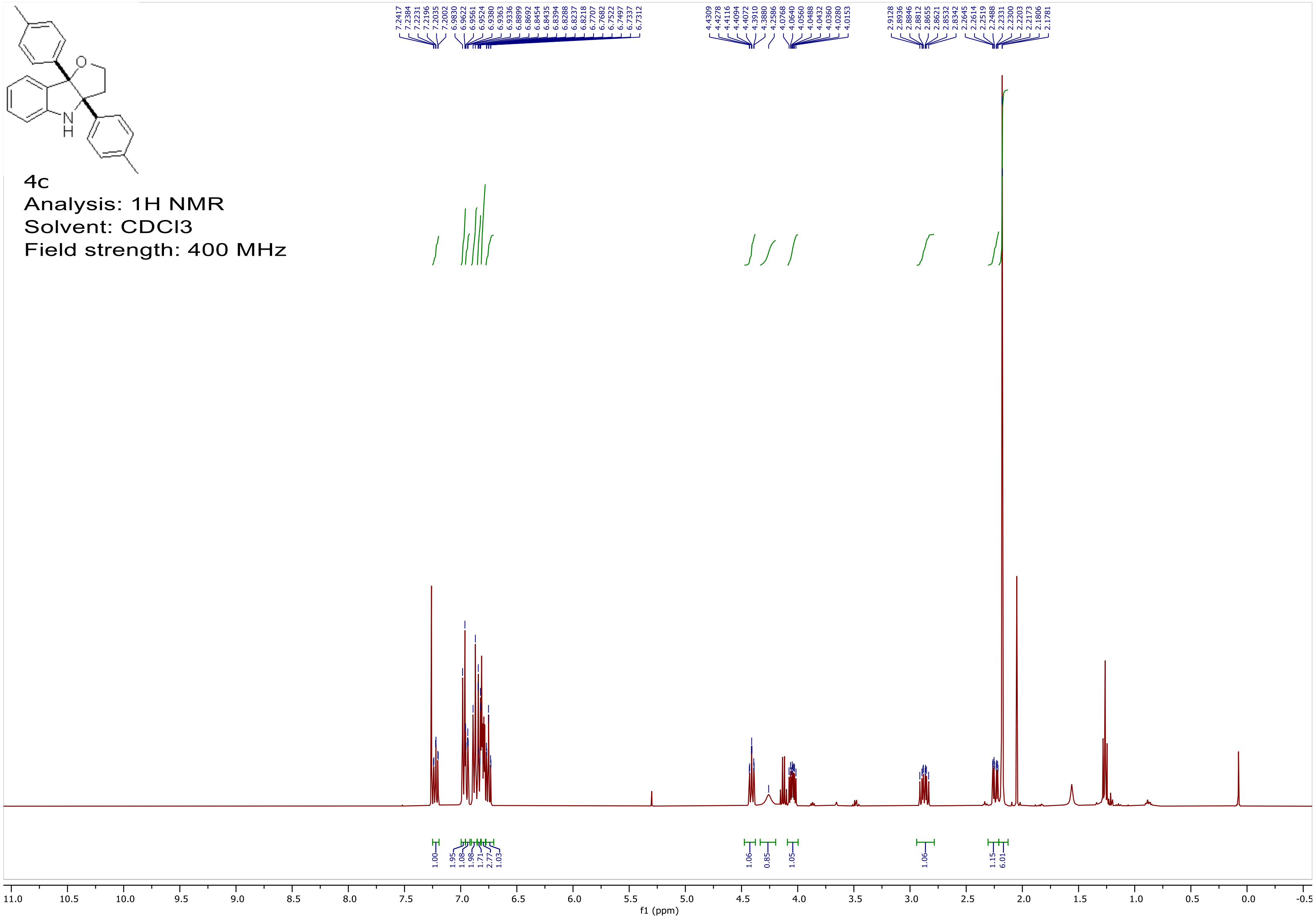
4c
Analysis: 1H NMR
Solvent: CDCl3
Field strength: 400 MHz
f1 (ppm)

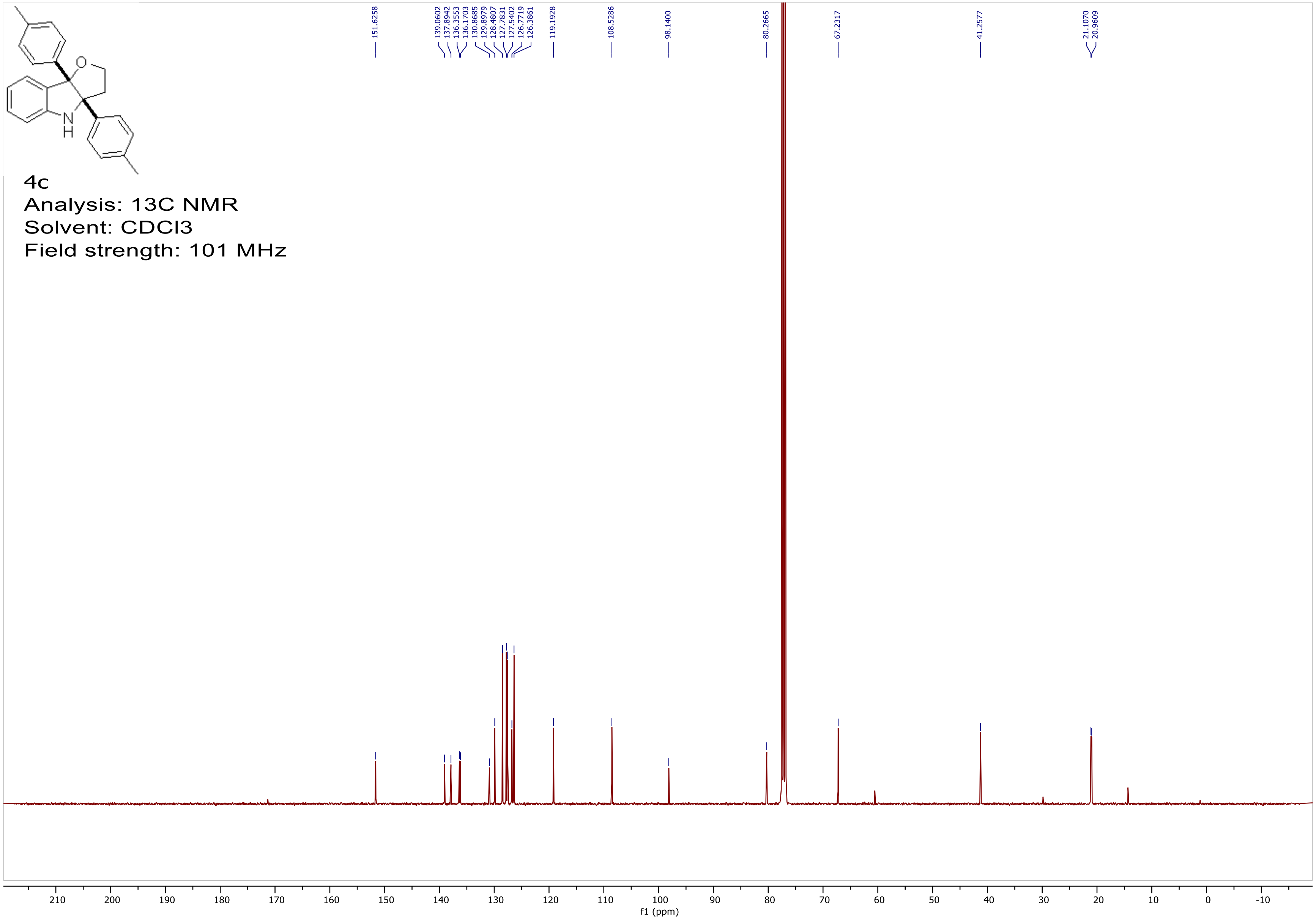
4c
Analysis: 13C NMR
Solvent: CDCl3
Field strength: 101 MHz
151.6258
139.0602
137.8942
136.3553
136.1703
130.8685
129.8979
128.4807
127.7831
127.5402
126.7719
126.3861
119.1928
108.5286
98.1400
80.2665
67.2317
41.2577
21.1070
20.9609
f1 (ppm)

4d
Analysis: 1H NMR
Solvent: CDCl3
Field strength: 400 MHz

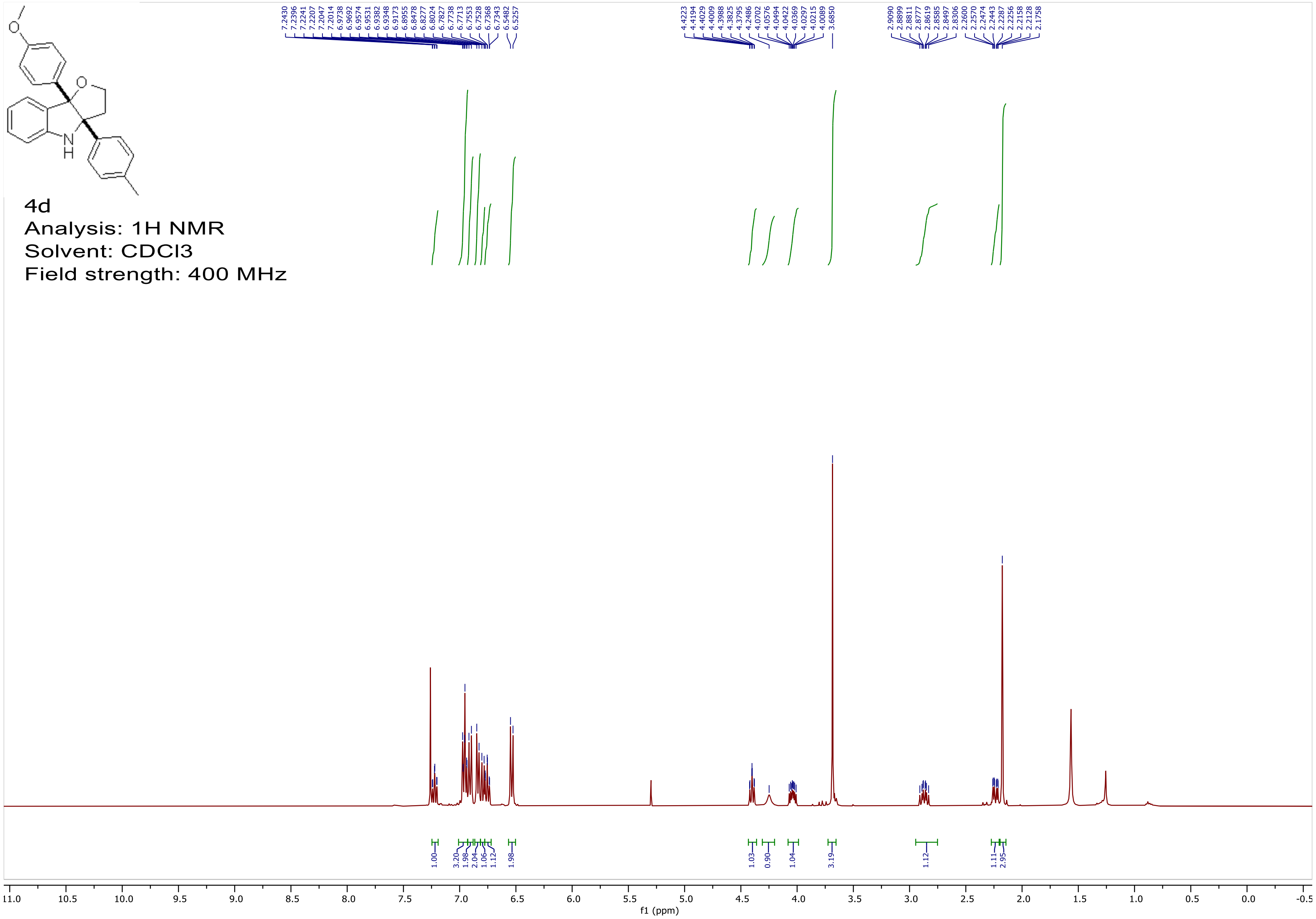

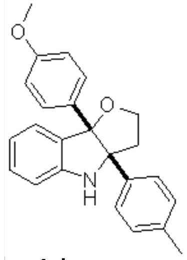

4d
Analysis: 13C NMR
Solvent: CDCl3
Field strength: 101 MHz

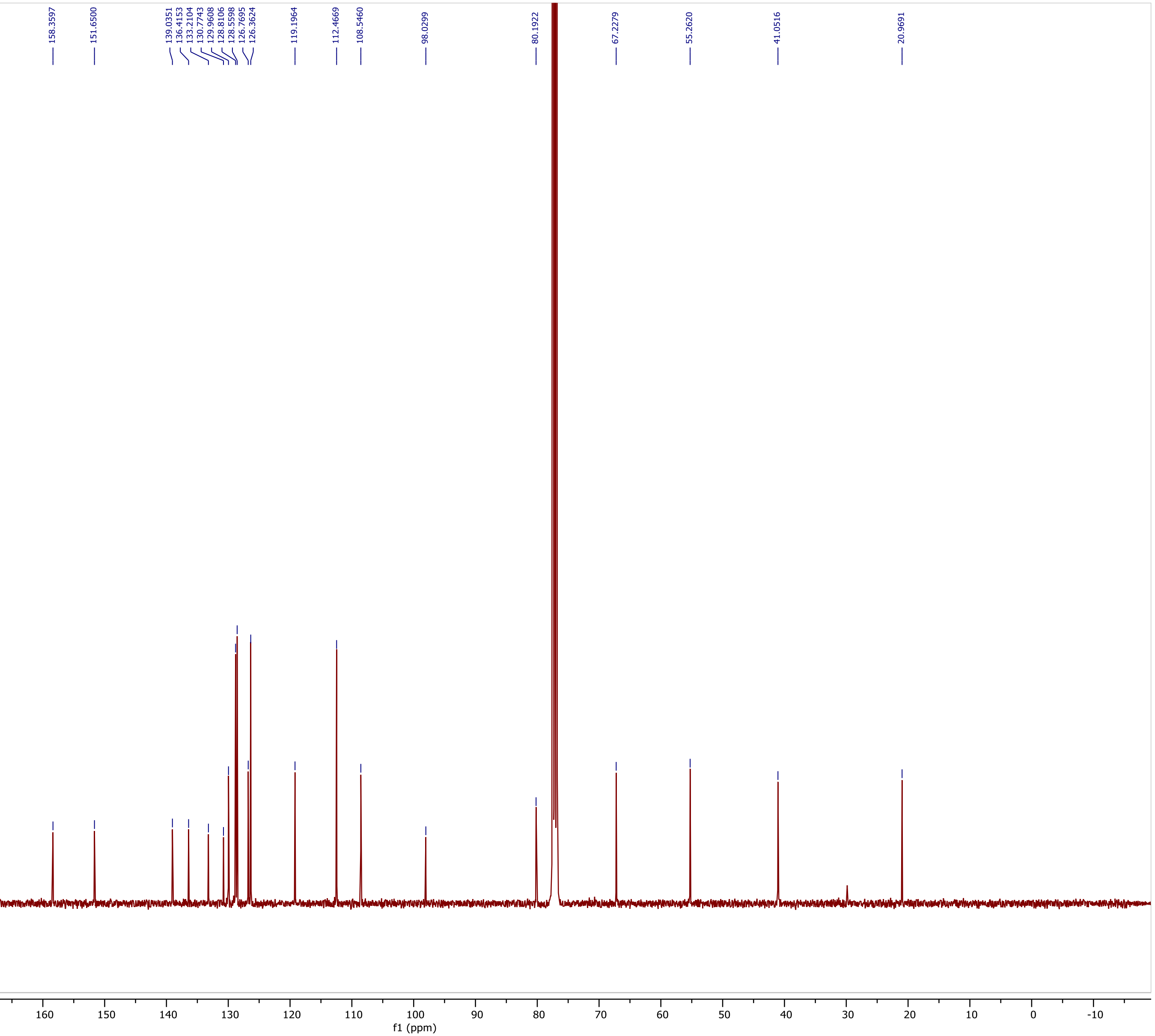

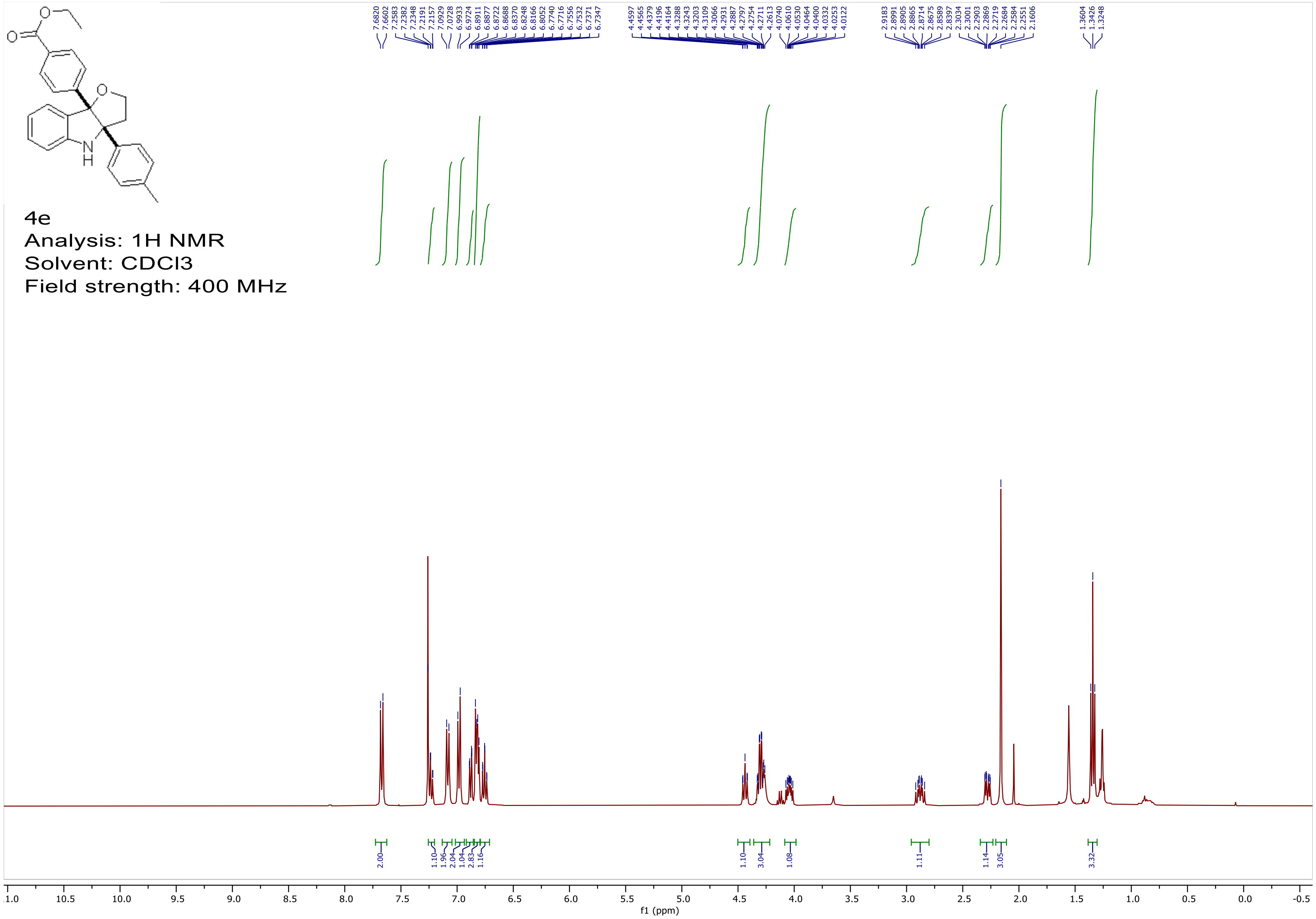
4e
Analysis: 1H NMR
Solvent: CDCl3
Field strength: 400 MHz
f1 (ppm)

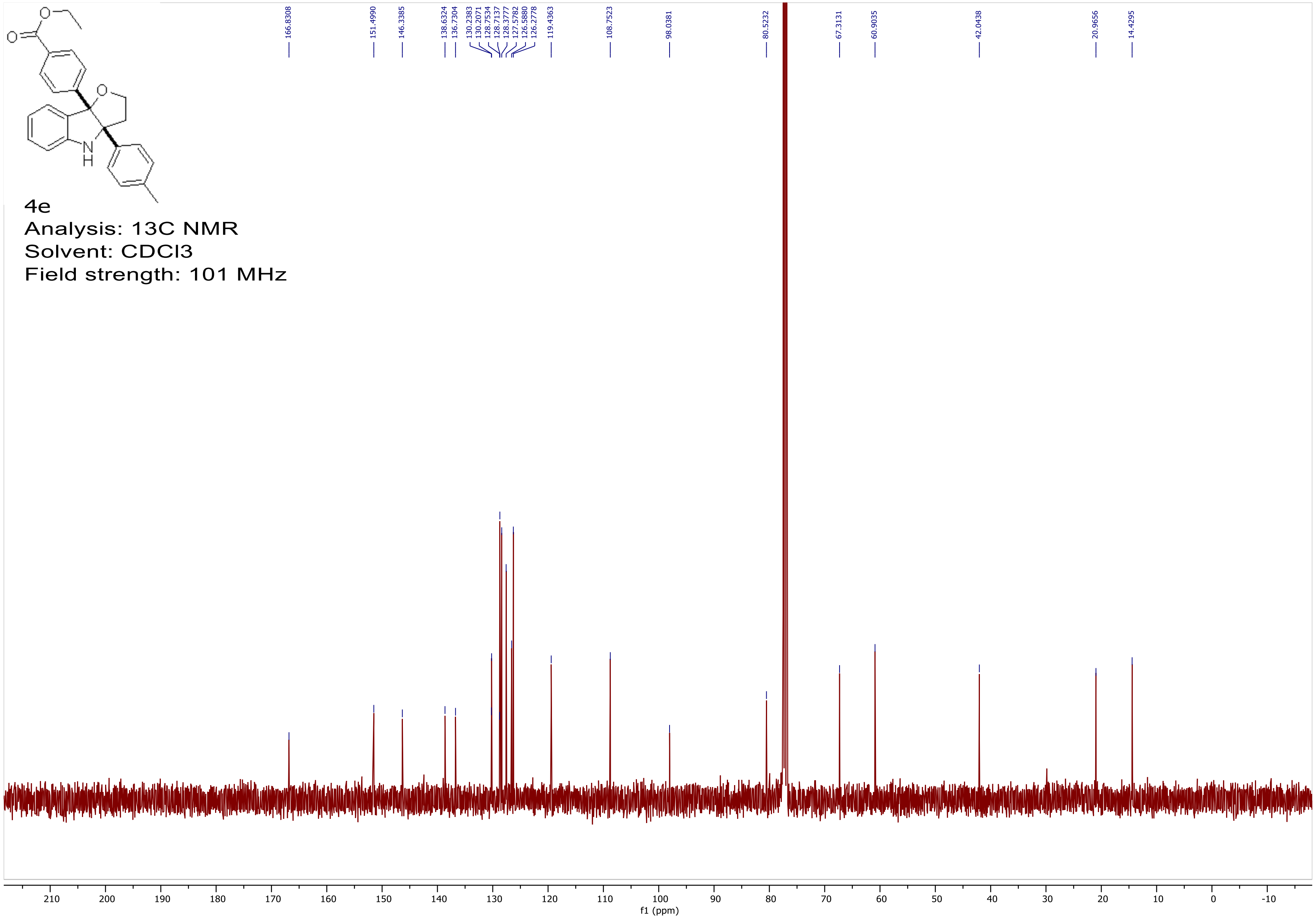
4e
Analysis: 13C NMR
Solvent: CDCl3
Field strength: 101 MHz
166.8308
151.4990
146.3385
138.6324
136.7304
130.2383
130.2071
128.7534
128.7137
128.3777
127.5782
126.5880
126.2778
119.4363
108.7523
98.0381
80.5232
67.3131
60.9035
42.0438
20.9656
14.4295
f1 (ppm)

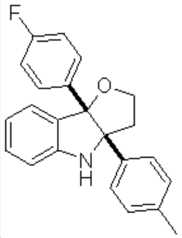

4f
Analysis: 1H NMR
Solvent: CDCl3
Field strength: 400 MHz

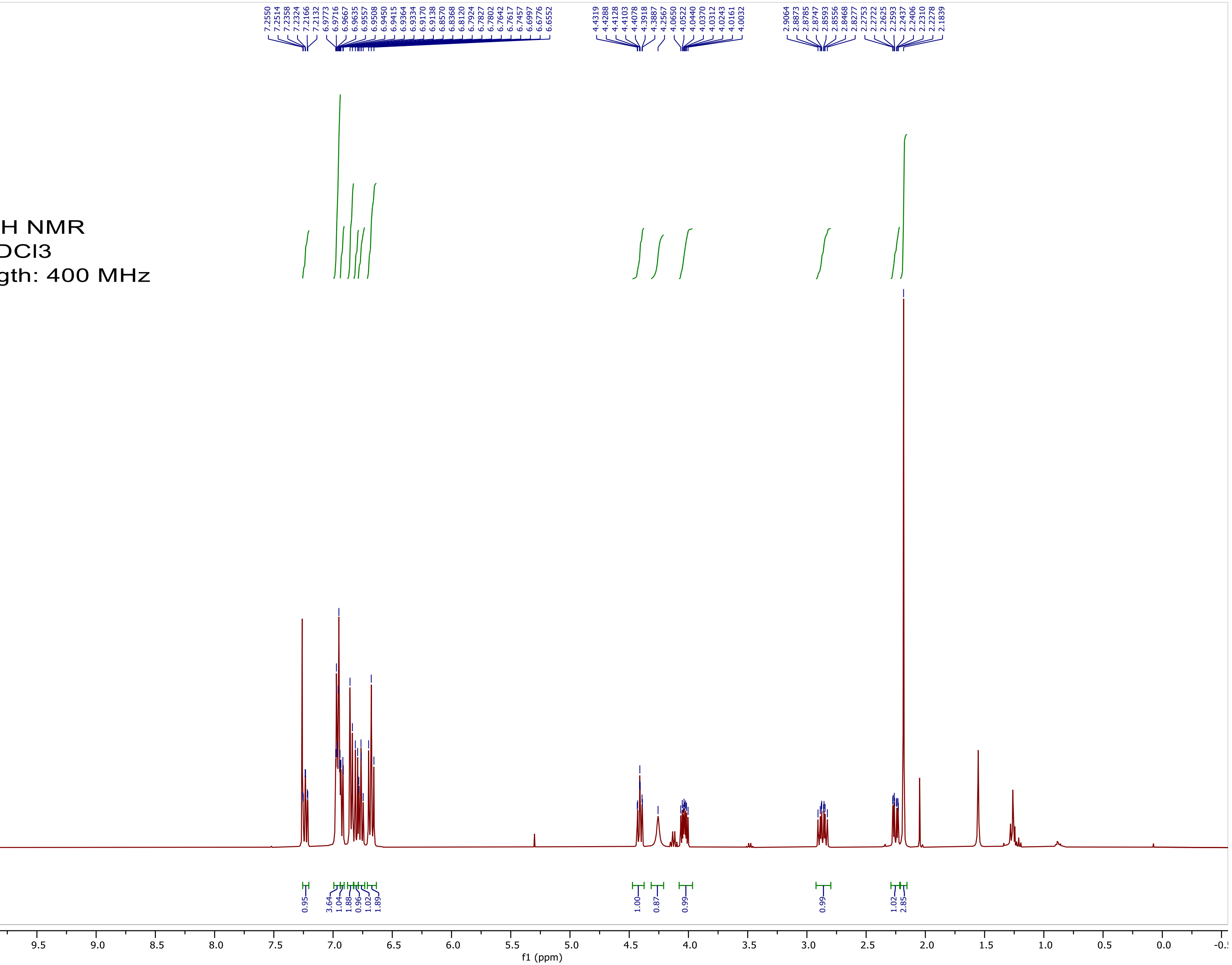

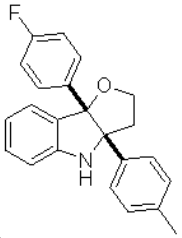

4f
Analysis: 13C NMR
Solvent: CDCl3
Field strength: 101 MHz

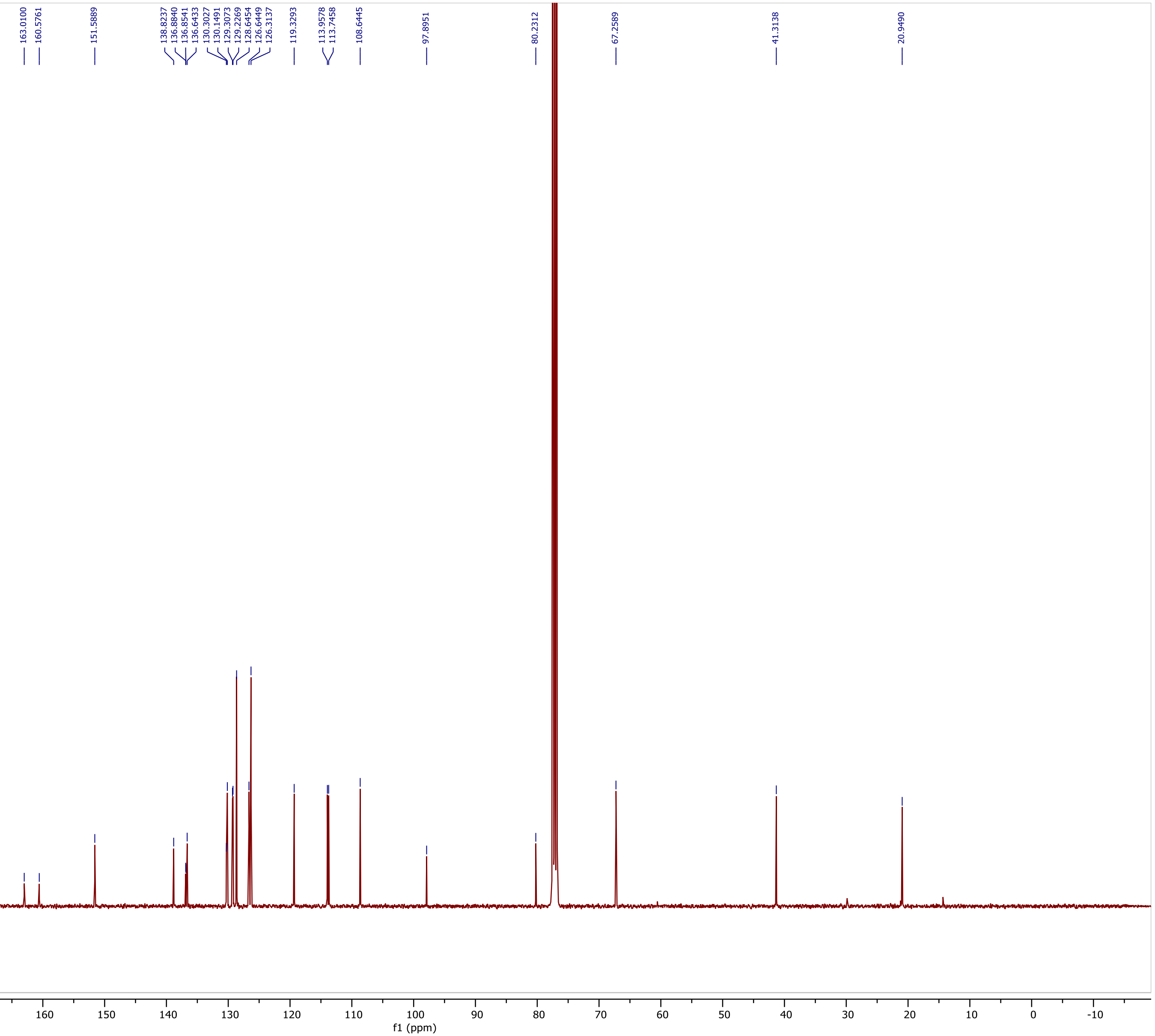

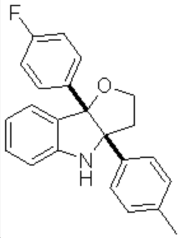

4f
Analysis: 19F NMR
Solvent: CDCl3
Field strength: 376 MHz

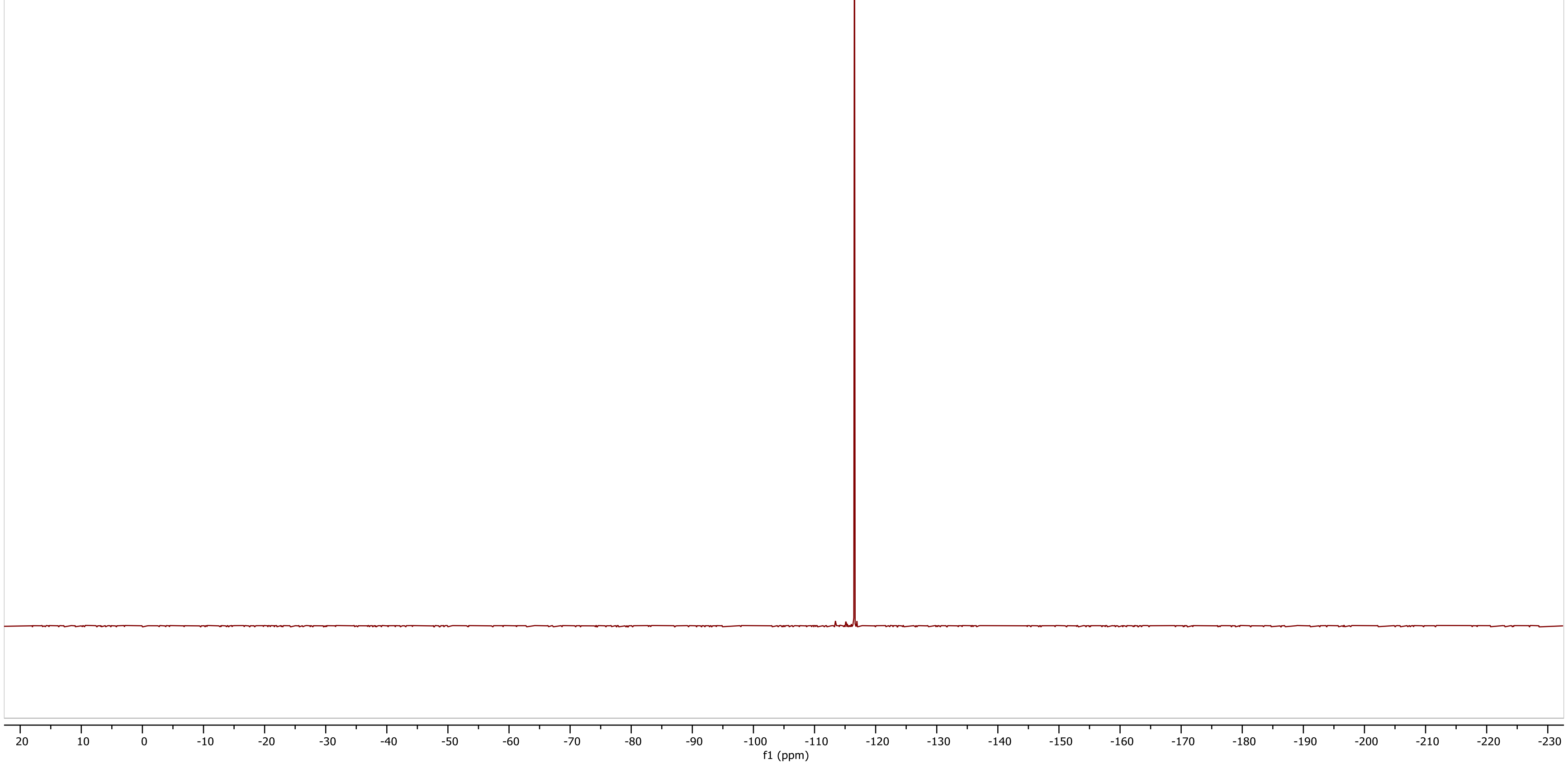

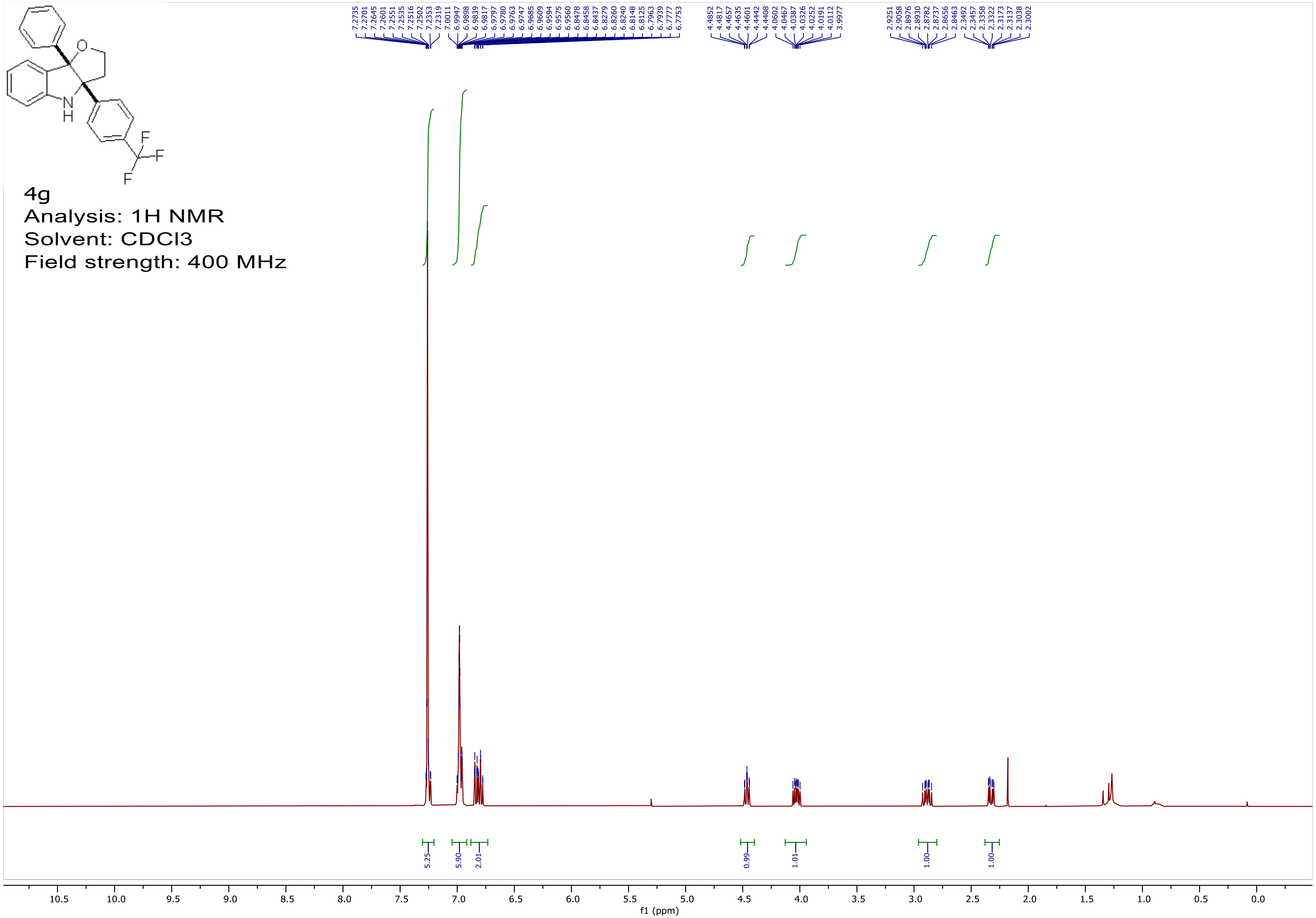
4g
Analysis: 1H NMR
Solvent: CDCl3
Field strength: 400 MHz
7.2735
7.2701
7.2645
7.2601
7.2551
7.2535
7.2516
7.2502
7.2353
7.2319
7.0011
6.9947
6.9898
6.9839
6.9817
6.9797
6.9780
6.9763
6.9747
6.9685
6.9609
6.9594
6.9575
6.9560
6.8478
6.8458
6.8437
6.8279
6.8260
6.8240
6.8148
6.8125
6.7963
6.7939
6.7777
6.7753
4.4852
4.4817
4.4657
4.4635
4.4601
4.4442
4.4408
4.0602
4.0467
4.0387
4.0326
4.0252
4.0191
4.0112
3.9977
2.9251
2.9058
2.8976
2.8930
2.8782
2.8737
2.8656
2.8463
2.3492
2.3457
2.3358
2.3322
2.3173
2.3137
2.3038
2.3002
5.25
5.90
2.01
0.99
1.01
1.00
1.00
10.5 10.0 9.5 9.0 8.5 8.0 7.5 7.0 6.5 6.0 5.5 5.0 4.5 4.0 3.5 3.0 2.5 2.0 1.5 1.0 0.5 0.0
f1 (ppm)

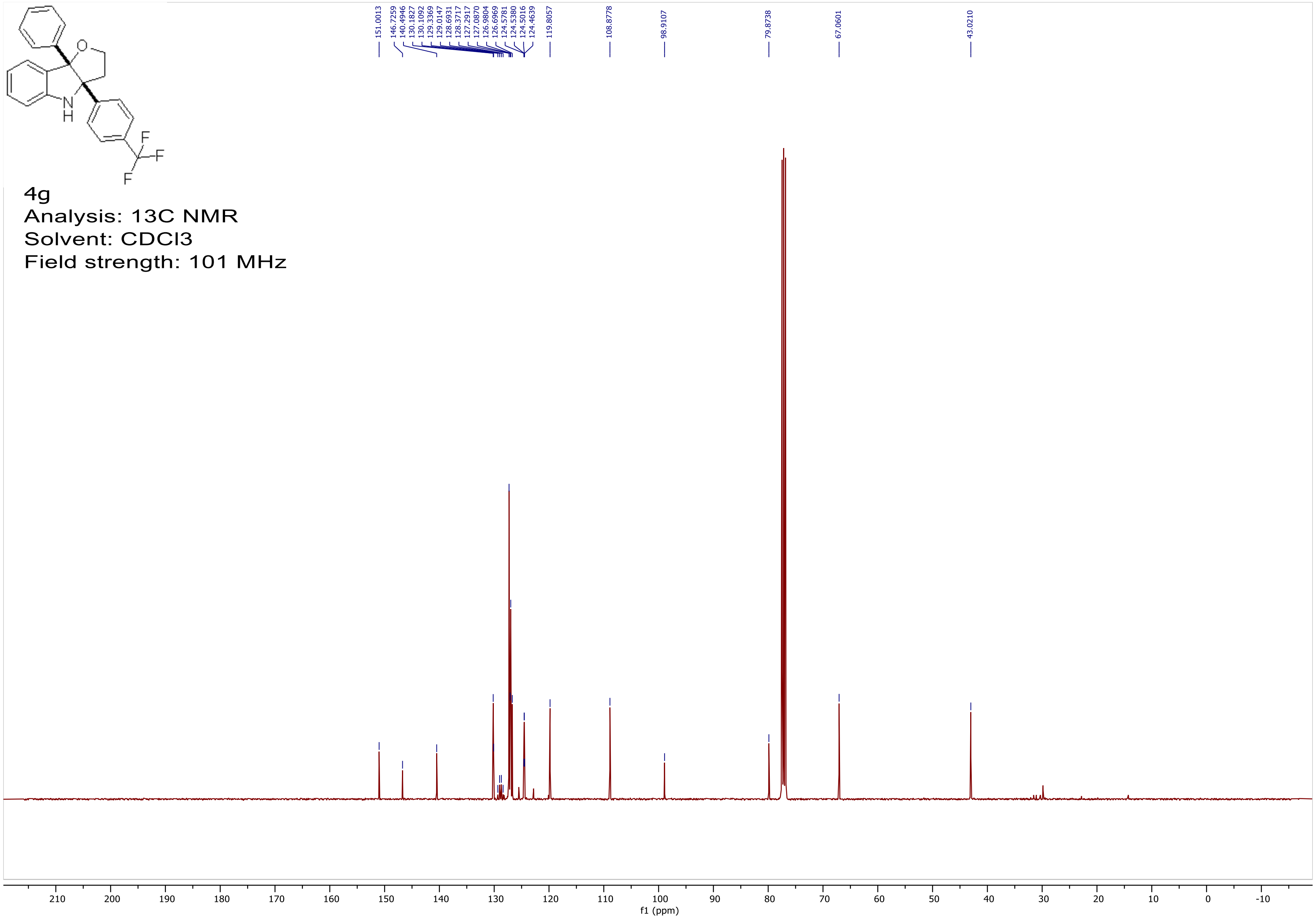
4g
Analysis: 13C NMR
Solvent: CDCl3
Field strength: 101 MHz
151.0013
146.7259
140.4946
130.1827
130.1092
129.3369
129.0147
128.6931
128.3717
127.2917
127.0870
126.9804
126.6969
124.5781
124.5380
124.5016
124.4639
119.8057
108.8778
98.9107
79.8738
67.0601
43.0210
f1 (ppm)

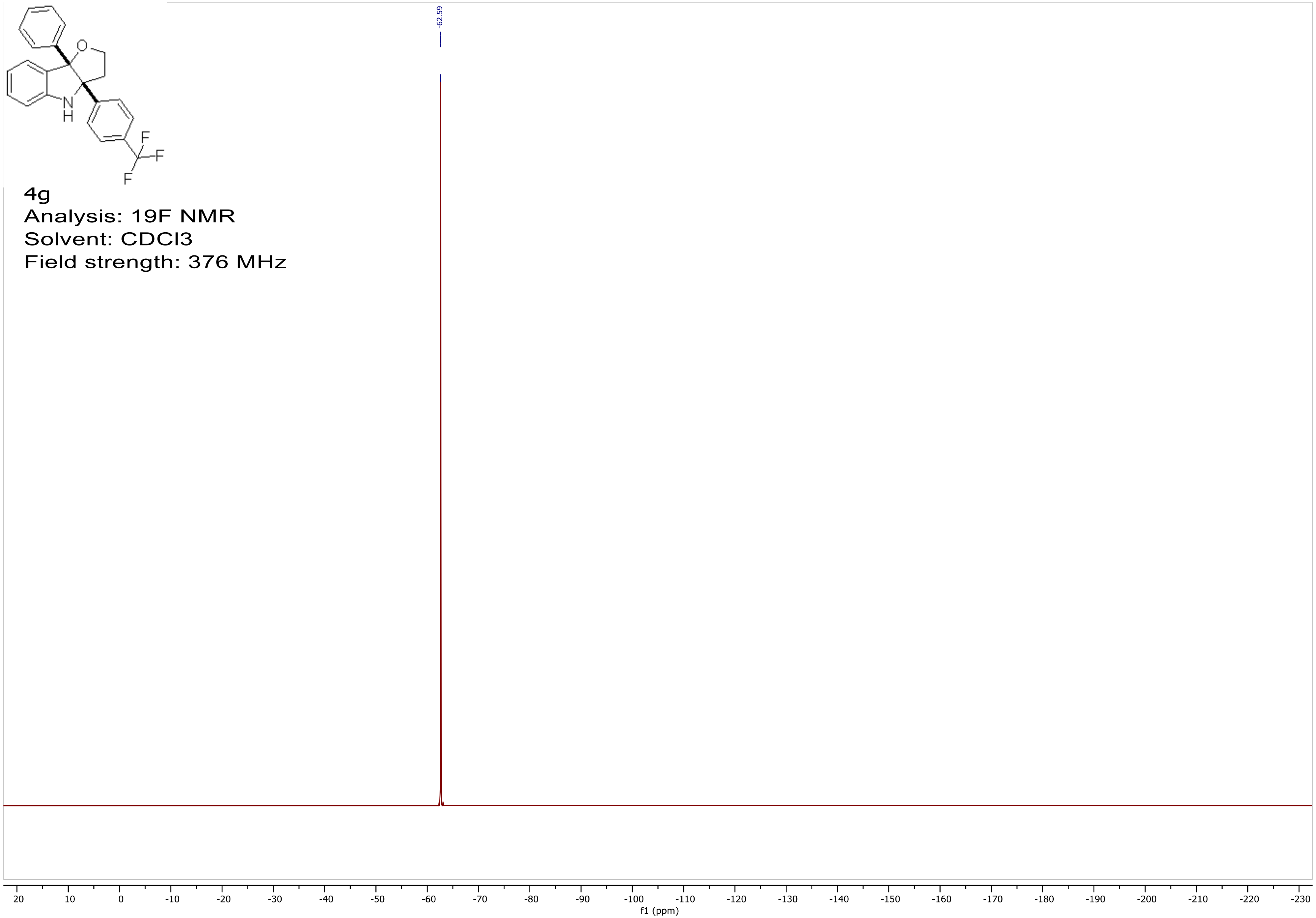
4g
Analysis: 19F NMR
Solvent: CDCl3
Field strength: 376 MHz
-62.59
f1 (ppm)

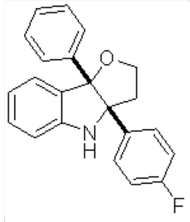

4h
Analysis: 1H NMR
Solvent: CDCl3
Field strength: 400 MHz

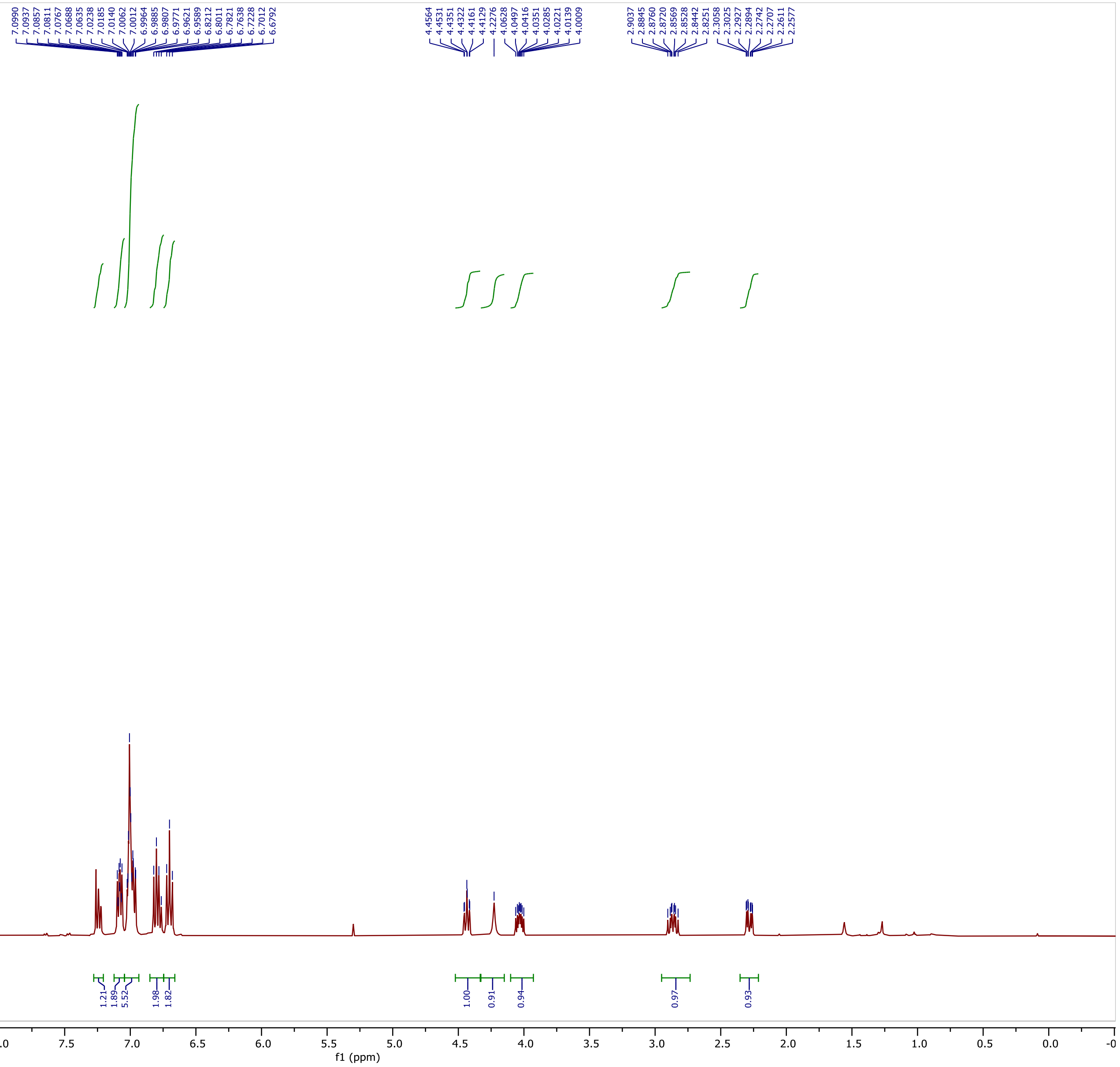

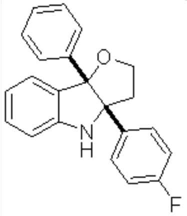

4h
Analysis: 13C NMR
Solvent: CDCl3
Field strength: 101 MHz

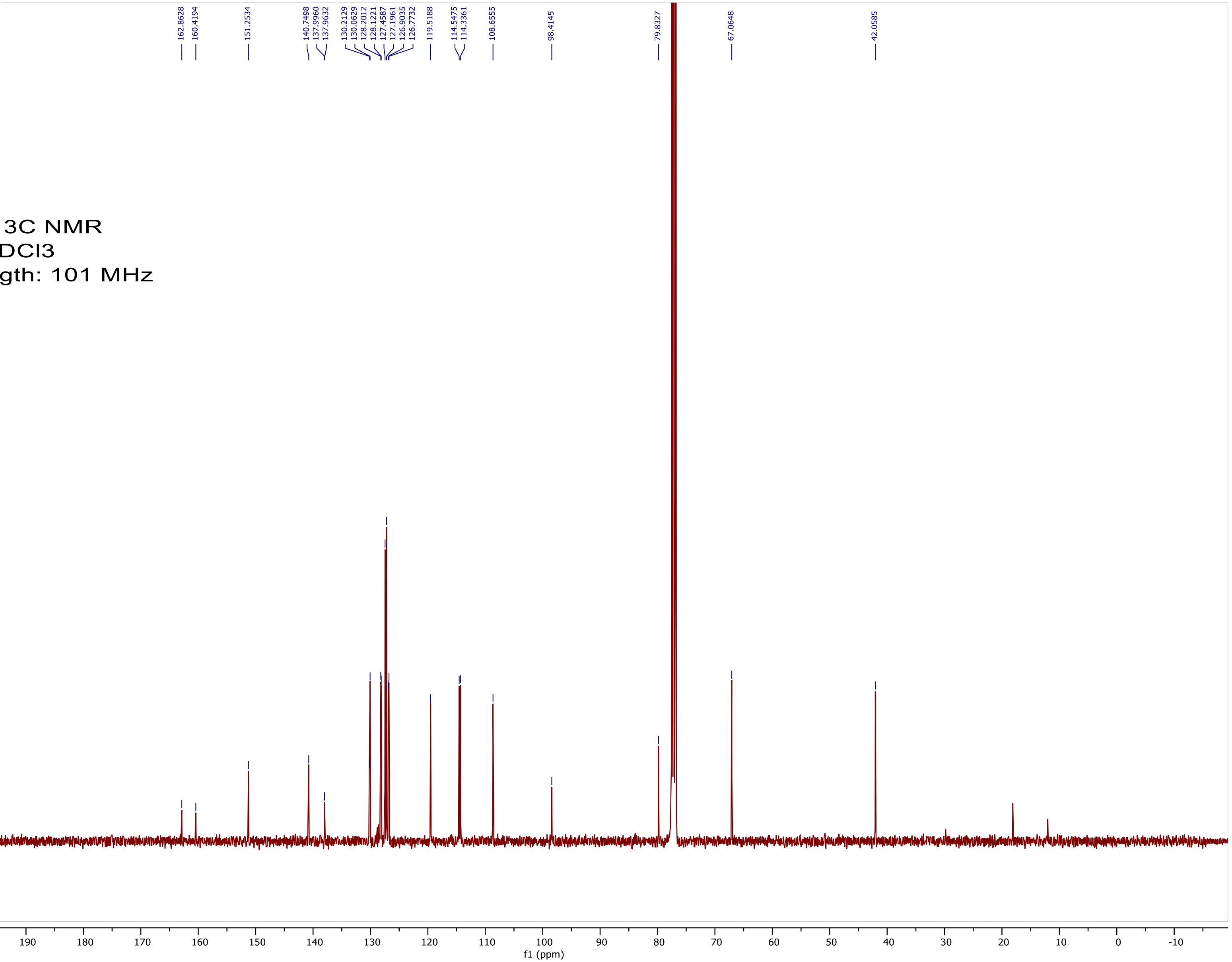

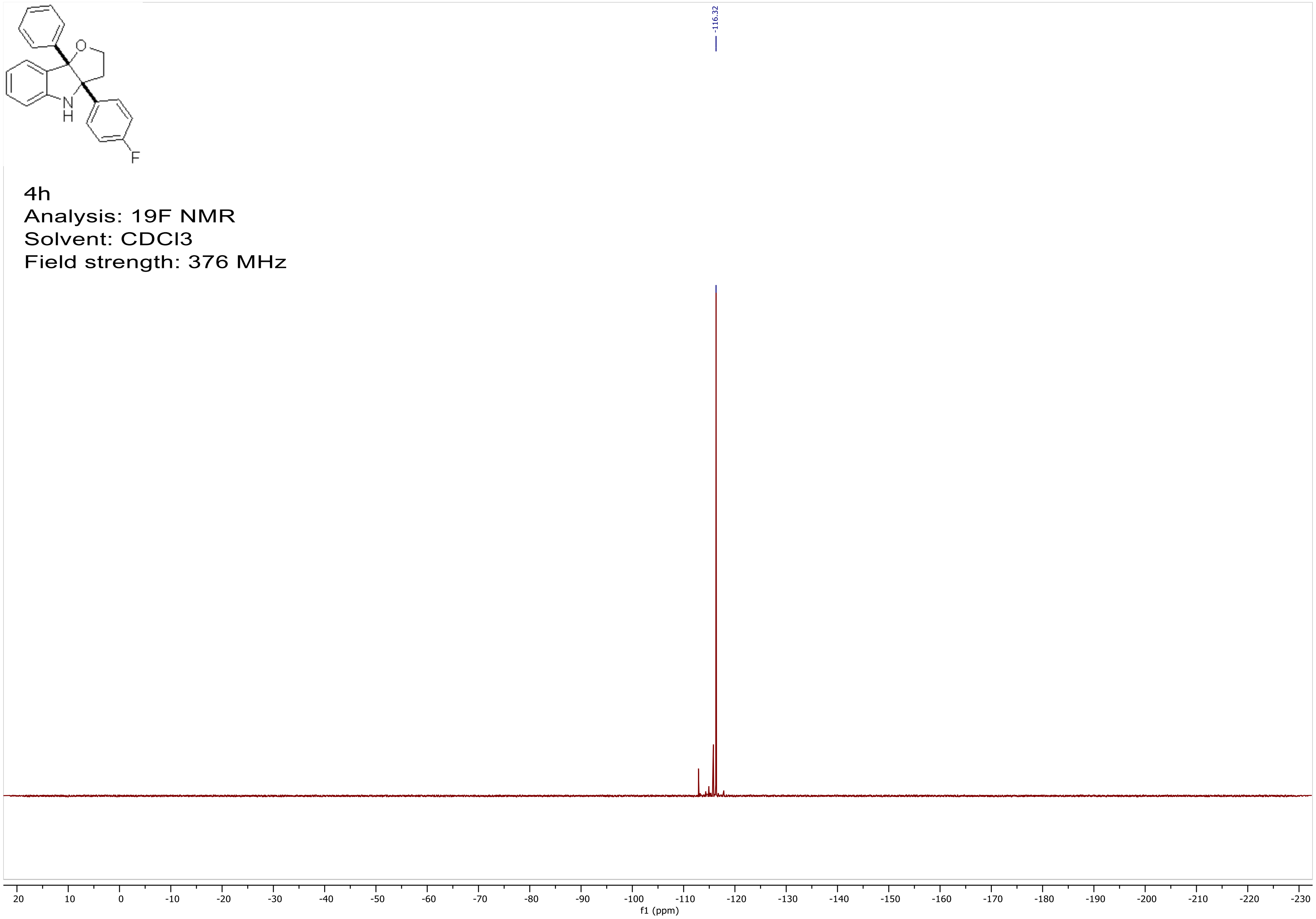

O
N
H
F
4h
Analysis: 19F NMR
Solvent: CDCl3
Field strength: 376 MHz
-116.32
20 10 0 -10 -20 -30 -40 -50 -60 -70 -80 -90 -100 -110 -120 -130 -140 -150 -160 -170 -180 -190 -200 -210 -220 -230
f1 (ppm)

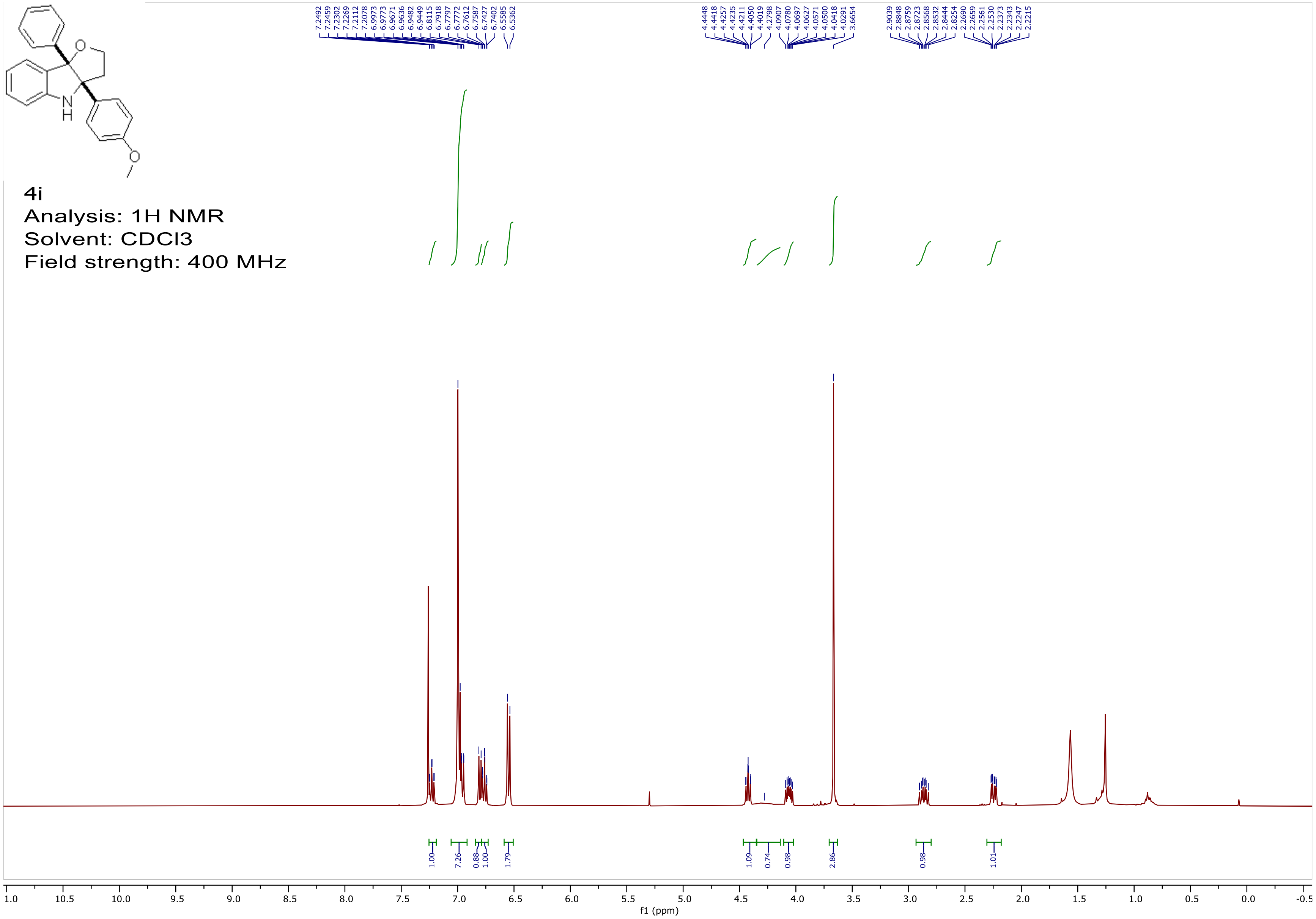
4i
Analysis: 1H NMR
Solvent: CDCl3
Field strength: 400 MHz
f1 (ppm)

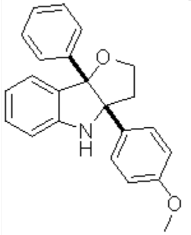

4i
Analysis: 13C NMR
Solvent: CDCl3
Field strength: 101 MHz

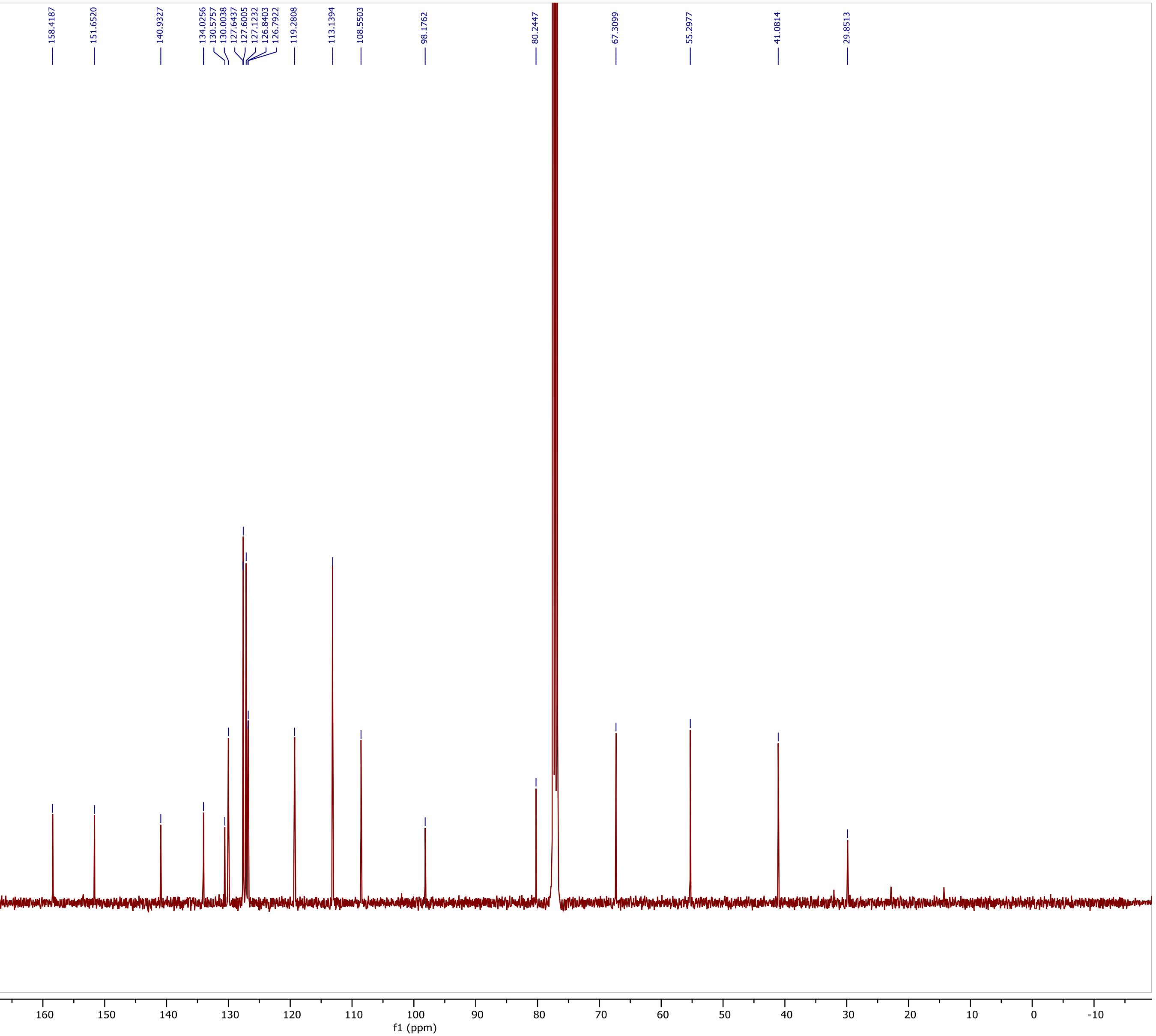

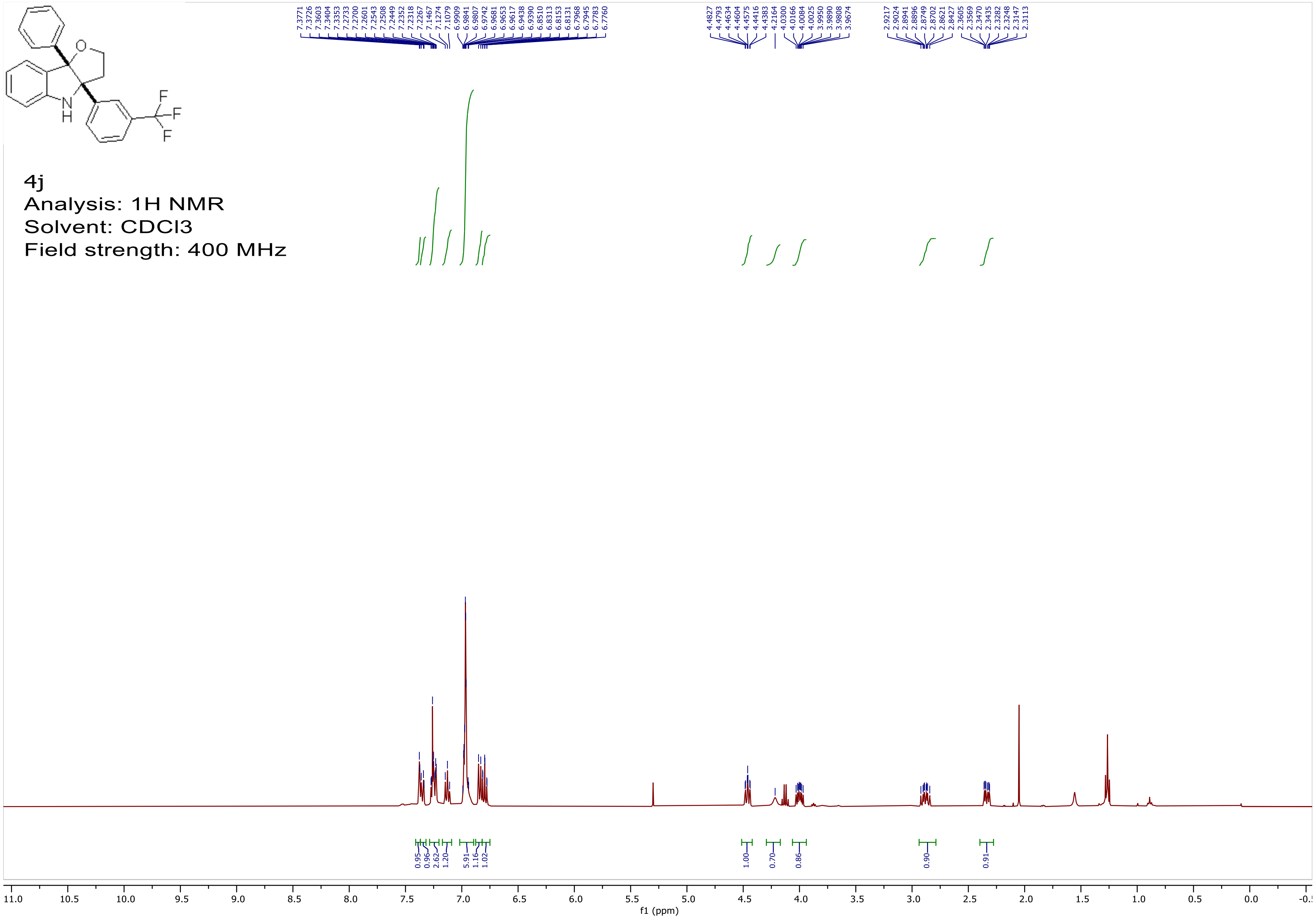
4j
Analysis: 1H NMR
Solvent: CDCl3
Field strength: 400 MHz
f1 (ppm)

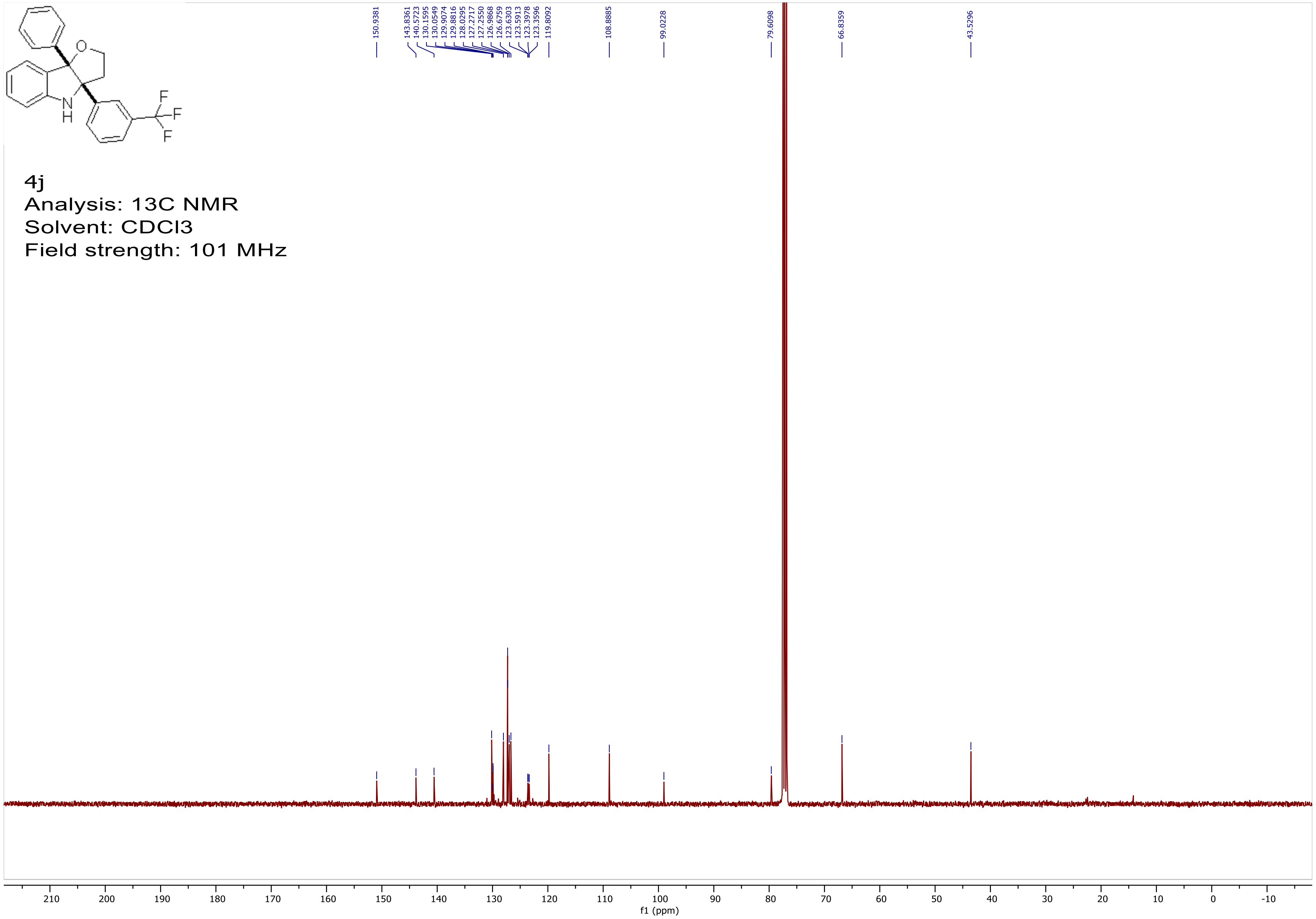

O
N
H
F
F
F
4j
Analysis: 13C NMR
Solvent: CDCl3
Field strength: 101 MHz
150.9381
143.8361
140.5723
130.1595
130.0549
129.9074
129.8816
128.0295
127.2717
127.2550
126.9868
126.6759
123.6303
123.5913
123.3978
123.3596
119.8092
108.8885
99.0228
79.6098
66.8359
43.5296
210 200 190 180 170 160 150 140 130 120 110 100 90 80 70 60 50 40 30 20 10 0 -10
f1 (ppm)

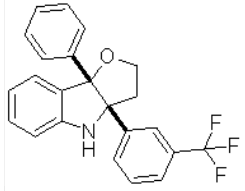

4j
Analysis: 19F NMR
Solvent: CDCl3
Field strength: 376 MHz

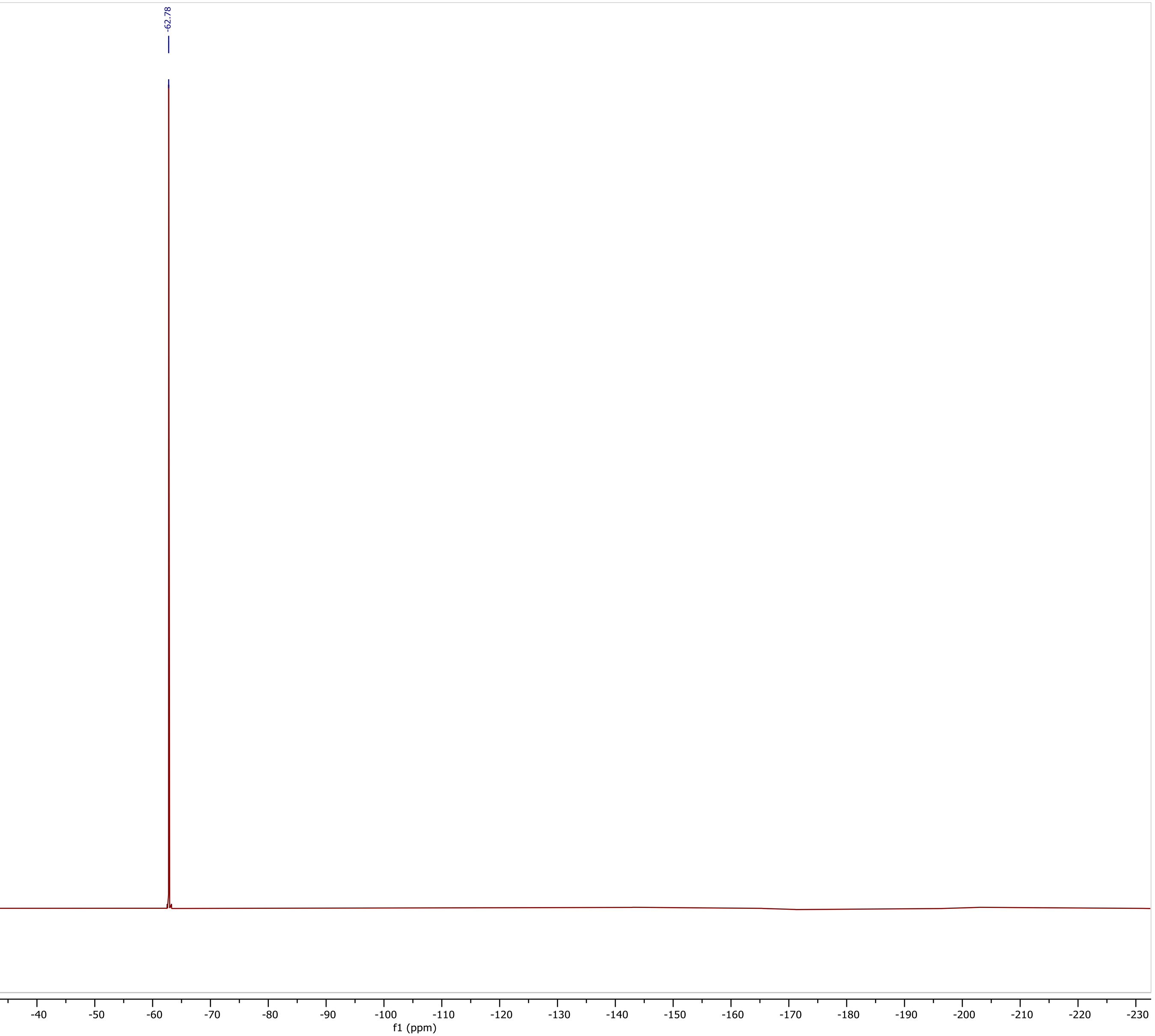

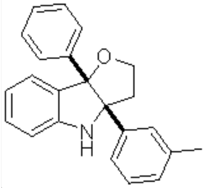

4k
Analysis: 1H NMR
Solvent: CDCl3
Field strength: 400 MHz

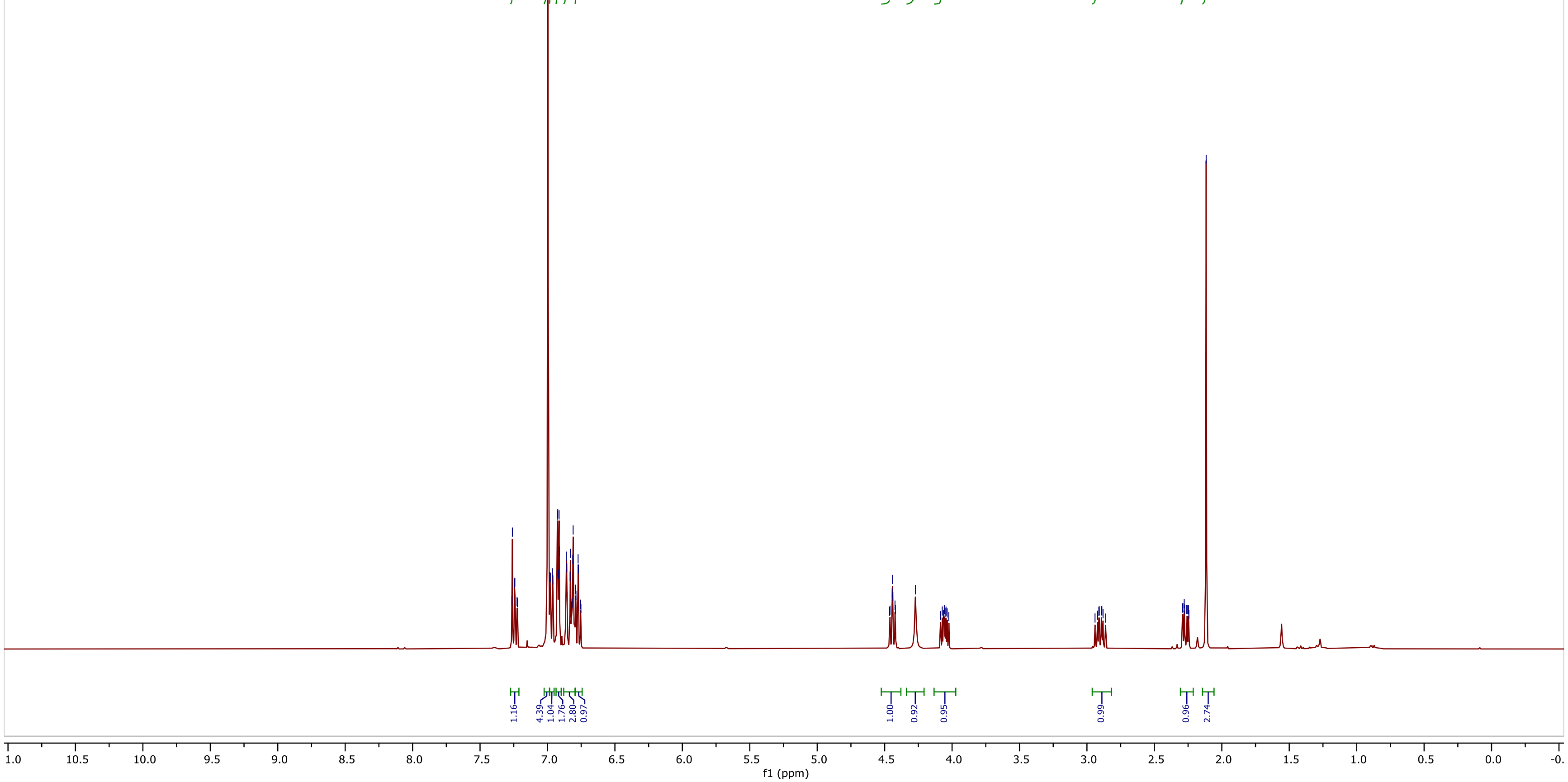

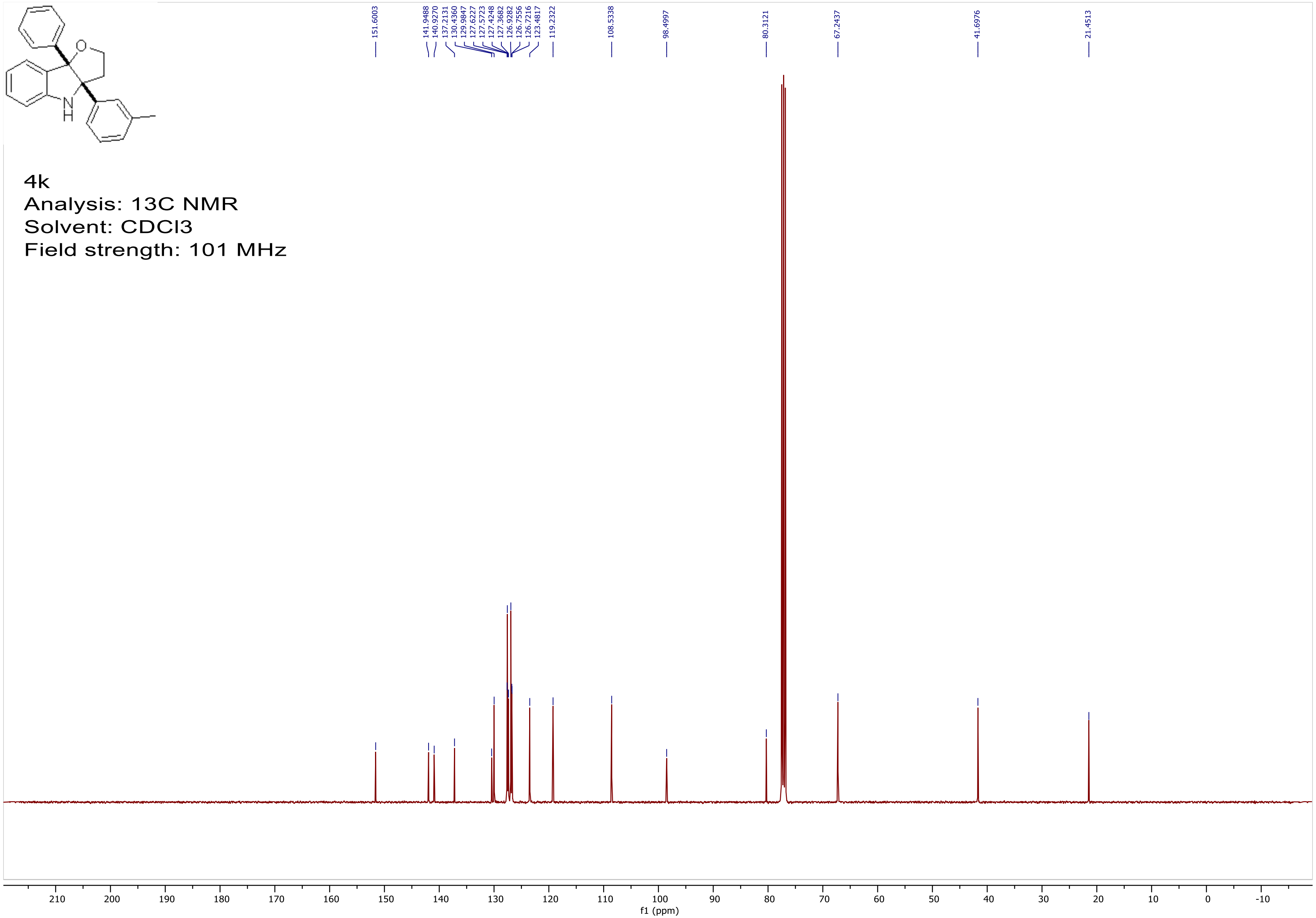
4k
Analysis: 13C NMR
Solvent: CDCl3
Field strength: 101 MHz
151.6003
141.9488
140.9270
137.2131
130.4360
129.9847
127.6227
127.5723
127.4248
127.3682
126.9282
126.7556
126.7216
123.4817
119.2322
108.5338
98.4997
80.3121
67.2437
41.6976
21.4513
210 200 190 180 170 160 150 140 130 120 110 100 90 80 70 60 50 40 30 20 10 0 -10
f1 (ppm)

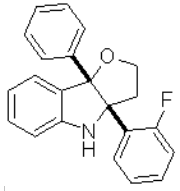

4l
Analysis: 1H NMR
Solvent: CDCl3
Field strength: 400 MHz

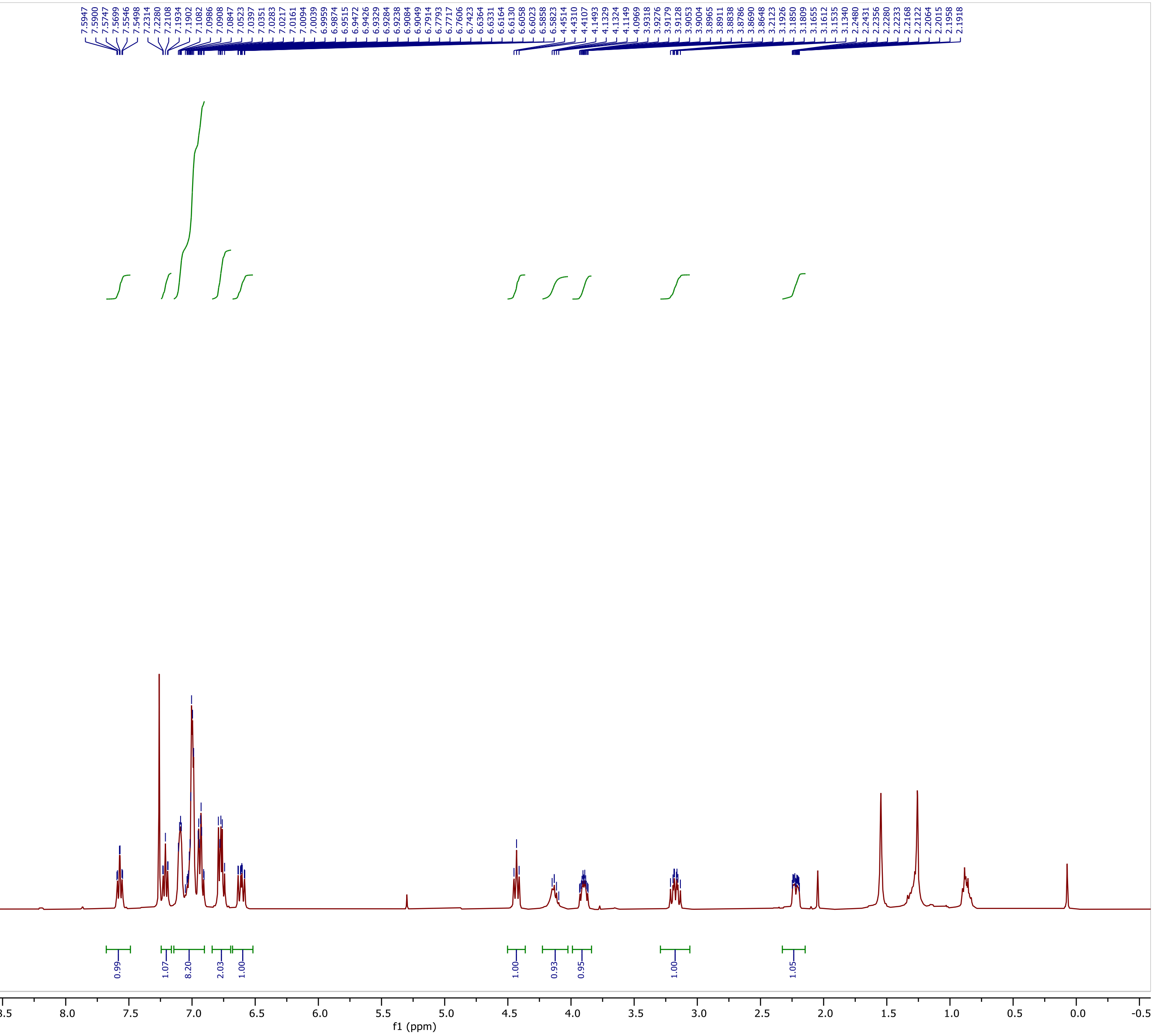

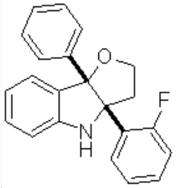

4l
Analysis: 13C NMR
Solvent: CDCl3
Field strength: 101 MHz

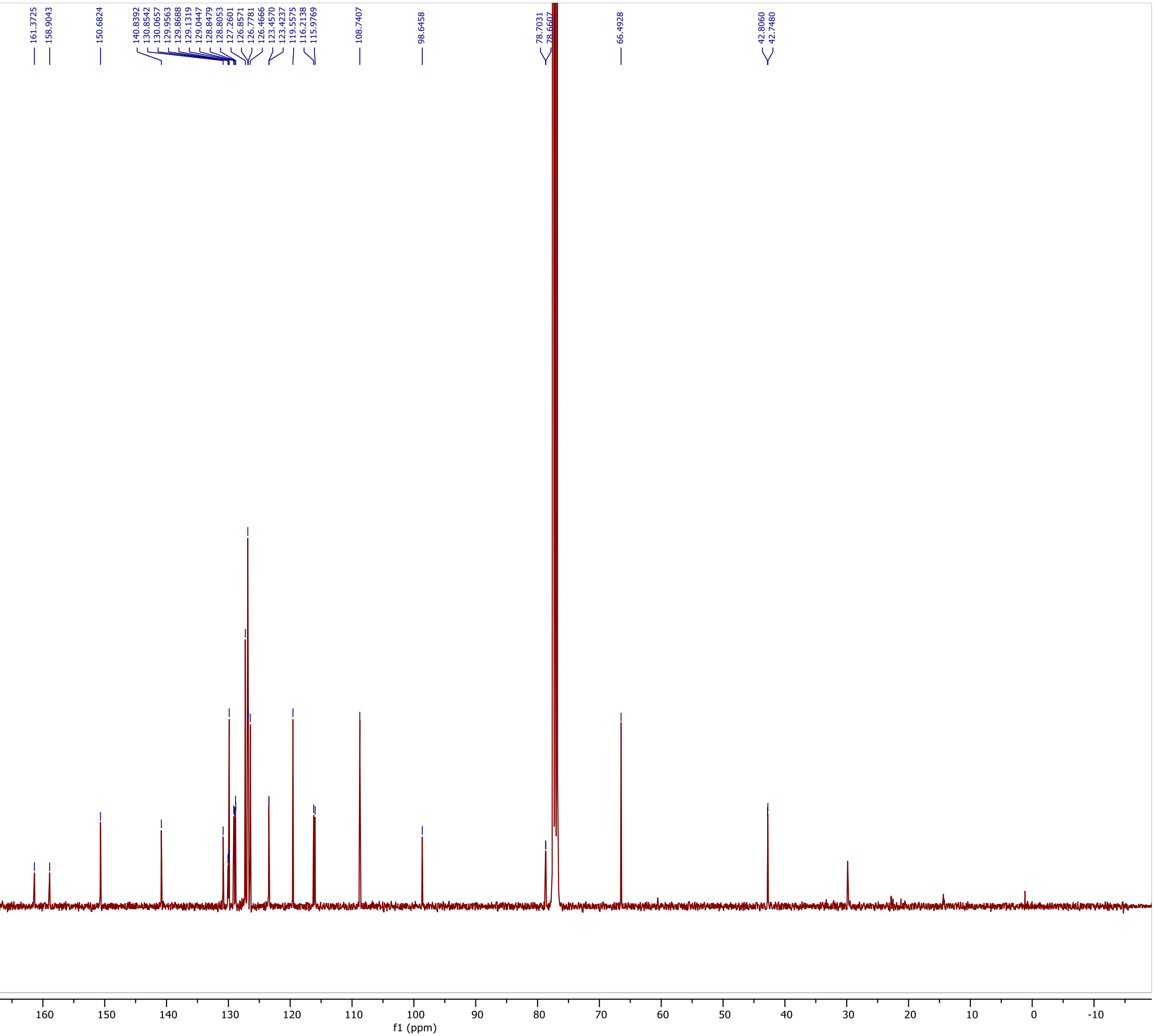

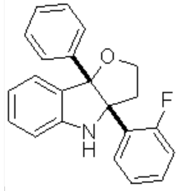

4l
Analysis: 19F NMR
Solvent: CDCl3
Field strength: 376 MHz

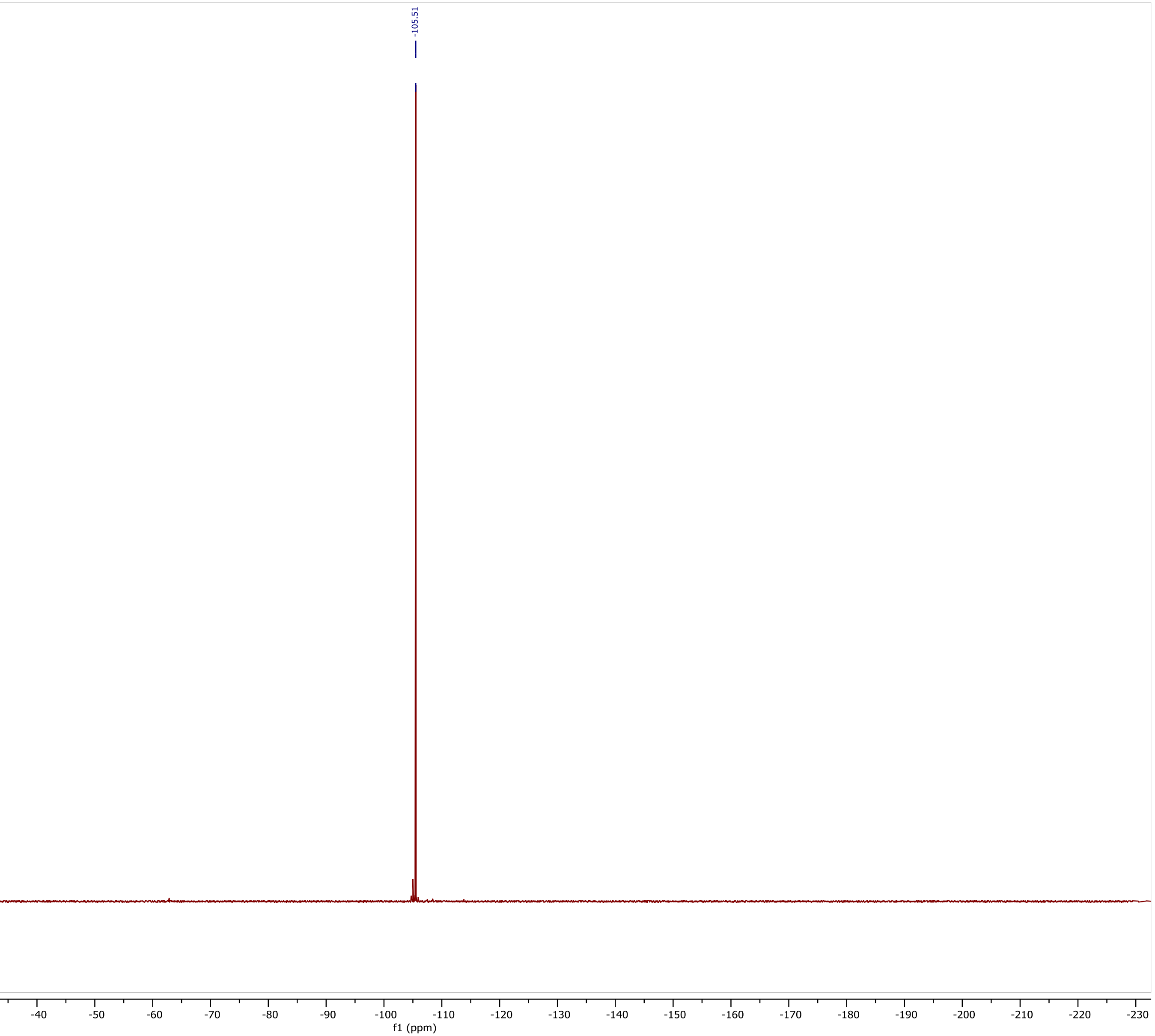

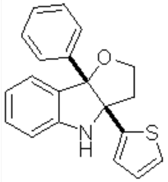

4m
Analysis: 1H NMR
Solvent: CDCl3
Field strength: 400 MHz

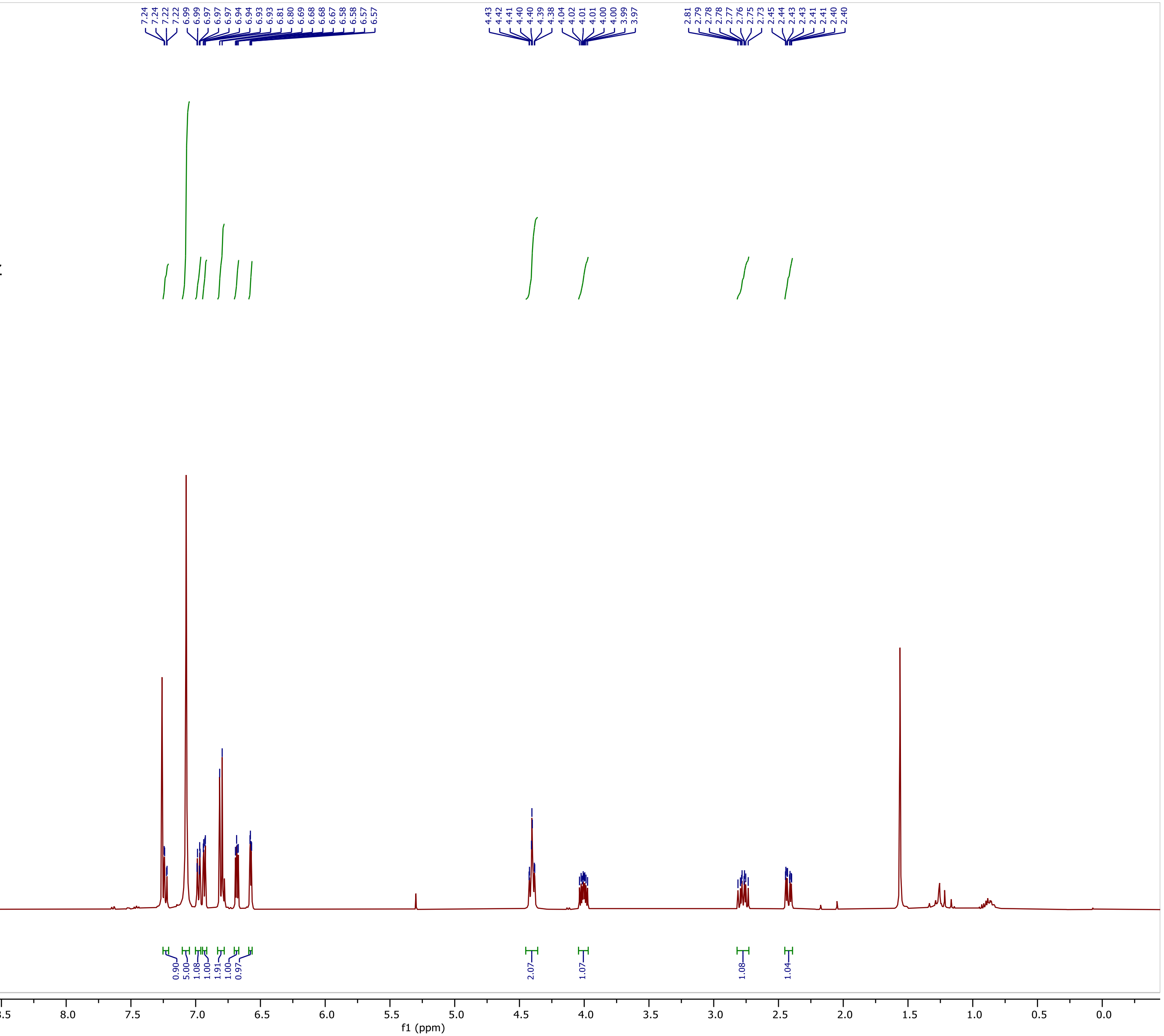

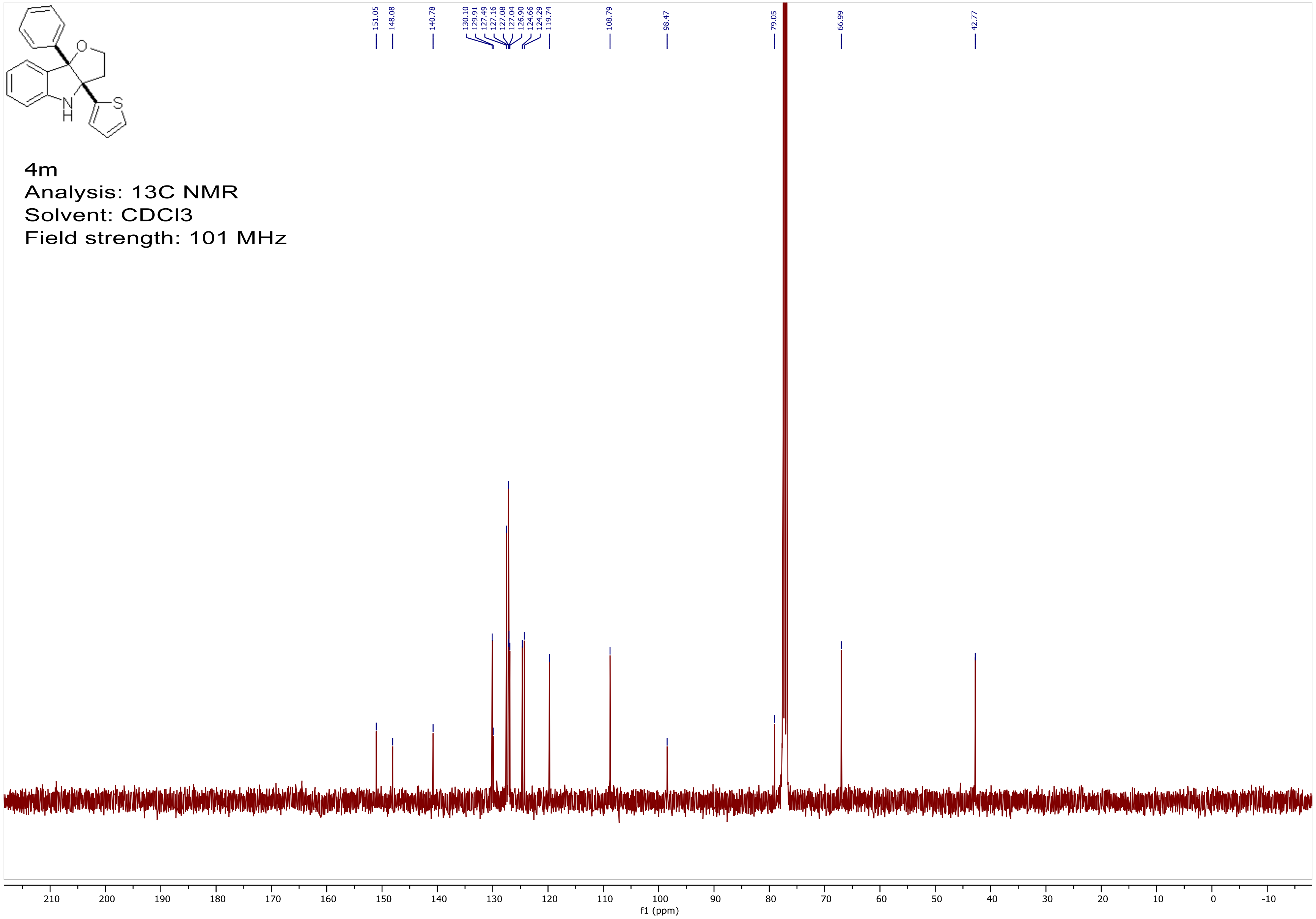
4m
Analysis: 13C NMR
Solvent: CDCl3
Field strength: 101 MHz
151.05
148.08
140.78
130.10
129.91
127.49
127.16
127.08
127.04
126.90
124.66
124.29
119.74
108.79
98.47
79.05
66.99
42.77
f1 (ppm)

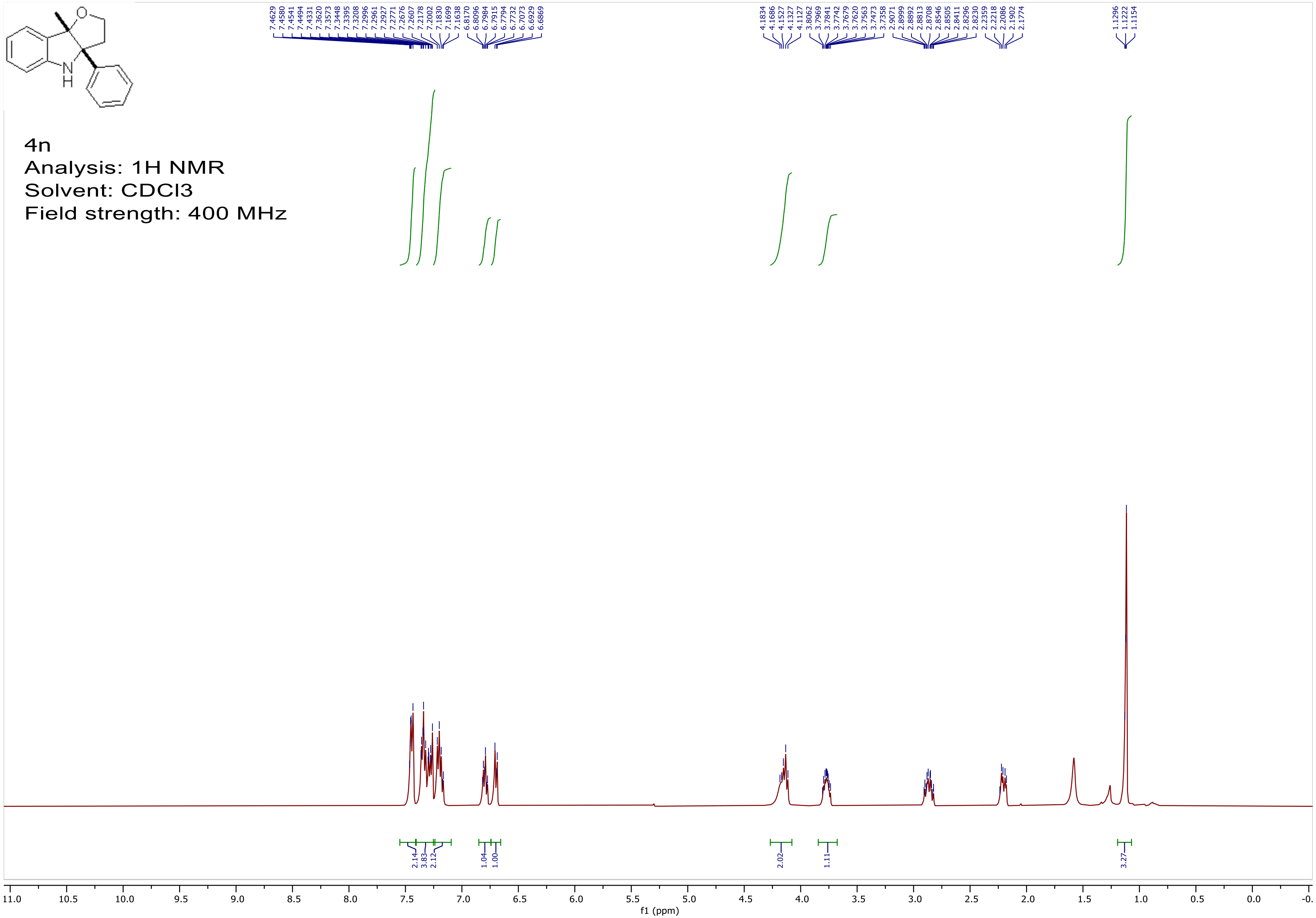
4n
Analysis: 1H NMR
Solvent: CDCl3
Field strength: 400 MHz
f1 (ppm)

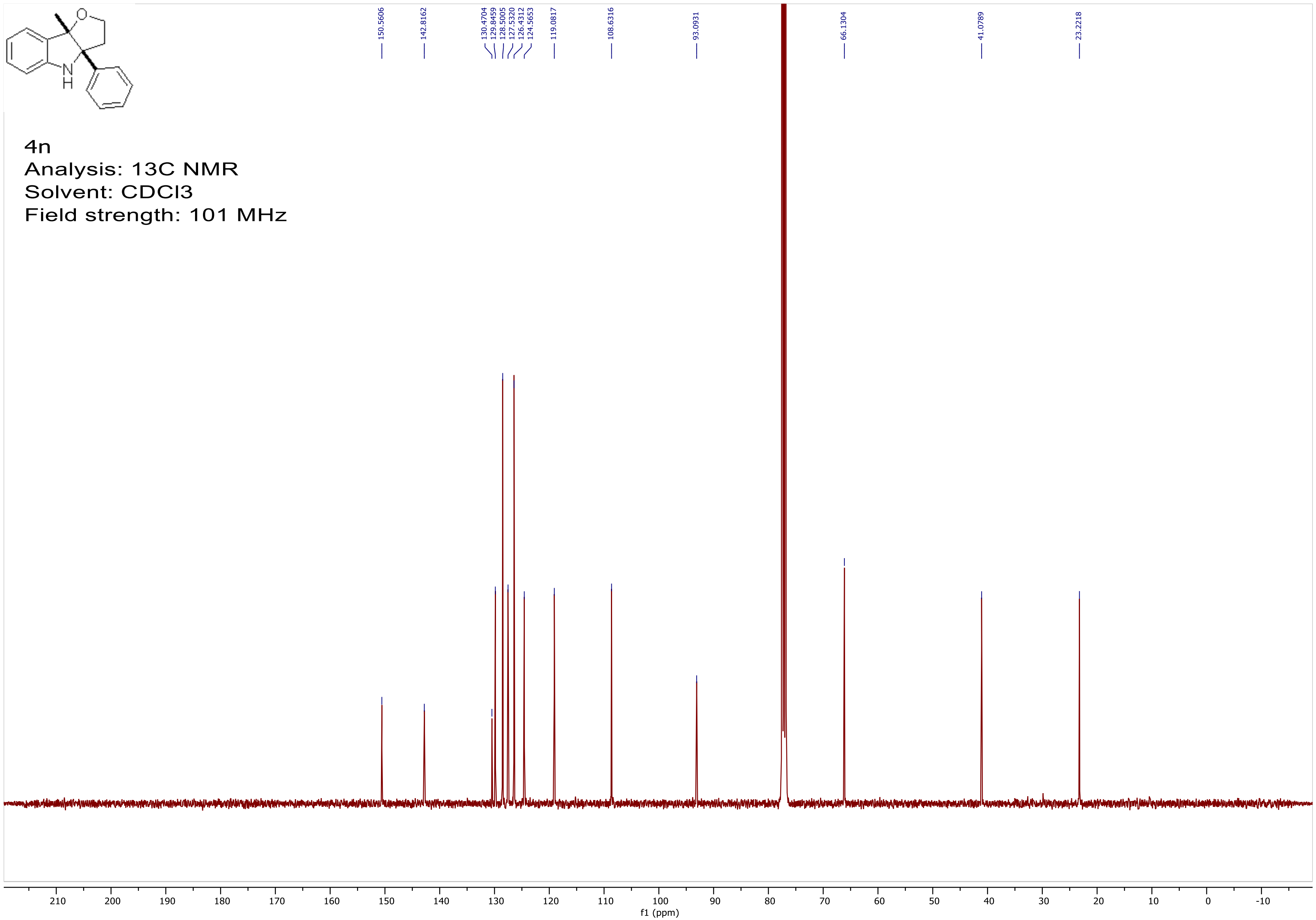
4n
Analysis: 13C NMR
Solvent: CDCl3
Field strength: 101 MHz
150.5606
142.8162
130.4704
129.8459
128.5005
127.5320
126.4312
124.5653
119.0817
108.6316
93.0931
66.1304
41.0789
23.2218
f1 (ppm)

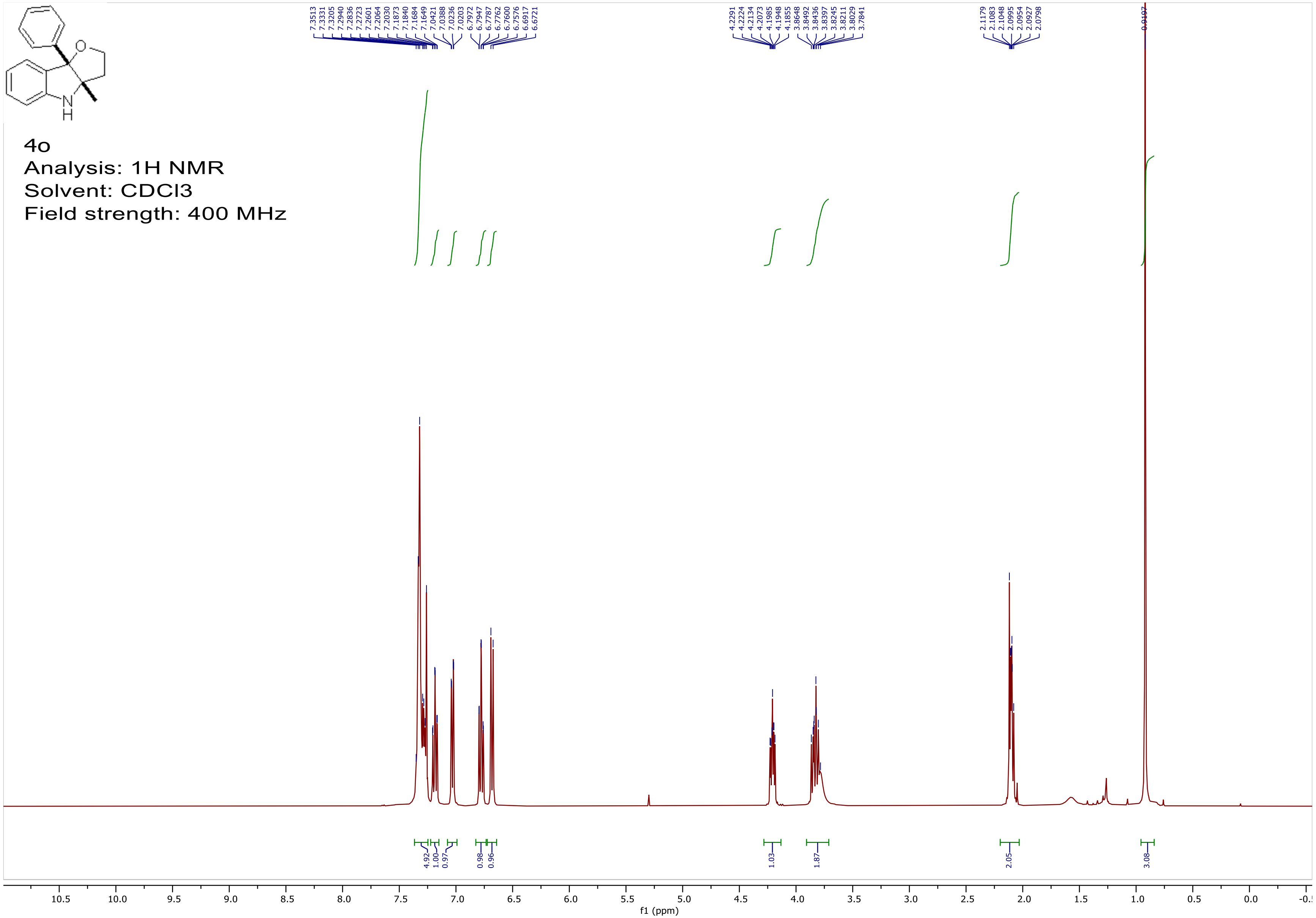
4o
Analysis: 1H NMR
Solvent: CDCl3
Field strength: 400 MHz
f1 (ppm)

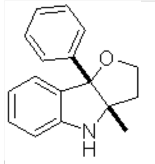

4o
Analysis: 13C NMR
Solvent: CDCl3
Field strength: 101 MHz

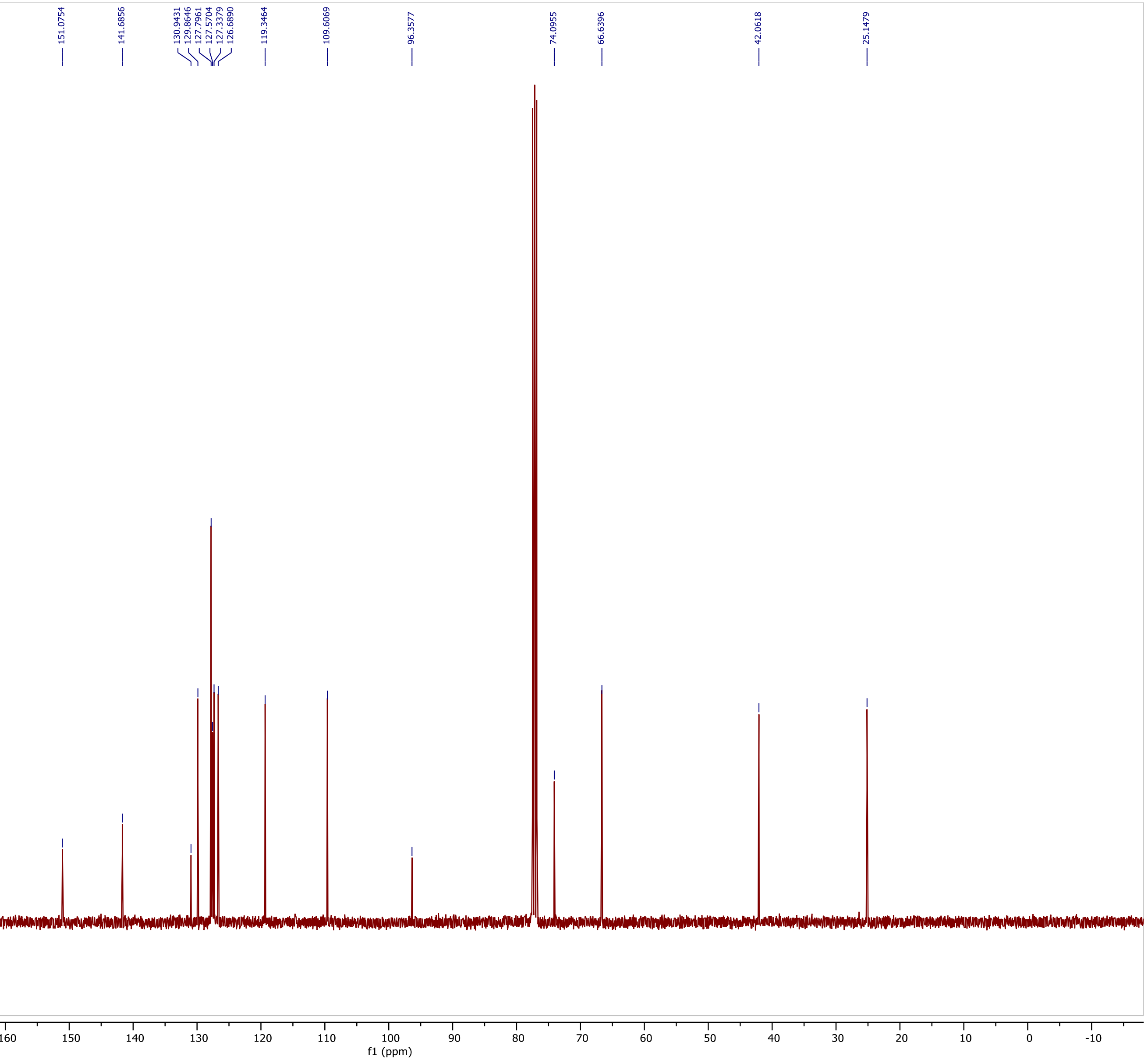

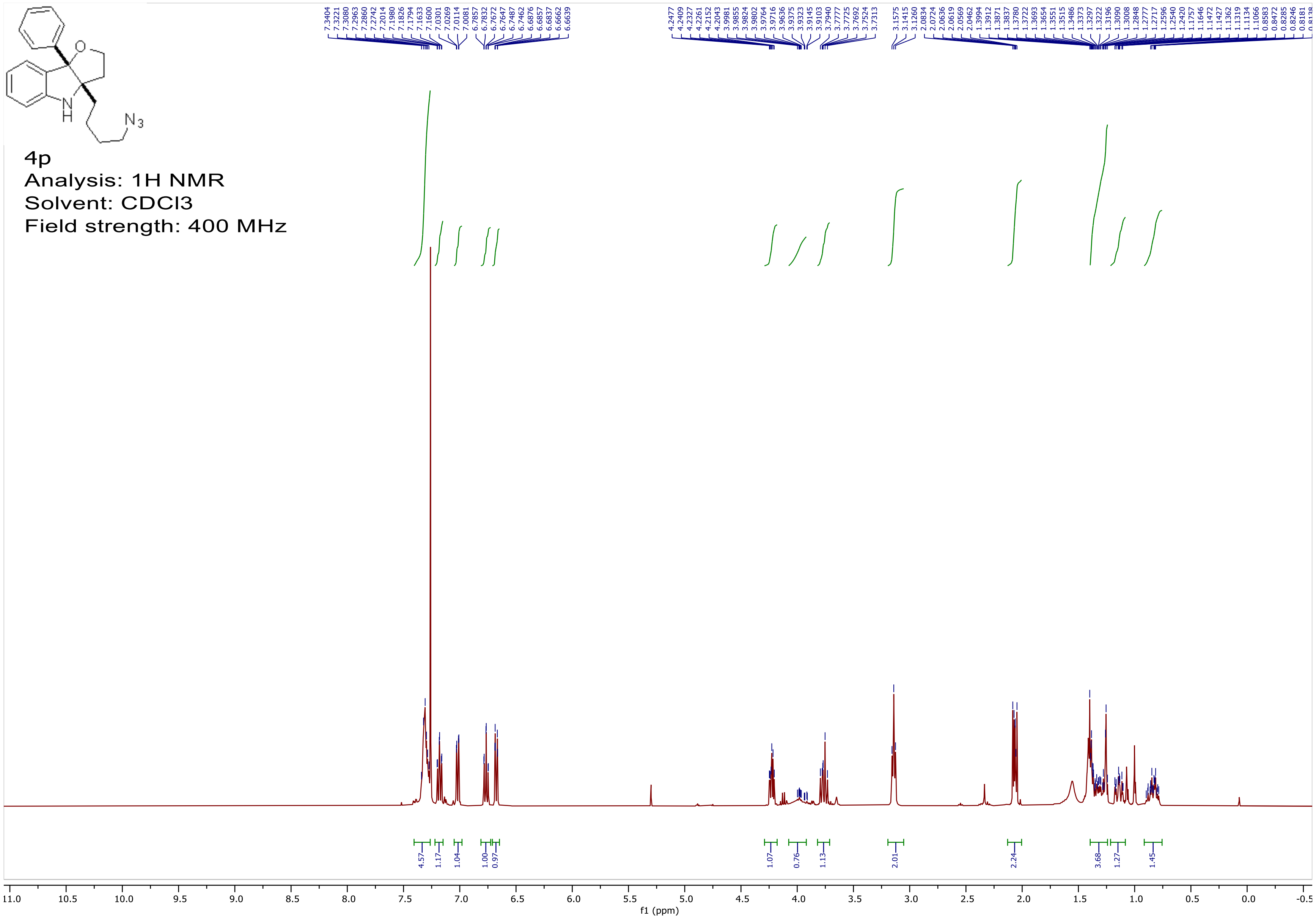
N3
4p
Analysis: 1H NMR
Solvent: CDCl3
Field strength: 400 MHz
f1 (ppm)

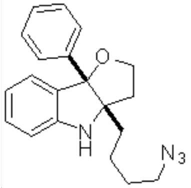

4p
Analysis: 13C NMR
Solvent: CDCl3
Field strength: 101 MHz

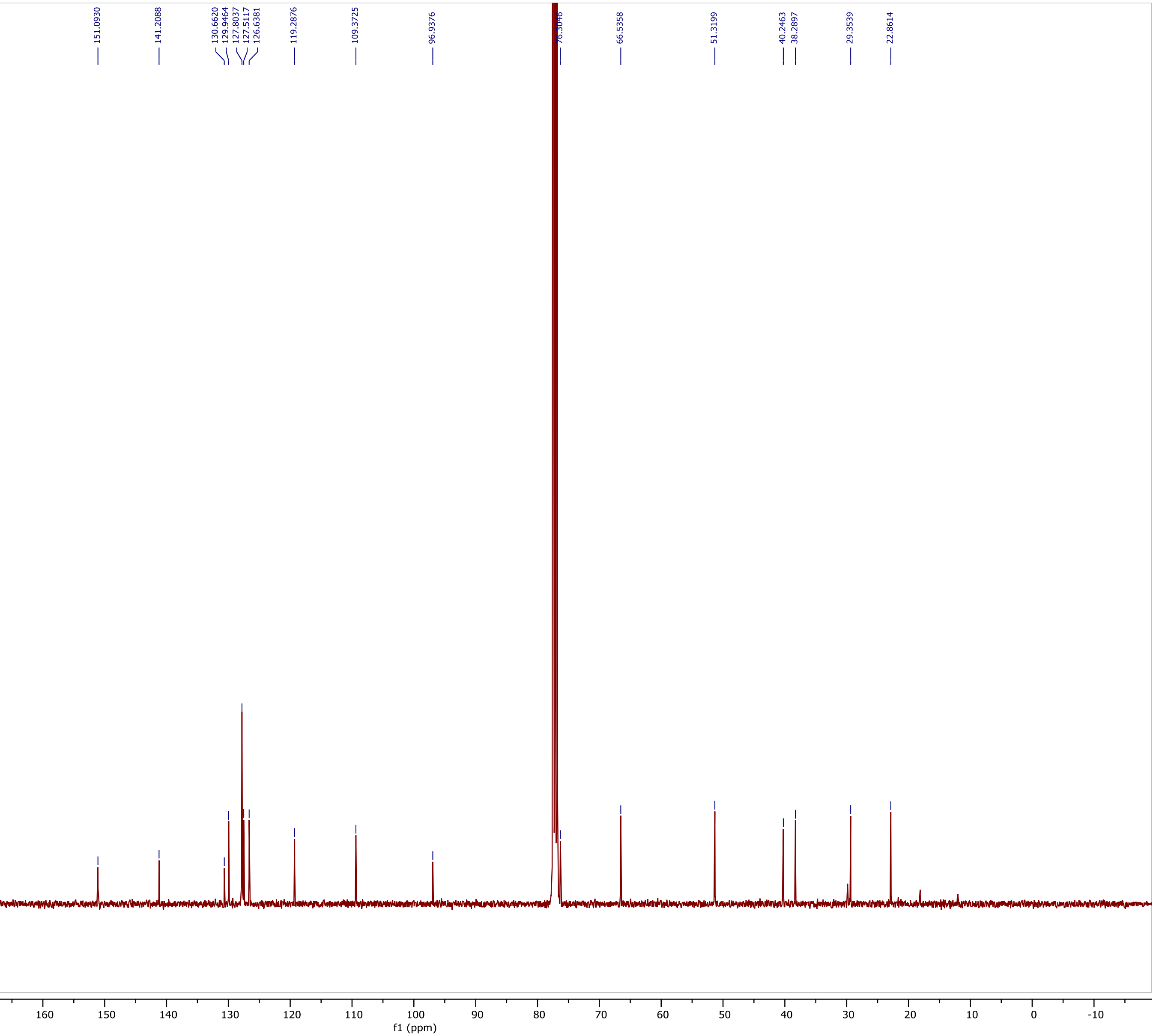

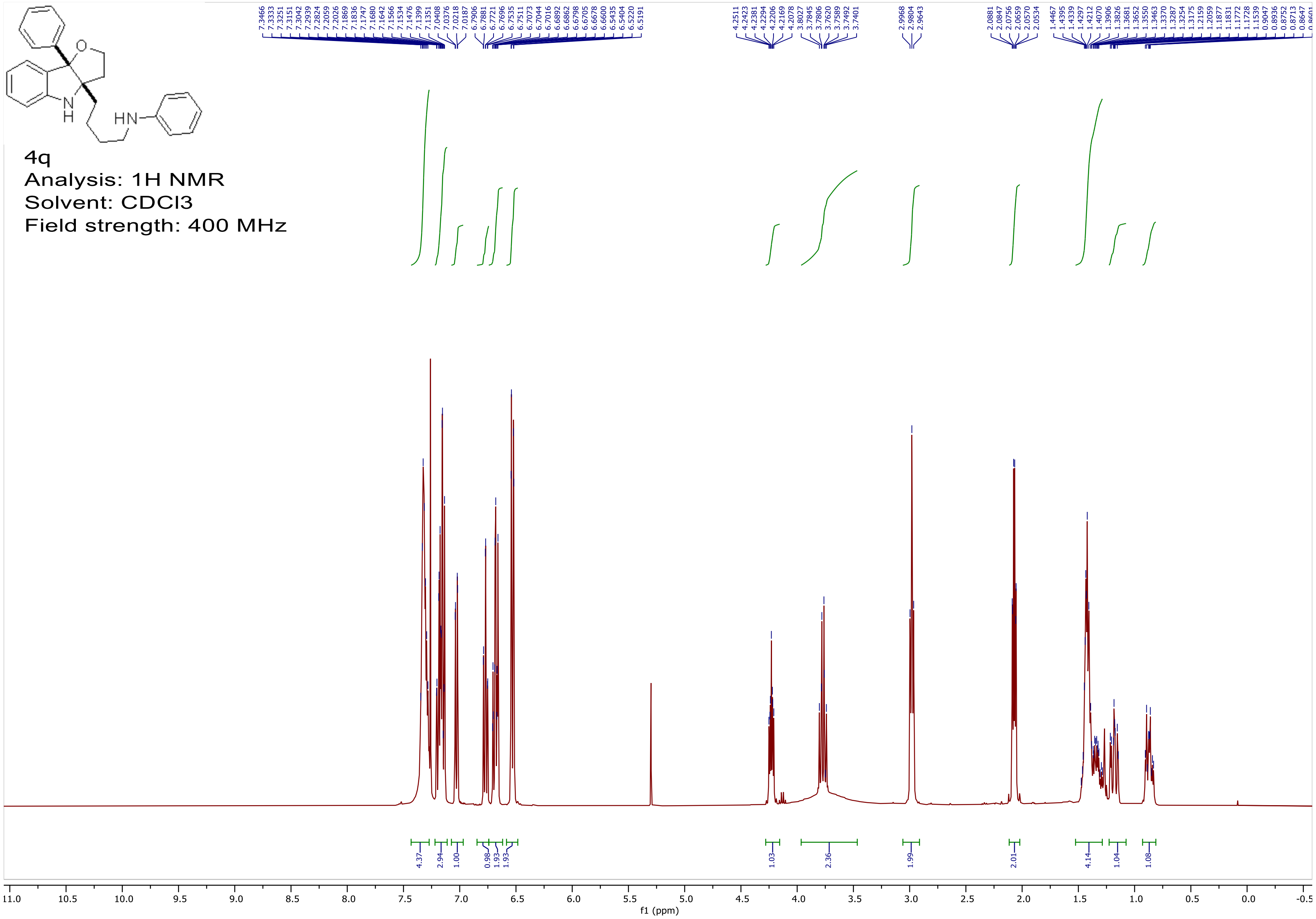
4q
Analysis: 1H NMR
Solvent: CDCl3
Field strength: 400 MHz
HN
N
H
O
4.37
2.94
1.00
0.98
1.93
1.93
1.03
2.36
1.99
2.01
4.14
1.04
1.08
f1 (ppm)

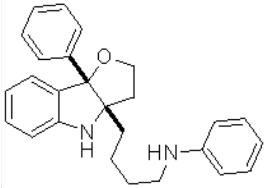

4q
Analysis: 13C NMR
Solvent: CDCl3
Field strength: 101 MHz

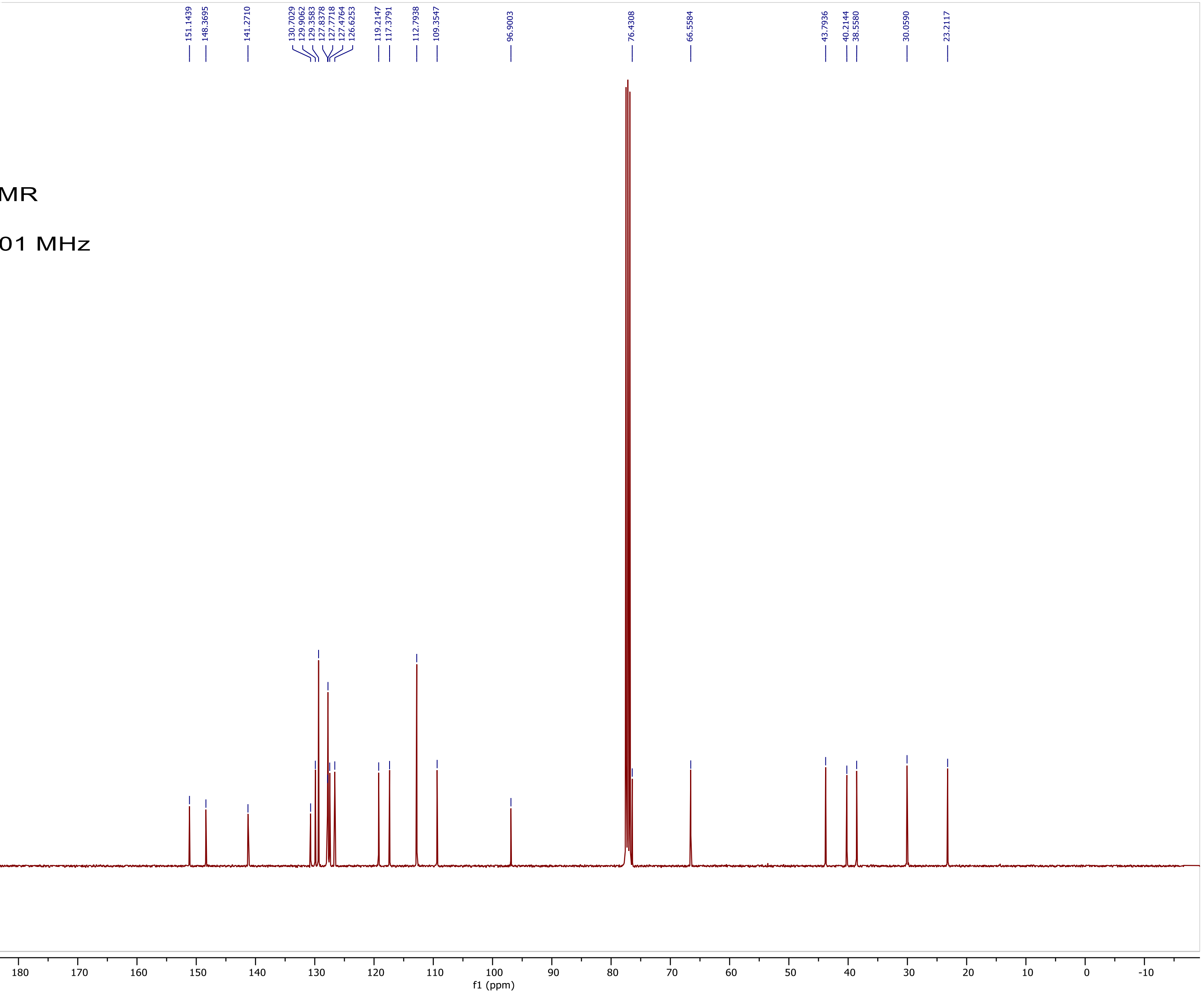

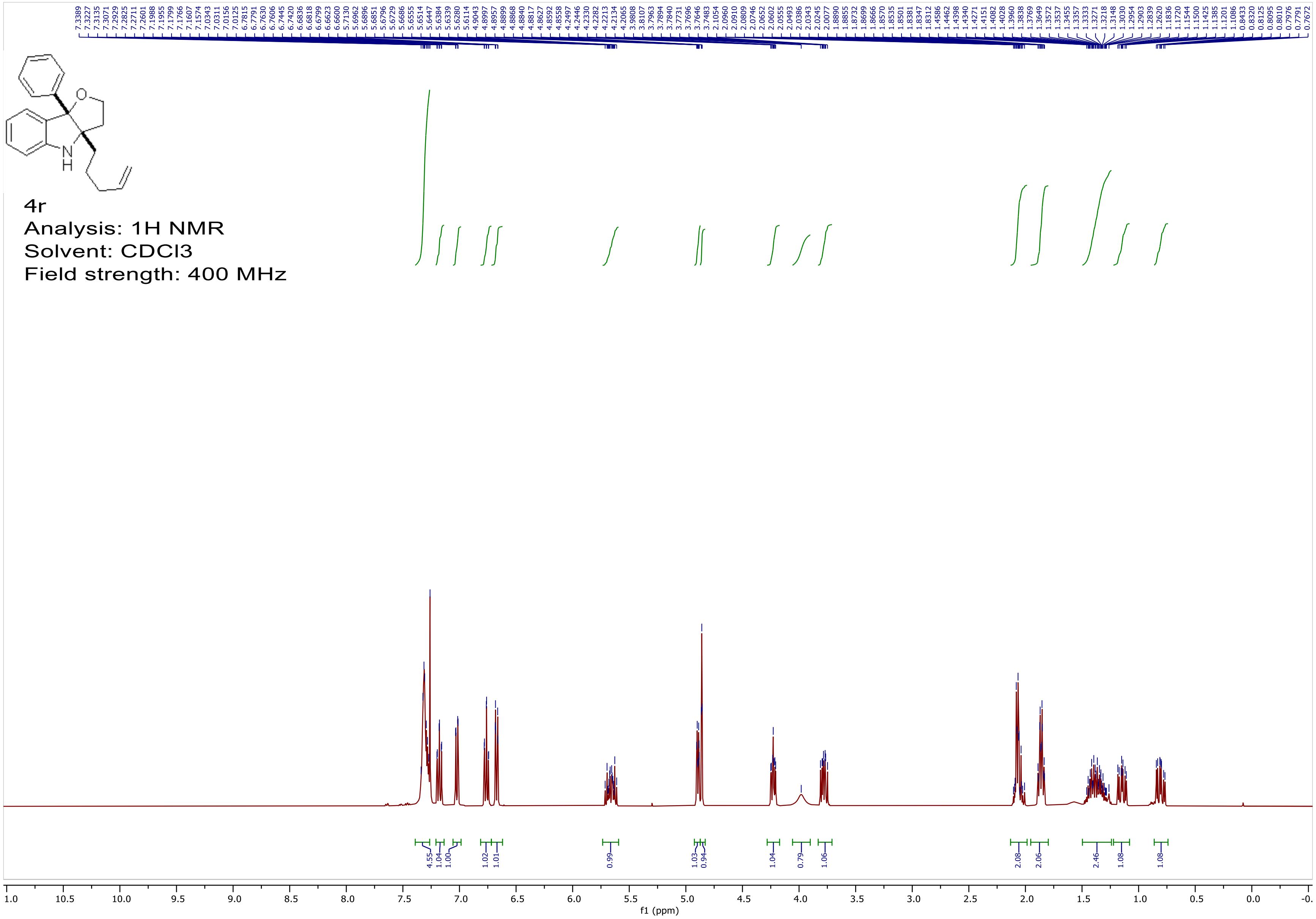
4r
Analysis: 1H NMR
Solvent: CDCl3
Field strength: 400 MHz
f1 (ppm)

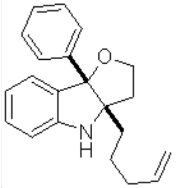

4r
Analysis: 13C NMR
Solvent: CDCl3
Field strength: 101 MHz

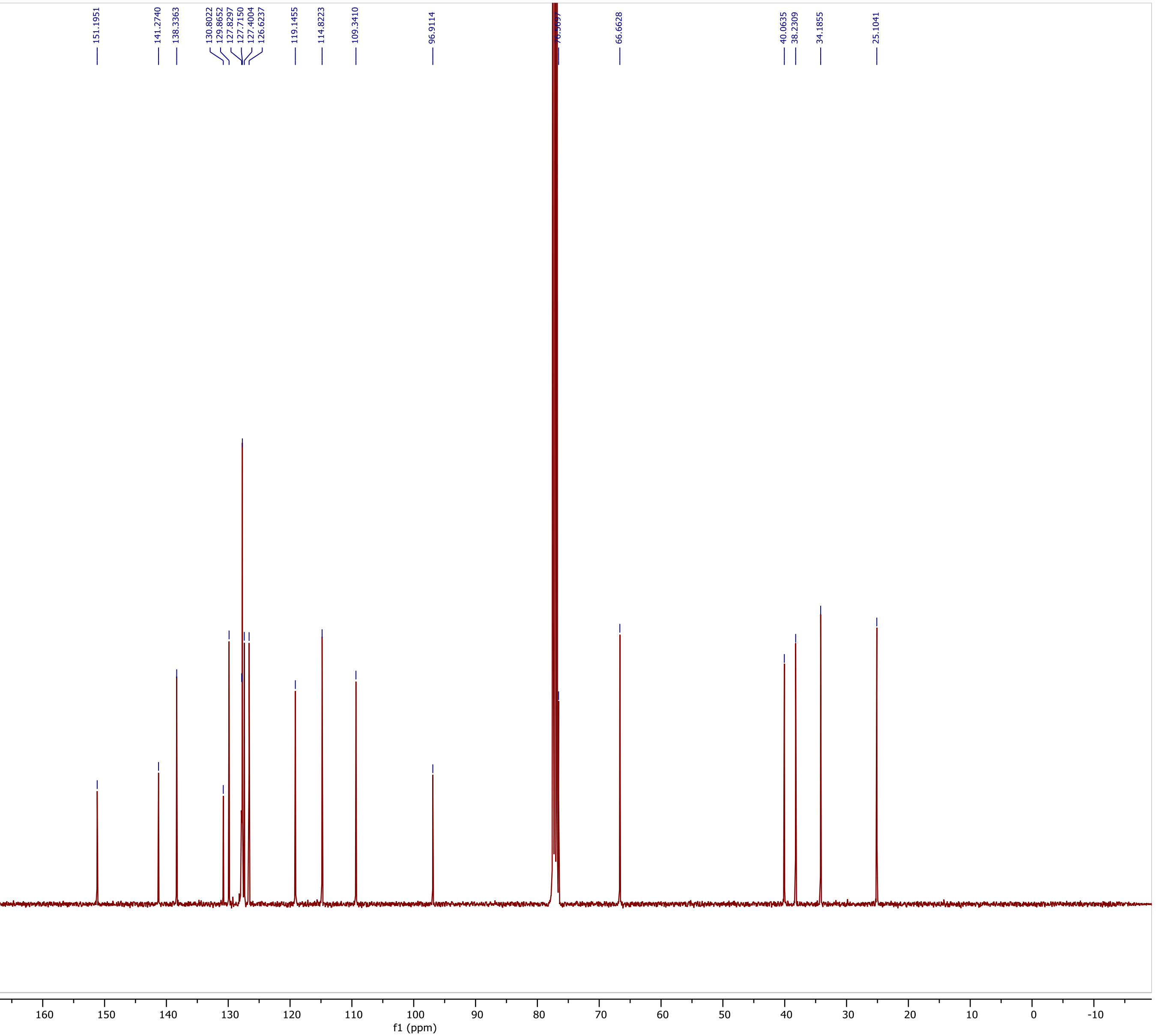

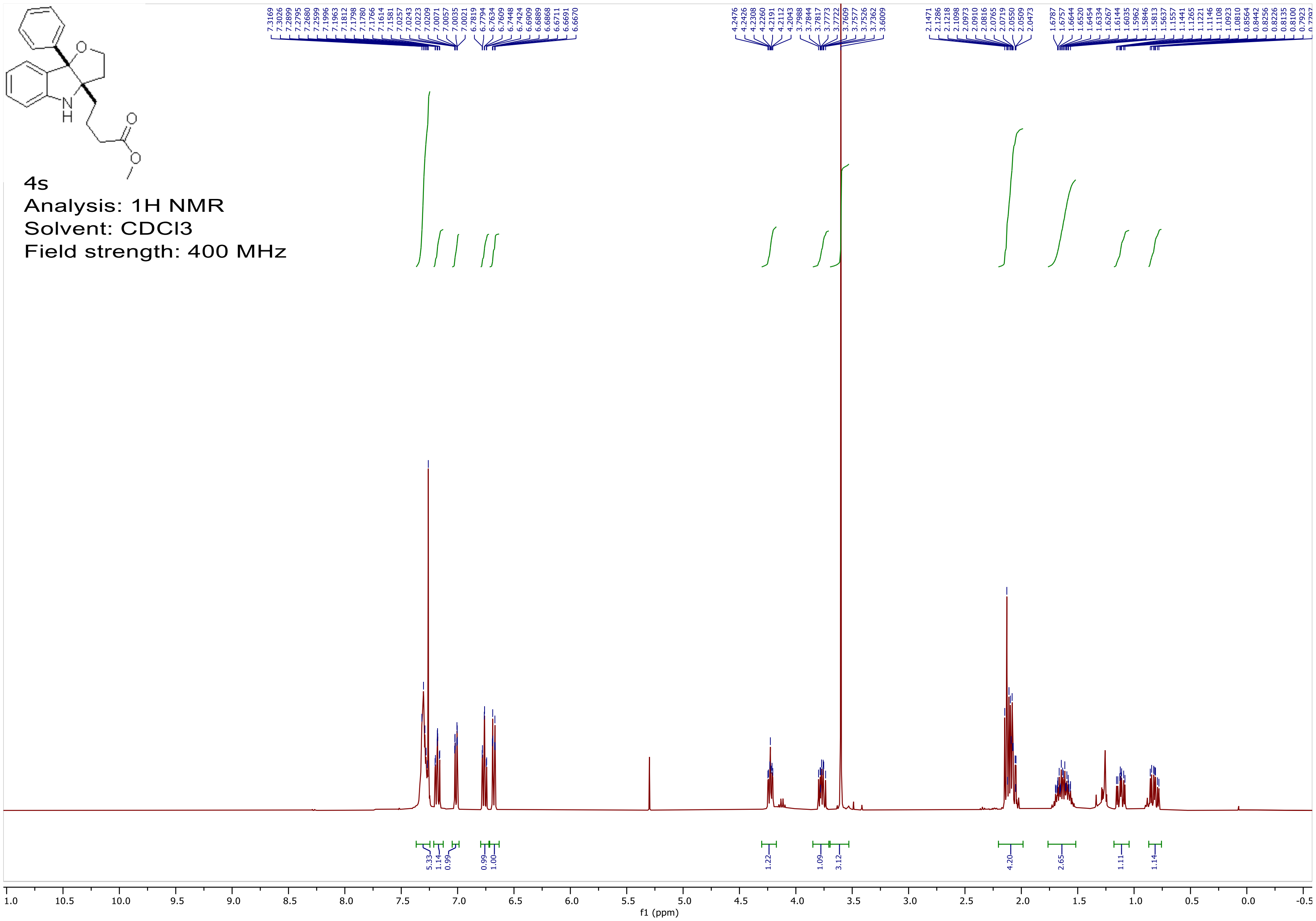

4s
Analysis: 1H NMR
Solvent: CDCl3
Field strength: 400 MHz
f1 (ppm)

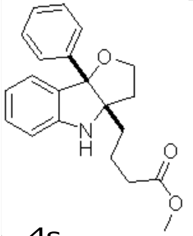

4s
Analysis: 13C NMR
Solvent: CDCl3
Field strength: 101 MHz

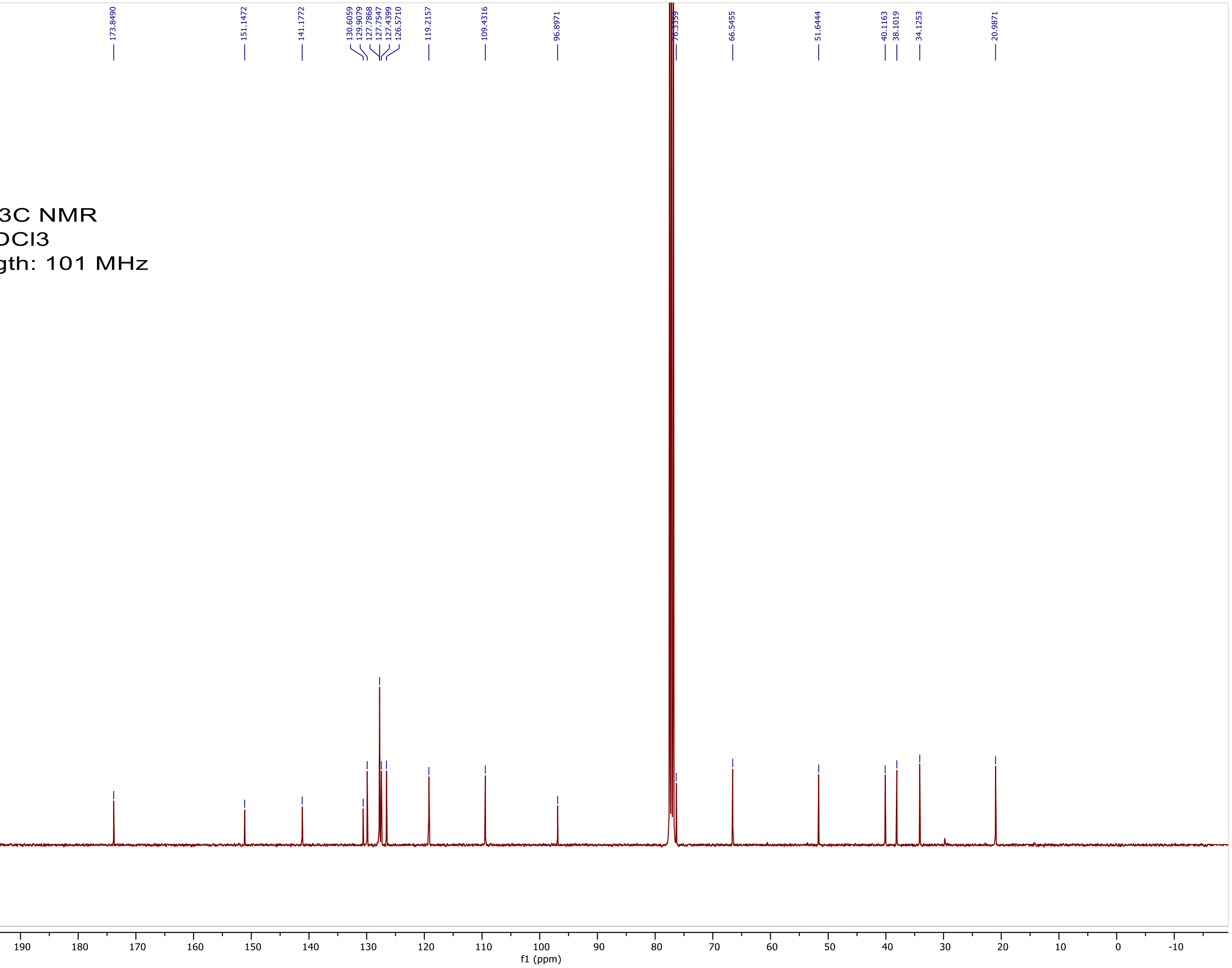

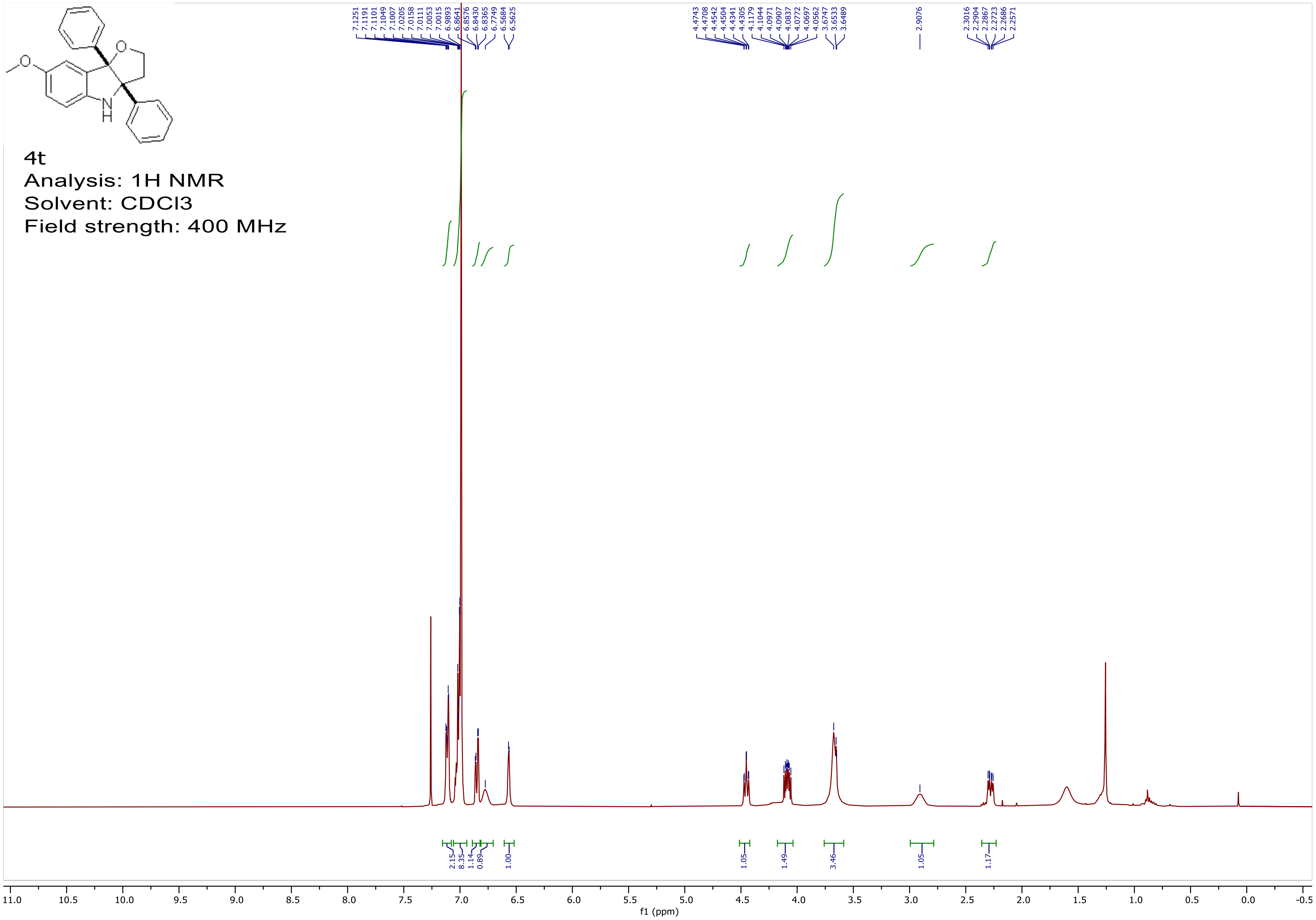
4t
Analysis: 1H NMR
Solvent: CDCl3
Field strength: 400 MHz
f1 (ppm)

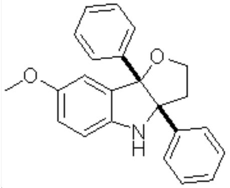

4t
Analysis: 13C NMR
Solvent: CDCl3
Field strength: 101 MHz

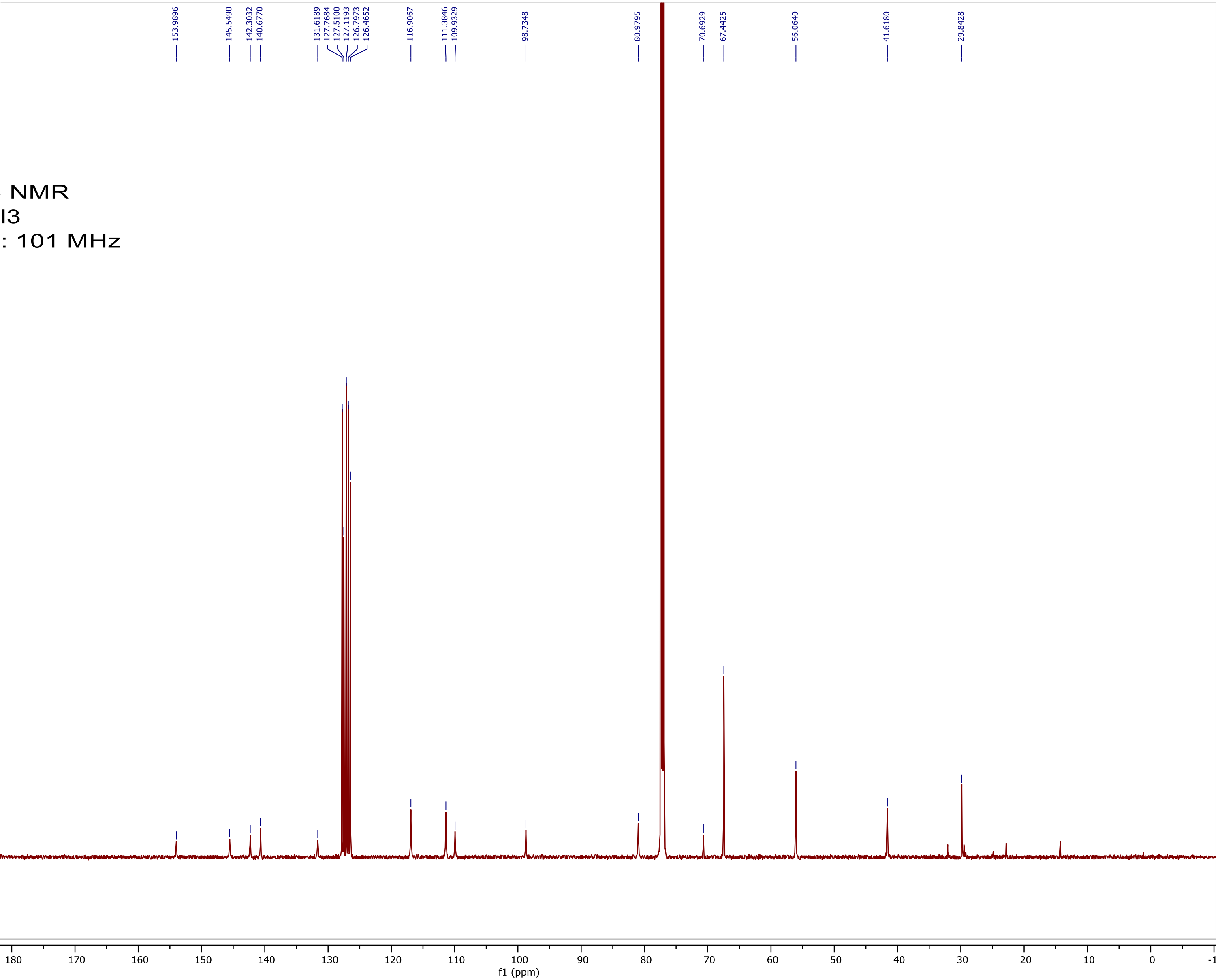

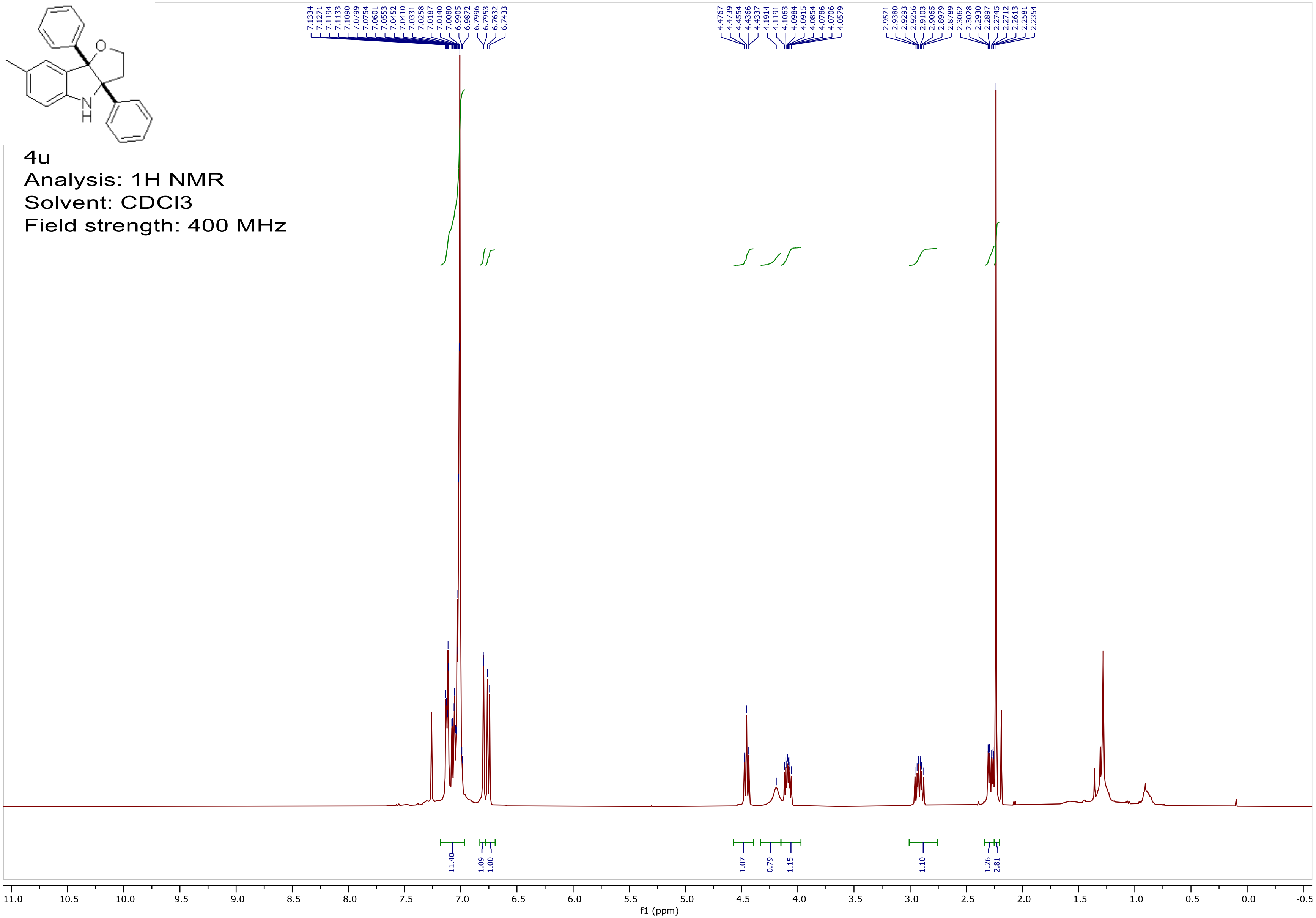

4u
Analysis: 1H NMR
Solvent: CDCl3
Field strength: 400 MHz
7.1334
7.1271
7.1194
7.1133
7.1090
7.0799
7.0754
7.0601
7.0553
7.0452
7.0410
7.0331
7.0258
7.0187
7.0140
7.0080
6.9905
6.9872
6.7996
6.7953
6.7632
6.7433
4.4767
4.4739
4.4554
4.4366
4.4337
4.1914
4.1191
4.1063
4.0984
4.0915
4.0854
4.0786
4.0706
4.0579
2.9571
2.9380
2.9293
2.9256
2.9103
2.9065
2.8979
2.8789
2.3062
2.3028
2.2930
2.2897
2.2745
2.2712
2.2613
2.2581
2.2354
11.40
1.09
1.00
1.07
0.79
1.15
1.10
1.26
2.81
f1 (ppm)

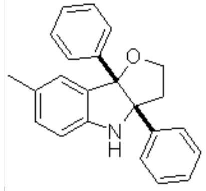

4u
Analysis: 13C NMR
Solvent: CDCl3
Field strength: 101 MHz

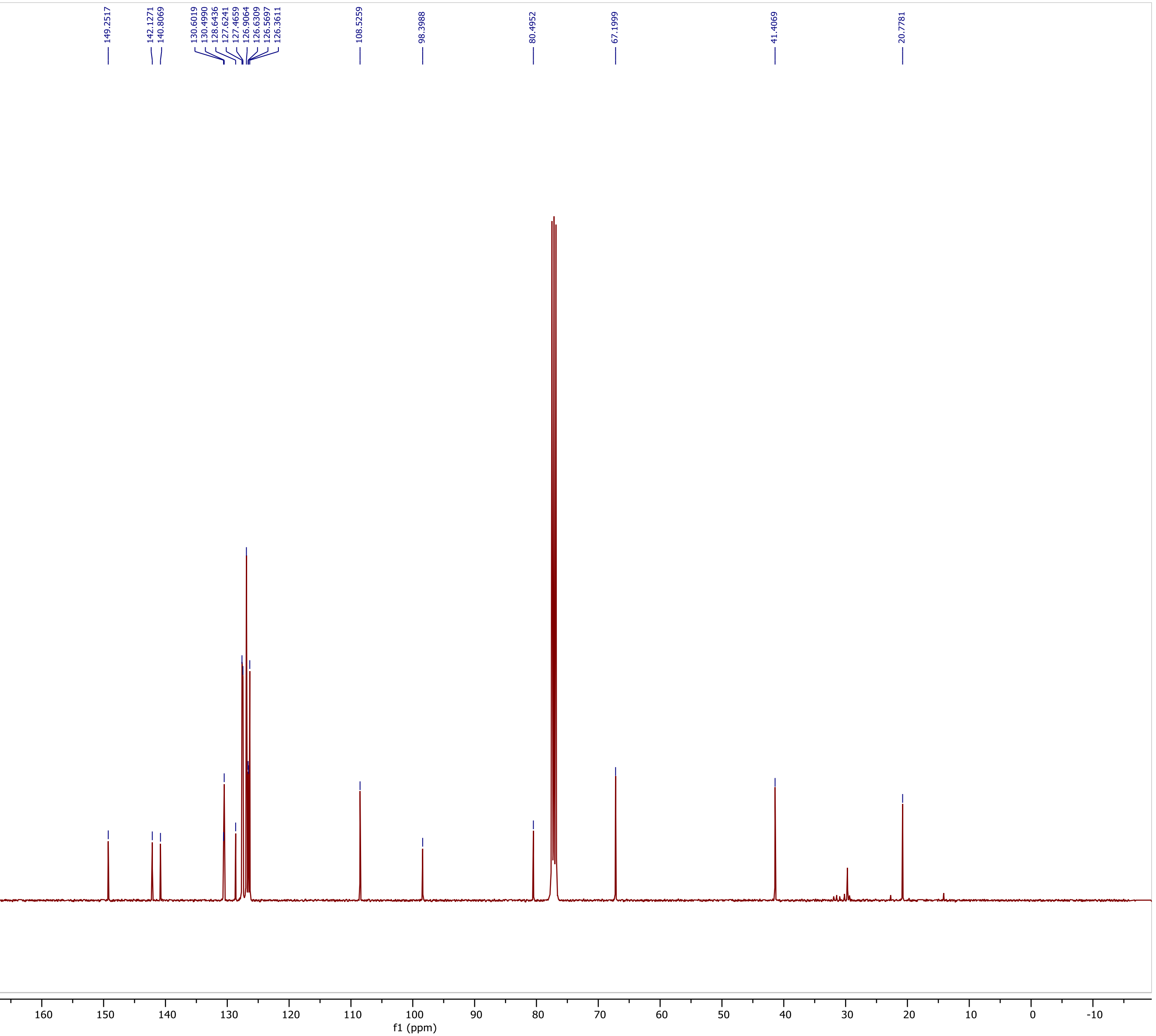

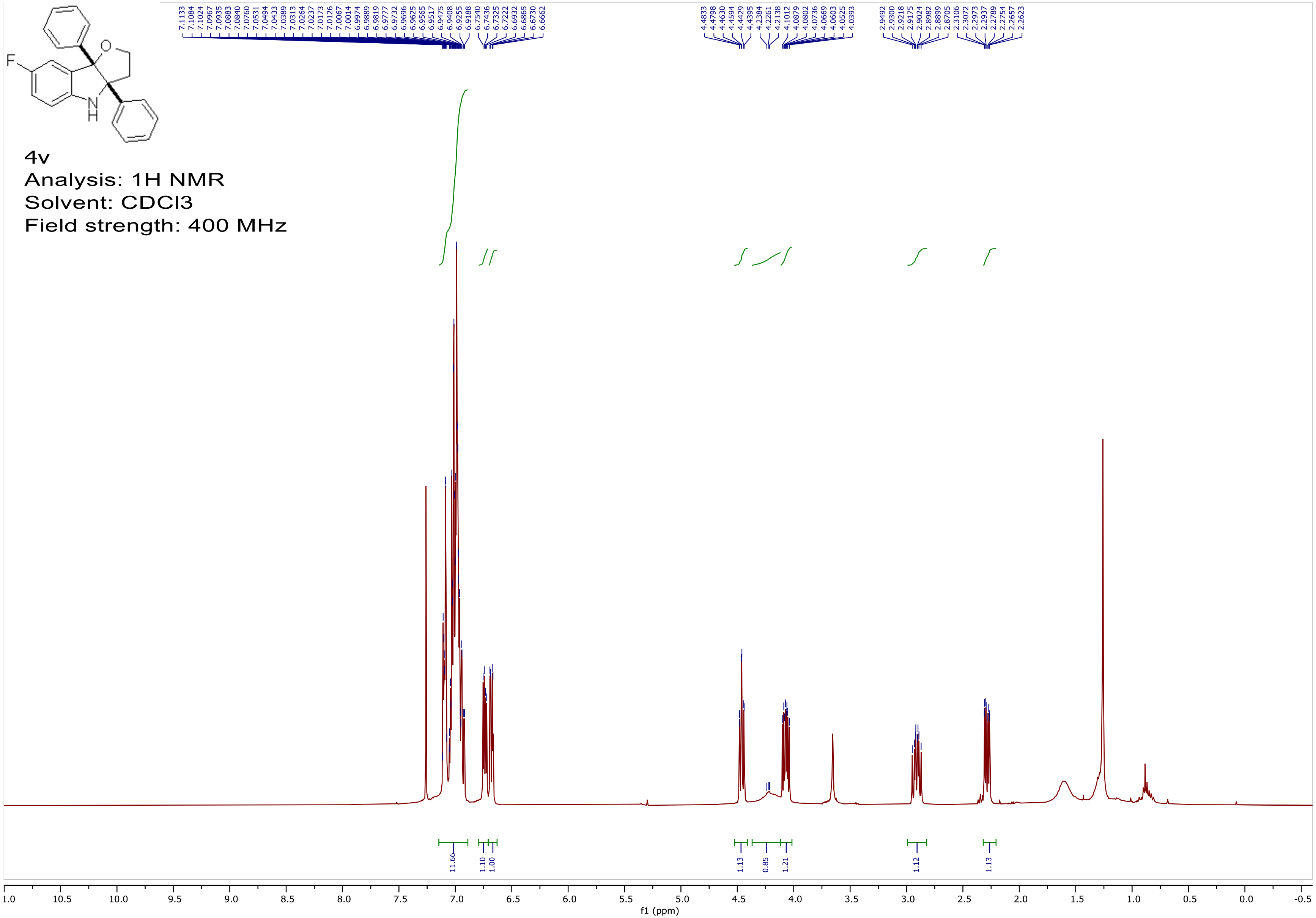
F
O
N
H
4v
Analysis: 1H NMR
Solvent: CDCl3
Field strength: 400 MHz
11.66
1.10
1.00
1.13
0.85
1.21
1.12
1.13
f1 (ppm)

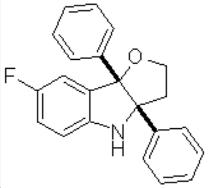

4v
Analysis: 13C NMR
Solvent: CDCl3
Field strength: 101 MHz

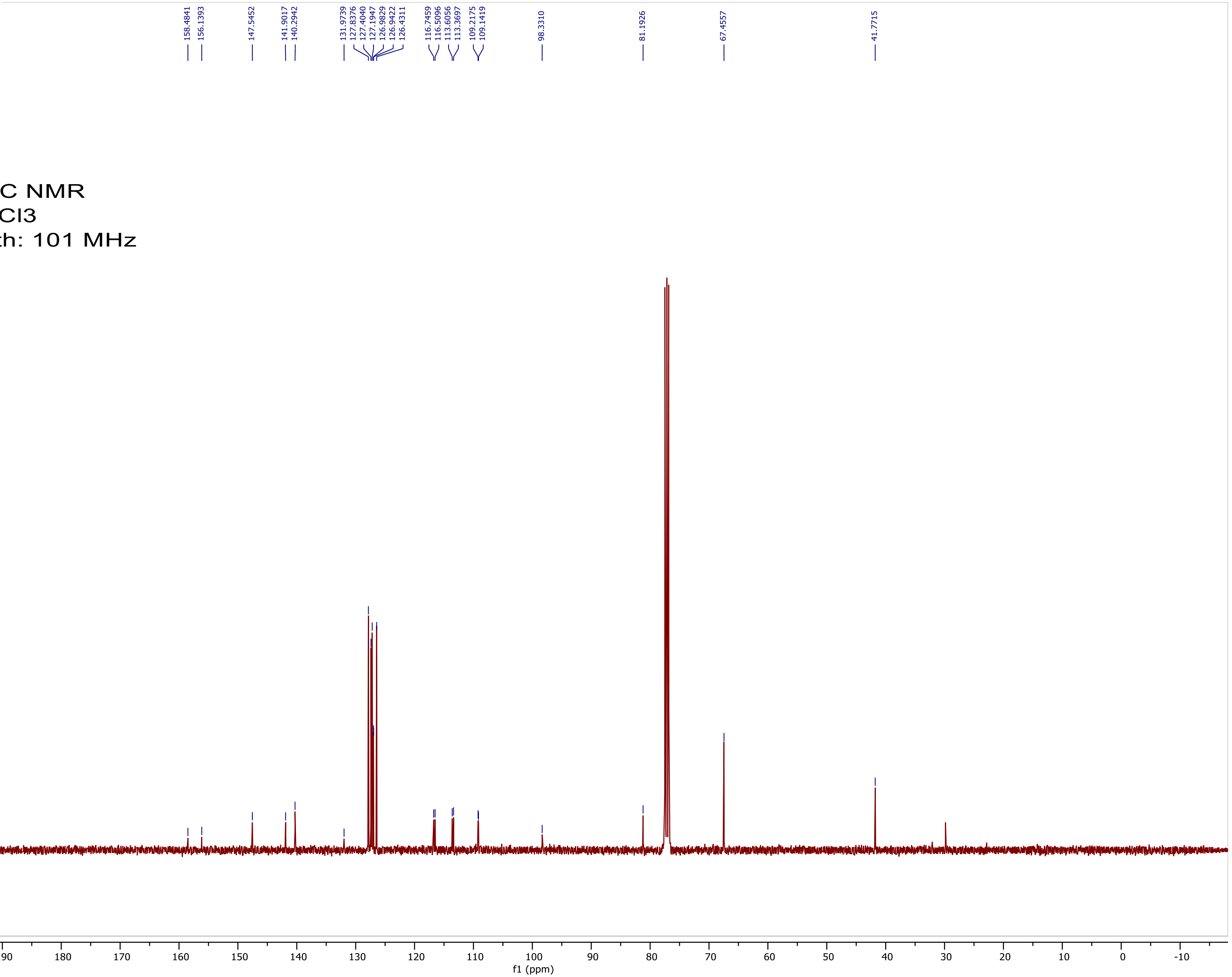

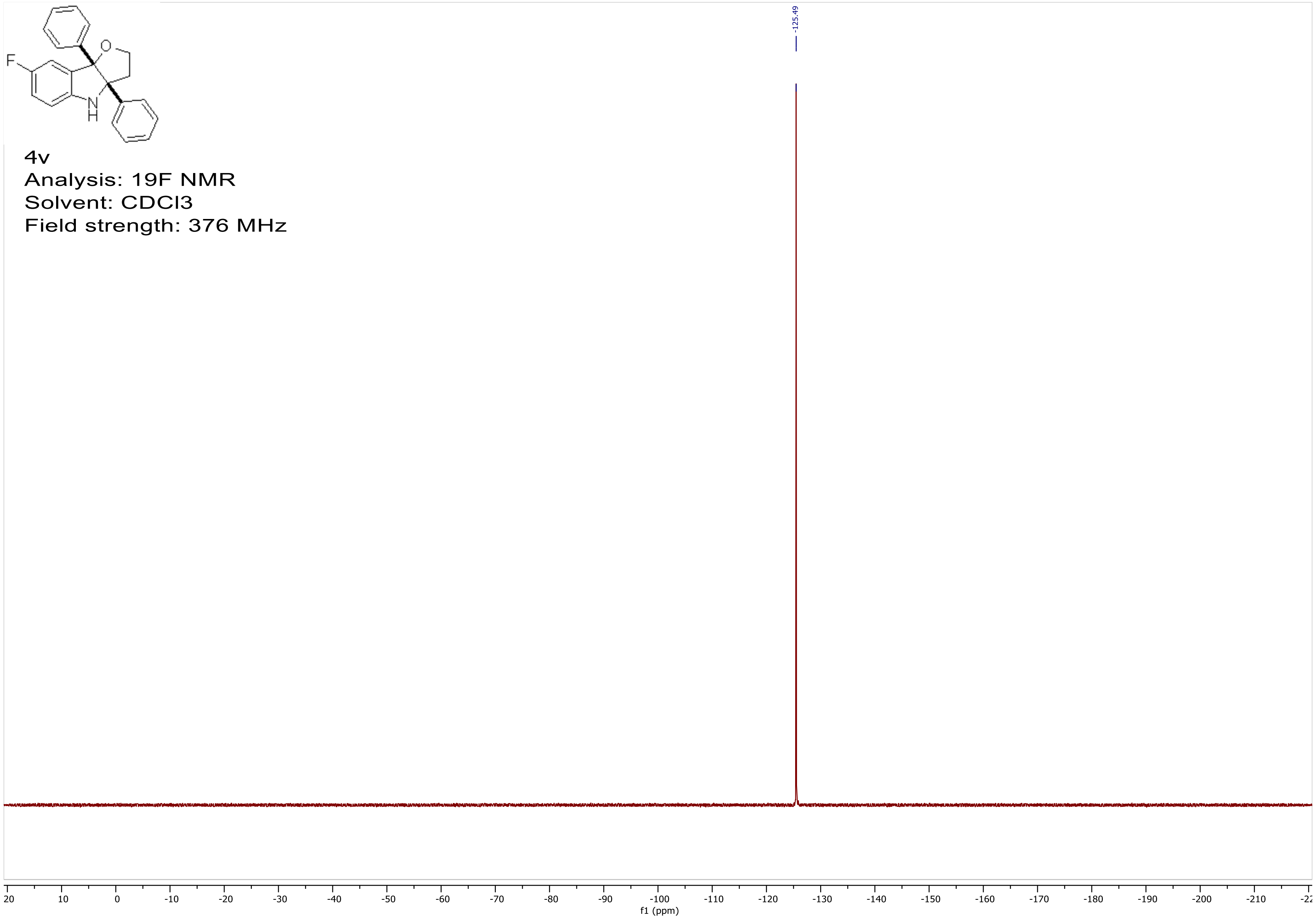
4v
Analysis: 19F NMR
Solvent: CDCl3
Field strength: 376 MHz
-125.49
f1 (ppm)
20 10 0 -10 -20 -30 -40 -50 -60 -70 -80 -90 -100 -110 -120 -130 -140 -150 -160 -170 -180 -190 -200 -210

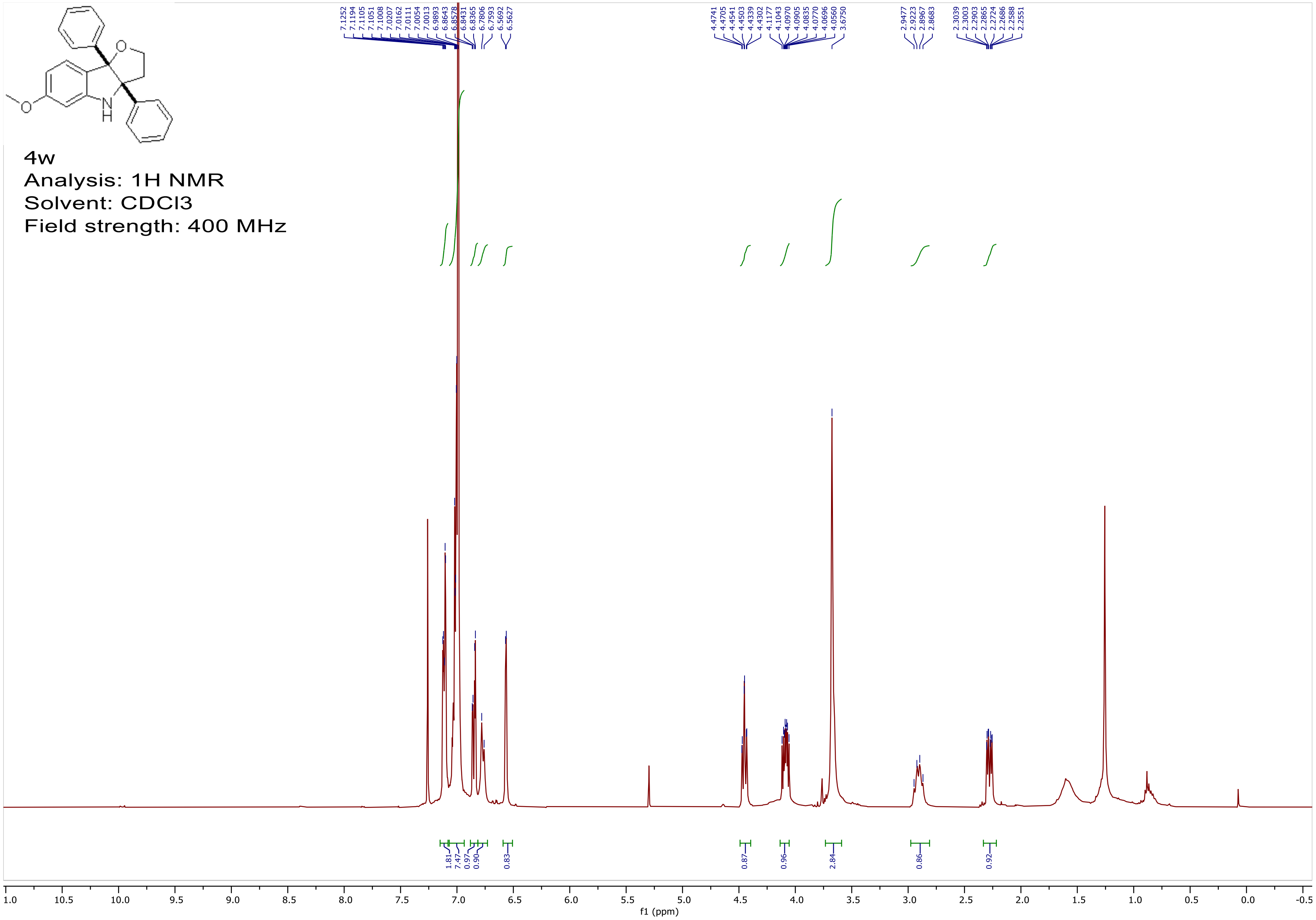
O
N
H
4w
Analysis: 1H NMR
Solvent: CDCl3
Field strength: 400 MHz
7.1252
7.1194
7.1105
7.1051
7.1008
7.0207
7.0162
7.0111
7.0054
7.0013
6.9893
6.8643
6.8578
6.8431
6.8365
6.7806
6.7593
6.5692
6.5627
4.4741
4.4705
4.4541
4.4503
4.4339
4.4302
4.1177
4.1043
4.0970
4.0905
4.0835
4.0770
4.0696
4.0560
3.6750
2.9477
2.9223
2.8967
2.8683
2.3039
2.3003
2.2903
2.2865
2.2724
2.2686
2.2588
2.2551
1.81
7.47
0.97
0.90
0.83
0.87
0.96
2.84
0.86
0.92
1.0 10.5 10.0 9.5 9.0 8.5 8.0 7.5 7.0 6.5 6.0 5.5 5.0 4.5 4.0 3.5 3.0 2.5 2.0 1.5 1.0 0.5 0.0 -0.5
f1 (ppm)

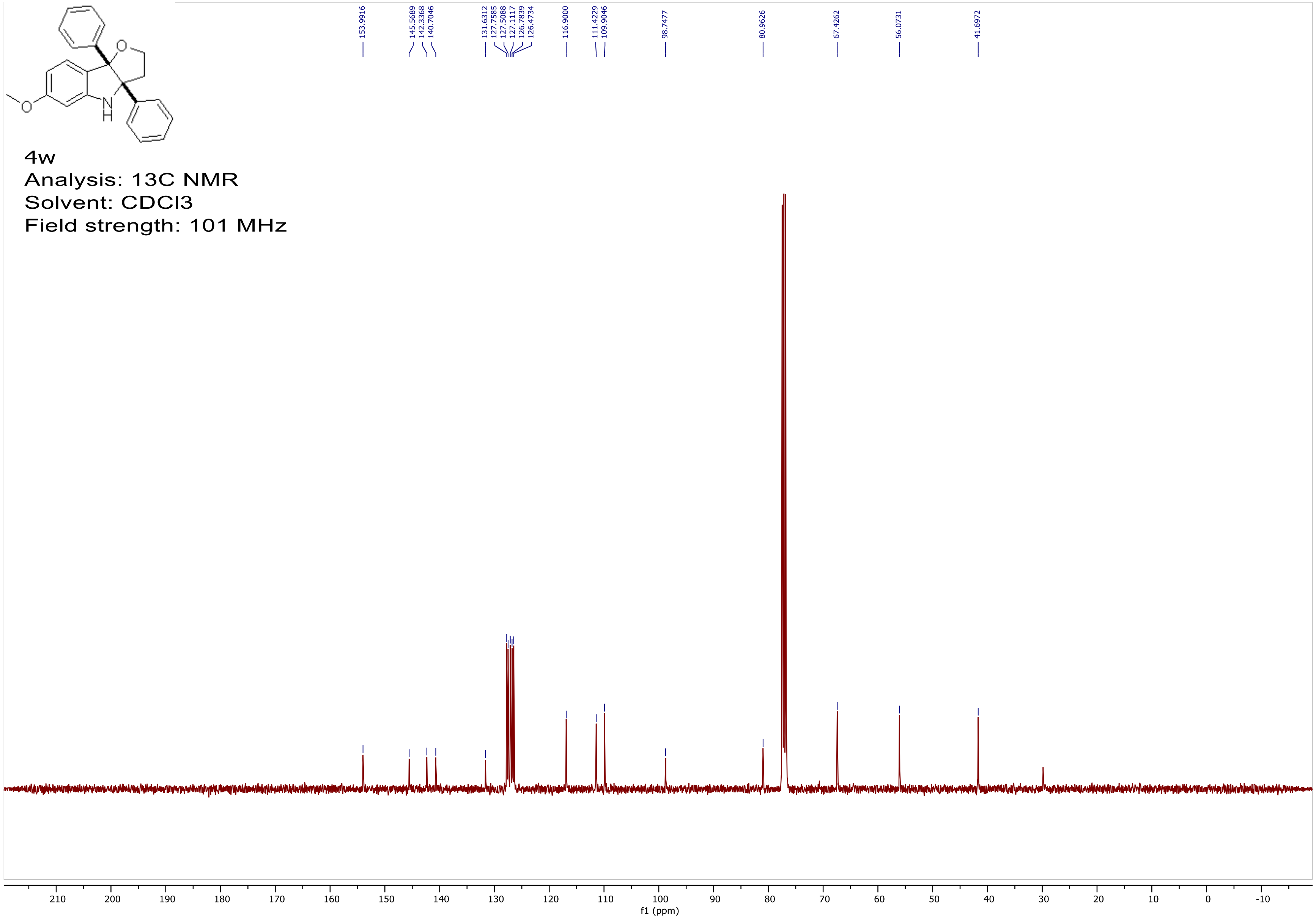
4w
Analysis: 13C NMR
Solvent: CDCl3
Field strength: 101 MHz
153.9916
145.5689
142.3368
140.7046
131.6312
127.7585
127.5088
127.1117
126.7839
126.4734
116.9000
111.4229
109.9046
98.7477
80.9626
67.4262
56.0731
41.6972
210 200 190 180 170 160 150 140 130 120 110 100 90 80 70 60 50 40 30 20 10 0 -10
f1 (ppm)

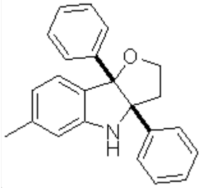

4x
Analysis: 1H NMR
Solvent: CDCl3
Field strength: 400 MHz

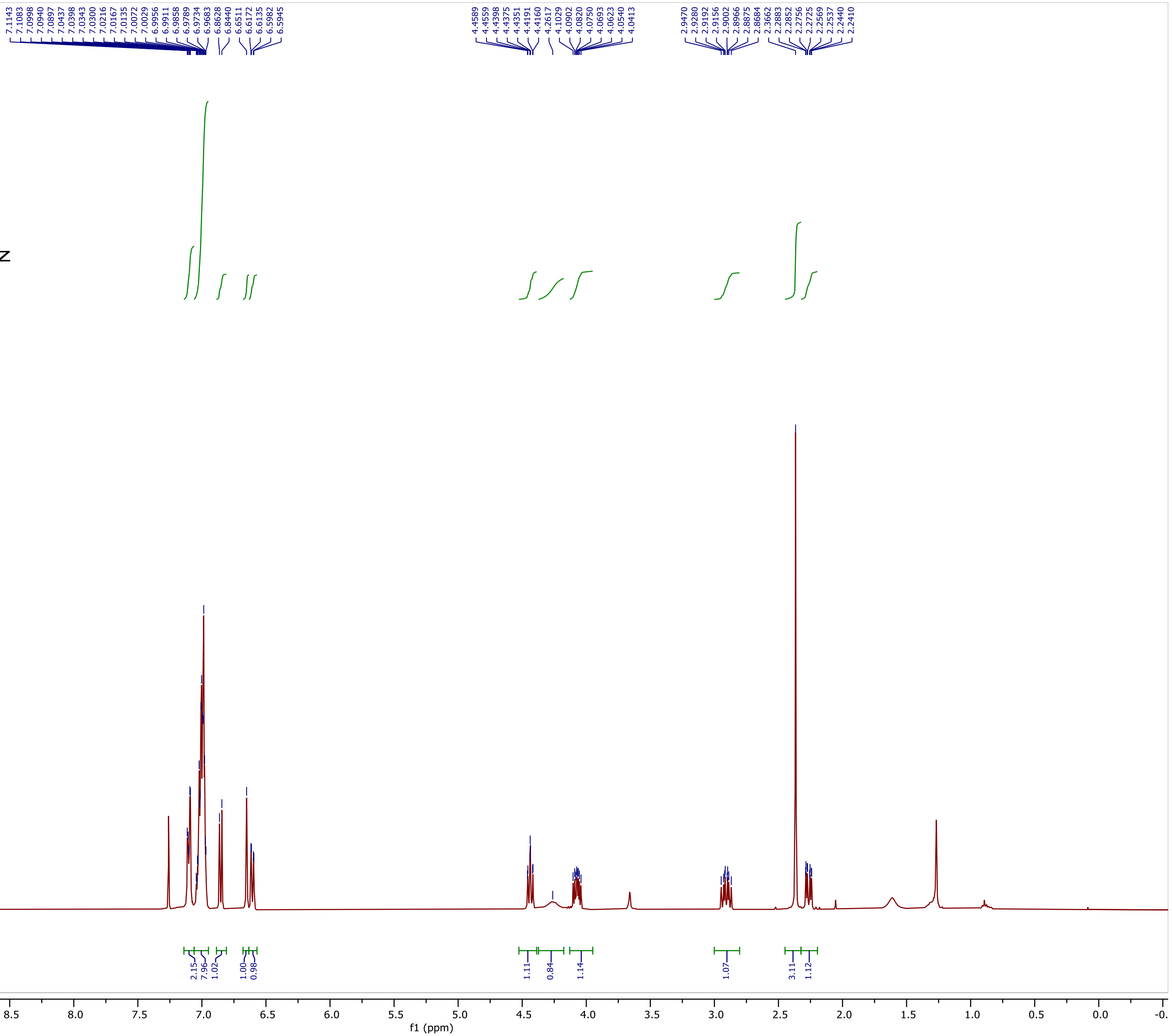

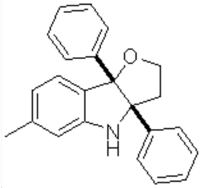

4x
Analysis: 13C NMR
Solvent: CDCl3
Field strength: 101 MHz

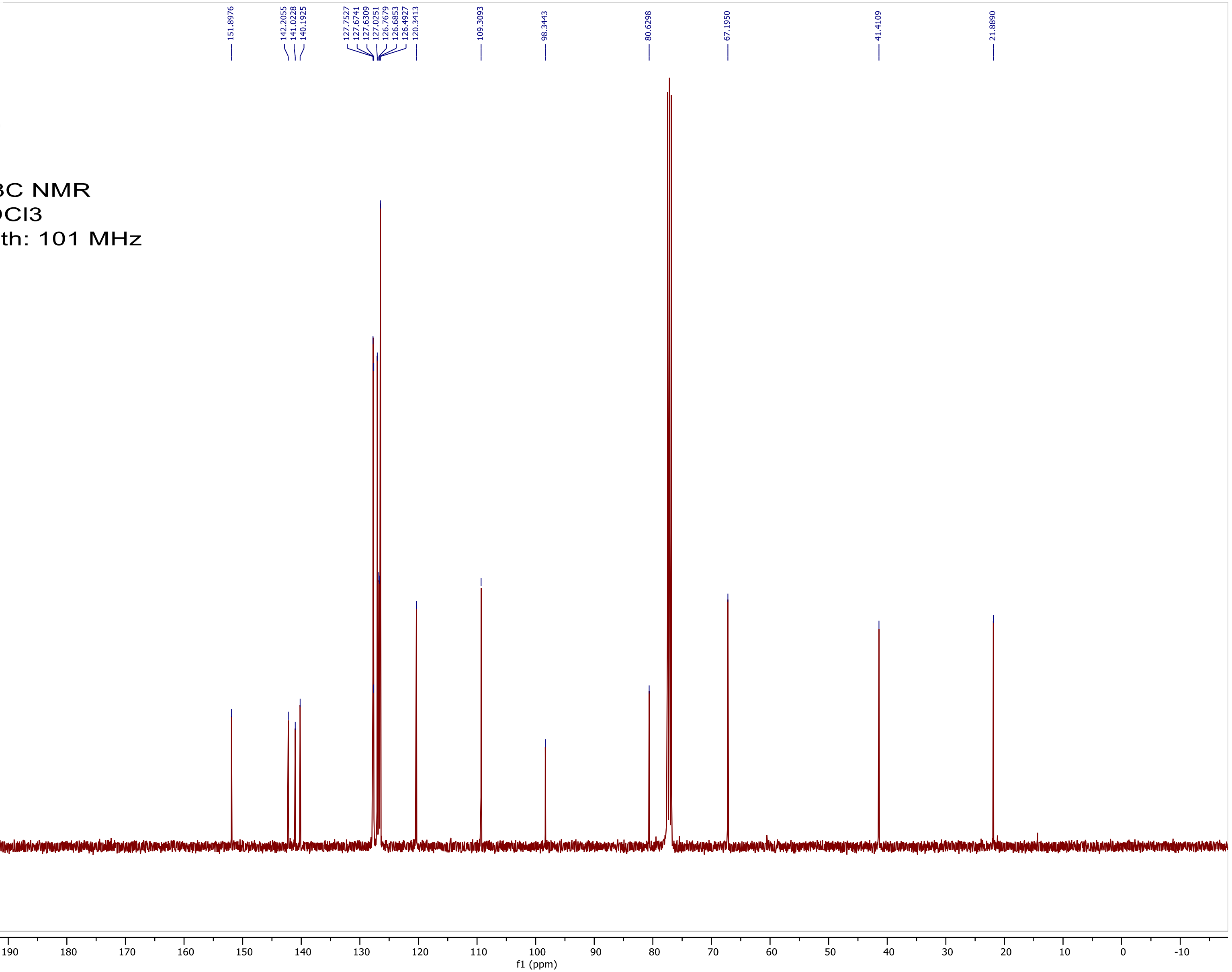

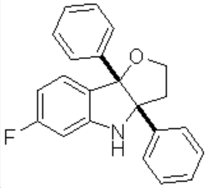

4y
Analysis: 1H NMR
Solvent: CDCl3
Field strength: 400 MHz

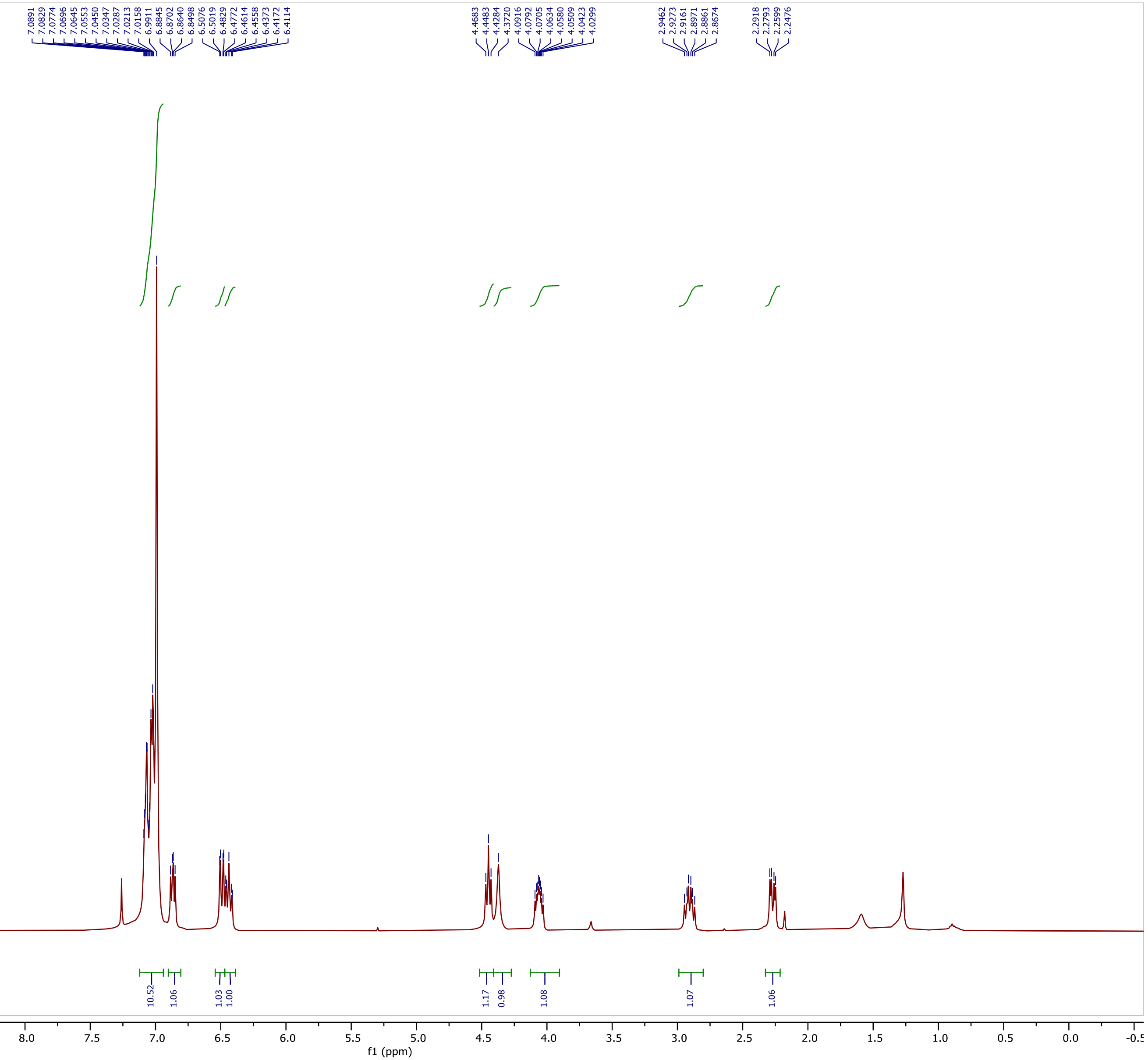

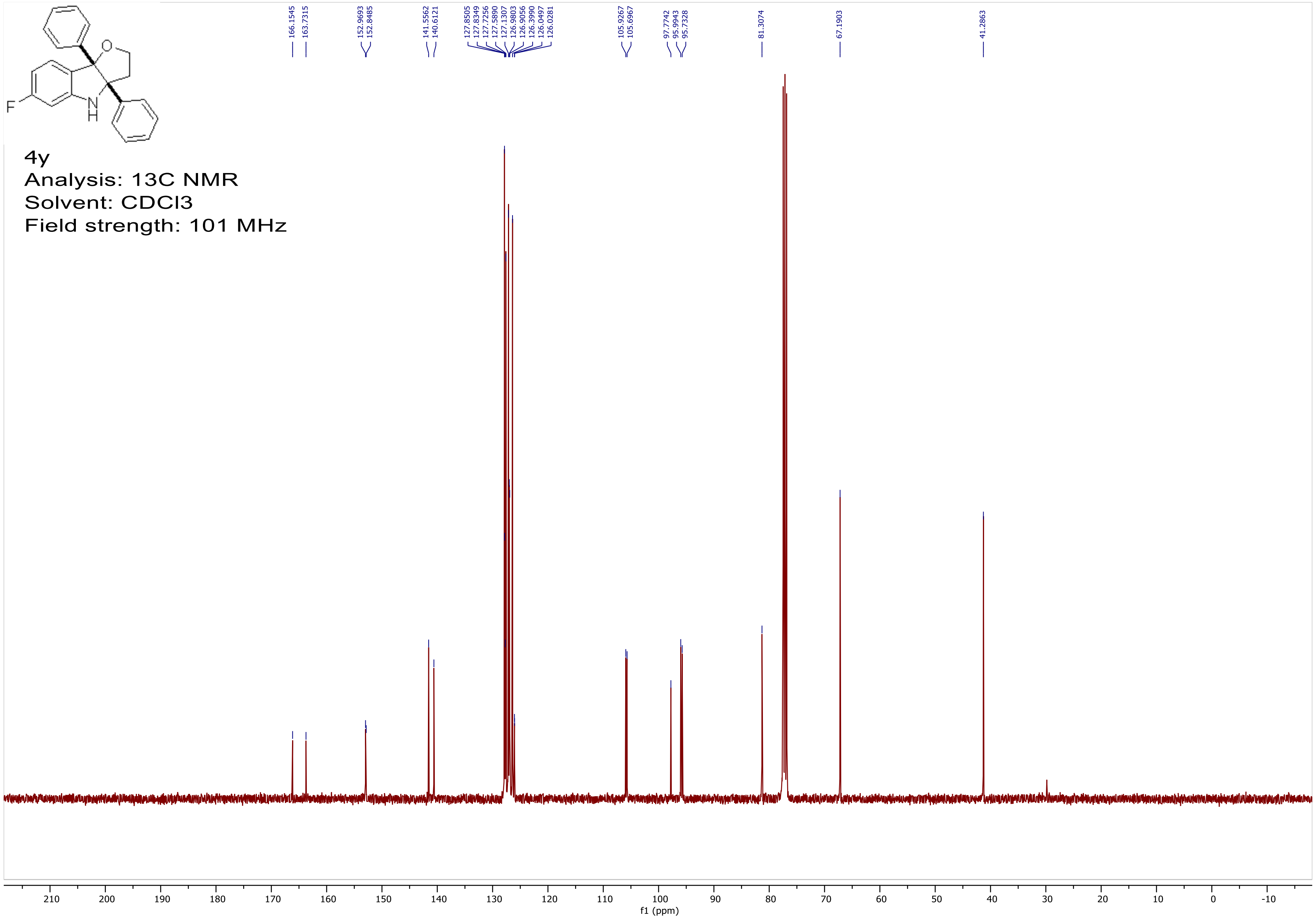
F
N
H
O
4y
Analysis: 13C NMR
Solvent: CDCl3
Field strength: 101 MHz
166.1545
163.7315
152.9693
152.8485
141.5562
140.6121
127.8505
127.8349
127.7256
127.5890
127.1307
126.9803
126.9056
126.3990
126.0497
126.0281
105.9267
105.6967
97.7742
95.9943
95.7328
81.3074
67.1903
41.2863
210
200
190
180
170
160
150
140
130
120
110
100
90
80
70
60
50
40
30
20
10
0
-10
f1 (ppm)

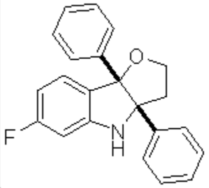

4y
Analysis: 19F NMR
Solvent: CDCl3
Field strength: 376 MHz

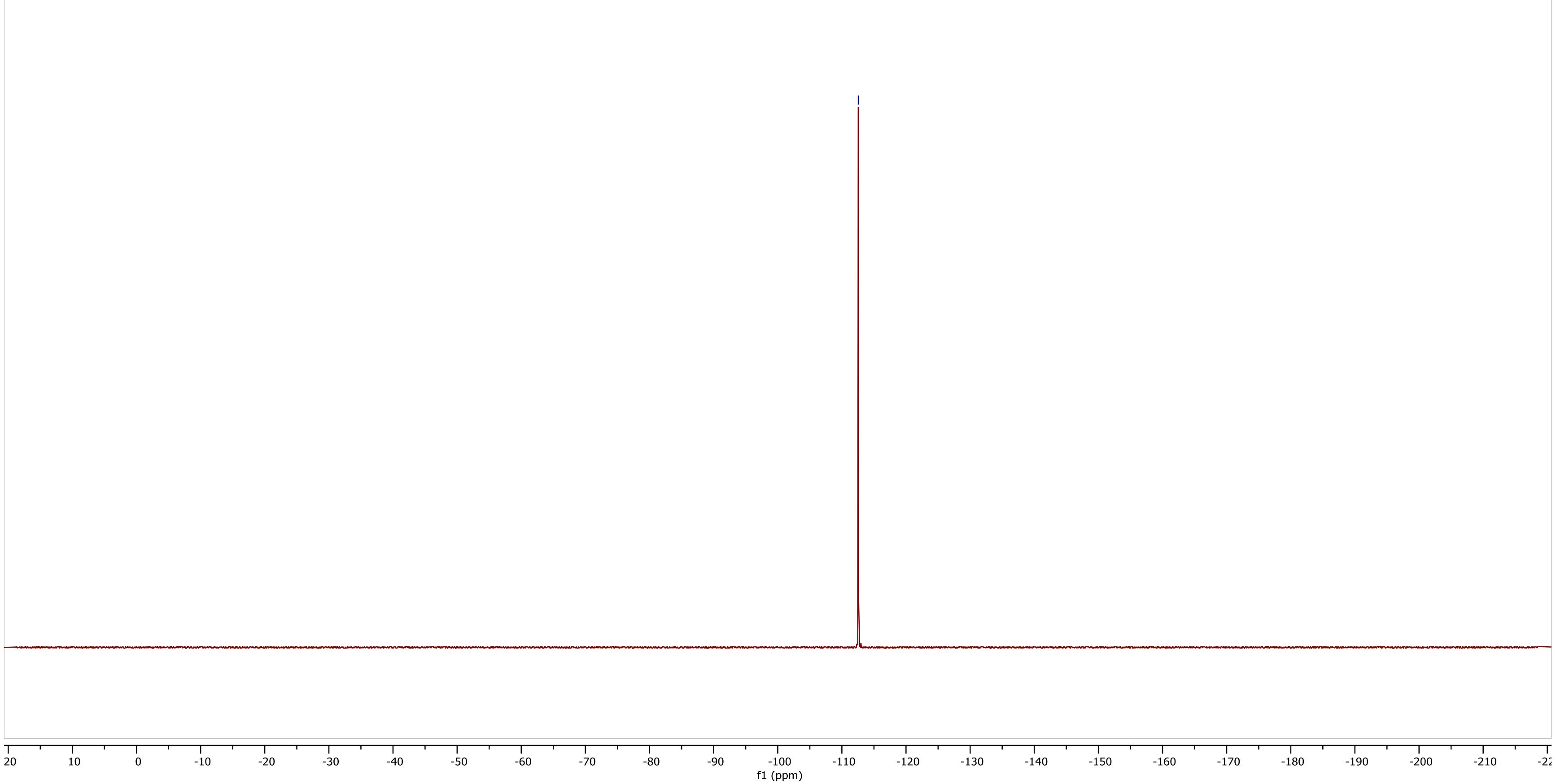

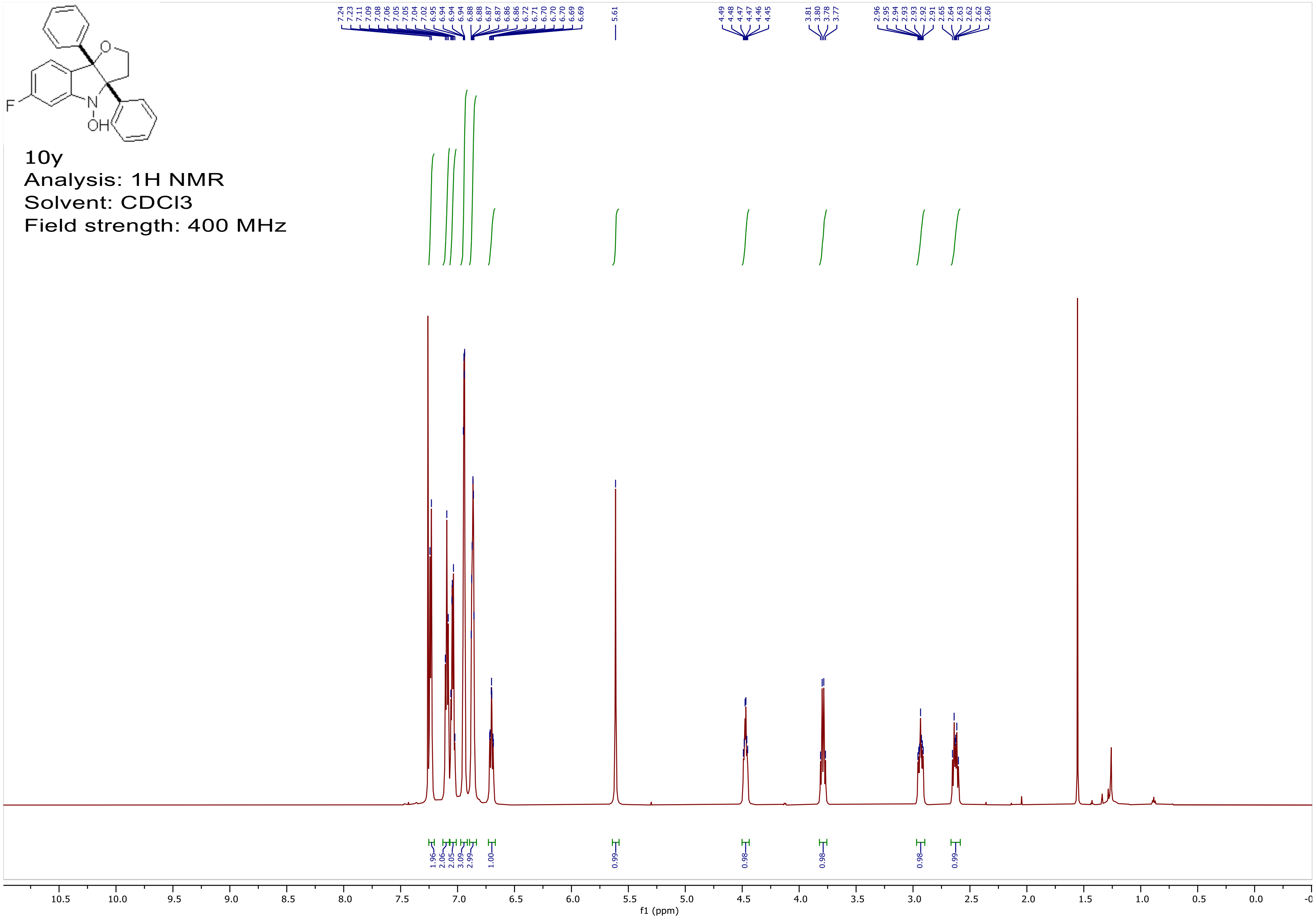
F
N
OH
O
10y
Analysis: 1H NMR
Solvent: CDCl3
Field strength: 400 MHz
7.24 7.23 7.11 7.09 7.08 7.06 7.05 7.05 7.04 7.02 6.95 6.94 6.94 6.94 6.94 6.88 6.87 6.87 6.86 6.86 6.72 6.71 6.70 6.70 6.69 6.69
5.61
4.49 4.48 4.47 4.47 4.46 4.45
3.81 3.80 3.78 3.77
2.96 2.95 2.94 2.93 2.93 2.92 2.91 2.65 2.64 2.63 2.62 2.62 2.60
1.96 2.06 2.05 3.09 2.99 1.00
0.99
0.98
0.98
0.98
0.99
10.5 10.0 9.5 9.0 8.5 8.0 7.5 7.0 6.5 6.0 5.5 5.0 4.5 4.0 3.5 3.0 2.5 2.0 1.5 1.0 0.5 0.0
f1 (ppm)

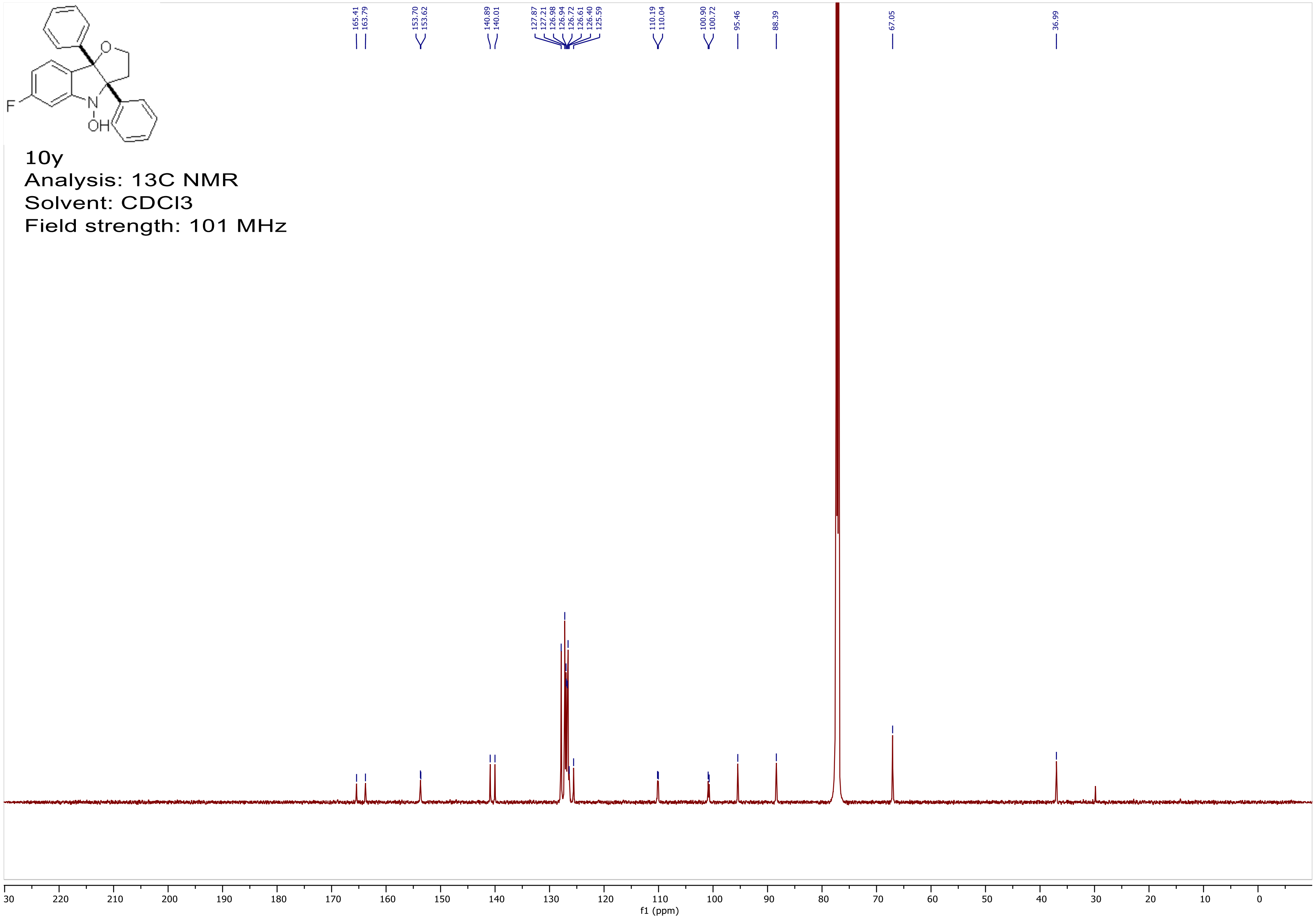
10y
Analysis: 13C NMR
Solvent: CDCl3
Field strength: 101 MHz
165.41
163.79
153.70
153.62
140.89
140.01
127.87
127.21
126.98
126.94
126.72
126.61
126.40
125.59
110.19
110.04
100.90
100.72
95.46
88.39
67.05
36.99
f1 (ppm)

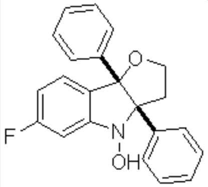

10y
Analysis: 19F NMR
Solvent: CDCl3
Field strength: 376 MHz

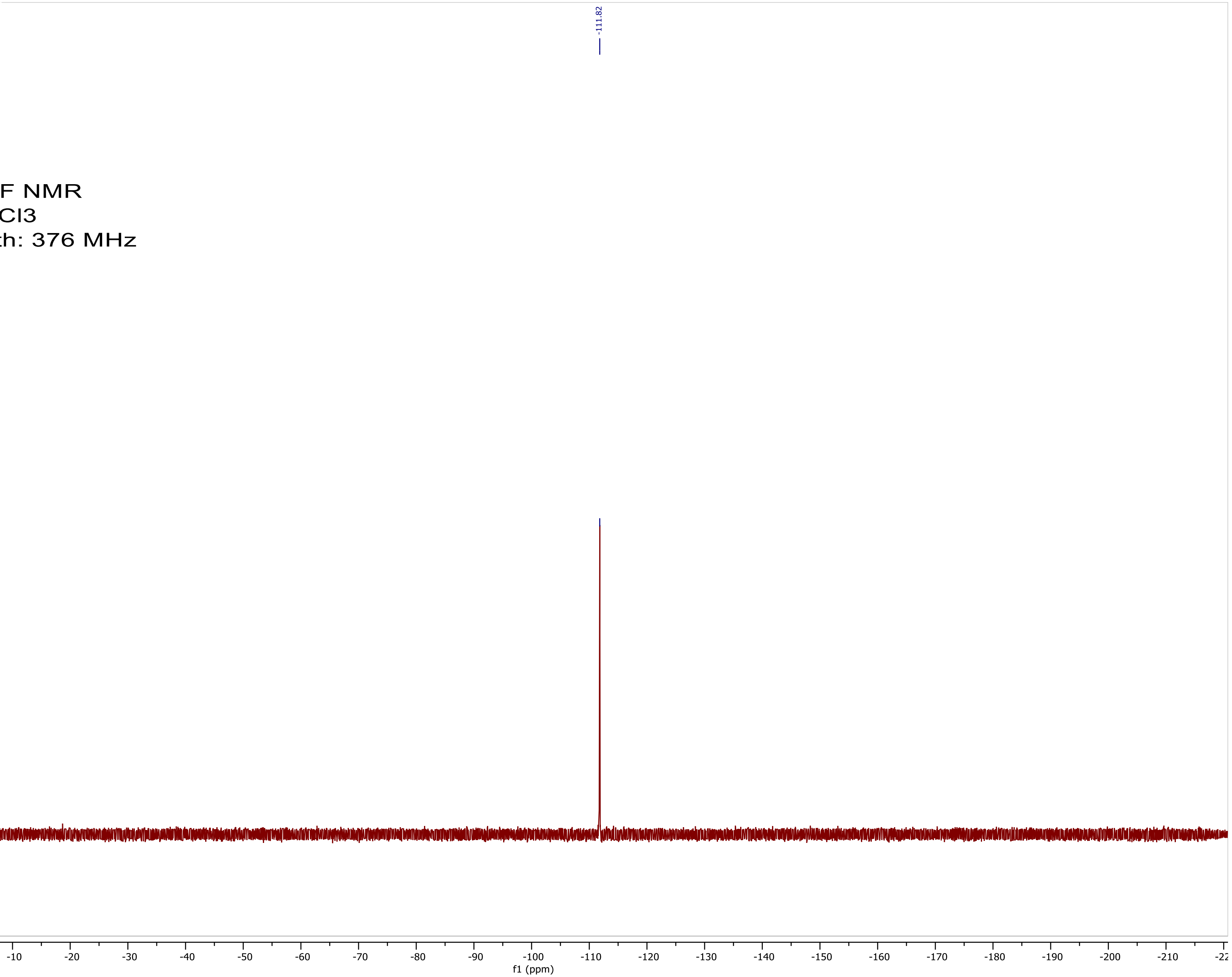

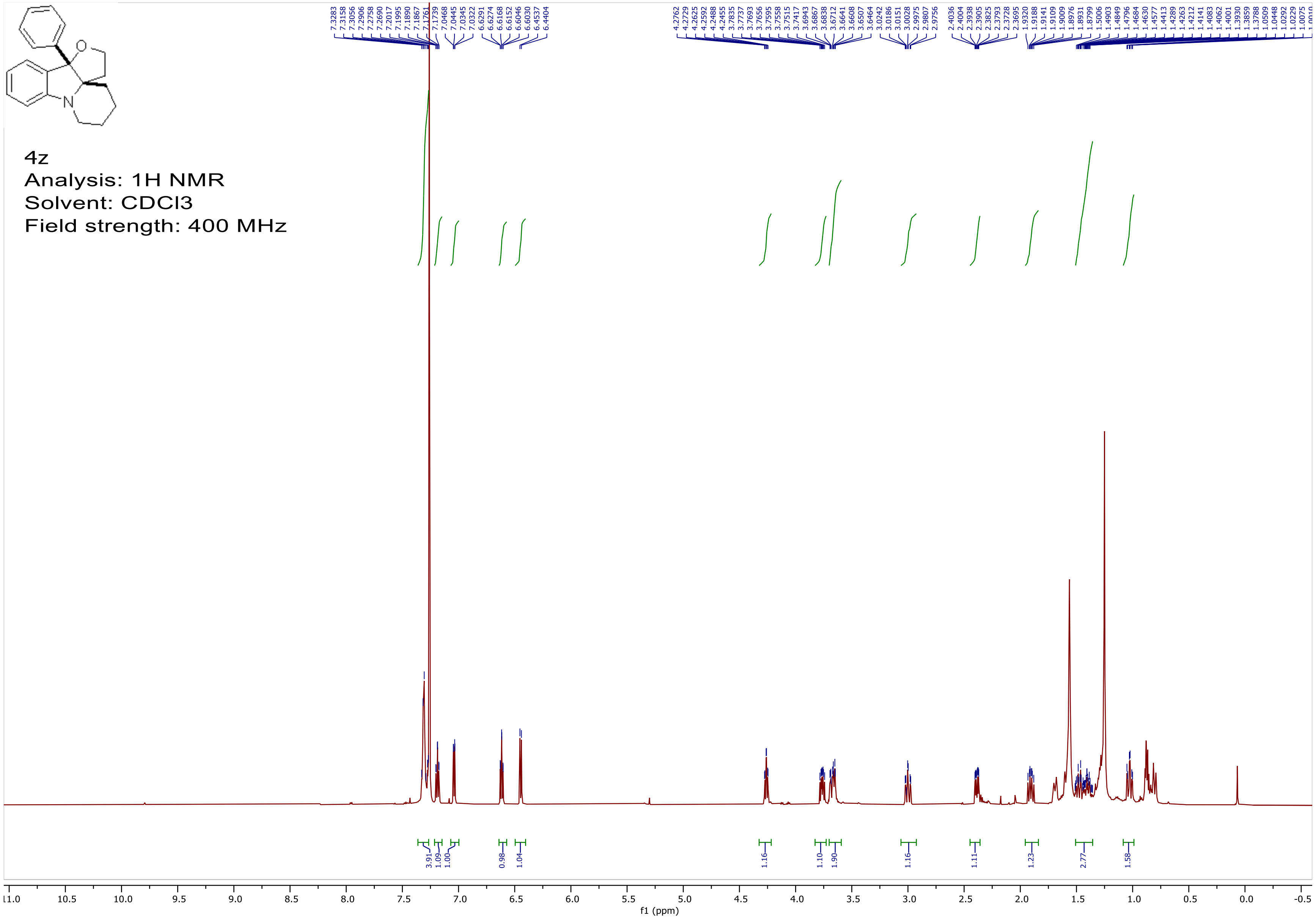
4z
Analysis: 1H NMR
Solvent: CDCl3
Field strength: 400 MHz
N
O
3.91
1.09
1.00
0.98
1.04
1.16
1.10
1.90
1.16
1.11
1.23
2.77
1.58
f1 (ppm)

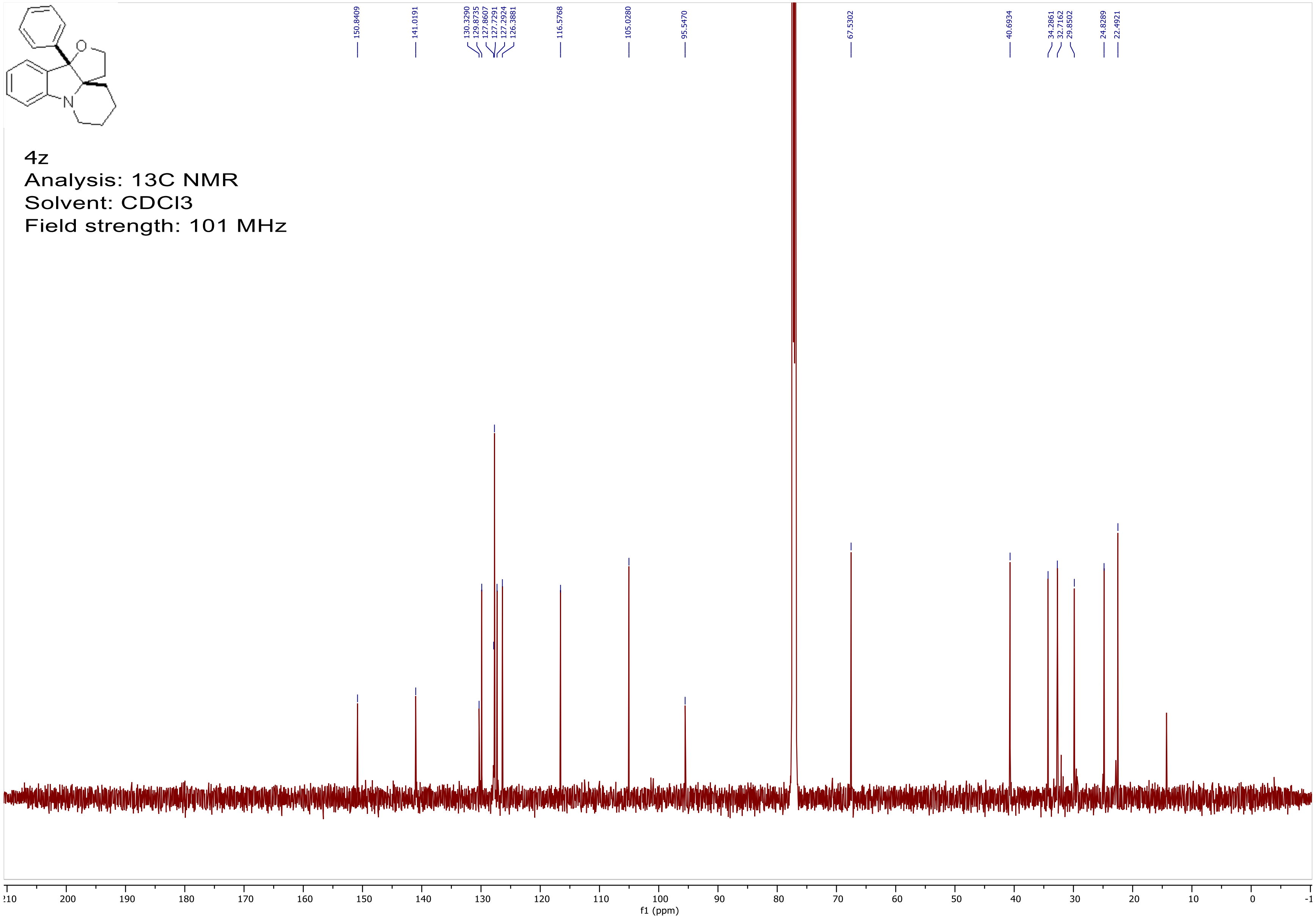
4z
Analysis: 13C NMR
Solvent: CDCl3
Field strength: 101 MHz
150.8409
141.0191
130.3290
129.8735
127.8607
127.7291
127.2924
126.3881
116.5768
105.0280
95.5470
67.5302
40.6934
34.2861
32.7162
29.8502
24.8289
22.4921
f1 (ppm)

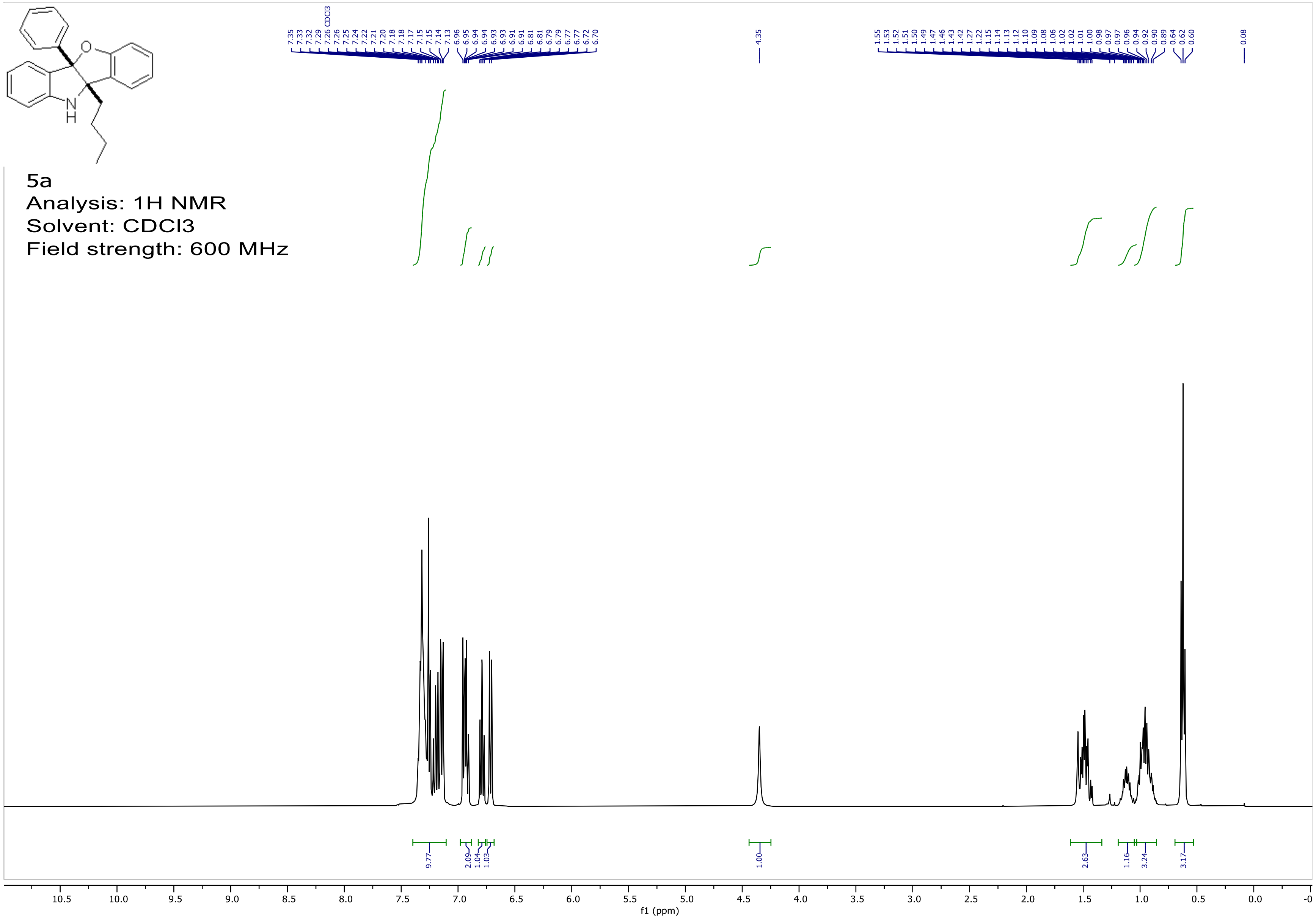
5a
Analysis: 1H NMR
Solvent: CDCl3
Field strength: 600 MHz
7.35 7.33 7.32 7.29 7.26 CDCl3 7.26 7.25 7.24 7.22 7.21 7.20 7.18 7.17 7.15 7.15 7.14 7.13 6.96 6.95 6.94 6.94 6.93 6.93 6.91 6.81 6.79 6.77 6.72 6.70
4.35
1.55 1.53 1.52 1.51 1.50 1.49 1.47 1.46 1.43 1.42 1.27 1.22 1.15 1.14 1.13 1.12 1.10 1.09 1.08 1.06 1.02 1.01 1.00 0.98 0.97 0.96 0.94 0.92 0.90 0.89 0.64 0.62 0.60
0.08
9.77 2.09 1.04 1.03 1.00 2.63 1.16 3.24 3.17
f1 (ppm)

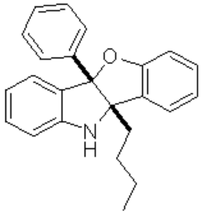

5a
Analysis: 13C NMR
Solvent: CDCl3
Field strength: 151 MHz

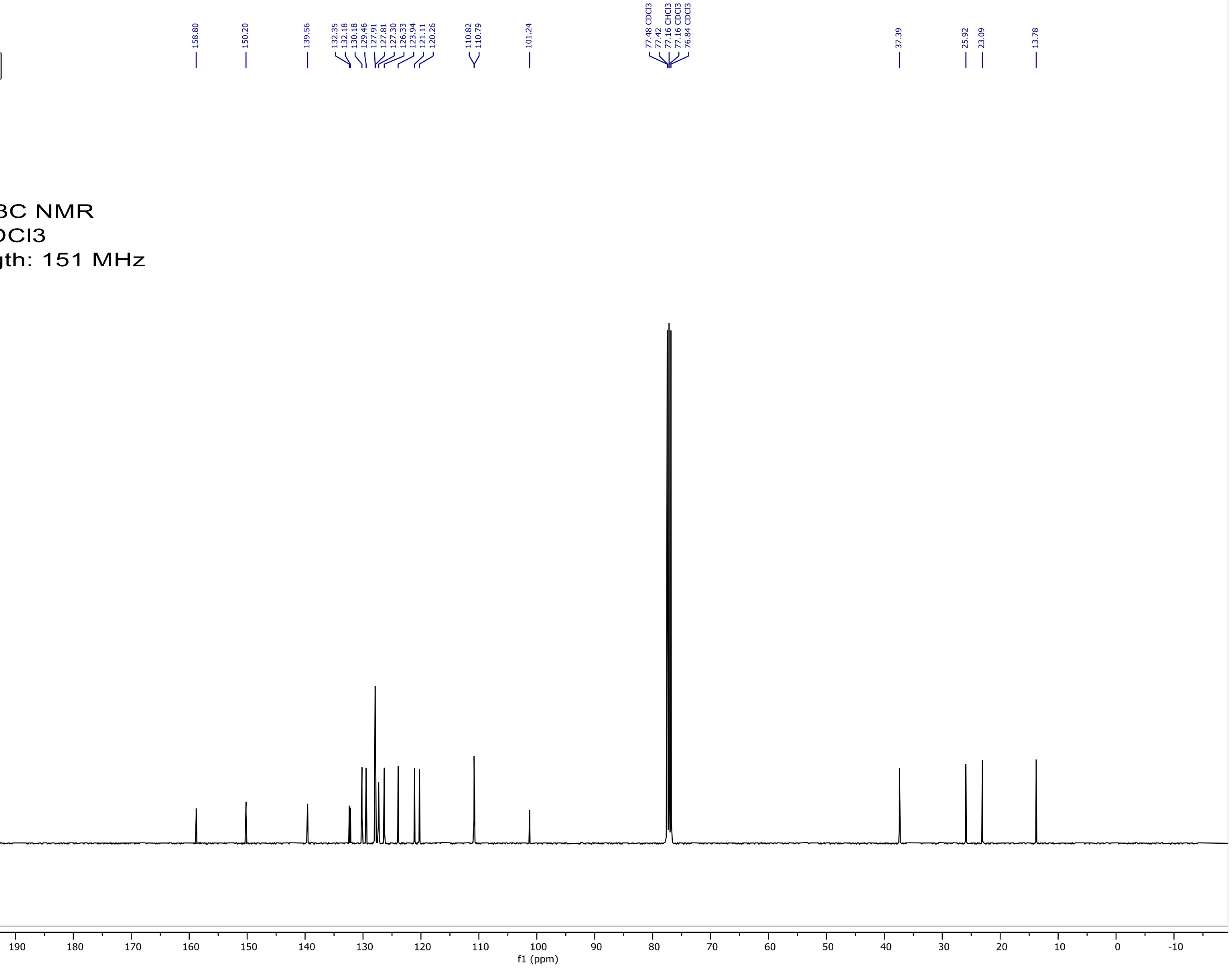

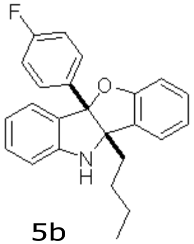

5b
Analysis: 1H NMR
Solvent: CDCl3
Field strength: 400 MHz

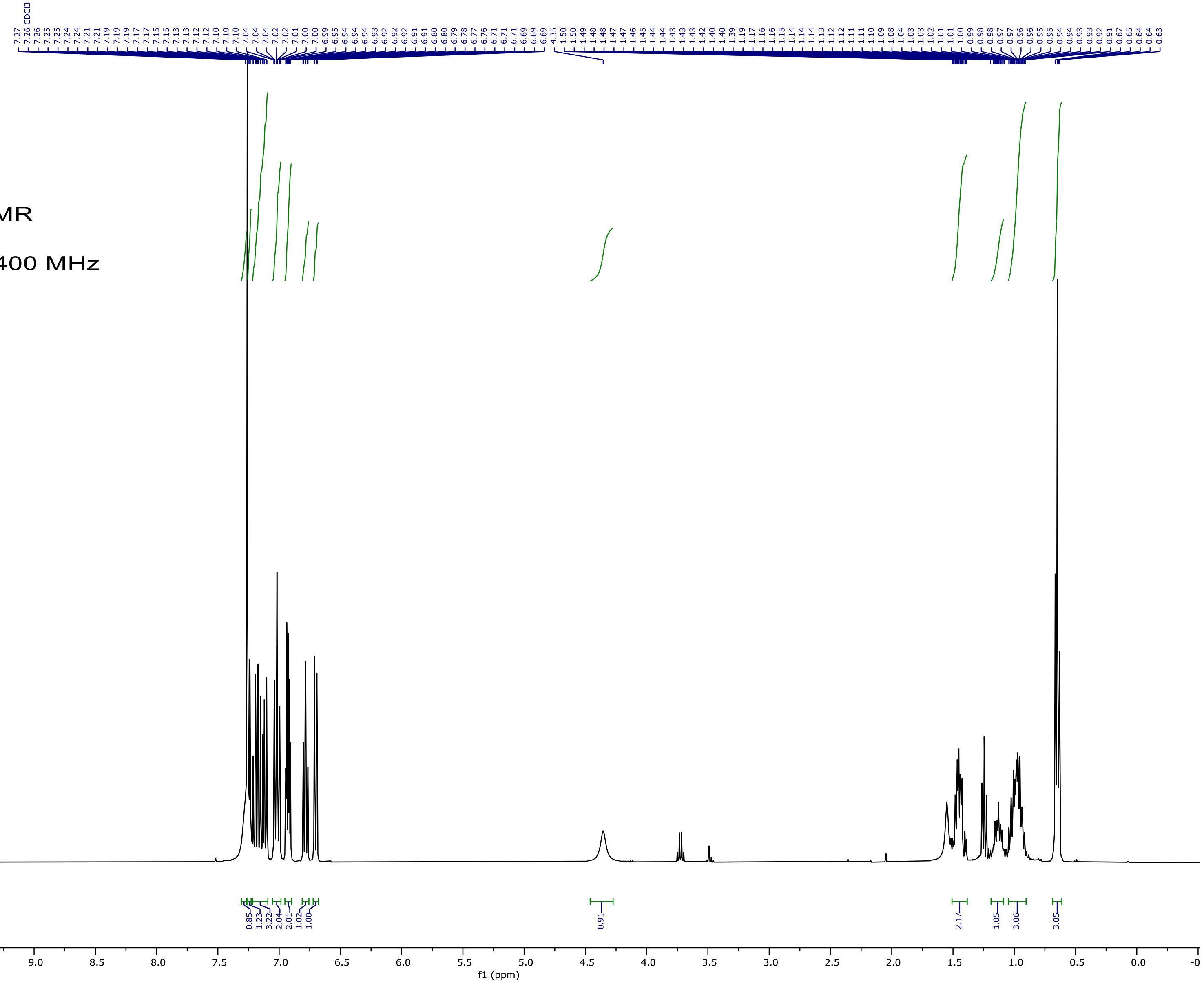

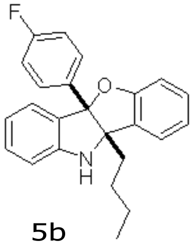

5b
Analysis: 13C NMR
Solvent: CDCl3
Field strength: 101 MHz

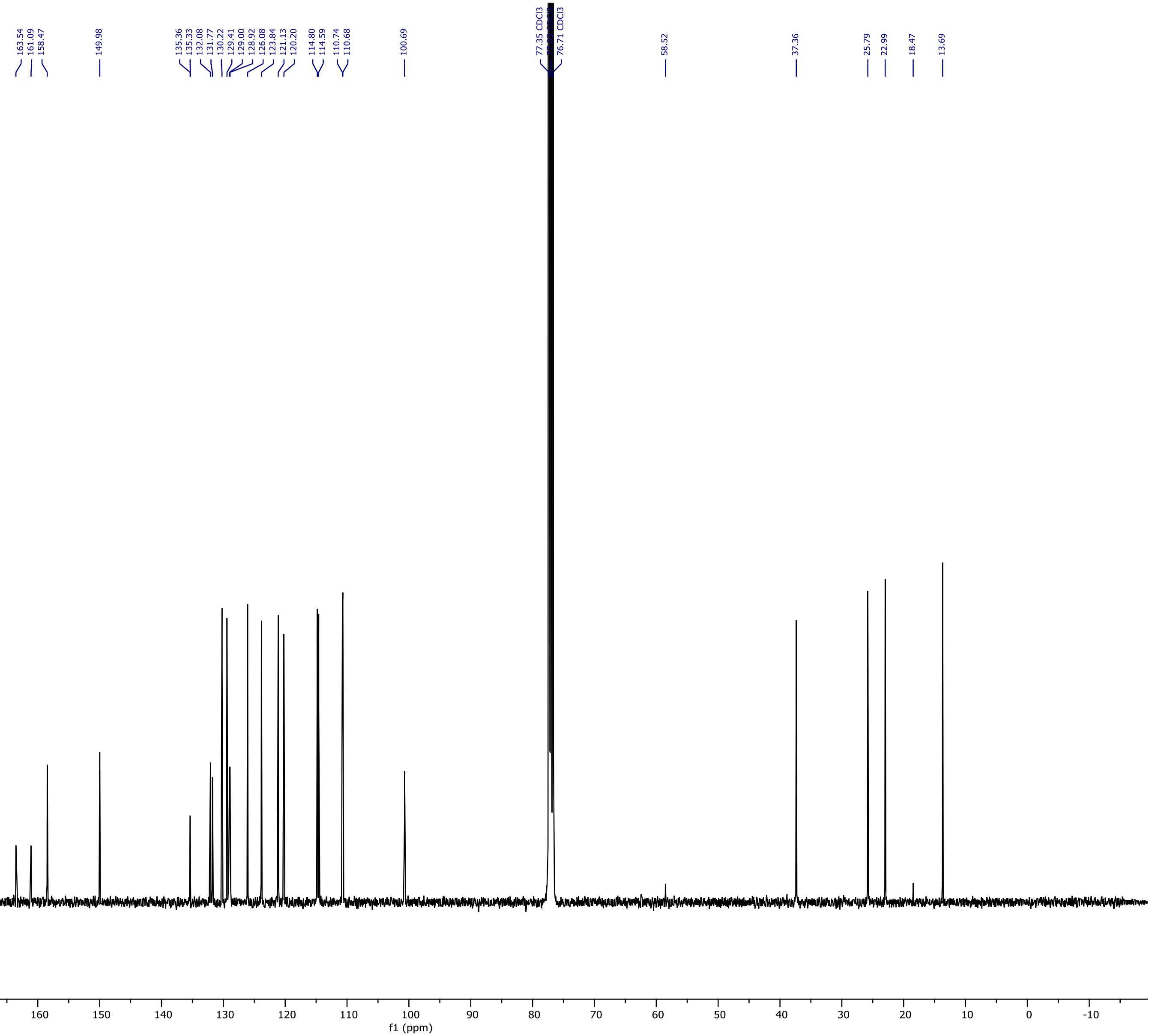

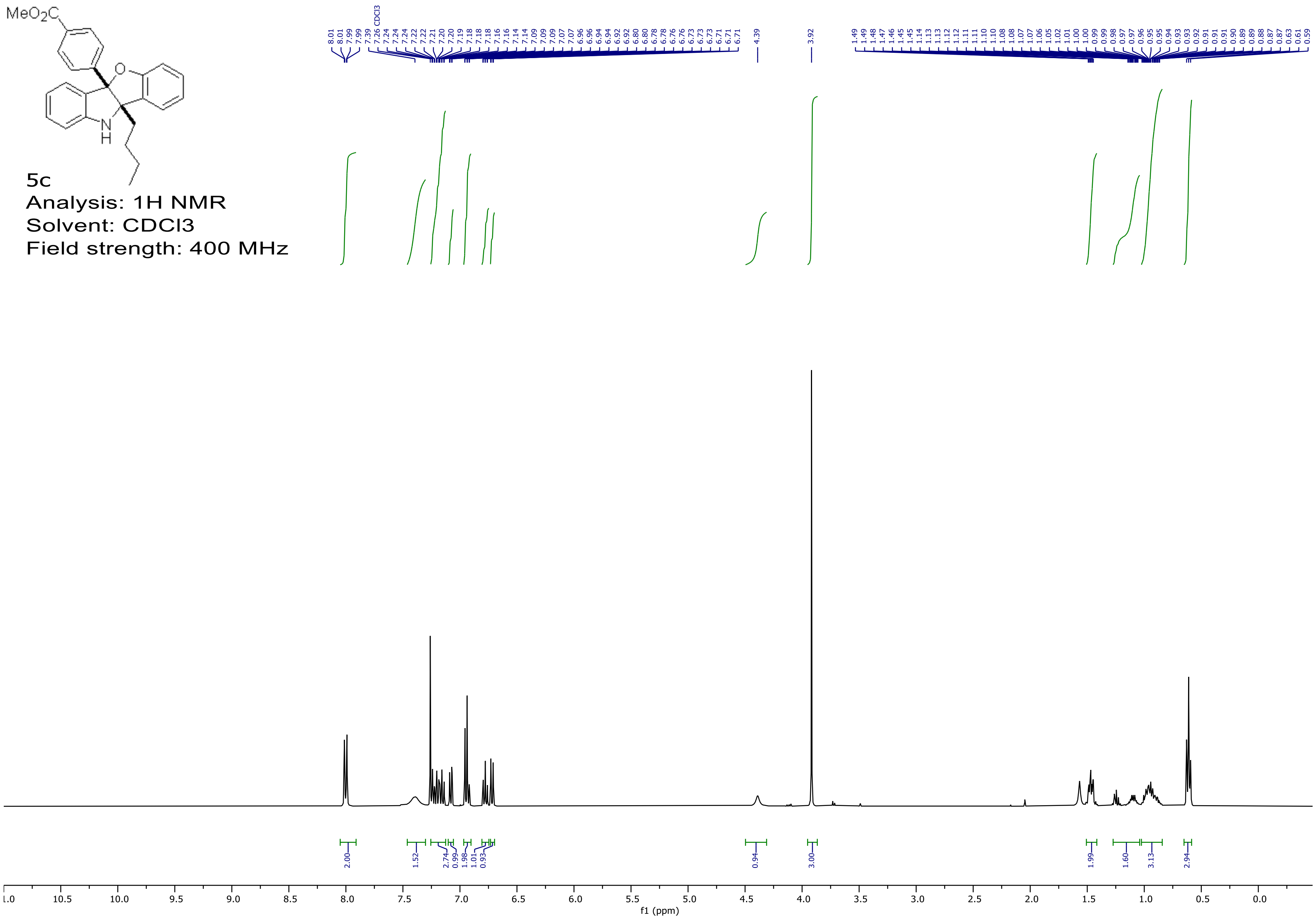
MeO2C
O
N
H
5c
Analysis: 1H NMR
Solvent: CDCl3
Field strength: 400 MHz
f1 (ppm)

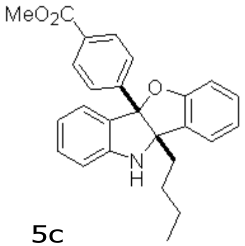

5c
Analysis: 13C NMR
Solvent: CDCl3
Field strength: 101 MHz

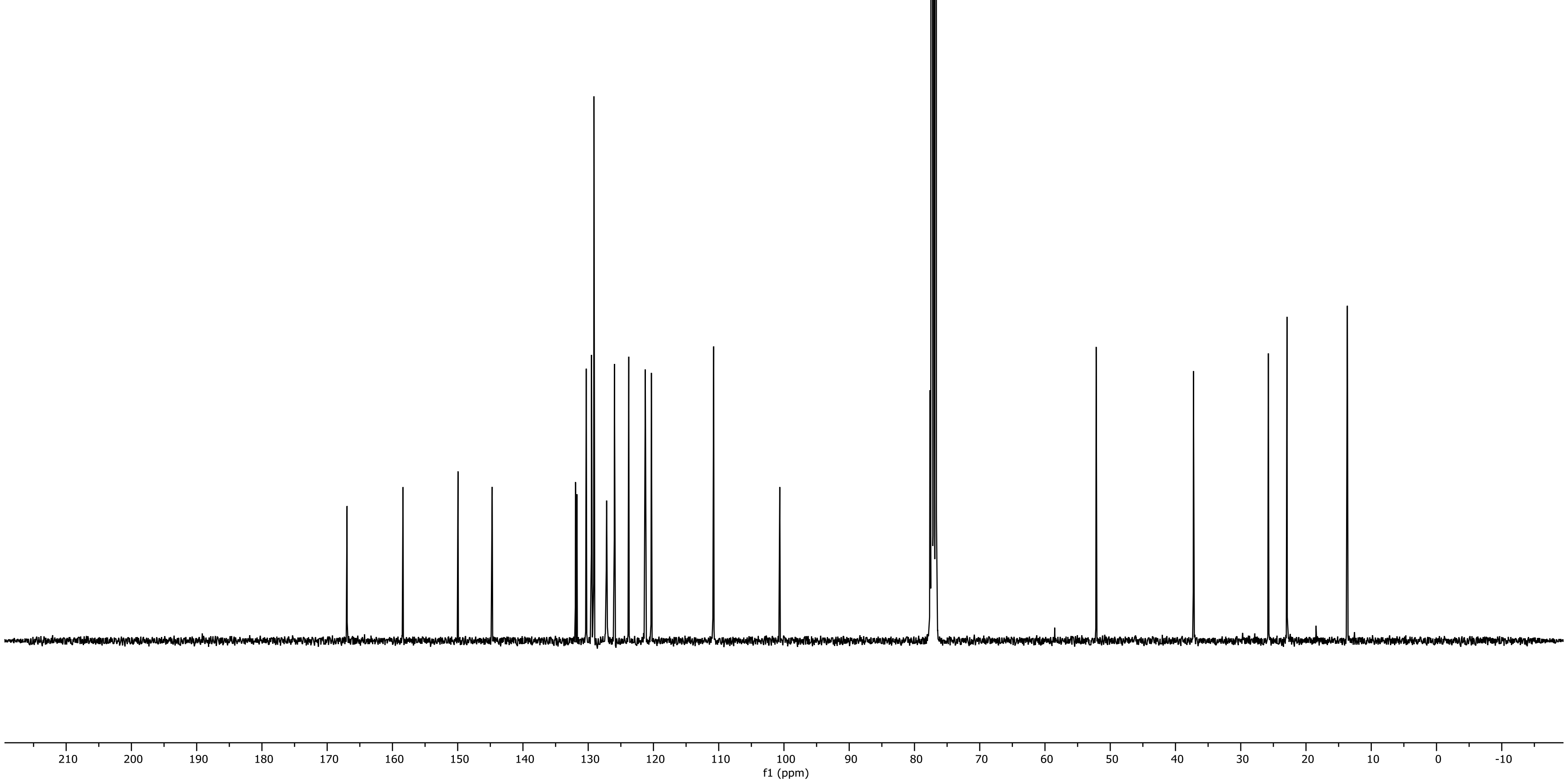

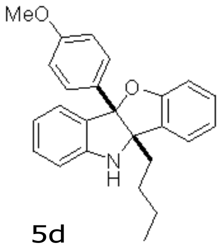

5d
Analysis: 1H NMR
Solvent: CDCl3
Field strength: 400 MHz

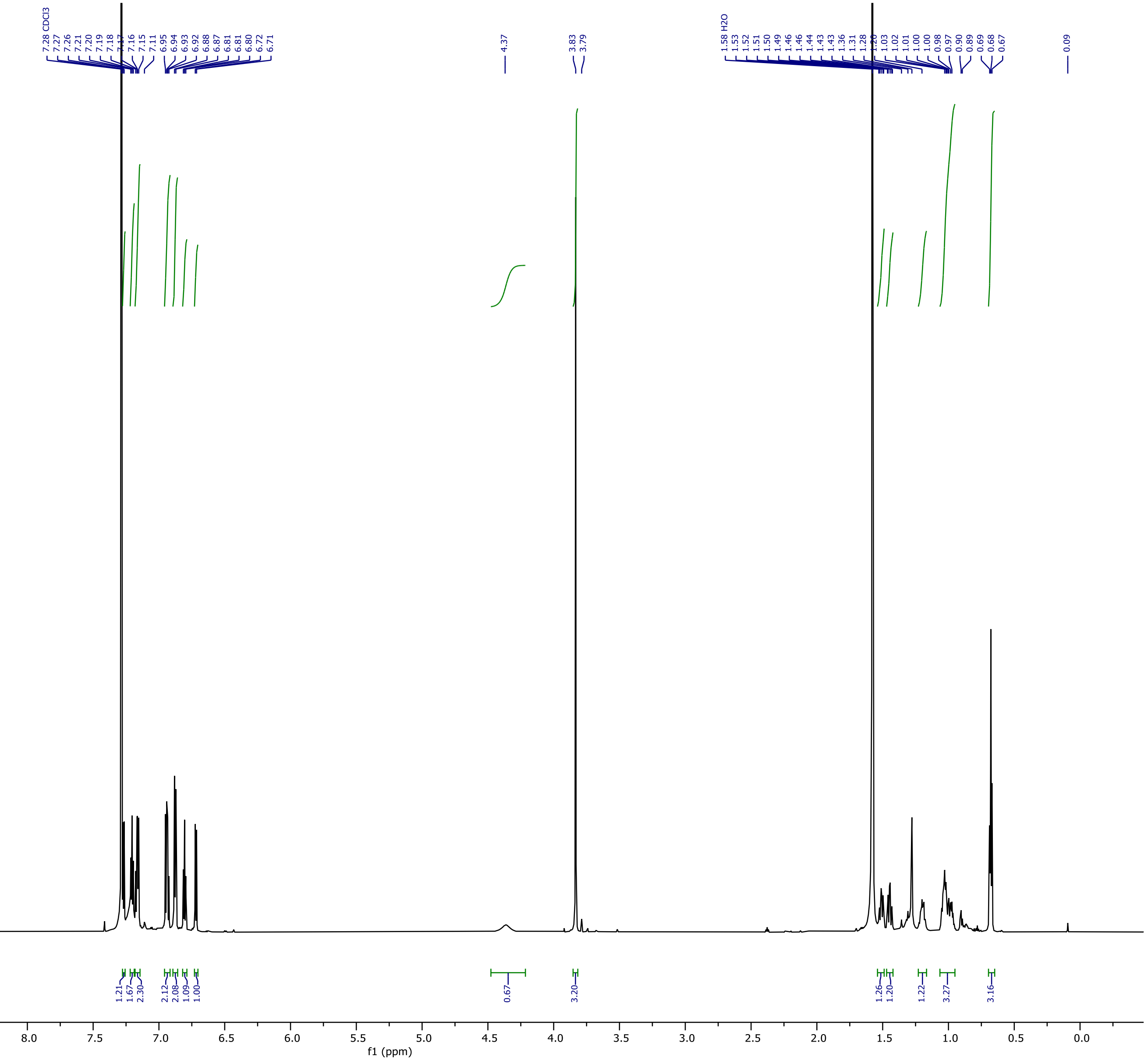

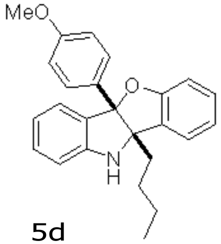

5d

Analysis: 13C NMR

Solvent: CDCl3

Field strength: 101 MHz

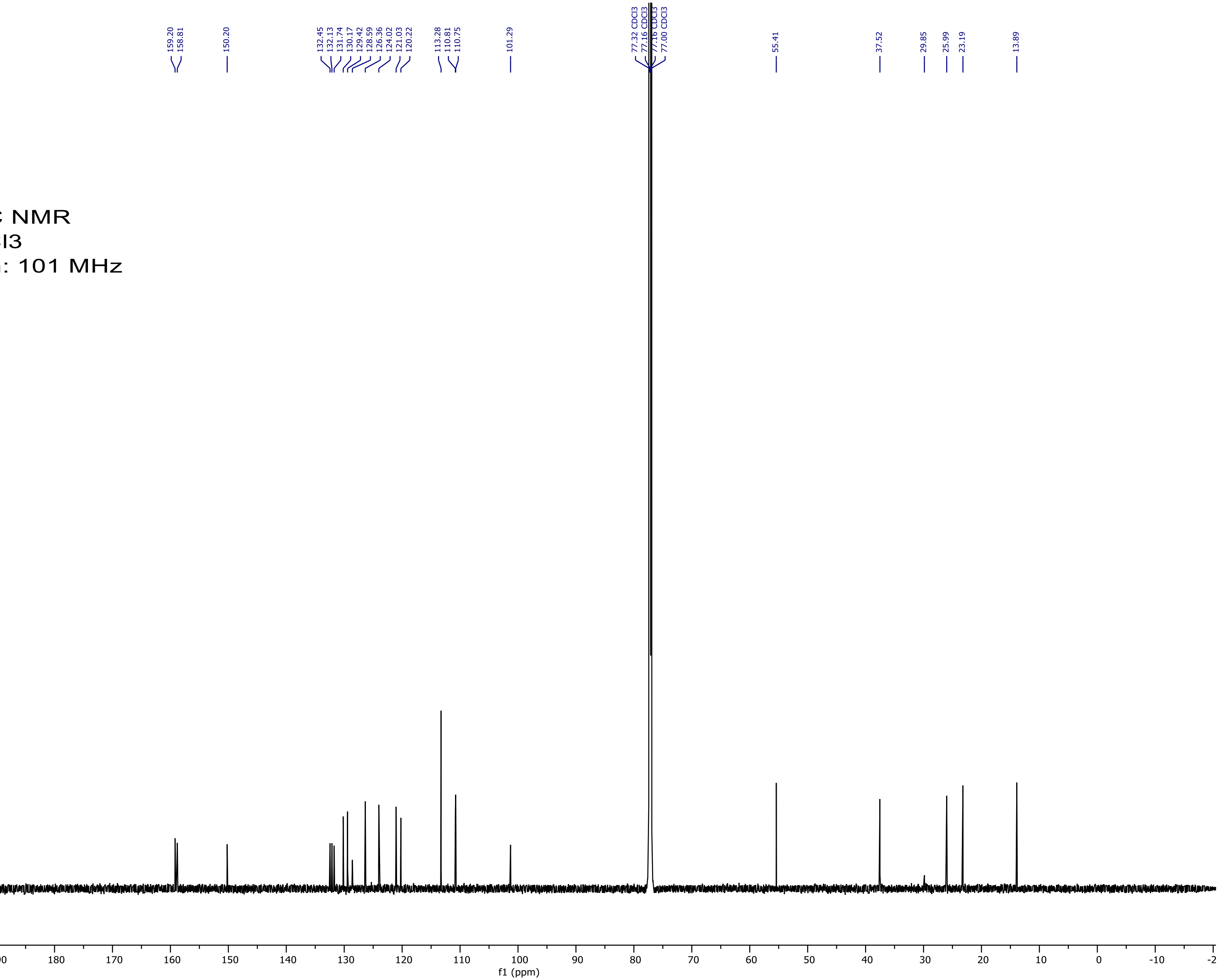

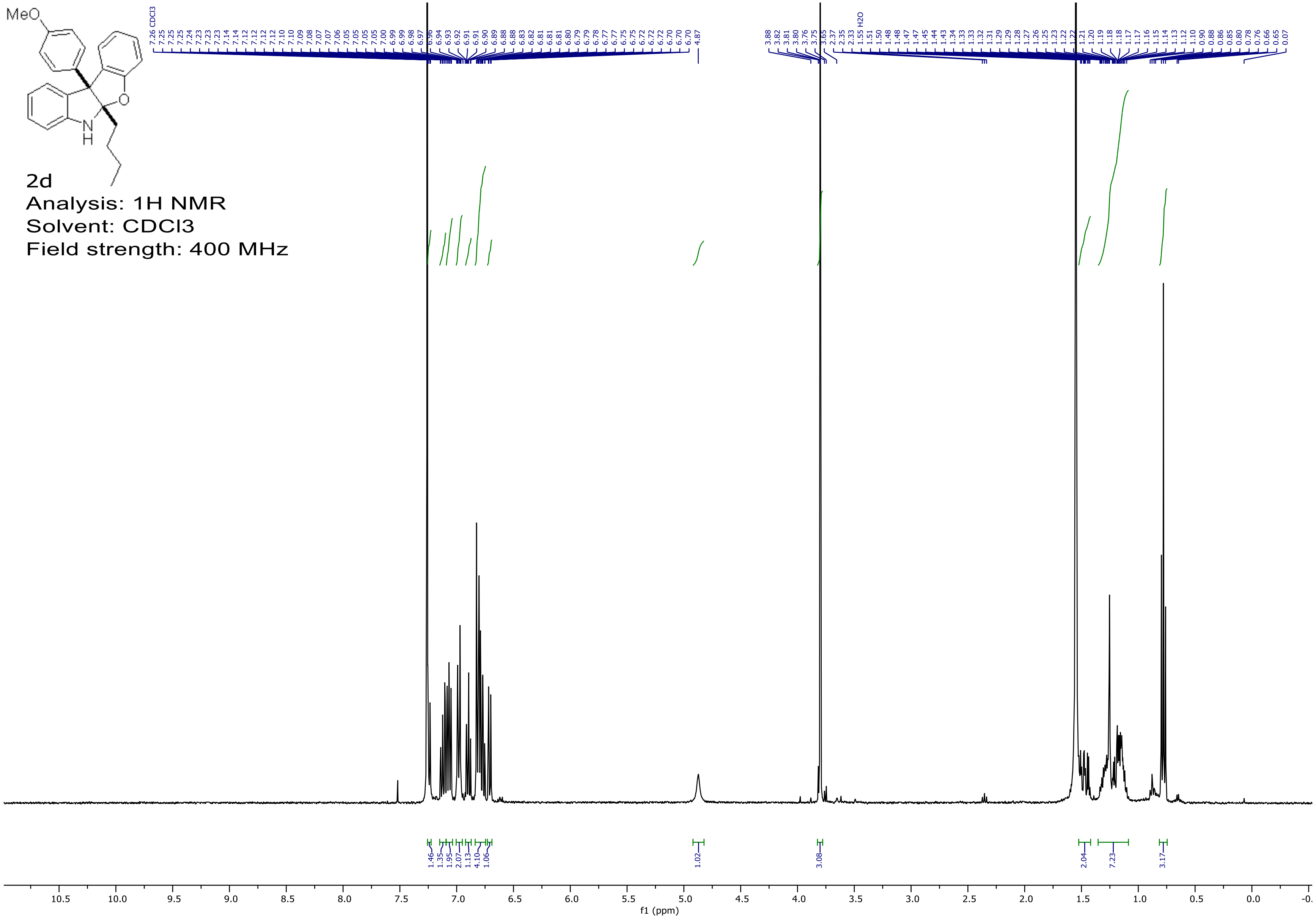
MeO
O
N
H
2d
Analysis: 1H NMR
Solvent: CDCl3
Field strength: 400 MHz
f1 (ppm)

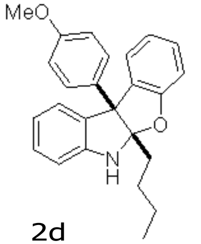

2d
Analysis: 13C NMR
Solvent: CDCl3
Field strength: 101 MHz

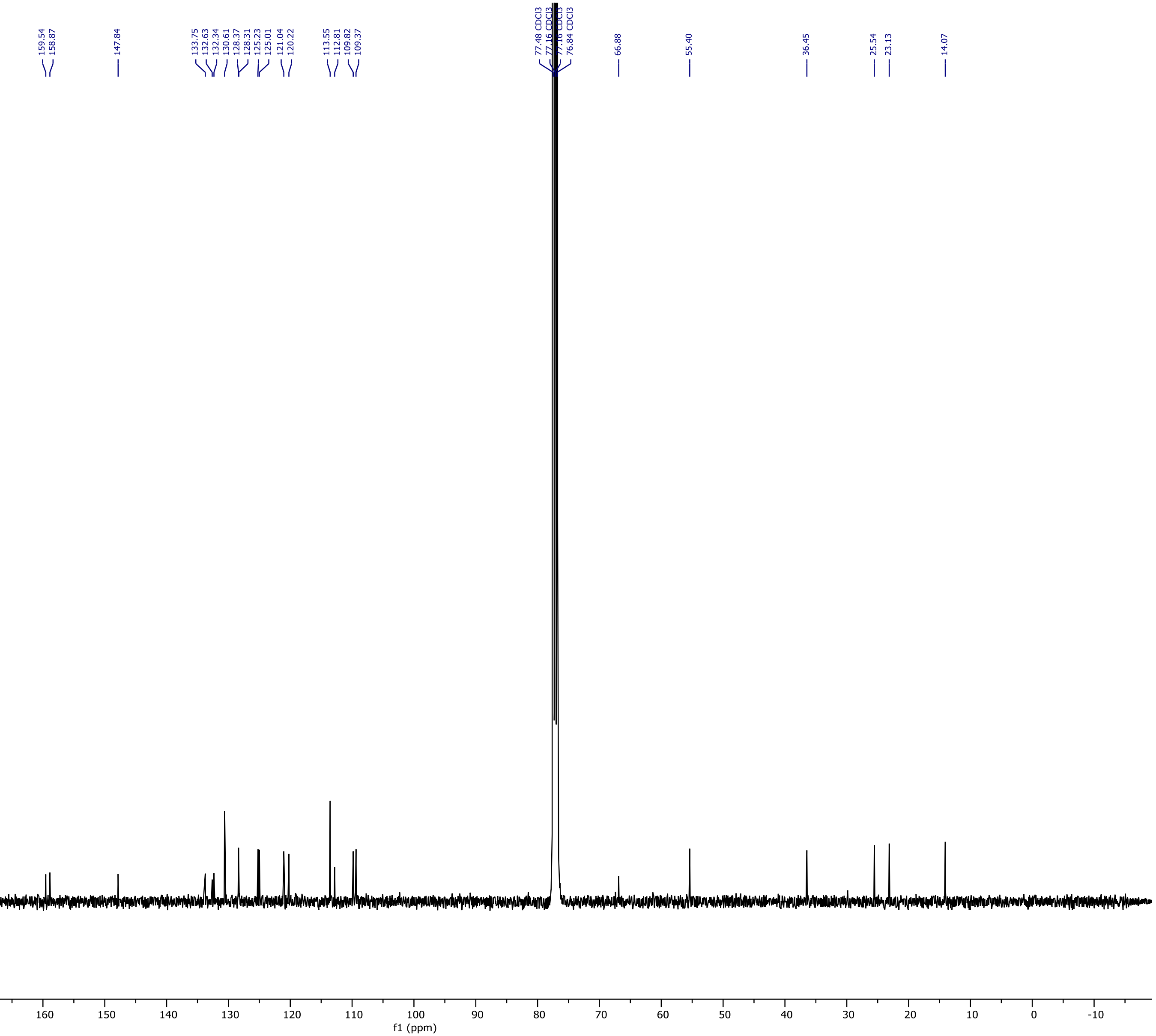

Supplement: Supplementary file 1 [file ol6c01004_si_001.pdf]
